# Supplementary material for: Programmed cell revival from imminent cell death enhances tissue repair and regeneration
Source: EMBO J. 2025 Aug 21;44(19):5244–89. doi: 10.1038/s44318-025-00540-y (PMC12489119; doi:10.1038/s44318-025-00540-y)
Supplement: Supplementary file 1 — Appendix [file 44318_2025_540_MOESM1_ESM.pdf]

Appendix for:

“Programmed cell revival from imminent cell death enhances tissue repair and regeneration”

Table of contents

Appendix Figure S1 2

Appendix Figure S2 3

Appendix Figure S3 4

Appendix Figure S4 5

Appendix Figure S5 6

Appendix Table S1 (Homer analysis). 8

Appendix Table S2 with exact P values for all figures 17

Appendix Figure S1

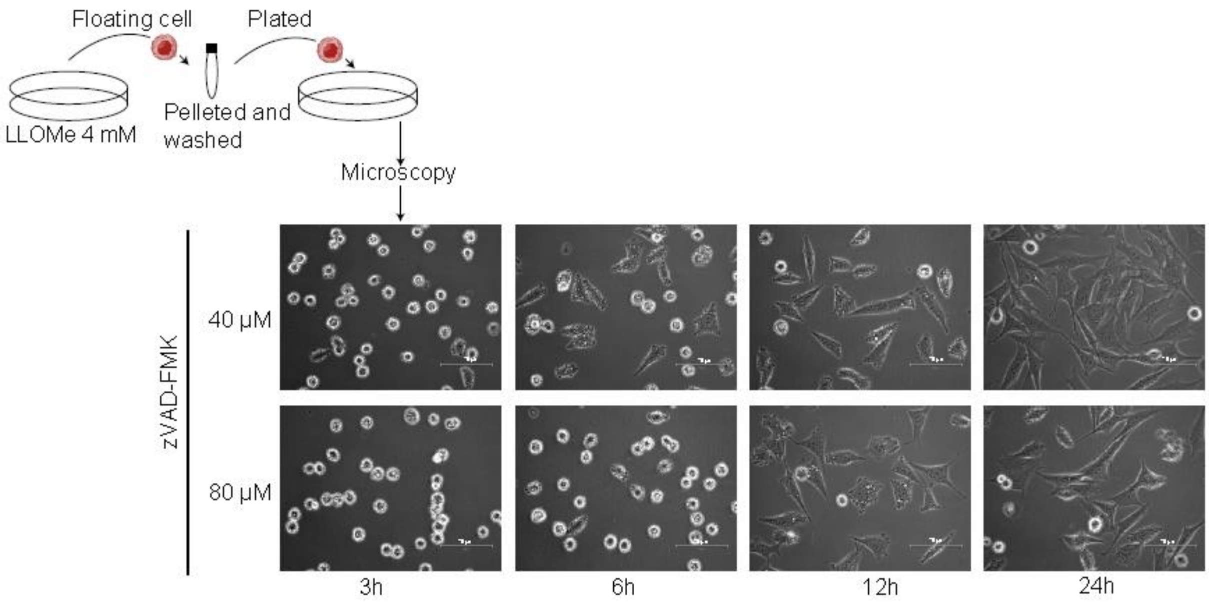

Appendix Figure S1. MEF cells were treated with 4 mM LLOMe. The floating cells were collected and washed with PBS before plating in a new dish with zVAD-FMK. Representative time-lapse live microscopy images of MEF cells after replating. Magnification 40X. Scale bar, 75 µm.

Appendix Figure S2

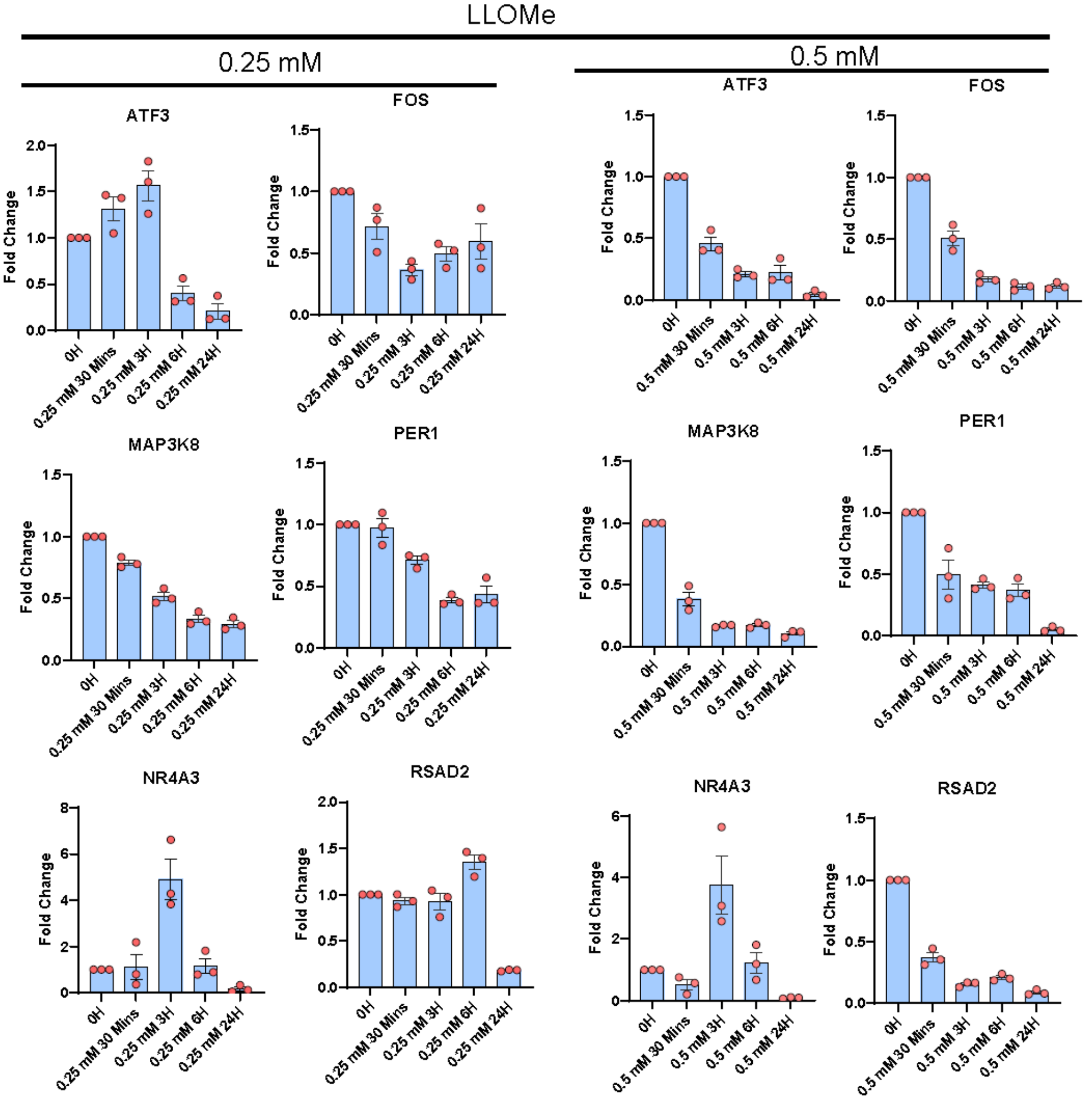

Appendix Figure S2. A qRT-PCR analysis of primary BMDMs treated with LLOMe (0.25 mM or 0.5 mM) for different time points as indicated in the figure (n=3, Mean ± SEM) .

Appendix Figure S3

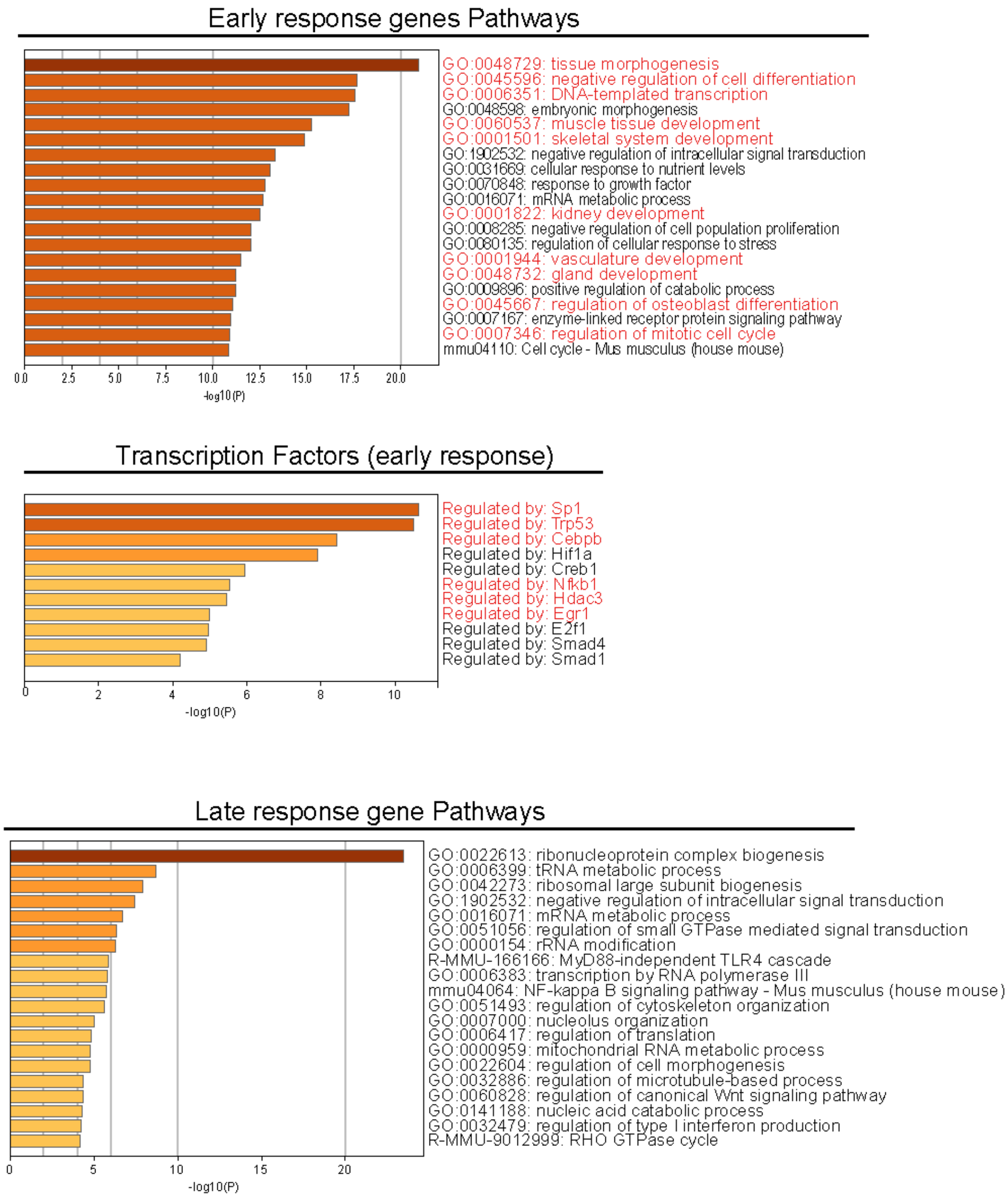

Appendix Figure S3. Metascape analysis of early response genes and late response genes identified by Sun et al., in RNA-seq experiment.

Appendix Figure S4

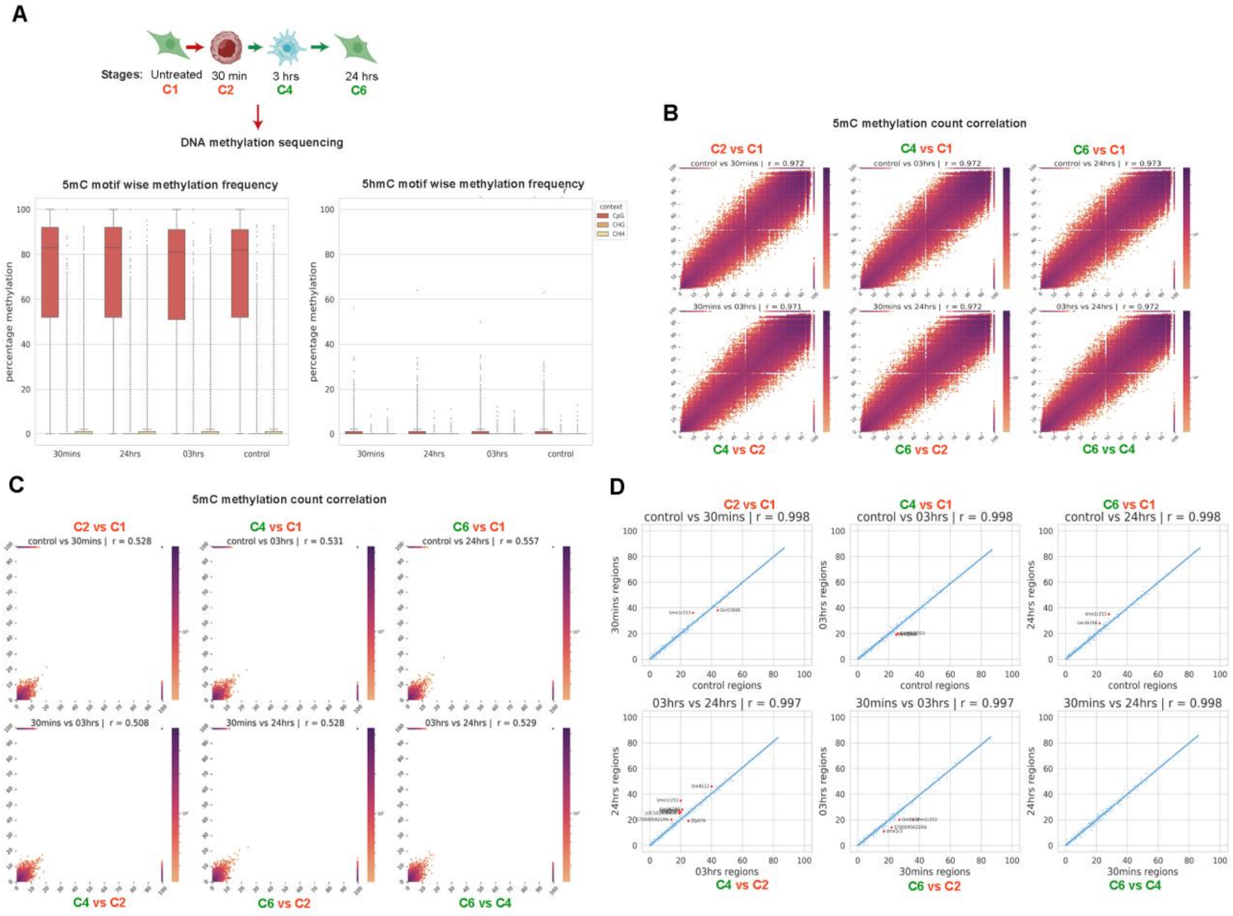

Appendix Figure S4. Genome-wide DNA methylation and ATAC-seq across cell death and revival phases. (A) upper panel, Genome-wide DNA methylation experiment is performed with MEF cells treated with 4 mM LLOMe at the indicated 4 time points (C1, C3, C4, C6). Lower panels, overall distribution of cytosine methylation at C1, C3, C4 and C6 stages, categorized by the sequence context (CpG, CHG, CHH). Left panel: 5-methylcytosine, Right panel: 5-hydroxymethylcytosine.

(B-C) Scatterplots depict the correlation of (B) 5mC levels (C) 5hmC between pair of different stages of samples. The color of each dot indicates the number of cytosines at that methylation level. Only those cytosines which are covered by at least 10 reads across the C1, C3, C4 and C6 stages were included in the analysis.

(D) Average promoter CpG methylation across 4 different stages. In each scatterplot, the promoter methylation level for each gene was plotted for the corresponding pair. Those promoters which showed an absolute

Appendix Figure S5

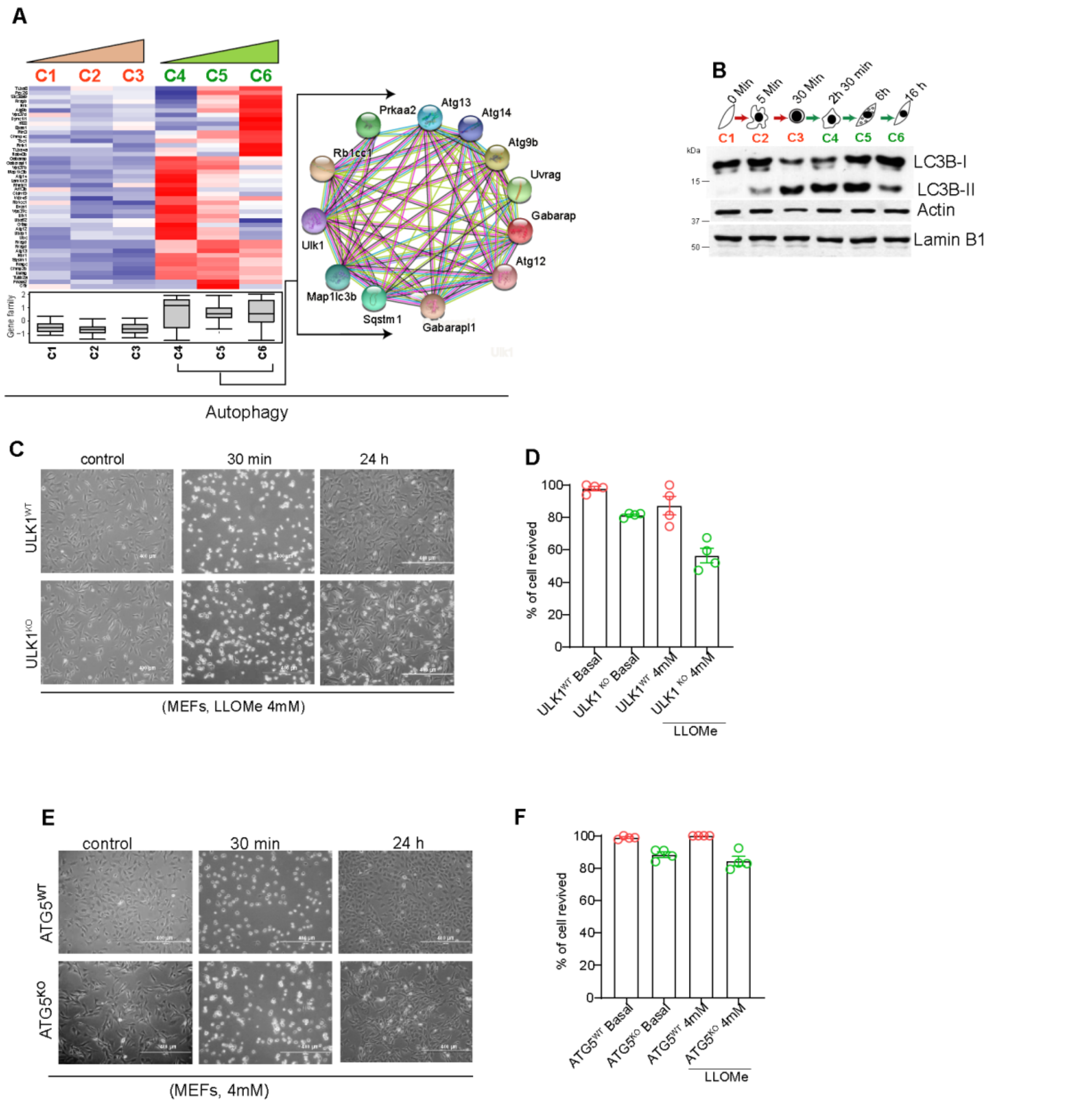

ppendix Figure S5. The status of autophagy during programmed cell revival
A) Heatmap generated for genes representing GO term autophagy, induced ($p \leq 0.05$, > 1.5 folds, base mean >10, =3) in C4 to C6 stages compared to C3 stage. Right panel, STRING analysis of core autophagy genes.
B) Western blot analysis with the lysate of MEF cells treated with 4 mM LLOMe for indicated time points with indicat-d antibodies.
C) Representative time-lapse live microscopy images of $ULK1^{WT}$ and $ULK1^{KO}$ MEF cells untreated or treated with 4 mM LLOMe. Magnification 10X. Scale bar, 400 µm.
D) The graph depicts the percentage of $ULK1^{WT}$ and $ULK1^{KO}$ MEF cells revived from LLOMe treatment.
E) Representative time-lapse live microscopy images of $ATG5^{WT}$ and $ATG5^{KO}$ MEF cells untreated or treated with 4 mM LLOMe. Magnification 10X. Scale bar, 400 µm.
F) Graph depicts the percentage of $ATG5^{WT}$ and $ATG5^{KO}$ MEF cells revived from LLOMe treatment.

Appendix TABLE S1

Appendix Table S1: Transcription factor-specific motif analysis on the significant peaks with increased accessibility at stage C4 compared to C3 (padj < 0.01, log2 fold change > 2) performed using the Homer analysis tool.

Homer Known Motif Enrichment Results (/home/LECD/ATAC_CCMB/Run2_NC_NP/Results/motifanalysis/TREATMENT_3H VS TREATMENT_30M/padj_L_0.01/padj_L_0.01_log2fc_G_1/)

[Homer *de novo* Motif Results](#)
[Gene Ontology Enrichment Results](#)
[Known Motif Enrichment Results (txt file)](#)
Total Target Sequences = 6660, Total Background Sequences = 91619

Rank	Motif	Name	P-value	log P-pvalue	q-value (Benjamini)	# Target Sequences with Motif	% of Targets Sequences with Motif	# Background Sequences with Motif	% of Background Sequences with Motif
1	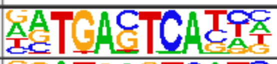	Atf3(bZIP)/GBM-ATF3-ChIP-Seq(GSE33912)/Homer	1e-998	-2.299e+03	0.0000	3163.0	47.49%	12084.6	13.19%
2	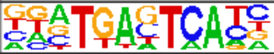	Fos(bZIP)/TSC-Fos-ChIP-Seq(GSE110950)/Homer	1e-996	-2.294e+03	0.0000	2981.0	44.76%	10653.8	11.63%
3	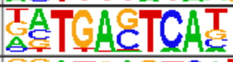	BATF(bZIP)/Th17-BATF-ChIP-Seq(GSE39756)/Homer	1e-980	-2.259e+03	0.0000	3129.0	46.98%	11990.7	13.09%
4	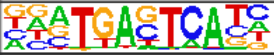	Fra1(bZIP)/BT549-Fra1-ChIP-Seq(GSE46166)/Homer	1e-962	-2.217e+03	0.0000	2859.0	42.93%	10041.5	10.96%
5	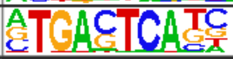	AP-1(bZIP)/ThioMac-PU.1-ChIP-Seq(GSE21512)/Homer	1e-938	-2.161e+03	0.0000	3314.0	49.76%	14041.6	15.33%
6	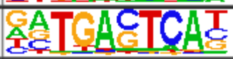	JunB(bZIP)/DendriticCells-Junb-ChIP-Seq(GSE36099)/Homer	1e-929	-2.140e+03	0.0000	2841.0	42.66%	10232.4	11.17%
7	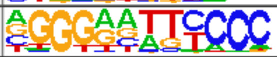	NFkB2-p52(RHD)/U266-NFKB2-ChIP-seq(GSE230466)/Homer	1e-898	-2.068e+03	0.0000	2103.0	31.58%	5441.3	5.94%
8	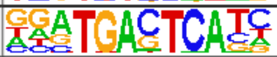	Fra2(bZIP)/Striatum-Fra2-ChIP-Seq(GSE43429)/Homer	1e-880	-2.028e+03	0.0000	2599.0	39.02%	8888.4	9.70%
9	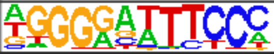	NFkB-p65(RHD)/GM12787-p65-ChIP-Seq(GSE19485)/Homer	1e-864	-1.990e+03	0.0000	2575.0	38.66%	8866.4	9.68%
10	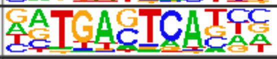	Fosl2(bZIP)/3T3L1-Fosl2-ChIP-Seq(GSE56872)/Homer	1e-734	-1.692e+03	0.0000	2047.0	30.74%	6345.6	6.93%
11	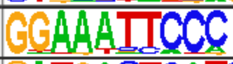	NFkB-p65-Rel(RHD)/ThioMac-LPS-Expression(GSE23622)/Homer	1e-677	-1.561e+03	0.0000	894.0	13.42%	907.7	0.99%
12	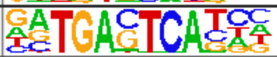	Jun-AP1(bZIP)/K562-cJun-ChIP-Seq(GSE31477)/Homer	1e-574	-1.323e+03	0.0000	1543.0	23.17%	4444.6	4.85%
13	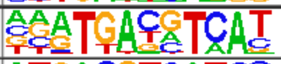	CREB5(bZIP)/LNCaP-CREB5.V5-ChIP-Seq(GSE137775)/Homer	1e-498	-1.147e+03	0.0000	1842.0	27.66%	7248.2	7.91%
14	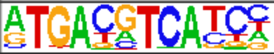	c-Jun-CRE(bZIP)/K562-cJun-ChIP-Seq(GSE31477)/Homer	1e-479	-1.103e+03	0.0000	1666.0	25.02%	6159.7	6.72%
15	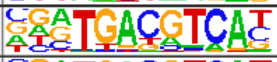	Atf7(bZIP)/3T3L1-Atf7-ChIP-Seq(GSE56872)/Homer	1e-465	-1.072e+03	0.0000	2053.0	30.83%	9351.7	10.21%
16	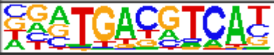	Atf2(bZIP)/3T3L1-Atf2-ChIP-Seq(GSE56872)/Homer	1e-441	-1.018e+03	0.0000	1694.0	25.44%	6792.5	7.41%
17	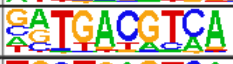	Atf1(bZIP)/K562-ATF1-ChIP-Seq(GSE31477)/Homer	1e-328	-7.573e+02	0.0000	2157.0	32.39%	12585.1	13.74%
18	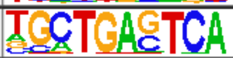	Bach2(bZIP)/OCILy7-Bach2-ChIP-Seq(GSE44420)/Homer	1e-322	-7.435e+02	0.0000	1079.0	16.20%	3714.2	4.05%
19	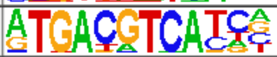	JunD(bZIP)/K562-JunD-ChIP-Seq/Homer	1e-281	-6.481e+02	0.0000	720.0	10.81%	1868.0	2.04%
20	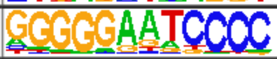	NFkB-p50,p52(RHD)/Monocyte-p50-ChIP-Chip(Schreiber_et_al.)/Homer	1e-244	-5.626e+02	0.0000	592.0	8.89%	1435.3	1.57%
21	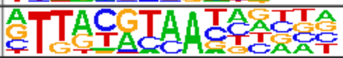	NFIL3(bZIP)/HepG2-NFIL3-ChIP-Seq(Encode)/Homer	1e-136	-3.132e+02	0.0000	1356.0	20.36%	9236.0	10.08%
22	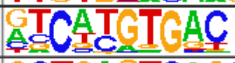	MITF(bHLH)/MastCells-MITF-ChIP-Seq(GSE48085)/Homer	1e-128	-2.960e+02	0.0000	2255.0	33.86%	19284.8	21.05%
23	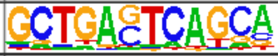	MafK(bZIP)/C2C12-MafK-ChIP-Seq(GSE36030)/Homer	1e-103	-2.382e+02	0.0000	772.0	11.59%	4495.4	4.91%
24	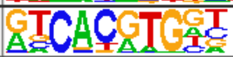	Usf2(bHLH)/C2C12-Usf2-ChIP-Seq(GSE36030)/Homer	1e-101	-2.345e+02	0.0000	919.0	13.80%	5892.7	6.43%
25	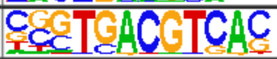	CRE(bZIP)/Promoter/Homer	1e-99	-2.291e+02	0.0000	680.0	10.21%	3768.2	4.11%
26	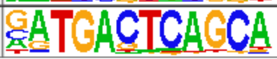	NF-E2(bZIP)/K562-NFE2-ChIP-Seq(GSE31477)/Homer	1e-99	-2.280e+02	0.0000	331.0	4.97%	1098.0	1.20%

27	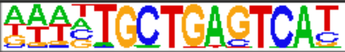	Bach1(bZIP)/K562-Bach1-ChIP-Seq(GSE31477)/Homer	1e-93	-2.147e+02	0.0000	302.0	4.53%	973.6	1.06%
28	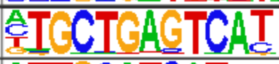	Nrf2(bZIP)/Lymphoblast-Nrf2-ChIP-Seq(GSE37589)/Homer	1e-92	-2.131e+02	0.0000	280.0	4.20%	846.5	0.92%
29	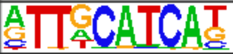	Chop(bZIP)/MEF-Chop-ChIP-Seq(GSE35681)/Homer	1e-85	-1.958e+02	0.0000	601.0	9.02%	3381.5	3.69%
30	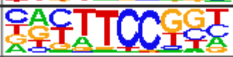	Fli1(ETS)/CD8-FLI-ChIP-Seq(GSE20898)/Homer	1e-84	-1.956e+02	0.0000	2089.0	31.37%	19312.3	21.08%
31	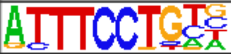	EWS:ERG-fusion(ETS)/CADO_ES1-EWS:ERG-ChIP-Seq(SRA014231)/Homer	1e-82	-1.892e+02	0.0000	1602.0	24.05%	13768.7	15.03%
32	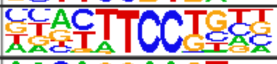	Etv2(ETS)/ES-ER71-ChIP-Seq(GSE59402)/Homer	1e-80	-1.860e+02	0.0000	1963.0	29.47%	18025.2	19.67%
33	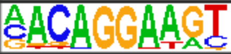	Ets1-distal(ETS)/CD4+-PolII-ChIP-Seq(Barski_et_al.)/Homer	1e-80	-1.855e+02	0.0000	937.0	14.07%	6673.5	7.28%
34	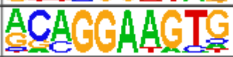	ETS1(ETS)/Jurkat-ETS1-ChIP-Seq(GSE17954)/Homer	1e-79	-1.837e+02	0.0000	2092.0	31.41%	19618.0	21.41%
35	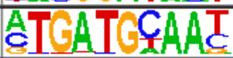	Atf4(bZIP)/MEF-Atf4-ChIP-Seq(GSE35681)/Homer	1e-77	-1.779e+02	0.0000	674.0	10.12%	4218.3	4.60%
36	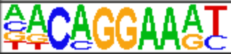	EWS:FLI1-fusion(ETS)/SK_N_MC-EWS:FLI1-ChIP-Seq(SRA014231)/Homer	1e-77	-1.775e+02	0.0000	1310.0	19.67%	10730.4	11.71%
37	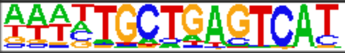	NFE2L2(bZIP)/HepG2-NFE2L2-ChIP-Seq(Encode)/Homer	1e-75	-1.748e+02	0.0000	259.0	3.89%	874.6	0.95%
38	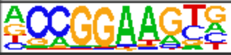	ETV4(ETS)/HepG2-ETV4-ChIP-Seq(ENCODE)/Homer	1e-71	-1.647e+02	0.0000	2063.0	30.98%	19718.8	21.52%
39	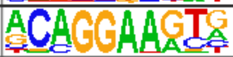	ERG(ETS)/VCaP-ERG-ChIP-Seq(GSE14097)/Homer	1e-69	-1.608e+02	0.0000	2873.0	43.14%	29974.8	32.72%
40	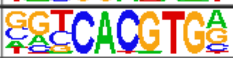	USF1(bHLH)/GM12878-Usf1-ChIP-Seq(GSE32465)/Homer	1e-69	-1.600e+02	0.0000	1005.0	15.09%	7750.6	8.46%
41	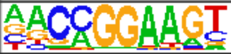	ETV1(ETS)/GIST48-ETV1-ChIP-Seq(GSE22441)/Homer	1e-67	-1.563e+02	0.0000	2485.0	37.31%	25150.2	27.45%
42	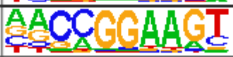	GABPA(ETS)/Jurkat-GABPa-ChIP-Seq(GSE17954)/Homer	1e-59	-1.365e+02	0.0000	1749.0	26.26%	16644.8	18.17%
43	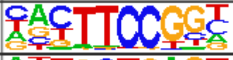	Elk1(ETS)/Hela-Elk1-ChIP-Seq(GSE31477)/Homer	1e-55	-1.273e+02	0.0000	998.0	14.98%	8242.4	9.00%
44	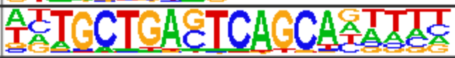	MafB(bZIP)/BMM-Mafb-ChIP-Seq(GSE75722)/Homer	1e-52	-1.218e+02	0.0000	971.0	14.58%	8051.4	8.79%
45	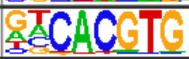	CLOCK(bHLH)/Liver-Clock-ChIP-Seq(GSE39860)/Homer	1e-51	-1.180e+02	0.0000	1147.0	17.22%	10079.7	11.00%
46	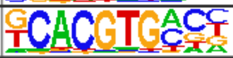	bHLHE41(bHLH)/proB-Bhlhe41-ChIP-Seq(GSE93764)/Homer	1e-45	-1.059e+02	0.0000	1742.0	26.16%	17392.5	18.98%
47	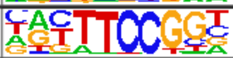	Elk4(ETS)/Hela-Elk4-ChIP-Seq(GSE31477)/Homer	1e-45	-1.058e+02	0.0000	925.0	13.89%	7862.8	8.58%
48	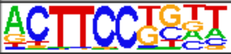	Elf4(ETS)/BMDM-Elf4-ChIP-Seq(GSE88699)/Homer	1e-44	-1.027e+02	0.0000	1883.0	28.27%	19219.7	20.98%
49	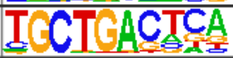	MafA(bZIP)/Islet-MafA-ChIP-Seq(GSE30298)/Homer	1e-43	-1.011e+02	0.0000	1714.0	25.74%	17192.5	18.77%
50	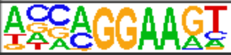	EHF(ETS)/LoVo-EHF-ChIP-Seq(GSE49402)/Homer	1e-43	-1.003e+02	0.0000	2238.0	33.60%	23746.2	25.92%
51	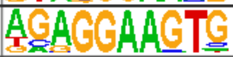	PU.1(ETS)/ThioMac-PU.1-ChIP-Seq(GSE21512)/Homer	1e-42	-9.855e+01	0.0000	1069.0	16.05%	9632.6	10.51%
52	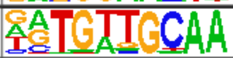	CEBP:AP1(bZIP)/ThioMac-CEBPb-ChIP-Seq(GSE21512)/Homer	1e-41	-9.633e+01	0.0000	1285.0	19.29%	12186.1	13.30%
53	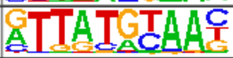	HLF(bZIP)/HSC-HLF.Flag-ChIP-Seq(GSE69817)/Homer	1e-41	-9.580e+01	0.0000	1277.0	19.17%	12106.7	13.21%
54	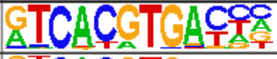	TFE3(bHLH)/MEF-TFE3-ChIP-Seq(GSE75757)/Homer	1e-40	-9.343e+01	0.0000	277.0	4.16%	1520.7	1.66%
55	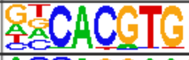	BMAL1(bHLH)/Liver-Bmal1-ChIP-Seq(GSE39860)/Homer	1e-37	-8.699e+01	0.0000	3048.0	45.77%	34805.1	37.99%
56	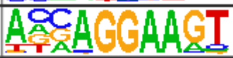	ELF3(ETS)/PDAC-ELF3-ChIP-Seq(GSE64557)/Homer	1e-36	-8.339e+01	0.0000	1412.0	21.20%	14064.8	15.35%
57	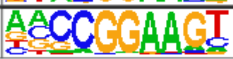	ELF1(ETS)/Jurkat-ELF1-ChIP-Seq(SRA014231)/Homer	1e-33	-7.682e+01	0.0000	888.0	13.33%	8090.3	8.83%

58	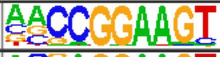	ETS(ETS)/Promoter/Homer	1e-33	-7.646e+01	0.0000	628.0	9.43%	5212.4	5.69%
59	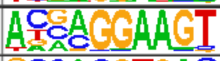	ELF5(ETS)/T47D-ELF5-ChIP-Seq(GSE30407)/Homer	1e-30	-7.116e+01	0.0000	1340.0	20.12%	13579.0	14.82%
60	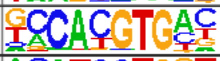	NPAS2(bHLH)/Liver-NPAS2-ChIP-Seq(GSE39860)/Homer	1e-29	-6.890e+01	0.0000	1968.0	29.55%	21474.3	23.44%
61	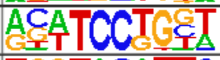	SPDEF(ETS)/VCaP-SPDEF-ChIP-Seq(SRA014231)/Homer	1e-28	-6.540e+01	0.0000	1830.0	27.48%	19864.1	21.68%
62	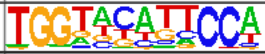	PRDM10(Zf)/HEK293-PRDM10.eGFP-ChIP-Seq(Encode)/Homer	1e-25	-5.905e+01	0.0000	1193.0	17.91%	12200.7	13.32%
63	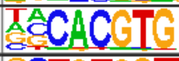	NPAS(bHLH)/Liver-NPAS-ChIP-Seq(GSE39860)/Homer	1e-25	-5.838e+01	0.0000	2585.0	38.81%	29948.0	32.69%
64	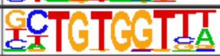	RUNX-AML(Runt)/CD4+-PolII-ChIP-Seq(Barski_et_al.)/Homer	1e-24	-5.729e+01	0.0000	1391.0	20.89%	14689.5	16.03%
65	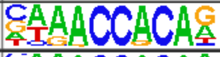	RUNX(Runt)/HPC7-Runx1-ChIP-Seq(GSE22178)/Homer	1e-24	-5.669e+01	0.0000	1343.0	20.17%	14119.5	15.41%
66	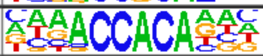	RUNX2(Runt)/PCa-RUNX2-ChIP-Seq(GSE33889)/Homer	1e-24	-5.573e+01	0.0000	1568.0	23.54%	16955.3	18.51%
67	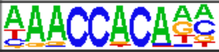	RUNX1(Runt)/Jurkat-RUNX1-ChIP-Seq(GSE29180)/Homer	1e-21	-5.034e+01	0.0000	1758.0	26.40%	19592.1	21.38%
68	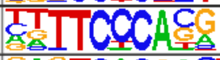	Rbpj1(?)/Panc1-Rbpj1-ChIP-Seq(GSE47459)/Homer	1e-21	-4.907e+01	0.0000	2441.0	36.65%	28521.7	31.13%
69	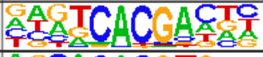	Npas4(bHLH)/Neuron-Npas4-ChIP-Seq(GSE127793)/Homer	1e-20	-4.724e+01	0.0000	1043.0	15.66%	10797.7	11.79%
70	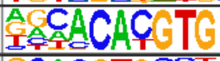	MNT(bHLH)/HepG2-MNT-ChIP-Seq(Encode)/Homer	1e-19	-4.406e+01	0.0000	1820.0	27.33%	20687.6	22.58%
71	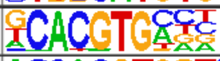	bHLHE40(bHLH)/HepG2-BHLHE40-ChIP-Seq(GSE31477)/Homer	1e-19	-4.393e+01	0.0000	504.0	7.57%	4562.7	4.98%
72	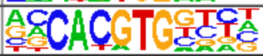	Max(bHLH)/K562-Max-ChIP-Seq(GSE31477)/Homer	1e-18	-4.265e+01	0.0000	1132.0	17.00%	12064.1	13.17%
73	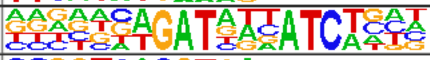	GATA(Zf),IR3/iTreg-Gata3-ChIP-Seq(GSE20898)/Homer	1e-17	-4.088e+01	0.0000	320.0	4.80%	2617.8	2.86%
74	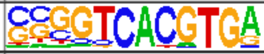	E-box(bHLH)/Promoter/Homer	1e-16	-3.876e+01	0.0000	160.0	2.40%	1042.6	1.14%
75	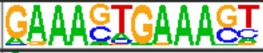	IRF1(IRF)/PBMC-IRF1-ChIP-Seq(GSE43036)/Homer	1e-16	-3.786e+01	0.0000	249.0	3.74%	1931.4	2.11%
76	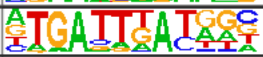	PBX2(Homeobox)/K562-PBX2-ChIP-Seq(Encode)/Homer	1e-15	-3.568e+01	0.0000	1311.0	19.68%	14610.8	15.95%
77	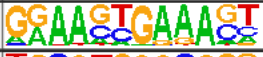	IRF8(IRF)/BMDM-IRF8-ChIP-Seq(GSE77884)/Homer	1e-15	-3.455e+01	0.0000	508.0	7.63%	4861.9	5.31%
78	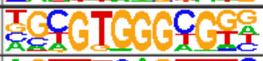	Egr2(Zf)/Thymocytes-Egr2-ChIP-Seq(GSE34254)/Homer	1e-14	-3.396e+01	0.0000	235.0	3.53%	1855.3	2.03%
79	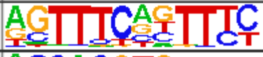	IRF3(IRF)/BMDM-Irf3-ChIP-Seq(GSE67343)/Homer	1e-14	-3.231e+01	0.0000	486.0	7.30%	4673.7	5.10%
80	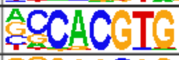	c-Myc(bHLH)/LNCAP-cMyc-ChIP-Seq(Unpublished)/Homer	1e-13	-3.101e+01	0.0000	596.0	8.95%	6005.4	6.55%
81	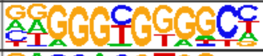	KLF1(Zf)/HUDEP2-KLF1-CutnRun(GSE136251)/Homer	1e-13	-3.044e+01	0.0000	1149.0	17.25%	12825.2	14.00%
82	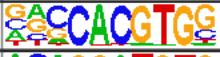	n-Myc(bHLH)/mES-nMyc-ChIP-Seq(GSE11431)/Homer	1e-13	-3.012e+01	0.0000	1080.0	16.22%	11973.6	13.07%
83	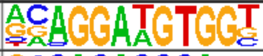	ETS:RUNX(ETS,Runt)/Jurkat-RUNX1-ChIP-Seq(GSE17954)/Homer	1e-13	-3.007e+01	0.0000	205.0	3.08%	1614.8	1.76%
84	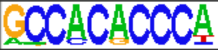	Klf4(Zf)/mES-Klf4-ChIP-Seq(GSE11431)/Homer	1e-12	-2.913e+01	0.0000	607.0	9.11%	6203.5	6.77%
85	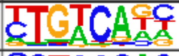	Tgif1(Homeobox)/mES-Tgif1-ChIP-Seq(GSE55404)/Homer	1e-12	-2.892e+01	0.0000	4438.0	66.64%	57154.7	62.38%
86	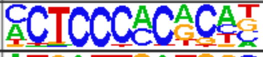	WT1(Zf)/Kidney-WT1-ChIP-Seq(GSE90016)/Homer	1e-12	-2.831e+01	0.0000	889.0	13.35%	9676.4	10.56%
87	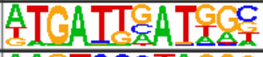	HOXA3(Homeobox)/mEmbryo-Hoxa3-ChIP-Seq(E-MTAB-8607)/Homer	1e-12	-2.822e+01	0.0000	276.0	4.14%	2410.0	2.63%
88	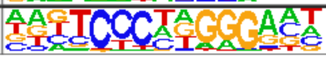	EBF2(EBF)/BrownAdipose-EBF2-ChIP-Seq(GSE97114)/Homer	1e-12	-2.814e+01	0.0000	1596.0	23.96%	18671.9	20.38%

89	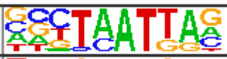	Emx2(Homeobox)/Cortex-Emx2-ChIP-Seq(GSE183130)/Homer	1e-12	-2.783e+01	0.0000	1683.0	25.27%	19820.2	21.63%
90	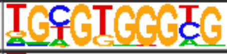	Egr1(Zf)/K562-Egr1-ChIP-Seq(GSE32465)/Homer	1e-12	-2.775e+01	0.0000	894.0	13.42%	9764.7	10.66%
91	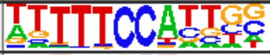	NFATC2(RHD)/Islets-NFATC2-ChIP-Seq(GSE158496)/Homer	1e-11	-2.661e+01	0.0000	2627.0	39.44%	32404.0	35.37%
92	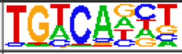	Tgif2(Homeobox)/mES-Tgif2-ChIP-Seq(GSE55404)/Homer	1e-11	-2.656e+01	0.0000	4577.0	68.72%	59298.6	64.72%
93	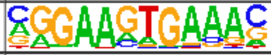	PU.1-IRF(ETS:IRF)/Bcell-PU.1-ChIP-Seq(GSE21512)/Homer	1e-11	-2.567e+01	0.0000	1958.0	29.40%	23566.7	25.72%
94	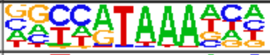	CDX4(Homeobox)/ZebrafishEmbryos-Cdx4.Myc-ChIP-Seq(GSE48254)/Homer	1e-11	-2.566e+01	0.0000	1120.0	16.82%	12708.7	13.87%
95	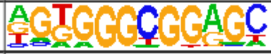	Sp5(Zf)/mES-Sp5.Flag-ChIP-Seq(GSE72989)/Homer	1e-11	-2.563e+01	0.0000	1185.0	17.79%	13536.7	14.77%
96	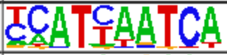	Pdx1(Homeobox)/Islet-Pdx1-ChIP-Seq(SRA008281)/Homer	1e-10	-2.533e+01	0.0000	1455.0	21.85%	17020.7	18.58%
97	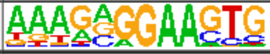	SpiB(ETS)/OCILY3-SPIB-ChIP-Seq(GSE56857)/Homer	1e-10	-2.481e+01	0.0000	416.0	6.25%	4092.5	4.47%
98	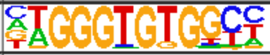	EKLF(Zf)/Erythrocyte-Klf1-ChIP-Seq(GSE20478)/Homer	1e-10	-2.473e+01	0.0000	406.0	6.10%	3978.9	4.34%
99	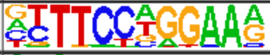	STAT4(Stat)/CD4-Stat4-ChIP-Seq(GSE22104)/Homer	1e-10	-2.438e+01	0.0000	1421.0	21.34%	16640.9	18.16%
100	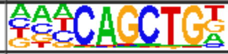	Ap4(bHLH)/AML-Tfap4-ChIP-Seq(GSE45738)/Homer	1e-10	-2.424e+01	0.0000	1942.0	29.16%	23457.3	25.60%
101	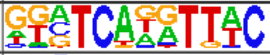	Six1(Homeobox)/Myoblast-Six1-ChIP-Chip(GSE20150)/Homer	1e-10	-2.422e+01	0.0000	437.0	6.56%	4358.0	4.76%
102	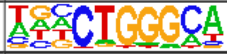	ZNF416(Zf)/HEK293-ZNF416.GFP-ChIP-Seq(GSE58341)/Homer	1e-9	-2.301e+01	0.0000	2092.0	31.41%	25533.8	27.87%
103	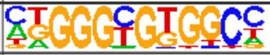	KLF6(Zf)/PDAC-KLF6-ChIP-Seq(GSE64557)/Homer	1e-9	-2.269e+01	0.0000	1323.0	19.86%	15482.9	16.90%
104	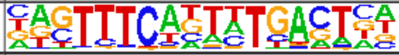	bZIP:IRF(bZIP,IRF)/Th17-BatF-ChIP-Seq(GSE39756)/Homer	1e-9	-2.265e+01	0.0000	680.0	10.21%	7353.4	8.03%
105	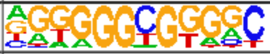	Klf15(Zf)/Liver-Klf15-ChIP-Seq(GSE166083)/Homer	1e-9	-2.251e+01	0.0000	1337.0	20.08%	15674.4	17.11%
106	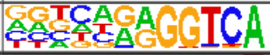	EAR2(NR)/K562-NR2F6-ChIP-Seq(Encode)/Homer	1e-9	-2.196e+01	0.0000	2151.0	32.30%	26399.2	28.81%
107	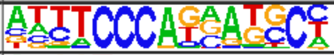	ZNF143|STAF(Zf)/CUTLL-ZNF143-ChIP-Seq(GSE29600)/Homer	1e-9	-2.193e+01	0.0000	655.0	9.83%	7078.1	7.73%
108	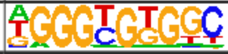	KLF5(Zf)/LoVo-KLF5-ChIP-Seq(GSE49402)/Homer	1e-9	-2.166e+01	0.0000	1617.0	24.28%	19372.5	21.14%
109	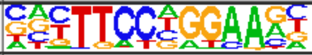	Stat3+il21(Stat)/CD4-Stat3-ChIP-Seq(GSE19198)/Homer	1e-9	-2.161e+01	0.0000	1076.0	16.16%	12379.3	13.51%
110	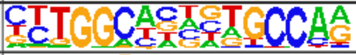	NF1(CTF)/LNCAP-NF1-ChIP-Seq(Unpublished)/Homer	1e-9	-2.110e+01	0.0000	639.0	9.59%	6919.8	7.55%
111	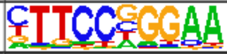	Stat3(Stat)/mES-Stat3-ChIP-Seq(GSE11431)/Homer	1e-9	-2.086e+01	0.0000	773.0	11.61%	8593.8	9.38%
112	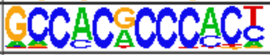	Klf9(Zf)/GBM-Klf9-ChIP-Seq(GSE62211)/Homer	1e-8	-2.058e+01	0.0000	517.0	7.76%	5451.5	5.95%
113	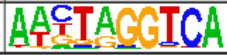	RORgt(NR)/EL4-RORgt.Flag-ChIP-Seq(GSE56019)/Homer	1e-8	-2.033e+01	0.0000	249.0	3.74%	2304.2	2.51%
114	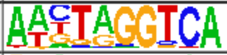	RORgt(NR)/EL4-RORgt.Flag-ChIP-Seq(GSE56019)/Homer	1e-8	-2.033e+01	0.0000	249.0	3.74%	2304.2	2.51%
115	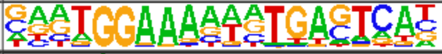	NFAT:AP1(RHD,bZIP)/Jurkat-NFATC1-ChIP-Seq(Jolma_et_al.)/Homer	1e-8	-1.983e+01	0.0000	318.0	4.77%	3110.6	3.40%
116	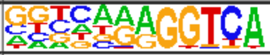	COUP-TFII(NR)/K562-NR2F1-ChIP-Seq(Encode)/Homer	1e-8	-1.978e+01	0.0000	2265.0	34.01%	28099.1	30.67%
117	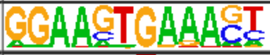	PU.1:IRF8(ETS:IRF)/pDC-Irf8-ChIP-Seq(GSE66899)/Homer	1e-8	-1.924e+01	0.0000	330.0	4.95%	3269.2	3.57%
118	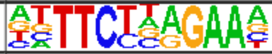	STAT5(Stat)/mCD4+-Stat5-ChIP-Seq(GSE12346)/Homer	1e-8	-1.854e+01	0.0000	566.0	8.50%	6139.6	6.70%
119	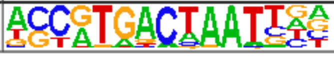	PAX3:FKHR-fusion(Paired,Homeobox)/Rh4-PAX3:FKHR-ChIP-Seq(GSE19063)/Homer	1e-8	-1.852e+01	0.0000	320.0	4.80%	3176.5	3.47%

120	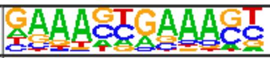	IRF2(IRF)/Erythroblas-IRF2-ChIP-Seq(GSE36985)/Homer	1e-7	-1.841e+01	0.0000	167.0	2.51%	1443.0	1.57%
121	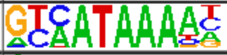	Cdx2(Homeobox)/mES-Cdx2-ChIP-Seq(GSE14586)/Homer	1e-7	-1.798e+01	0.0000	845.0	12.69%	9657.4	10.54%
122	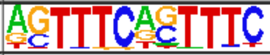	ISRE(IRF)/ThioMac-LPS-Expression(GSE23622)/Homer	1e-7	-1.776e+01	0.0000	104.0	1.56%	791.3	0.86%
123	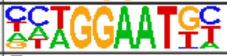	TEAD(TEA)/Fibroblast-PU.1-ChIP-Seq(Unpublished)/Homer	1e-7	-1.750e+01	0.0000	993.0	14.91%	11573.6	12.63%
124	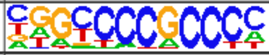	Sp2(Zf)/HEK293-Sp2.eGFP-ChIP-Seq(Encode)/Homer	1e-7	-1.744e+01	0.0000	1797.0	26.98%	22069.4	24.09%
125	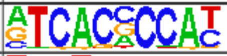	Srebp1a(bHLH)/HepG2-Srebp1a-ChIP-Seq(GSE31477)/Homer	1e-7	-1.742e+01	0.0000	407.0	6.11%	4251.9	4.64%
126	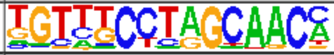	Rfx6(HTH)/Min6b1-Rfx6.HA-ChIP-Seq(GSE62844)/Homer	1e-7	-1.739e+01	0.0000	1618.0	24.29%	19710.2	21.51%
127	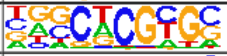	Zfp541(Zf)/Sperm-Zfp541-ChIP-Seq(GSE163916)/Homer	1e-7	-1.736e+01	0.0000	629.0	9.44%	6977.1	7.62%
128	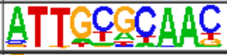	CEBP(bZIP)/ThioMac-CEBPb-ChIP-Seq(GSE21512)/Homer	1e-7	-1.735e+01	0.0000	813.0	12.21%	9289.0	10.14%
129	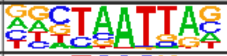	En1(Homeobox)/SUM149-EN1-ChIP-Seq(GSE120957)/Homer	1e-7	-1.715e+01	0.0000	2281.0	34.25%	28547.1	31.16%
130	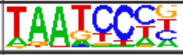	Pitx1(Homeobox)/Chicken-Pitx1-ChIP-Seq(GSE38910)/Homer	1e-7	-1.696e+01	0.0000	4565.0	68.54%	59954.2	65.44%
131	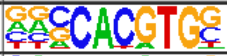	c-Myc(bHLH)/mES-cMyc-ChIP-Seq(GSE11431)/Homer	1e-7	-1.672e+01	0.0000	780.0	11.71%	8908.7	9.72%
132	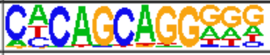	Unknown-ESC-element(?)/mES-Nanog-ChIP-Seq(GSE11724)/Homer	1e-7	-1.665e+01	0.0000	1017.0	15.27%	11937.2	13.03%
133	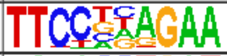	STAT6(Stat)/Macrophage-Stat6-ChIP-Seq(GSE38377)/Homer	1e-7	-1.645e+01	0.0000	870.0	13.06%	10068.6	10.99%
134	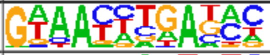	Six2(Homeobox)/NephronProgenitor-Six2-ChIP-Seq(GSE39837)/Homer	1e-6	-1.600e+01	0.0000	1395.0	20.95%	16893.0	18.44%
135	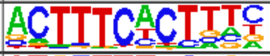	PRDM1(Zf)/Hela-PRDM1-ChIP-Seq(GSE31477)/Homer	1e-6	-1.575e+01	0.0000	787.0	11.82%	9056.0	9.88%
136	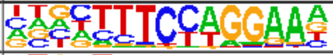	Bcl6(Zf)/Liver-Bcl6-ChIP-Seq(GSE31578)/Homer	1e-6	-1.575e+01	0.0000	1988.0	29.85%	24758.1	27.02%
137	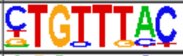	Foxo1(Forkhead)/RAW-Foxo1-ChIP-Seq(Fan_et_al.)/Homer	1e-6	-1.568e+01	0.0000	2620.0	39.34%	33258.8	36.30%
138	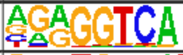	COUP-TFII(NR)/Artia-Nr2f2-ChIP-Seq(GSE46497)/Homer	1e-6	-1.555e+01	0.0000	2547.0	38.24%	32284.2	35.24%
139	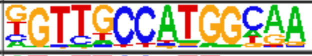	Rfx1(HTH)/NPC-H3K4me1-ChIP-Seq(GSE16256)/Homer	1e-6	-1.538e+01	0.0000	358.0	5.38%	3743.5	4.09%
140	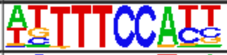	NFAT(RHD)/Jurkat-NFATC1-ChIP-Seq(Jolma_et_al.)/Homer	1e-6	-1.523e+01	0.0000	1234.0	18.53%	14850.3	16.21%
141	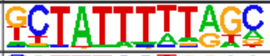	Mef2d(MADS)/Retina-Mef2d-ChIP-Seq(GSE61391)/Homer	1e-6	-1.510e+01	0.0000	232.0	3.48%	2262.3	2.47%
142	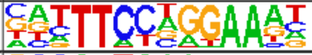	STAT1(Stat)/HelaS3-STAT1-ChIP-Seq(GSE12782)/Homer	1e-6	-1.428e+01	0.0000	452.0	6.79%	4938.4	5.39%
143	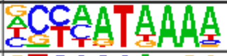	Hoxd13(Homeobox)/ChickenMSG-Hoxd13.Flag-ChIP-Seq(GSE86088)/Homer	1e-5	-1.382e+01	0.0000	1910.0	28.68%	23902.8	26.09%
144	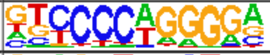	EBF1(EBF)/Near-E2A-ChIP-Seq(GSE21512)/Homer	1e-5	-1.356e+01	0.0000	1602.0	24.05%	19831.5	21.65%
145	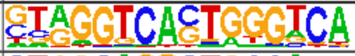	Reverb(NR),DR2/RAW-Reverba.biotin-ChIP-Seq(GSE45914)/Homer	1e-5	-1.347e+01	0.0000	275.0	4.13%	2824.4	3.08%
146	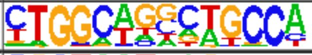	Tlx?(NR)/NPC-H3K4me1-ChIP-Seq(GSE16256)/Homer	1e-5	-1.341e+01	0.0000	712.0	10.69%	8249.8	9.00%
147	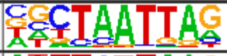	LHX9(Homeobox)/Hct116-LHX9.V5-ChIP-Seq(GSE116822)/Homer	1e-5	-1.328e+01	0.0000	1794.0	26.94%	22408.9	24.46%
148	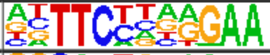	STAT6(Stat)/CD4-Stat6-ChIP-Seq(GSE22104)/Homer	1e-5	-1.299e+01	0.0000	814.0	12.22%	9581.8	10.46%
149	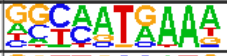	Hoxd10(Homeobox)/ChickenMSG-Hoxd10.Flag-ChIP-Seq(GSE86088)/Homer	1e-5	-1.290e+01	0.0000	1521.0	22.84%	18820.0	20.54%
150	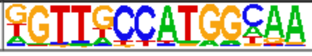	X-box(HTH)/NPC-H3K4me1-ChIP-Seq(GSE16256)/Homer	1e-5	-1.263e+01	0.0000	208.0	3.12%	2063.2	2.25%

151	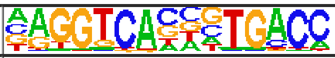	ERE(NR),IR3/MCF7-ERa-ChIP-Seq(Unpublished)/Homer	1e-5	-1.236e+01	0.0000	492.0	7.39%	5534.3	6.04%
152	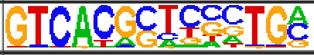	PAX5(Paired,Homeobox),condensed/GM12878-PAX5-ChIP-Seq(GSE32465)/Homer	1e-5	-1.215e+01	0.0000	205.0	3.08%	2044.1	2.23%
153	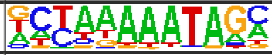	Mef2c(MADS)/GM12878-Mef2c-ChIP-Seq(GSE32465)/Homer	1e-5	-1.211e+01	0.0000	470.0	7.06%	5273.6	5.76%
154	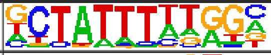	Mef2b(MADS)/HEK293-Mef2b.V5-ChIP-Seq(GSE67450)/Homer	1e-5	-1.188e+01	0.0000	938.0	14.08%	11264.9	12.30%
155	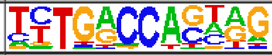	Bcl11a(Zf)/HSPC-BCL11A-ChIP-Seq(GSE104676)/Homer	1e-5	-1.176e+01	0.0000	1205.0	18.09%	14760.0	16.11%
156	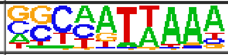	Unknown(Homeobox)/Limb-p300-ChIP-Seq/Homer	1e-5	-1.169e+01	0.0000	739.0	11.10%	8710.8	9.51%
157	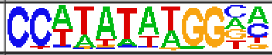	CArG(MADS)/PUER-Srf-ChIP-Seq(Sullivan_et_al.)/Homer	1e-5	-1.159e+01	0.0000	452.0	6.79%	5077.2	5.54%
158	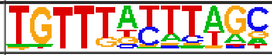	FoxD3(forkhead)/ZebrafishEmbryo-Foxd3.biotin-ChIP-seq(GSE106676)/Homer	1e-4	-1.151e+01	0.0000	1098.0	16.49%	13380.5	14.60%
159	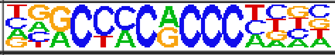	KLF3(Zf)/MEF-Klf3-ChIP-Seq(GSE44748)/Homer	1e-4	-1.136e+01	0.0000	604.0	9.07%	7008.4	7.65%
160	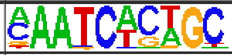	Gfi1b(Zf)/HPC7-Gfi1b-ChIP-Seq(GSE22178)/Homer	1e-4	-1.127e+01	0.0000	908.0	13.63%	10924.6	11.92%
161	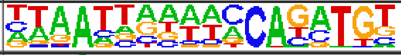	Pitx1:Ebox(Homeobox,bHLH)/Hindlimb-Pitx1-ChIP-Seq(GSE41591)/Homer	1e-4	-1.124e+01	0.0000	269.0	4.04%	2841.7	3.10%
162	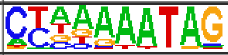	Mef2a(MADS)/HL1-Mef2a.biotin-ChIP-Seq(GSE21529)/Homer	1e-4	-1.121e+01	0.0000	495.0	7.43%	5638.4	6.15%
163	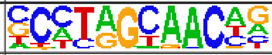	Rfx5(HTH)/GM12878-Rfx5-ChIP-Seq(GSE31477)/Homer	1e-4	-1.111e+01	0.0000	502.0	7.54%	5732.4	6.26%
164	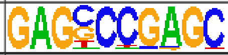	ZNF519(Zf)/HEK293-ZNF519.GFP-ChIP-Seq(GSE58341)/Homer	1e-4	-1.097e+01	0.0000	264.0	3.96%	2792.9	3.05%
165	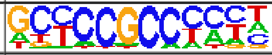	KLF17(Zf)/2cell-Klf17-CutnTag(GSE211845)/Homer	1e-4	-1.078e+01	0.0001	422.0	6.34%	4747.6	5.18%
166	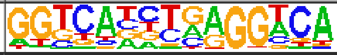	THRa(NR)/C17.2-THRa-ChIP-Seq(GSE38347)/Homer	1e-4	-1.058e+01	0.0001	698.0	10.48%	8264.9	9.02%
167	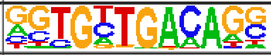	Tbx20(T-box)/Heart-Tbx20-ChIP-Seq(GSE29636)/Homer	1e-4	-1.031e+01	0.0001	432.0	6.49%	4899.3	5.35%
168	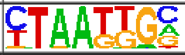	Isl1(Homeobox)/Neuron-Isl1-ChIP-Seq(GSE31456)/Homer	1e-4	-9.933e+00	0.0001	2475.0	37.16%	31946.4	34.87%
169	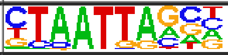	Gsx2(Homeobox)/LGE-Gsx2.Flag-ChIP-Seq(GSE162589)/Homer	1e-4	-9.904e+00	0.0001	1694.0	25.44%	21431.4	23.39%
170	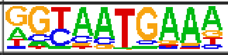	Hoxa10(Homeobox)/ChickenMSG-Hoxa10.Flag-ChIP-Seq(GSE86088)/Homer	1e-4	-9.800e+00	0.0002	803.0	12.06%	9681.3	10.57%
171	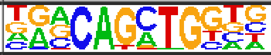	Atoh7(bHLH)/Retina-Atoh7-CutnRun(GSE156756)/Homer	1e-4	-9.769e+00	0.0002	1278.0	19.19%	15913.2	17.37%
172	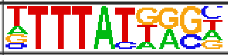	Hoxc13(Homeobox)/EB-Hoxc13.HA-ChIP-Seq(GSE142377)/Homer	1e-4	-9.730e+00	0.0002	1705.0	25.60%	21598.1	23.57%
173	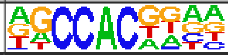	ZBTB9(Zf)/HEK293T-ZBTB9-ChIP-Seq(GSE251687)/Homer	1e-4	-9.618e+00	0.0002	2526.0	37.93%	32679.5	35.67%
174	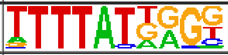	HOXB13(Homeobox)/ProstateTumor-HOXB13-ChIP-Seq(GSE56288)/Homer	1e-4	-9.547e+00	0.0002	1274.0	19.13%	15883.2	17.34%
175	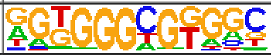	KLF14(Zf)/HEK293-KLF14.GFP-ChIP-Seq(GSE58341)/Homer	1e-4	-9.526e+00	0.0002	1975.0	29.65%	25244.2	27.55%
176	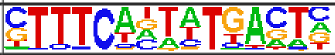	IRF:BATF(IRF:bZIP)/pDC-Irf8-ChIP-Seq(GSE66899)/Homer	1e-4	-9.504e+00	0.0002	191.0	2.87%	1974.2	2.15%
177	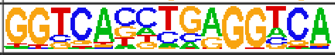	THRb(NR)/HepG2-THRb.Flag-ChIP-Seq(Encode)/Homer	1e-4	-9.493e+00	0.0002	901.0	13.53%	10983.1	11.99%
178	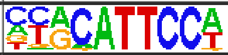	TEAD1(TEAD)/HepG2-TEAD1-ChIP-Seq(Encode)/Homer	1e-4	-9.276e+00	0.0002	1354.0	20.33%	16972.1	18.52%
179	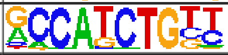	NeuroD1(bHLH)/Islet-NeuroD1-ChIP-Seq(GSE30298)/Homer	1e-3	-9.205e+00	0.0003	1344.0	20.18%	16847.4	18.39%
180	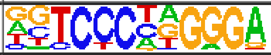	EBF(EBF)/proBcell-EBF-ChIP-Seq(GSE21978)/Homer	1e-3	-9.123e+00	0.0003	377.0	5.66%	4278.3	4.67%
181	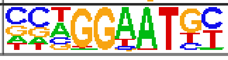	TEAD4(TEA)/Tropoblast-Tead4-ChIP-Seq(GSE37350)/Homer	1e-3	-8.840e+00	0.0004	1217.0	18.27%	15203.3	16.59%

182	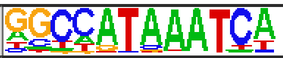	Hoxc9(Homeobox)/Ainv15-Hoxc9-ChIP-Seq(GSE21812)/Homer	1e-3	-8.797e+00	0.0004	635.0	9.53%	7588.0	8.28%
183	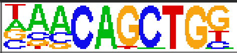	Tcf21(bHLH)/ArterySmoothMuscle-Tcf21-ChIP-Seq(GSE61369)/Homer	1e-3	-8.793e+00	0.0004	1476.0	22.16%	18649.4	20.36%
184	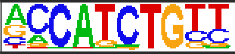	NeuroG2(bHLH)/Fibroblast-NeuroG2-ChIP-Seq(GSE75910)/Homer	1e-3	-8.627e+00	0.0005	2410.0	36.19%	31239.8	34.10%
185	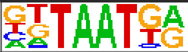	Nkx6.1(Homeobox)/Islet-Nkx6.1-ChIP-Seq(GSE40975)/Homer	1e-3	-8.254e+00	0.0007	2992.0	44.92%	39222.3	42.81%
186	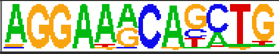	ETS:E-box(ETS,bHLH)/HPC7-Scl-ChIP-Seq(GSE22178)/Homer	1e-3	-8.184e+00	0.0007	185.0	2.78%	1955.6	2.13%
187	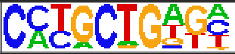	Zic(Zf)/Cerebellum-ZIC1.2-ChIP-Seq(GSE60731)/Homer	1e-3	-8.164e+00	0.0007	1304.0	19.58%	16432.6	17.94%
188	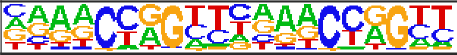	Tcfcp2l1(CP2)/mES-Tcfcp2l1-ChIP-Seq(GSE11431)/Homer	1e-3	-7.994e+00	0.0008	250.0	3.75%	2758.5	3.01%
189	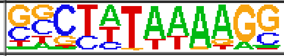	TATA-Box(TBP)/Promoter/Homer	1e-3	-7.991e+00	0.0008	1610.0	24.17%	20537.6	22.42%
190	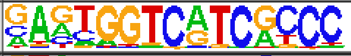	ZNF669(Zf)/HEK293-ZNF669.GFP-ChIP-Seq(GSE58341)/Homer	1e-3	-7.981e+00	0.0008	119.0	1.79%	1178.3	1.29%
191	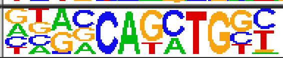	Atoh1(bHLH)/Cerebellum-Atoh1-ChIP-Seq(GSE22111)/Homer	1e-3	-7.911e+00	0.0009	1733.0	26.02%	22197.6	24.23%
192	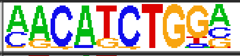	ZBTB18(Zf)/HEK293-ZBTB18.GFP-ChIP-Seq(GSE58341)/Homer	1e-3	-7.862e+00	0.0009	866.0	13.00%	10677.4	11.65%
193	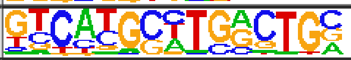	Pax8(Paired,Homeobox)/Thyroid-Pax8-ChIP-Seq(GSE26938)/Homer	1e-3	-7.830e+00	0.0010	507.0	7.61%	6015.4	6.57%
194	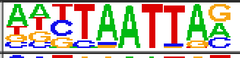	Lhx3(Homeobox)/Neuron-Lhx3-ChIP-Seq(GSE31456)/Homer	1e-3	-7.707e+00	0.0011	1919.0	28.81%	24727.7	26.99%
195	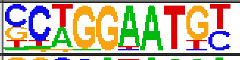	TEAD2(TEA)/Py2T-Tead2-ChIP-Seq(GSE55709)/Homer	1e-3	-7.702e+00	0.0011	764.0	11.47%	9358.0	10.21%
196	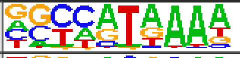	Hoxd11(Homeobox)/ChickenMSG-Hoxd11.Flag-ChIP-Seq(GSE86088)/Homer	1e-3	-7.695e+00	0.0011	2825.0	42.42%	37024.1	40.41%
197	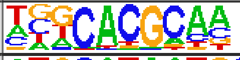	Arnt:Ahr(bHLH)/MCF7-Arnt-ChIP-Seq(Lo_et_al.)/Homer	1e-3	-7.690e+00	0.0011	725.0	10.89%	8849.9	9.66%
198	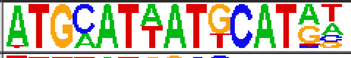	OCT:OCT-short(POU,Homeobox)/NPC-OCT6-ChIP-Seq(GSE43916)/Homer	1e-3	-7.682e+00	0.0011	860.0	12.91%	10616.6	11.59%
199	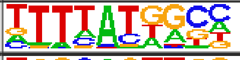	Hoxa11(Homeobox)/ChickenMSG-Hoxa11.Flag-ChIP-Seq(GSE86088)/Homer	1e-3	-7.480e+00	0.0013	2696.0	40.48%	35297.3	38.53%
200	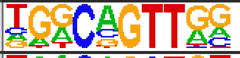	AMYB(HTH)/Testes-AMYB-ChIP-Seq(GSE44588)/Homer	1e-3	-7.431e+00	0.0014	2117.0	31.79%	27440.1	29.95%
201	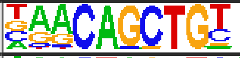	Myf5(bHLH)/GM-Myf5-ChIP-Seq(GSE24852)/Homer	1e-3	-7.386e+00	0.0015	1111.0	16.68%	13959.6	15.24%
202	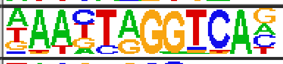	RORgt(NR)/Liver-Rorc-ChIP-Seq(GSE101115)/Homer	1e-3	-7.196e+00	0.0018	149.0	2.24%	1562.6	1.71%
203	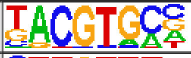	HIF-1a(bHLH)/MCF7-HIF1a-ChIP-Seq(GSE28352)/Homer	1e-3	-7.162e+00	0.0018	289.0	4.34%	3289.0	3.59%
204	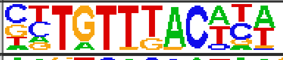	Foxa2(Forkhead)/Liver-Foxa2-ChIP-Seq(GSE25694)/Homer	1e-3	-7.075e+00	0.0020	1119.0	16.80%	14101.6	15.39%
205	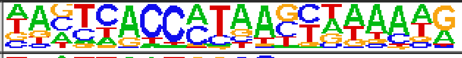	PSE(SNAPc)/K562-mStart-Seq/Homer	1e-2	-6.795e+00	0.0026	695.0	10.44%	8541.6	9.32%
206	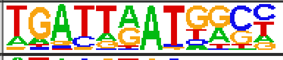	Hoxb4(Homeobox)/ES-Hoxb4-ChIP-Seq(GSE34014)/Homer	1e-2	-6.782e+00	0.0026	312.0	4.68%	3602.2	3.93%
207	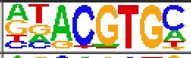	HIF-1b(HLH)/T47D-HIF1b-ChIP-Seq(GSE59937)/Homer	1e-2	-6.772e+00	0.0026	1248.0	18.74%	15854.6	17.30%
208	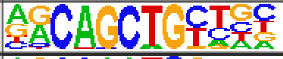	MyoD(bHLH)/Myotube-MyoD-ChIP-Seq(GSE21614)/Homer	1e-2	-6.663e+00	0.0029	1142.0	17.15%	14456.6	15.78%
209	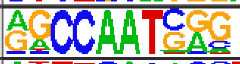	NFY(CCAAT)/Promoter/Homer	1e-2	-6.629e+00	0.0030	1035.0	15.54%	13039.8	14.23%
210	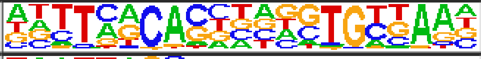	Brachyury(T-box)/Mesoendoderm-Brachyury-ChIP-exo(GSE54963)/Homer	1e-2	-6.555e+00	0.0032	437.0	6.56%	5213.1	5.69%
211	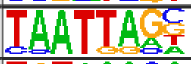	Lhx2(Homeobox)/HFSC-Lhx2-ChIP-Seq(GSE48068)/Homer	1e-2	-6.496e+00	0.0034	1256.0	18.86%	15996.7	17.46%
212	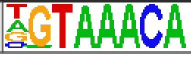	Foxo3(Forkhead)/U2OS-Foxo3-ChIP-Seq(E-MTAB-2701)/Homer	1e-2	-6.352e+00	0.0039	982.0	14.74%	12369.3	13.50%

213	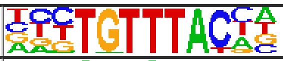	FOXP1(Forkhead)/H9-FOXP1-ChIP-Seq(GSE31006)/Homer	1e-2	-6.206e+00	0.0045	572.0	8.59%	6991.2	7.63%
214	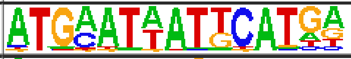	OCT:OCT(POU,Homeobox,IR1)/NPC-Brn2-ChIP-Seq(GSE35496)/Homer	1e-2	-6.145e+00	0.0047	25.0	0.38%	180.2	0.20%
215	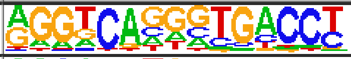	ERb(NR),IR3/Ovary-ERb-ChIP-Seq(GSE203391)/Homer	1e-2	-6.001e+00	0.0054	512.0	7.69%	6229.7	6.80%
216	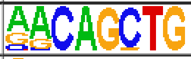	MyoG(bHLH)/C2C12-MyoG-ChIP-Seq(GSE36024)/Homer	1e-2	-5.926e+00	0.0058	1603.0	24.07%	20728.3	22.62%
217	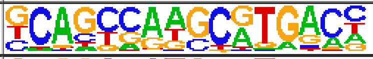	PAX5(Paired,Homeobox)/GM12878-PAX5-ChIP-Seq(GSE32465)/Homer	1e-2	-5.881e+00	0.0061	533.0	8.00%	6513.3	7.11%
218	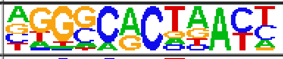	ZNF264(Zf)/HEK293-ZNF264.GFP-ChIP-Seq(GSE58341)/Homer	1e-2	-5.826e+00	0.0064	909.0	13.65%	11464.9	12.51%
219	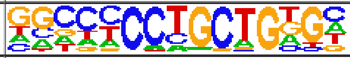	Zic3(Zf)/mES-Zic3-ChIP-Seq(GSE37889)/Homer	1e-2	-5.759e+00	0.0068	825.0	12.39%	10360.3	11.31%
220	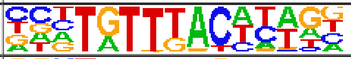	Foxa3(Forkhead)/Liver-Foxa3-ChIP-Seq(GSE77670)/Homer	1e-2	-5.662e+00	0.0075	432.0	6.49%	5221.2	5.70%
221	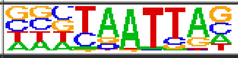	DLX2(Homeobox)/BasalGanglia-Dlx2-ChIP-seq(GSE124936)/Homer	1e-2	-5.618e+00	0.0078	1744.0	26.19%	22677.3	24.75%
222	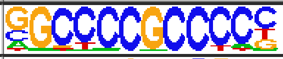	Sp1(Zf)/Promoter/Homer	1e-2	-5.610e+00	0.0078	151.0	2.27%	1656.8	1.81%
223	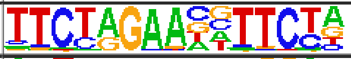	HRE(HSF)/Striatum-HSF1-ChIP-Seq(GSE38000)/Homer	1e-2	-5.597e+00	0.0078	378.0	5.68%	4530.0	4.94%
224	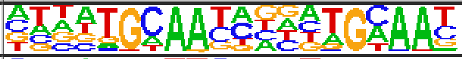	CEBP:CEBP(bZIP)/MEF-Chop-ChIP-Seq(GSE35681)/Homer	1e-2	-5.571e+00	0.0080	183.0	2.75%	2054.2	2.24%
225	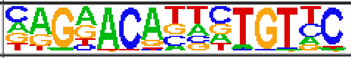	GRE(NR),IR3/RAW264.7-GRE-ChIP-Seq(Unpublished)/Homer	1e-2	-5.362e+00	0.0098	390.0	5.86%	4703.2	5.13%
226	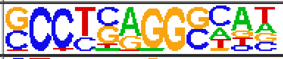	AP-2gamma(AP2)/MCF7-TFAP2C-ChIP-Seq(GSE21234)/Homer	1e-2	-5.249e+00	0.0110	1328.0	19.94%	17134.8	18.70%
227	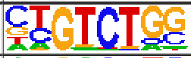	Smad2(MAD)/ES-SMAD2-ChIP-Seq(GSE29422)/Homer	1e-2	-5.212e+00	0.0113	2343.0	35.18%	30870.1	33.69%
228	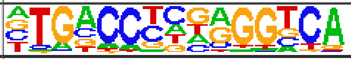	FXR(NR),ER2/Liver-FXR-ChIP-Seq(GSE133700)/Homer	1e-2	-5.180e+00	0.0117	741.0	11.13%	9317.5	10.17%
229	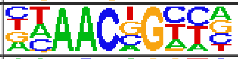	BMYB(HTH)/Hela-BMYB-ChIP-Seq(GSE27030)/Homer	1e-2	-5.128e+00	0.0122	1996.0	29.97%	26170.2	28.56%
230	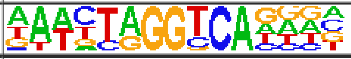	RORa(NR)/Liver-Rora-ChIP-Seq(GSE101115)/Homer	1e-2	-4.778e+00	0.0173	216.0	3.24%	2516.7	2.75%
231	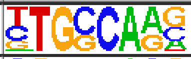	NF1-halfsite(CTF)/LNCaP-NF1-ChIP-Seq(Unpublished)/Homer	1e-2	-4.777e+00	0.0173	2373.0	35.63%	31361.7	34.23%
232	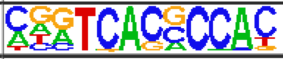	Srebp2(bHLH)/HepG2-Srebp2-ChIP-Seq(GSE31477)/Homer	1e-2	-4.761e+00	0.0174	223.0	3.35%	2606.2	2.84%
233	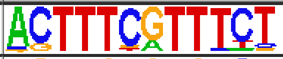	T1ISRE(IRF)/ThioMac-Ifnb-Expression/Homer	1e-2	-4.706e+00	0.0183	20.0	0.30%	151.8	0.17%
234	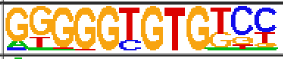	KLF10(Zf)/HEK293-KLF10.GFP-ChIP-Seq(GSE58341)/Homer	1e-2	-4.622e+00	0.0198	800.0	12.01%	10168.0	11.10%
235	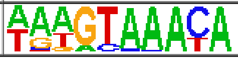	FOXA1(Forkhead)/MCF7-FOXA1-ChIP-Seq(GSE26831)/Homer	1e-2	-4.605e+00	0.0201	1157.0	17.37%	14938.8	16.31%

Appendix TABLE S2

Appendix Table S2: Exact P Values for the Figures

Exact P Values for the Figures

Figure 5D

	Signficance	Adjusted P Value
LLOMe vs. AY9944-5uM	****	<0.0001
LLOMe vs. AY9944-10uM	****	<0.0001
LLOMe vs. AY9944-20uM	****	<0.0001
LLOMe vs. Fatostatin-10uM	****	<0.0001
LLOMe vs. Fatostatin-20uM	****	<0.0001
LLOMe vs. Fatostatin-50uM	****	<0.0001
LLOMe vs. KU5593-10uM	****	<0.0001
LLOMe vs. KU5593-20uM	****	<0.0001
LLOMe vs. KU5593-50uM	****	<0.0001
LLOMe vs. GSK690693-2.5uM	ns	>0.9999
LLOMe vs. GSK690693-5uM	****	<0.0001
LLOMe vs. GSK690693-10uM	****	<0.0001
LLOMe vs. IWP-4-5uM	ns	>0.9999
LLOMe vs. IWP-4-20uM	ns	0.6133
LLOMe vs. IWP-4-50uM	****	<0.0001
LLOMe vs. EGCG 50μM	****	<0.0001
LLOMe vs. EGCG 100μM	****	<0.0001
LLOMe vs. EGCG 200μM	****	<0.0001
LLOMe vs. Amelaxanox-50uM	ns	>0.9999
LLOMe vs. Amelaxanox-100uM	****	<0.0001
LLOMe vs. Amelaxanox-200uM	****	<0.0001
LLOMe vs. H-151-5uM	ns	0.606
LLOMe vs. H-151-10uM	***	0.0001
LLOMe vs. H-151-20uM	****	<0.0001
LLOMe vs. Bay-11-7085-5uM	****	<0.0001
LLOMe vs. Bay-11-7085-10uM	****	<0.0001
LLOMe vs. Bay-11-7085-20uM	****	<0.0001
LLOMe vs. Ca074-Me-5uM	****	<0.0001
LLOMe vs. Ca074-Me-10uM	****	<0.0001
LLOMe vs. Ca074-Me-20uM	****	<0.0001
LLOMe vs. ML385 (5uM)	ns	0.9989
LLOMe vs. ML385 (10uM)	*	0.014

Figure 5E

	Significance	Adjusted P Value
LLOMe vs. AY9944-5uM	*	0.0204
LLOMe vs. AY9944-10uM	***	0.0006
LLOMe vs. AY9944-20uM	****	<0.0001
LLOMe vs. Fatostatin-10uM	*	0.0296
LLOMe vs. Fatostatin-20uM	****	<0.0001
LLOMe vs. Fatostatin-50uM	****	<0.0001
LLOMe vs. KU5593-10uM	****	<0.0001
LLOMe vs. KU5593-20uM	****	<0.0001
LLOMe vs. KU5593-50uM	****	<0.0001
LLOMe vs. GSK690693-2.5uM	ns	0.7946

LLOMe vs. GSK690693-5uM	ns	0.86
LLOMe vs. GSK690693-10uM	****	<0.0001
LLOMe vs. IWP-4-5uM	****	<0.0001
LLOMe vs. IWP-4-20uM	****	<0.0001
LLOMe vs. IWP-4-50uM	****	<0.0001
LLOMe vs. EGCG 50μM	ns	>0.9999
LLOMe vs. EGCG 100μM	***	0.0005
LLOMe vs. EGCG 200μM	****	<0.0001
LLOMe vs. Amelaxanox-50uM	****	<0.0001
LLOMe vs. Amelaxanox-100uM	****	<0.0001
LLOMe vs. Amelaxanox-200uM	****	<0.0001
LLOMe vs. H-151-5uM	****	<0.0001
LLOMe vs. H-151-10uM	****	<0.0001
LLOMe vs. H-151-20uM	****	<0.0001
LLOMe vs. Bay-11-7085-5uM	****	<0.0001
LLOMe vs. Bay-11-7085-10uM	****	<0.0001
LLOMe vs. Bay-11-7085-20uM	****	<0.0001
LLOMe vs. Ca074-Me-5uM	****	<0.0001
LLOMe vs. Ca074-Me-10uM	****	<0.0001
LLOMe vs. Ca074-Me-20uM	****	<0.0001

Figure 5H

**2mM**	Significance	Adjusted P Value
si-Control vs. si-Ror-alpha	***	0.0001
si-Control vs. si-Lipin1	****	<0.0001
si-Control vs. si-Per1	****	<0.0001
si-Control vs. si-Ep300	****	<0.0001
si-Control vs. si-Foxo3	**	0.0099
si-Control vs. si-Crebbp	****	<0.0001
si-Control vs. si-Nr4a3	****	<0.0001
si-Control vs. si-Atf3	****	<0.0001
si-Control vs. si-Map3k8	****	<0.0001
si-Control vs. si-Tef	****	<0.0001
si-Control vs. si-Fos	****	<0.0001
si-Control vs. si-NFkb	****	<0.0001

**4mM**	Significance	Adjusted P Value
si-Control vs. si-Ror-alpha	****	<0.0001
si-Control vs. si-Lipin1	****	<0.0001
si-Control vs. si-Per1	****	<0.0001
si-Control vs. si-Foxo3	****	<0.0001
si-Control vs. si-Atf3	****	<0.0001
si-Control vs. si-Map3k8	*	0.0112
si-Control vs. si-Tef	****	<0.0001
si-Control vs. si-Nfkb (rel)	****	<0.0001
si-Control vs. si-Fos	****	<0.0001
si-Control vs. si-Nr4a3	****	<0.0001
si-Control vs. si-Ep300	****	<0.0001

	Significance	Adjusted P Value
si-Control vs. si-Crebbp	****	<0.0001

Figure 7D

	Significance	Adjusted P Value
LLOMe vs Control	***	<0.001

Figure 7G

	Significance	Adjusted P Value
LLOMe vs Control	****	<0.0001

Figure 7J

	Significance	Adjusted P Value
Hematopoietic progenitors/stem cells		
LLOMe vs Control	****	<0.0001
Mitotic Cells	Significance	Adjusted P Value
LLOMe vs Control	***	0.0001

Figure 7M

	Significance	Adjusted P Value
LLOMe vs Control	***	0.0007

Figure EV7A

	Significance	Adjusted P Value
IL-17A		
Control vs. 3H LLOMe	**	0.0054
Control vs. 30H LLOMe	***	0.0001
3H LLOMe vs. 30H LLOMe	**	0.0061
CCL3	Significance	Adjusted P Value
Control vs. 3H LLOMe	ns	0.3497
Control vs. 30H LLOMe	*	0.0269
3H LLOMe vs. 30H LLOMe	ns	0.1763
CCL5	Significance	Adjusted P Value
Control vs. 3H LLOMe	ns	0.6046
Control vs. 30H LLOMe	**	0.0097
3H LLOMe vs. 30H LLOMe	*	0.0295
CXCL1	Significance	Adjusted P Value
Control vs. 3H LLOMe	*	0.0106
Control vs. 30H LLOMe	*	0.0103
3H LLOMe vs. 30H LLOMe	ns	0.9998
CXCL10	Significance	Adjusted P Value
Control vs. 3H LLOMe	*	0.0111
Control vs. 30H LLOMe	ns	0.0987
3H LLOMe vs. 30H LLOMe	ns	0.2314

IL-6	Significance	Adjusted P Value
Control vs. 3H LLOMe	**	0.0019
Control vs. 30H LLOMe	ns	0.0547
3H LLOMe vs. 30H LLOMe	*	0.0395

Figure EV7B

IL-4	Significance	Adjusted P Value
Control vs. 30mins	ns	>0.9999
30mins vs. 3hrs	****	<0.0001
3hrs vs. 30hrs	****	<0.0001

IL-10	Significance	Adjusted P Value
Control vs. 30mins	ns	0.7046
30mins vs. 3hrs	****	<0.0001
3hrs vs. 30hrs	****	<0.0001

IL-6	Significance	Adjusted P Value
Control vs. 30mins	ns	0.7649
30mins vs. 3hrs	****	<0.0001
3hrs vs. 30hrs	****	<0.0001

IL-1β	Significance	Adjusted P Value
Control vs. 30mins	ns	0.9972
30mins vs. 3hrs	ns	0.8969
3hrs vs. 30hrs	****	<0.0001

TNF-α	Significance	Adjusted P Value
Control vs. 30mins	ns	>0.9999
30mins vs. 3hrs	ns	>0.9999
3hrs vs. 30hrs	**	0.0067
