# Supplementary figures and images for: Programmed cell revival from imminent cell death enhances tissue repair and regeneration (part 1 of 4)
Source: EMBO J. 2025 Aug 21;44(19):5244–89. doi: 10.1038/s44318-025-00540-y (PMC12489119; doi:10.1038/s44318-025-00540-y)

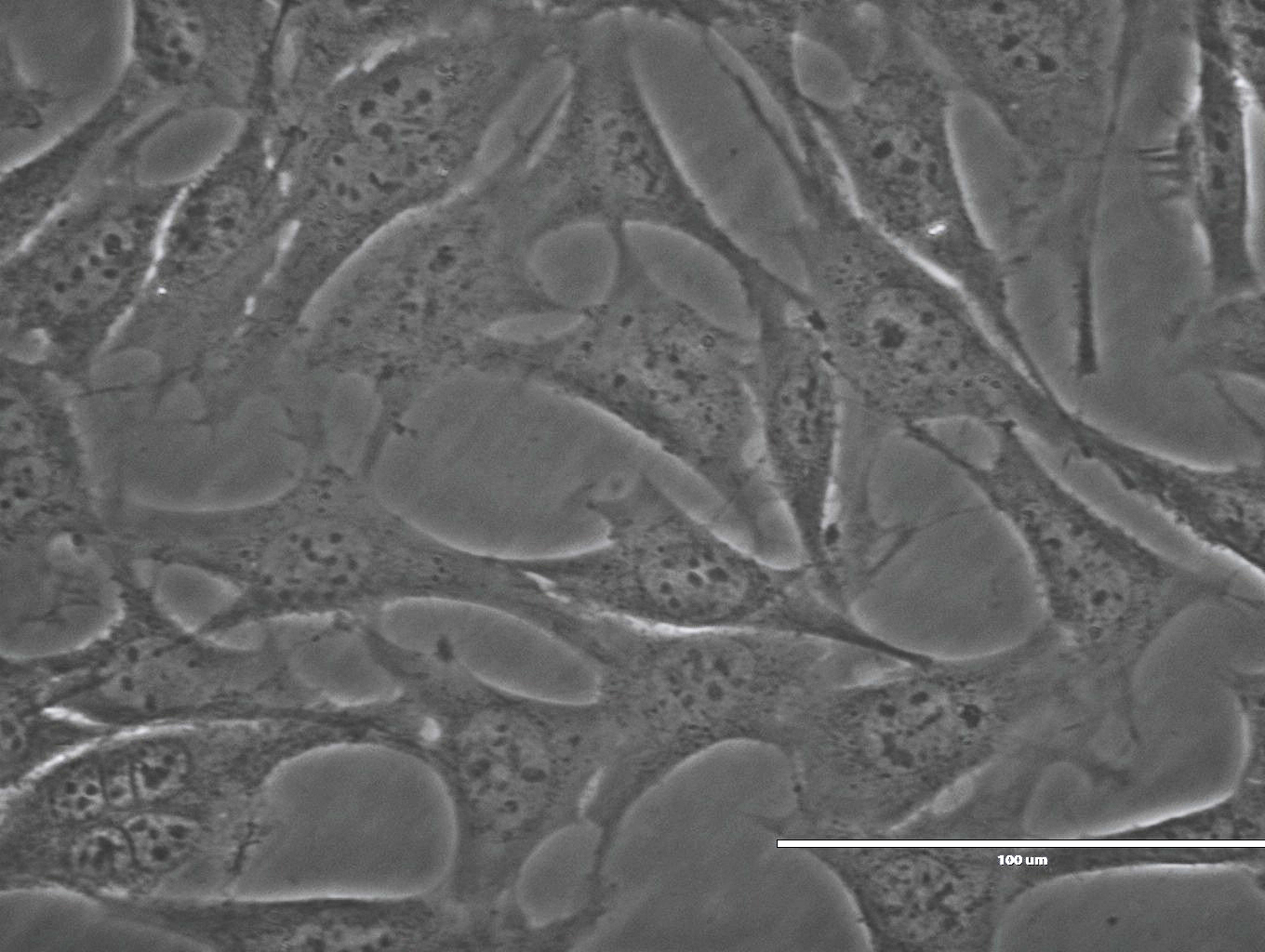

Supplement: Supplementary file 27 — Source data Fig. 1 [file 44318_2025_540_MOESM27_ESM.zip › SD Figure 1/1A/Microscopy MEF_0 h.JPG]

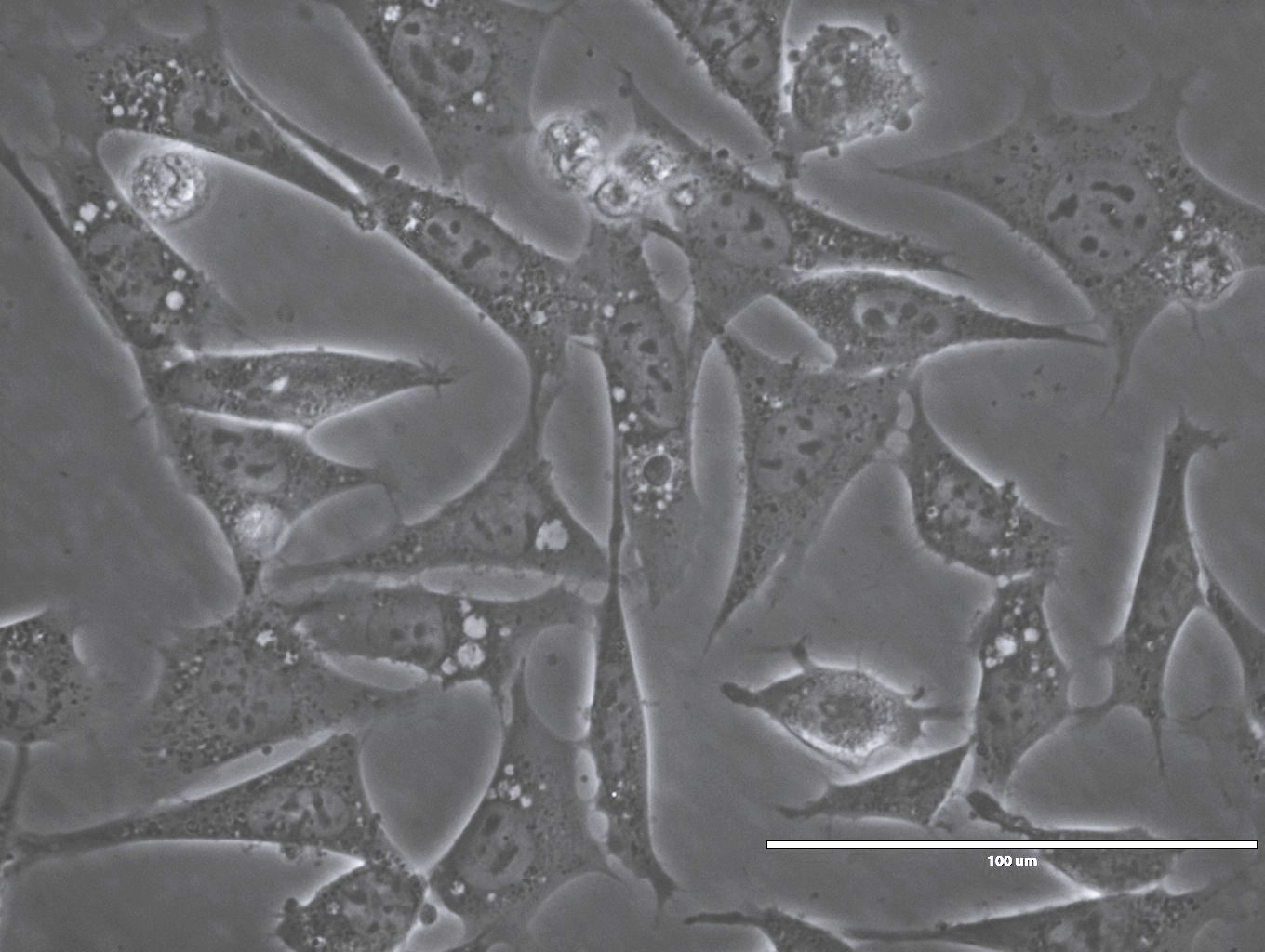

Supplement: Supplementary file 27 — Source data Fig. 1 [file 44318_2025_540_MOESM27_ESM.zip › SD Figure 1/1A/Microscopy MEF_12 h.jpg]

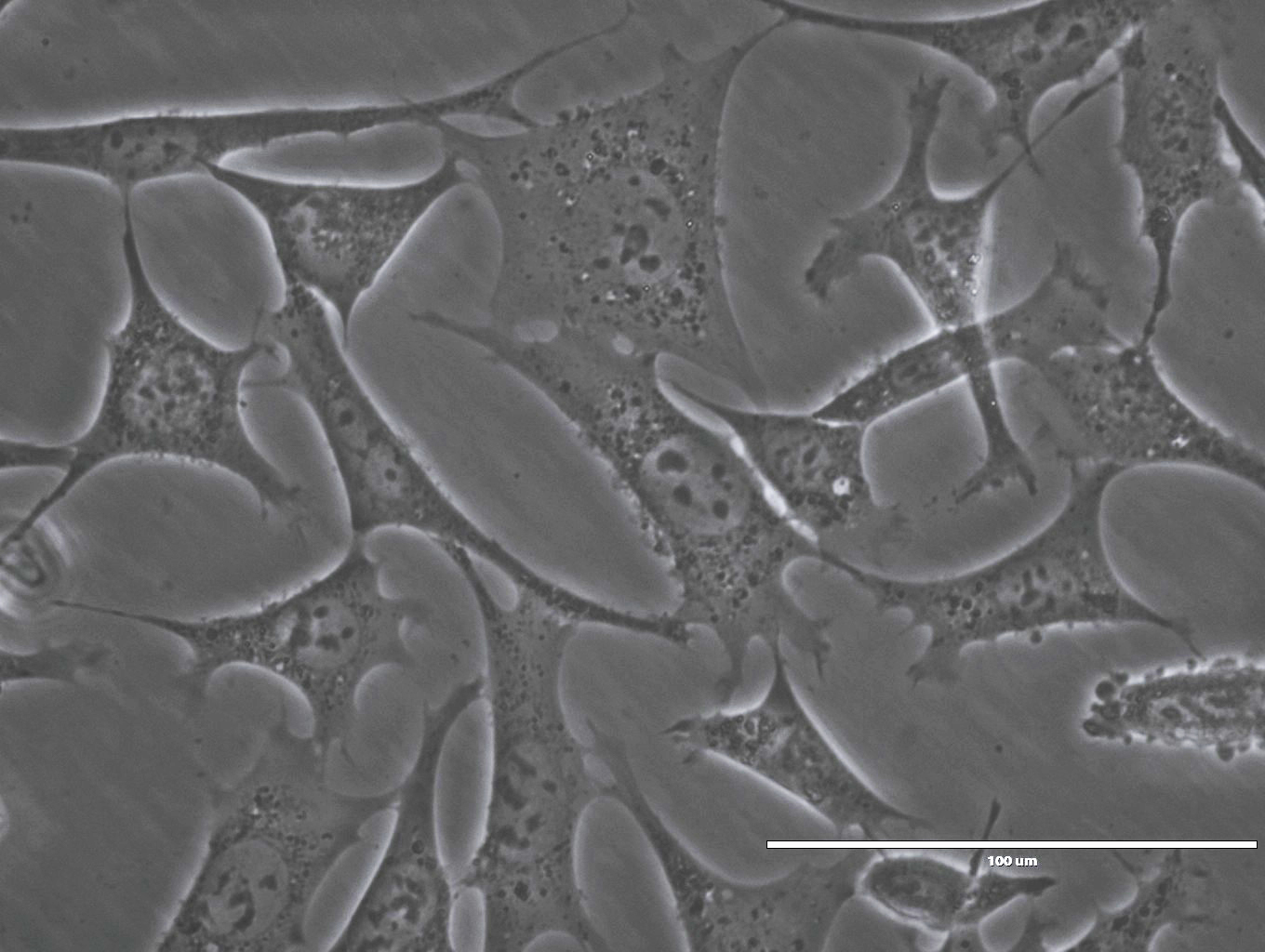

Supplement: Supplementary file 27 — Source data Fig. 1 [file 44318_2025_540_MOESM27_ESM.zip › SD Figure 1/1A/Microscopy MEF_18 h.jpg]

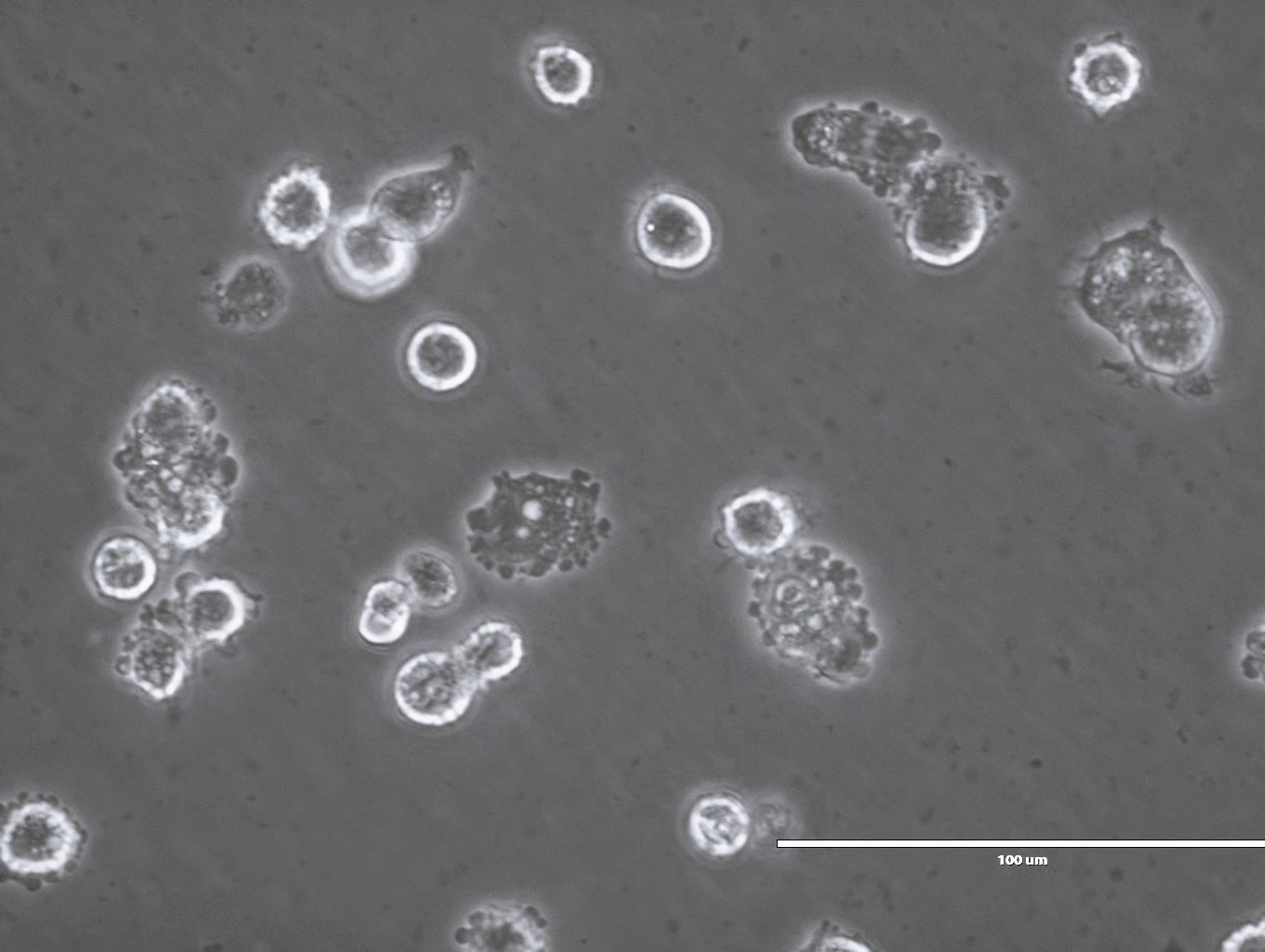

Supplement: Supplementary file 27 — Source data Fig. 1 [file 44318_2025_540_MOESM27_ESM.zip › SD Figure 1/1A/Microscopy MEF_2 h.jpg]

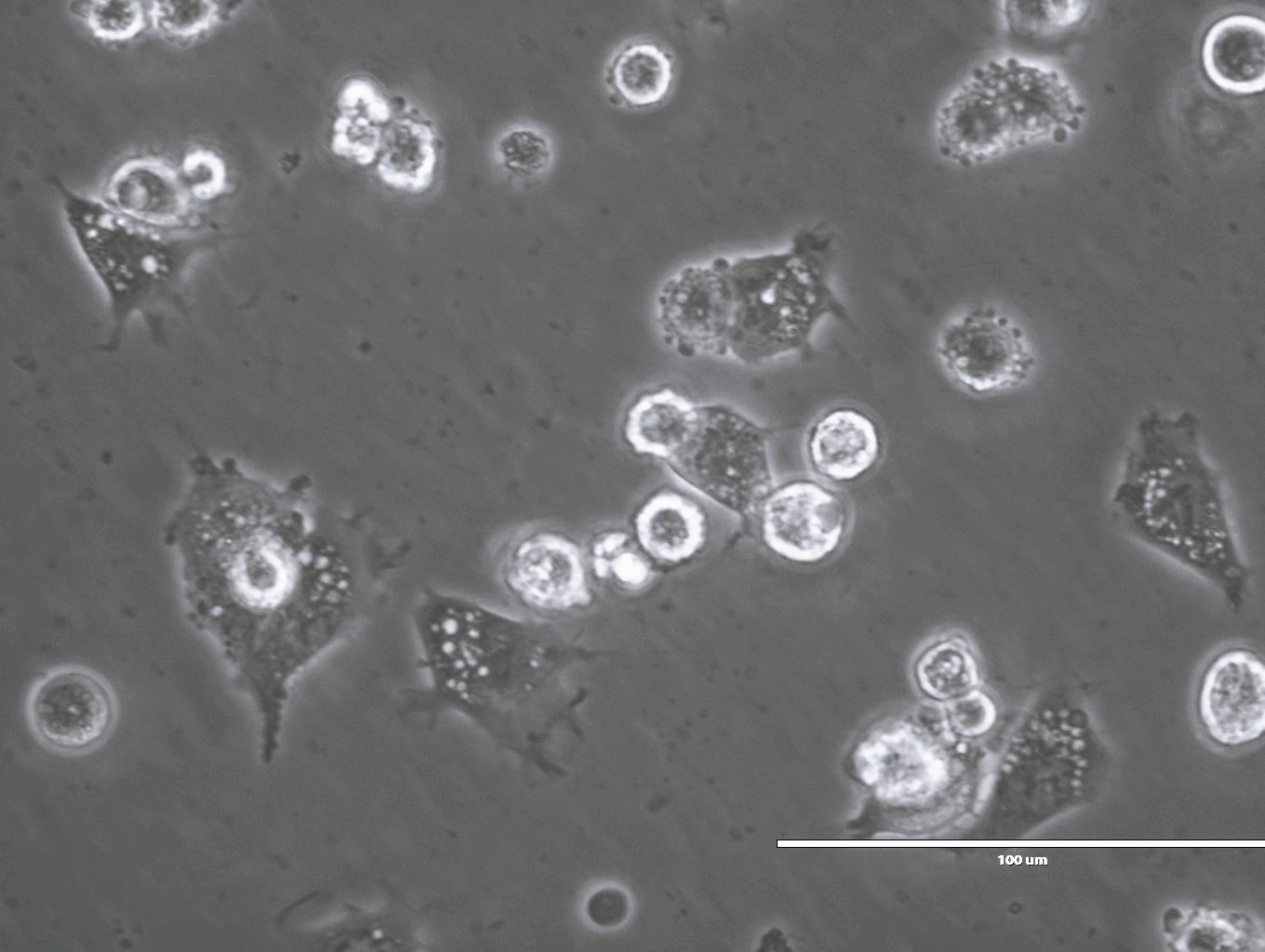

Supplement: Supplementary file 27 — Source data Fig. 1 [file 44318_2025_540_MOESM27_ESM.zip › SD Figure 1/1A/Microscopy MEF_3 h.jpg]

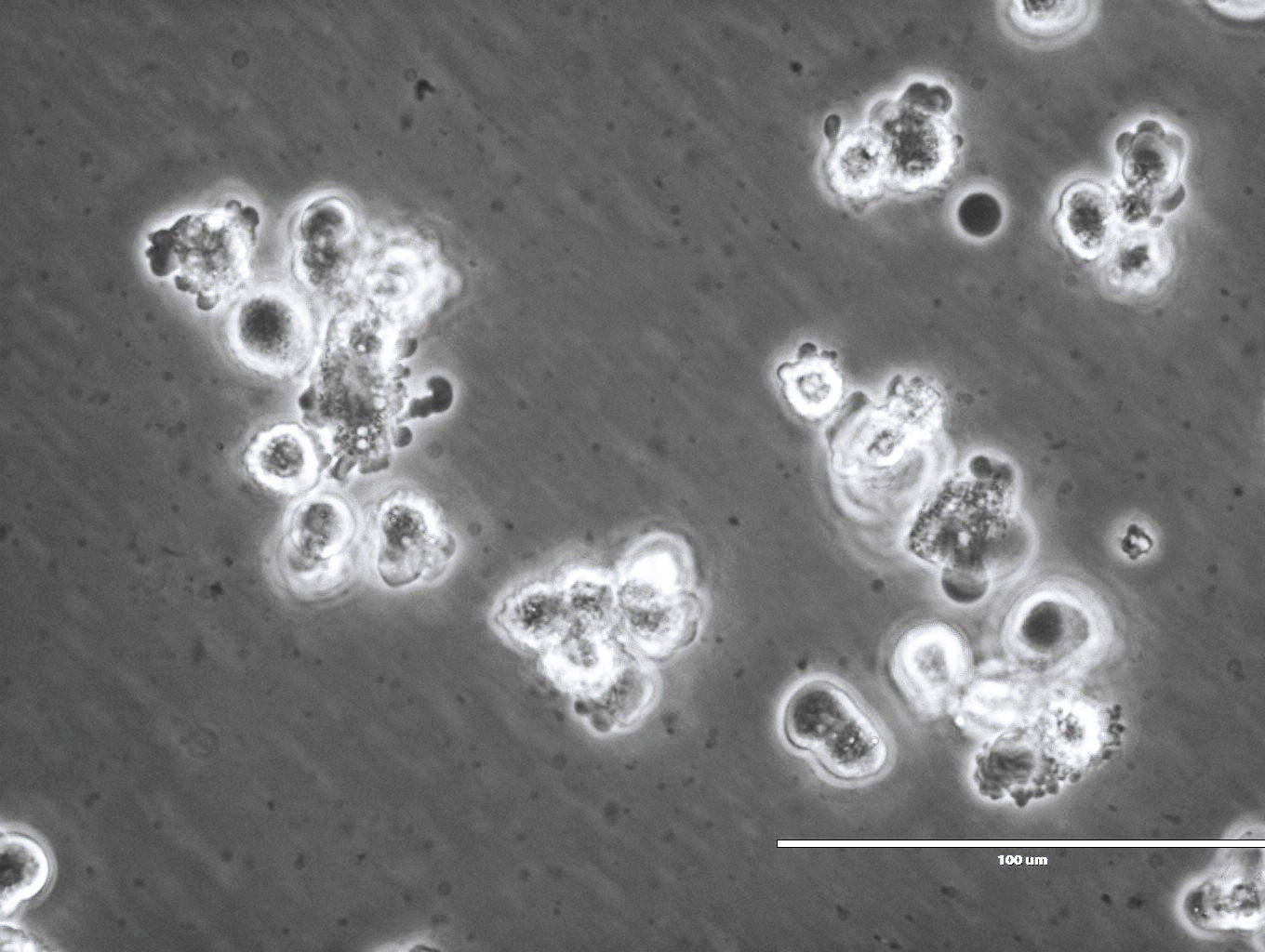

Supplement: Supplementary file 27 — Source data Fig. 1 [file 44318_2025_540_MOESM27_ESM.zip › SD Figure 1/1A/Microscopy MEF_30 min.jpg]

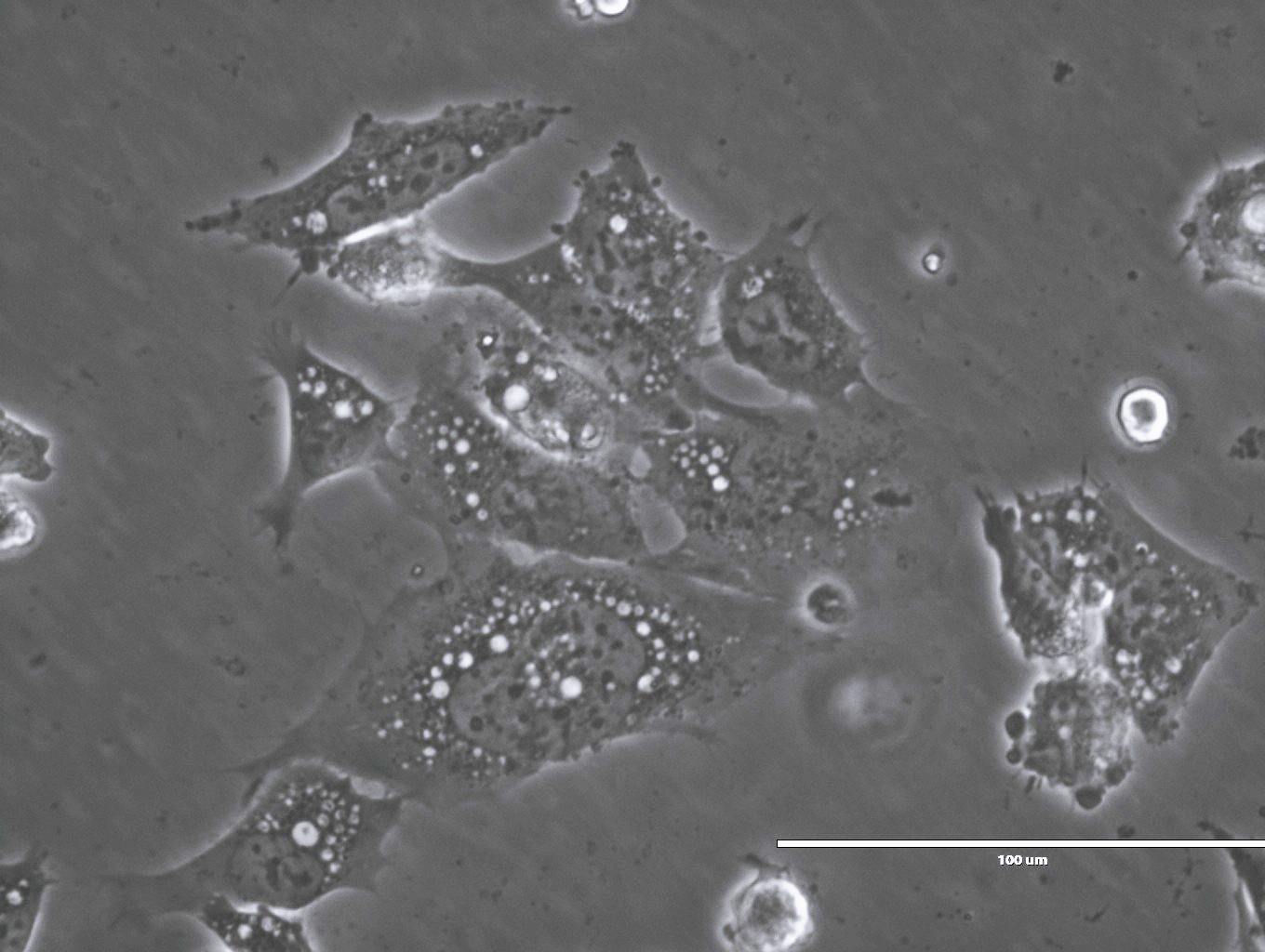

Supplement: Supplementary file 27 — Source data Fig. 1 [file 44318_2025_540_MOESM27_ESM.zip › SD Figure 1/1A/Microscopy MEF_6 h.jpg]

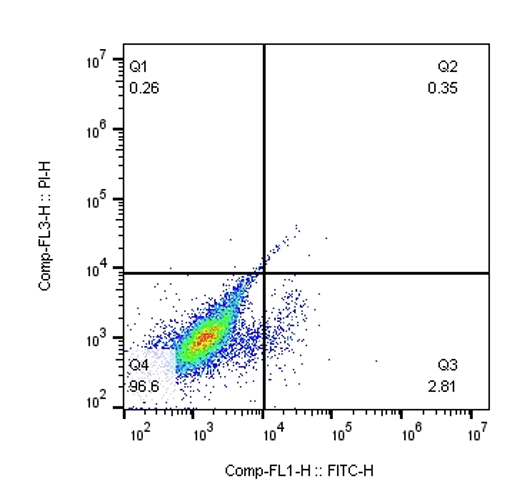

Supplement: Supplementary file 27 — Source data Fig. 1 [file 44318_2025_540_MOESM27_ESM.zip › SD Figure 1/1G/FACS Control.png]

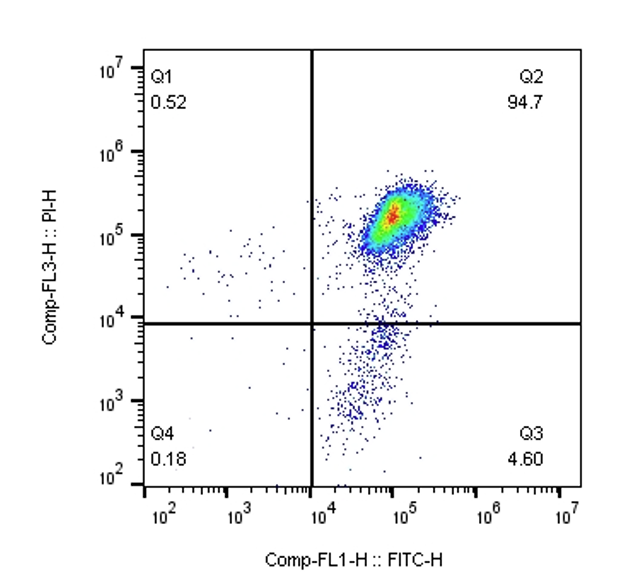

Supplement: Supplementary file 27 — Source data Fig. 1 [file 44318_2025_540_MOESM27_ESM.zip › SD Figure 1/1G/FACS CXH+LLOMe 16h.png]

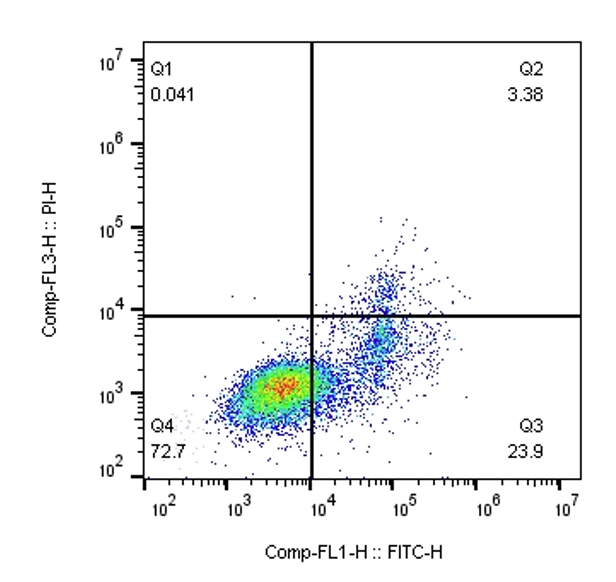

Supplement: Supplementary file 27 — Source data Fig. 1 [file 44318_2025_540_MOESM27_ESM.zip › SD Figure 1/1G/FACS LLOMe_16h.png]

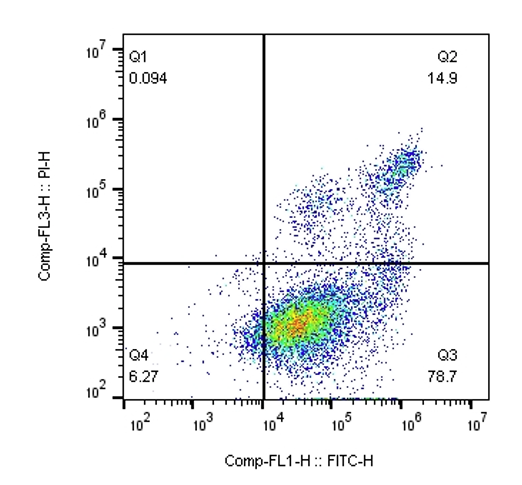

Supplement: Supplementary file 27 — Source data Fig. 1 [file 44318_2025_540_MOESM27_ESM.zip › SD Figure 1/1G/FACS LLOMe_30 min.png]

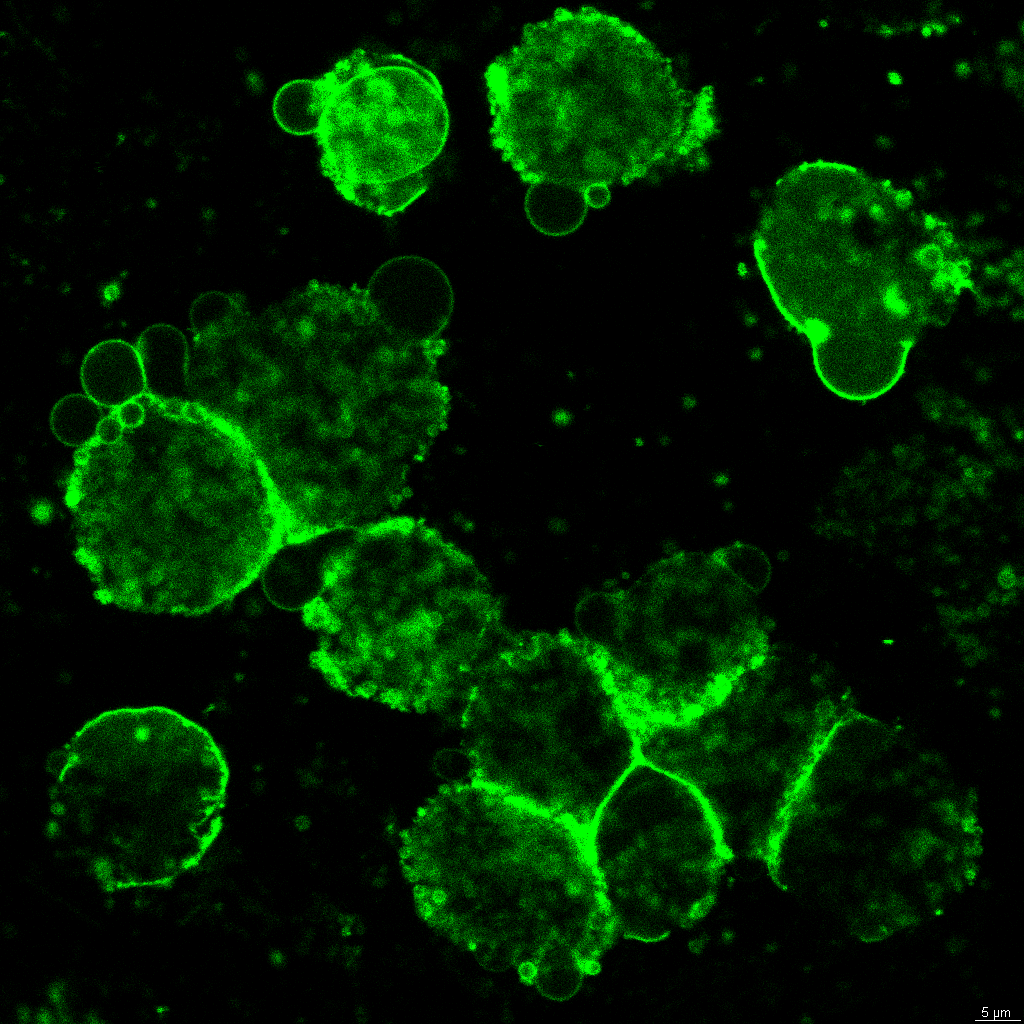

Supplement: Supplementary file 27 — Source data Fig. 1 [file 44318_2025_540_MOESM27_ESM.zip › SD Figure 1/1H/Microscopy Annexin FITC_12mM.tif]

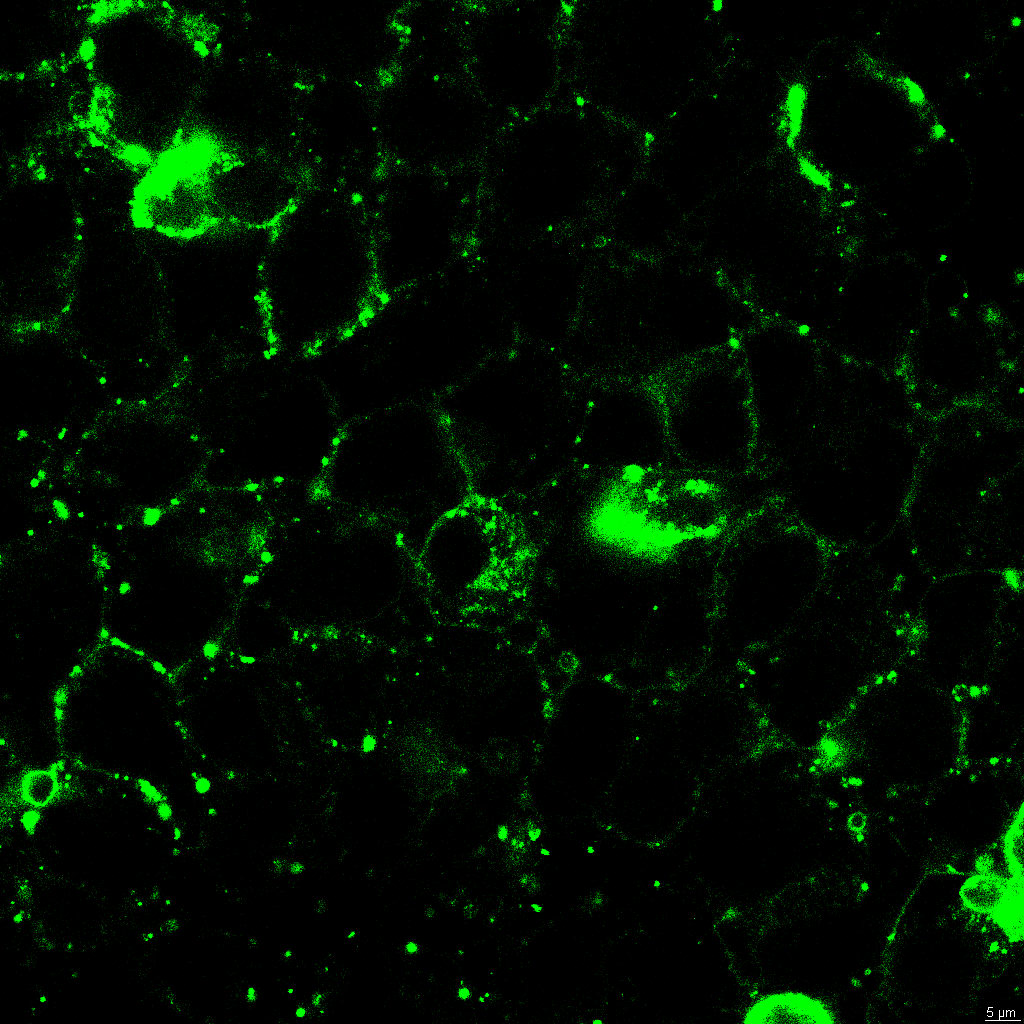

Supplement: Supplementary file 27 — Source data Fig. 1 [file 44318_2025_540_MOESM27_ESM.zip › SD Figure 1/1H/Microscopy Annexin FITC_4mM.tif]

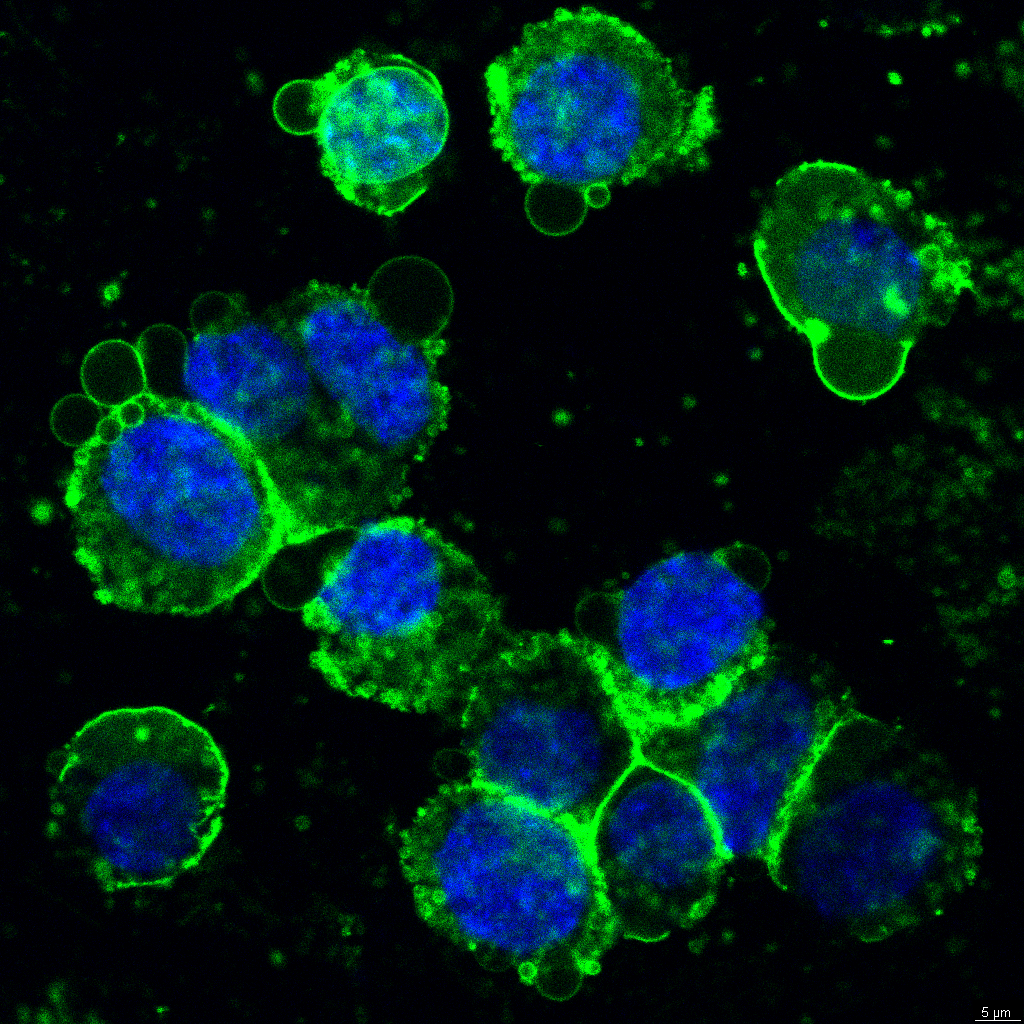

Supplement: Supplementary file 27 — Source data Fig. 1 [file 44318_2025_540_MOESM27_ESM.zip › SD Figure 1/1H/Microscopy Hoechst_Annexin FITC_12mM.tif]

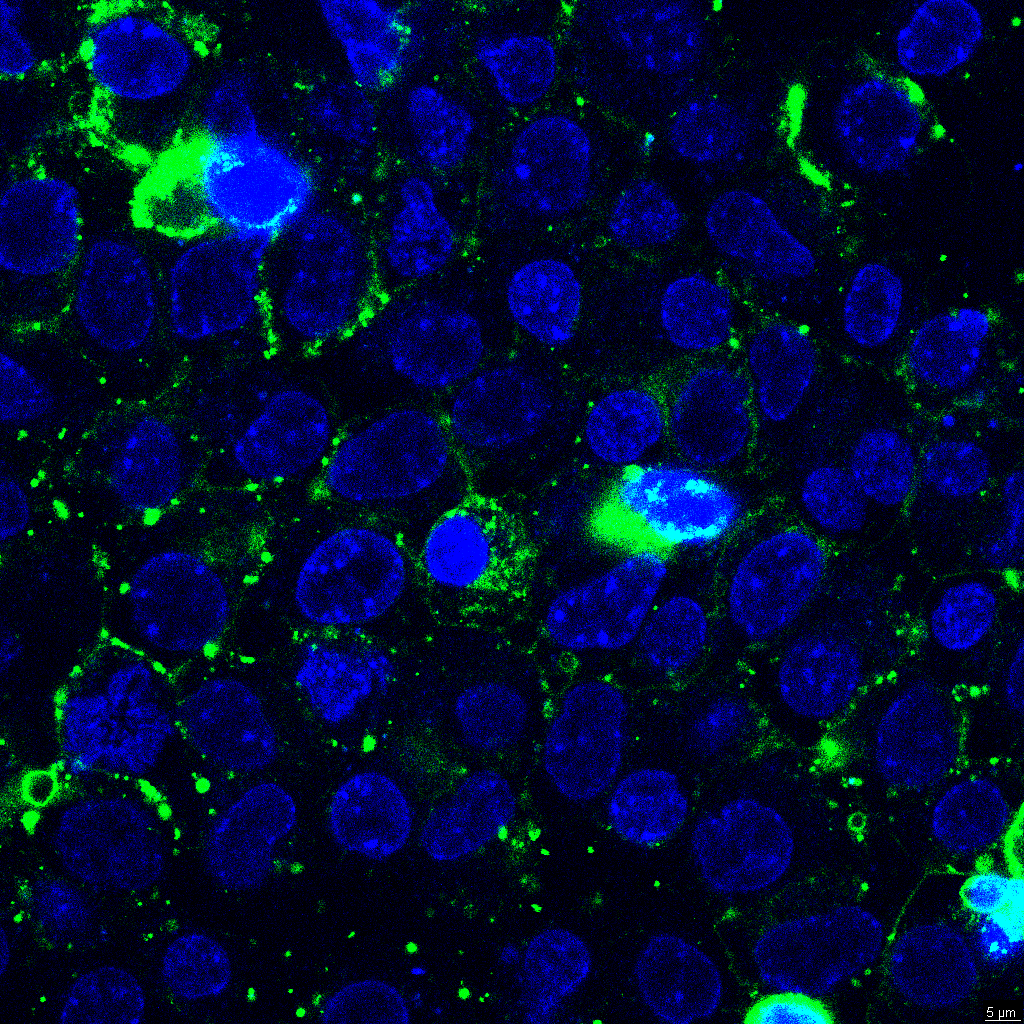

Supplement: Supplementary file 27 — Source data Fig. 1 [file 44318_2025_540_MOESM27_ESM.zip › SD Figure 1/1H/Microscopy Hoechst_Annexin FITC_4mM.tiff]

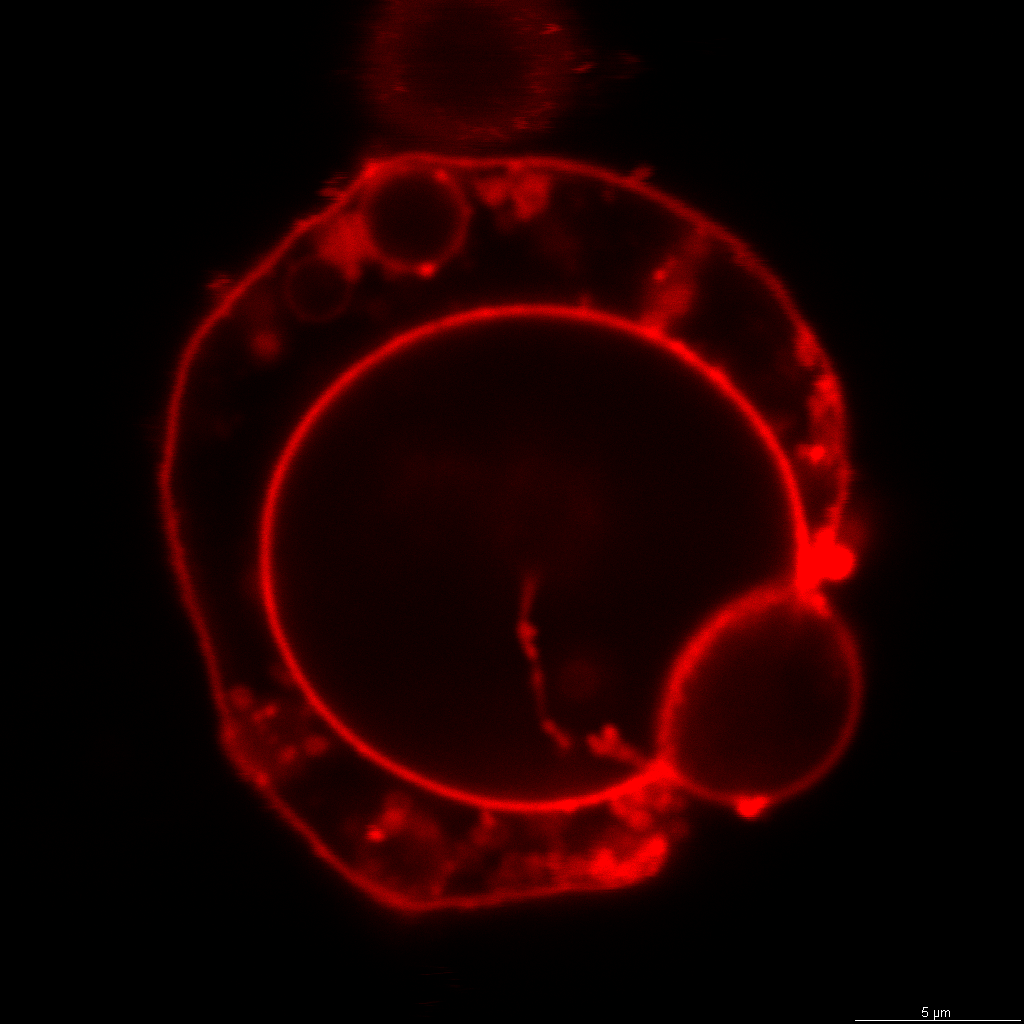

Supplement: Supplementary file 27 — Source data Fig. 1 [file 44318_2025_540_MOESM27_ESM.zip › SD Figure 1/1I/Microscopy 12mM_DIL.tif]

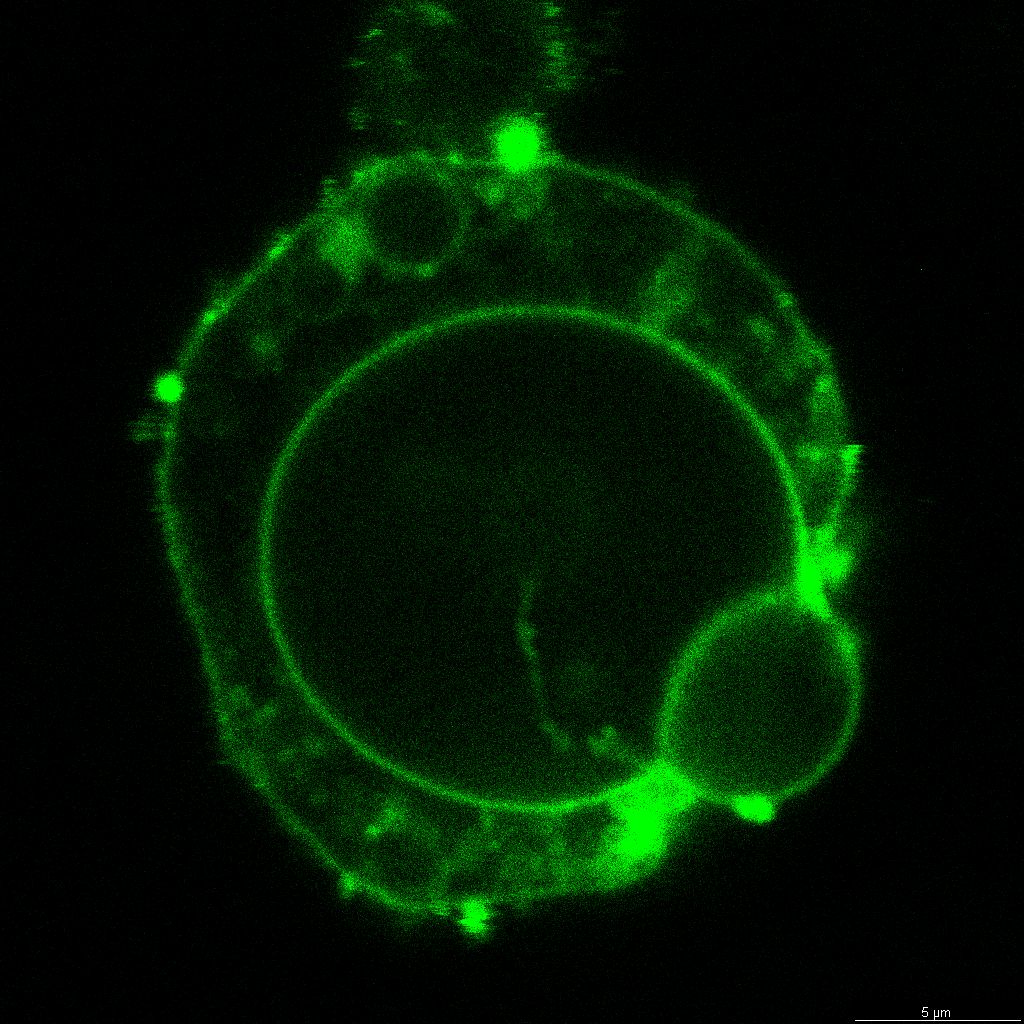

Supplement: Supplementary file 27 — Source data Fig. 1 [file 44318_2025_540_MOESM27_ESM.zip › SD Figure 1/1I/Microscopy 12mM_FITC.tif]

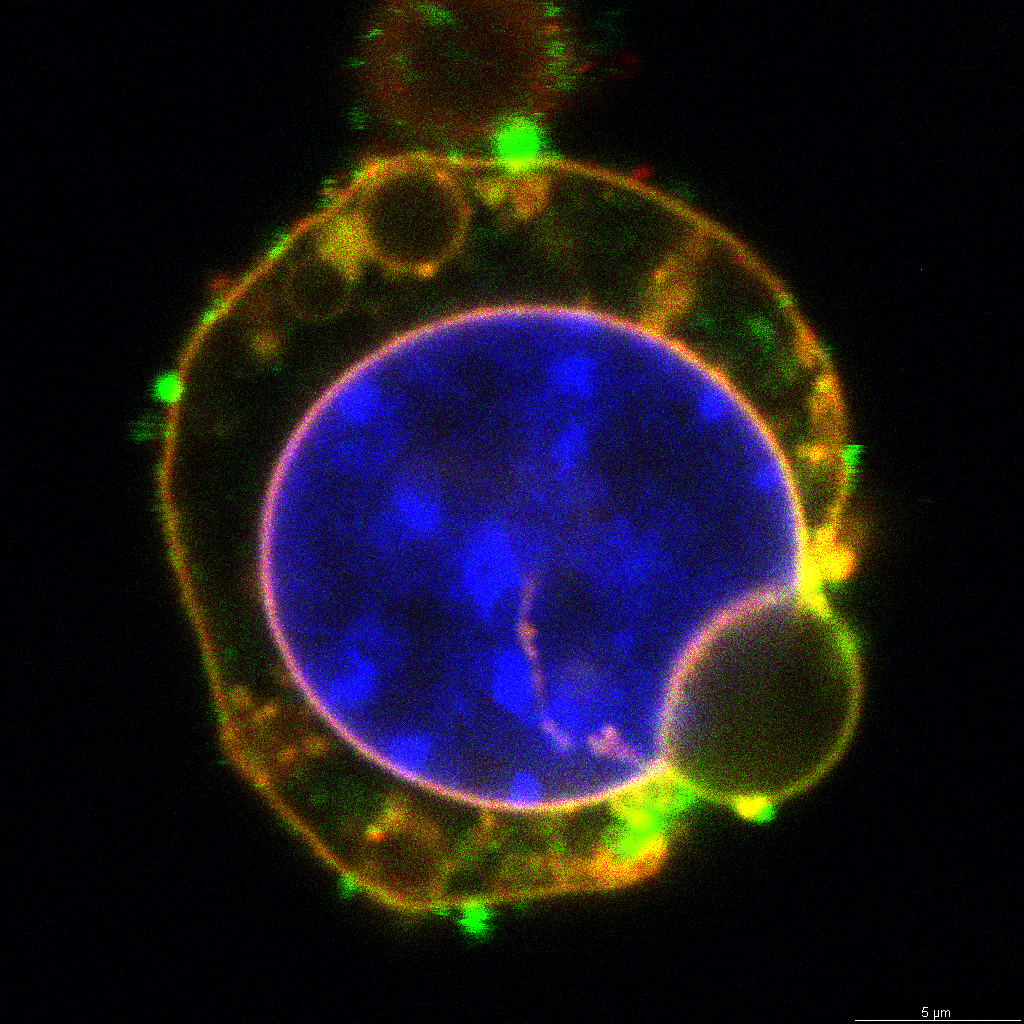

Supplement: Supplementary file 27 — Source data Fig. 1 [file 44318_2025_540_MOESM27_ESM.zip › SD Figure 1/1I/Microscopy 12mM_merge.tif]

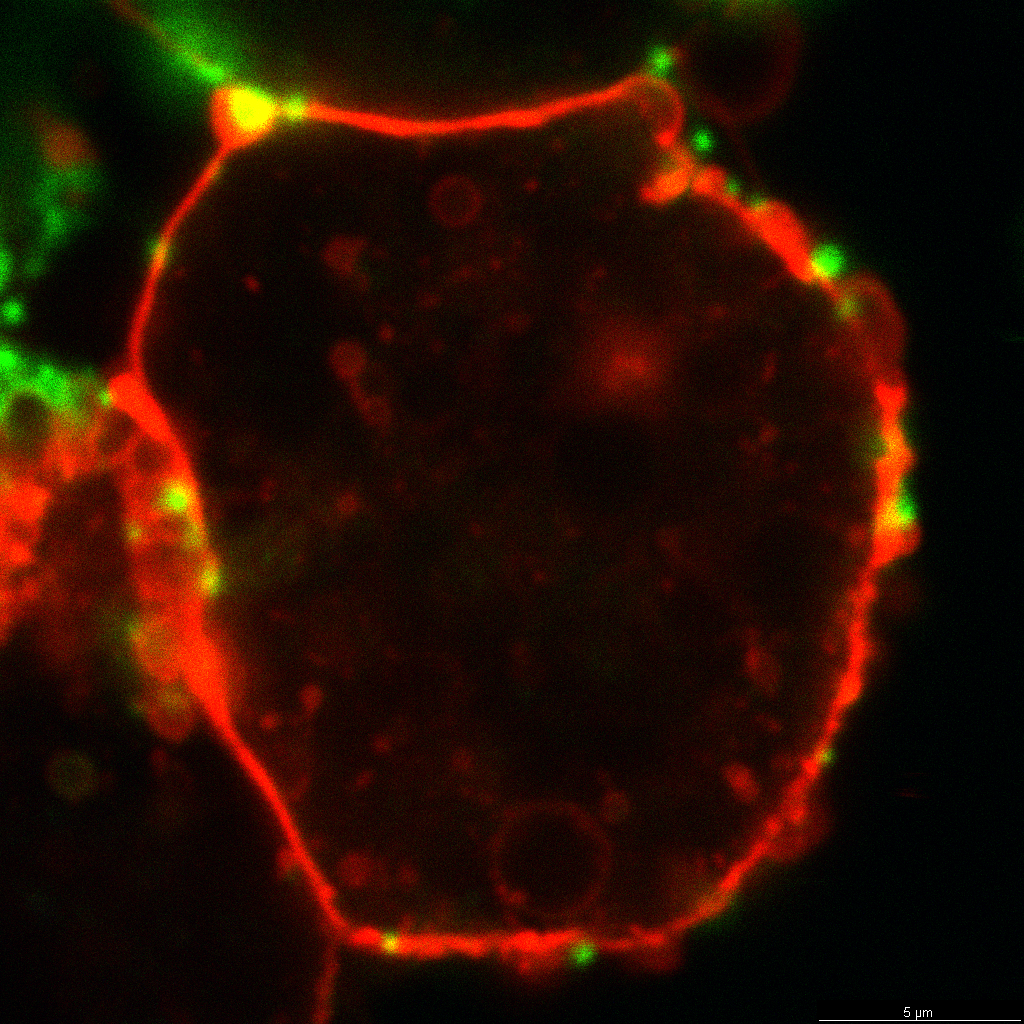

Supplement: Supplementary file 27 — Source data Fig. 1 [file 44318_2025_540_MOESM27_ESM.zip › SD Figure 1/1I/Microscopy Annexin FITC+DIL+DAPI_4mM.tif]

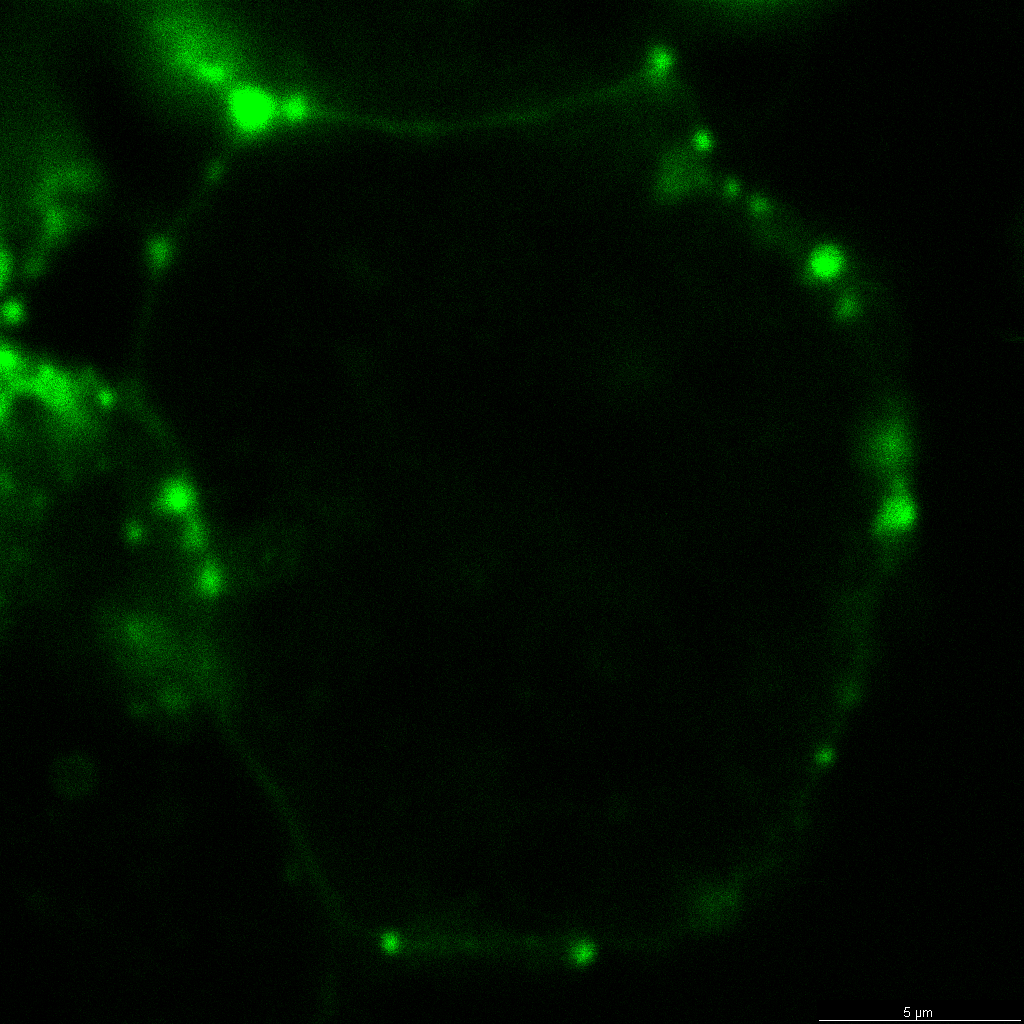

Supplement: Supplementary file 27 — Source data Fig. 1 [file 44318_2025_540_MOESM27_ESM.zip › SD Figure 1/1I/Microscopy Annexin FITC_4mM.tif]

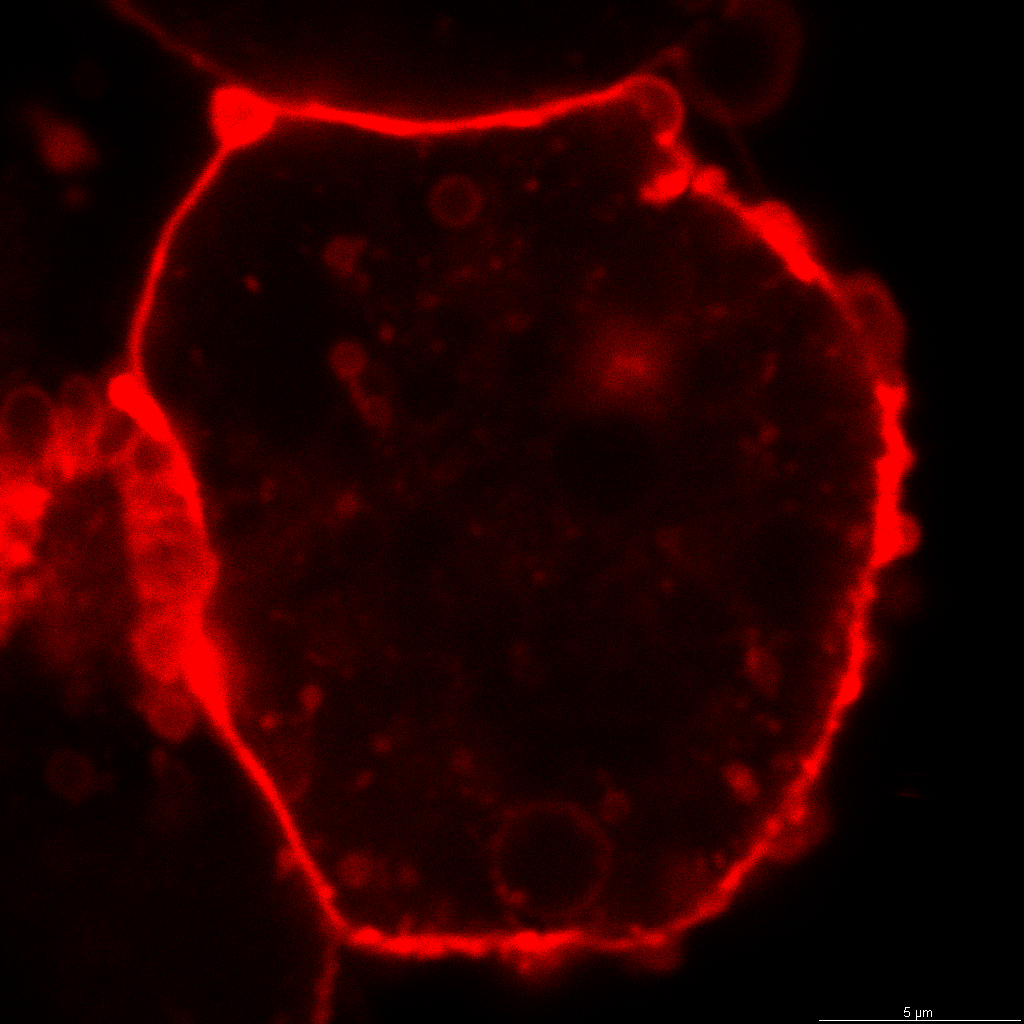

Supplement: Supplementary file 27 — Source data Fig. 1 [file 44318_2025_540_MOESM27_ESM.zip › SD Figure 1/1I/Microscopy DIL_4mM.tif]

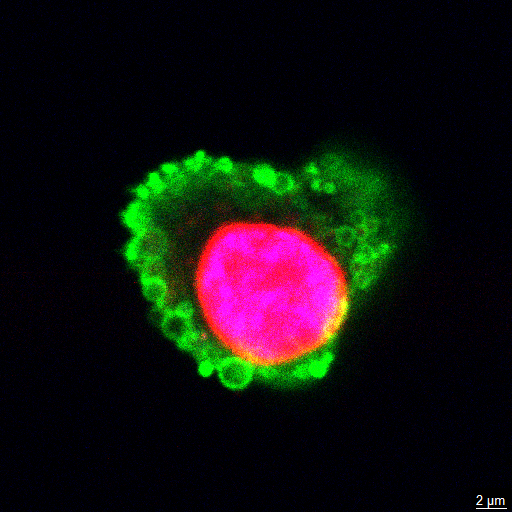

Supplement: Supplementary file 27 — Source data Fig. 1 [file 44318_2025_540_MOESM27_ESM.zip › SD Figure 1/1J/Microscopy 12mM merge.tif]

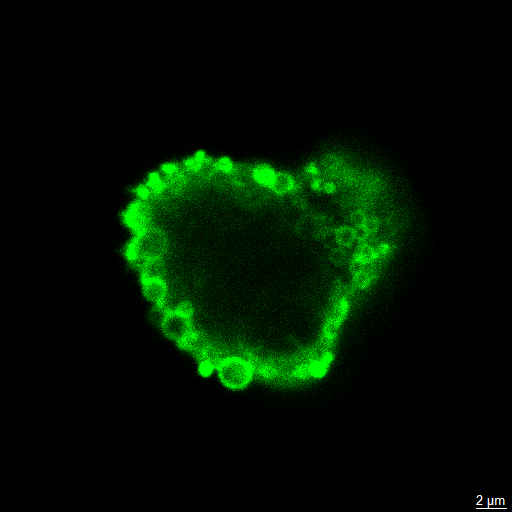

Supplement: Supplementary file 27 — Source data Fig. 1 [file 44318_2025_540_MOESM27_ESM.zip › SD Figure 1/1J/Microscopy 12mM_Annexin FITC.tif]

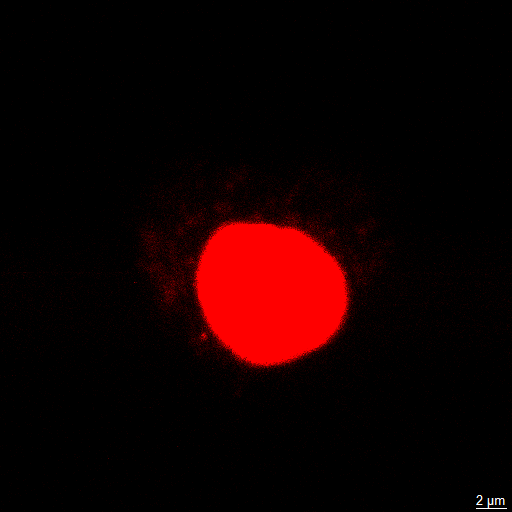

Supplement: Supplementary file 27 — Source data Fig. 1 [file 44318_2025_540_MOESM27_ESM.zip › SD Figure 1/1J/Microscopy 12mM_EtBr.tif]

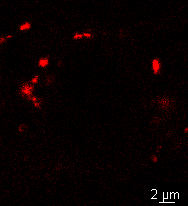

Supplement: Supplementary file 27 — Source data Fig. 1 [file 44318_2025_540_MOESM27_ESM.zip › SD Figure 1/1J/Microscopy 4mM LLOMe_EtBr.tif]

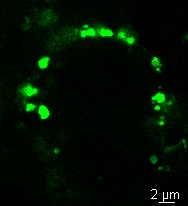

Supplement: Supplementary file 27 — Source data Fig. 1 [file 44318_2025_540_MOESM27_ESM.zip › SD Figure 1/1J/Microscopy 4mM_Annexin FITC.tif]

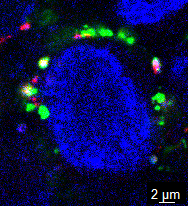

Supplement: Supplementary file 27 — Source data Fig. 1 [file 44318_2025_540_MOESM27_ESM.zip › SD Figure 1/1J/Microscopy 4mM_merge.tif]

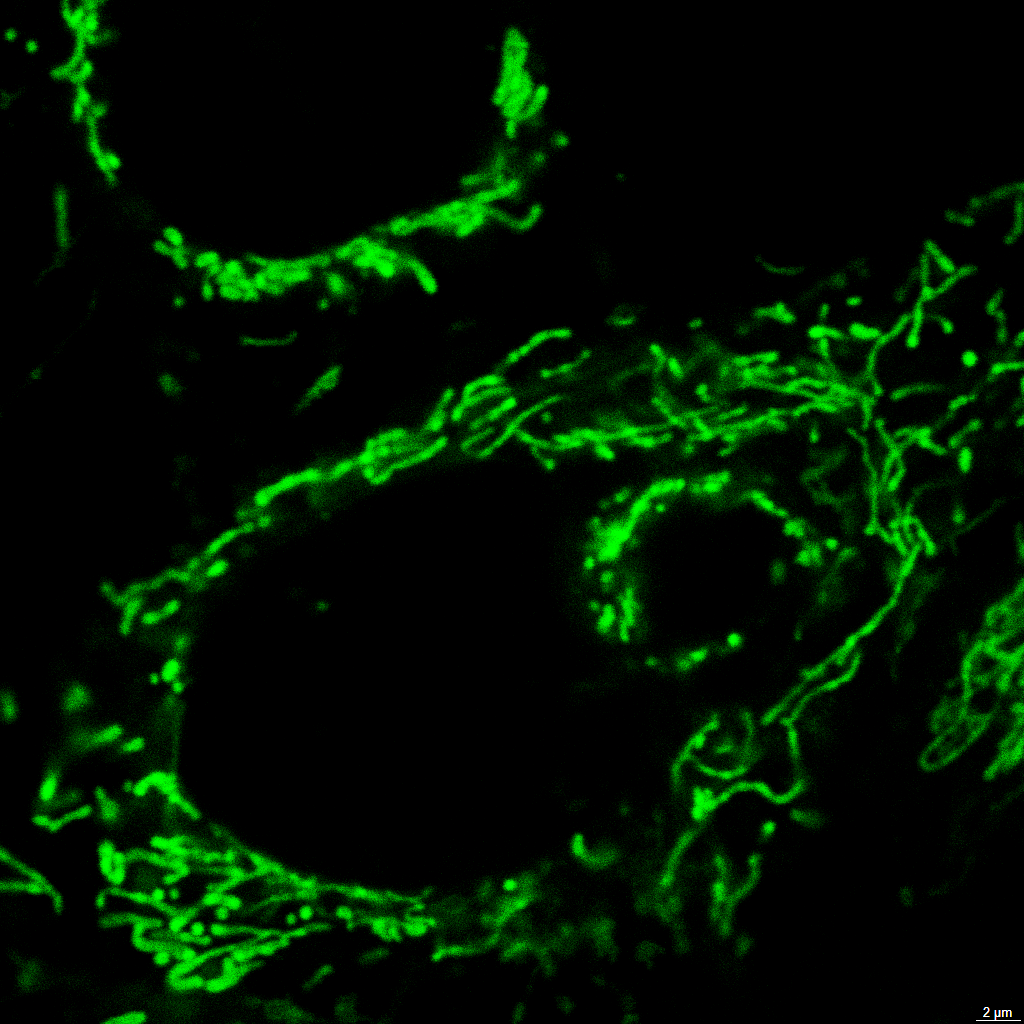

Supplement: Supplementary file 27 — Source data Fig. 1 [file 44318_2025_540_MOESM27_ESM.zip › SD Figure 1/1K/Microscopy Mitotracker 0 min.tif]

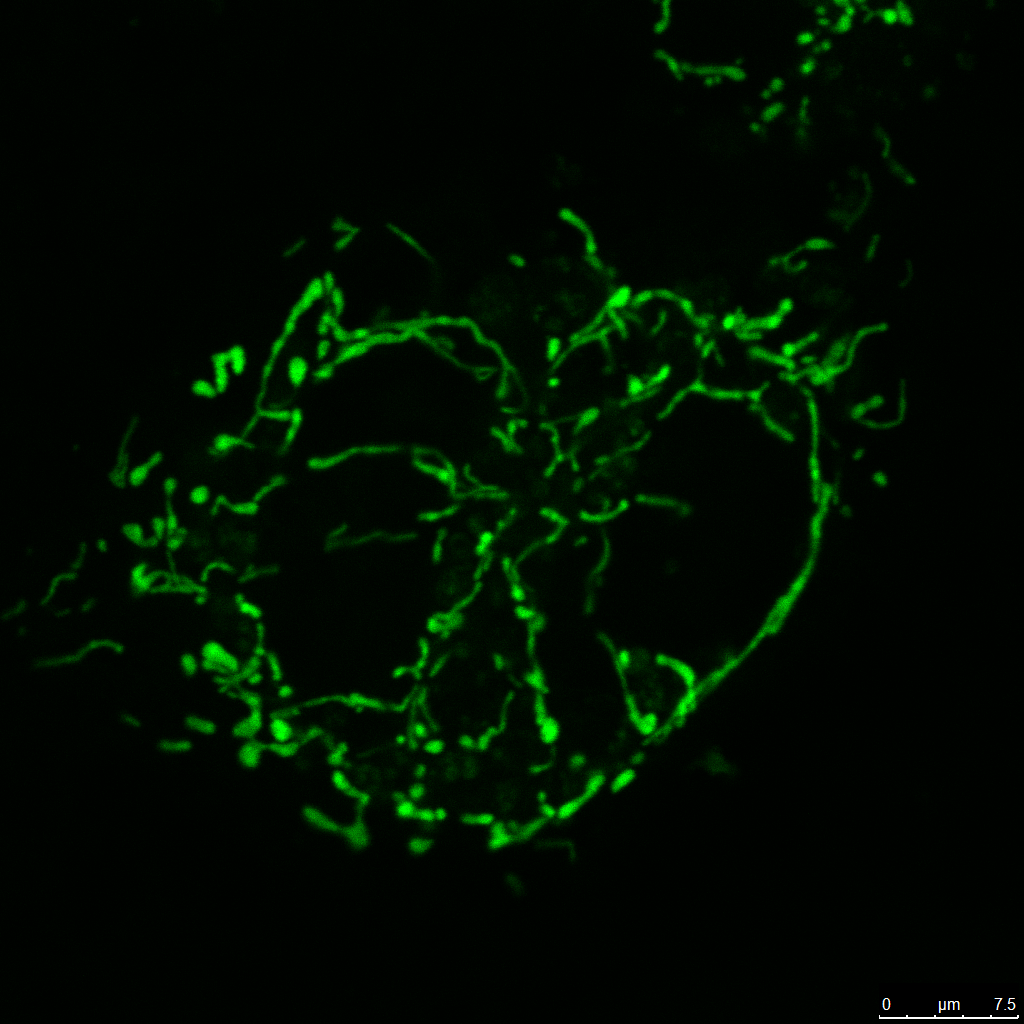

Supplement: Supplementary file 27 — Source data Fig. 1 [file 44318_2025_540_MOESM27_ESM.zip › SD Figure 1/1K/Microscopy Mitotracker 6h.tif]

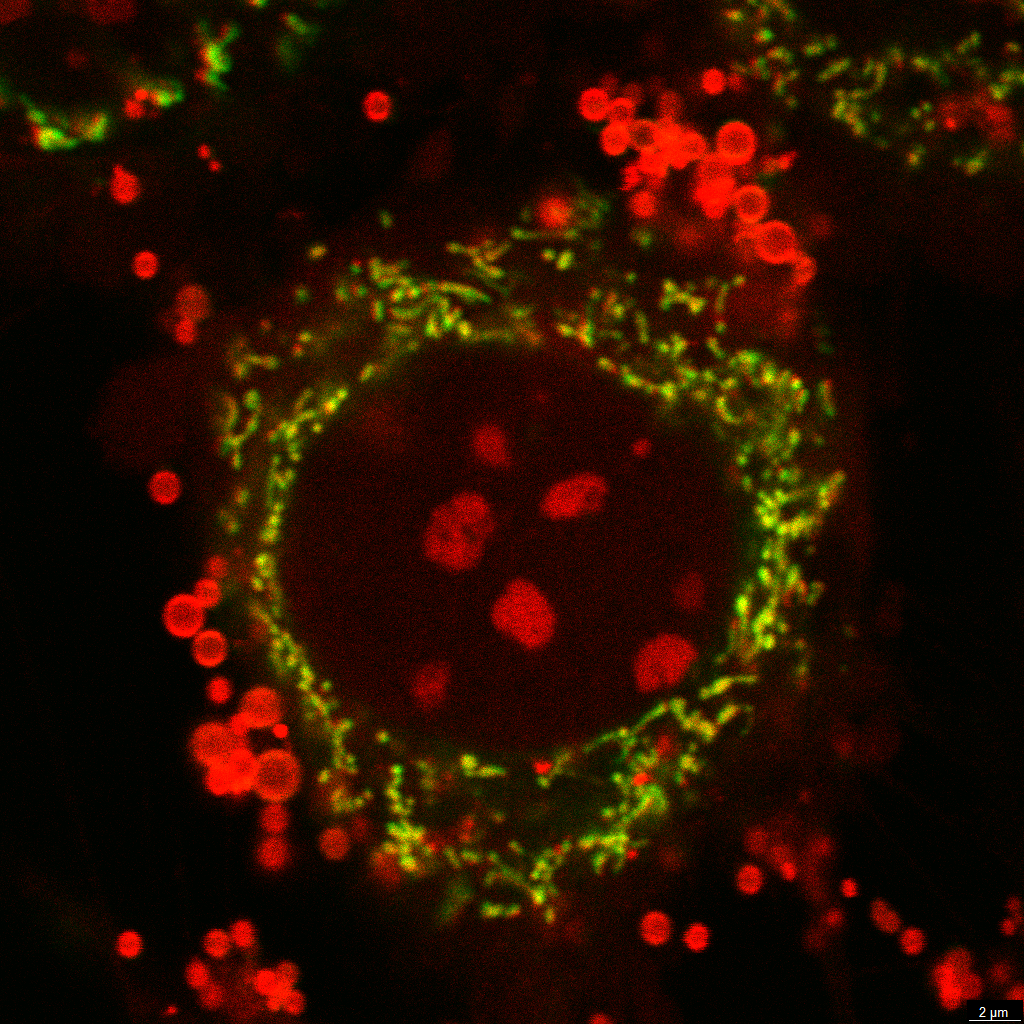

Supplement: Supplementary file 27 — Source data Fig. 1 [file 44318_2025_540_MOESM27_ESM.zip › SD Figure 1/1K/Microscopy Mitotracker and Rhod 2AM merge-1 5 min.tif]

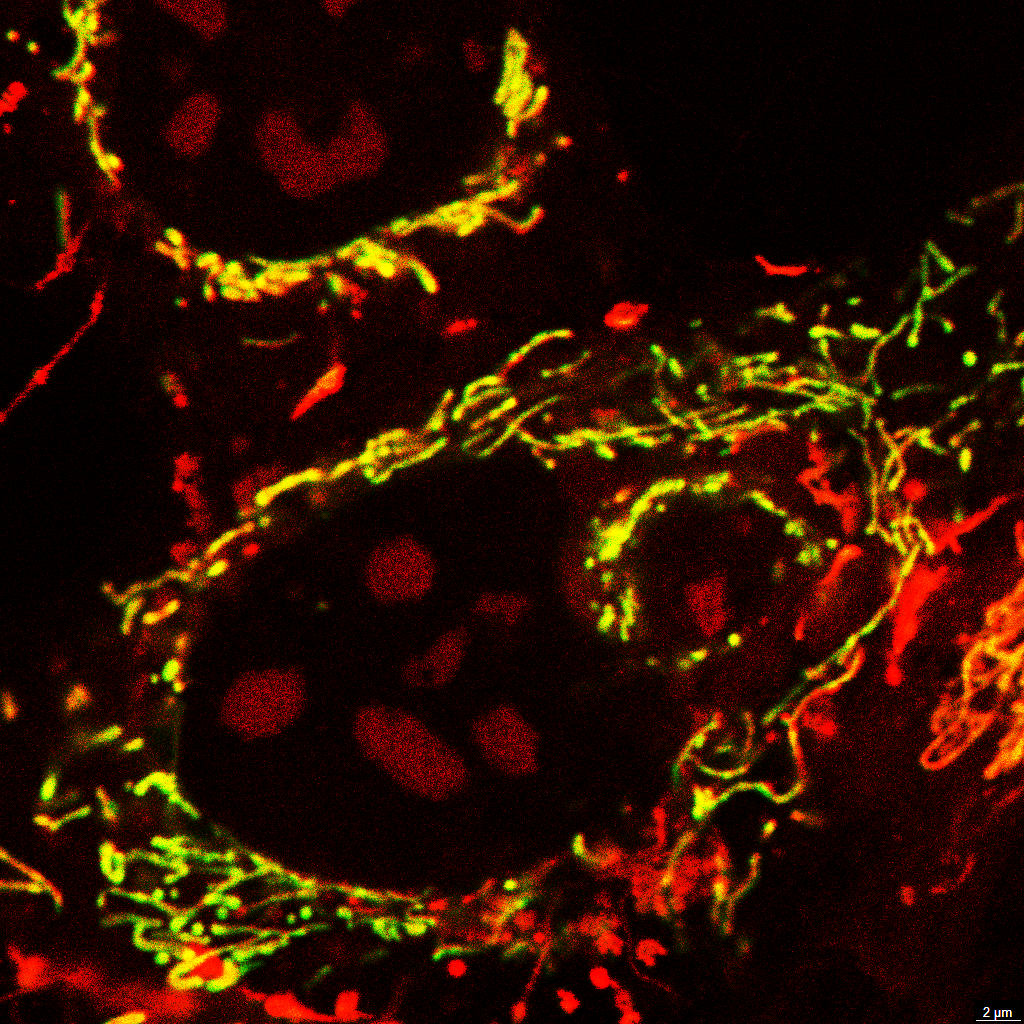

Supplement: Supplementary file 27 — Source data Fig. 1 [file 44318_2025_540_MOESM27_ESM.zip › SD Figure 1/1K/Microscopy Mitotracker and Rhod-2AM merge 0 min.tif]

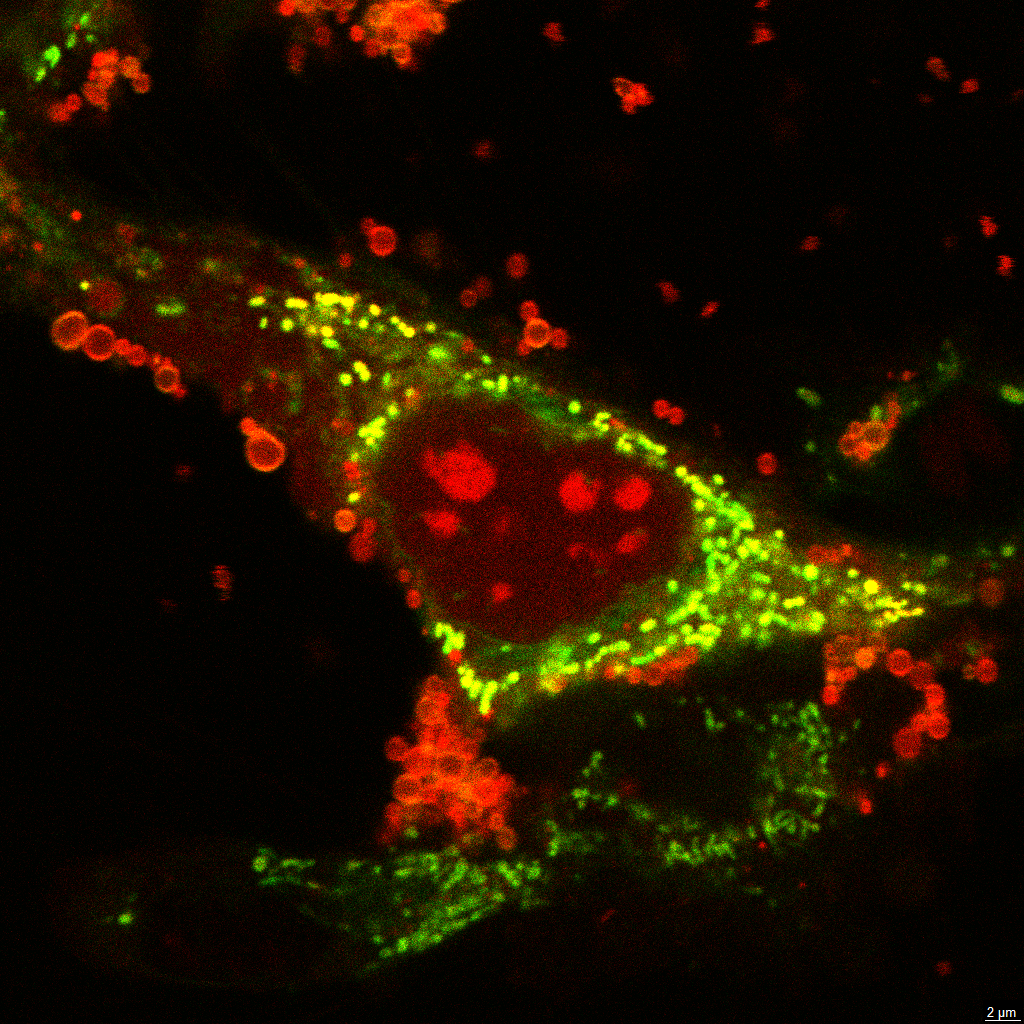

Supplement: Supplementary file 27 — Source data Fig. 1 [file 44318_2025_540_MOESM27_ESM.zip › SD Figure 1/1K/Microscopy Mitotracker and Rhod2AM merge-2 5 min.tif]

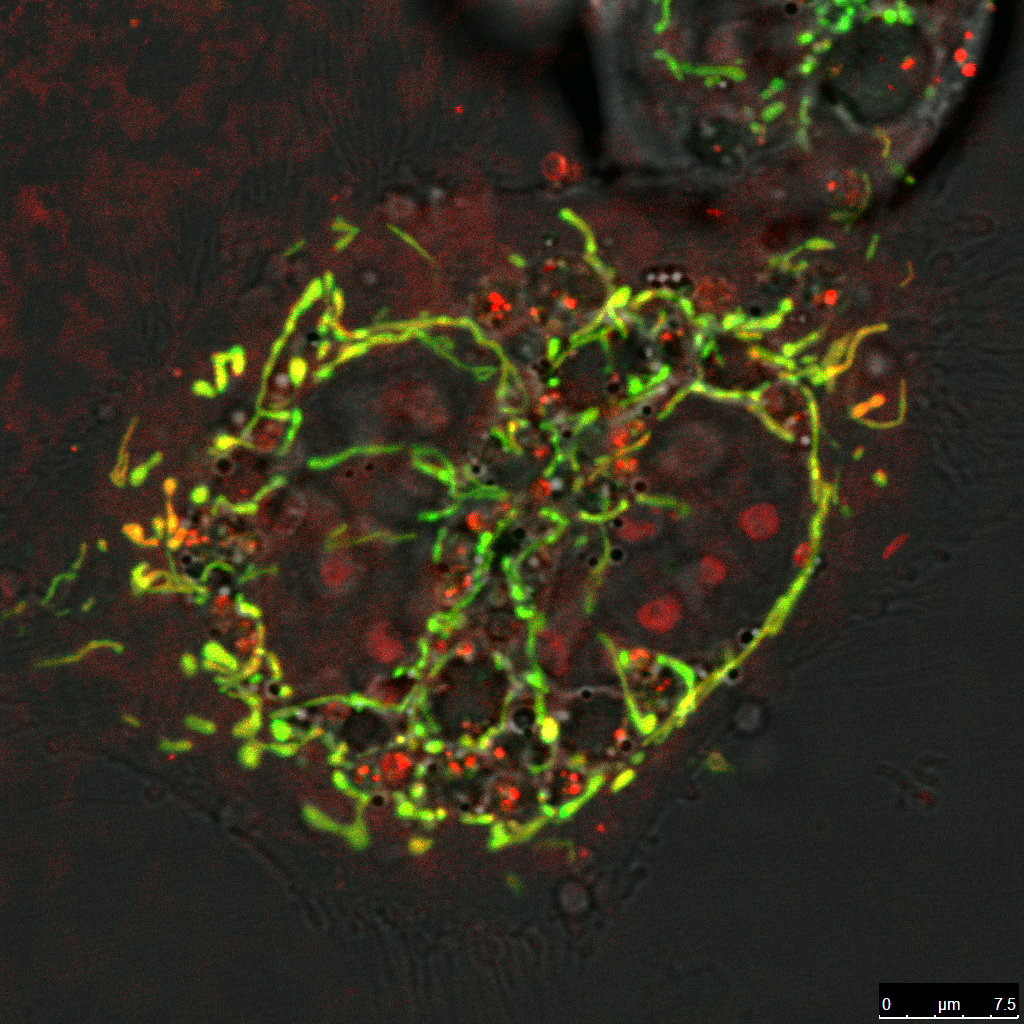

Supplement: Supplementary file 27 — Source data Fig. 1 [file 44318_2025_540_MOESM27_ESM.zip › SD Figure 1/1K/Microscopy Mitotracker _Rhod-2AM 6h.tif]

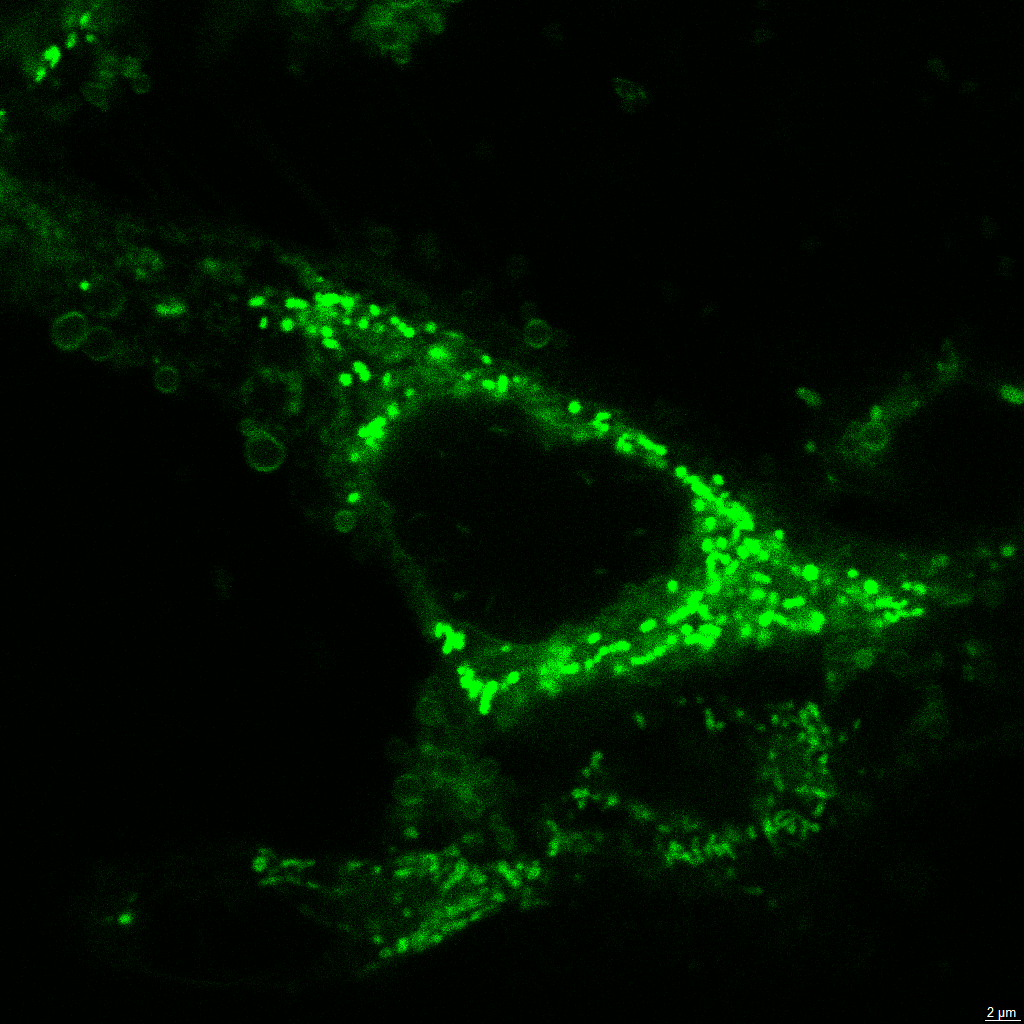

Supplement: Supplementary file 27 — Source data Fig. 1 [file 44318_2025_540_MOESM27_ESM.zip › SD Figure 1/1K/Microscopy Mitotracker-2 5 min.tif]

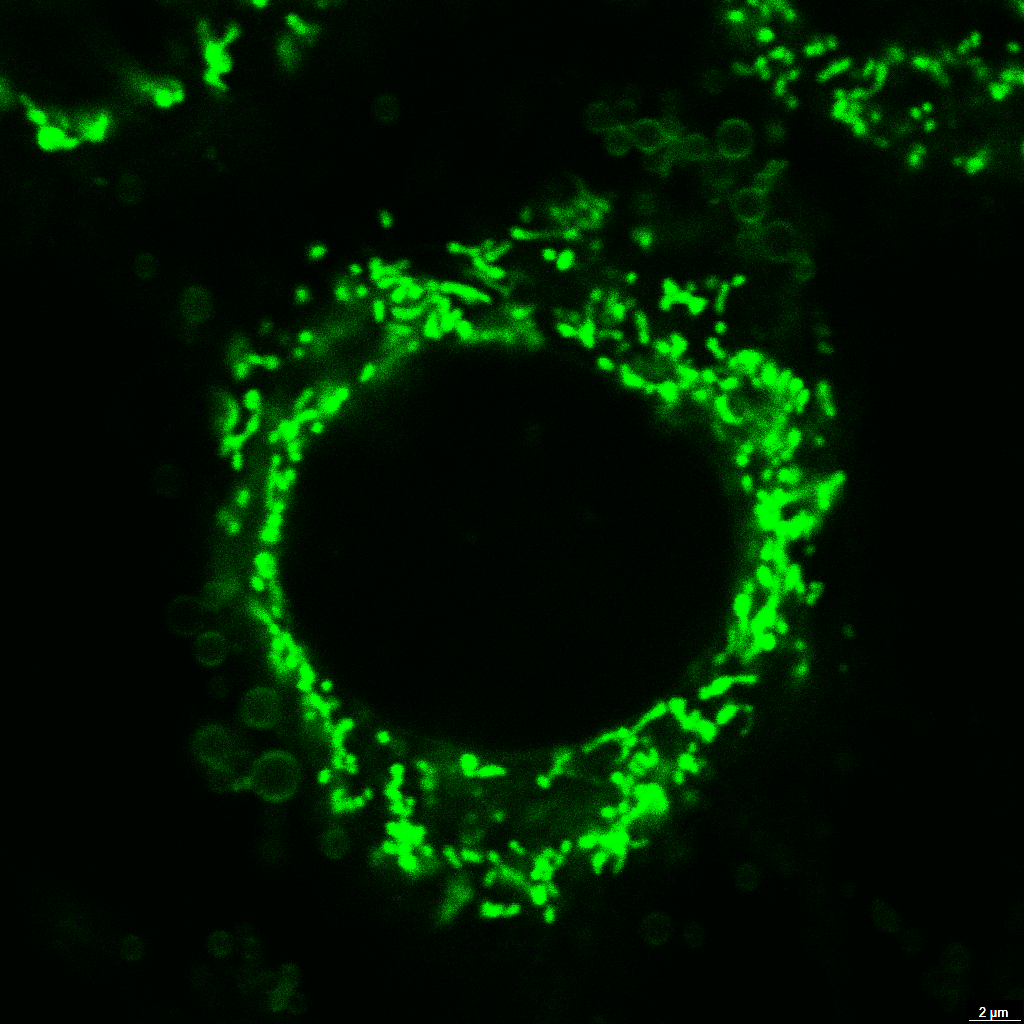

Supplement: Supplementary file 27 — Source data Fig. 1 [file 44318_2025_540_MOESM27_ESM.zip › SD Figure 1/1K/Microscopy Mitotracker_1 5 min.tif]

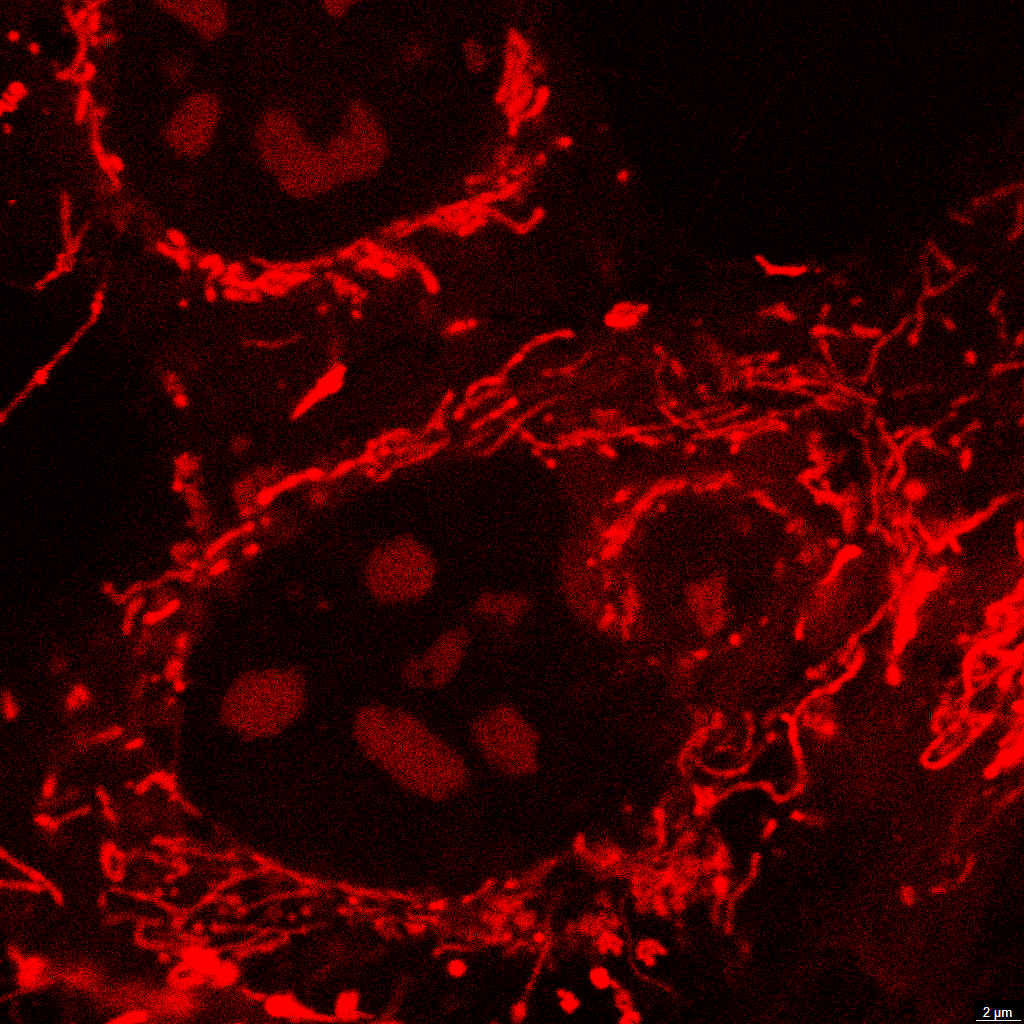

Supplement: Supplementary file 27 — Source data Fig. 1 [file 44318_2025_540_MOESM27_ESM.zip › SD Figure 1/1K/Microscopy Rhod 2AM 0 min.tif]

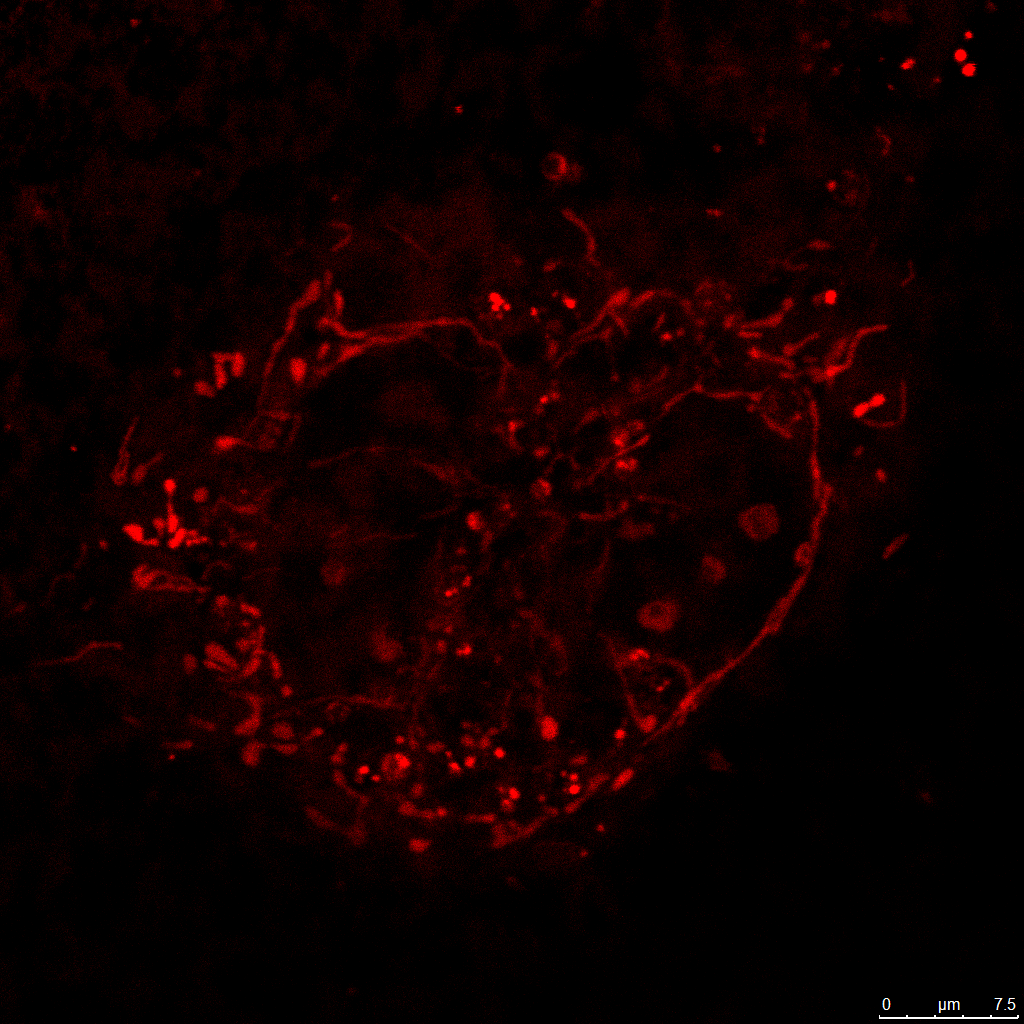

Supplement: Supplementary file 27 — Source data Fig. 1 [file 44318_2025_540_MOESM27_ESM.zip › SD Figure 1/1K/Microscopy Rhod-2AM 6h.tif]

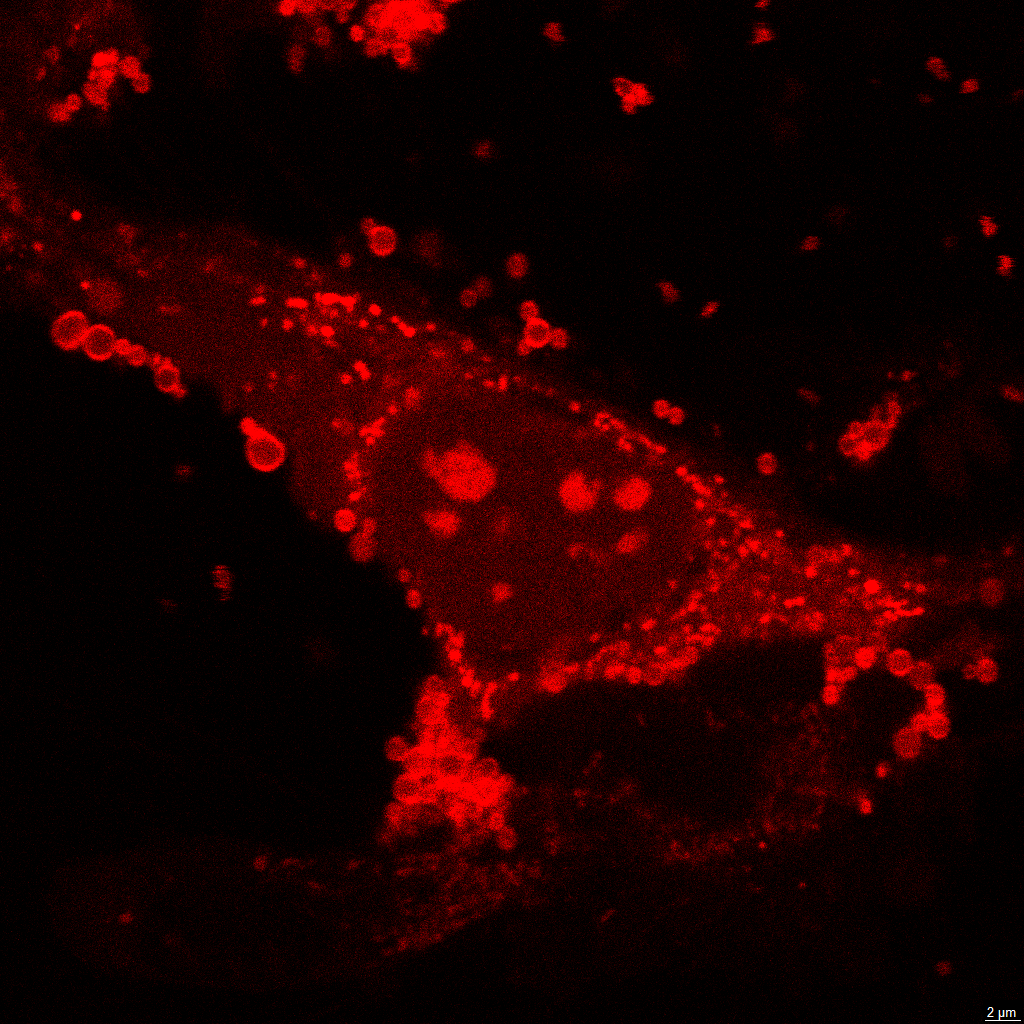

Supplement: Supplementary file 27 — Source data Fig. 1 [file 44318_2025_540_MOESM27_ESM.zip › SD Figure 1/1K/Microscopy Rhod2AM-2 5 min.tif]

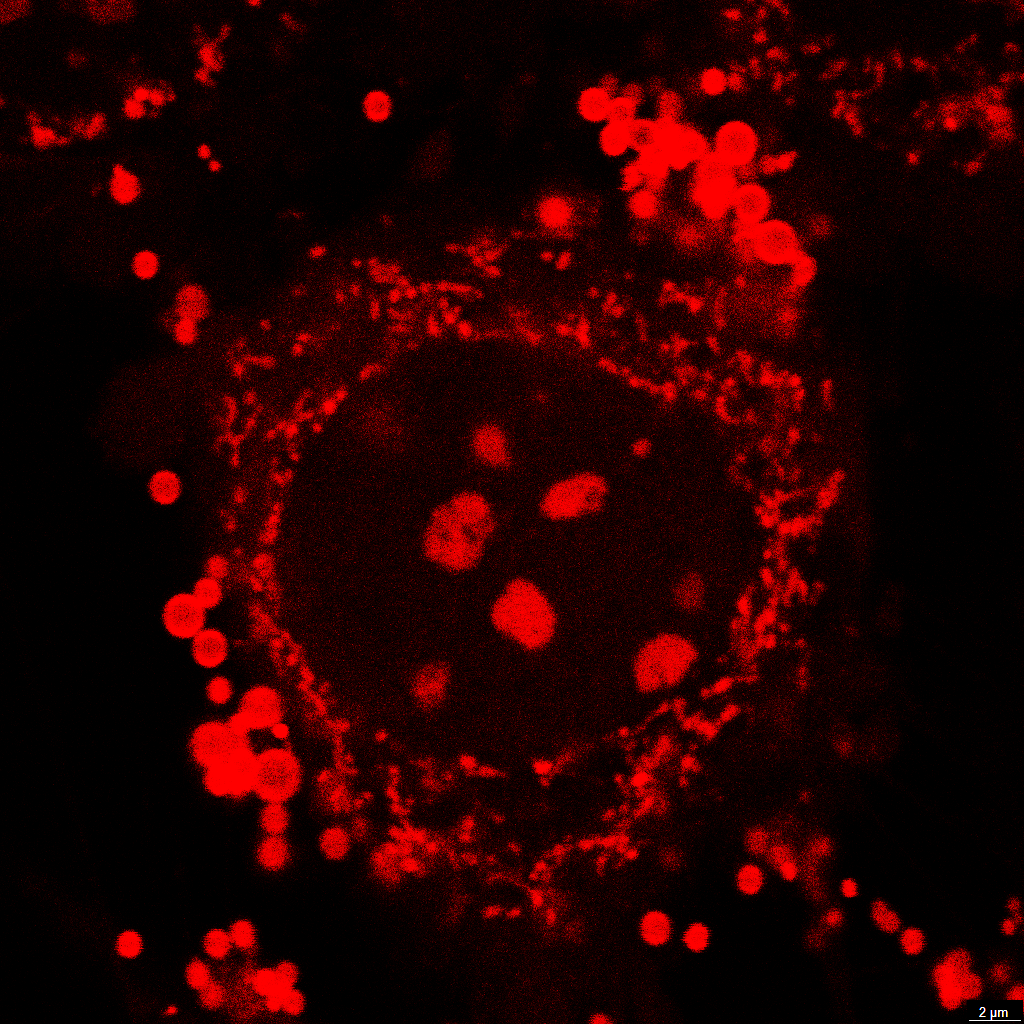

Supplement: Supplementary file 27 — Source data Fig. 1 [file 44318_2025_540_MOESM27_ESM.zip › SD Figure 1/1K/Microscopy Rhod2AM_1 5 min.tif]

Figure-1

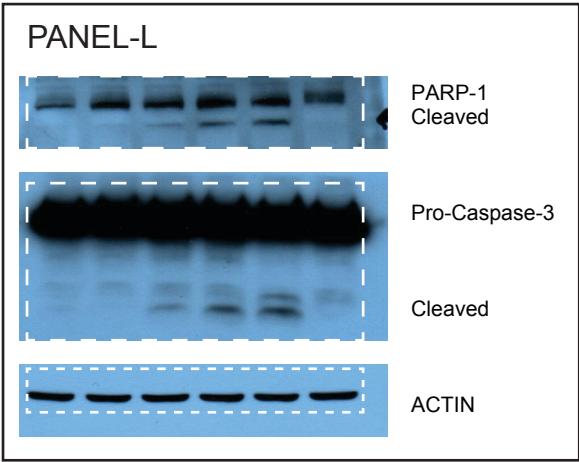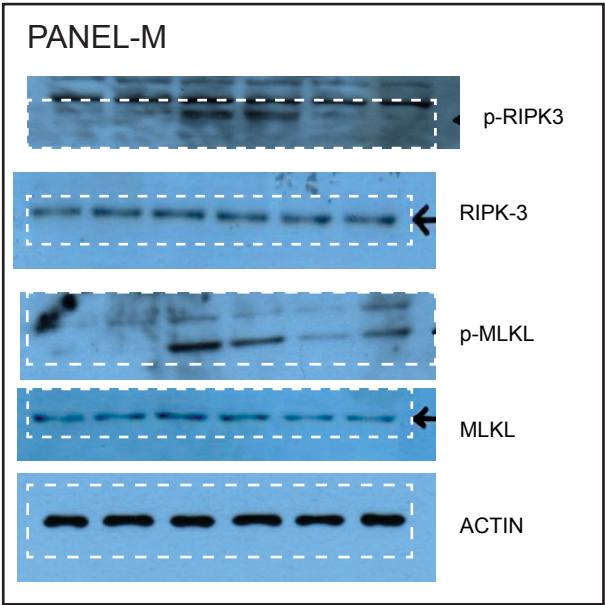

Supplement: Supplementary file 27 — Source data Fig. 1 [file 44318_2025_540_MOESM27_ESM.zip › SD Figure 1/1L and 1M/Western Blot.pdf]

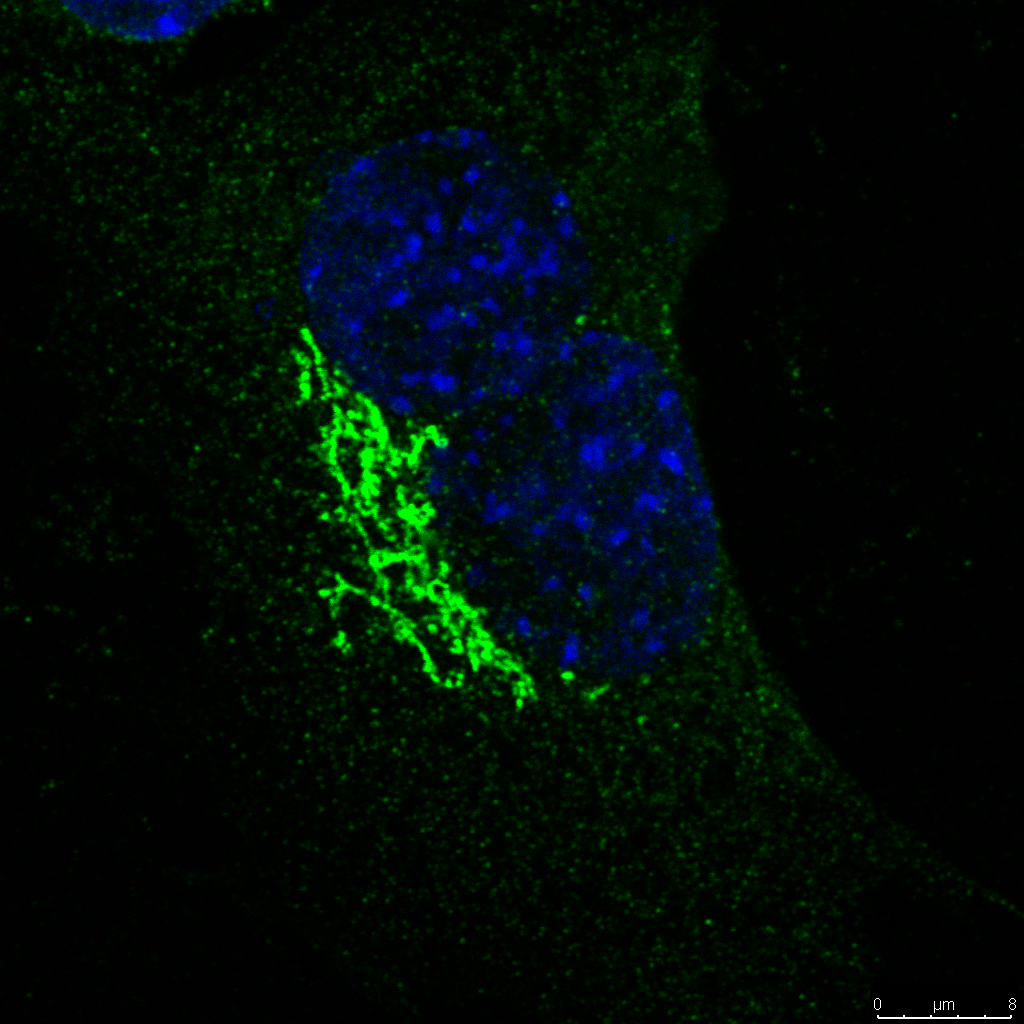

Supplement: Supplementary file 28 — Source data Fig. 2 [file 44318_2025_540_MOESM28_ESM.zip › SD Figure 2/2B/Microscopy 16h Golgi.tif]

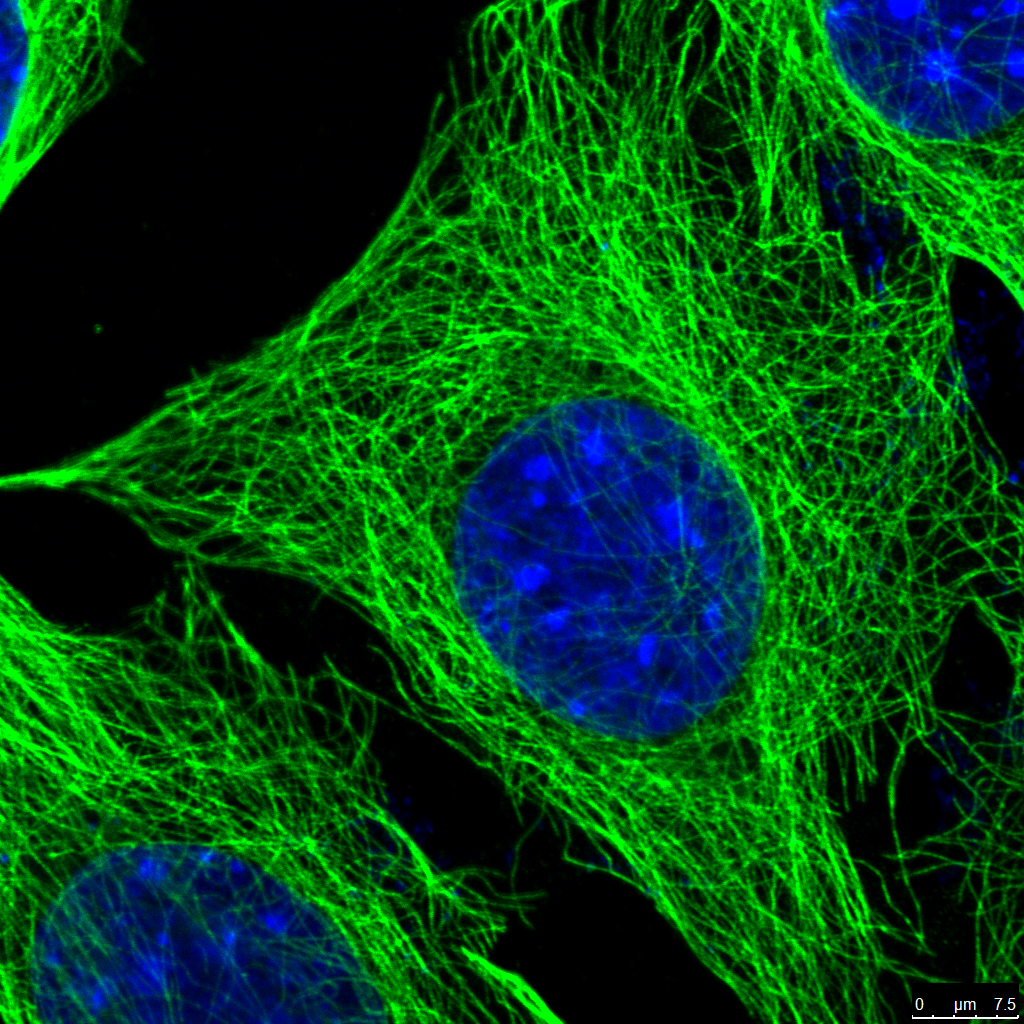

Supplement: Supplementary file 28 — Source data Fig. 2 [file 44318_2025_540_MOESM28_ESM.zip › SD Figure 2/2B/Microscopy 16h Microtubules.tif]

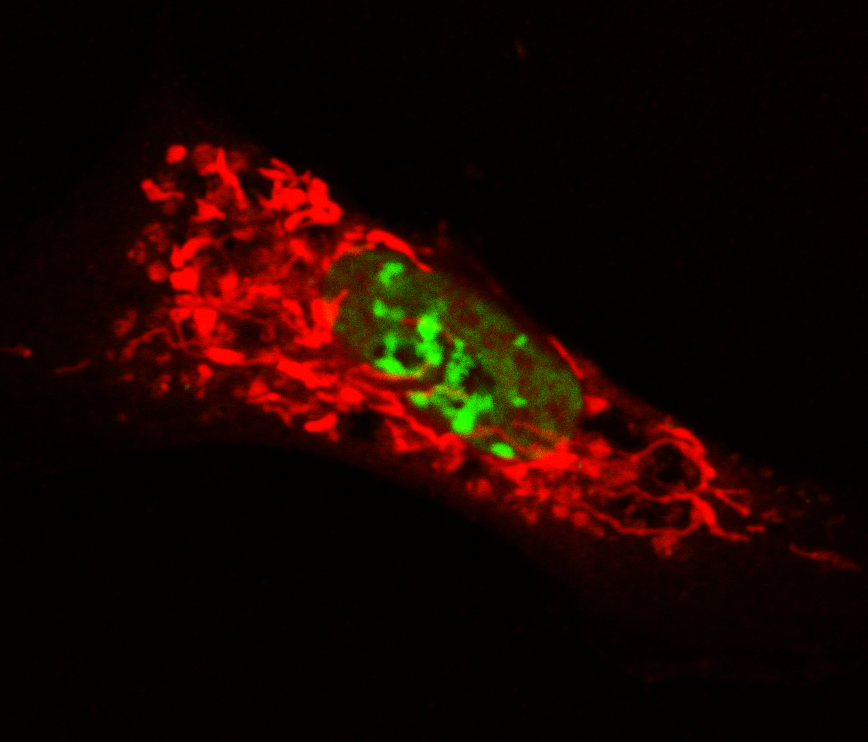

Supplement: Supplementary file 28 — Source data Fig. 2 [file 44318_2025_540_MOESM28_ESM.zip › SD Figure 2/2B/Microscopy 16h Mitochondria.tif]

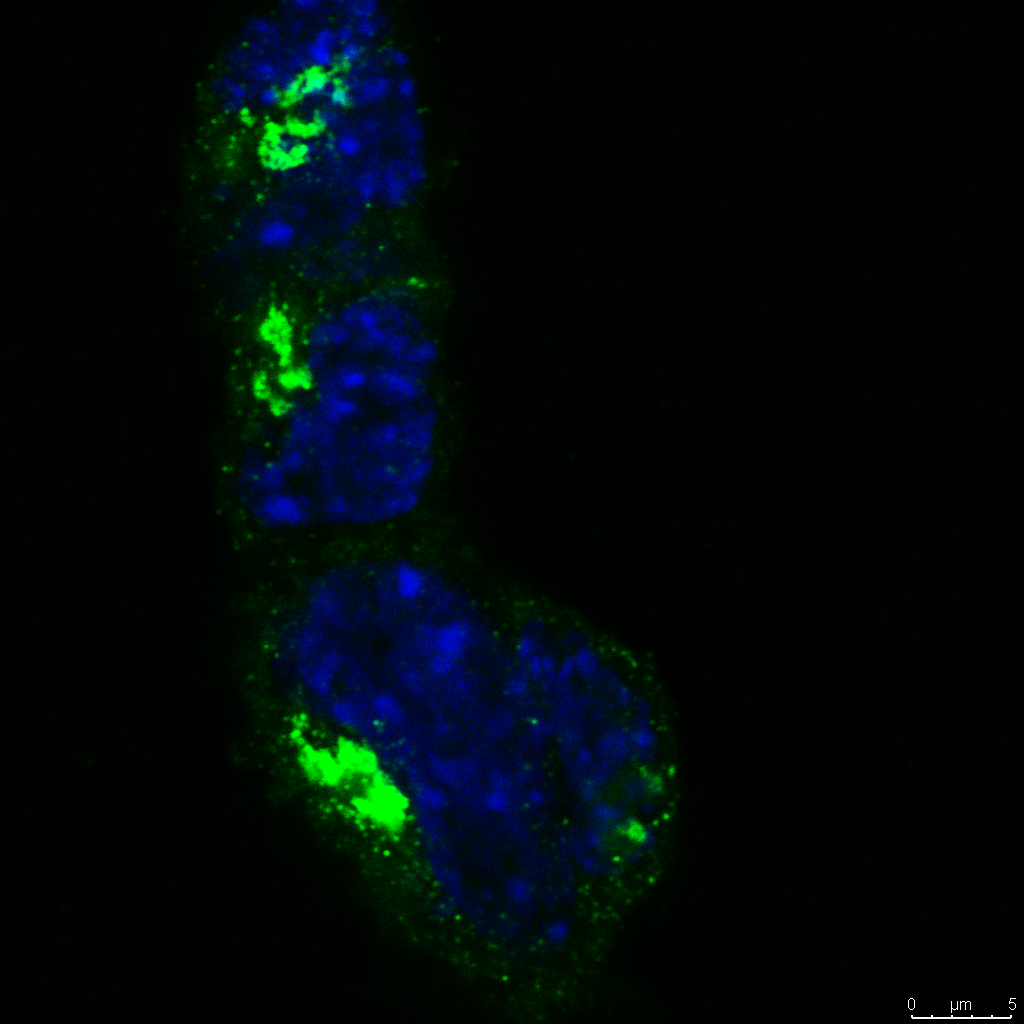

Supplement: Supplementary file 28 — Source data Fig. 2 [file 44318_2025_540_MOESM28_ESM.zip › SD Figure 2/2B/Microscopy 2h 30 min Golgi.tif]

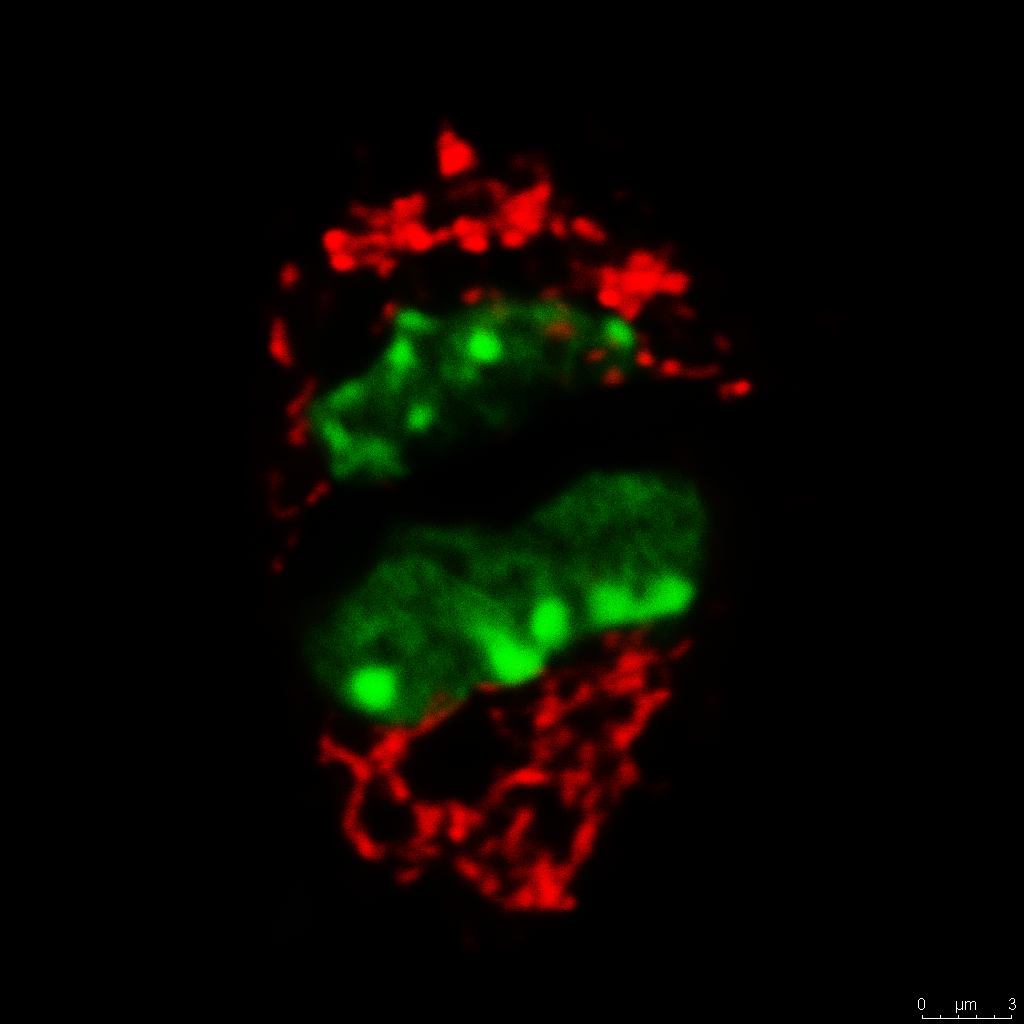

Supplement: Supplementary file 28 — Source data Fig. 2 [file 44318_2025_540_MOESM28_ESM.zip › SD Figure 2/2B/Microscopy 2h 30 min Mitochondria.tif]

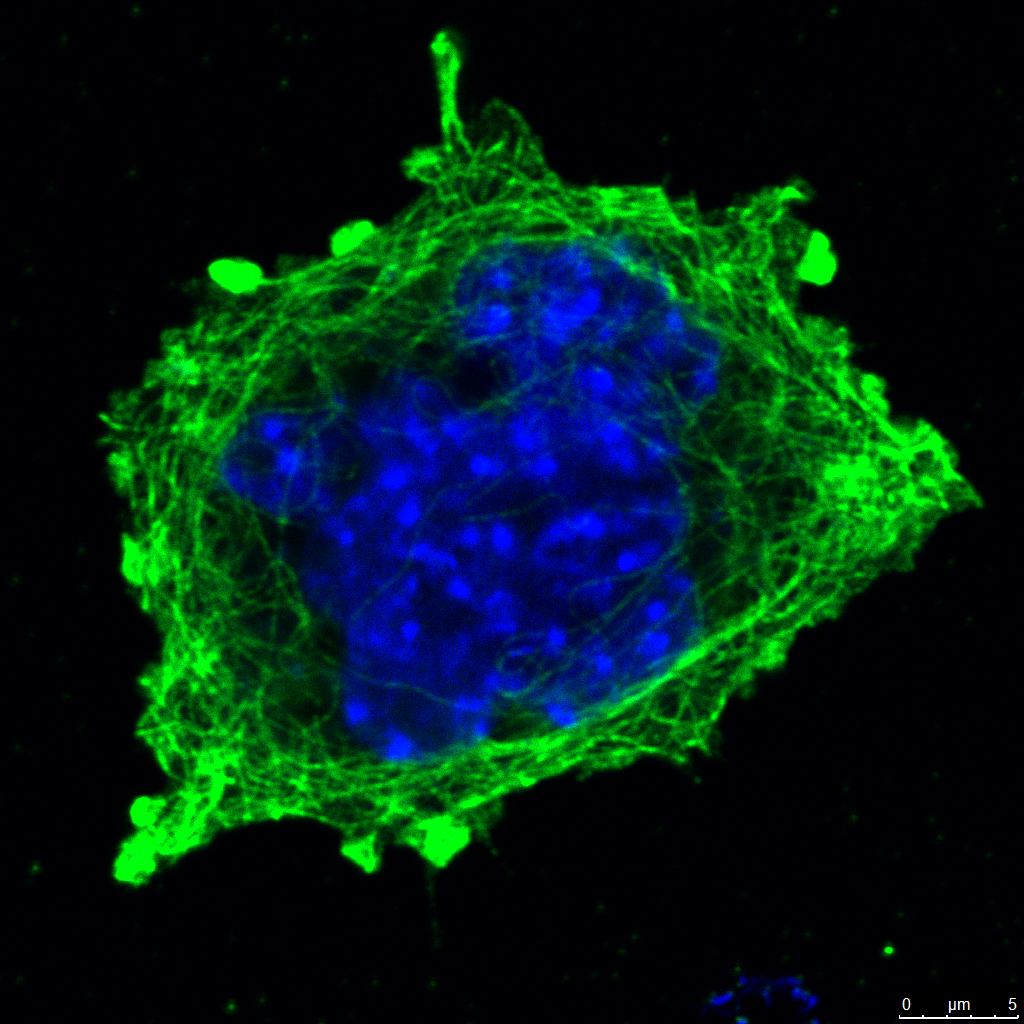

Supplement: Supplementary file 28 — Source data Fig. 2 [file 44318_2025_540_MOESM28_ESM.zip › SD Figure 2/2B/Microscopy 2h 30 Mins Microtubules.tif]

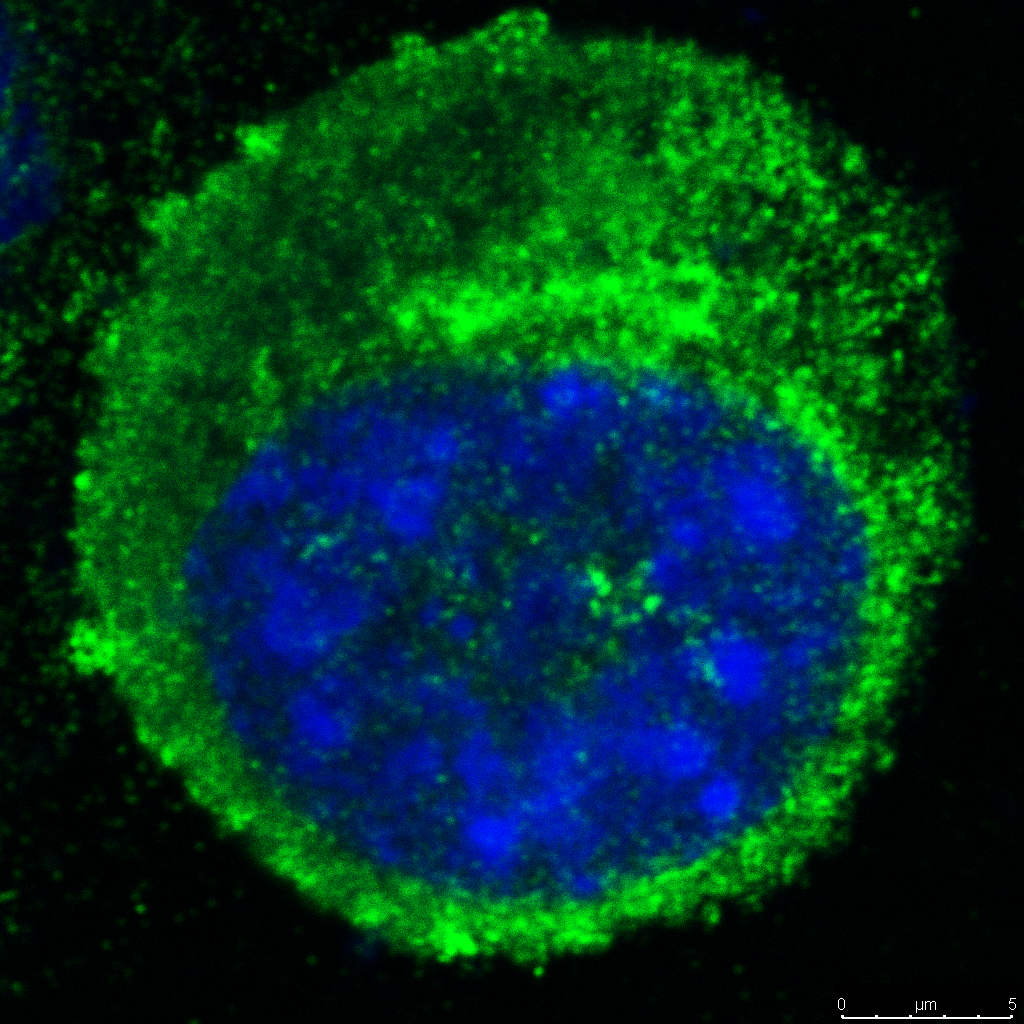

Supplement: Supplementary file 28 — Source data Fig. 2 [file 44318_2025_540_MOESM28_ESM.zip › SD Figure 2/2B/Microscopy 30 min Golgi.tif]

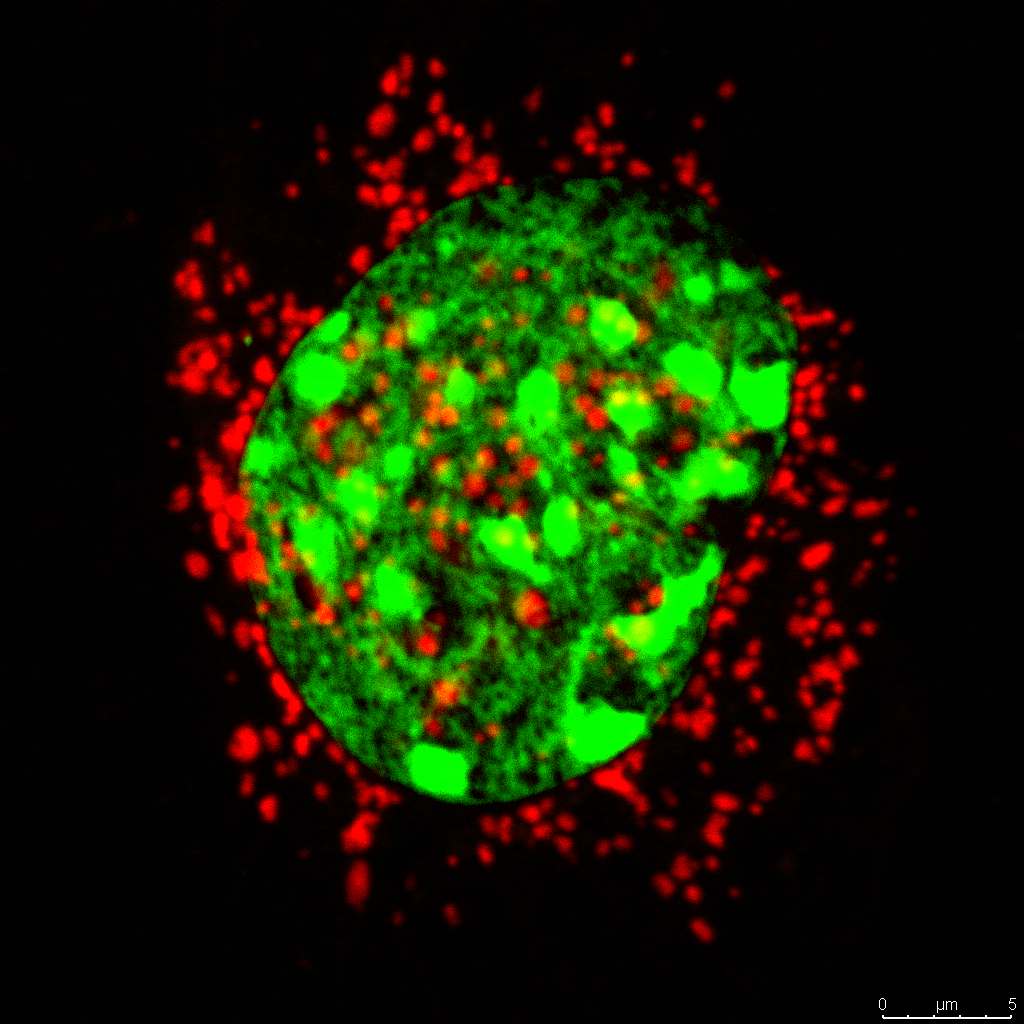

Supplement: Supplementary file 28 — Source data Fig. 2 [file 44318_2025_540_MOESM28_ESM.zip › SD Figure 2/2B/Microscopy 30 min Mitochondria.tif]

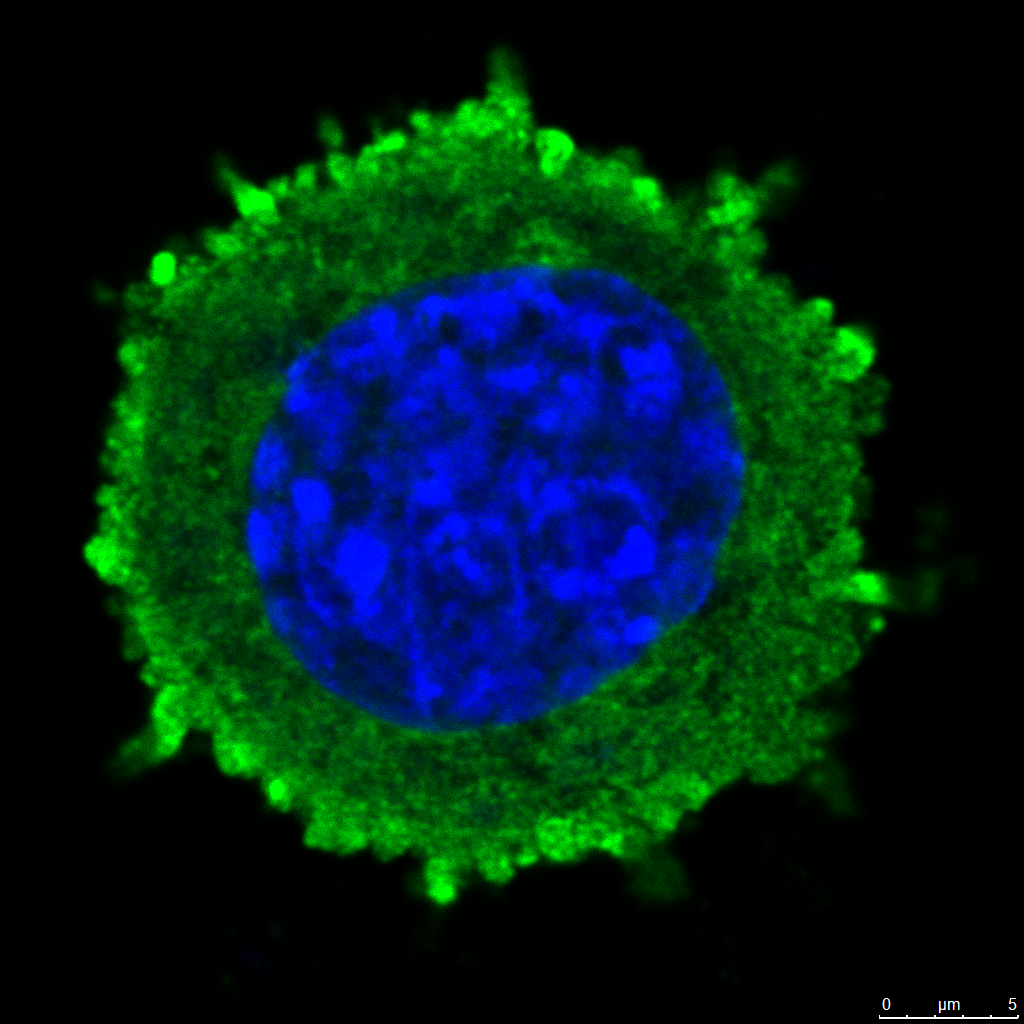

Supplement: Supplementary file 28 — Source data Fig. 2 [file 44318_2025_540_MOESM28_ESM.zip › SD Figure 2/2B/Microscopy 30 Mins Microtubules.tif]

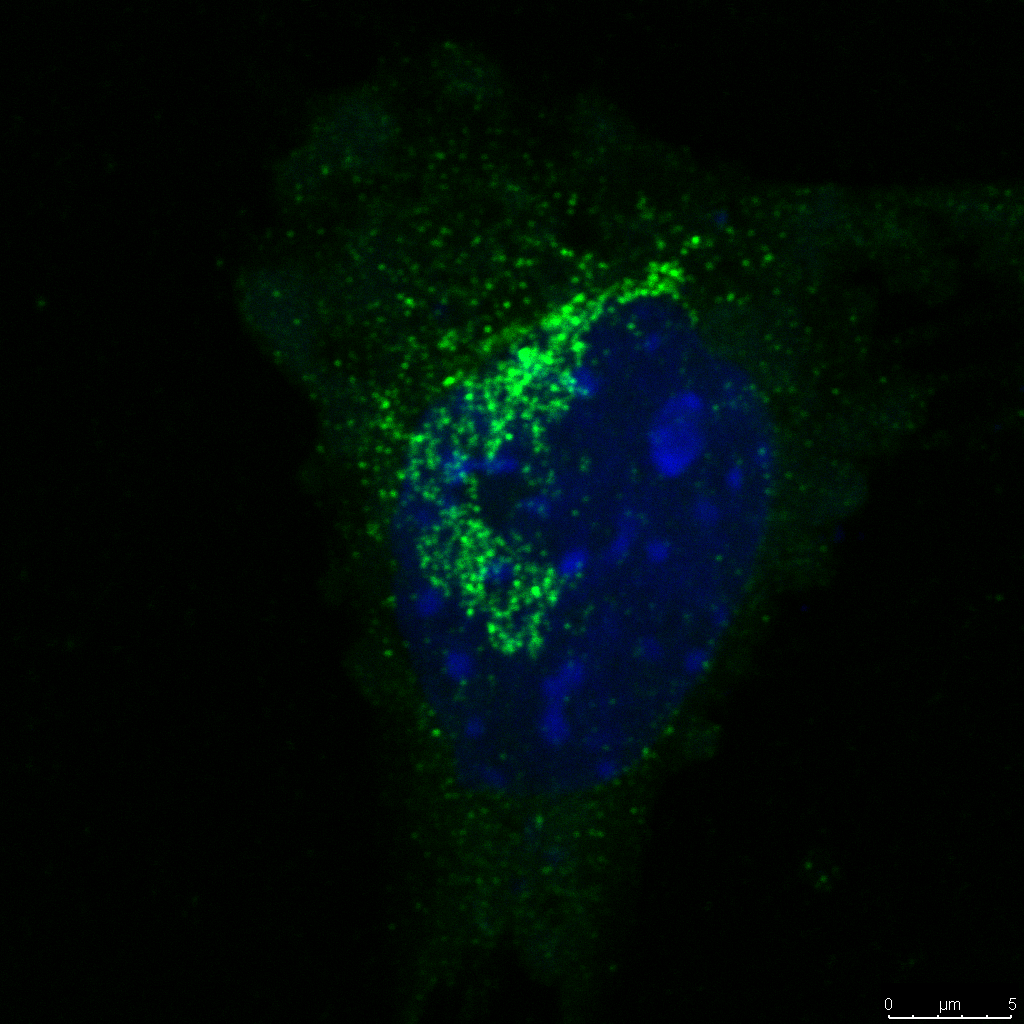

Supplement: Supplementary file 28 — Source data Fig. 2 [file 44318_2025_540_MOESM28_ESM.zip › SD Figure 2/2B/Microscopy 5 min Golgi.tif]

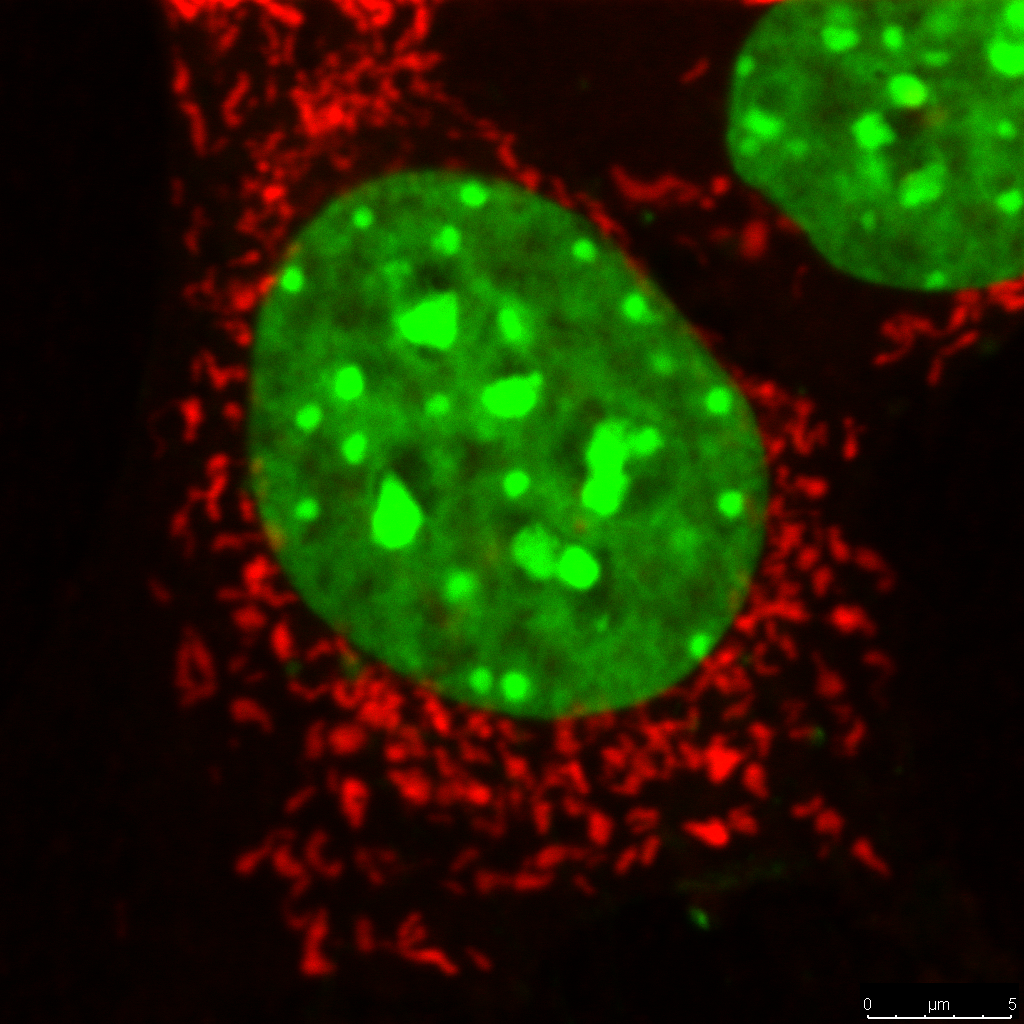

Supplement: Supplementary file 28 — Source data Fig. 2 [file 44318_2025_540_MOESM28_ESM.zip › SD Figure 2/2B/Microscopy 5 min Mitochondria.tif]

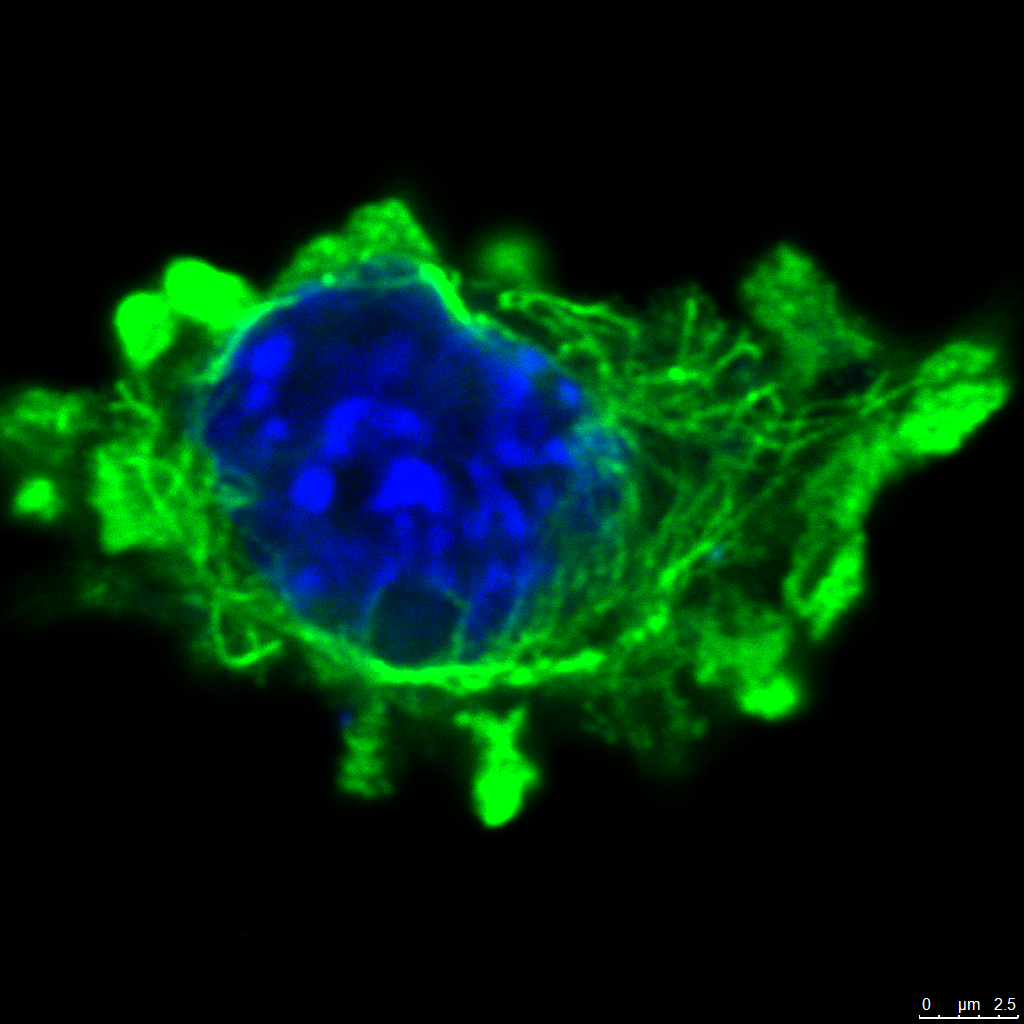

Supplement: Supplementary file 28 — Source data Fig. 2 [file 44318_2025_540_MOESM28_ESM.zip › SD Figure 2/2B/Microscopy 5 Mins Microtubules.tif]

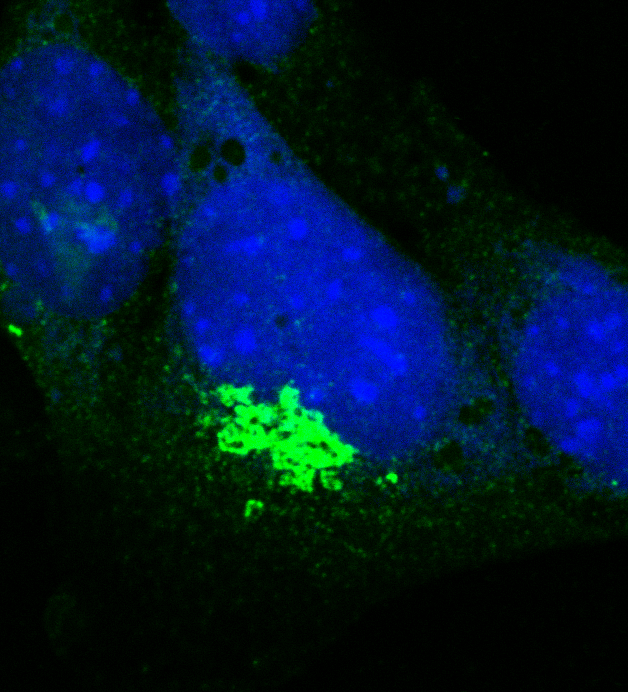

Supplement: Supplementary file 28 — Source data Fig. 2 [file 44318_2025_540_MOESM28_ESM.zip › SD Figure 2/2B/Microscopy Control Golgi.tif]

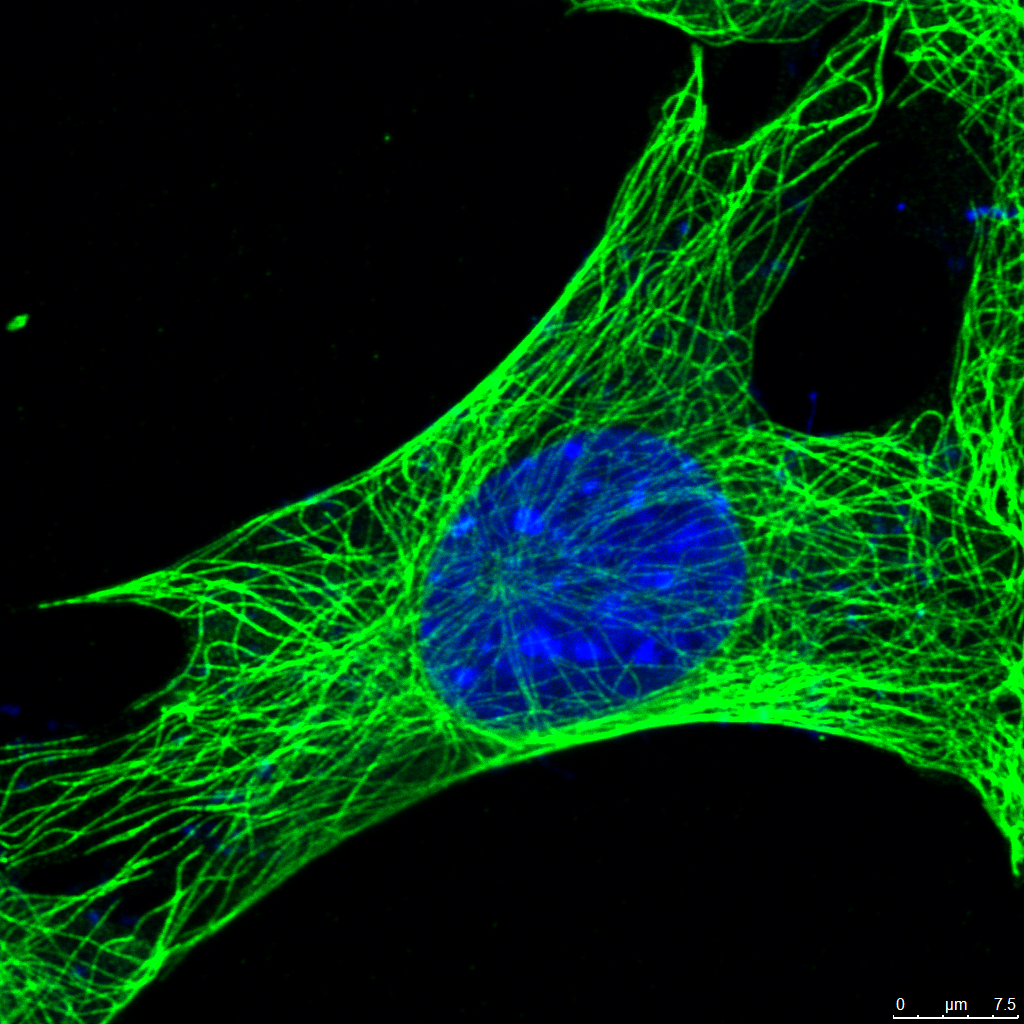

Supplement: Supplementary file 28 — Source data Fig. 2 [file 44318_2025_540_MOESM28_ESM.zip › SD Figure 2/2B/Microscopy Control Microtubules.tif]

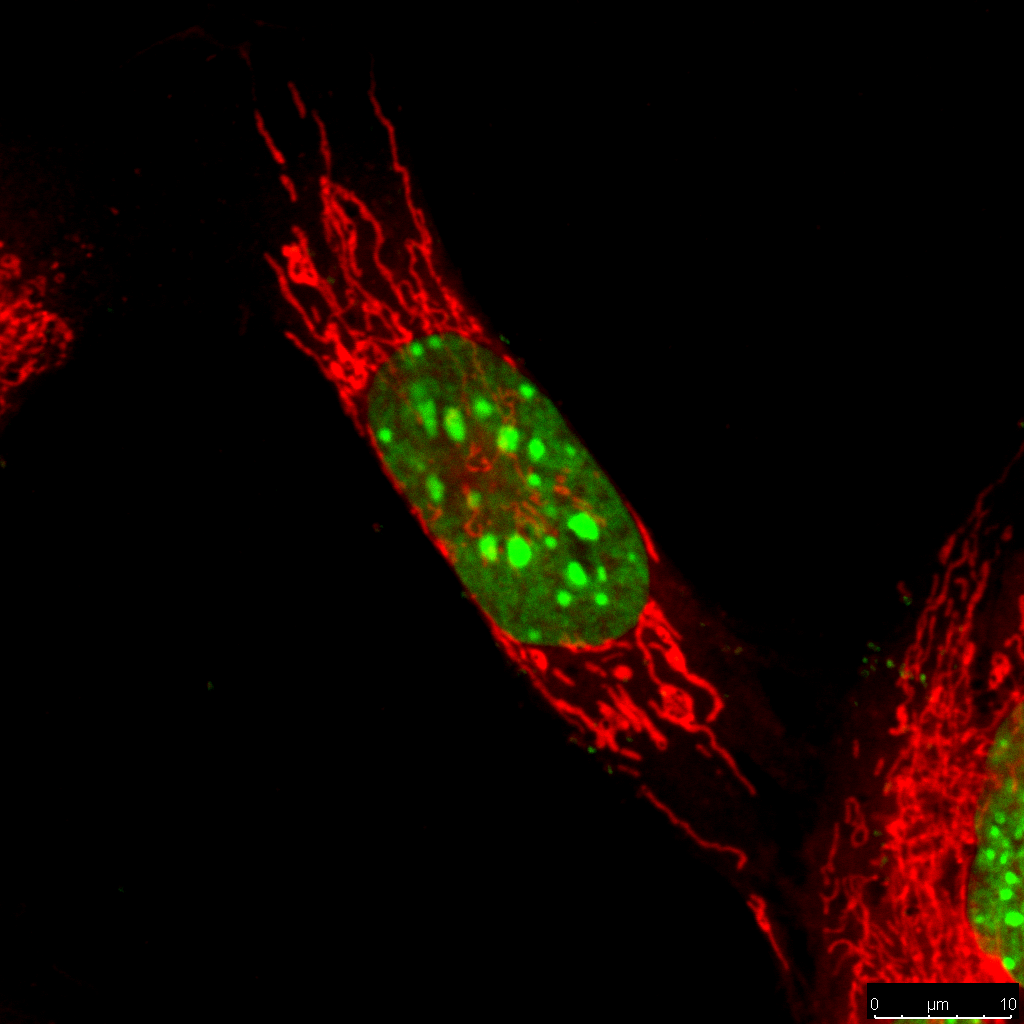

Supplement: Supplementary file 28 — Source data Fig. 2 [file 44318_2025_540_MOESM28_ESM.zip › SD Figure 2/2B/Microscopy Control Mitochondria.tif]

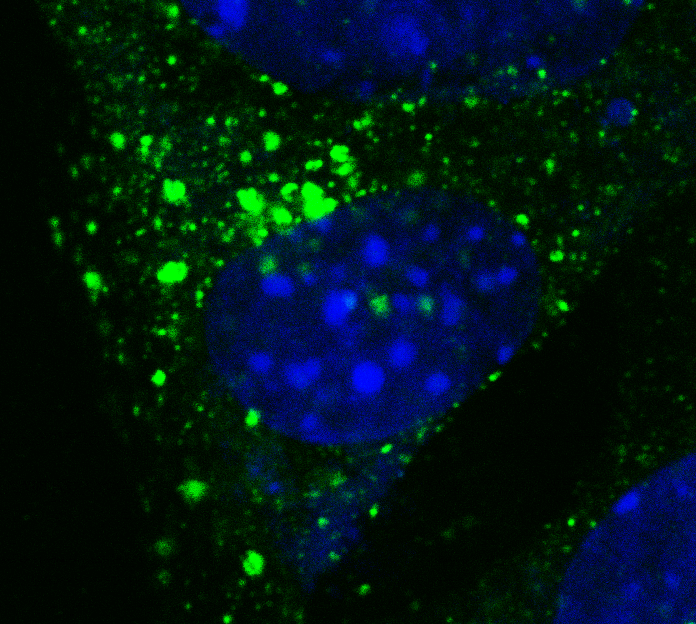

Supplement: Supplementary file 28 — Source data Fig. 2 [file 44318_2025_540_MOESM28_ESM.zip › SD Figure 2/2C/Microscopy 16h.tif]

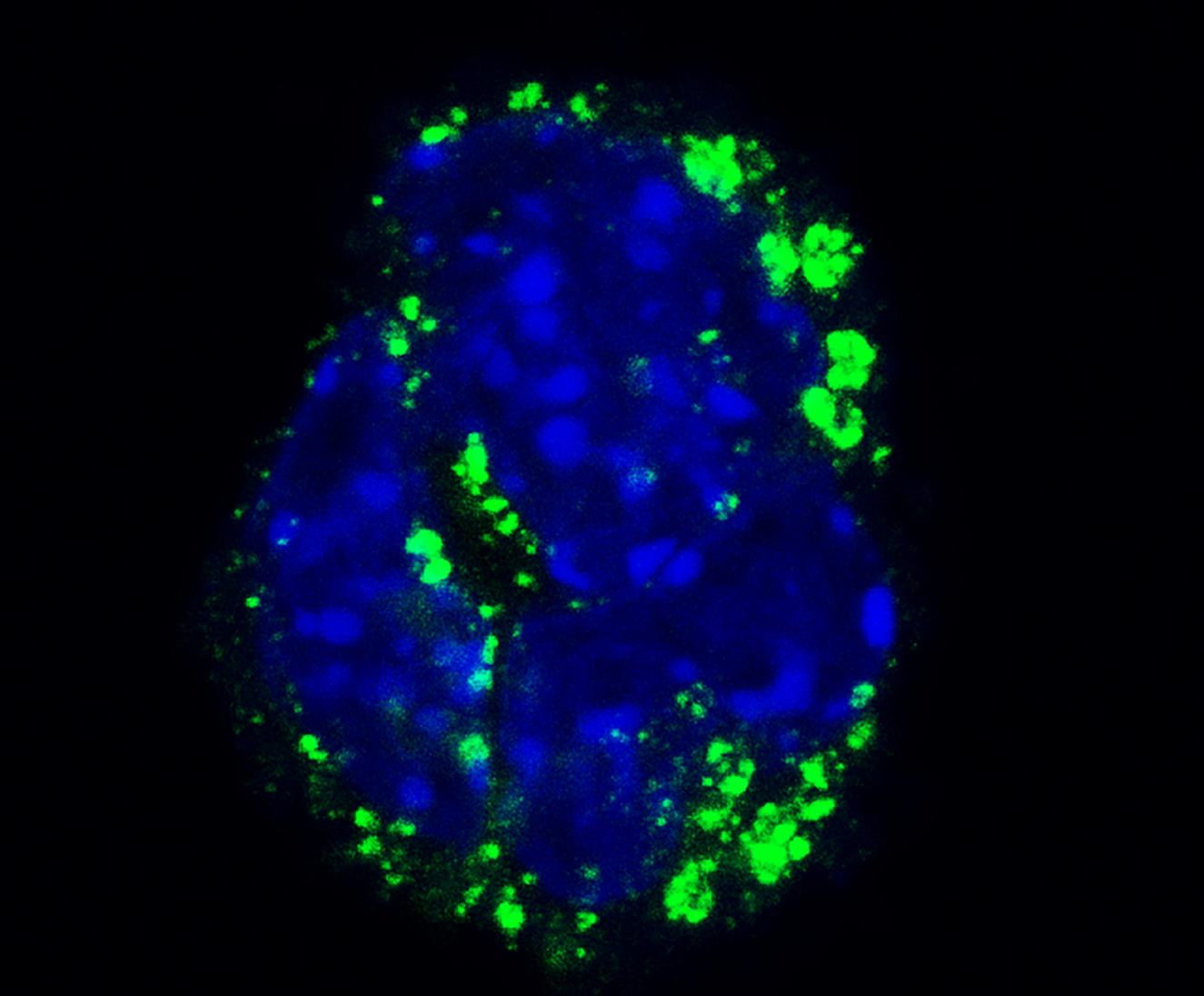

Supplement: Supplementary file 28 — Source data Fig. 2 [file 44318_2025_540_MOESM28_ESM.zip › SD Figure 2/2C/Microscopy 2h 30 min.tif]

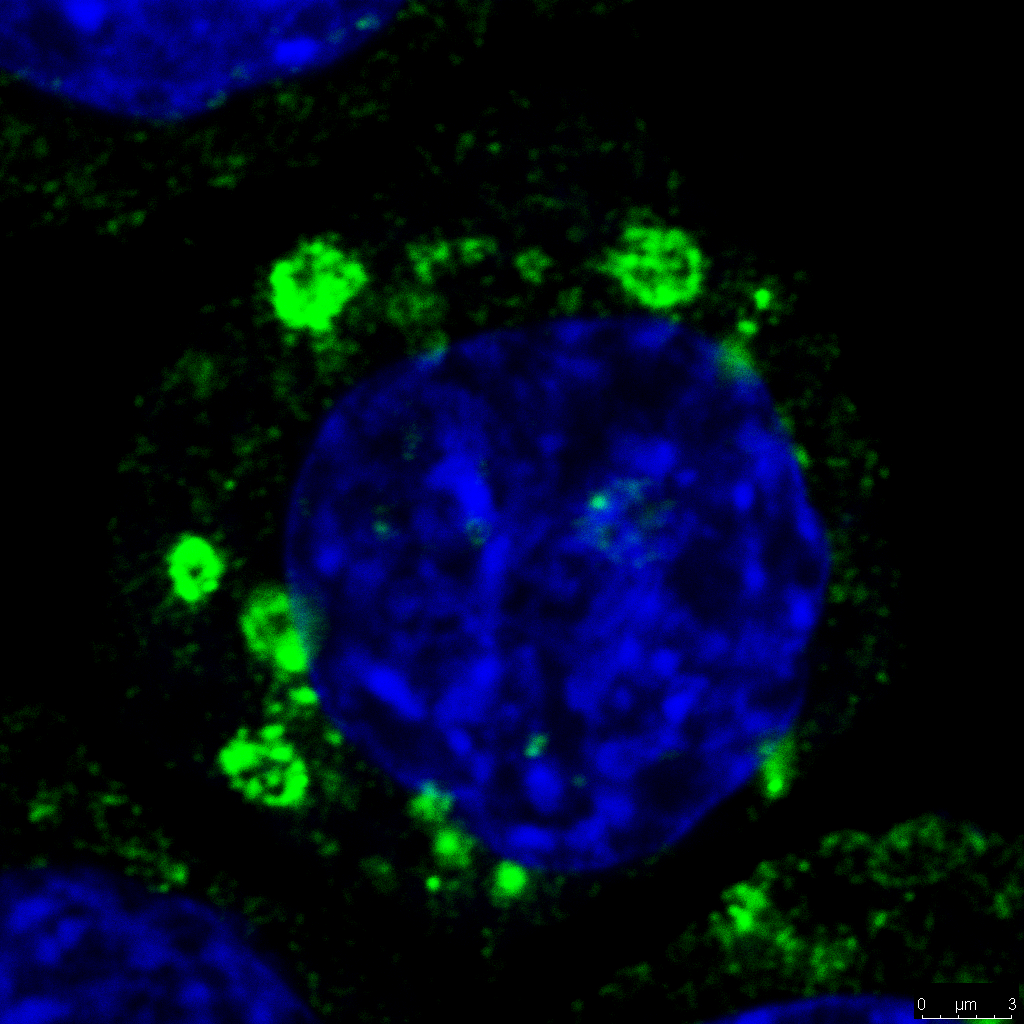

Supplement: Supplementary file 28 — Source data Fig. 2 [file 44318_2025_540_MOESM28_ESM.zip › SD Figure 2/2C/Microscopy 30 min.tif]

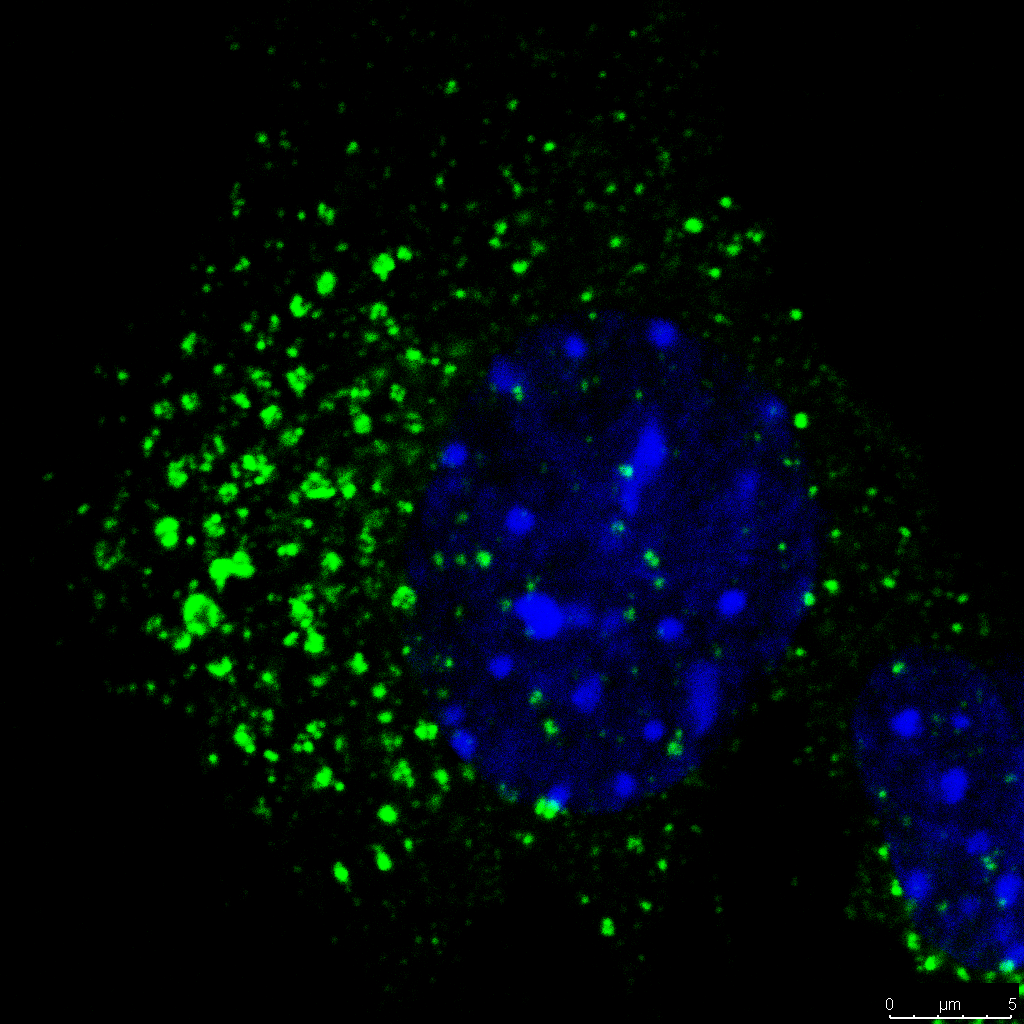

Supplement: Supplementary file 28 — Source data Fig. 2 [file 44318_2025_540_MOESM28_ESM.zip › SD Figure 2/2C/Microscopy 5 min.tif]

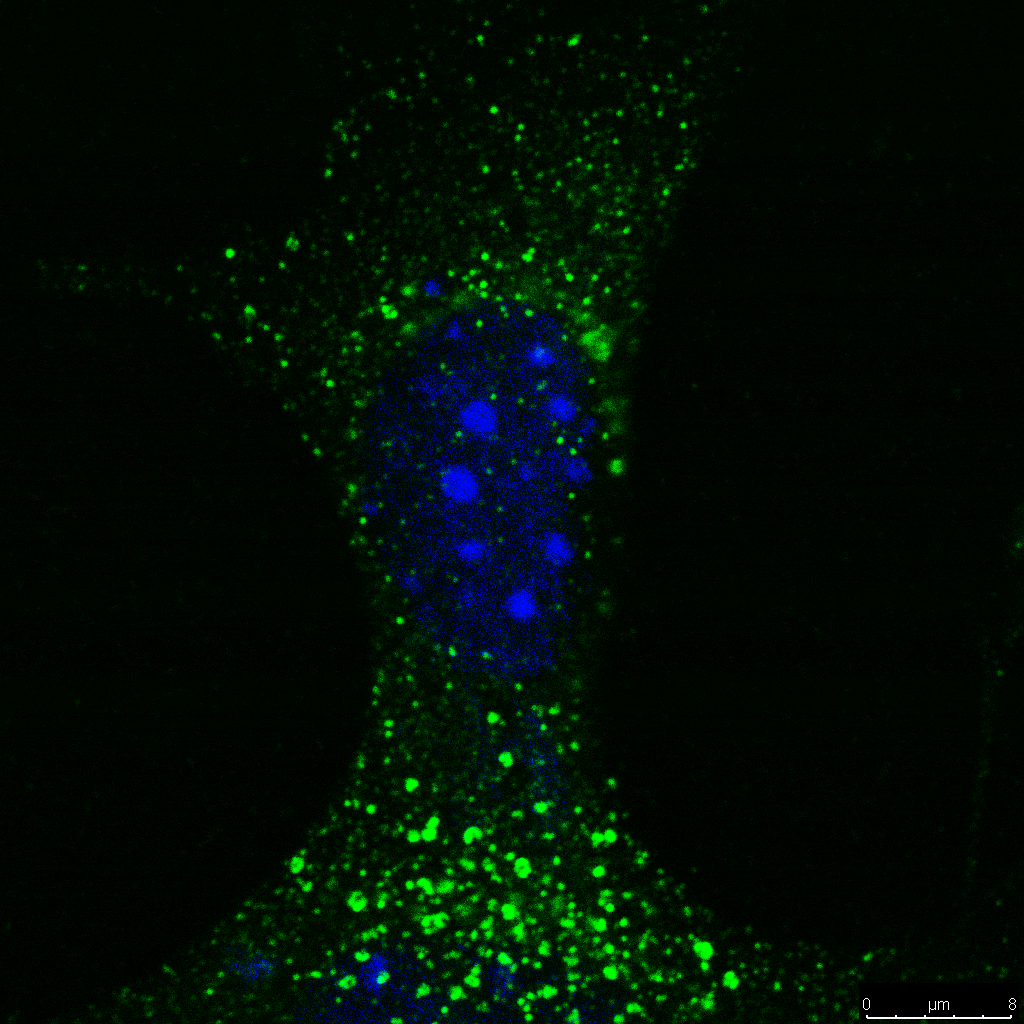

Supplement: Supplementary file 28 — Source data Fig. 2 [file 44318_2025_540_MOESM28_ESM.zip › SD Figure 2/2C/Microscopy Control.tif]

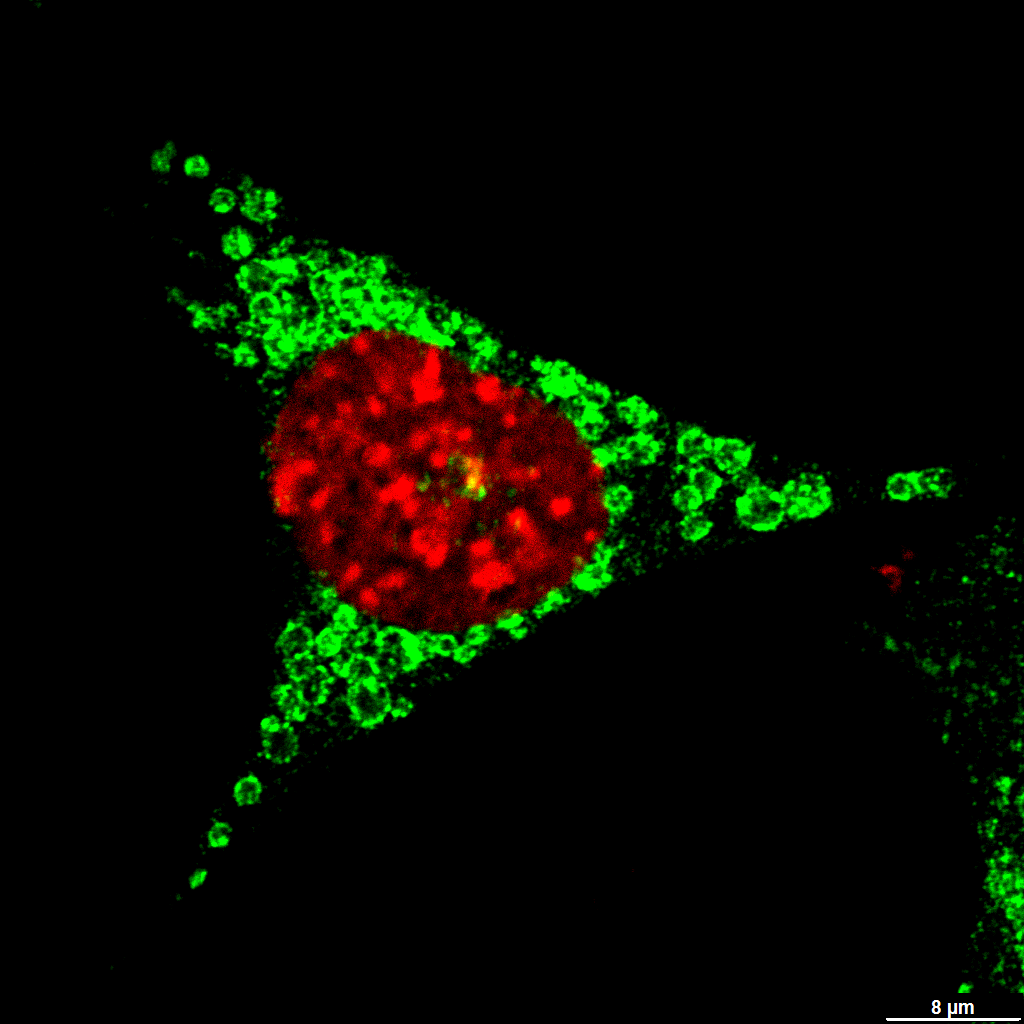

Supplement: Supplementary file 28 — Source data Fig. 2 [file 44318_2025_540_MOESM28_ESM.zip › SD Figure 2/2D/Microscopy 16h Late Endosome.tif]

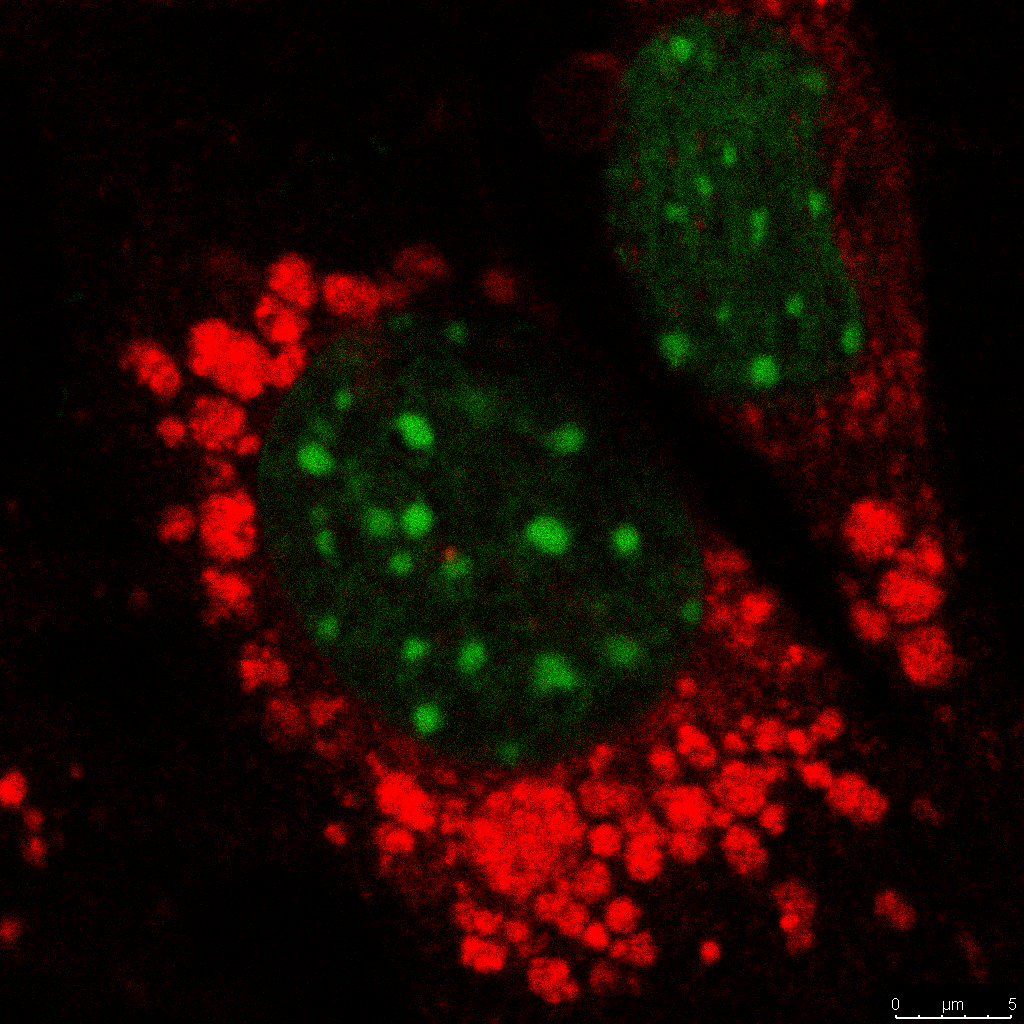

Supplement: Supplementary file 28 — Source data Fig. 2 [file 44318_2025_540_MOESM28_ESM.zip › SD Figure 2/2D/Microscopy 16h Lysosome.tif]

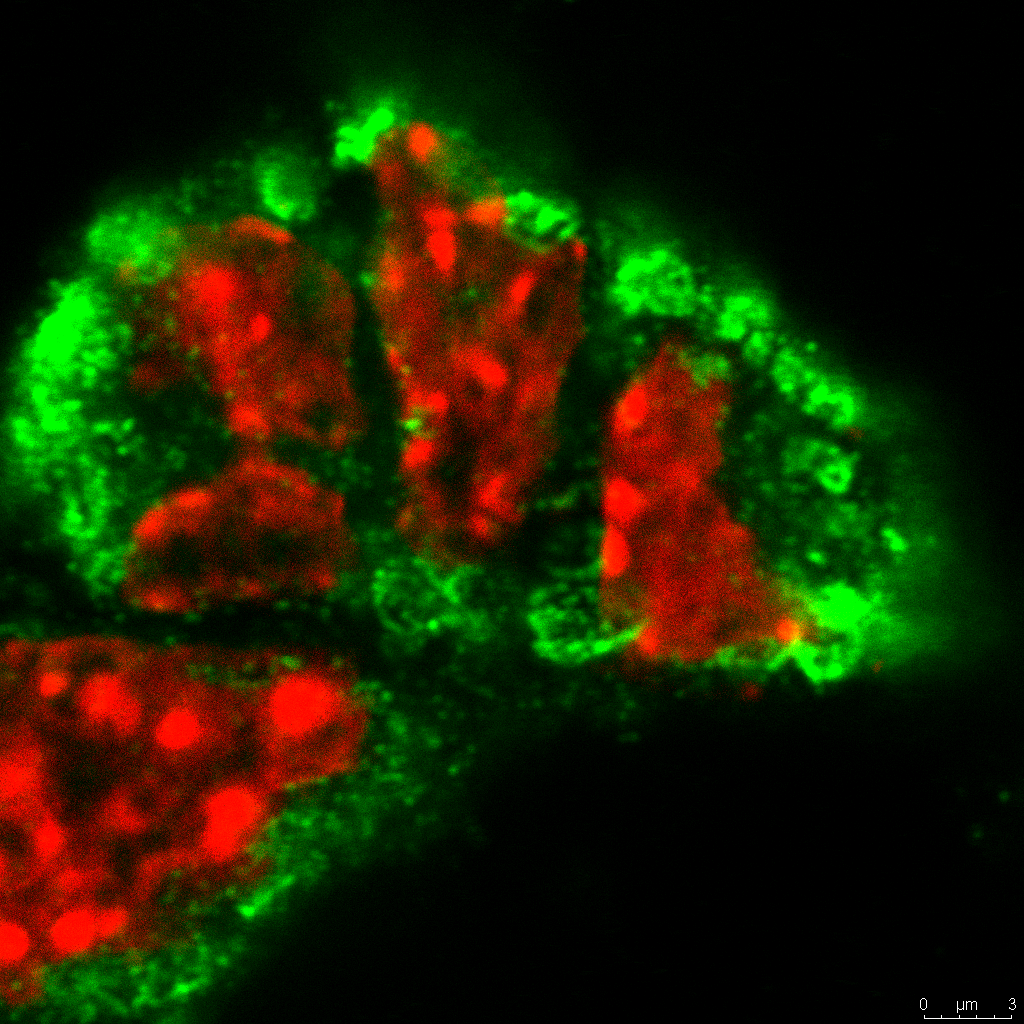

Supplement: Supplementary file 28 — Source data Fig. 2 [file 44318_2025_540_MOESM28_ESM.zip › SD Figure 2/2D/Microscopy 2h 30 min Late Endosome.tif]

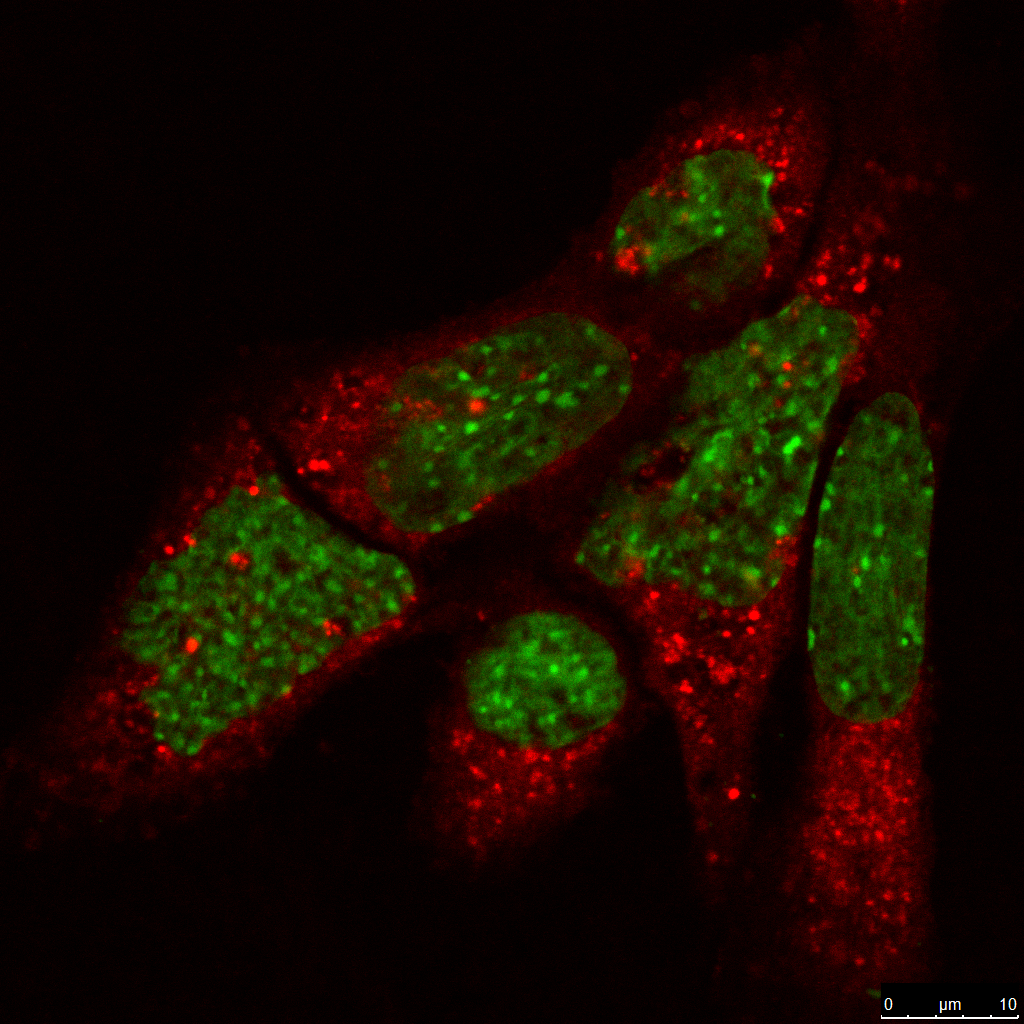

Supplement: Supplementary file 28 — Source data Fig. 2 [file 44318_2025_540_MOESM28_ESM.zip › SD Figure 2/2D/Microscopy 2h 30 min Lysosome.tif]

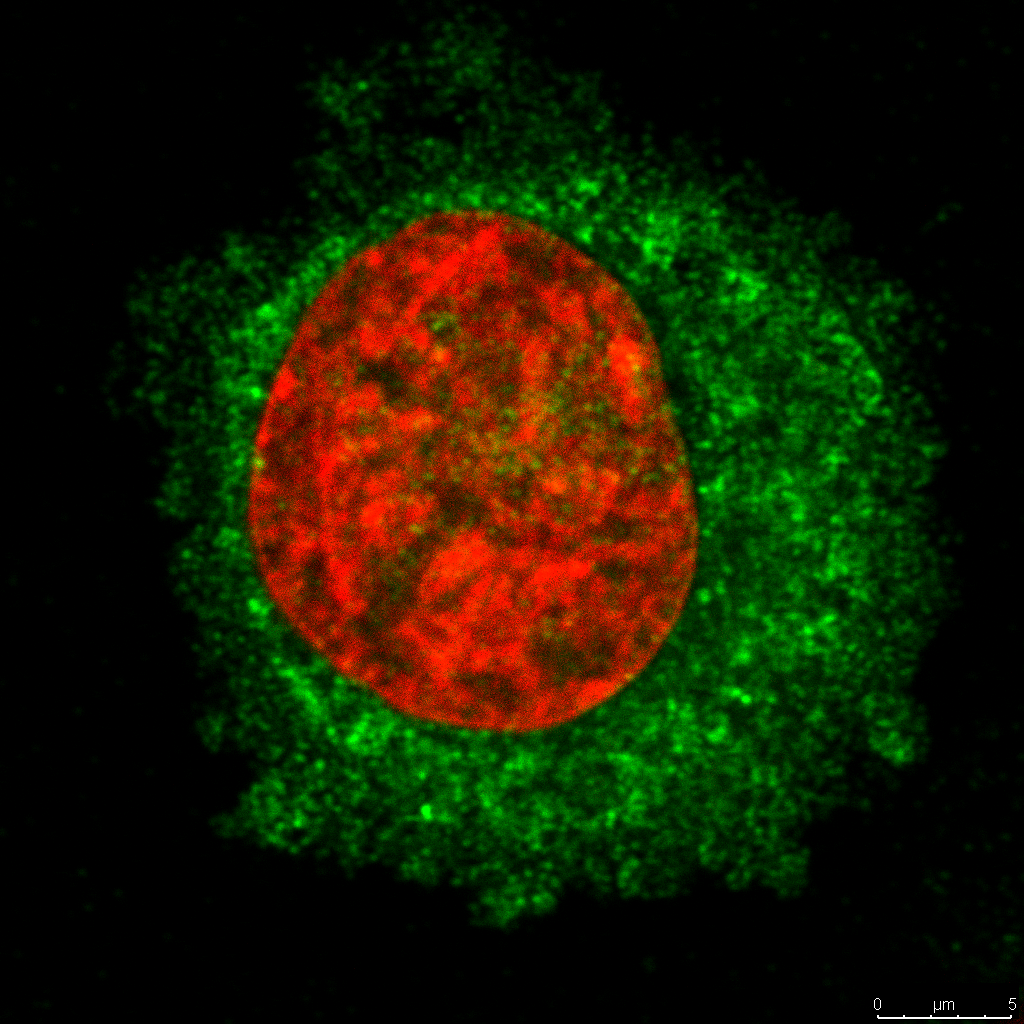

Supplement: Supplementary file 28 — Source data Fig. 2 [file 44318_2025_540_MOESM28_ESM.zip › SD Figure 2/2D/Microscopy 30 min Late Endosome.tif]

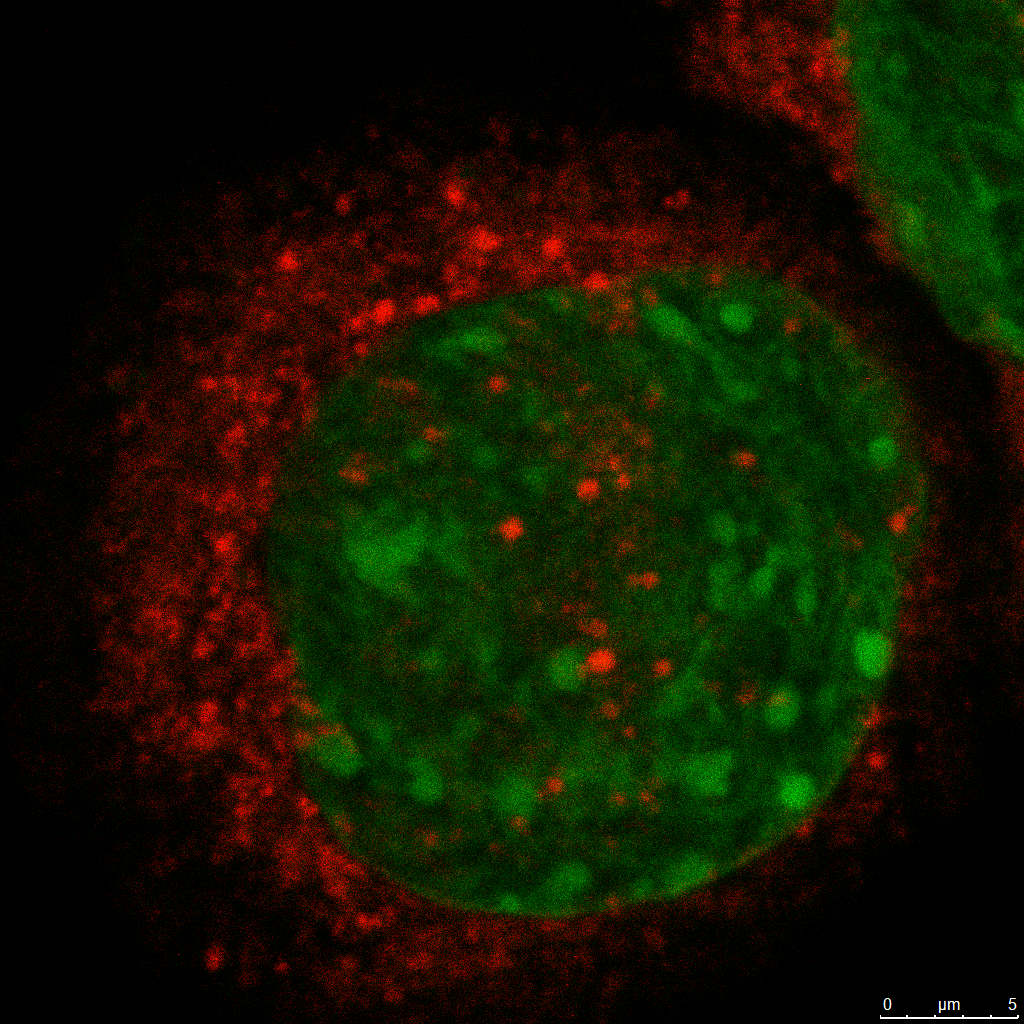

Supplement: Supplementary file 28 — Source data Fig. 2 [file 44318_2025_540_MOESM28_ESM.zip › SD Figure 2/2D/Microscopy 30 min Lysosome.tif]

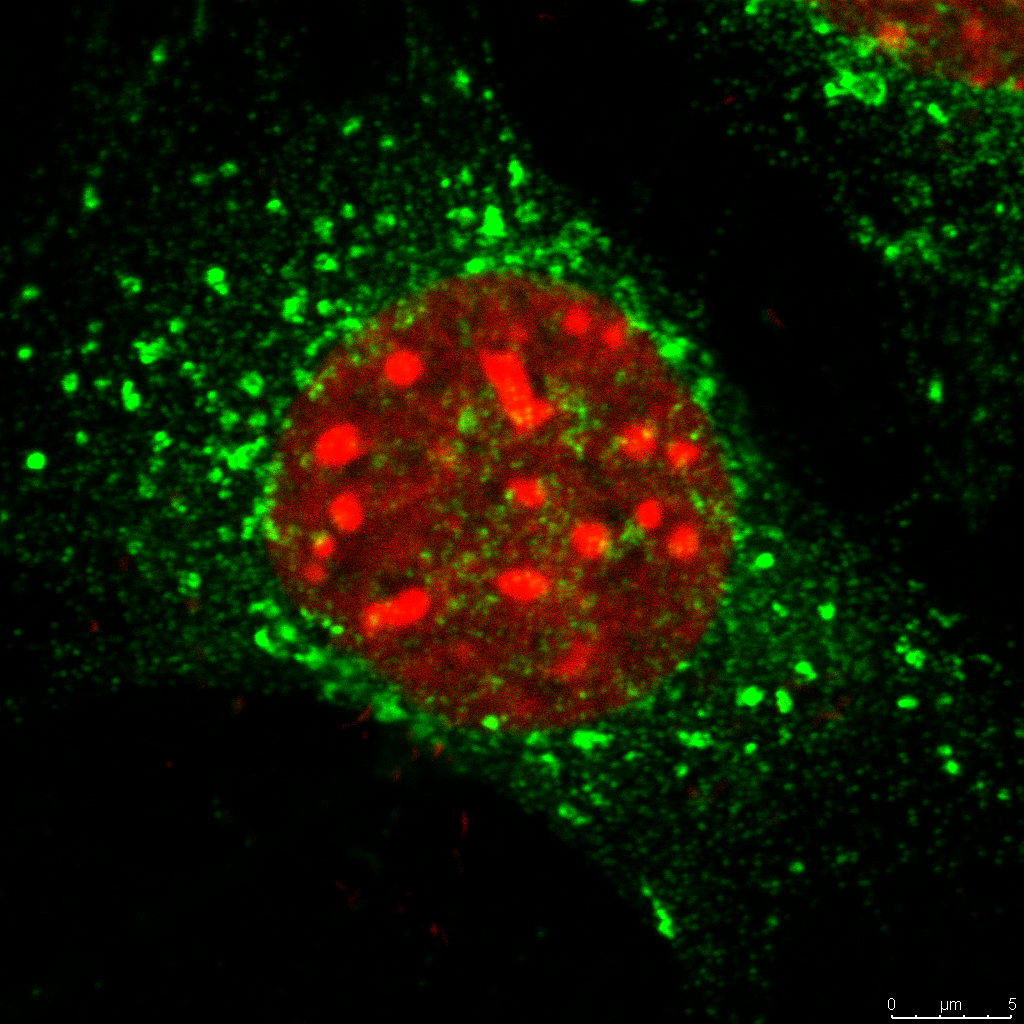

Supplement: Supplementary file 28 — Source data Fig. 2 [file 44318_2025_540_MOESM28_ESM.zip › SD Figure 2/2D/Microscopy 5 min Late Endosome.tif]

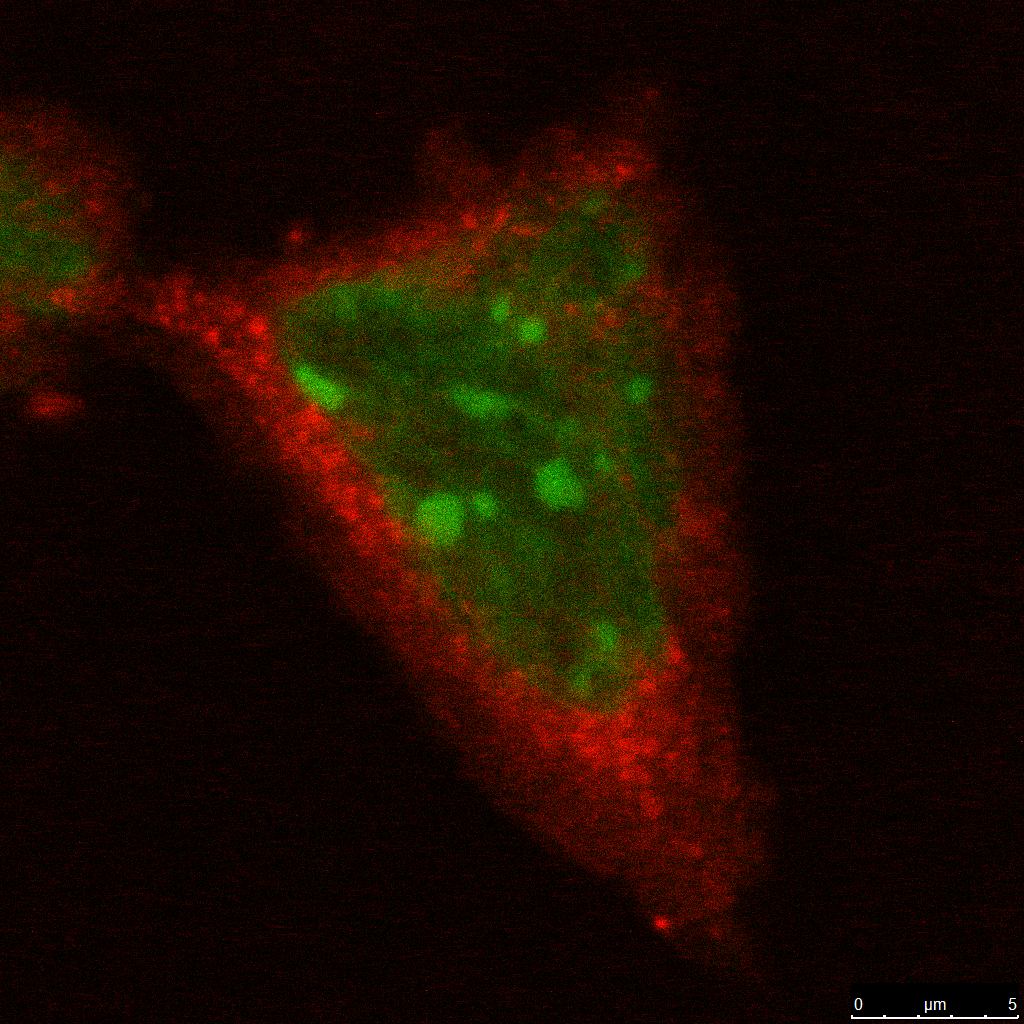

Supplement: Supplementary file 28 — Source data Fig. 2 [file 44318_2025_540_MOESM28_ESM.zip › SD Figure 2/2D/Microscopy 5 min Lysosome.tif]

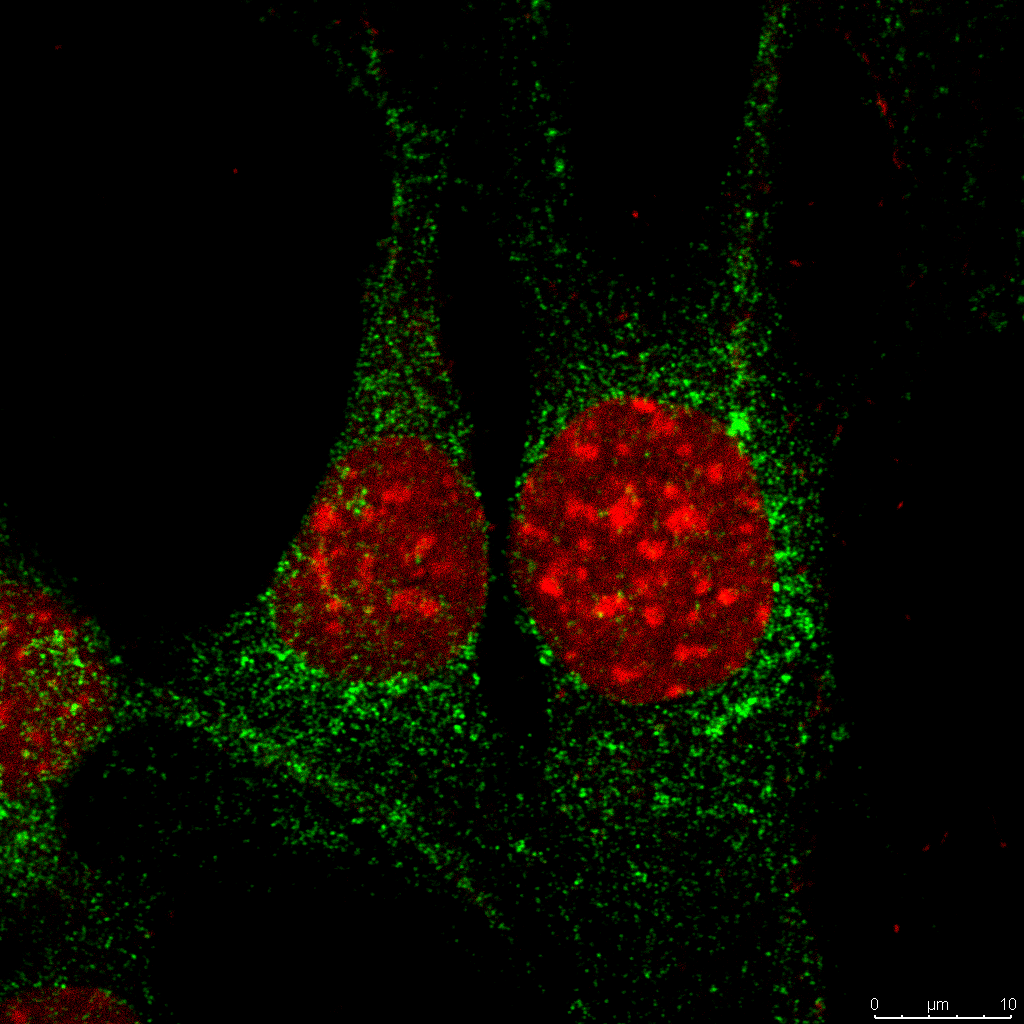

Supplement: Supplementary file 28 — Source data Fig. 2 [file 44318_2025_540_MOESM28_ESM.zip › SD Figure 2/2D/Microscopy Control Late Endosome.tif]

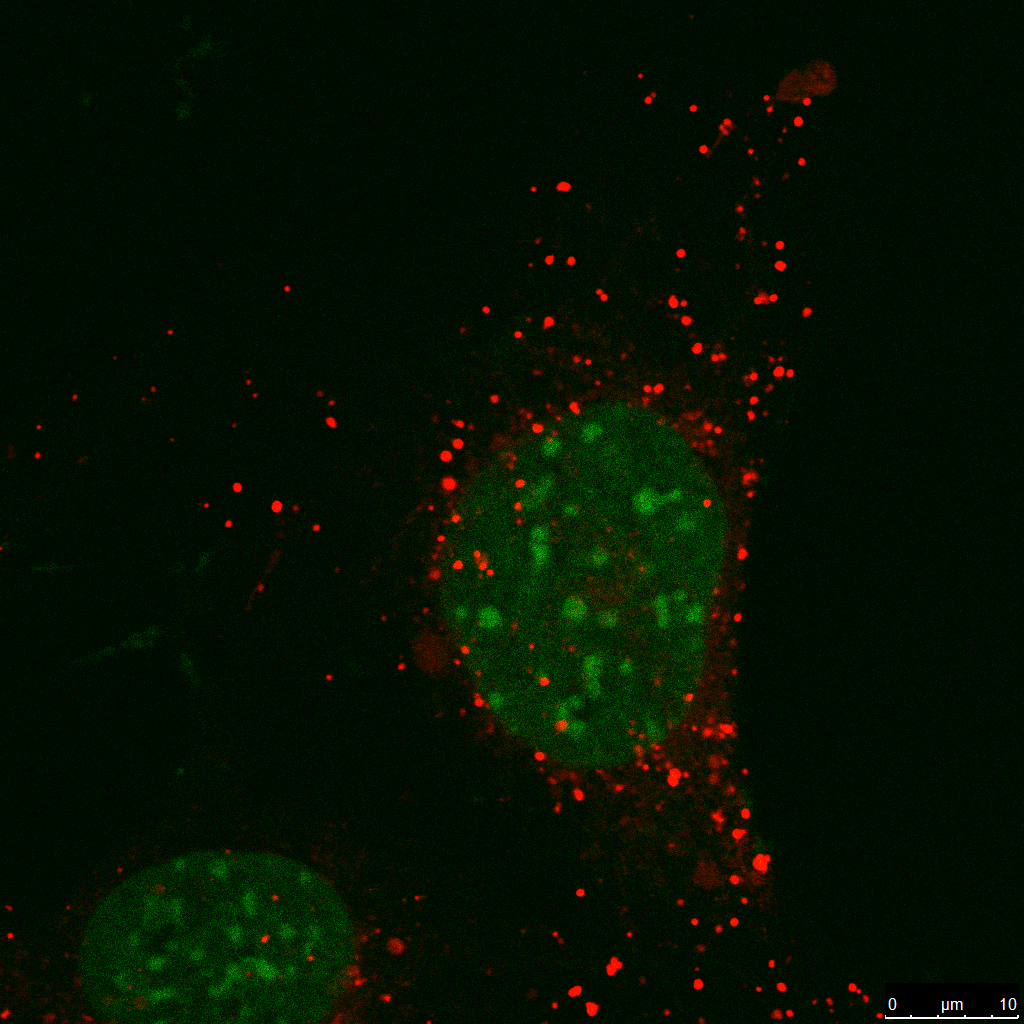

Supplement: Supplementary file 28 — Source data Fig. 2 [file 44318_2025_540_MOESM28_ESM.zip › SD Figure 2/2D/Microscopy Control Lysosome.tif]

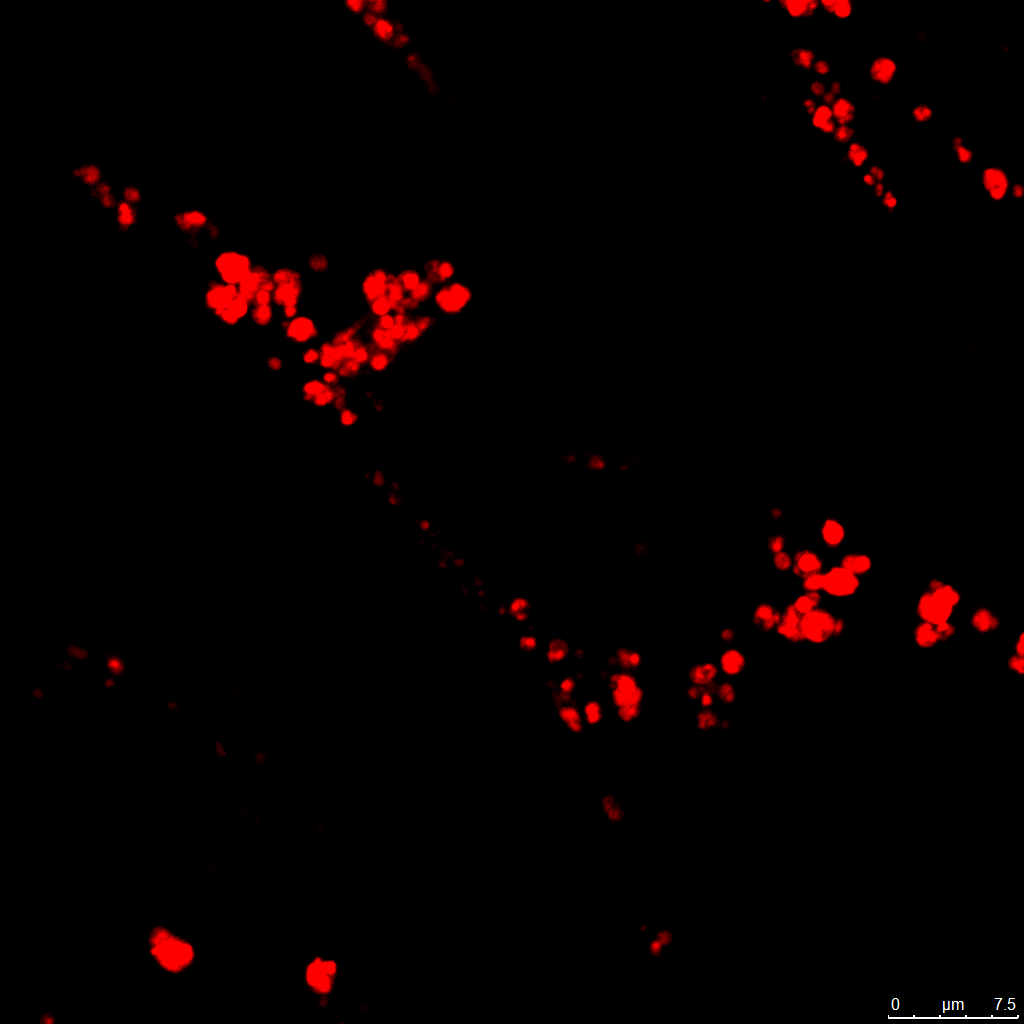

Supplement: Supplementary file 28 — Source data Fig. 2 [file 44318_2025_540_MOESM28_ESM.zip › SD Figure 2/2E/Microscopy MEF_6hr Lysotracker Red.tif]

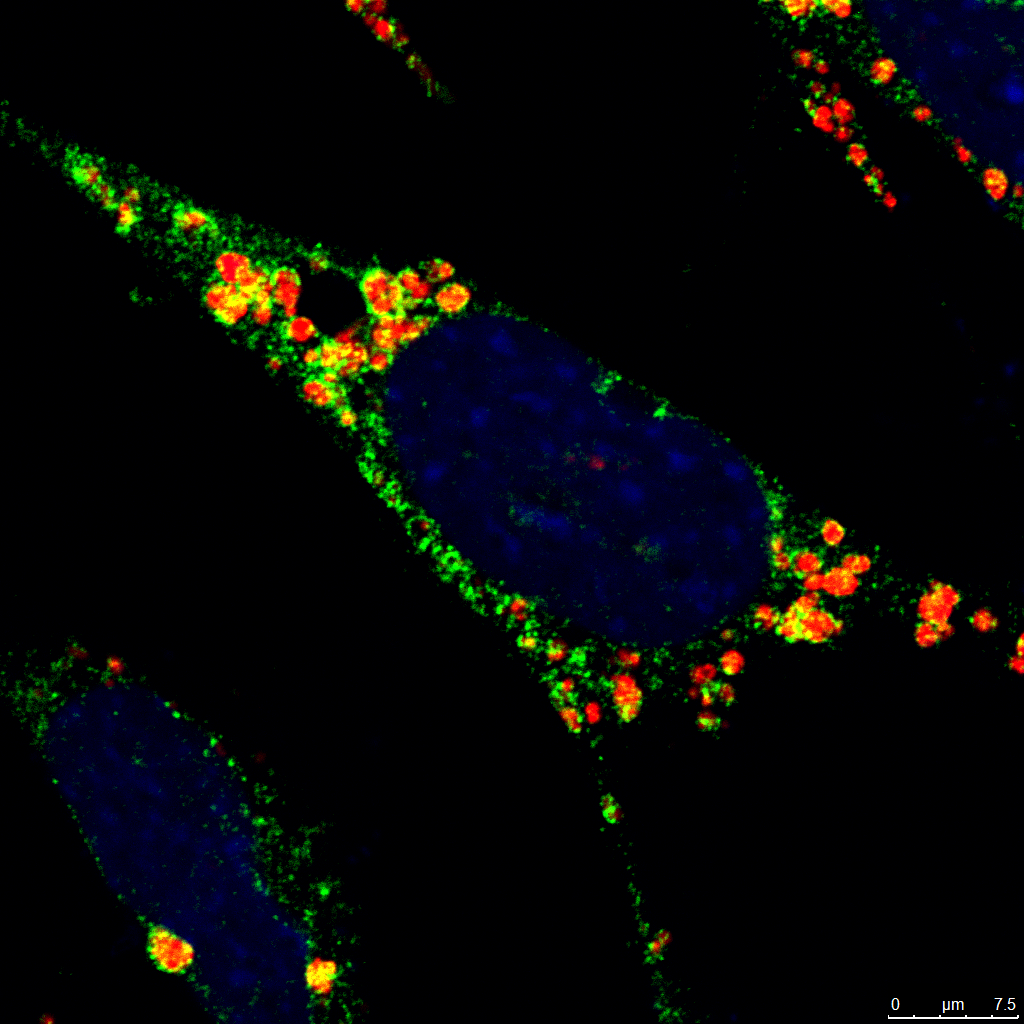

Supplement: Supplementary file 28 — Source data Fig. 2 [file 44318_2025_540_MOESM28_ESM.zip › SD Figure 2/2E/Microscopy MEF_6hr_RAB7 AND Lysotracker Red.tif]

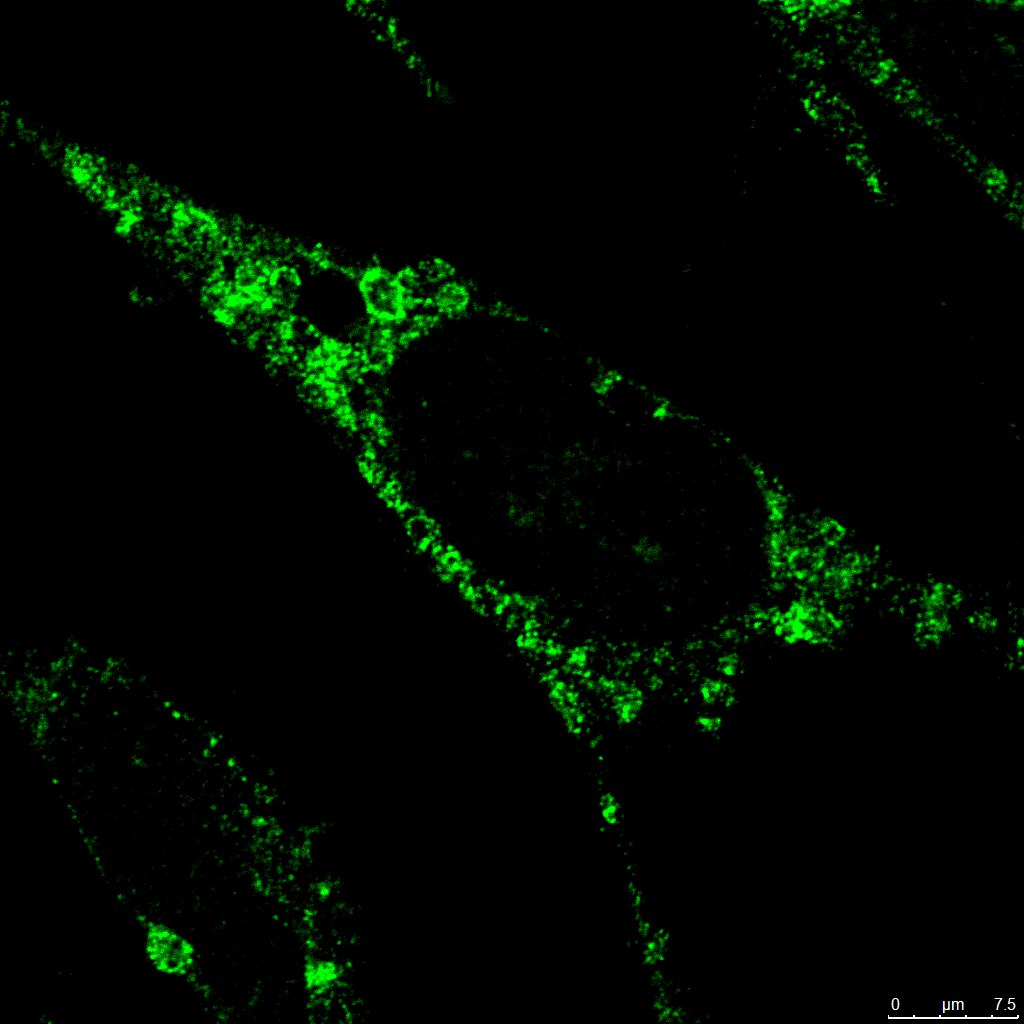

Supplement: Supplementary file 28 — Source data Fig. 2 [file 44318_2025_540_MOESM28_ESM.zip › SD Figure 2/2E/Microscopy MEF_6hr_RAB7.tif]

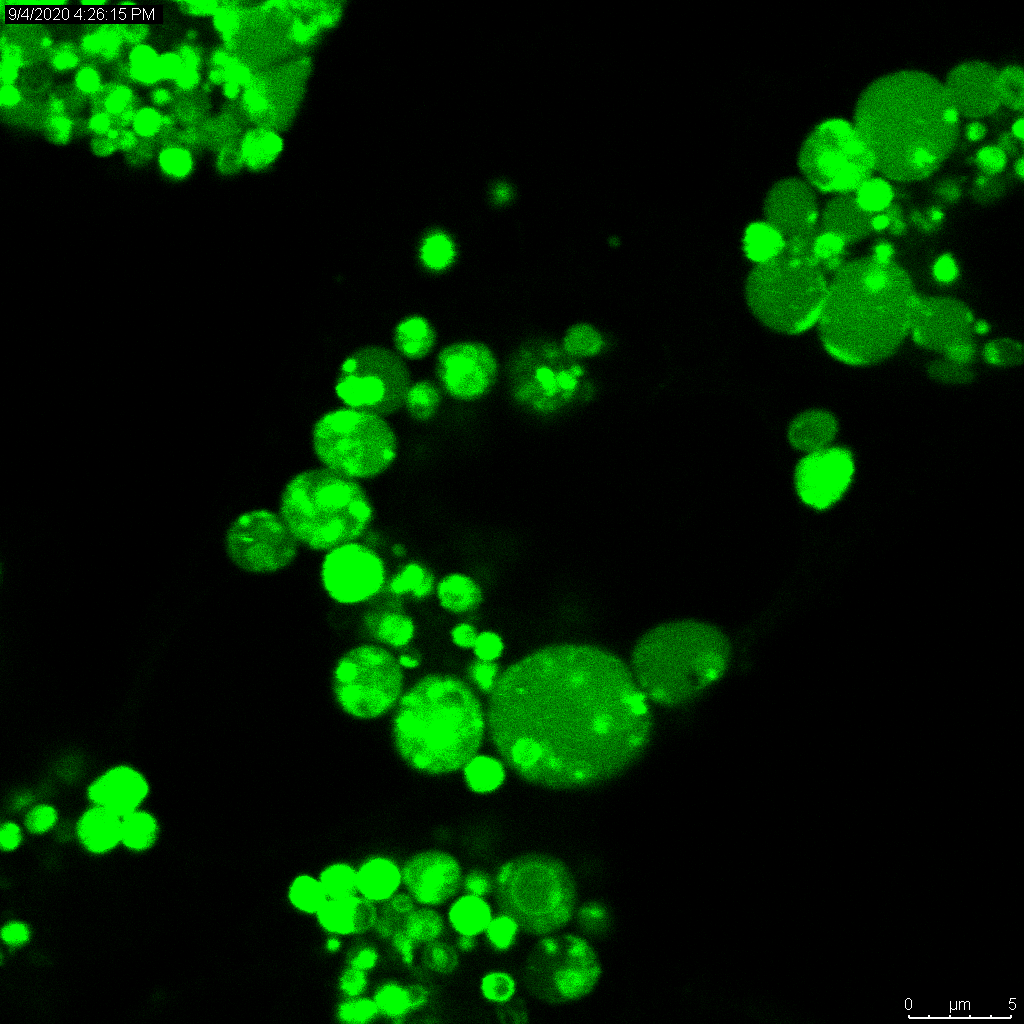

Supplement: Supplementary file 28 — Source data Fig. 2 [file 44318_2025_540_MOESM28_ESM.zip › SD Figure 2/2F/Microscopy MEF_LLOMe Lysotracker Green_6 hours Recovery.tif]

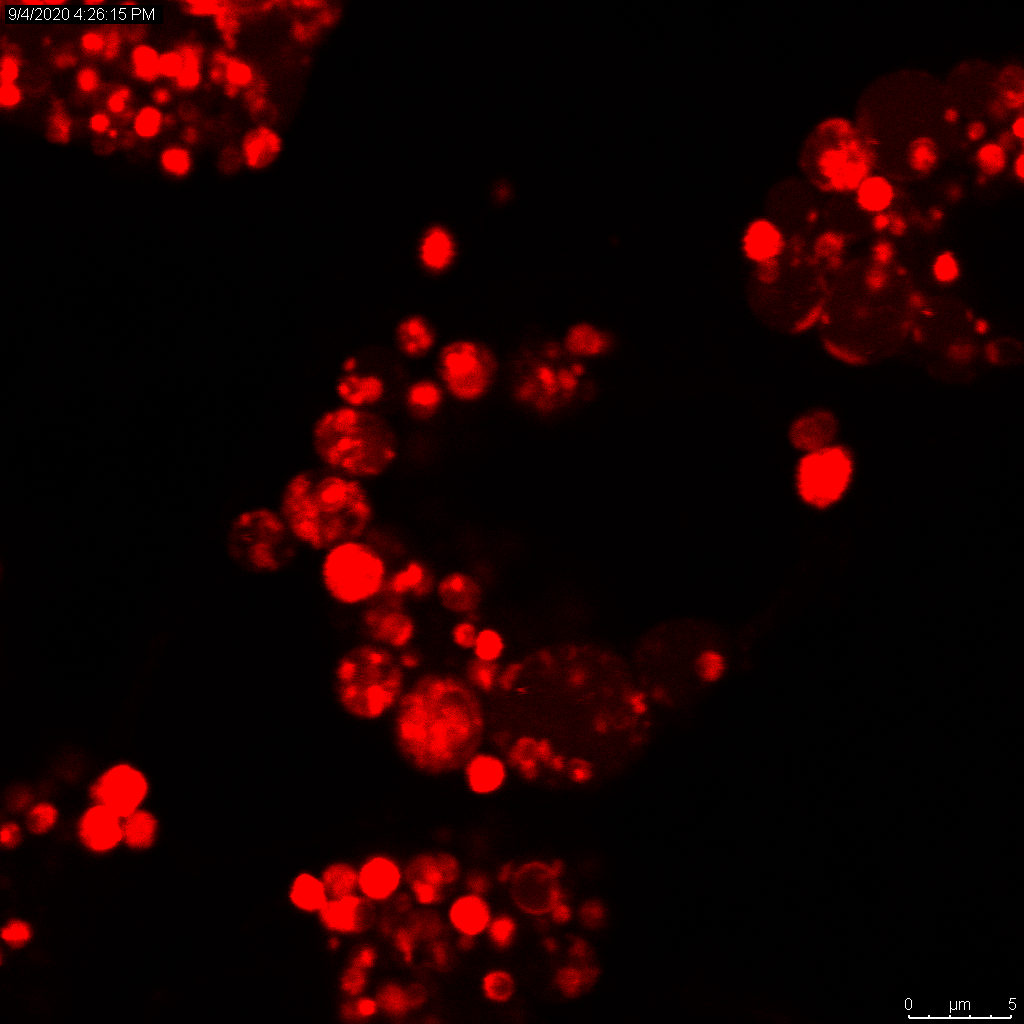

Supplement: Supplementary file 28 — Source data Fig. 2 [file 44318_2025_540_MOESM28_ESM.zip › SD Figure 2/2F/Microscopy MEF_LLOMe_Magic Red 6 hours Recovery.tif]

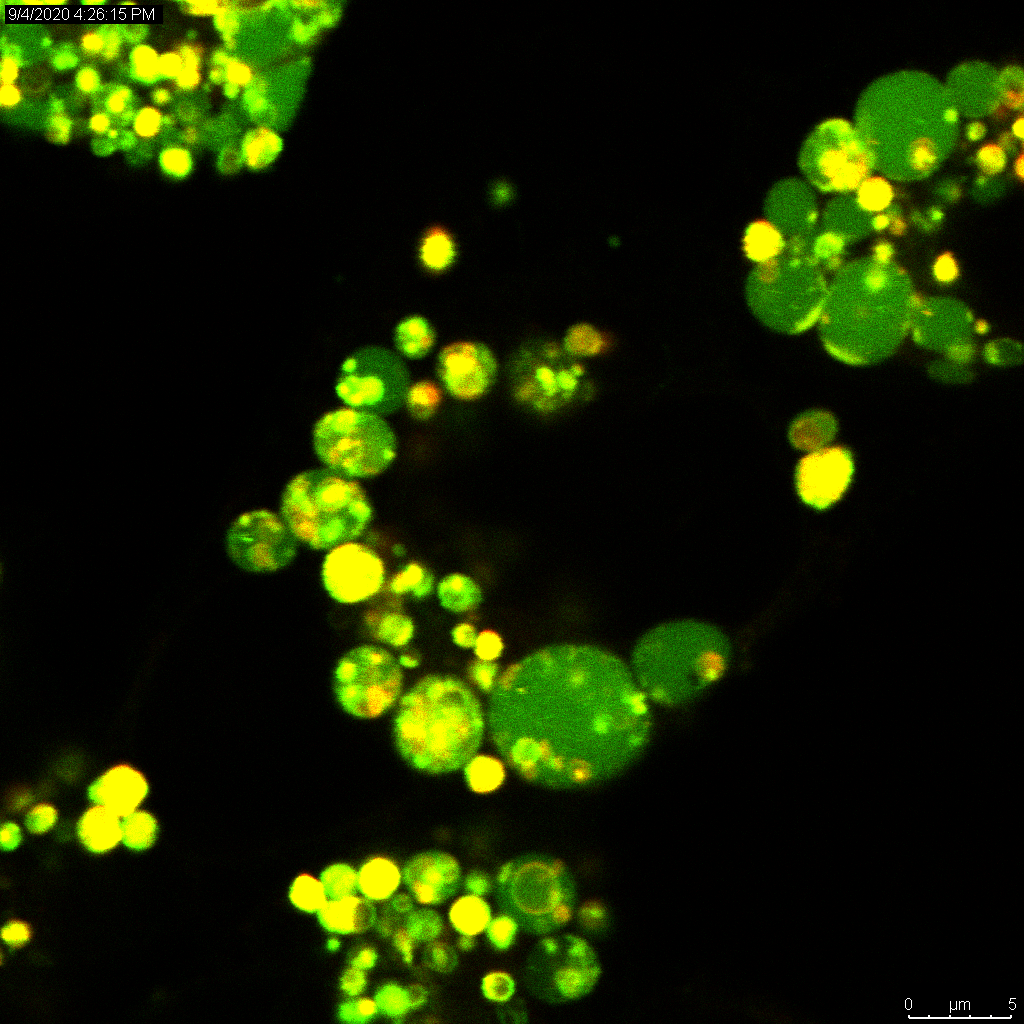

Supplement: Supplementary file 28 — Source data Fig. 2 [file 44318_2025_540_MOESM28_ESM.zip › SD Figure 2/2F/Microscopy MEF_LLOMe_Magic Red_Lysotracker Green_6 hours Recovery.tif]

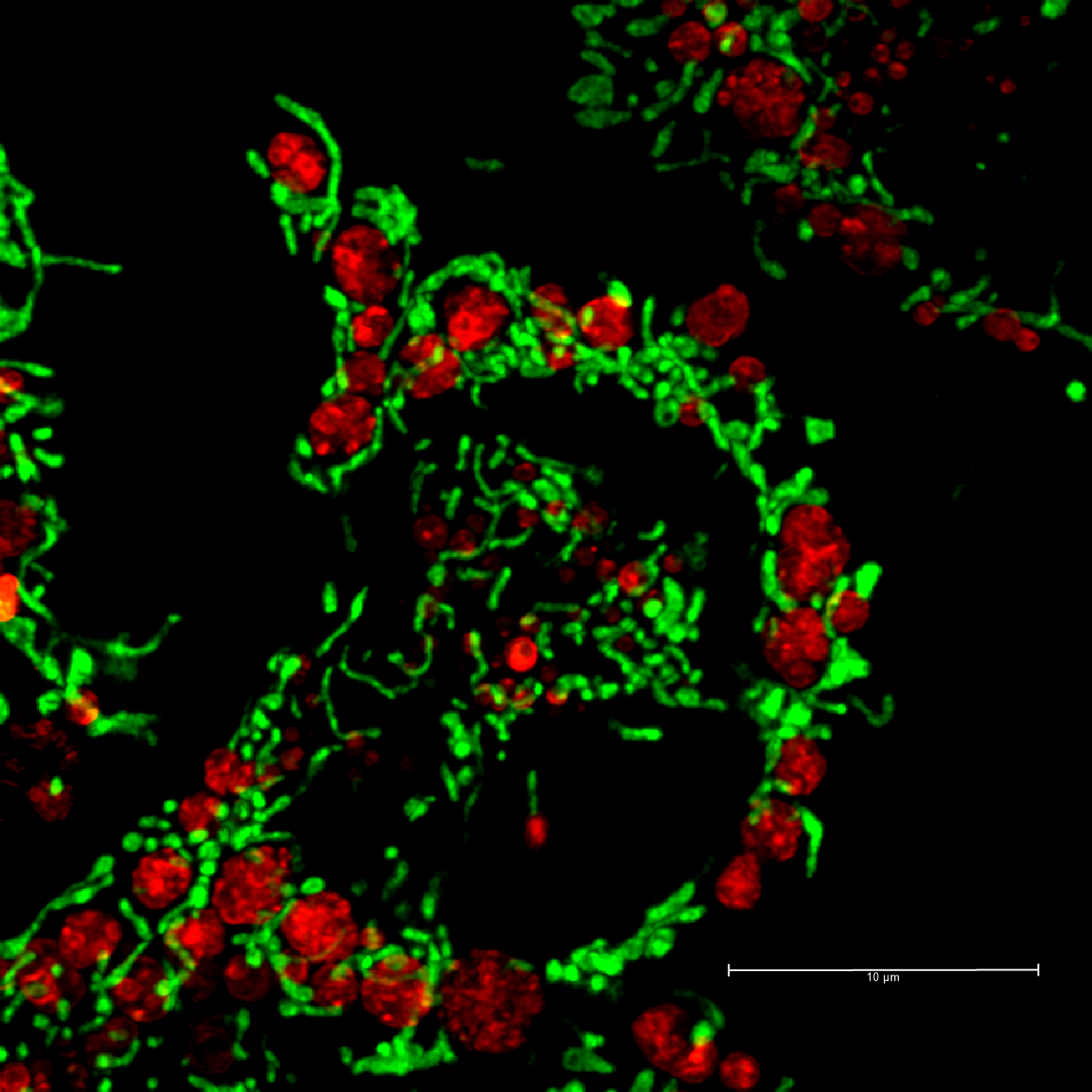

Supplement: Supplementary file 28 — Source data Fig. 2 [file 44318_2025_540_MOESM28_ESM.zip › SD Figure 2/2G/Microscopy Lysotracker Red_Mitotracker _6H .tif]

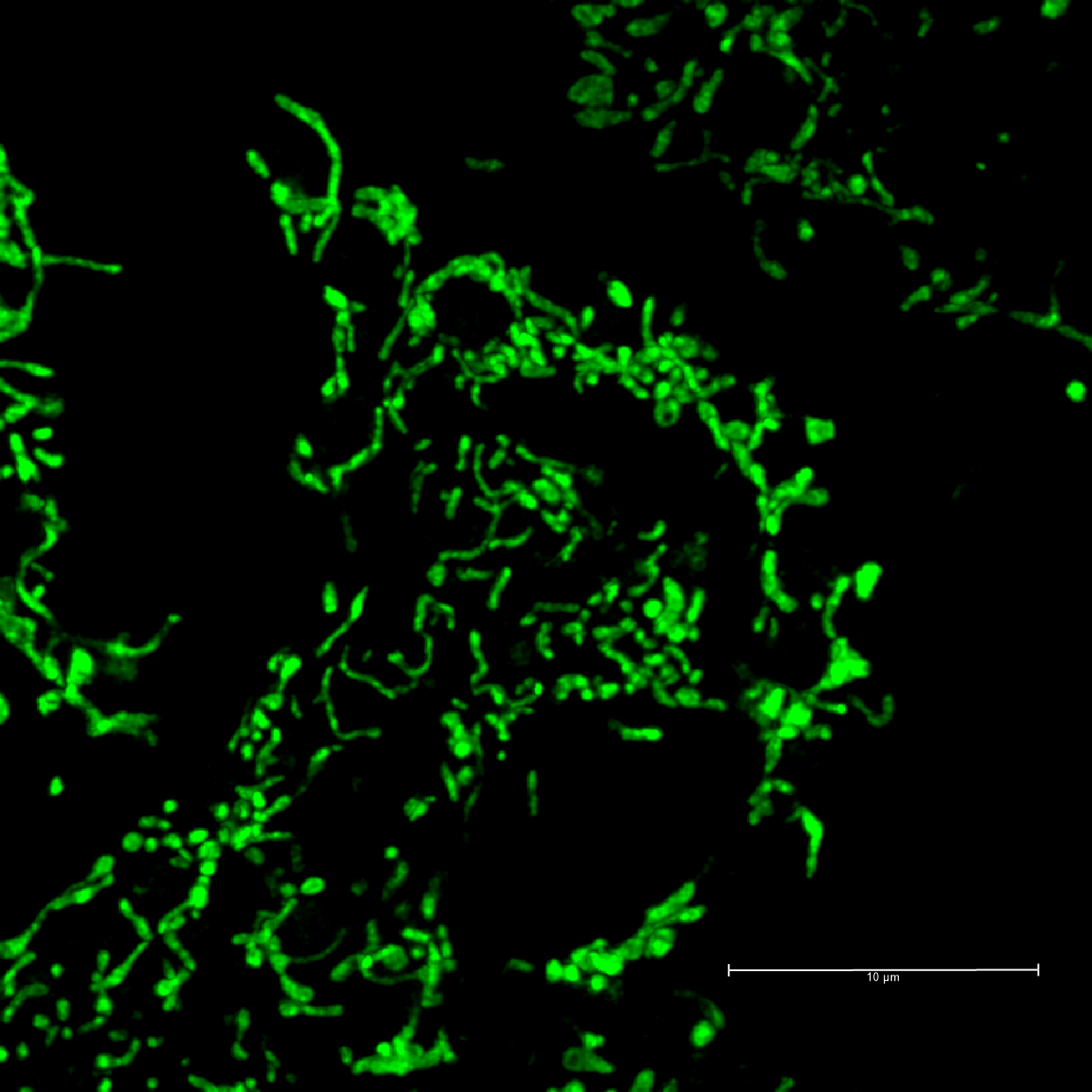

Supplement: Supplementary file 28 — Source data Fig. 2 [file 44318_2025_540_MOESM28_ESM.zip › SD Figure 2/2G/Microscopy Mitotracker.tif]

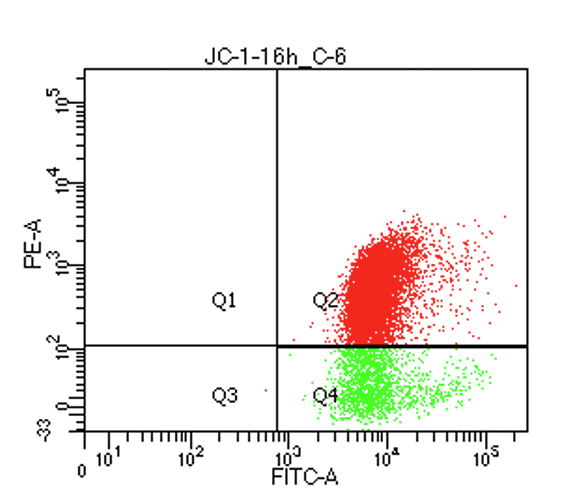

Supplement: Supplementary file 28 — Source data Fig. 2 [file 44318_2025_540_MOESM28_ESM.zip › SD Figure 2/2H/FACS 16h.png]

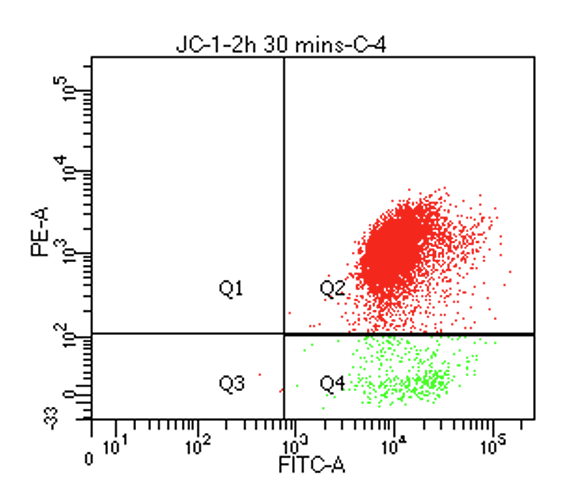

Supplement: Supplementary file 28 — Source data Fig. 2 [file 44318_2025_540_MOESM28_ESM.zip › SD Figure 2/2H/FACS 2h 30min.png]

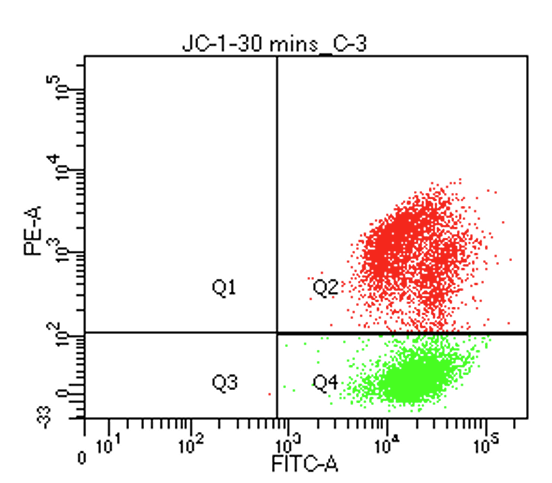

Supplement: Supplementary file 28 — Source data Fig. 2 [file 44318_2025_540_MOESM28_ESM.zip › SD Figure 2/2H/FACS 30min.png]

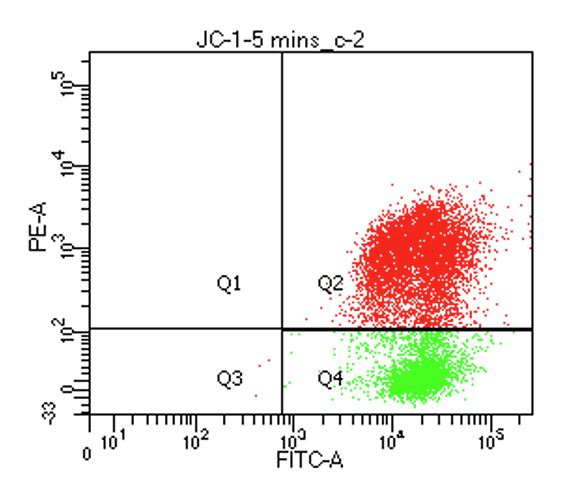

Supplement: Supplementary file 28 — Source data Fig. 2 [file 44318_2025_540_MOESM28_ESM.zip › SD Figure 2/2H/FACS 5min.png]

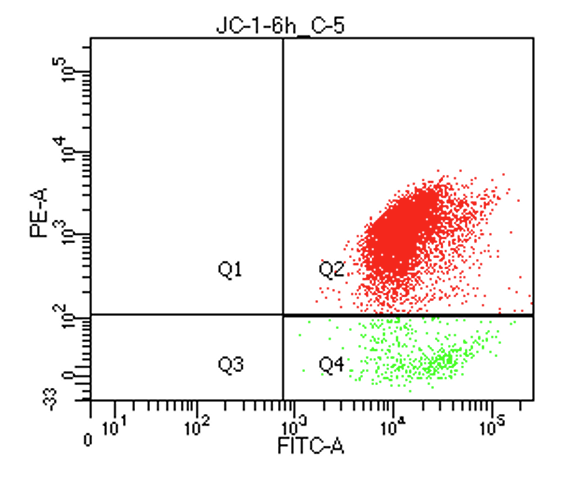

Supplement: Supplementary file 28 — Source data Fig. 2 [file 44318_2025_540_MOESM28_ESM.zip › SD Figure 2/2H/FACS 6h.png]

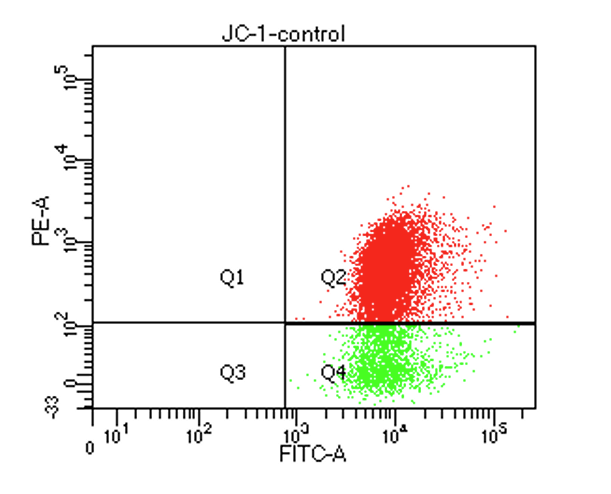

Supplement: Supplementary file 28 — Source data Fig. 2 [file 44318_2025_540_MOESM28_ESM.zip › SD Figure 2/2H/FACS Control.png]

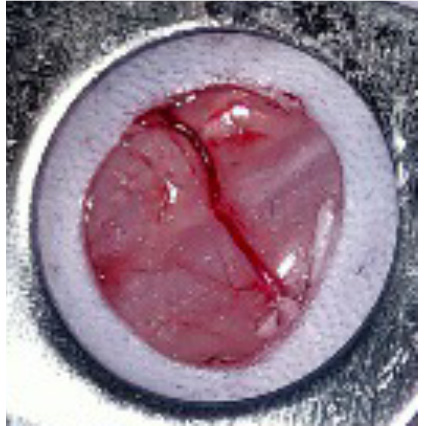

Supplement: Supplementary file 31 — Source data Fig. 6 [file 44318_2025_540_MOESM31_ESM.zip › SD Figure 6/6A/Macroscopic Photo 4mM LLOMe Day0.jpg]

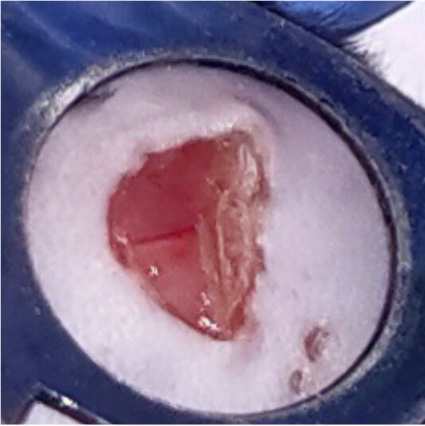

Supplement: Supplementary file 31 — Source data Fig. 6 [file 44318_2025_540_MOESM31_ESM.zip › SD Figure 6/6A/Macroscopic Photo 4mM LLOMe Day1.jpg]

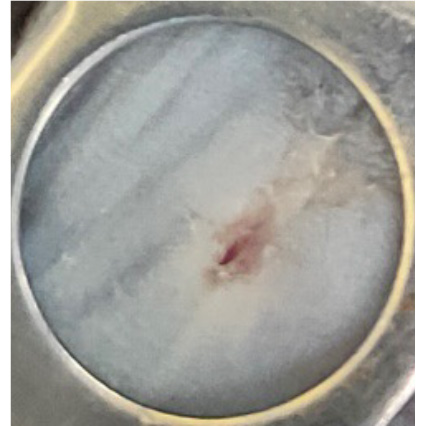

Supplement: Supplementary file 31 — Source data Fig. 6 [file 44318_2025_540_MOESM31_ESM.zip › SD Figure 6/6A/Macroscopic Photo 4mM LLOMe Day10.jpg]

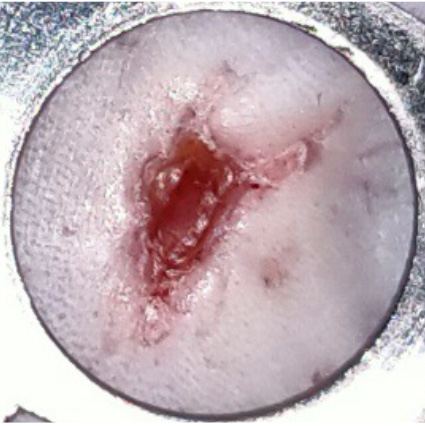

Supplement: Supplementary file 31 — Source data Fig. 6 [file 44318_2025_540_MOESM31_ESM.zip › SD Figure 6/6A/Macroscopic Photo 4mM LLOMe Day2.jpg]

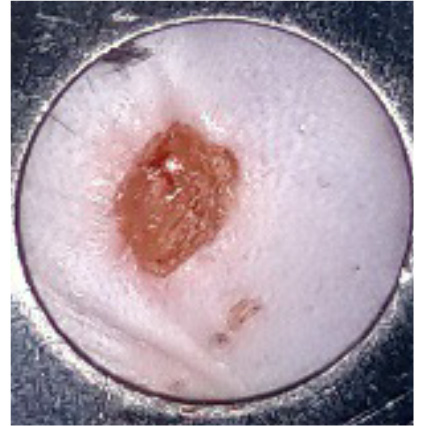

Supplement: Supplementary file 31 — Source data Fig. 6 [file 44318_2025_540_MOESM31_ESM.zip › SD Figure 6/6A/Macroscopic Photo 4mM LLOMe Day3.jpg]

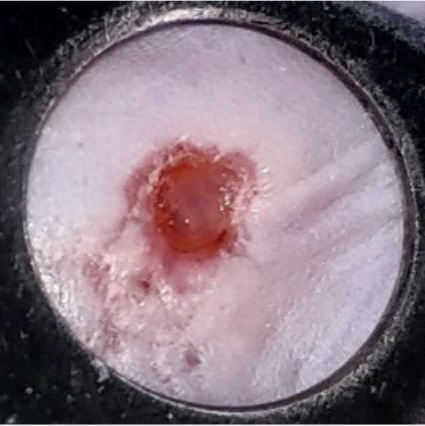

Supplement: Supplementary file 31 — Source data Fig. 6 [file 44318_2025_540_MOESM31_ESM.zip › SD Figure 6/6A/Macroscopic Photo 4mM LLOMe Day4.jpg]

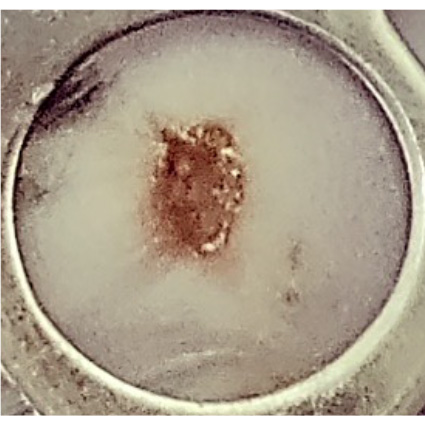

Supplement: Supplementary file 31 — Source data Fig. 6 [file 44318_2025_540_MOESM31_ESM.zip › SD Figure 6/6A/Macroscopic Photo 4mM LLOMe Day5.jpg]

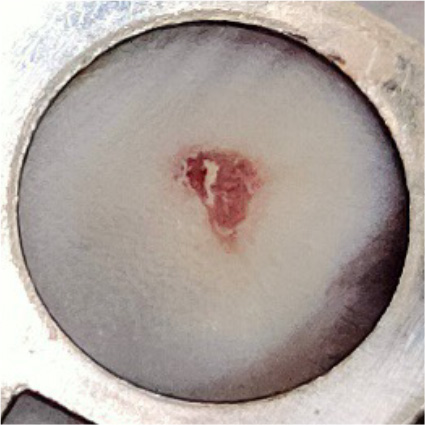

Supplement: Supplementary file 31 — Source data Fig. 6 [file 44318_2025_540_MOESM31_ESM.zip › SD Figure 6/6A/Macroscopic Photo 4mM LLOMe Day6.jpg]

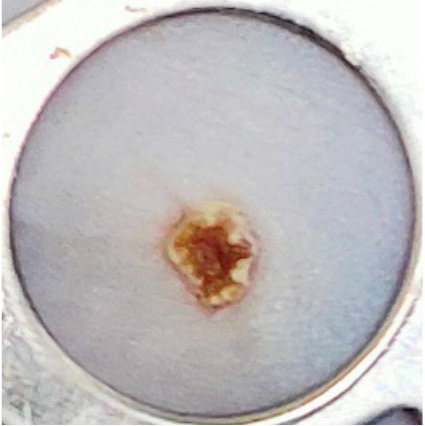

Supplement: Supplementary file 31 — Source data Fig. 6 [file 44318_2025_540_MOESM31_ESM.zip › SD Figure 6/6A/Macroscopic Photo 4mM LLOMe Day7.jpg]

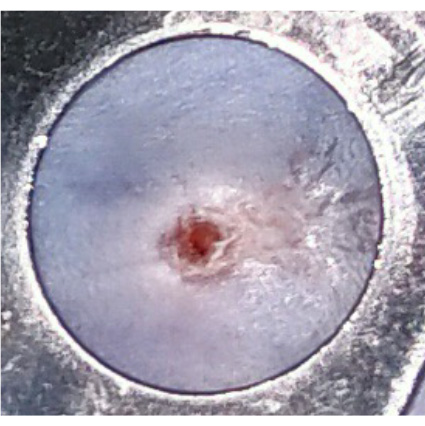

Supplement: Supplementary file 31 — Source data Fig. 6 [file 44318_2025_540_MOESM31_ESM.zip › SD Figure 6/6A/Macroscopic Photo 4mM LLOMe Day8.jpg]

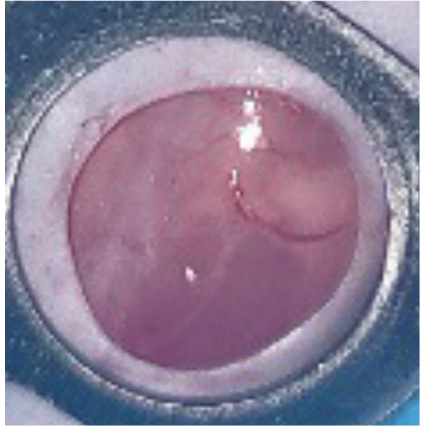

Supplement: Supplementary file 31 — Source data Fig. 6 [file 44318_2025_540_MOESM31_ESM.zip › SD Figure 6/6A/Macroscopic Photo 8mM LLOMe Day0.jpg]

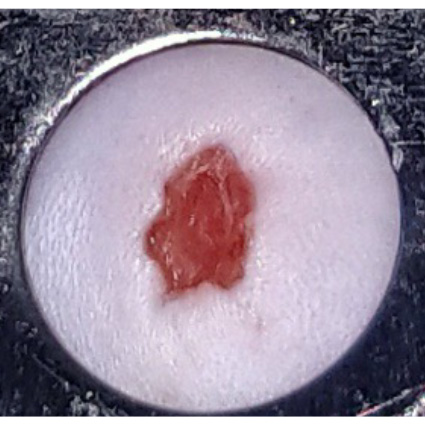

Supplement: Supplementary file 31 — Source data Fig. 6 [file 44318_2025_540_MOESM31_ESM.zip › SD Figure 6/6A/Macroscopic Photo 8mM LLOMe Day1.jpg]

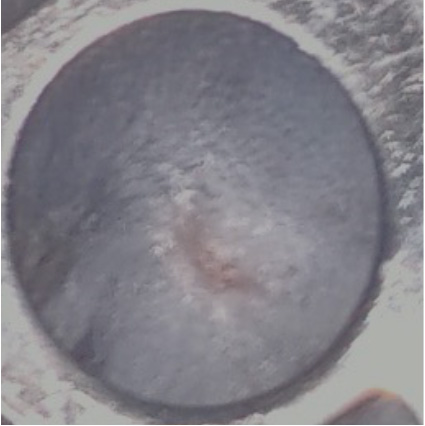

Supplement: Supplementary file 31 — Source data Fig. 6 [file 44318_2025_540_MOESM31_ESM.zip › SD Figure 6/6A/Macroscopic Photo 8mM LLOMe Day10.jpg]

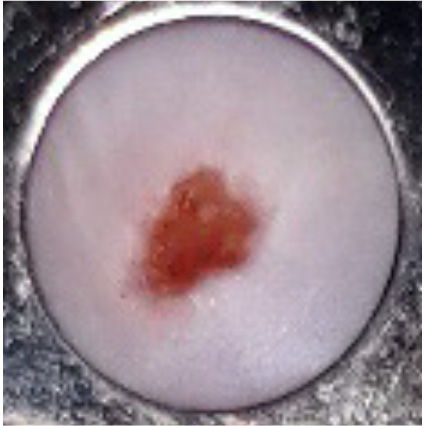

Supplement: Supplementary file 31 — Source data Fig. 6 [file 44318_2025_540_MOESM31_ESM.zip › SD Figure 6/6A/Macroscopic Photo 8mM LLOMe Day2.jpg]

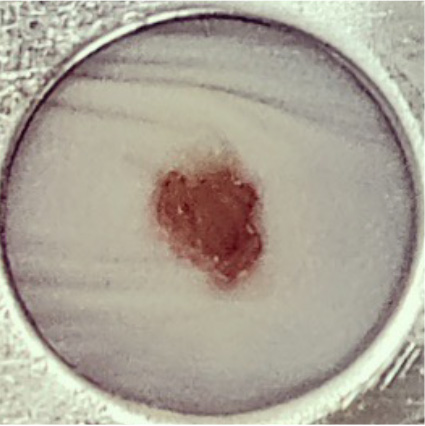

Supplement: Supplementary file 31 — Source data Fig. 6 [file 44318_2025_540_MOESM31_ESM.zip › SD Figure 6/6A/Macroscopic Photo 8mM LLOMe Day3.jpg]

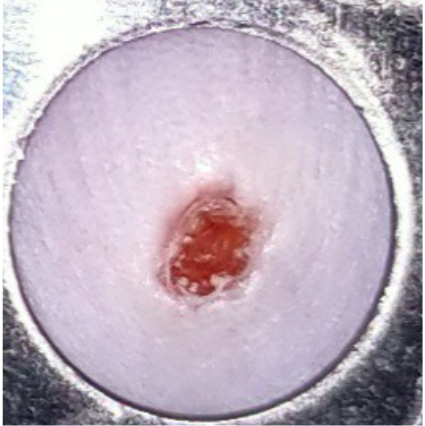

Supplement: Supplementary file 31 — Source data Fig. 6 [file 44318_2025_540_MOESM31_ESM.zip › SD Figure 6/6A/Macroscopic Photo 8mM LLOMe Day4.jpg]
