# Supplementary material for: Programmed cell revival from imminent cell death enhances tissue repair and regeneration
Source: EMBO J. 2025 Aug 21;44(19):5244–89. doi: 10.1038/s44318-025-00540-y (PMC12489119; doi:10.1038/s44318-025-00540-y)
Supplement: Supplementary file 29 — Source data Fig. 3 [file 44318_2025_540_MOESM29_ESM.zip › SD Figure 3/3L/Western Blot.pdf]

Figure-3

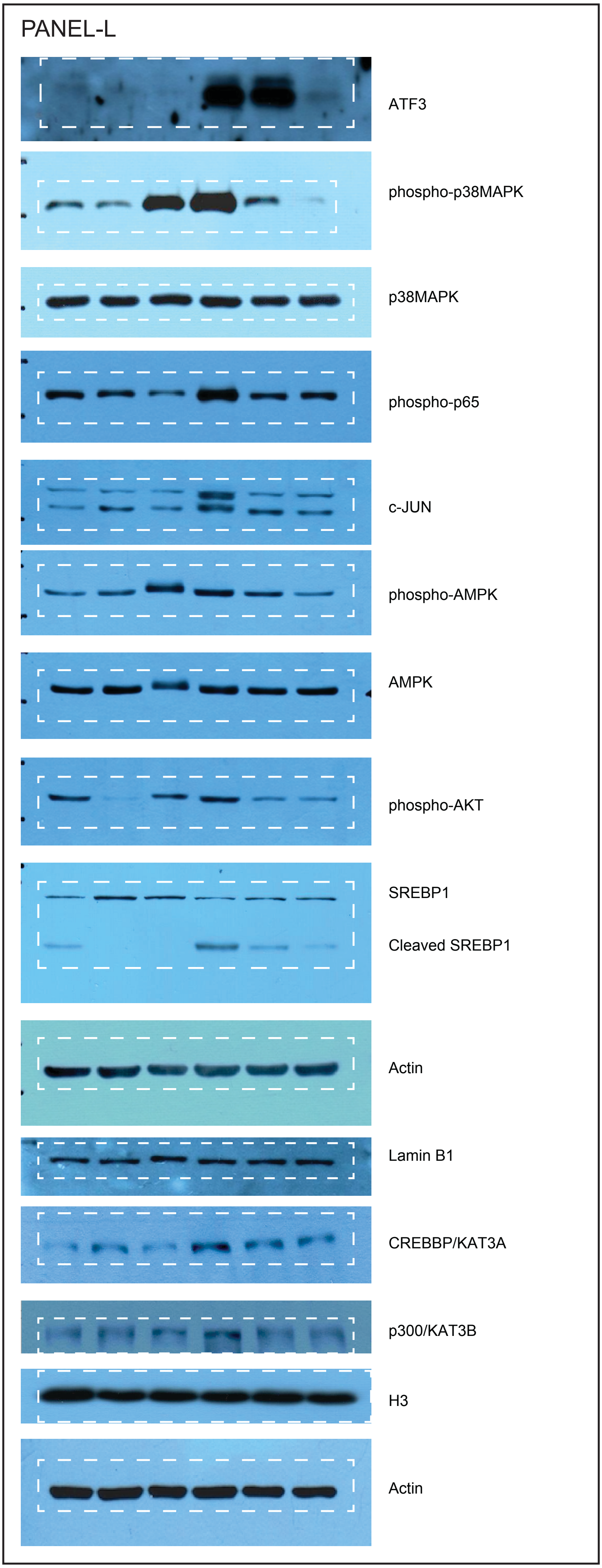

PANEL-L
ATF3
phospho-p38MAPK
p38MAPK
phospho-p65
c-JUN
phospho-AMPK
AMPK
phospho-AKT
SREBP1
Cleaved SREBP1
Actin
Lamin B1
CREBBP/KAT3A
p300/KAT3B
H3
Actin
