# Supplementary figures and images for: Programmed cell revival from imminent cell death enhances tissue repair and regeneration (part 2 of 4)
Source: EMBO J. 2025 Aug 21;44(19):5244–89. doi: 10.1038/s44318-025-00540-y (PMC12489119; doi:10.1038/s44318-025-00540-y)

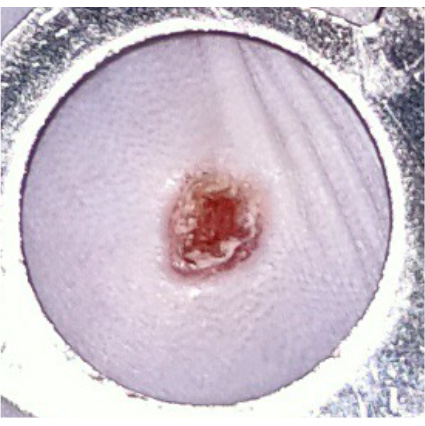

Supplement: Supplementary file 31 — Source data Fig. 6 [file 44318_2025_540_MOESM31_ESM.zip › SD Figure 6/6A/Macroscopic Photo 8mM LLOMe Day5.jpg]

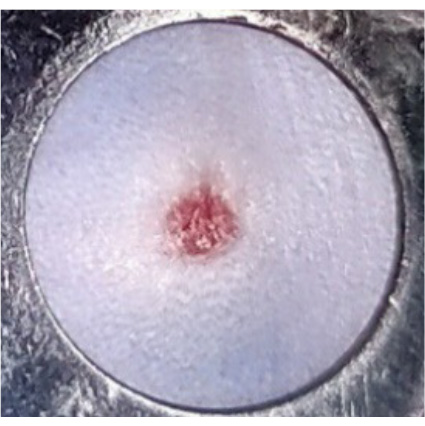

Supplement: Supplementary file 31 — Source data Fig. 6 [file 44318_2025_540_MOESM31_ESM.zip › SD Figure 6/6A/Macroscopic Photo 8mM LLOMe Day6.jpg]

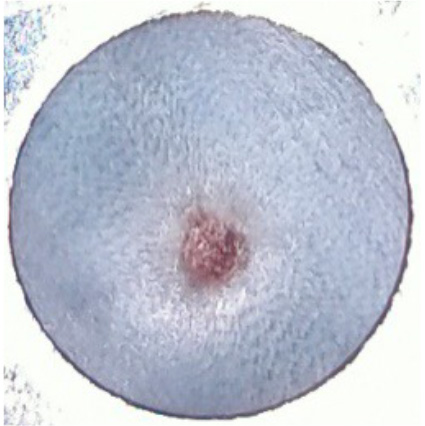

Supplement: Supplementary file 31 — Source data Fig. 6 [file 44318_2025_540_MOESM31_ESM.zip › SD Figure 6/6A/Macroscopic Photo 8mM LLOMe Day7.jpg]

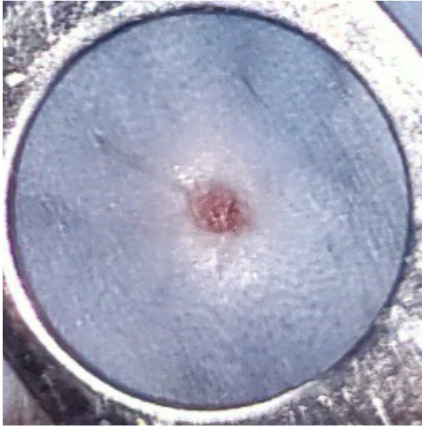

Supplement: Supplementary file 31 — Source data Fig. 6 [file 44318_2025_540_MOESM31_ESM.zip › SD Figure 6/6A/Macroscopic Photo 8mM LLOMe Day8.jpg]

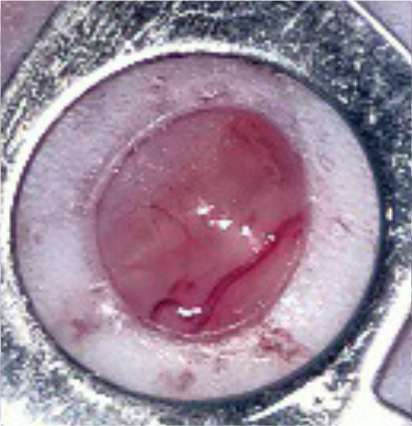

Supplement: Supplementary file 31 — Source data Fig. 6 [file 44318_2025_540_MOESM31_ESM.zip › SD Figure 6/6A/Macroscopic Photo Control Day0.jpg]

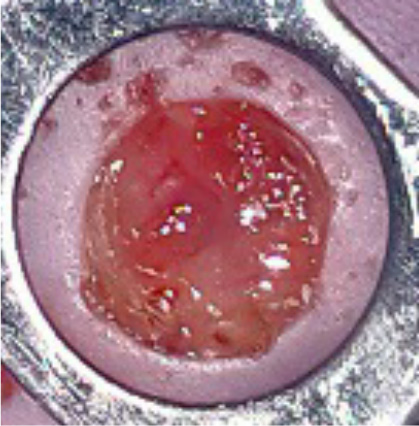

Supplement: Supplementary file 31 — Source data Fig. 6 [file 44318_2025_540_MOESM31_ESM.zip › SD Figure 6/6A/Macroscopic Photo Control Day1.jpg]

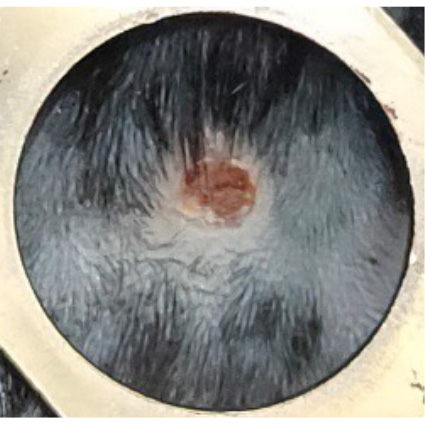

Supplement: Supplementary file 31 — Source data Fig. 6 [file 44318_2025_540_MOESM31_ESM.zip › SD Figure 6/6A/Macroscopic Photo Control Day10.jpg]

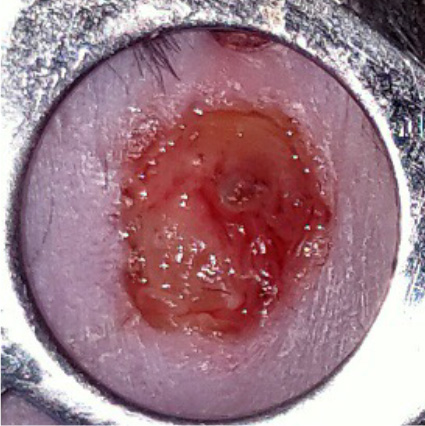

Supplement: Supplementary file 31 — Source data Fig. 6 [file 44318_2025_540_MOESM31_ESM.zip › SD Figure 6/6A/Macroscopic Photo Control Day2.jpg]

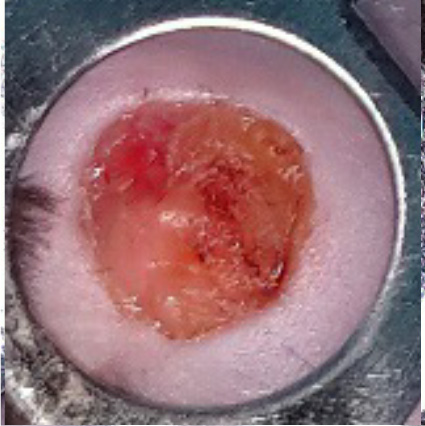

Supplement: Supplementary file 31 — Source data Fig. 6 [file 44318_2025_540_MOESM31_ESM.zip › SD Figure 6/6A/Macroscopic Photo Control Day3.jpg]

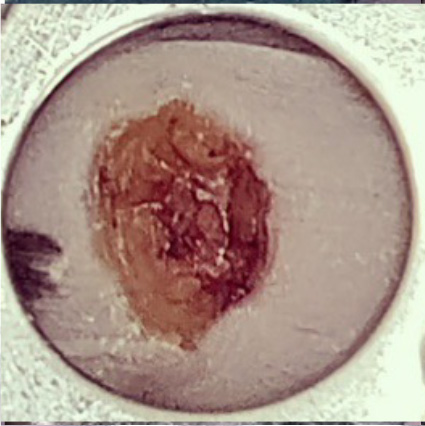

Supplement: Supplementary file 31 — Source data Fig. 6 [file 44318_2025_540_MOESM31_ESM.zip › SD Figure 6/6A/Macroscopic Photo Control Day4.jpg]

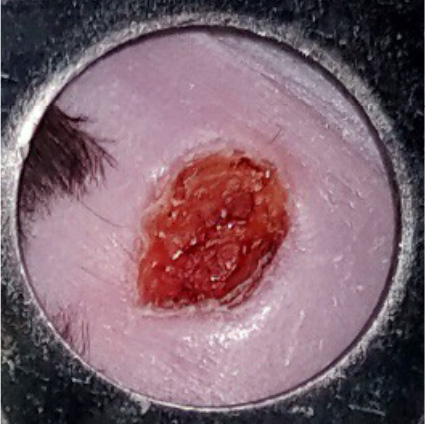

Supplement: Supplementary file 31 — Source data Fig. 6 [file 44318_2025_540_MOESM31_ESM.zip › SD Figure 6/6A/Macroscopic Photo Control Day5.jpg]

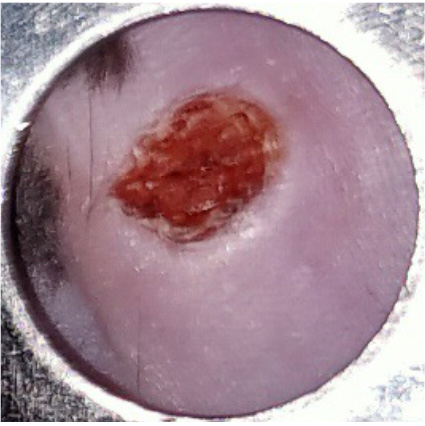

Supplement: Supplementary file 31 — Source data Fig. 6 [file 44318_2025_540_MOESM31_ESM.zip › SD Figure 6/6A/Macroscopic Photo Control Day6.jpg]

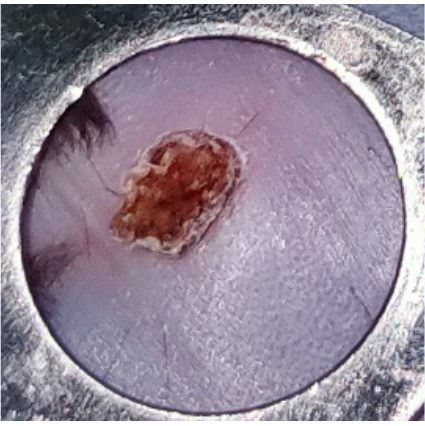

Supplement: Supplementary file 31 — Source data Fig. 6 [file 44318_2025_540_MOESM31_ESM.zip › SD Figure 6/6A/Macroscopic Photo Control Day7.jpg]

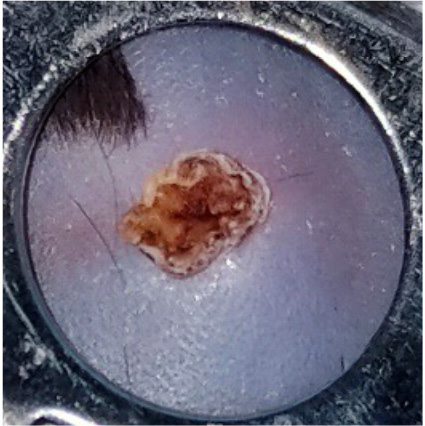

Supplement: Supplementary file 31 — Source data Fig. 6 [file 44318_2025_540_MOESM31_ESM.zip › SD Figure 6/6A/Macroscopic Photo Control Day8.jpg]

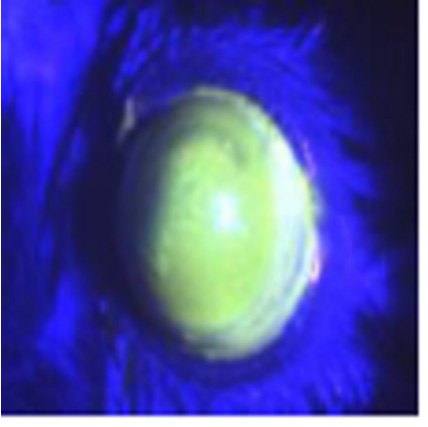

Supplement: Supplementary file 31 — Source data Fig. 6 [file 44318_2025_540_MOESM31_ESM.zip › SD Figure 6/6D/Macroscopic Photo Injured Control1 Day0.jpg]

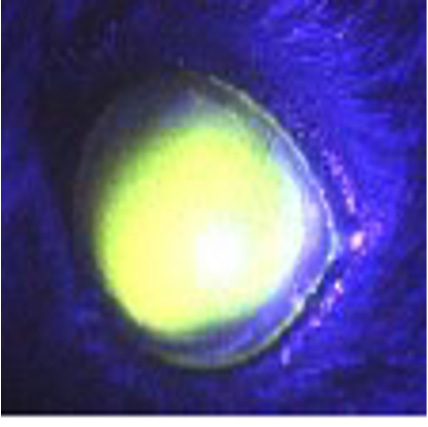

Supplement: Supplementary file 31 — Source data Fig. 6 [file 44318_2025_540_MOESM31_ESM.zip › SD Figure 6/6D/Macroscopic Photo Injured Control1 Day2.jpg]

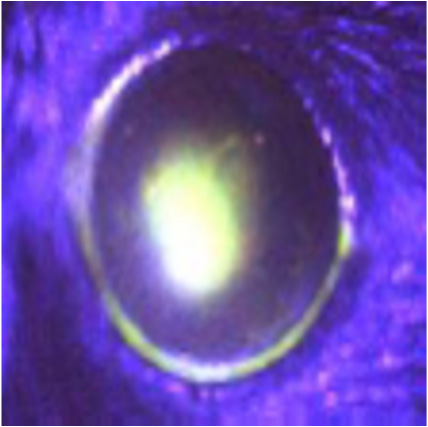

Supplement: Supplementary file 31 — Source data Fig. 6 [file 44318_2025_540_MOESM31_ESM.zip › SD Figure 6/6D/Macroscopic Photo Injured Control1 Day5.jpg]

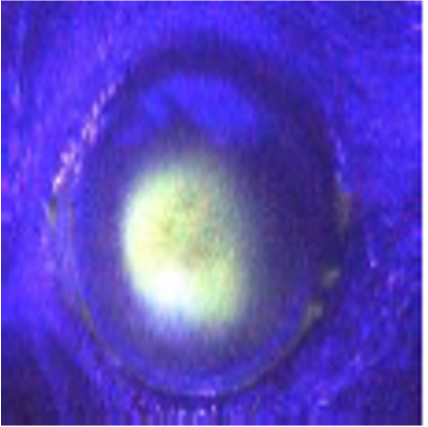

Supplement: Supplementary file 31 — Source data Fig. 6 [file 44318_2025_540_MOESM31_ESM.zip › SD Figure 6/6D/Macroscopic Photo Injured Control1 Day7.jpg]

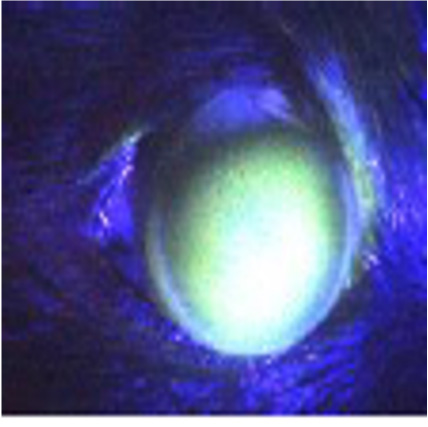

Supplement: Supplementary file 31 — Source data Fig. 6 [file 44318_2025_540_MOESM31_ESM.zip › SD Figure 6/6D/Macroscopic Photo Injured Control2 Day0.jpg]

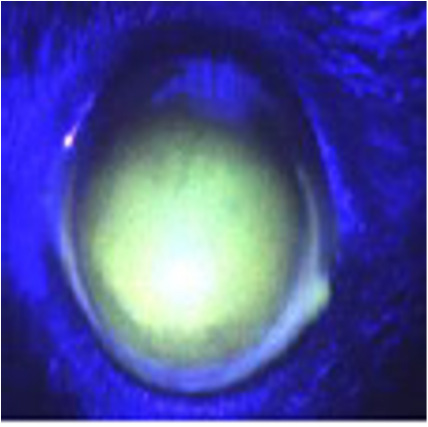

Supplement: Supplementary file 31 — Source data Fig. 6 [file 44318_2025_540_MOESM31_ESM.zip › SD Figure 6/6D/Macroscopic Photo Injured Control2 Day2.jpg]

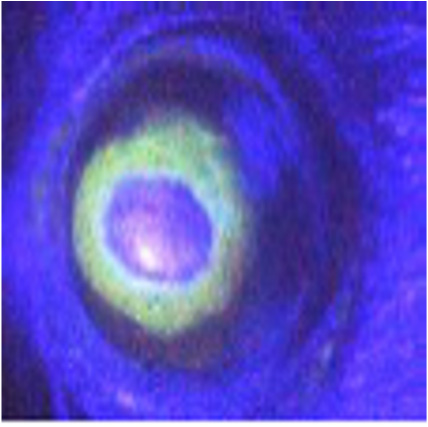

Supplement: Supplementary file 31 — Source data Fig. 6 [file 44318_2025_540_MOESM31_ESM.zip › SD Figure 6/6D/Macroscopic Photo Injured Control2 Day5.jpg]

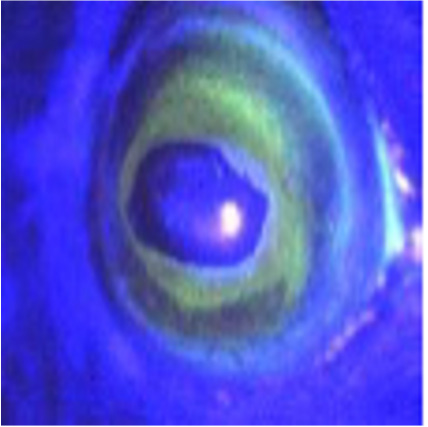

Supplement: Supplementary file 31 — Source data Fig. 6 [file 44318_2025_540_MOESM31_ESM.zip › SD Figure 6/6D/Macroscopic Photo Injured Control2 Day7.jpg]

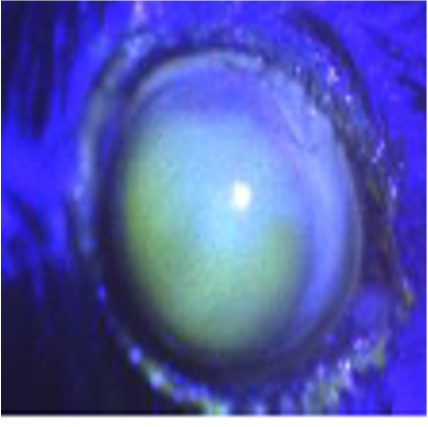

Supplement: Supplementary file 31 — Source data Fig. 6 [file 44318_2025_540_MOESM31_ESM.zip › SD Figure 6/6D/Macroscopic Photo Injured LLOMe1 Day0.jpg]

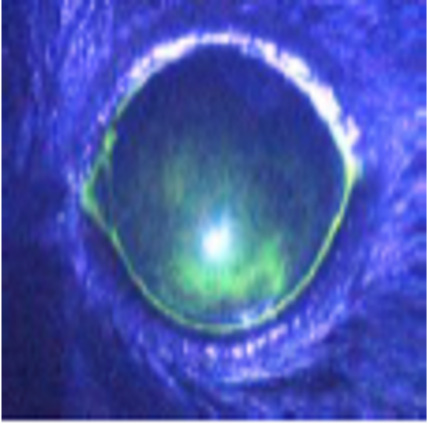

Supplement: Supplementary file 31 — Source data Fig. 6 [file 44318_2025_540_MOESM31_ESM.zip › SD Figure 6/6D/Macroscopic Photo Injured LLOMe1 Day2.jpg]

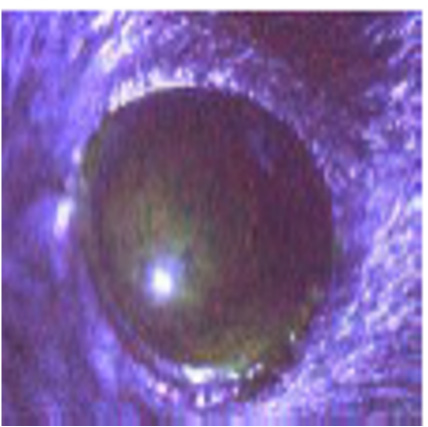

Supplement: Supplementary file 31 — Source data Fig. 6 [file 44318_2025_540_MOESM31_ESM.zip › SD Figure 6/6D/Macroscopic Photo Injured LLOMe1 Day5.jpg]

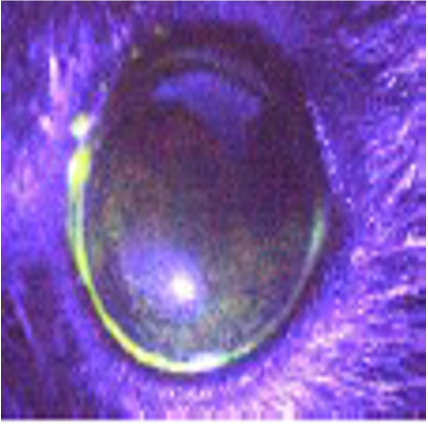

Supplement: Supplementary file 31 — Source data Fig. 6 [file 44318_2025_540_MOESM31_ESM.zip › SD Figure 6/6D/Macroscopic Photo Injured LLOMe1 Day7.jpg]

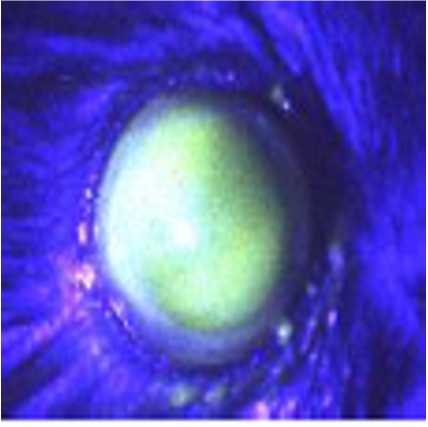

Supplement: Supplementary file 31 — Source data Fig. 6 [file 44318_2025_540_MOESM31_ESM.zip › SD Figure 6/6D/Macroscopic Photo Injured LLOMe2 Day0.jpg]

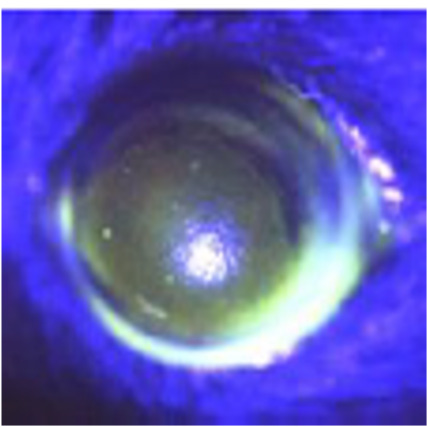

Supplement: Supplementary file 31 — Source data Fig. 6 [file 44318_2025_540_MOESM31_ESM.zip › SD Figure 6/6D/Macroscopic Photo Injured LLOMe2 Day2.jpg]

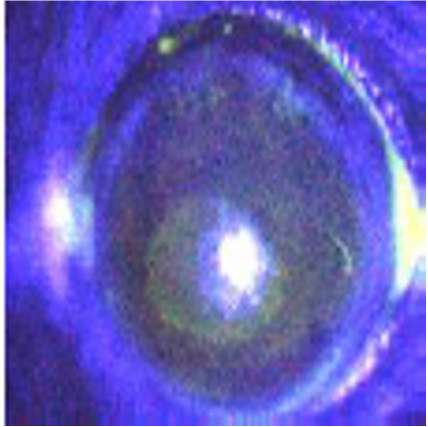

Supplement: Supplementary file 31 — Source data Fig. 6 [file 44318_2025_540_MOESM31_ESM.zip › SD Figure 6/6D/Macroscopic Photo Injured LLOMe2 Day5.jpg]

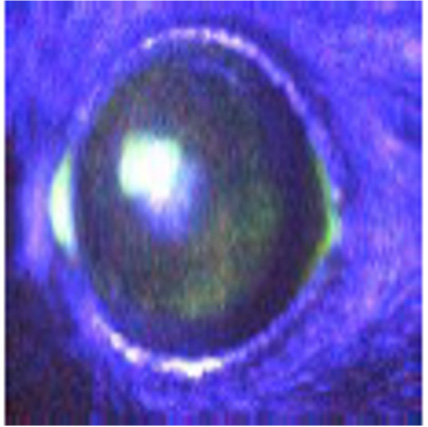

Supplement: Supplementary file 31 — Source data Fig. 6 [file 44318_2025_540_MOESM31_ESM.zip › SD Figure 6/6D/Macroscopic Photo Injured LLOMe2 Day7.jpg]

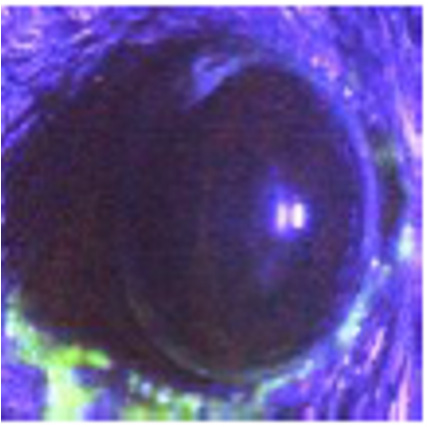

Supplement: Supplementary file 31 — Source data Fig. 6 [file 44318_2025_540_MOESM31_ESM.zip › SD Figure 6/6D/Macroscopic Photo Uninjured Control Day0.jpg]

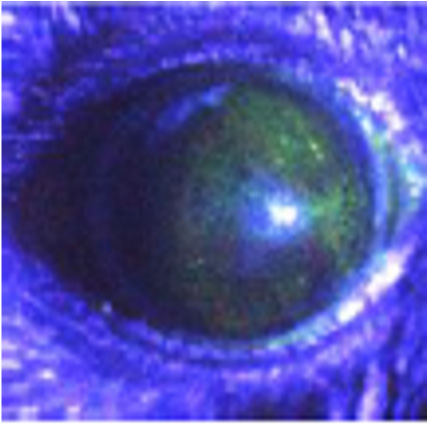

Supplement: Supplementary file 31 — Source data Fig. 6 [file 44318_2025_540_MOESM31_ESM.zip › SD Figure 6/6D/Macroscopic Photo Uninjured Control Day7.jpg]

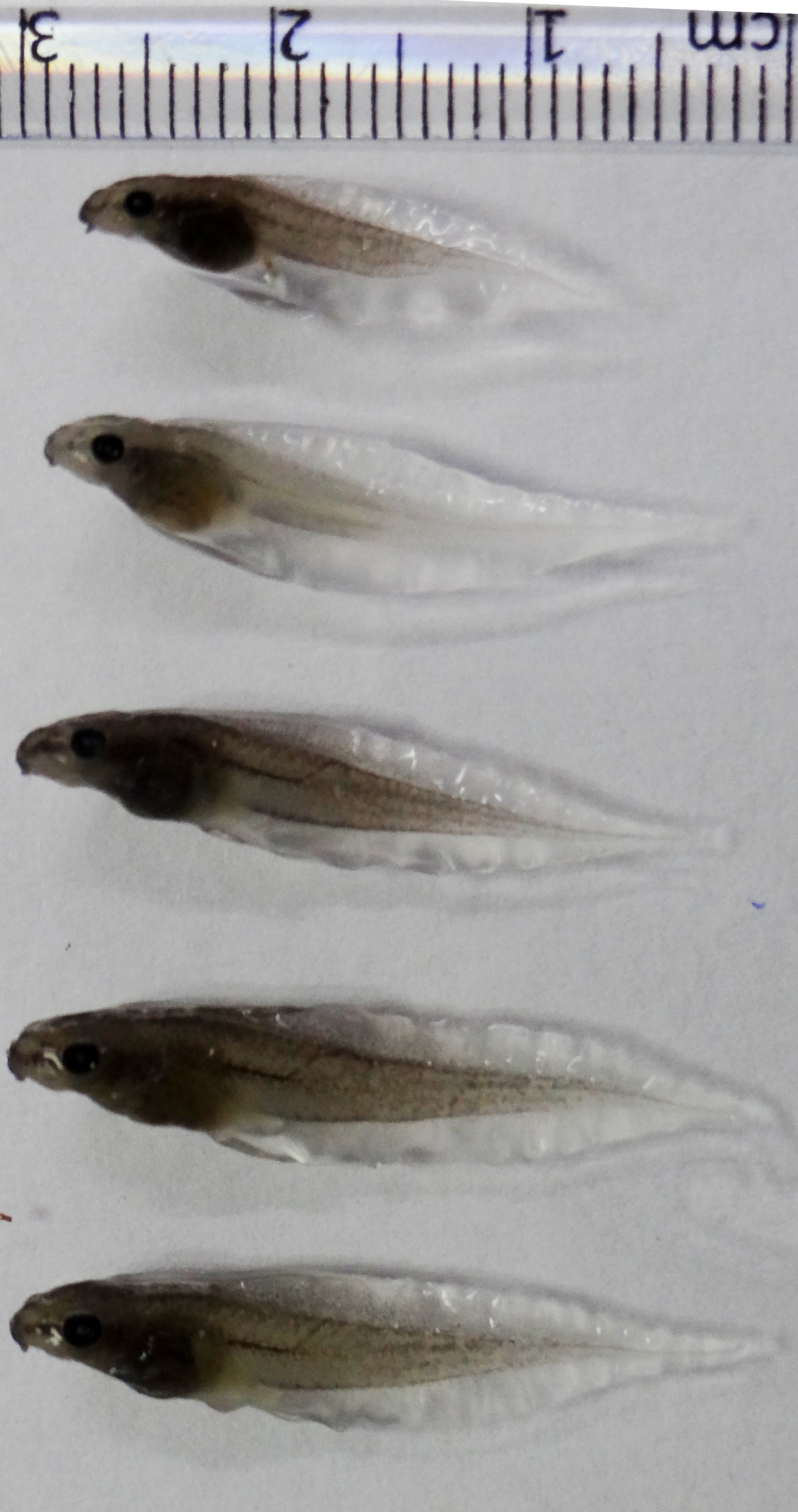

Supplement: Supplementary file 31 — Source data Fig. 6 [file 44318_2025_540_MOESM31_ESM.zip › SD Figure 6/6G/Macroscopic Photo Day 9.JPG]

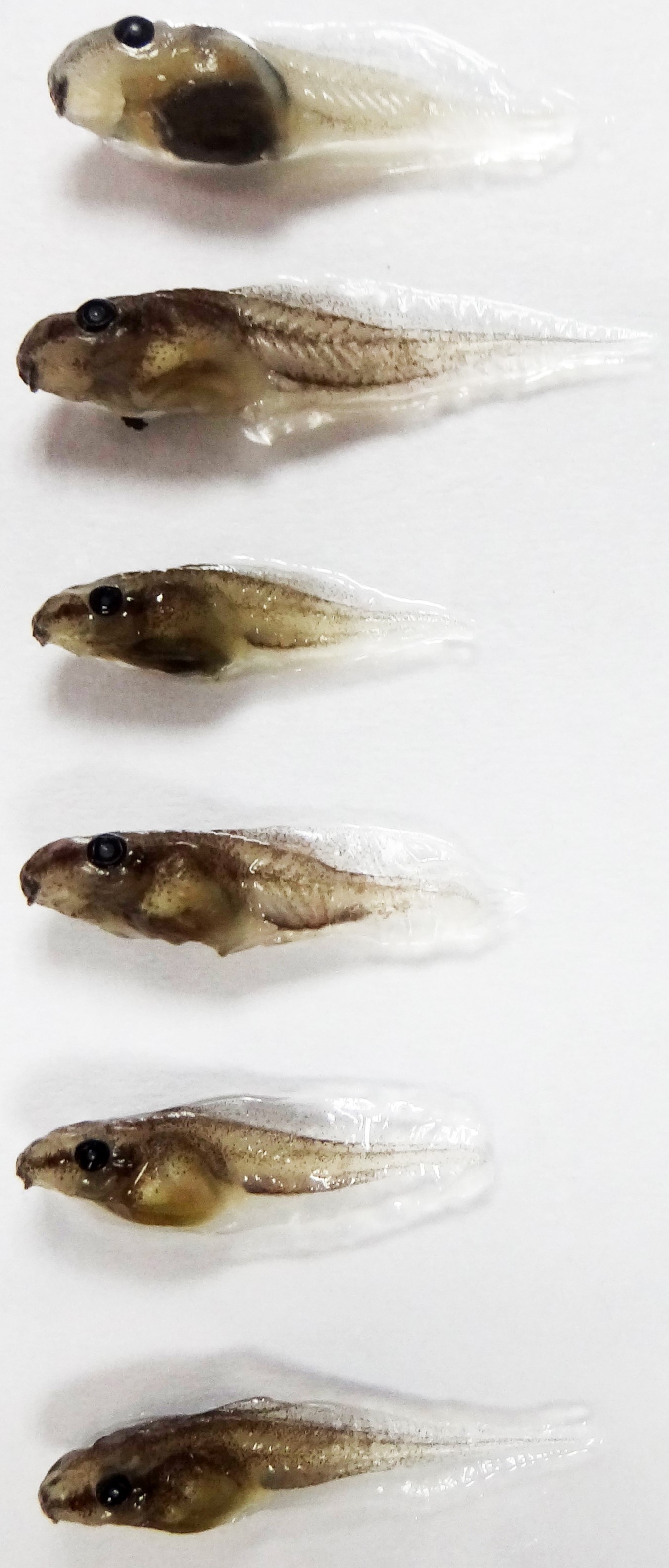

Supplement: Supplementary file 31 — Source data Fig. 6 [file 44318_2025_540_MOESM31_ESM.zip › SD Figure 6/6I/Macroscopic Photo Day 7.jpg]

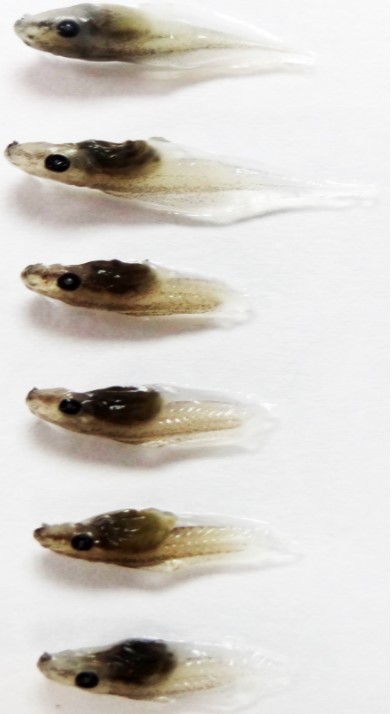

Supplement: Supplementary file 31 — Source data Fig. 6 [file 44318_2025_540_MOESM31_ESM.zip › SD Figure 6/6I/Macroscopic Photo DAY-5.jpg]

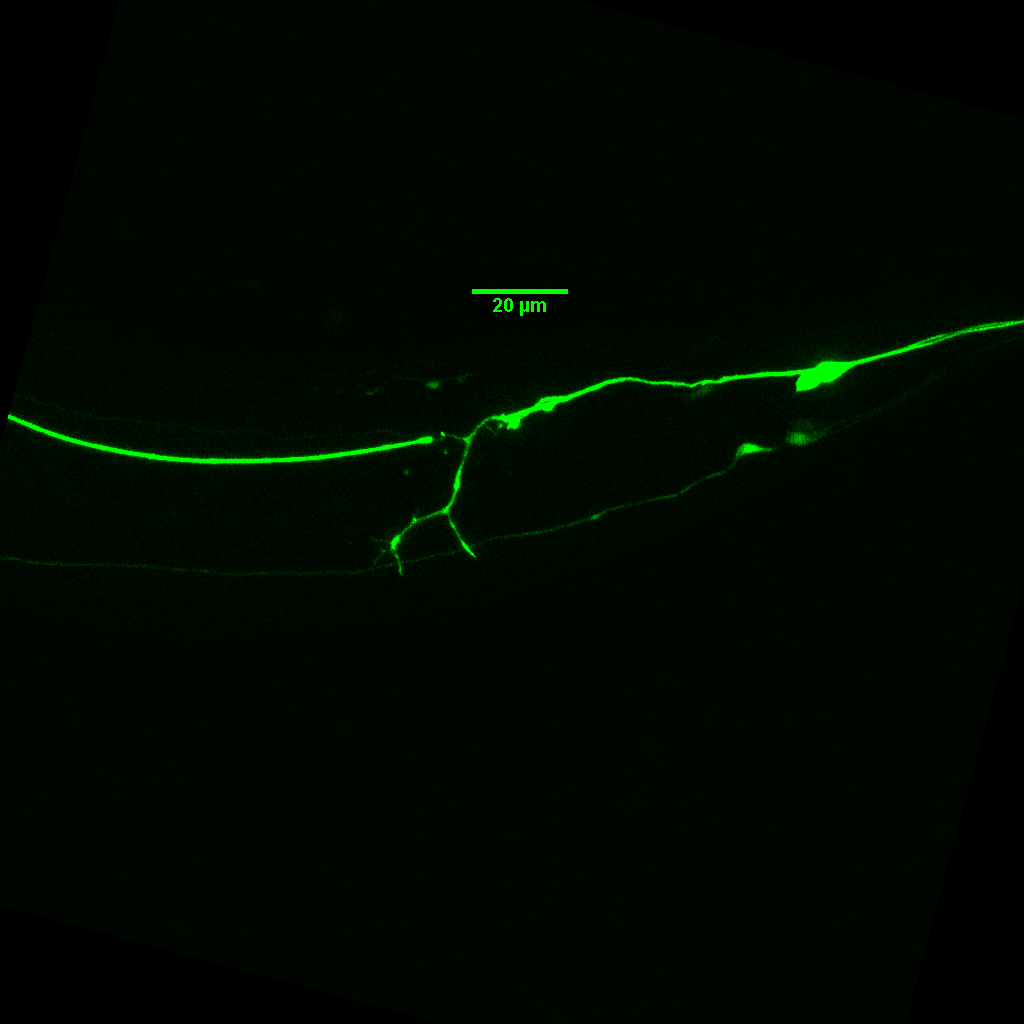

Supplement: Supplementary file 32 — Source data Fig. 7 [file 44318_2025_540_MOESM32_ESM.zip › SD Figure 7/7B/Microscopy 10mM LLOMe.tif]

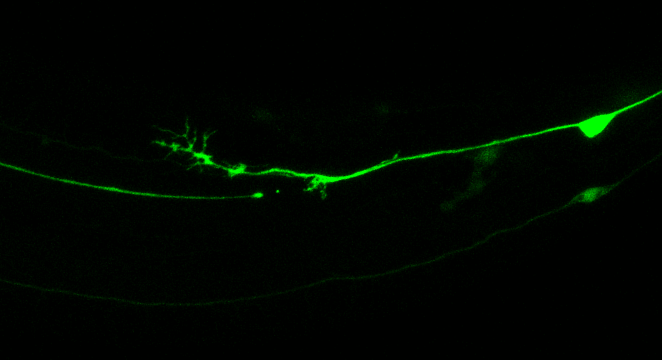

Supplement: Supplementary file 32 — Source data Fig. 7 [file 44318_2025_540_MOESM32_ESM.zip › SD Figure 7/7B/Microscopy Control.tif]

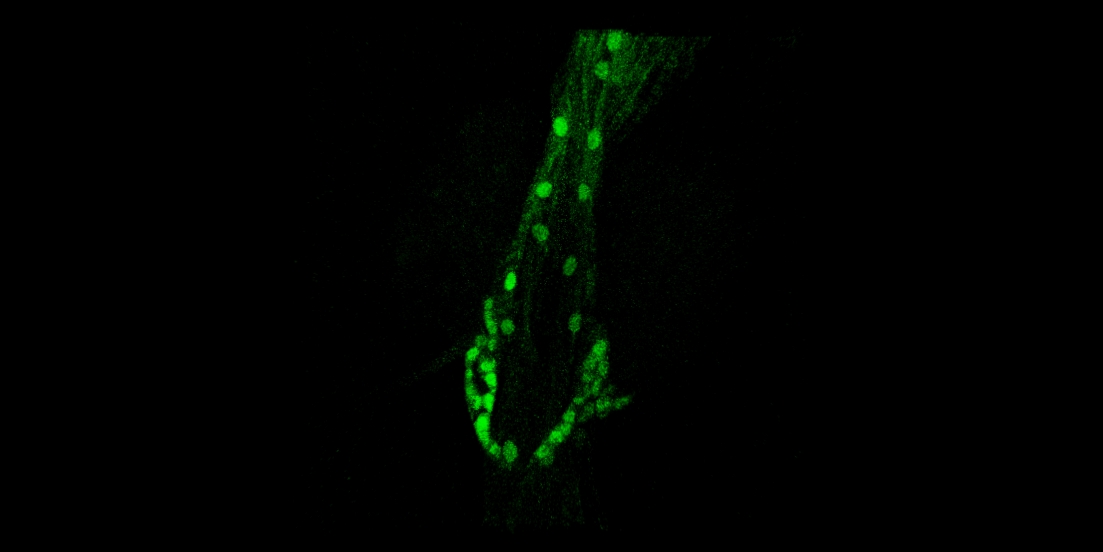

Supplement: Supplementary file 32 — Source data Fig. 7 [file 44318_2025_540_MOESM32_ESM.zip › SD Figure 7/7F/Microscopy 10X STATGFP Control_GFP.jpg]

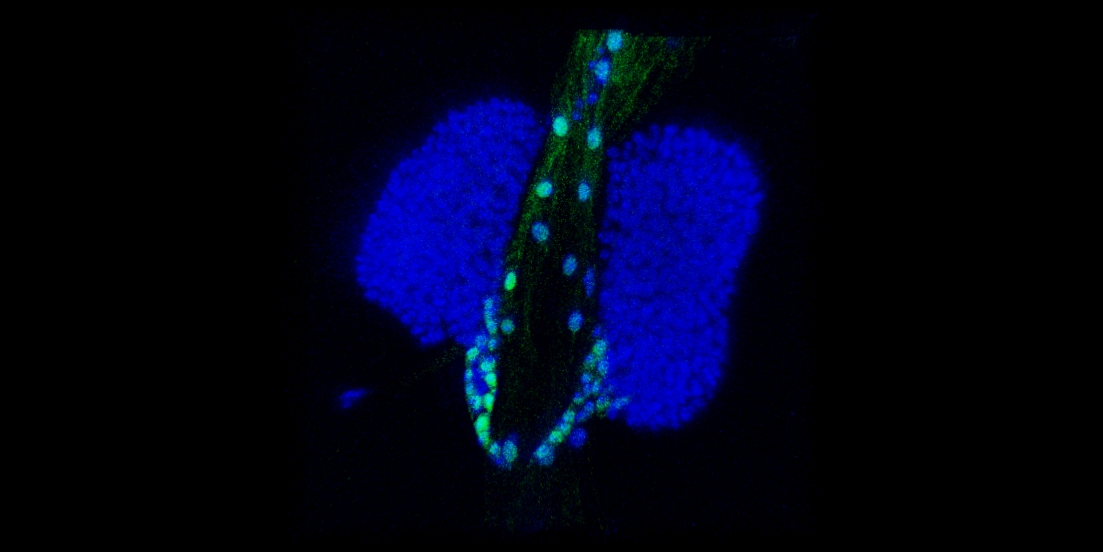

Supplement: Supplementary file 32 — Source data Fig. 7 [file 44318_2025_540_MOESM32_ESM.zip › SD Figure 7/7F/Microscopy 10X STATGFP Control_GFP_DAPI.jpg]

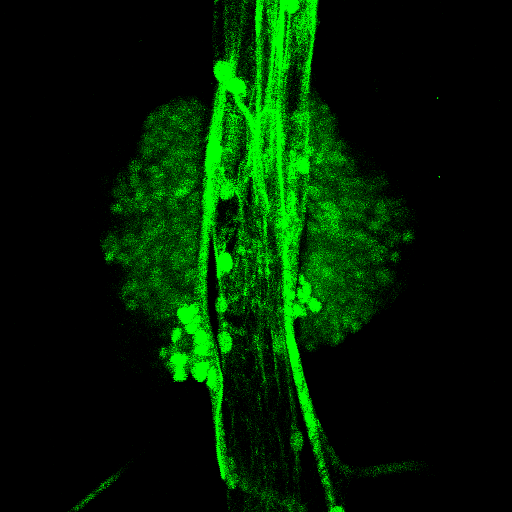

Supplement: Supplementary file 32 — Source data Fig. 7 [file 44318_2025_540_MOESM32_ESM.zip › SD Figure 7/7F/Microscopy 10XSTATGFP LLOMe_GFP.tif]

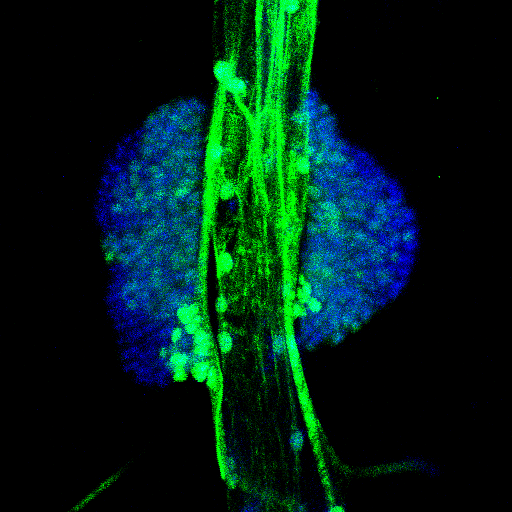

Supplement: Supplementary file 32 — Source data Fig. 7 [file 44318_2025_540_MOESM32_ESM.zip › SD Figure 7/7F/Microscopy 10XSTATGFP LLOMe_GFP_DAPI.tif]

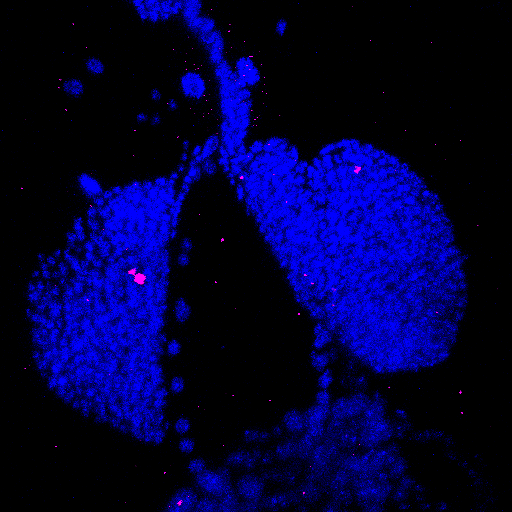

Supplement: Supplementary file 32 — Source data Fig. 7 [file 44318_2025_540_MOESM32_ESM.zip › SD Figure 7/7I/Microscopy Control H3P-DAPI.tif]

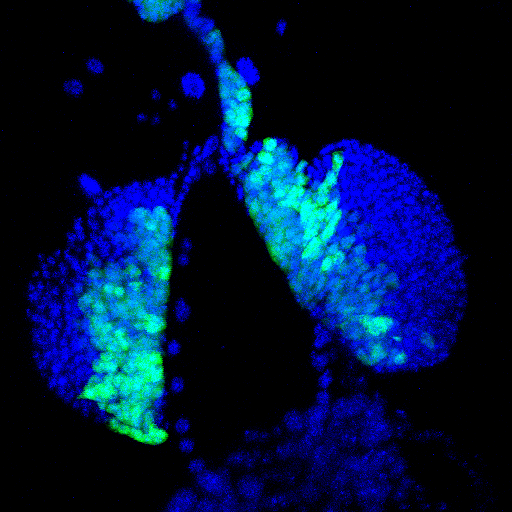

Supplement: Supplementary file 32 — Source data Fig. 7 [file 44318_2025_540_MOESM32_ESM.zip › SD Figure 7/7I/Microscopy Control_tep4-GFP_DAPI.tif]

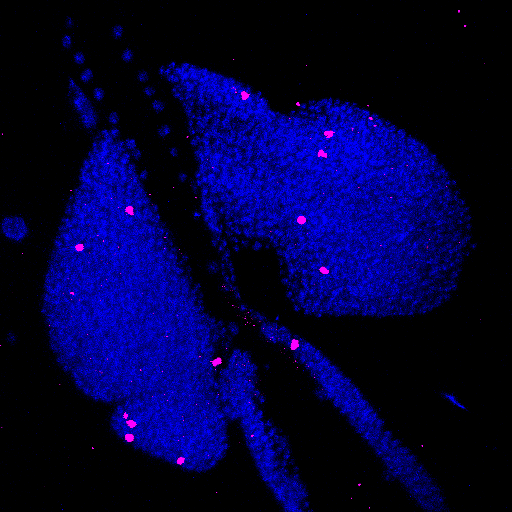

Supplement: Supplementary file 32 — Source data Fig. 7 [file 44318_2025_540_MOESM32_ESM.zip › SD Figure 7/7I/Microscopy LLOMe 8mM_H3P DAPI.tif]

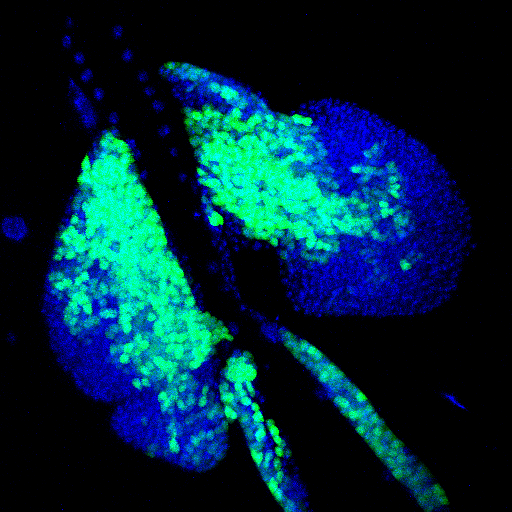

Supplement: Supplementary file 32 — Source data Fig. 7 [file 44318_2025_540_MOESM32_ESM.zip › SD Figure 7/7I/Microscopy LLOMe8mM_tep4-GFP DAPI.tif]

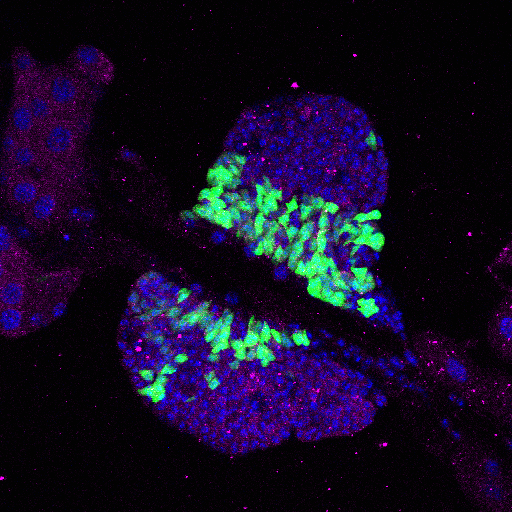

Supplement: Supplementary file 32 — Source data Fig. 7 [file 44318_2025_540_MOESM32_ESM.zip › SD Figure 7/7L/Microscopy Control_DAPI_Relish_tep4GFP.tif]

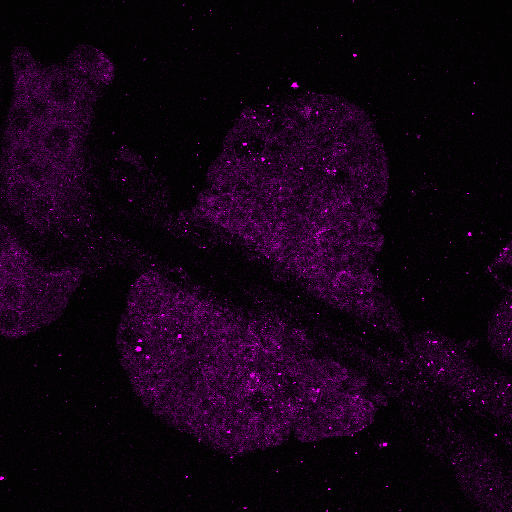

Supplement: Supplementary file 32 — Source data Fig. 7 [file 44318_2025_540_MOESM32_ESM.zip › SD Figure 7/7L/Microscopy Control_Relish.tif]

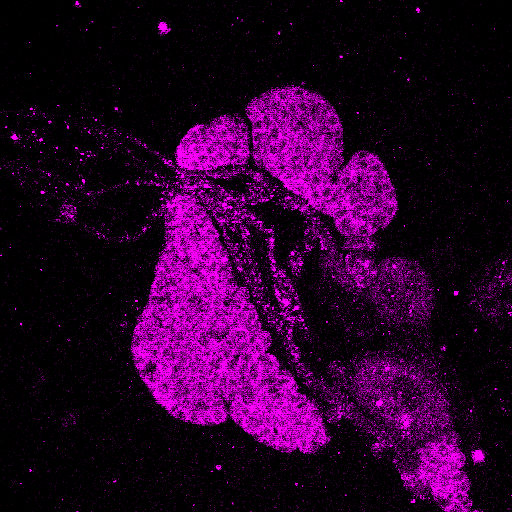

Supplement: Supplementary file 32 — Source data Fig. 7 [file 44318_2025_540_MOESM32_ESM.zip › SD Figure 7/7L/Microscopy LLOMe_Relish.tif]

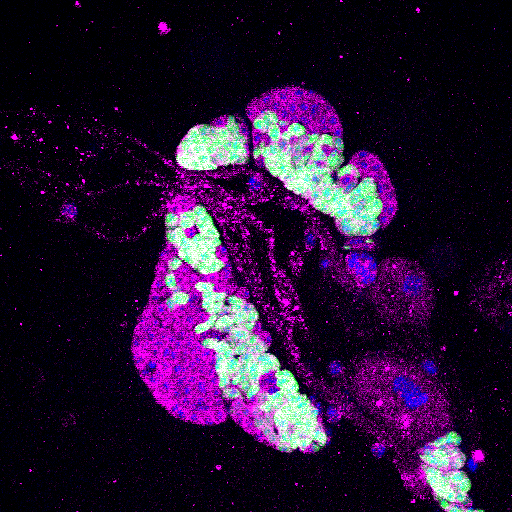

Supplement: Supplementary file 32 — Source data Fig. 7 [file 44318_2025_540_MOESM32_ESM.zip › SD Figure 7/7L/Microscopy LLOMe_Relish_DAPI_tep4GFP.tif]

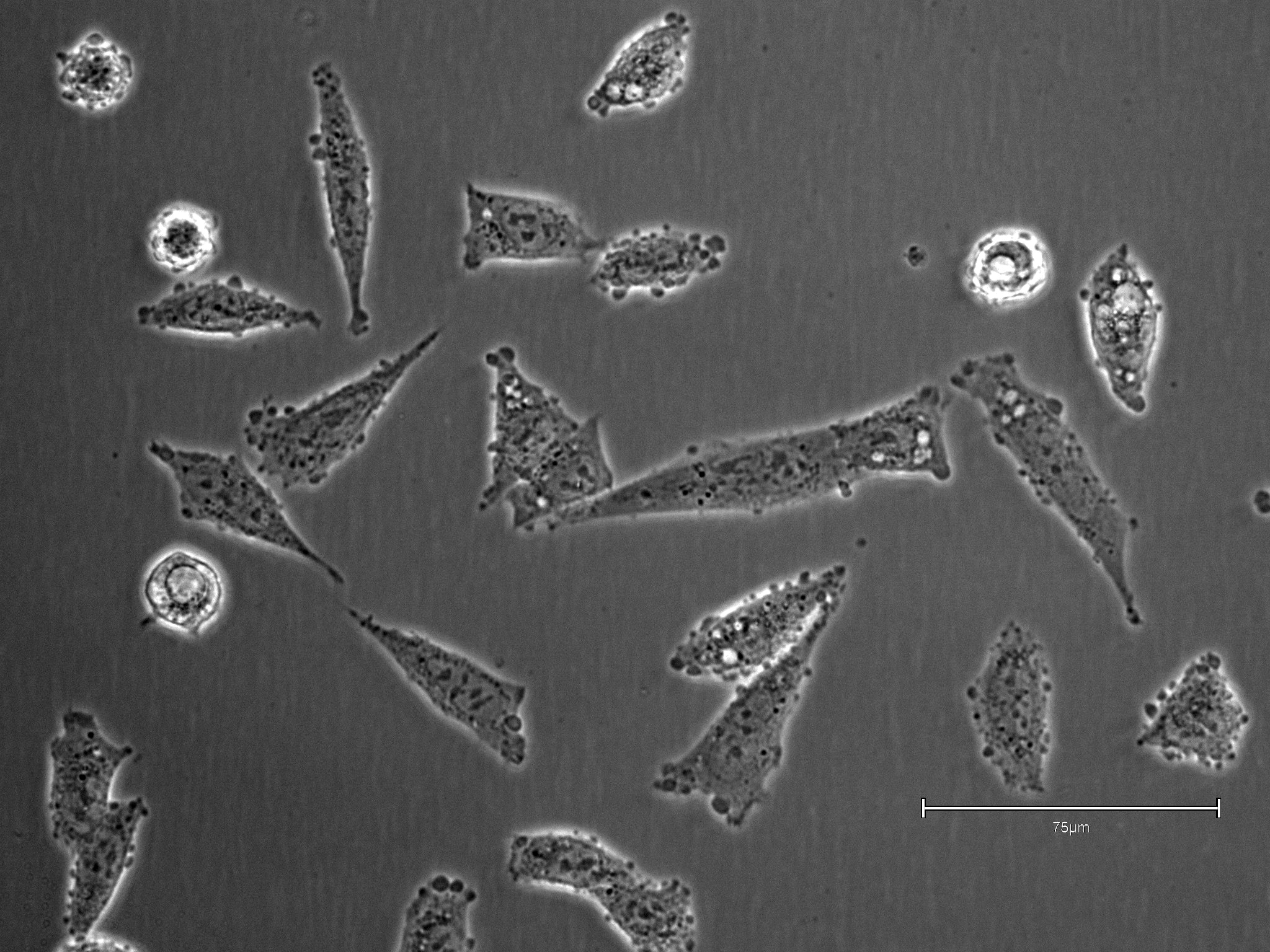

Supplement: Supplementary file 33 — Source data EV and Appendix [file 44318_2025_540_MOESM33_ESM.zip › Source data EV and Appendix/Appendix Figure S1/40uM/12H.jpg]

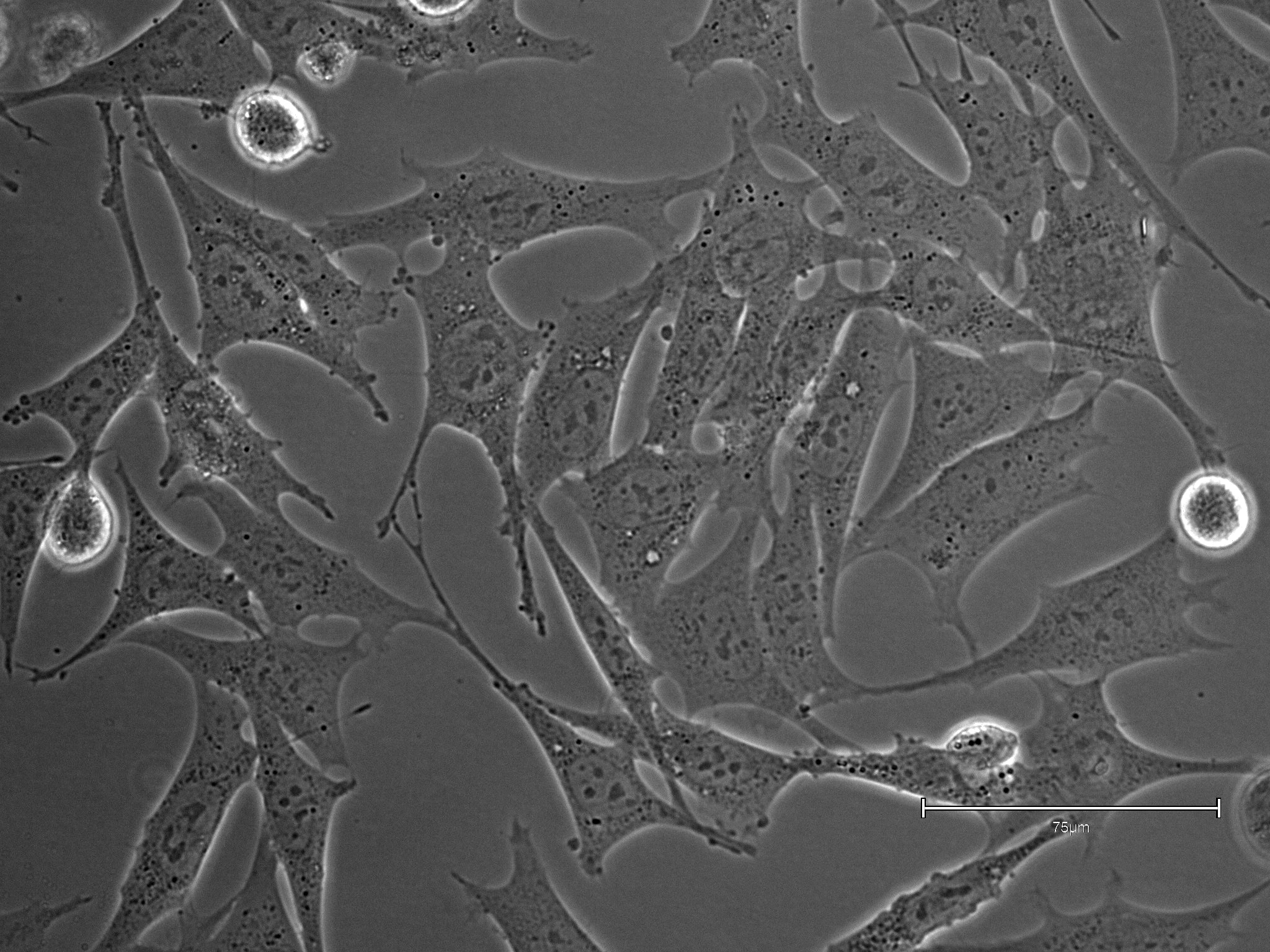

Supplement: Supplementary file 33 — Source data EV and Appendix [file 44318_2025_540_MOESM33_ESM.zip › Source data EV and Appendix/Appendix Figure S1/40uM/24H.jpg]

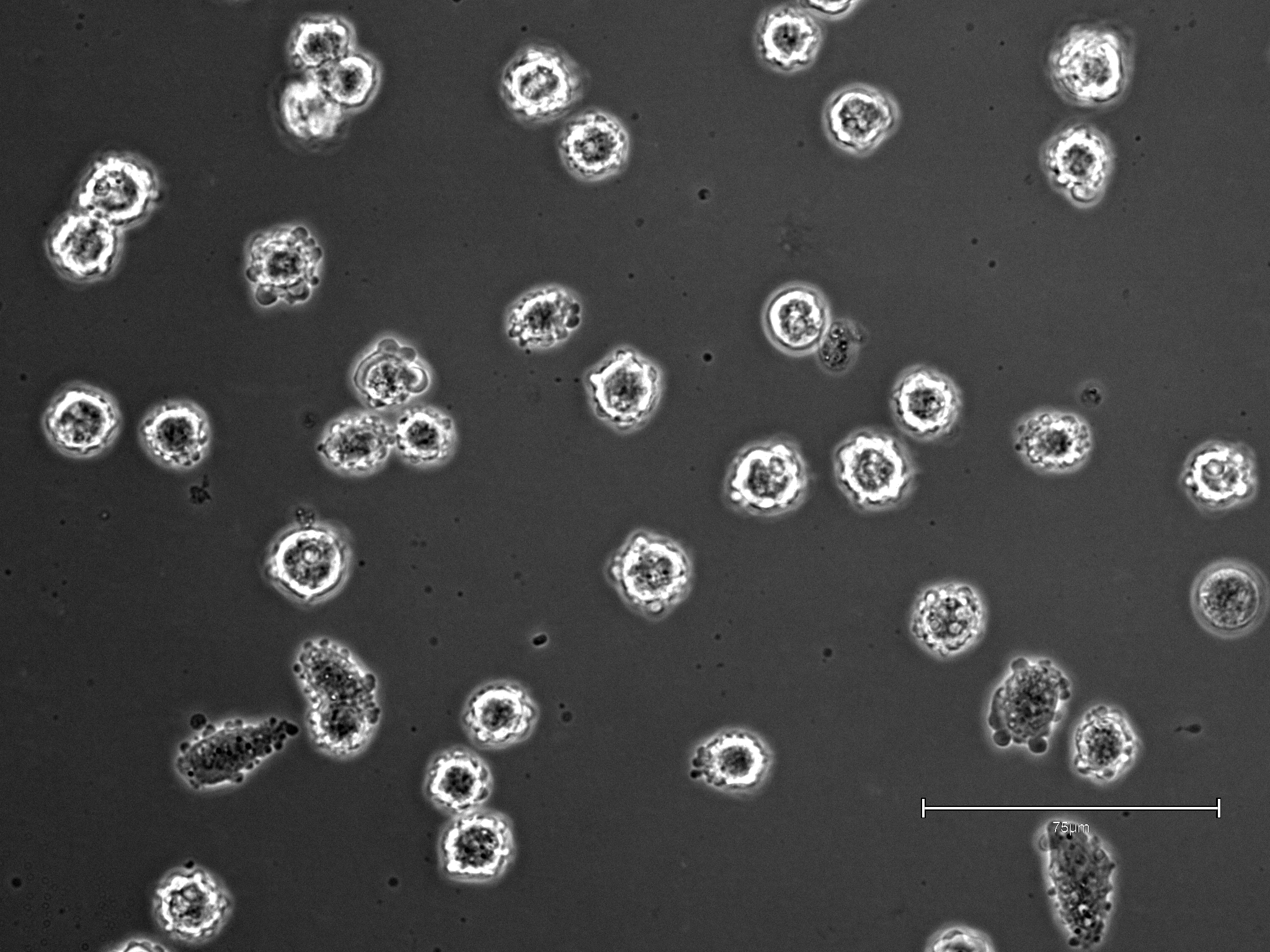

Supplement: Supplementary file 33 — Source data EV and Appendix [file 44318_2025_540_MOESM33_ESM.zip › Source data EV and Appendix/Appendix Figure S1/40uM/3H.tif]

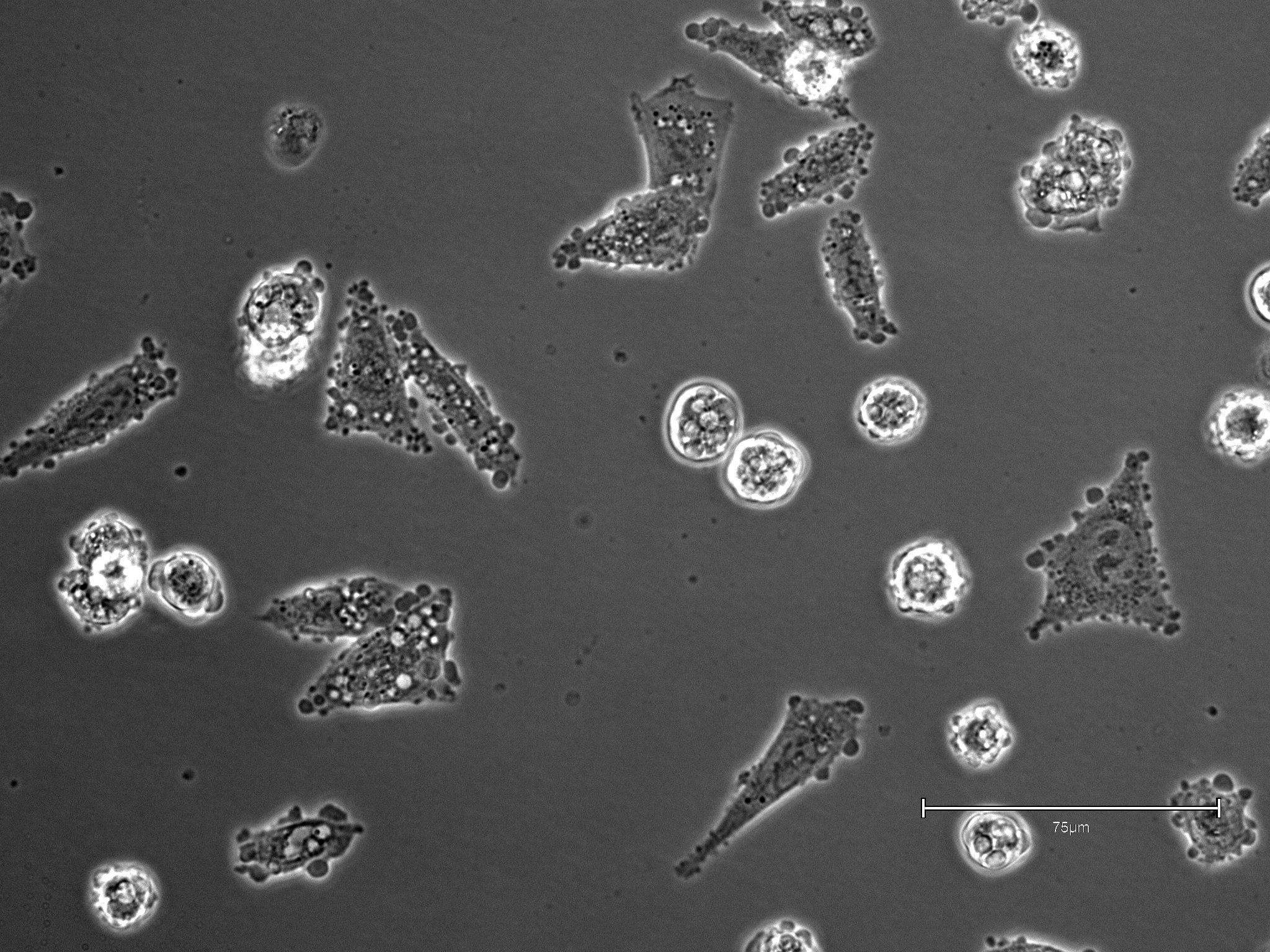

Supplement: Supplementary file 33 — Source data EV and Appendix [file 44318_2025_540_MOESM33_ESM.zip › Source data EV and Appendix/Appendix Figure S1/40uM/6H.jpg]

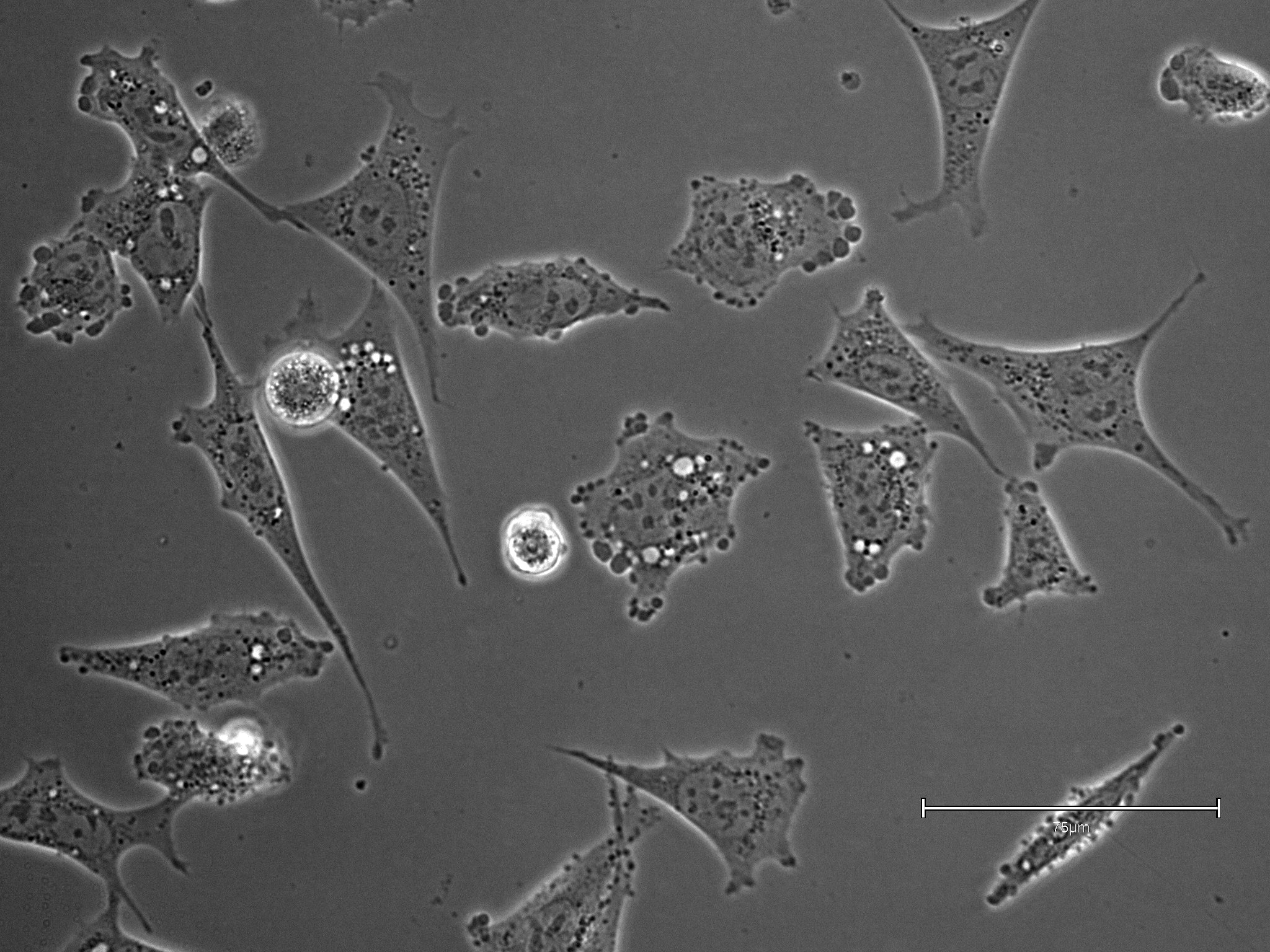

Supplement: Supplementary file 33 — Source data EV and Appendix [file 44318_2025_540_MOESM33_ESM.zip › Source data EV and Appendix/Appendix Figure S1/80uM/12H.jpg]

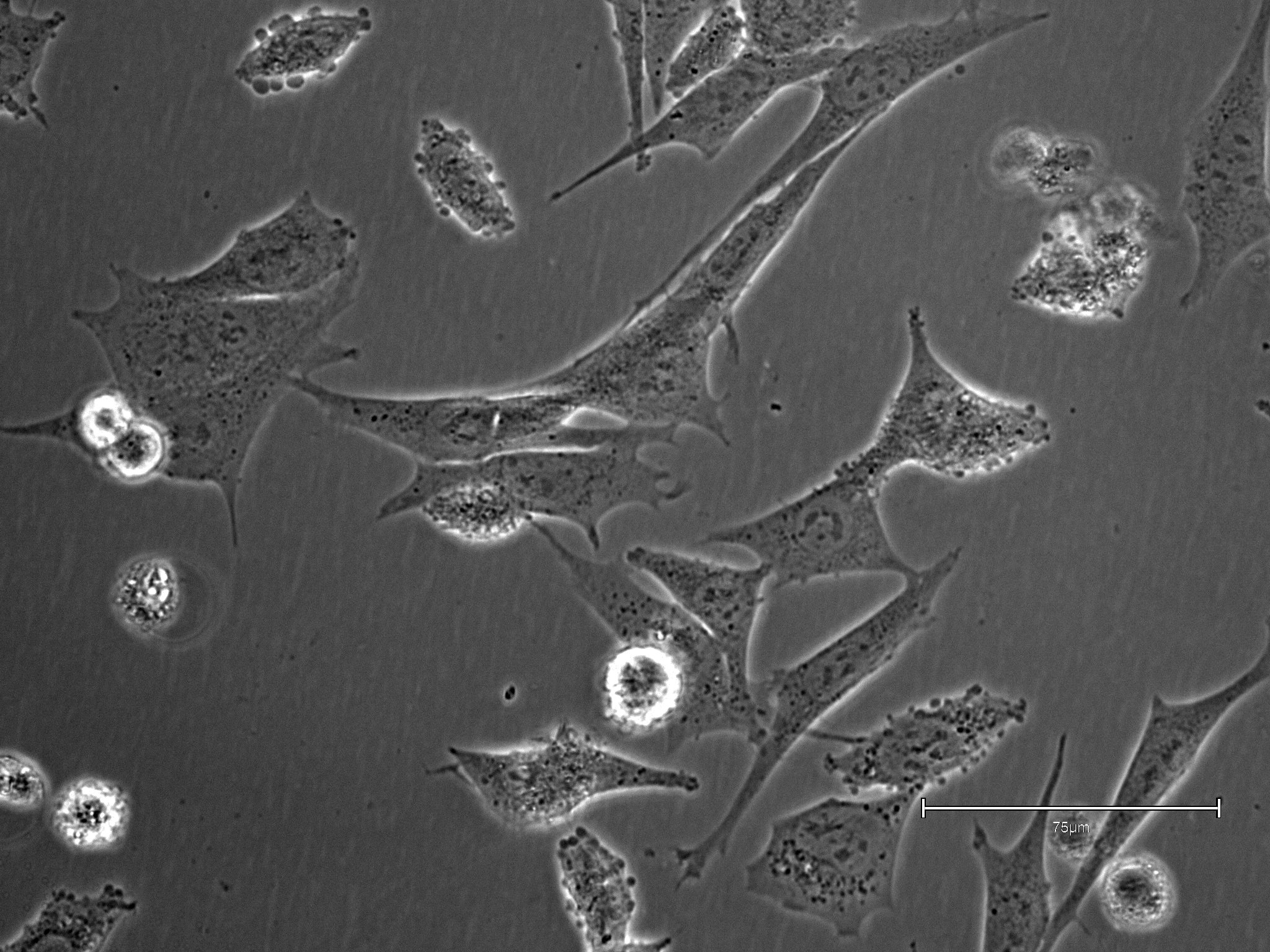

Supplement: Supplementary file 33 — Source data EV and Appendix [file 44318_2025_540_MOESM33_ESM.zip › Source data EV and Appendix/Appendix Figure S1/80uM/24H.jpg]

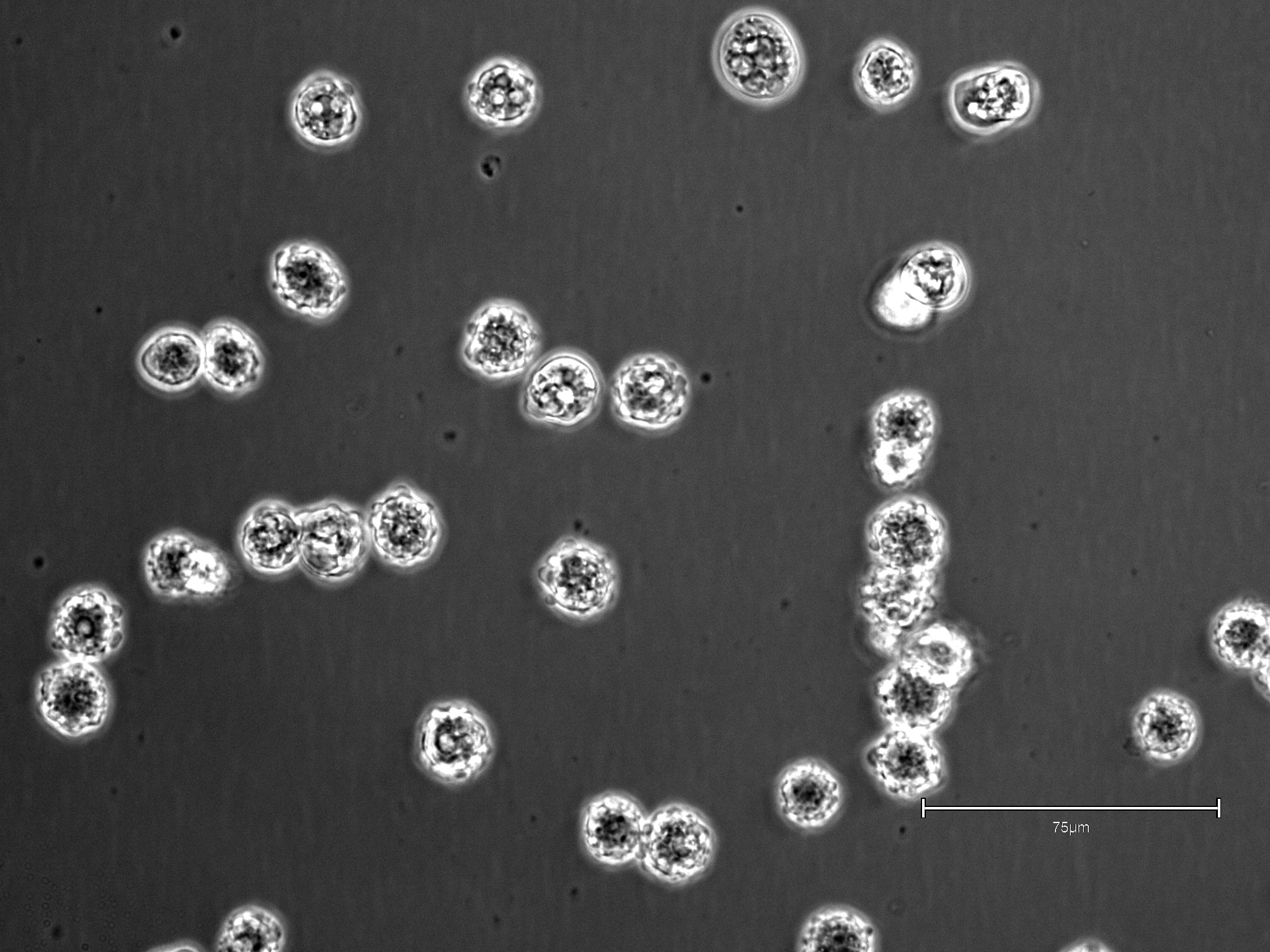

Supplement: Supplementary file 33 — Source data EV and Appendix [file 44318_2025_540_MOESM33_ESM.zip › Source data EV and Appendix/Appendix Figure S1/80uM/3H.tif]

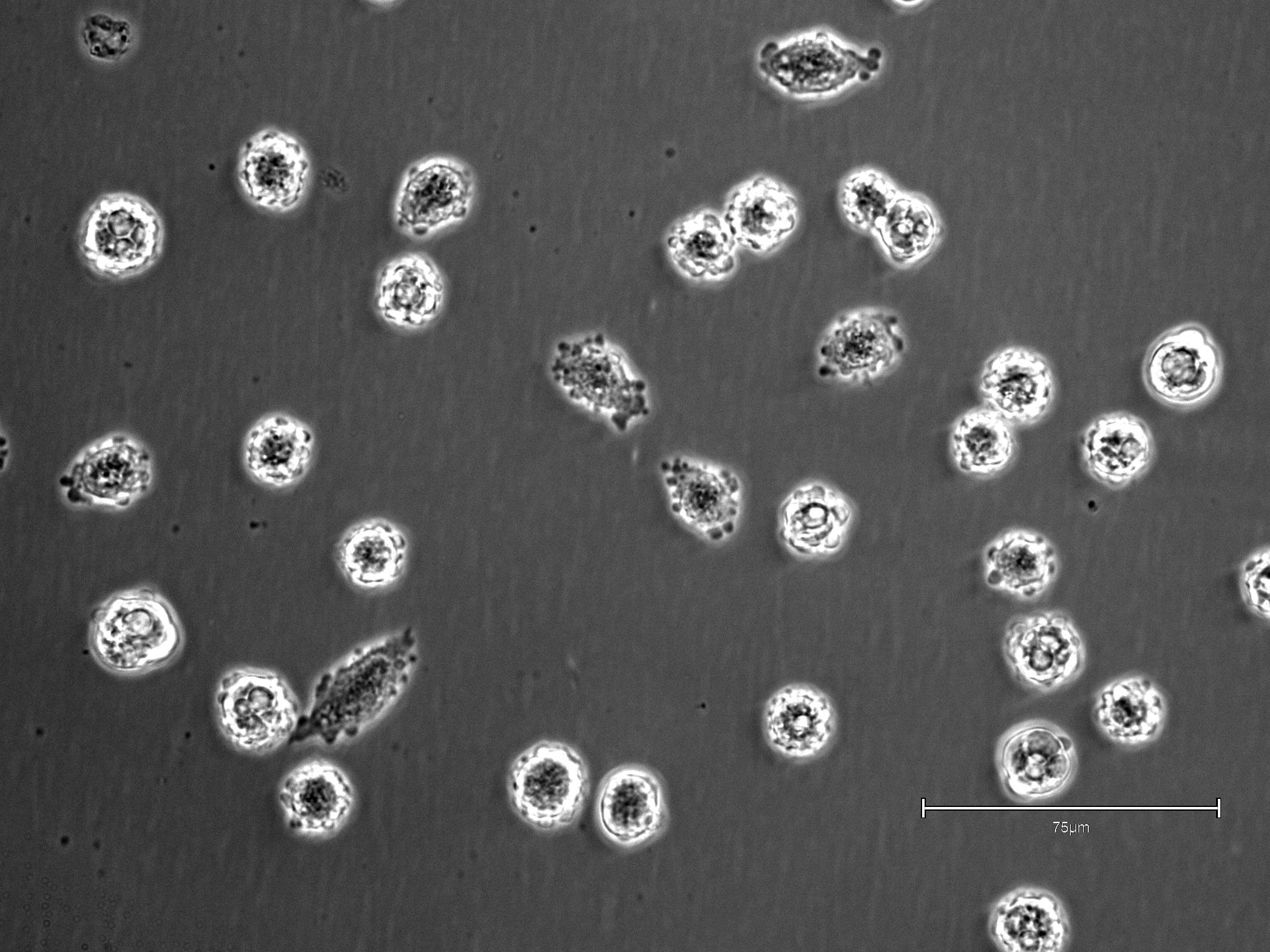

Supplement: Supplementary file 33 — Source data EV and Appendix [file 44318_2025_540_MOESM33_ESM.zip › Source data EV and Appendix/Appendix Figure S1/80uM/6H.tif]

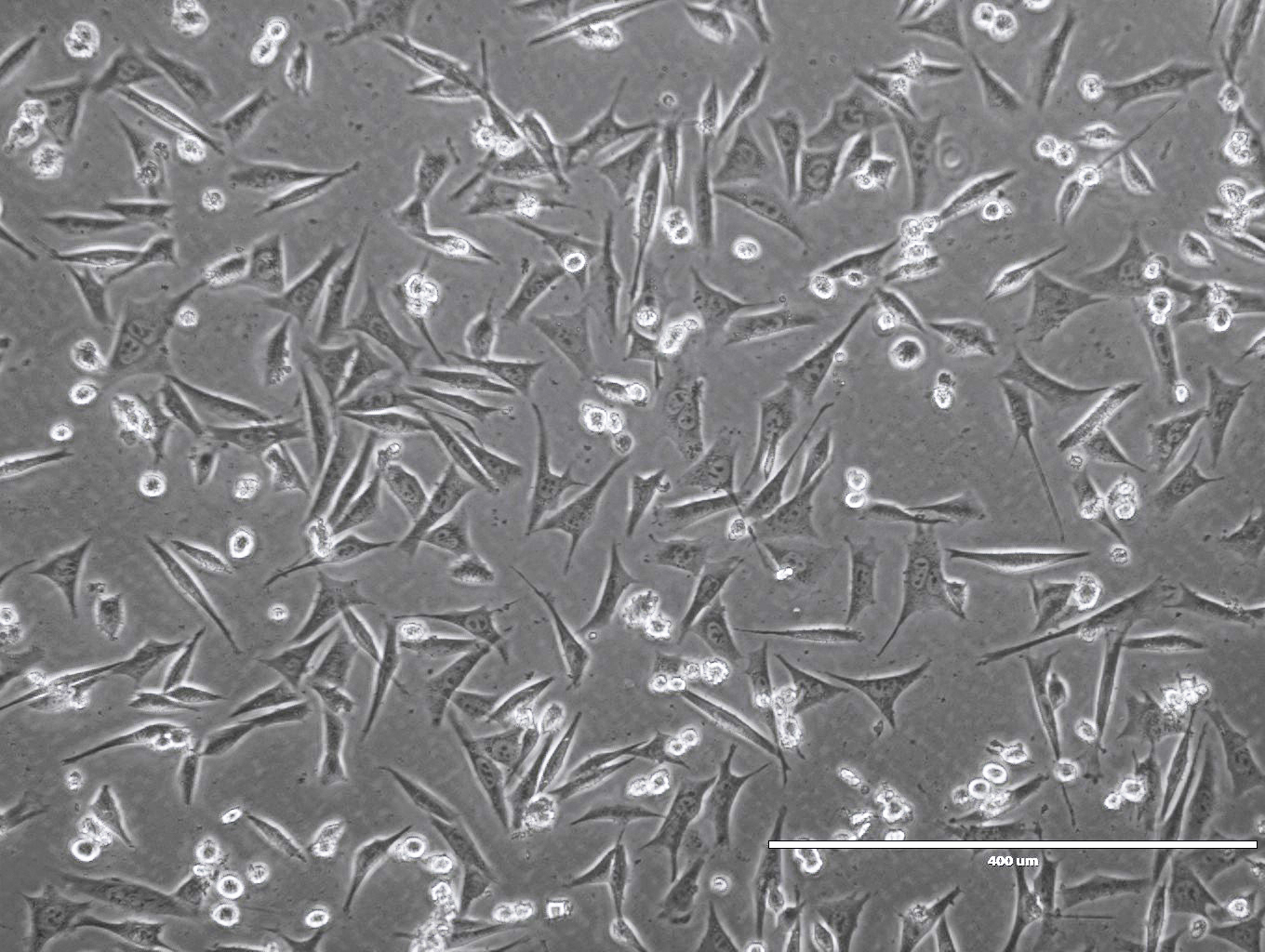

Supplement: Supplementary file 33 — Source data EV and Appendix [file 44318_2025_540_MOESM33_ESM.zip › Source data EV and Appendix/Appendix Figure S5/C/ULK1 KO/ULK1 KO 24 h.jpg]

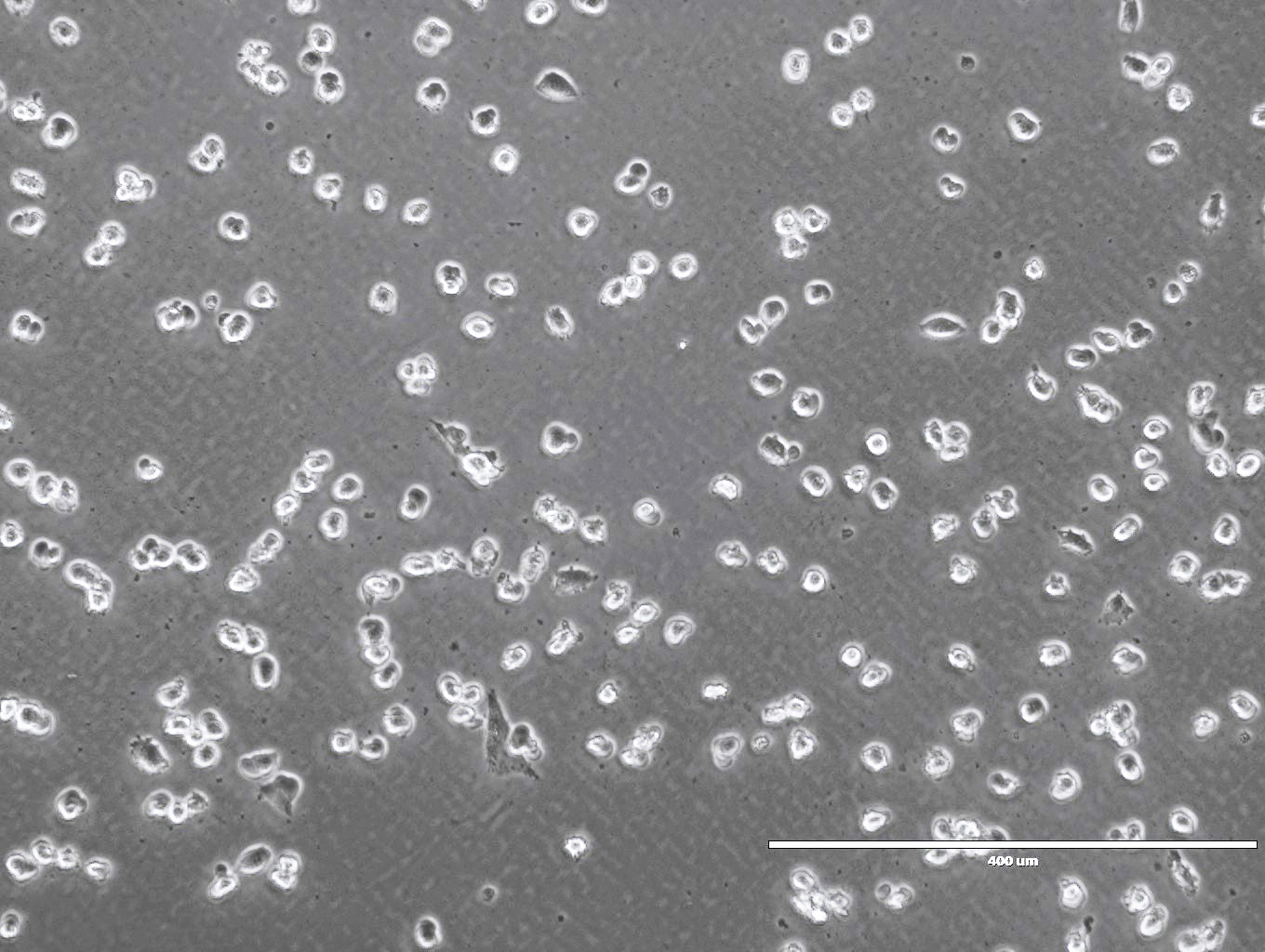

Supplement: Supplementary file 33 — Source data EV and Appendix [file 44318_2025_540_MOESM33_ESM.zip › Source data EV and Appendix/Appendix Figure S5/C/ULK1 KO/ULK1 KO 30 min.jpg]

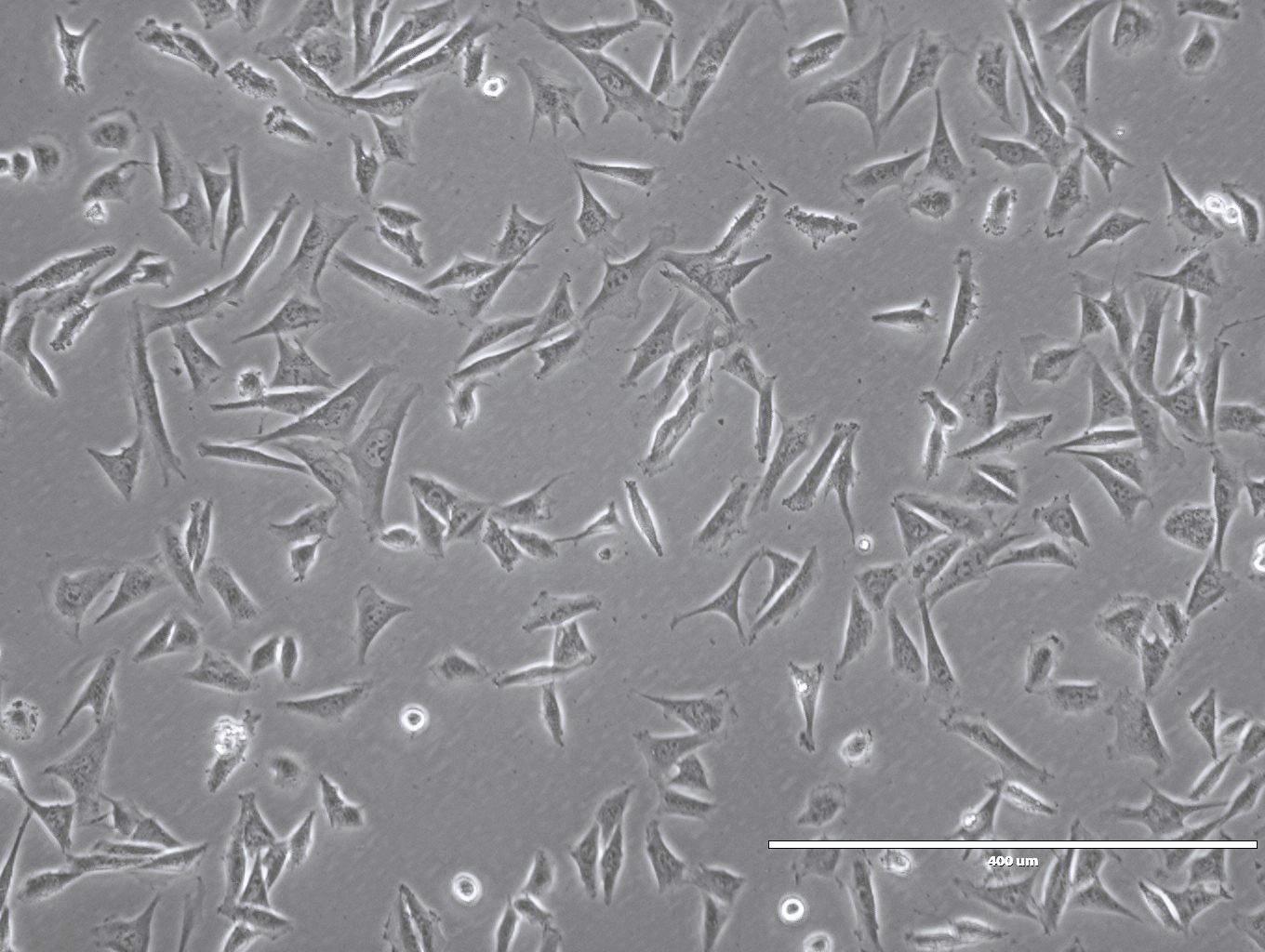

Supplement: Supplementary file 33 — Source data EV and Appendix [file 44318_2025_540_MOESM33_ESM.zip › Source data EV and Appendix/Appendix Figure S5/C/ULK1 KO/ULK1 KO Control.jpg]

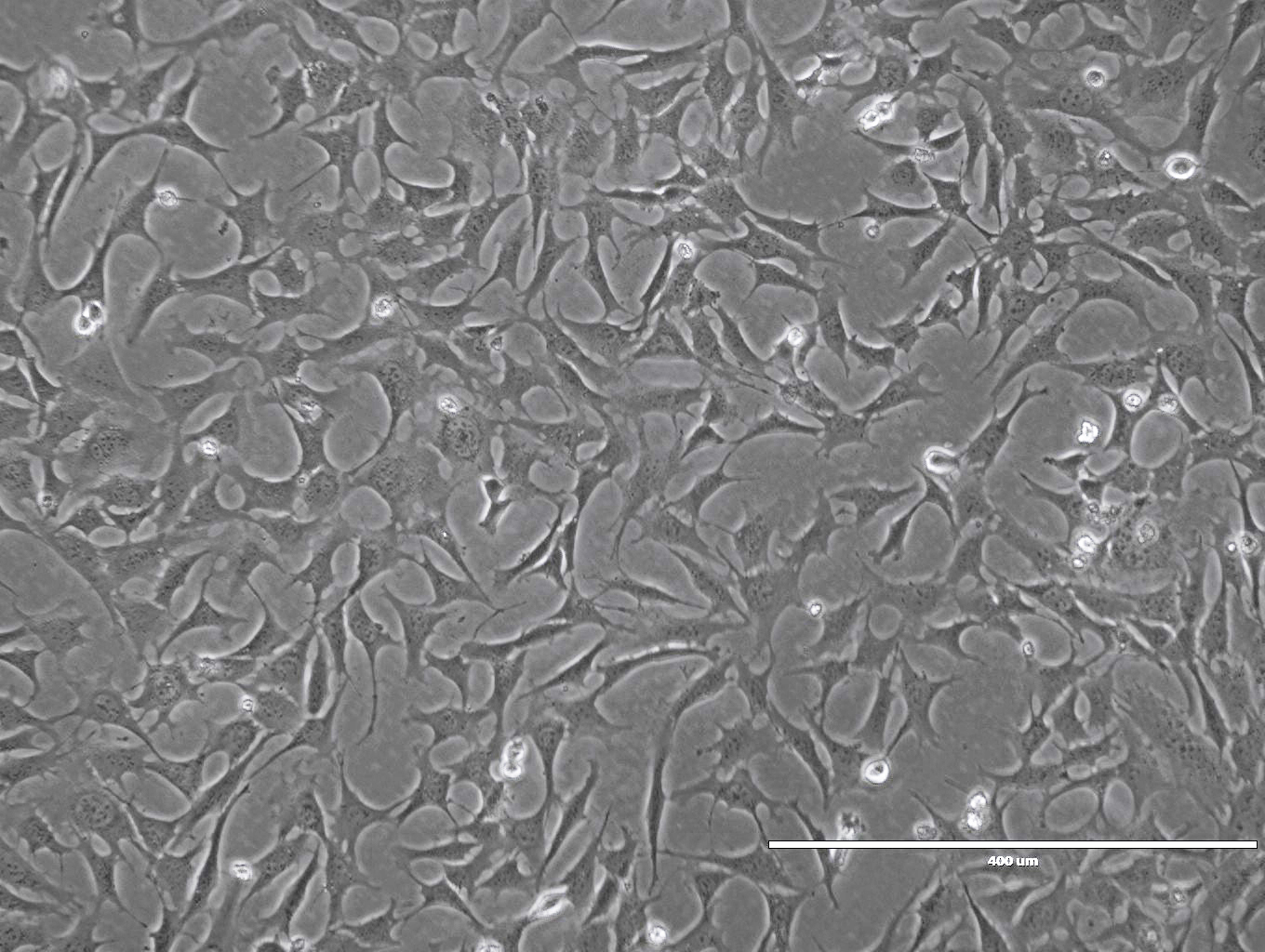

Supplement: Supplementary file 33 — Source data EV and Appendix [file 44318_2025_540_MOESM33_ESM.zip › Source data EV and Appendix/Appendix Figure S5/C/ULK1 WT/ULK1 WT 24 h.jpg]

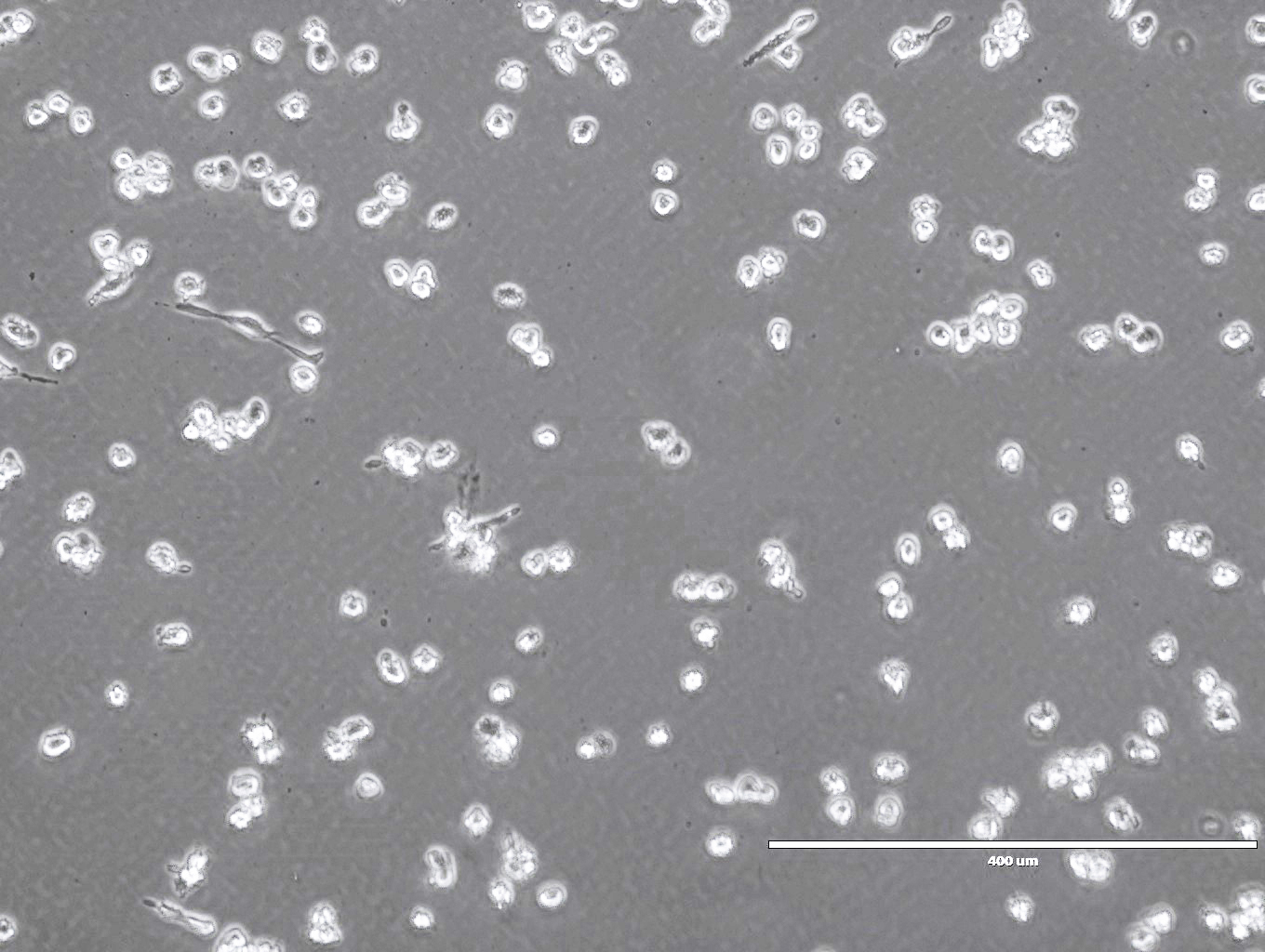

Supplement: Supplementary file 33 — Source data EV and Appendix [file 44318_2025_540_MOESM33_ESM.zip › Source data EV and Appendix/Appendix Figure S5/C/ULK1 WT/ULK1 WT 30 min.jpg]

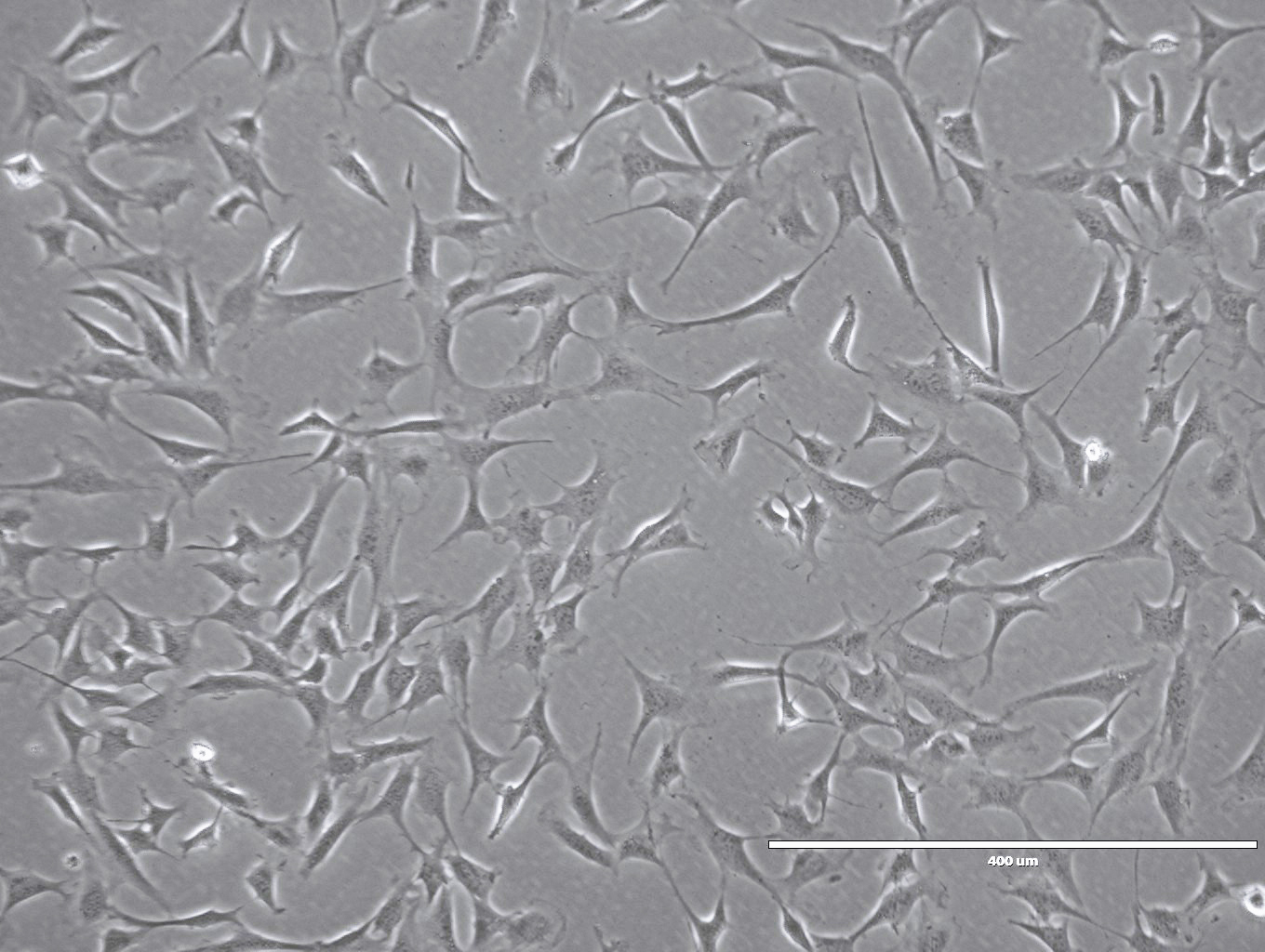

Supplement: Supplementary file 33 — Source data EV and Appendix [file 44318_2025_540_MOESM33_ESM.zip › Source data EV and Appendix/Appendix Figure S5/C/ULK1 WT/ULK1 WT Control.jpg]

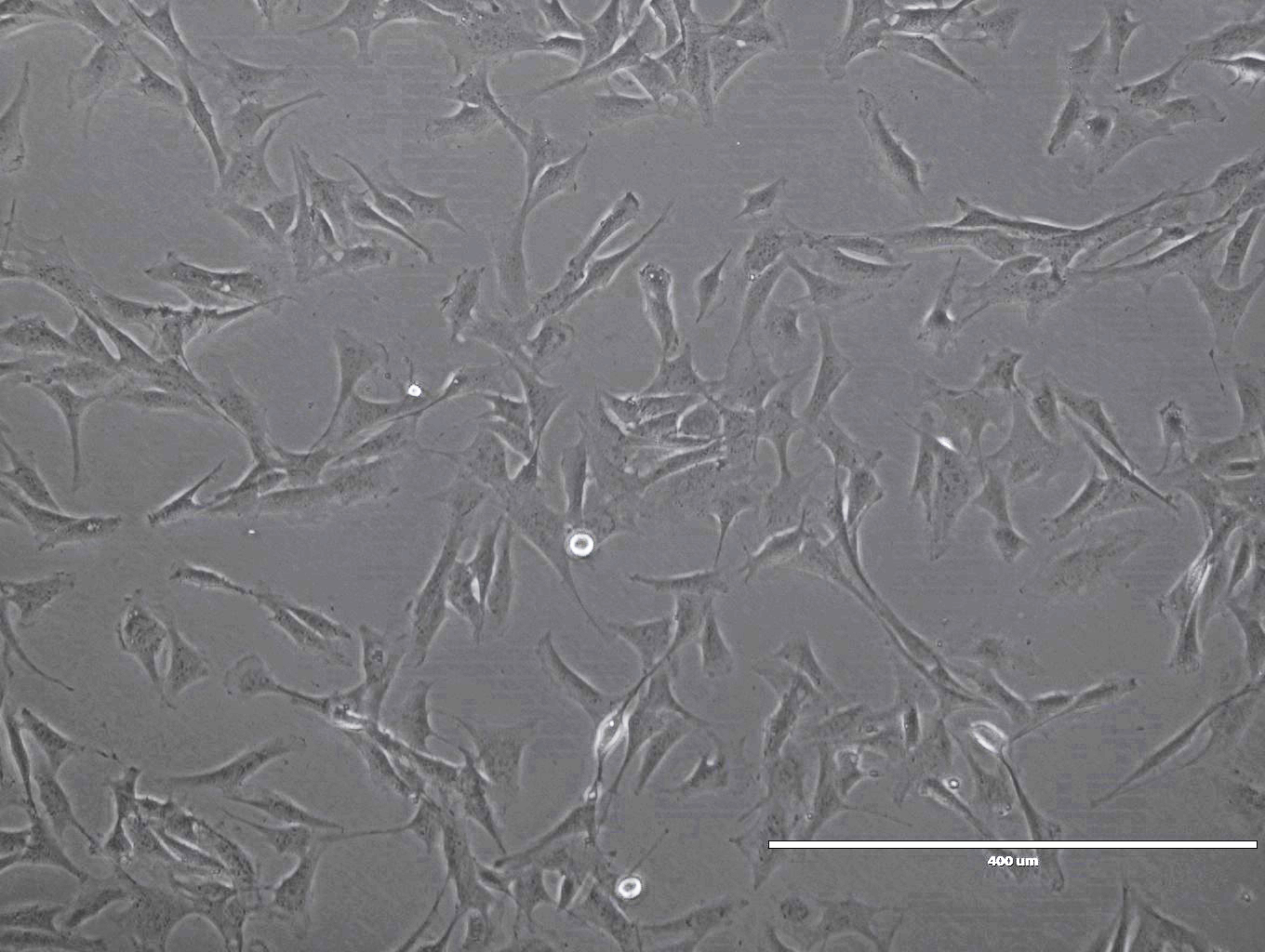

Supplement: Supplementary file 33 — Source data EV and Appendix [file 44318_2025_540_MOESM33_ESM.zip › Source data EV and Appendix/Appendix Figure S5/E/ATG5 KO/ATG5 KO Control.jpg]

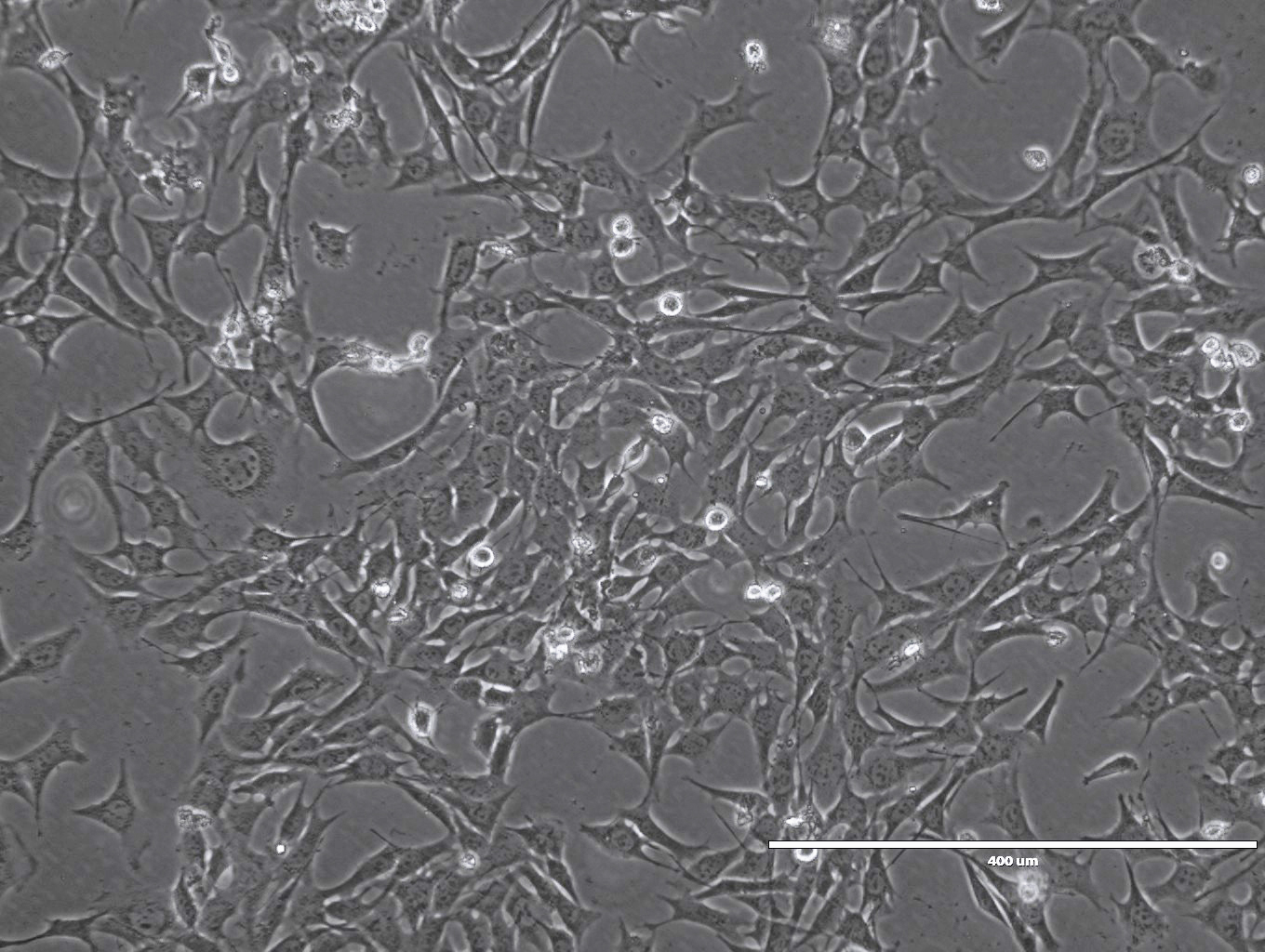

Supplement: Supplementary file 33 — Source data EV and Appendix [file 44318_2025_540_MOESM33_ESM.zip › Source data EV and Appendix/Appendix Figure S5/E/ATG5 KO/ATG5KO 24 h.jpg]

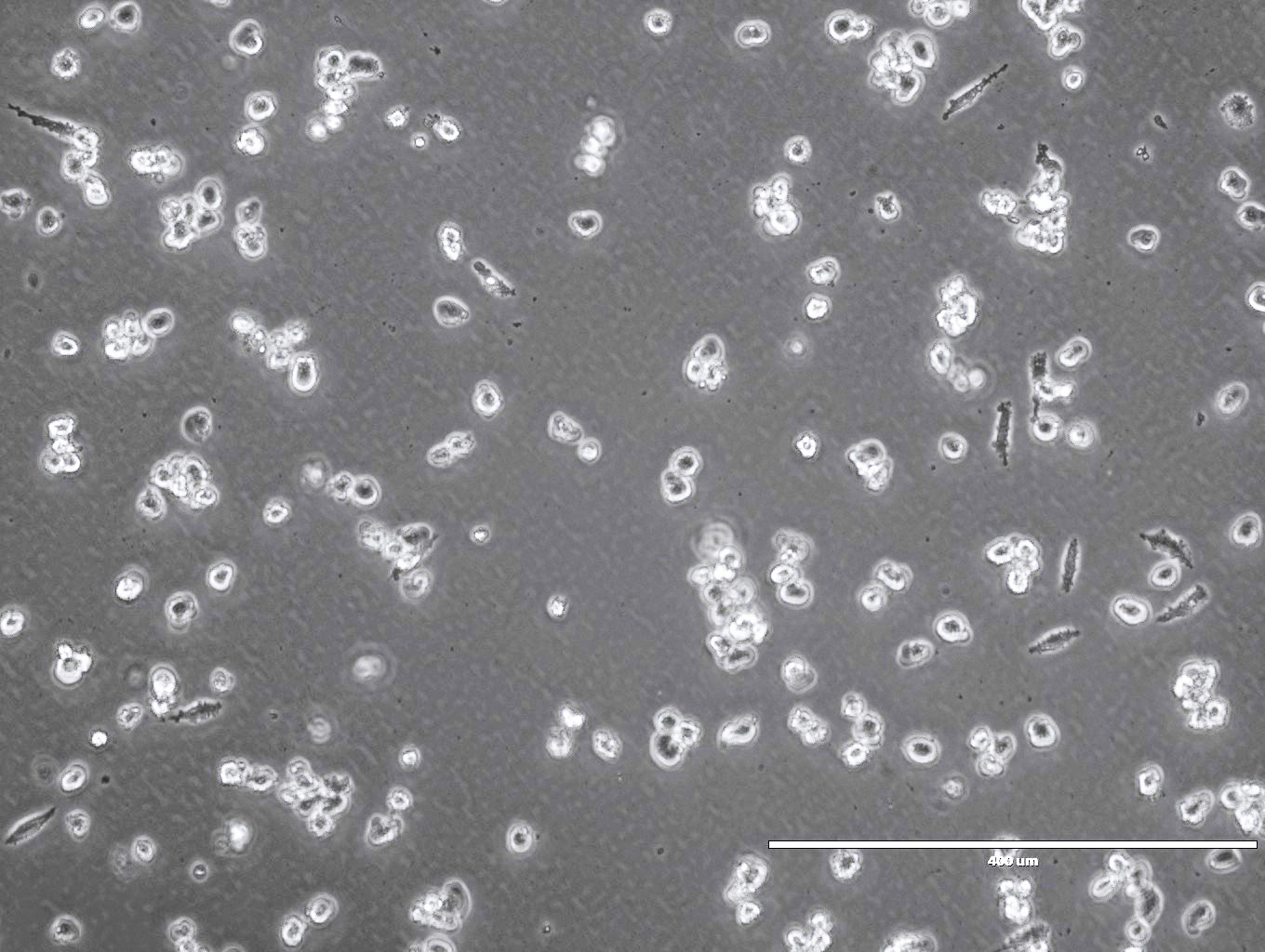

Supplement: Supplementary file 33 — Source data EV and Appendix [file 44318_2025_540_MOESM33_ESM.zip › Source data EV and Appendix/Appendix Figure S5/E/ATG5 KO/ATG5KO 30 min.jpg]

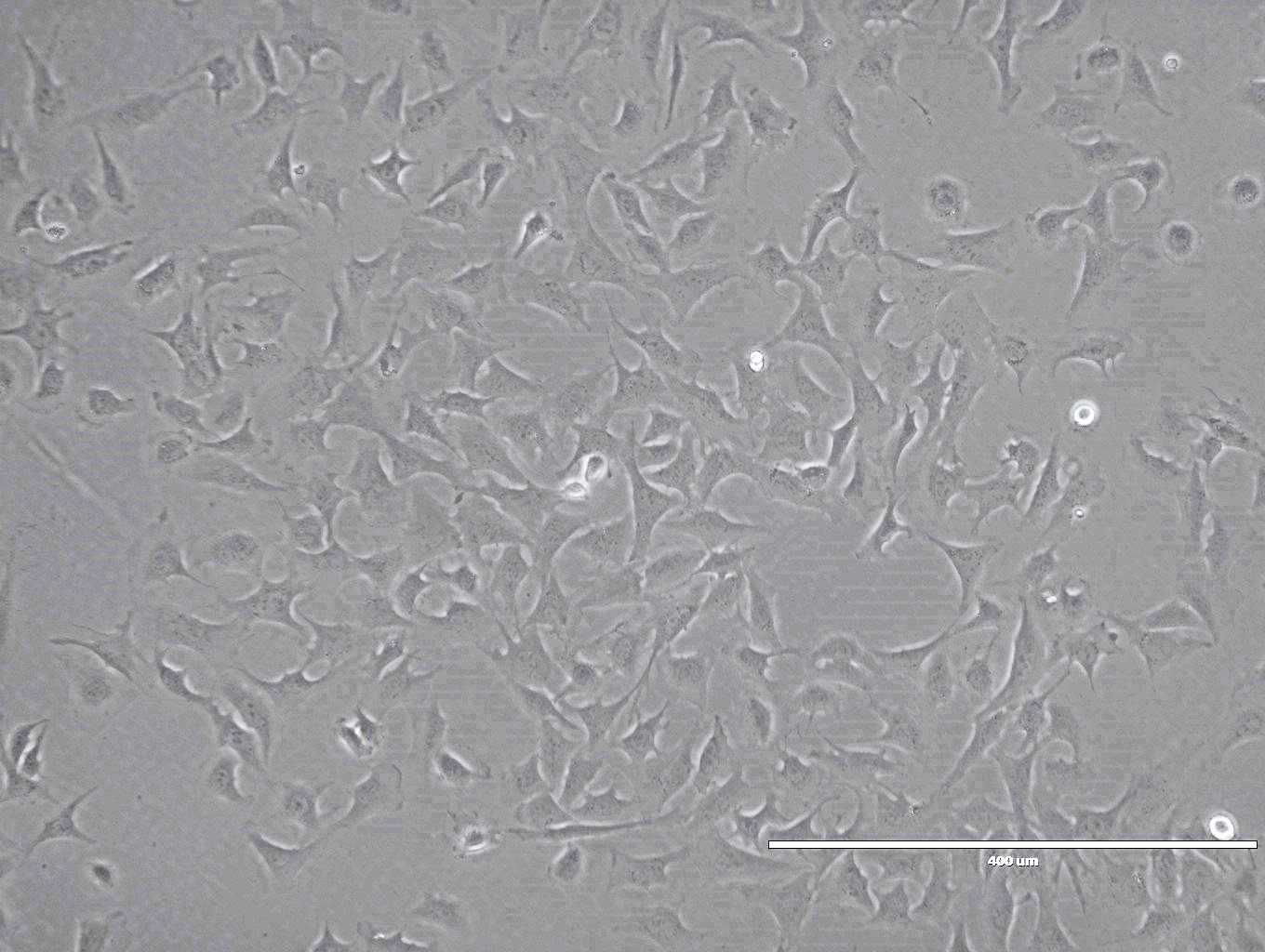

Supplement: Supplementary file 33 — Source data EV and Appendix [file 44318_2025_540_MOESM33_ESM.zip › Source data EV and Appendix/Appendix Figure S5/E/ATG5 WT/ATG 5 WT Control.jpg]

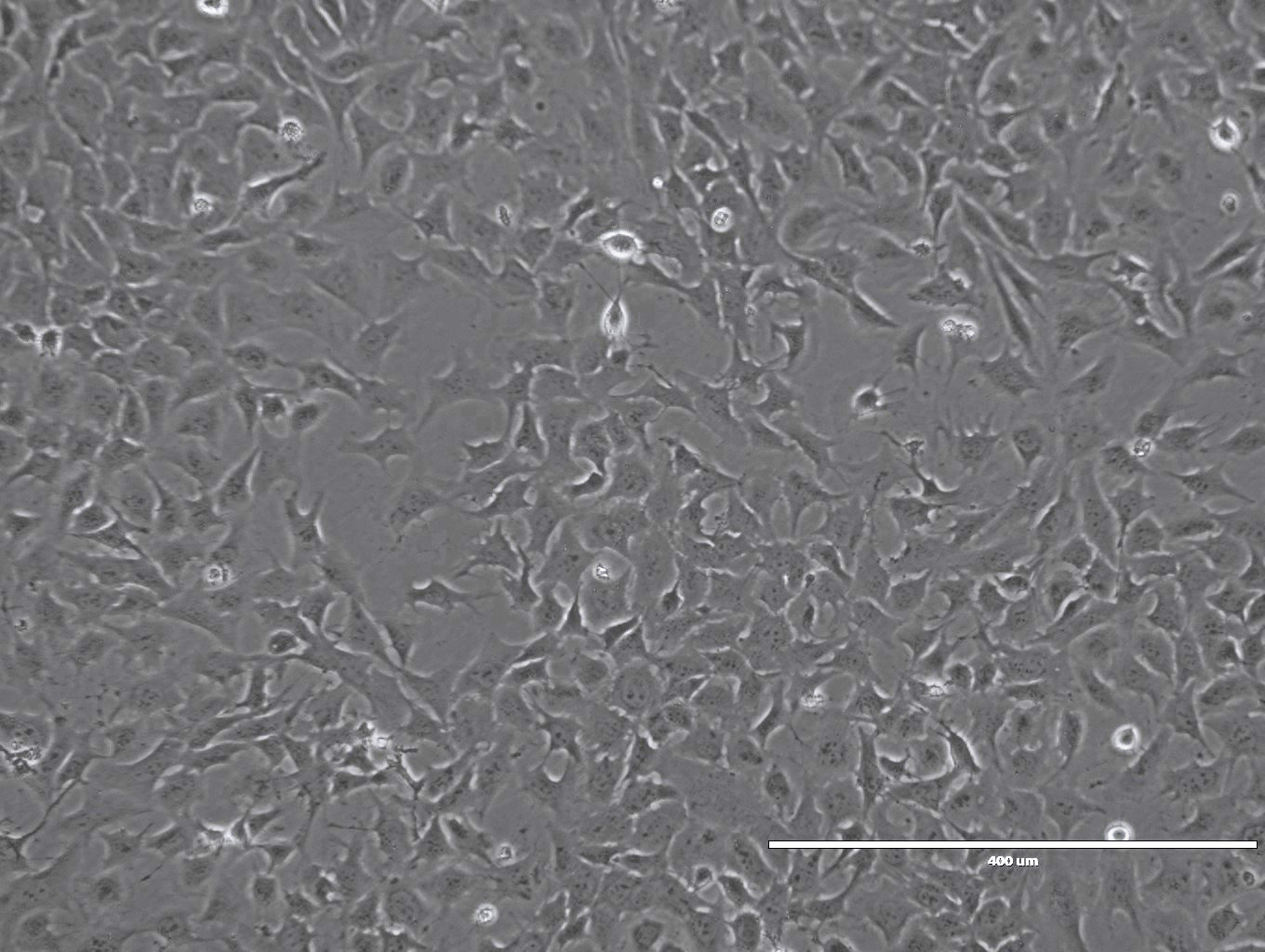

Supplement: Supplementary file 33 — Source data EV and Appendix [file 44318_2025_540_MOESM33_ESM.zip › Source data EV and Appendix/Appendix Figure S5/E/ATG5 WT/ATG5 WT 24h.jpg]

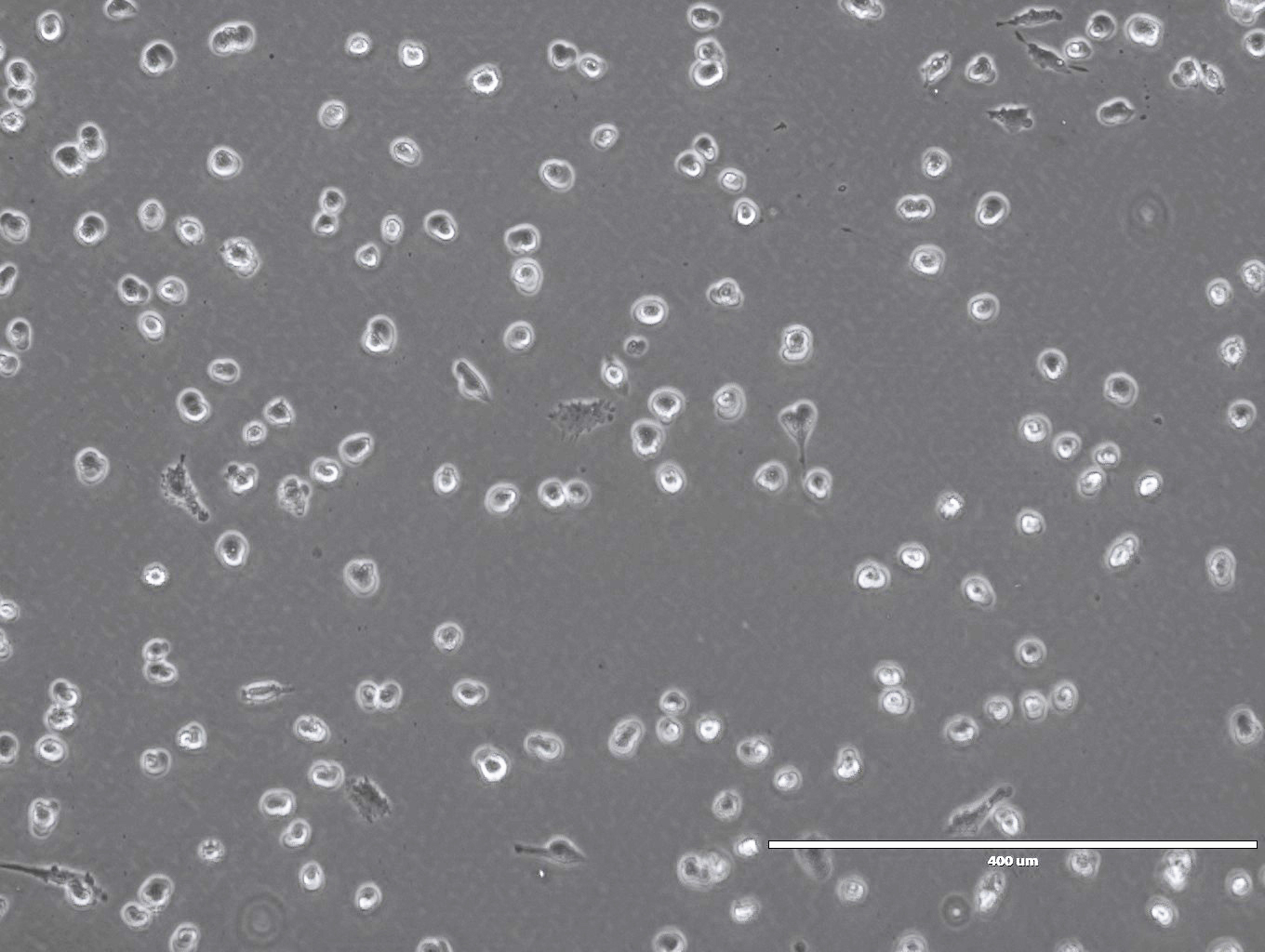

Supplement: Supplementary file 33 — Source data EV and Appendix [file 44318_2025_540_MOESM33_ESM.zip › Source data EV and Appendix/Appendix Figure S5/E/ATG5 WT/ATG5 WT 30 min.jpg]

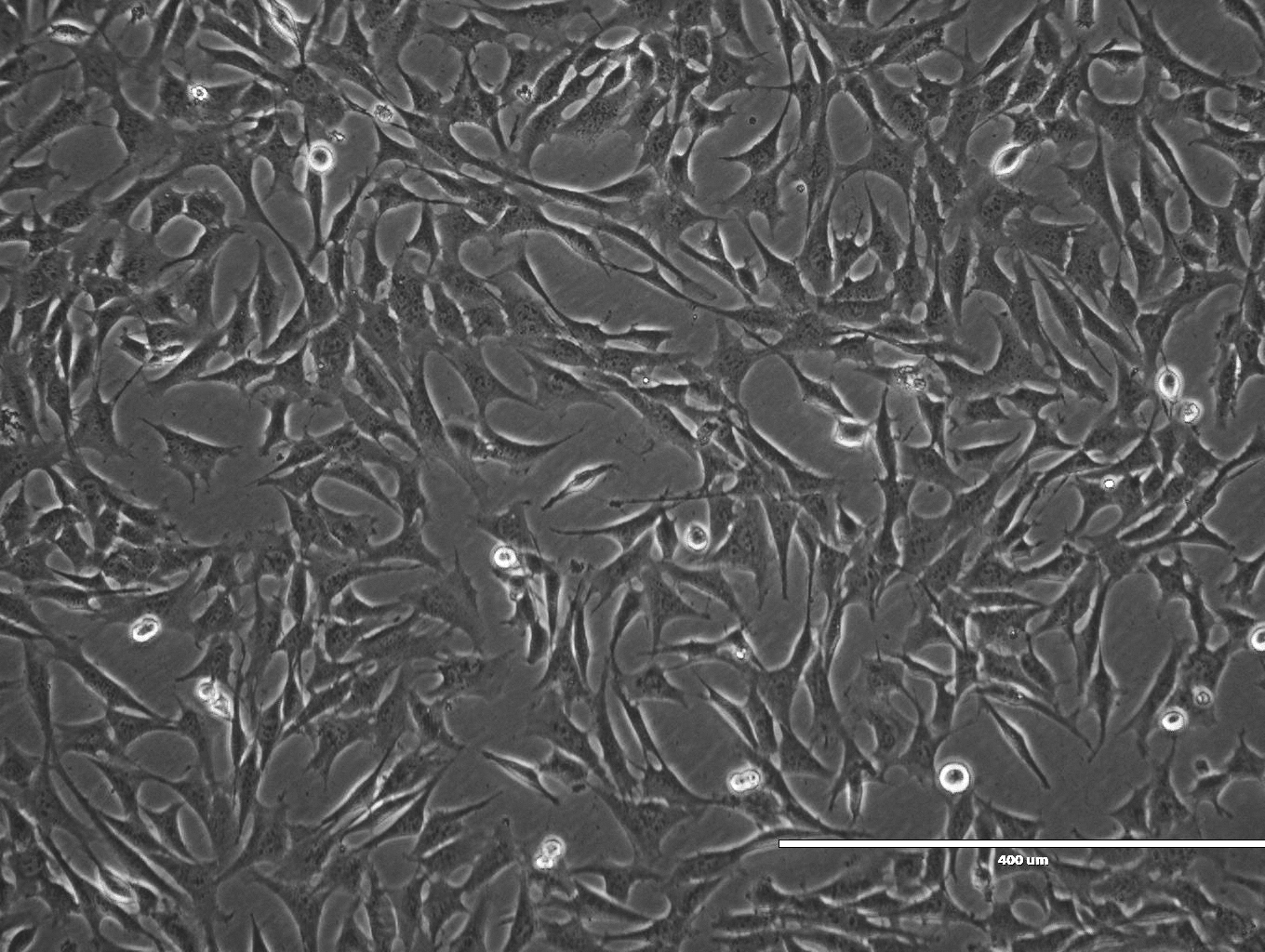

Supplement: Supplementary file 33 — Source data EV and Appendix [file 44318_2025_540_MOESM33_ESM.zip › Source data EV and Appendix/Figure EV 1/1A/MEF_0 h (1).jpg]

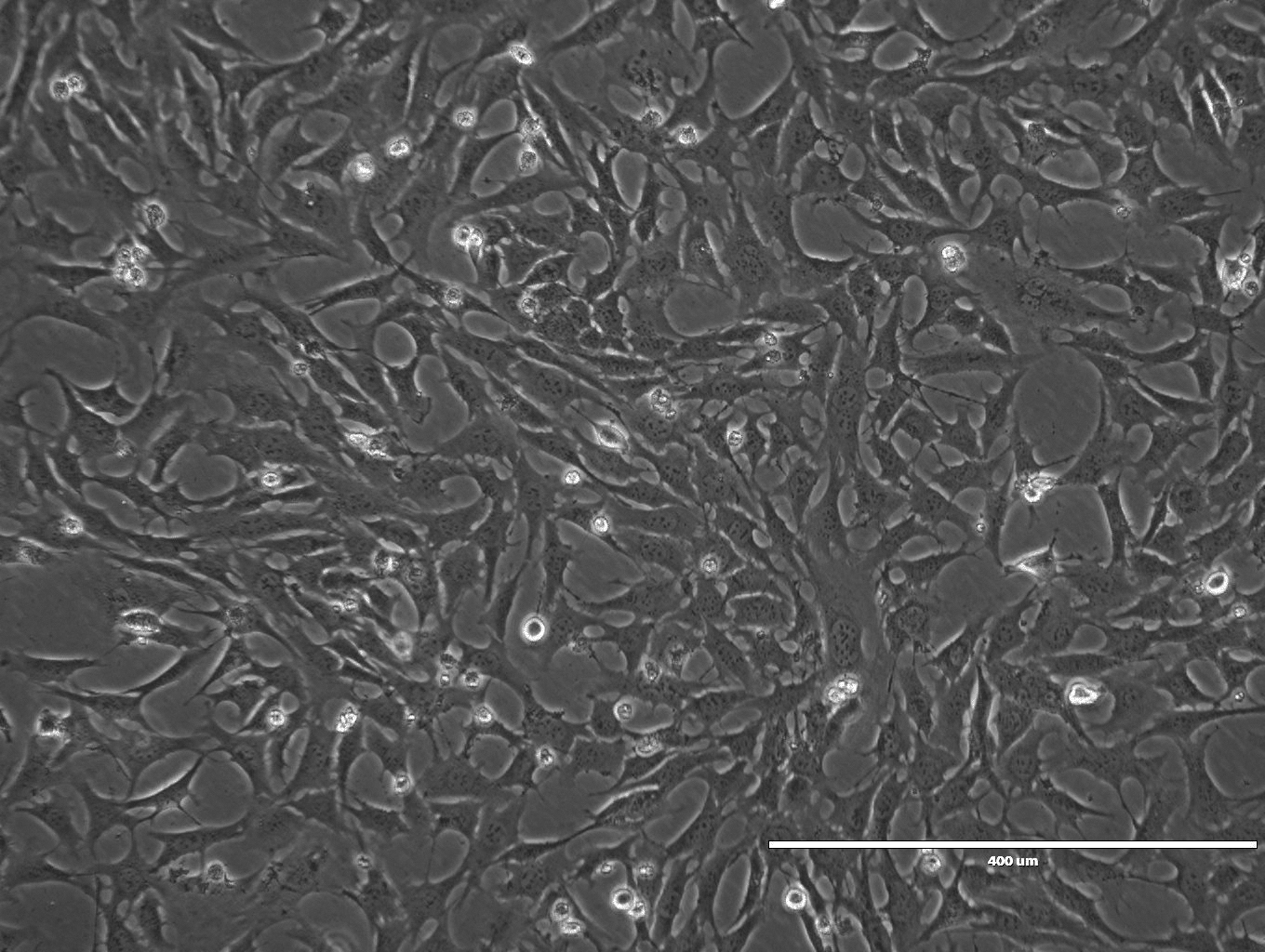

Supplement: Supplementary file 33 — Source data EV and Appendix [file 44318_2025_540_MOESM33_ESM.zip › Source data EV and Appendix/Figure EV 1/1A/MEF_24 h (1).jpg]

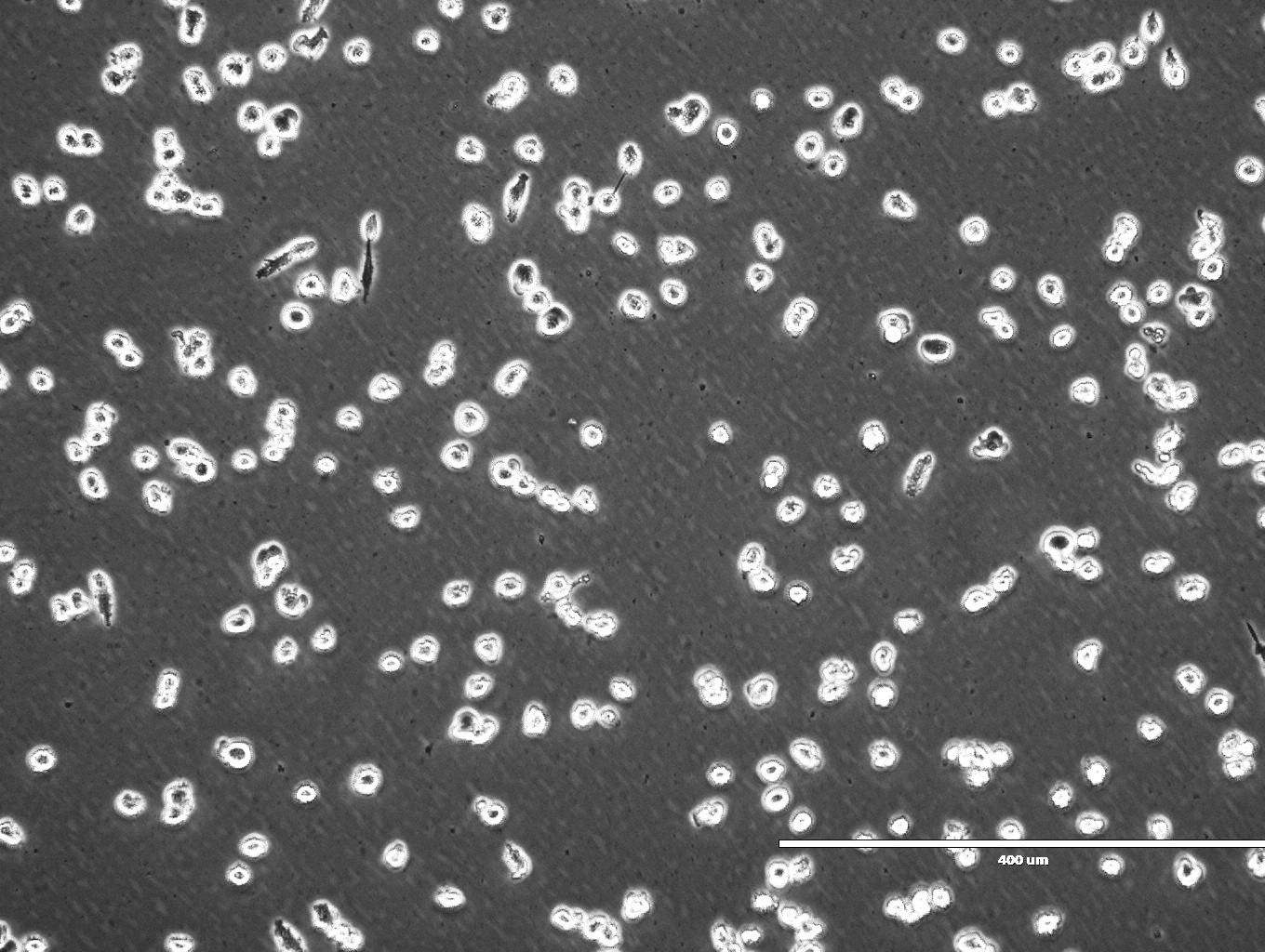

Supplement: Supplementary file 33 — Source data EV and Appendix [file 44318_2025_540_MOESM33_ESM.zip › Source data EV and Appendix/Figure EV 1/1A/MEF_30 min (1).jpg]

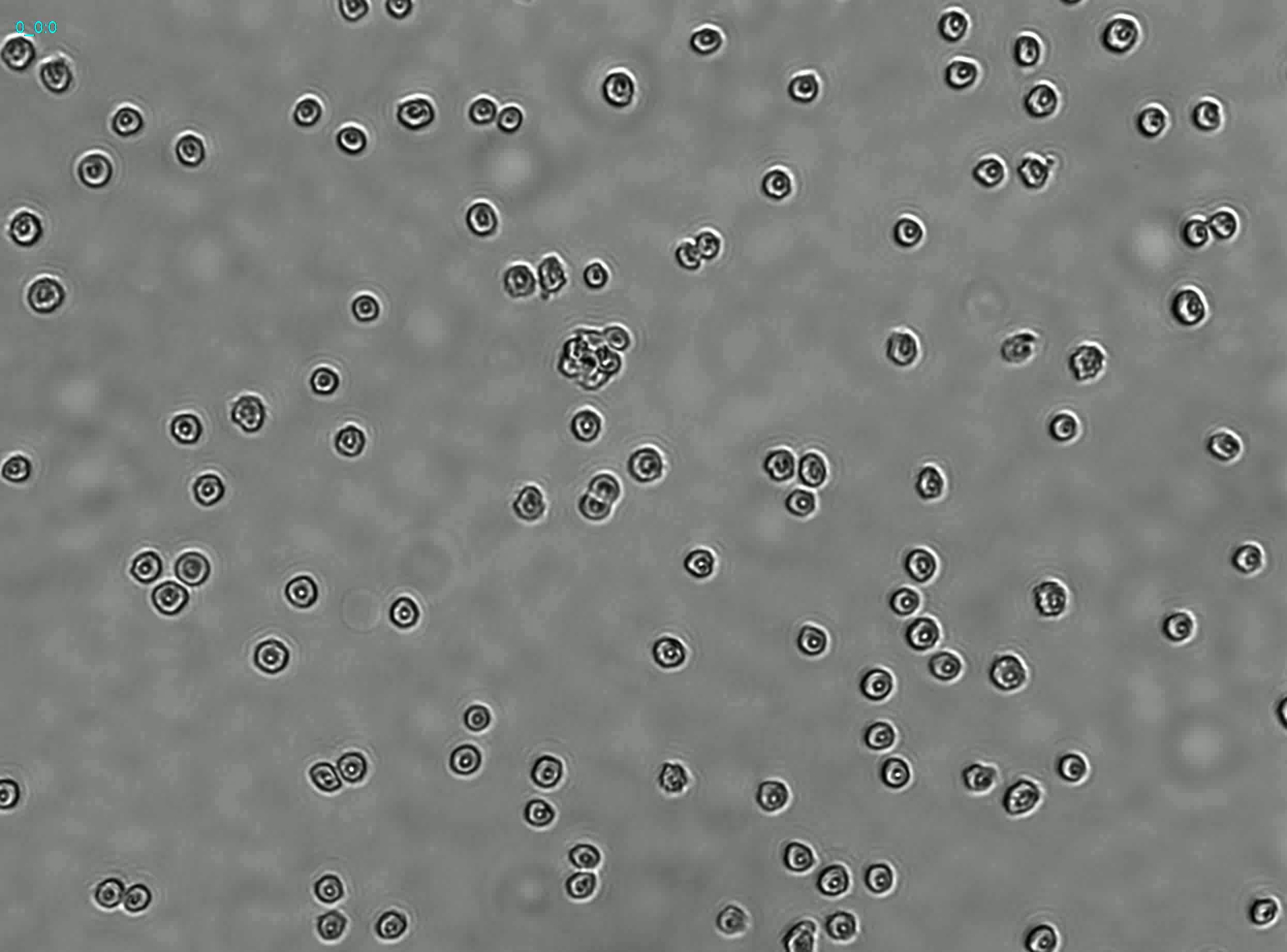

Supplement: Supplementary file 33 — Source data EV and Appendix [file 44318_2025_540_MOESM33_ESM.zip › Source data EV and Appendix/Figure EV 1/1B/0h.jpg]

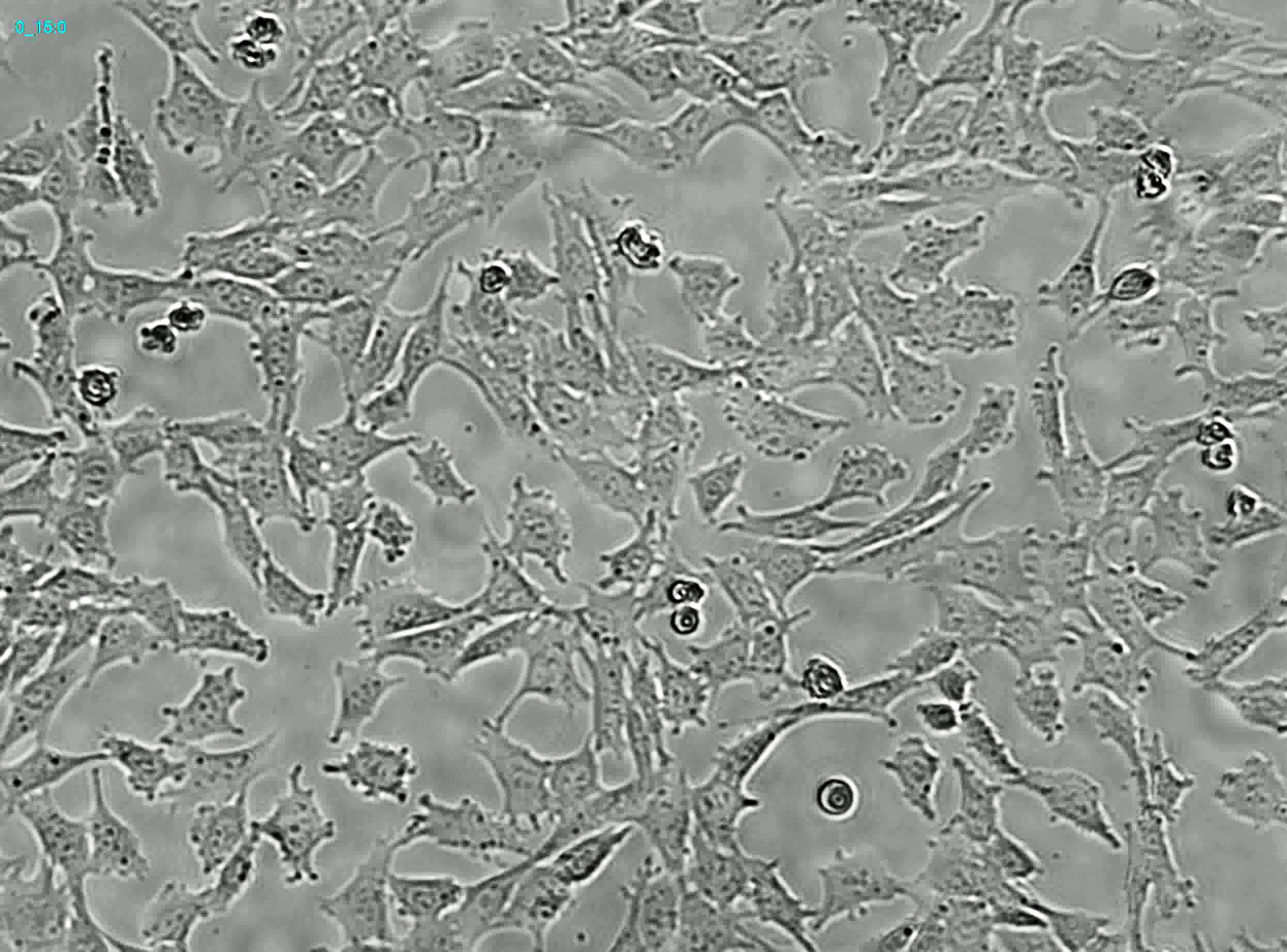

Supplement: Supplementary file 33 — Source data EV and Appendix [file 44318_2025_540_MOESM33_ESM.zip › Source data EV and Appendix/Figure EV 1/1B/15h.jpg]

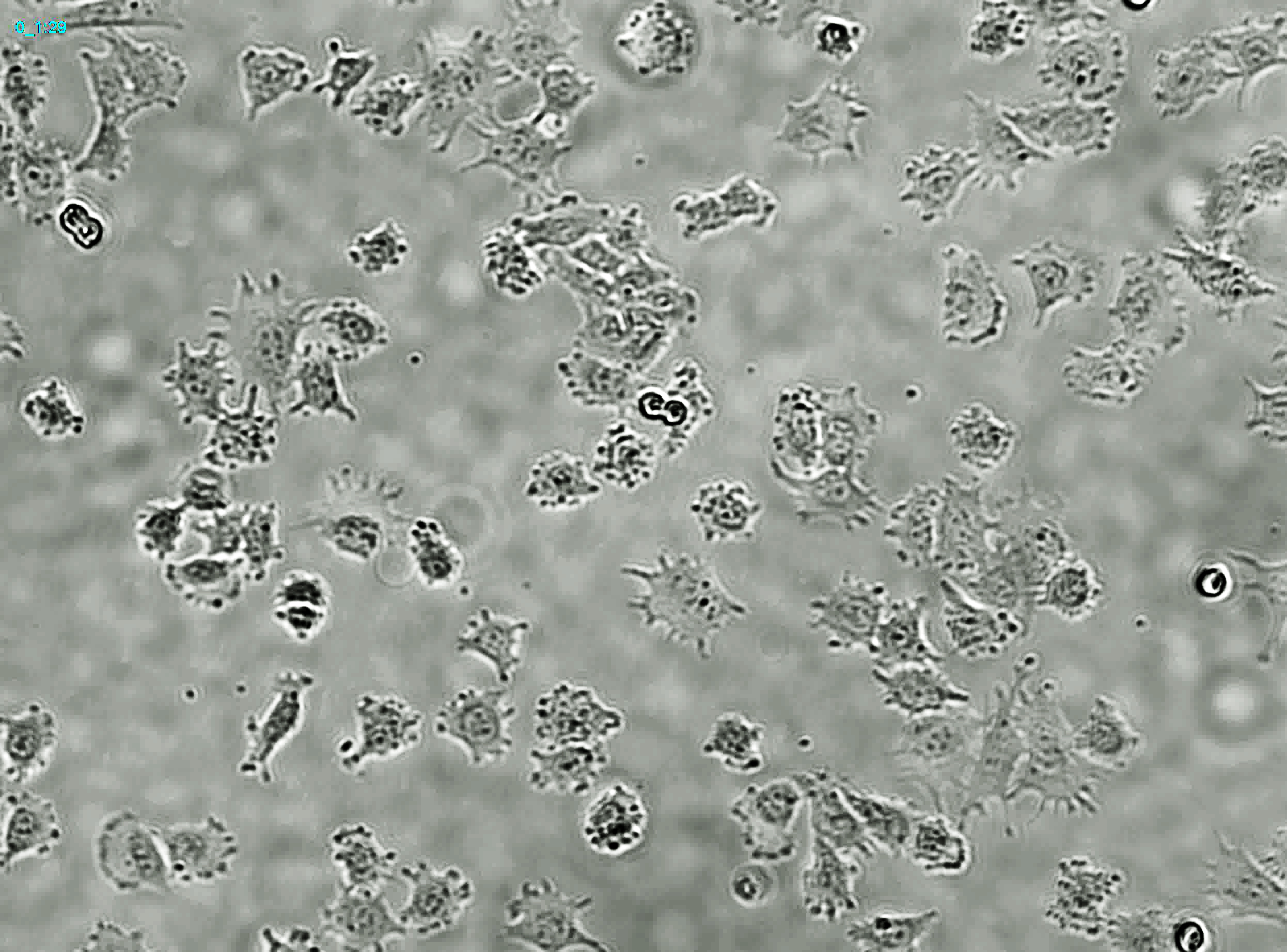

Supplement: Supplementary file 33 — Source data EV and Appendix [file 44318_2025_540_MOESM33_ESM.zip › Source data EV and Appendix/Figure EV 1/1B/1h 30min.jpg]

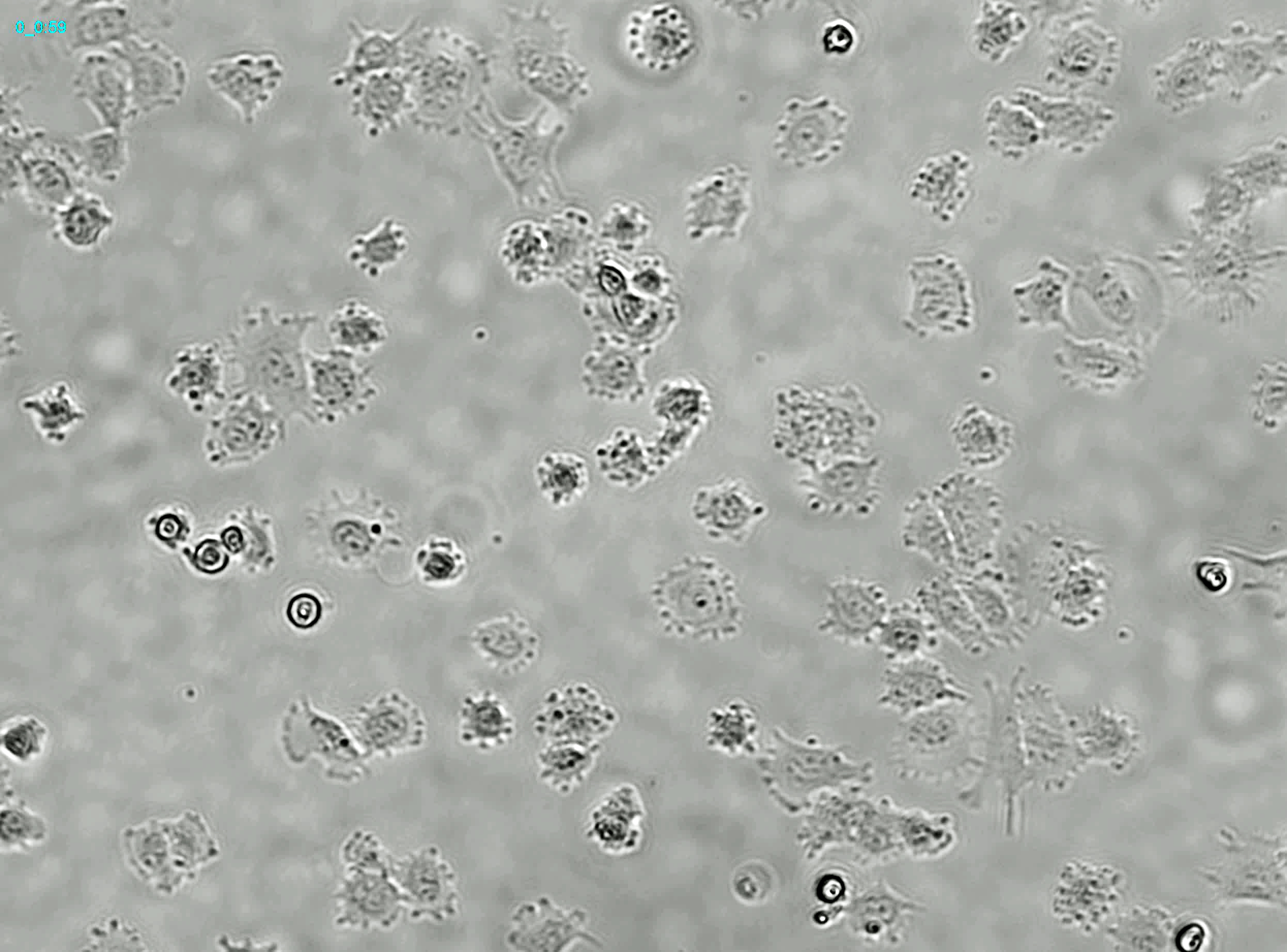

Supplement: Supplementary file 33 — Source data EV and Appendix [file 44318_2025_540_MOESM33_ESM.zip › Source data EV and Appendix/Figure EV 1/1B/1h.jpg]

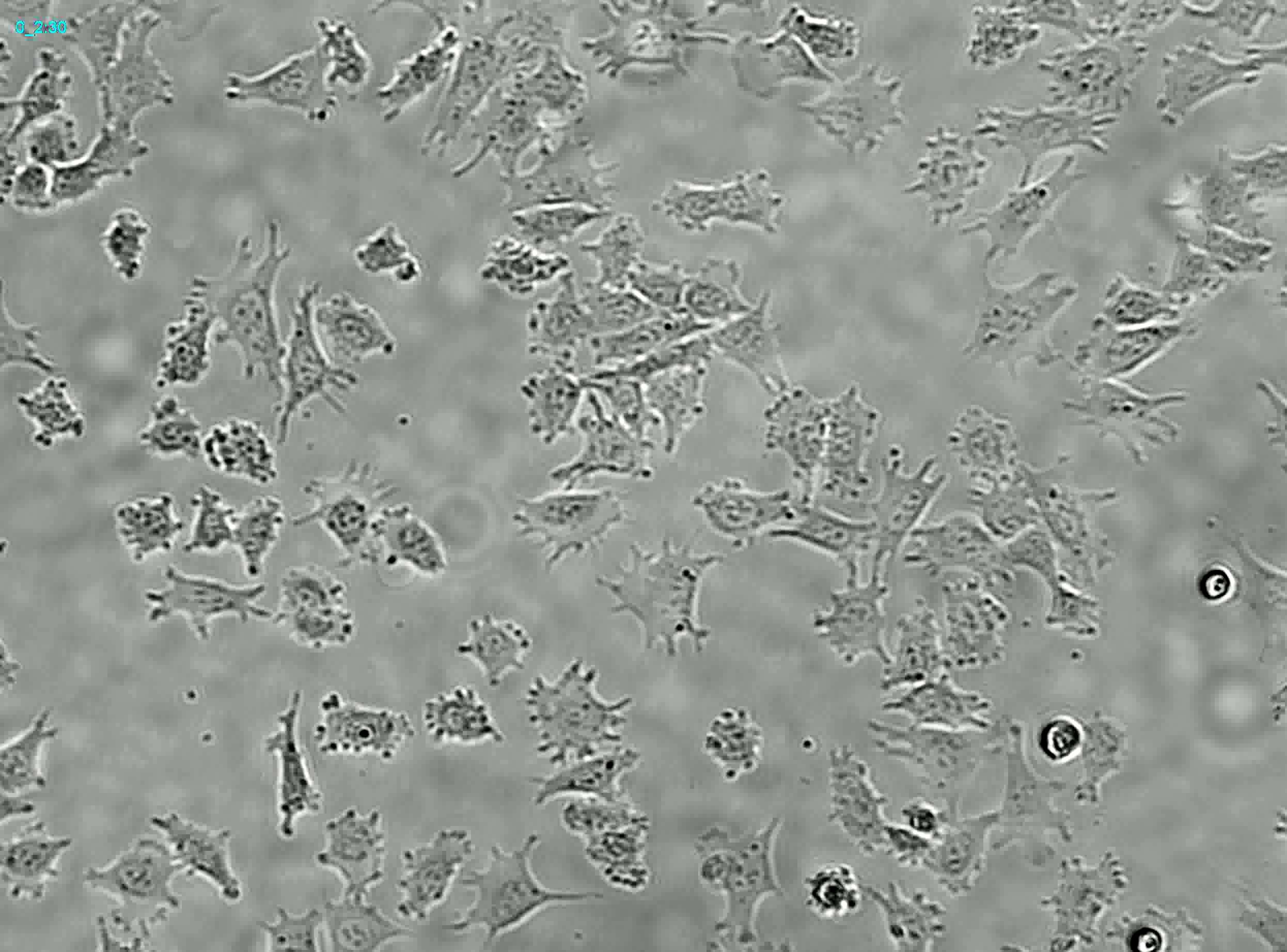

Supplement: Supplementary file 33 — Source data EV and Appendix [file 44318_2025_540_MOESM33_ESM.zip › Source data EV and Appendix/Figure EV 1/1B/2h 30 min.jpg]

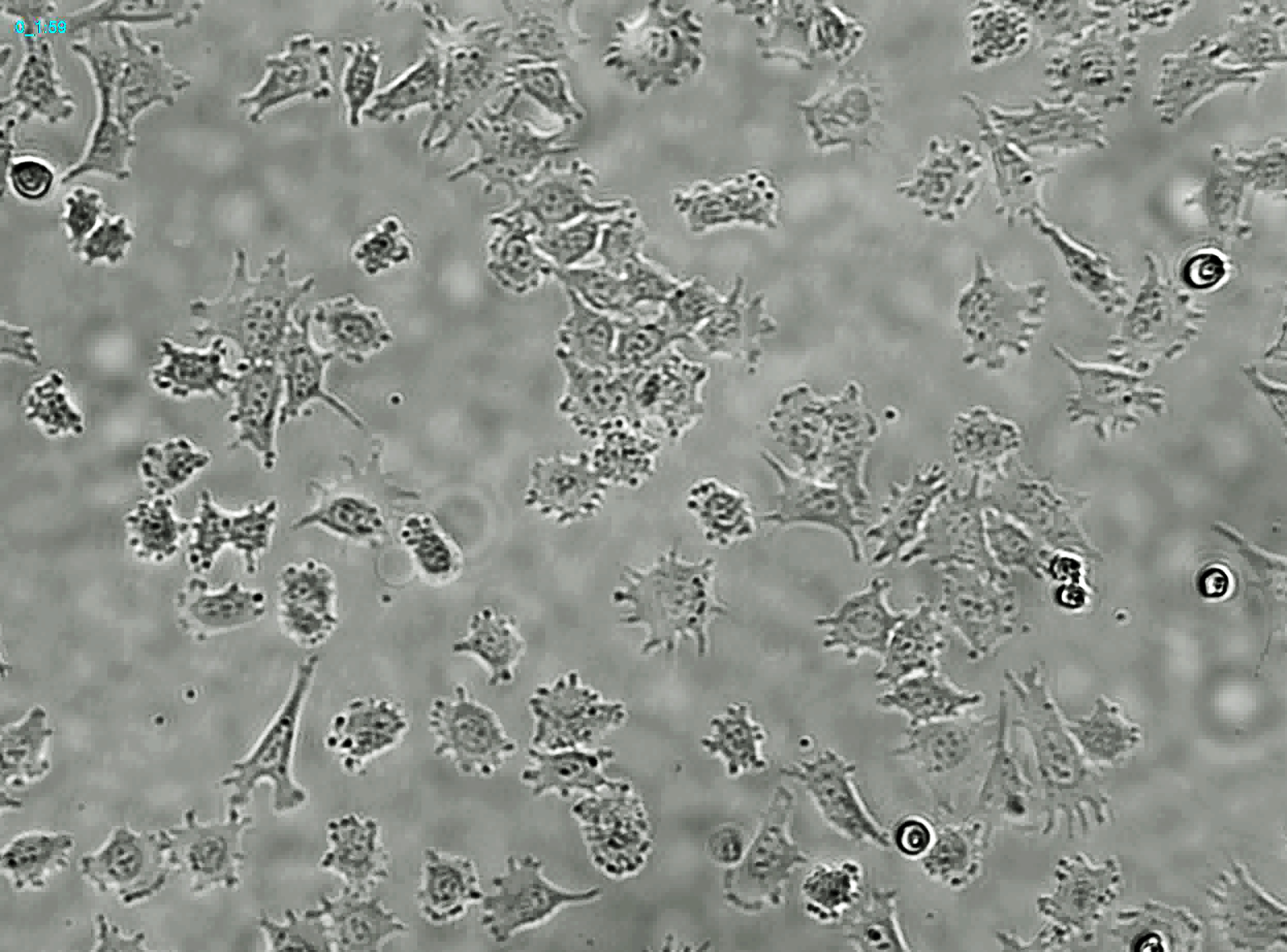

Supplement: Supplementary file 33 — Source data EV and Appendix [file 44318_2025_540_MOESM33_ESM.zip › Source data EV and Appendix/Figure EV 1/1B/2h.jpg]

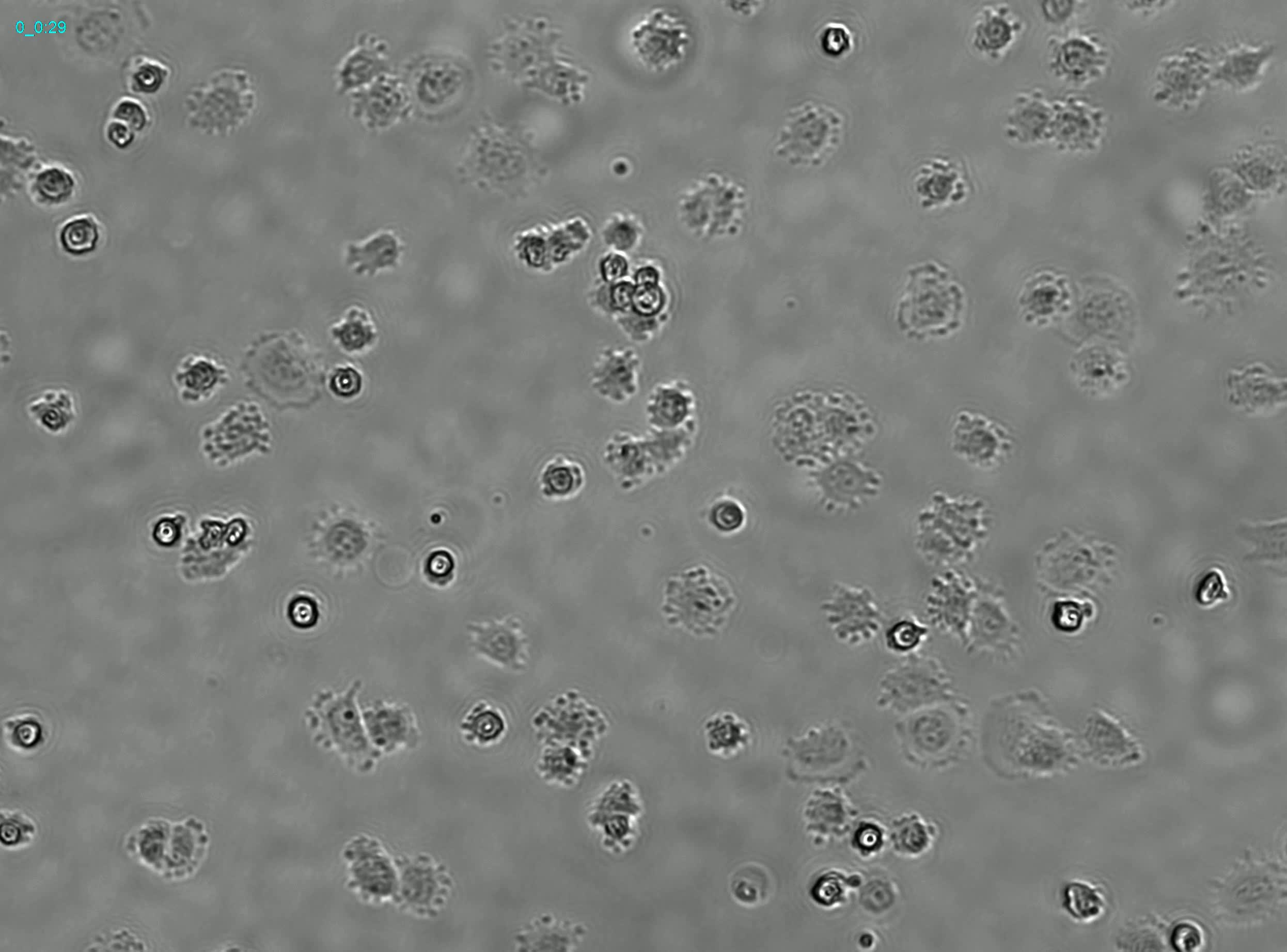

Supplement: Supplementary file 33 — Source data EV and Appendix [file 44318_2025_540_MOESM33_ESM.zip › Source data EV and Appendix/Figure EV 1/1B/30 min.jpg]

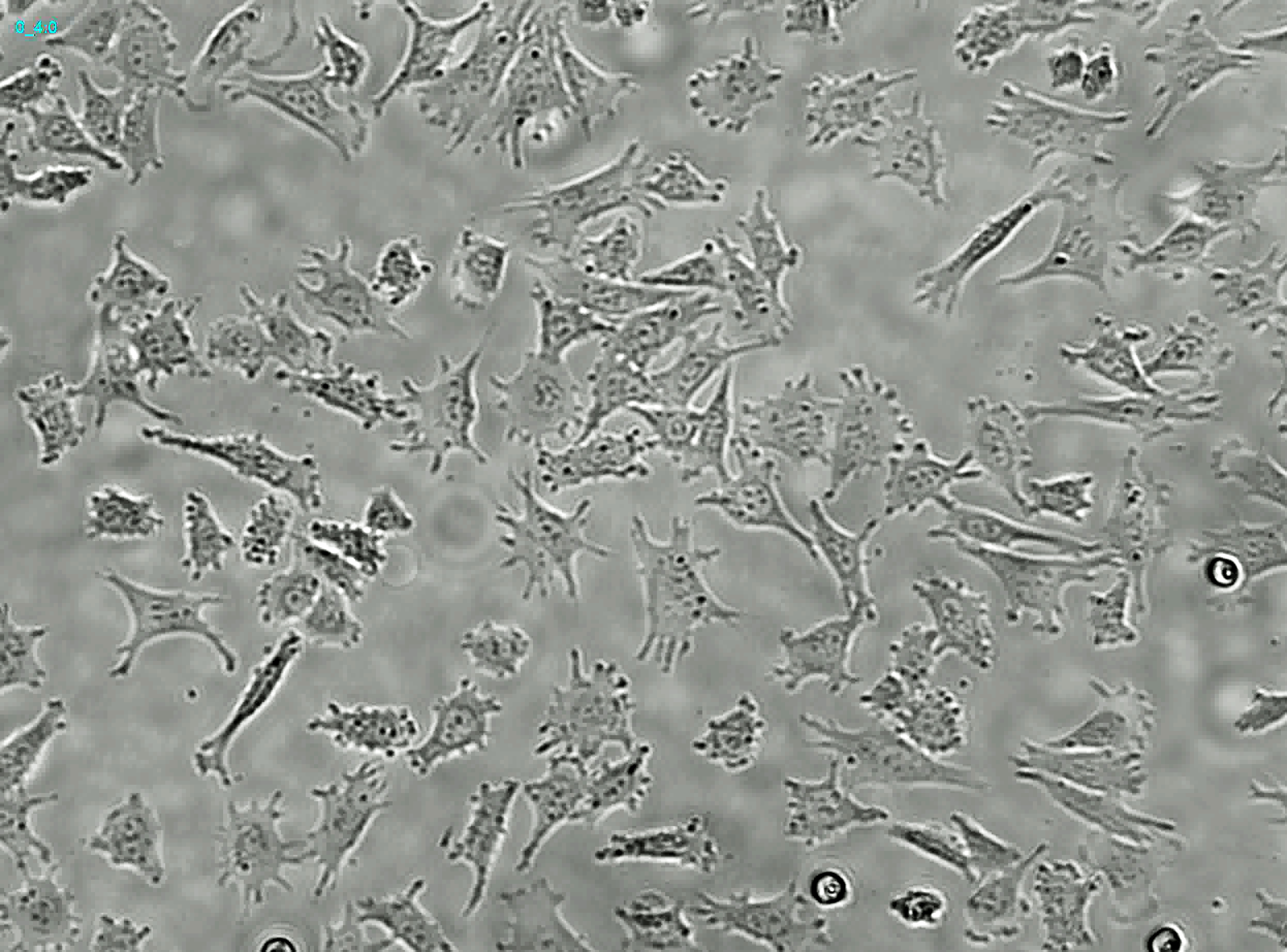

Supplement: Supplementary file 33 — Source data EV and Appendix [file 44318_2025_540_MOESM33_ESM.zip › Source data EV and Appendix/Figure EV 1/1B/4h.jpg]

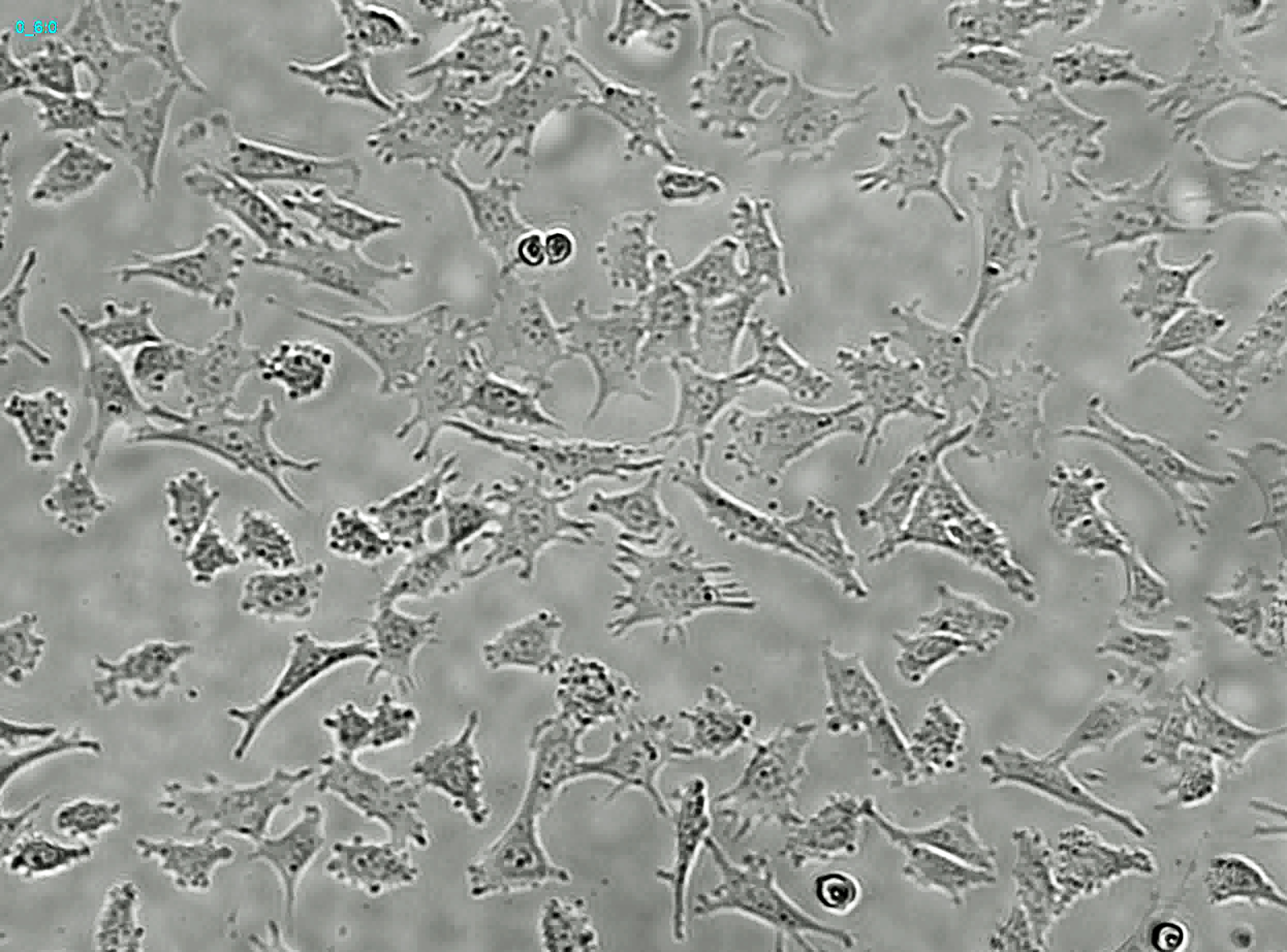

Supplement: Supplementary file 33 — Source data EV and Appendix [file 44318_2025_540_MOESM33_ESM.zip › Source data EV and Appendix/Figure EV 1/1B/6h.jpg]

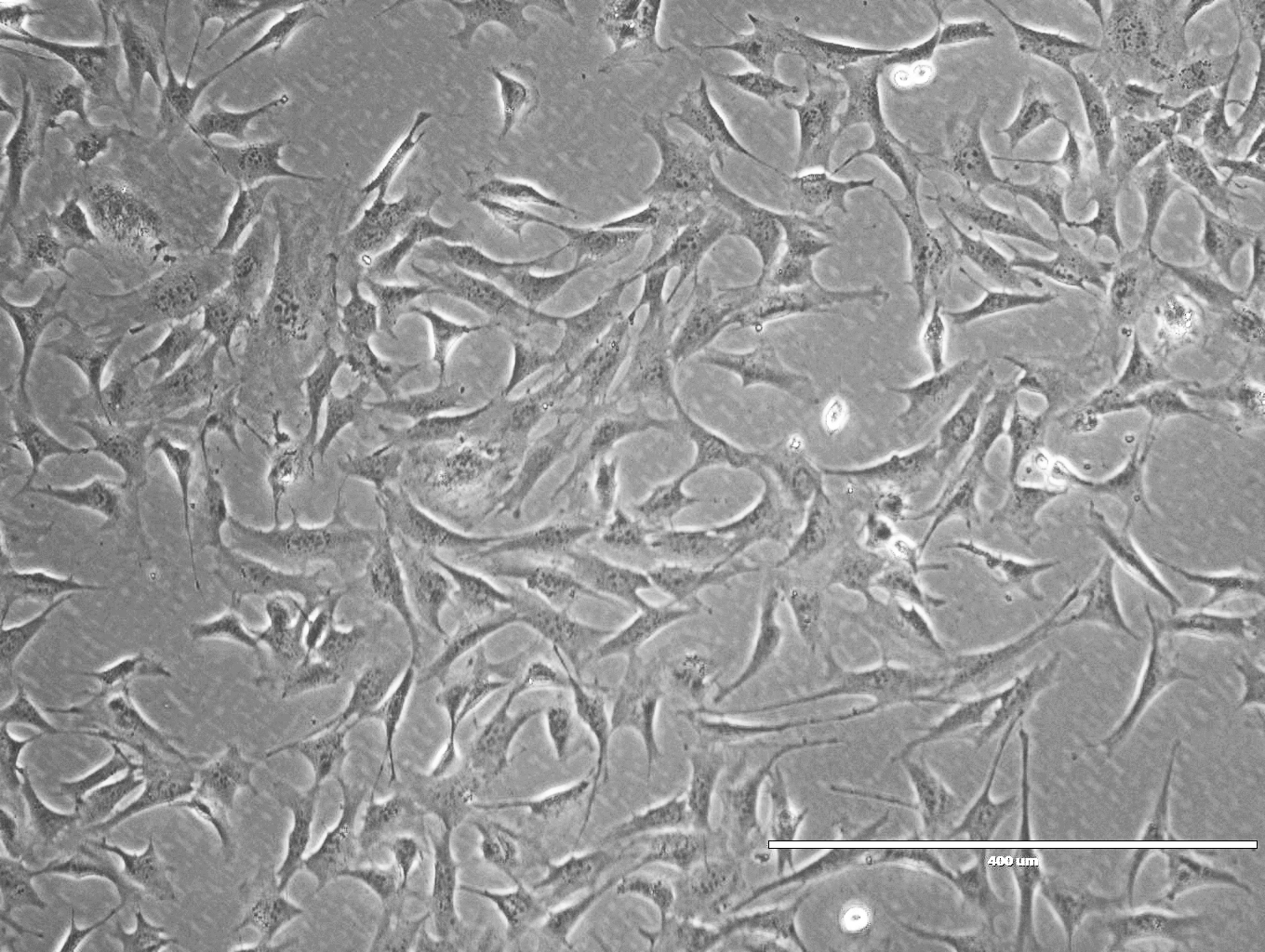

Supplement: Supplementary file 33 — Source data EV and Appendix [file 44318_2025_540_MOESM33_ESM.zip › Source data EV and Appendix/Figure EV 1/1D/ALUM/MEF_0h.jpg]

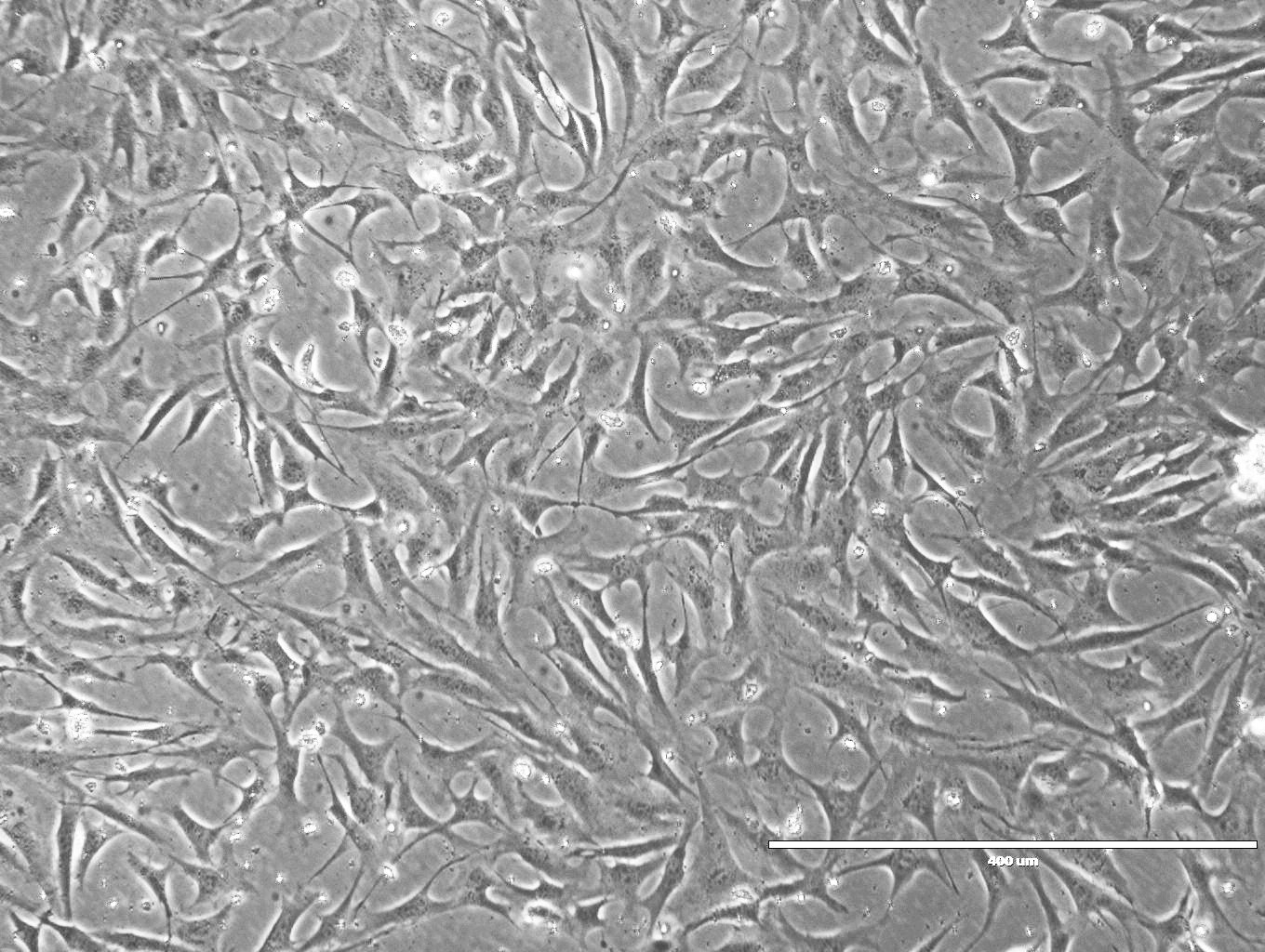

Supplement: Supplementary file 33 — Source data EV and Appendix [file 44318_2025_540_MOESM33_ESM.zip › Source data EV and Appendix/Figure EV 1/1D/ALUM/MEF_12h.jpg]

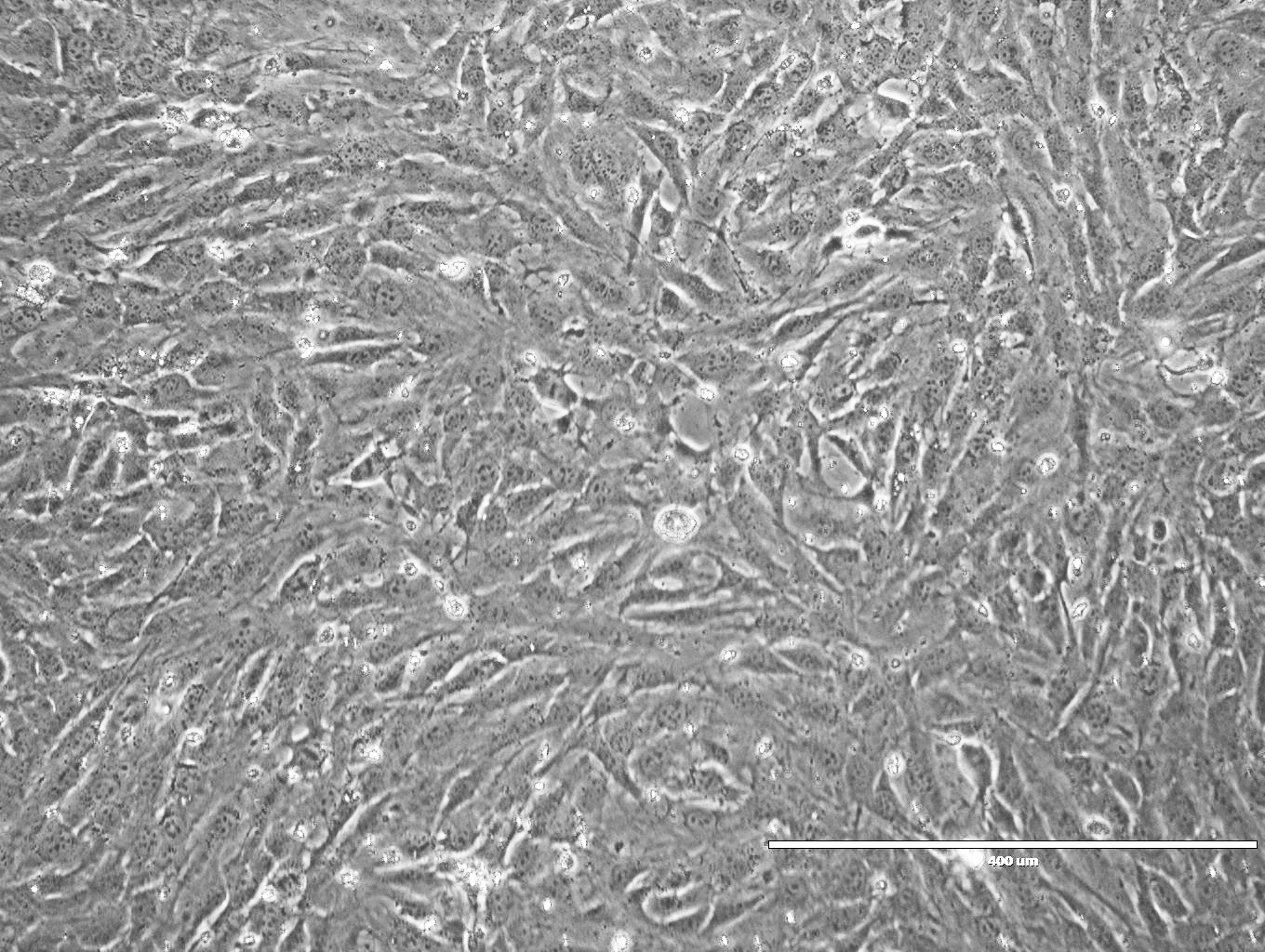

Supplement: Supplementary file 33 — Source data EV and Appendix [file 44318_2025_540_MOESM33_ESM.zip › Source data EV and Appendix/Figure EV 1/1D/ALUM/MEF_24h.jpg]

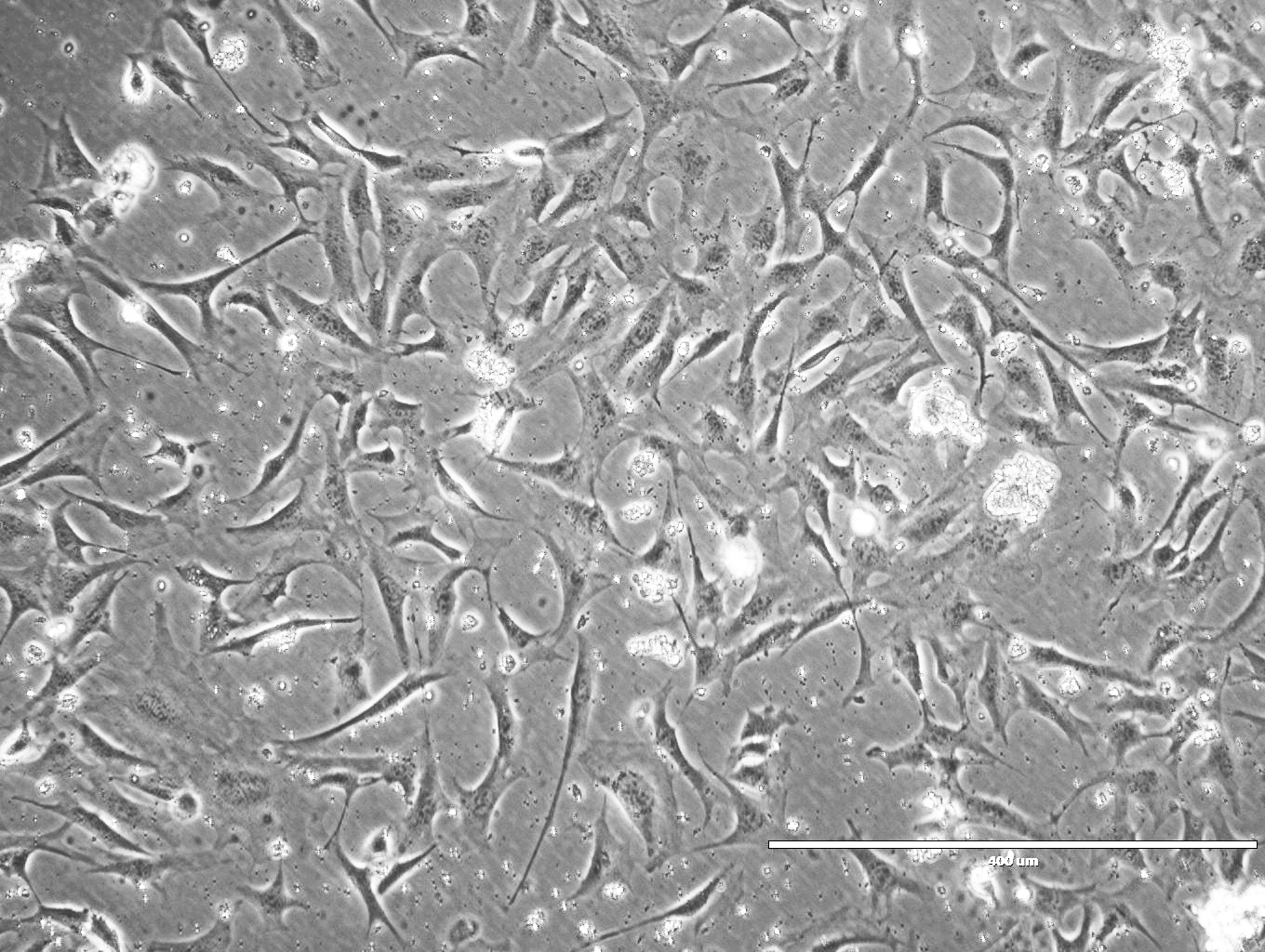

Supplement: Supplementary file 33 — Source data EV and Appendix [file 44318_2025_540_MOESM33_ESM.zip › Source data EV and Appendix/Figure EV 1/1D/ALUM/MEF_3h.jpg]

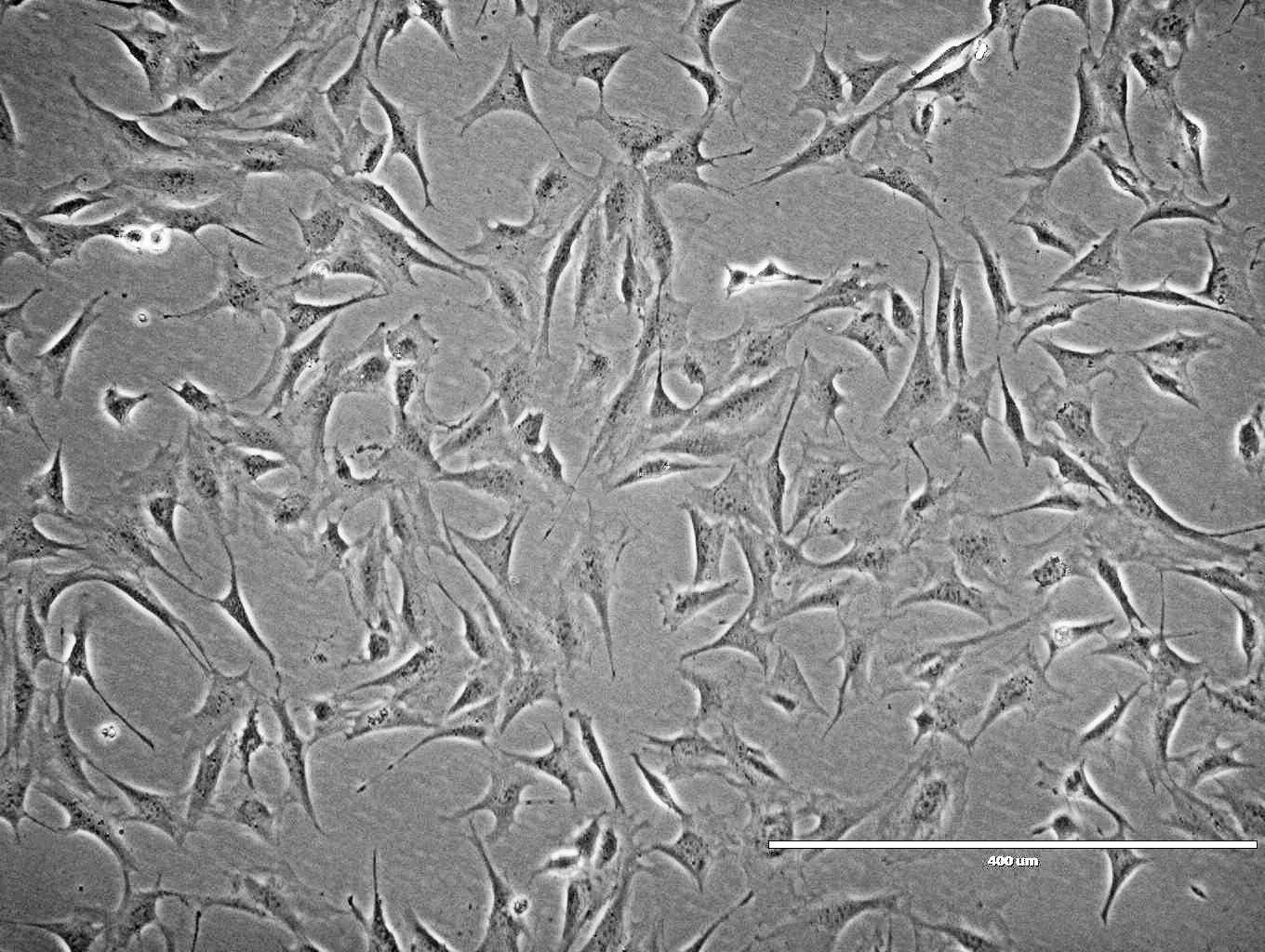

Supplement: Supplementary file 33 — Source data EV and Appendix [file 44318_2025_540_MOESM33_ESM.zip › Source data EV and Appendix/Figure EV 1/1D/GPN/MEF_0 h.jpg]

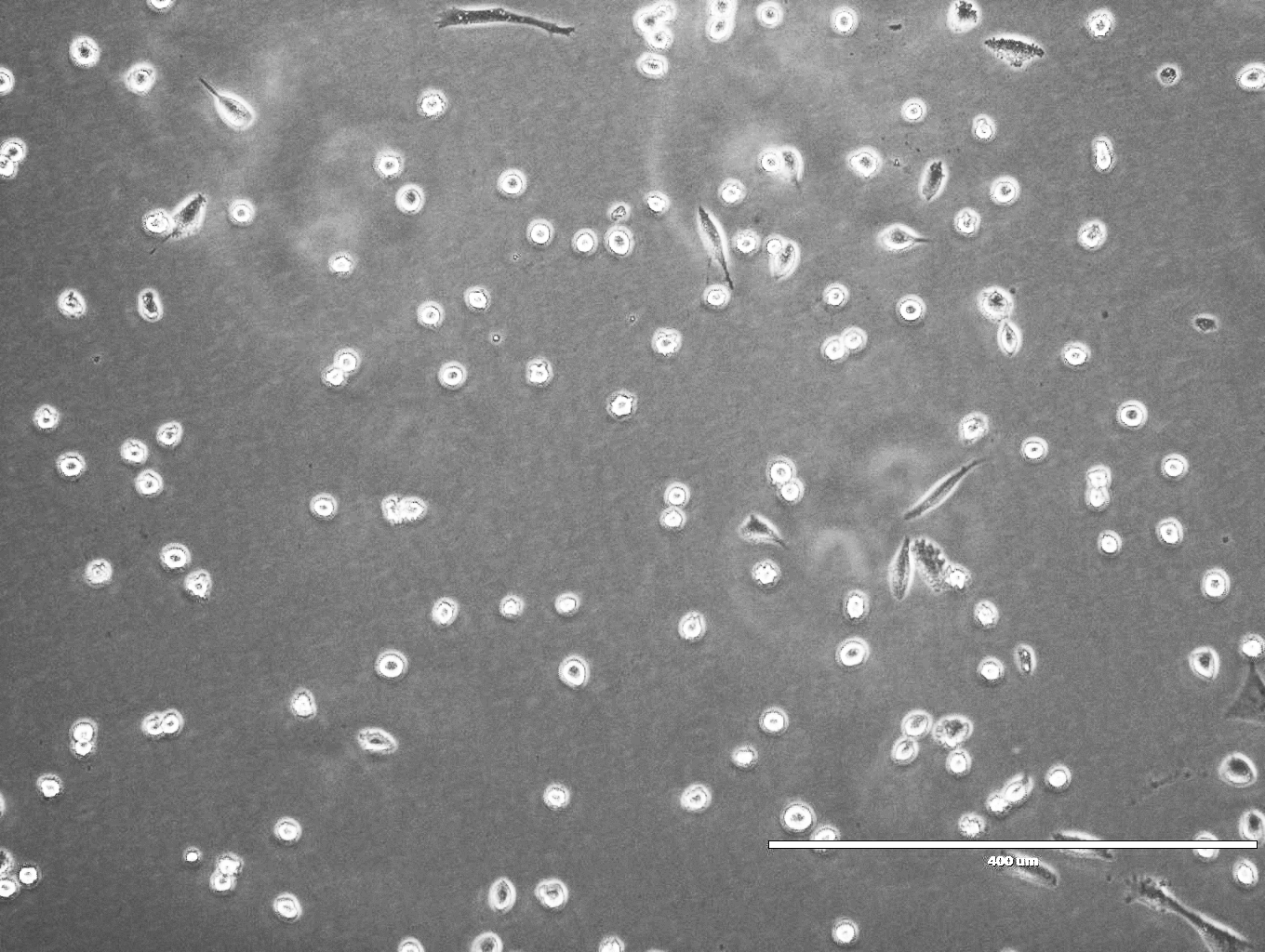

Supplement: Supplementary file 33 — Source data EV and Appendix [file 44318_2025_540_MOESM33_ESM.zip › Source data EV and Appendix/Figure EV 1/1D/GPN/MEF_1.5 h.jpg]

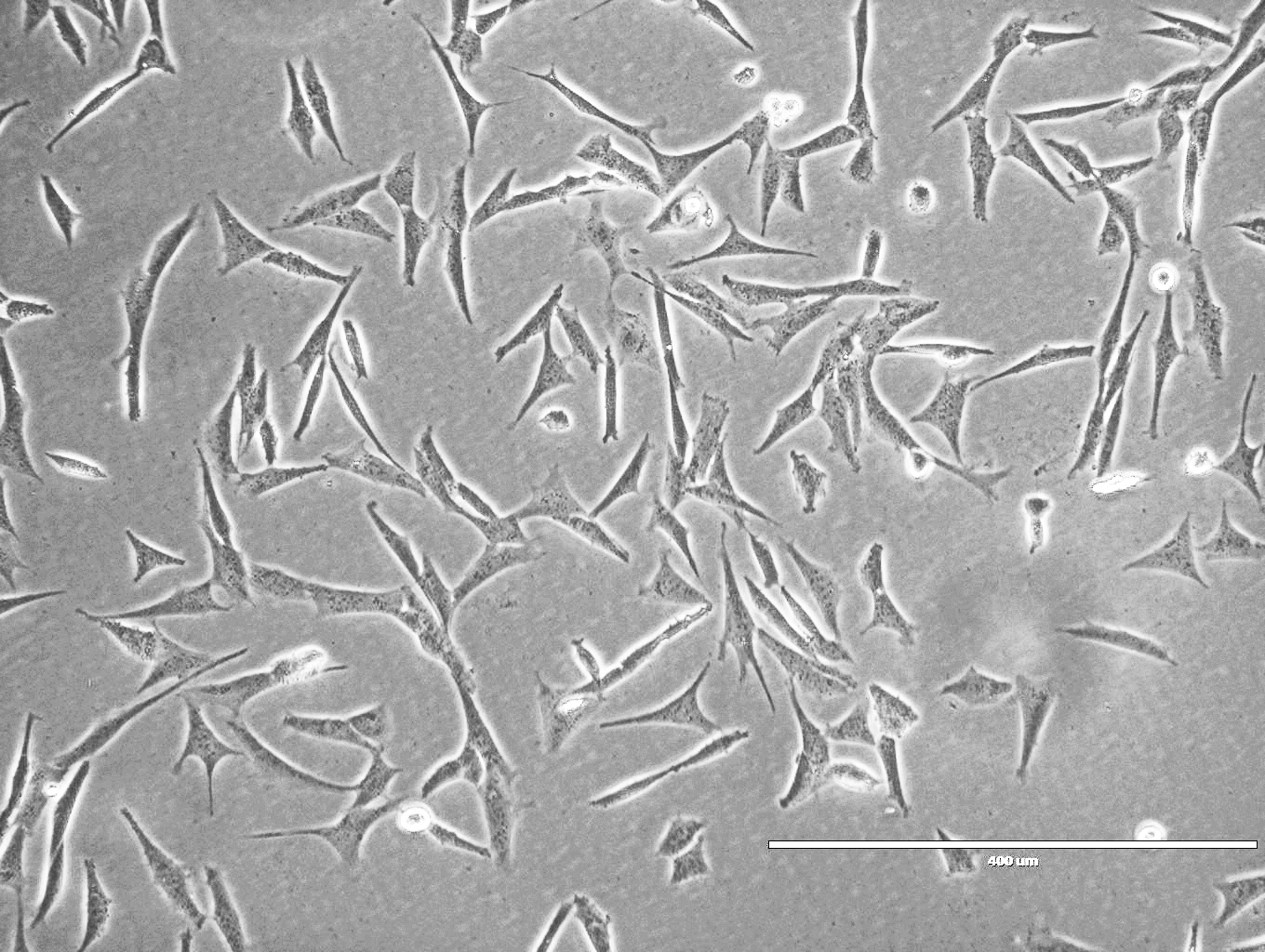

Supplement: Supplementary file 33 — Source data EV and Appendix [file 44318_2025_540_MOESM33_ESM.zip › Source data EV and Appendix/Figure EV 1/1D/GPN/MEF_12 h.jpg]

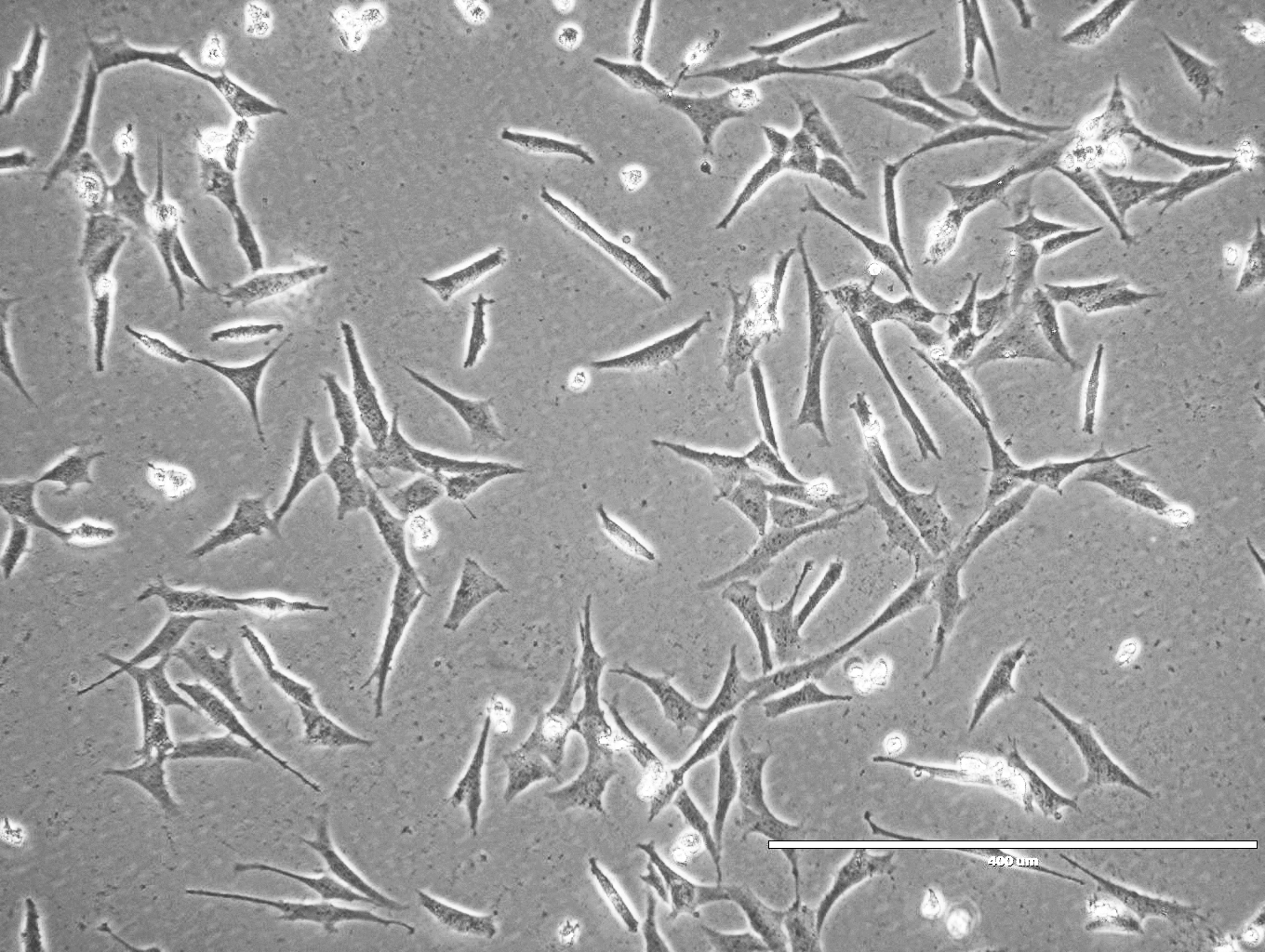

Supplement: Supplementary file 33 — Source data EV and Appendix [file 44318_2025_540_MOESM33_ESM.zip › Source data EV and Appendix/Figure EV 1/1D/GPN/MEF_24 h.jpg]

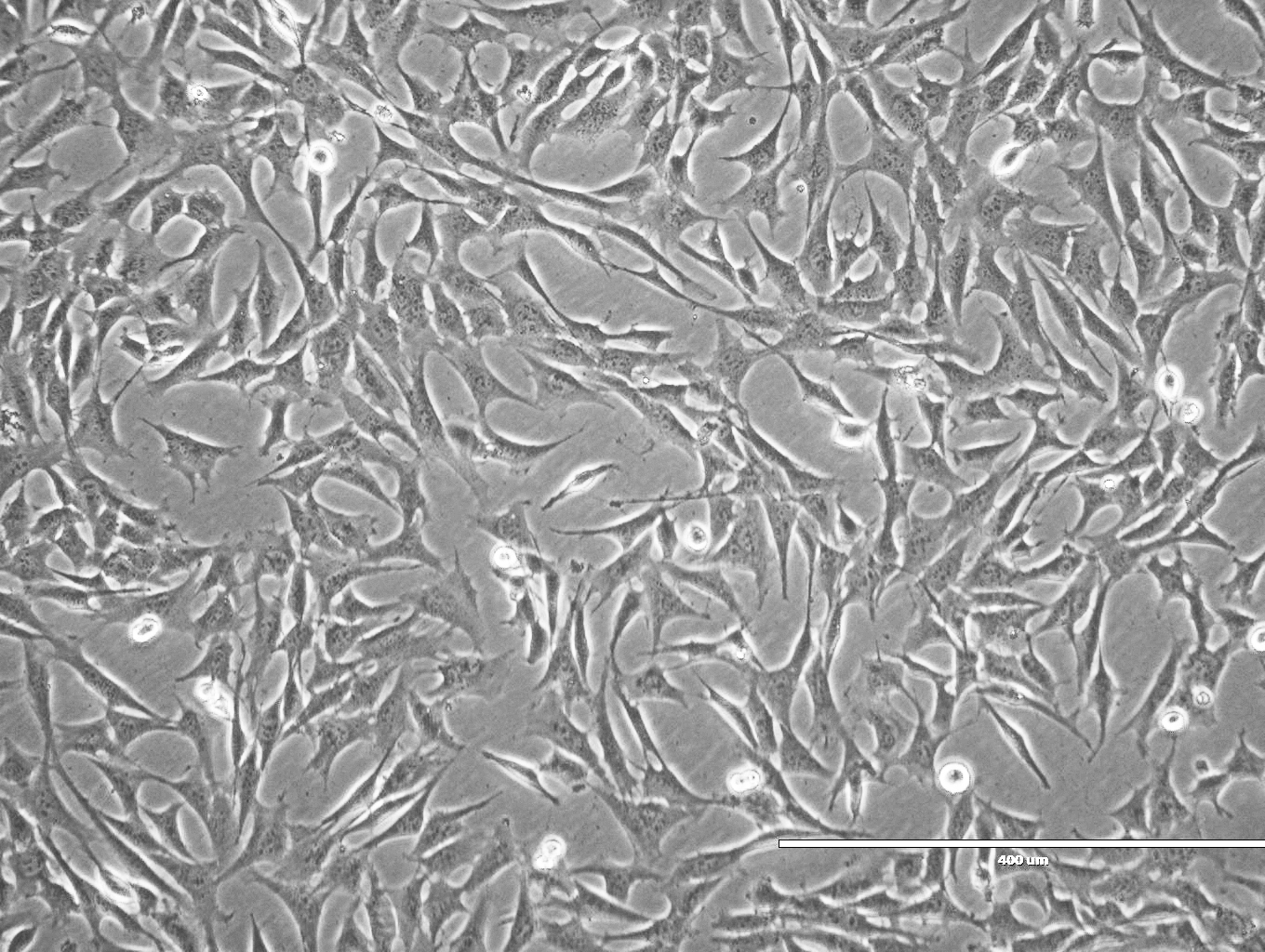

Supplement: Supplementary file 33 — Source data EV and Appendix [file 44318_2025_540_MOESM33_ESM.zip › Source data EV and Appendix/Figure EV 1/1D/LLOMe/MEF_0 h.jpg]

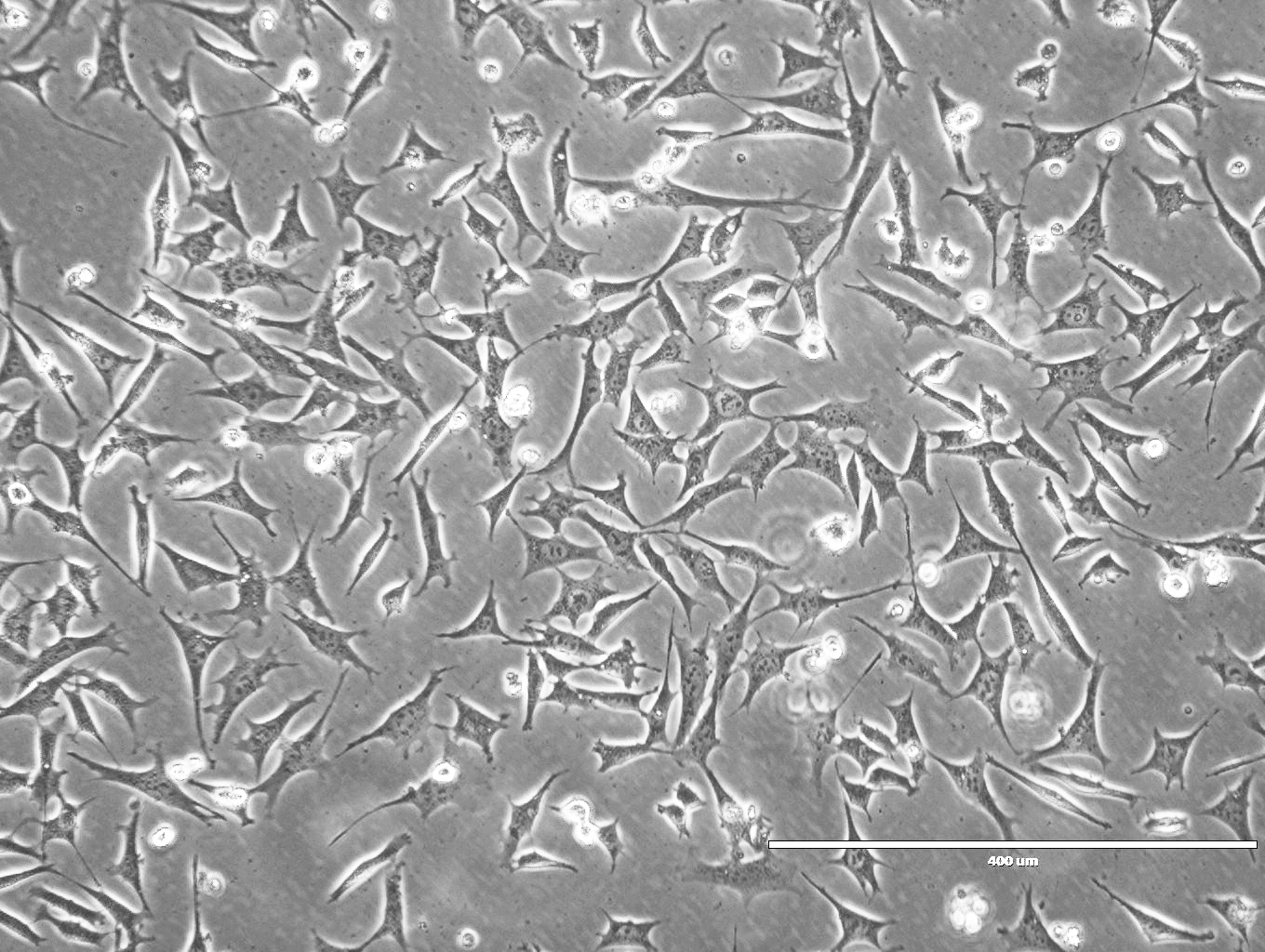

Supplement: Supplementary file 33 — Source data EV and Appendix [file 44318_2025_540_MOESM33_ESM.zip › Source data EV and Appendix/Figure EV 1/1D/LLOMe/MEF_12 h.jpg]

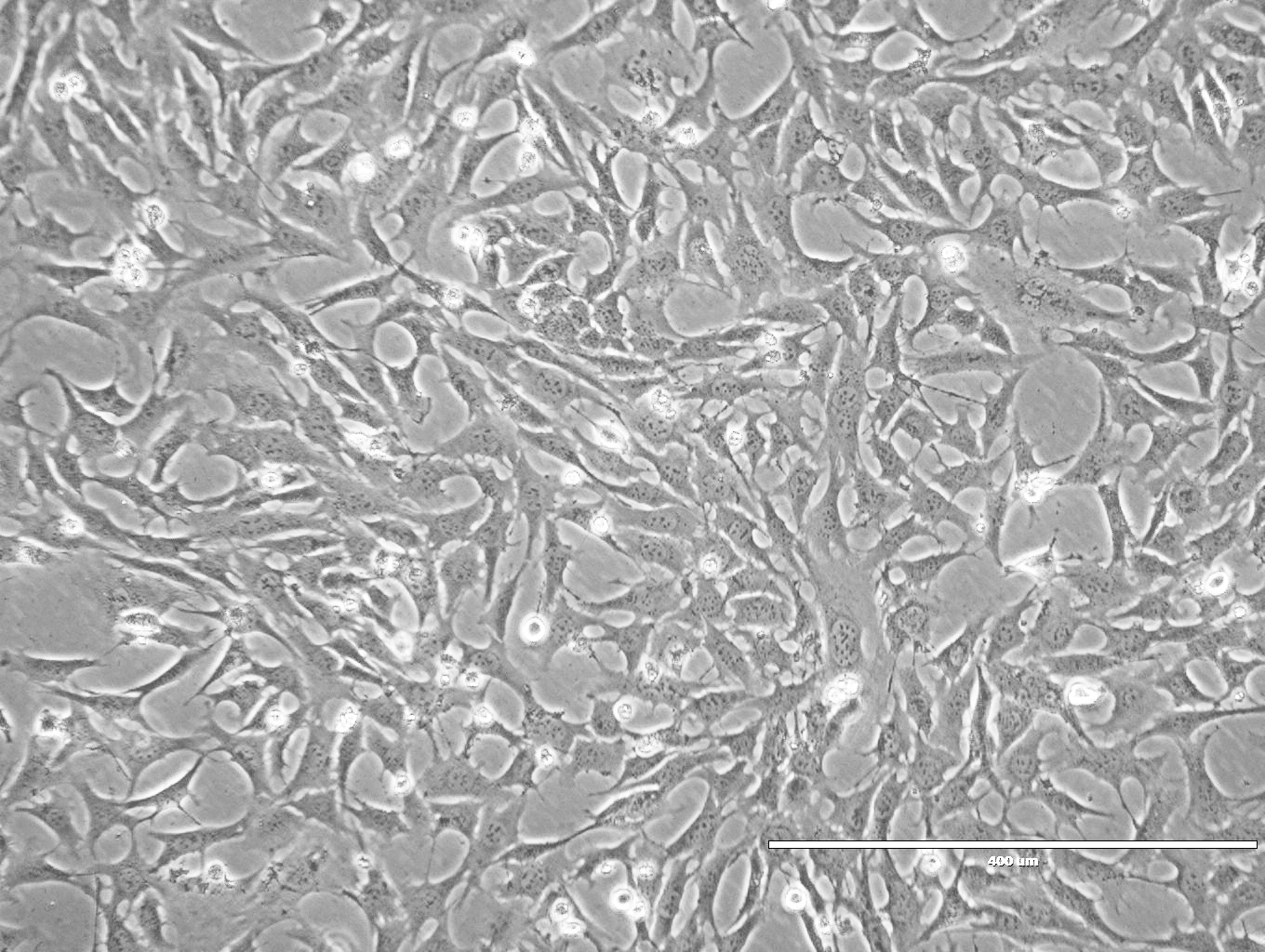

Supplement: Supplementary file 33 — Source data EV and Appendix [file 44318_2025_540_MOESM33_ESM.zip › Source data EV and Appendix/Figure EV 1/1D/LLOMe/MEF_24 h.jpg]

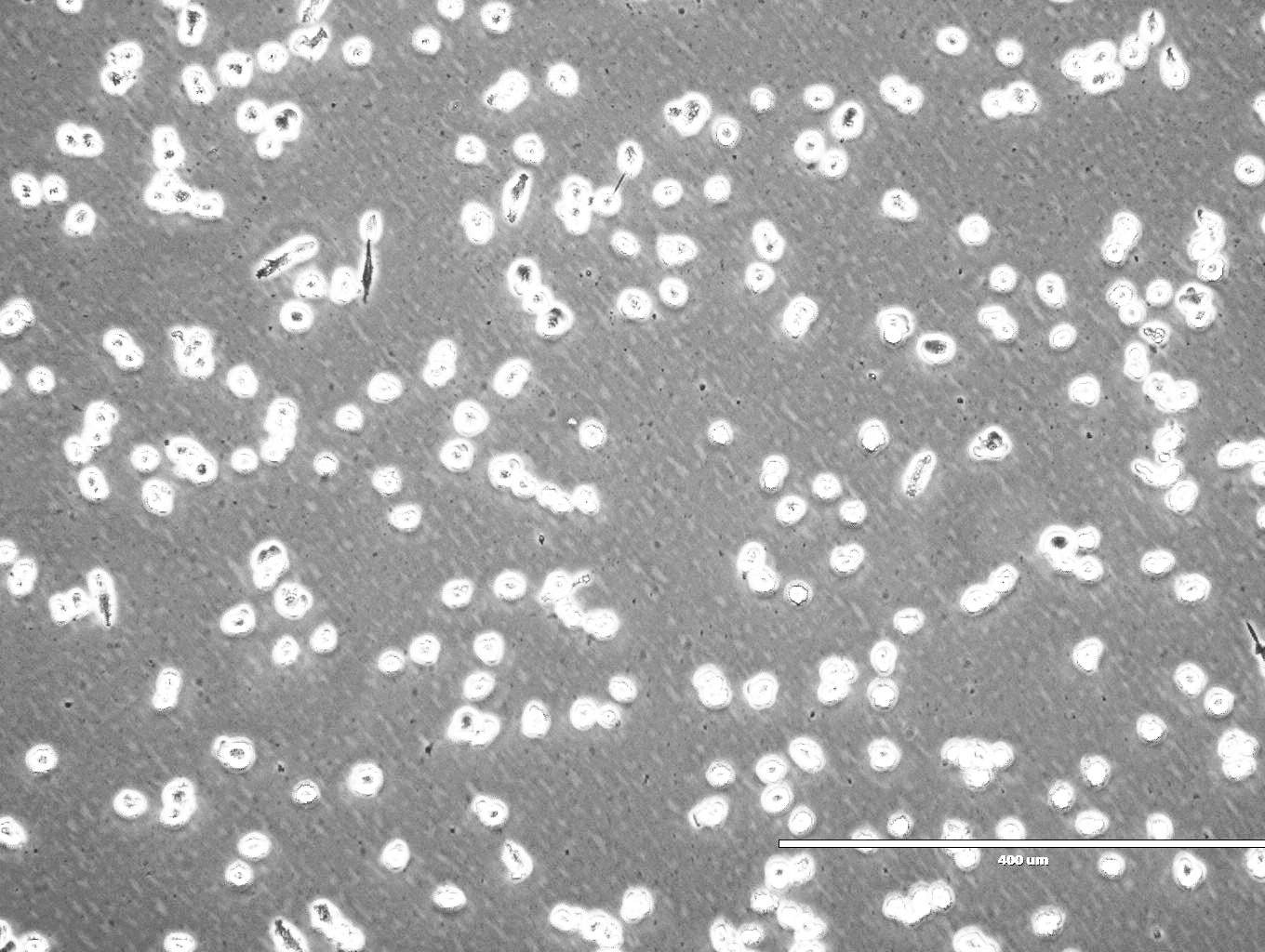

Supplement: Supplementary file 33 — Source data EV and Appendix [file 44318_2025_540_MOESM33_ESM.zip › Source data EV and Appendix/Figure EV 1/1D/LLOMe/MEF_30 min.jpg]

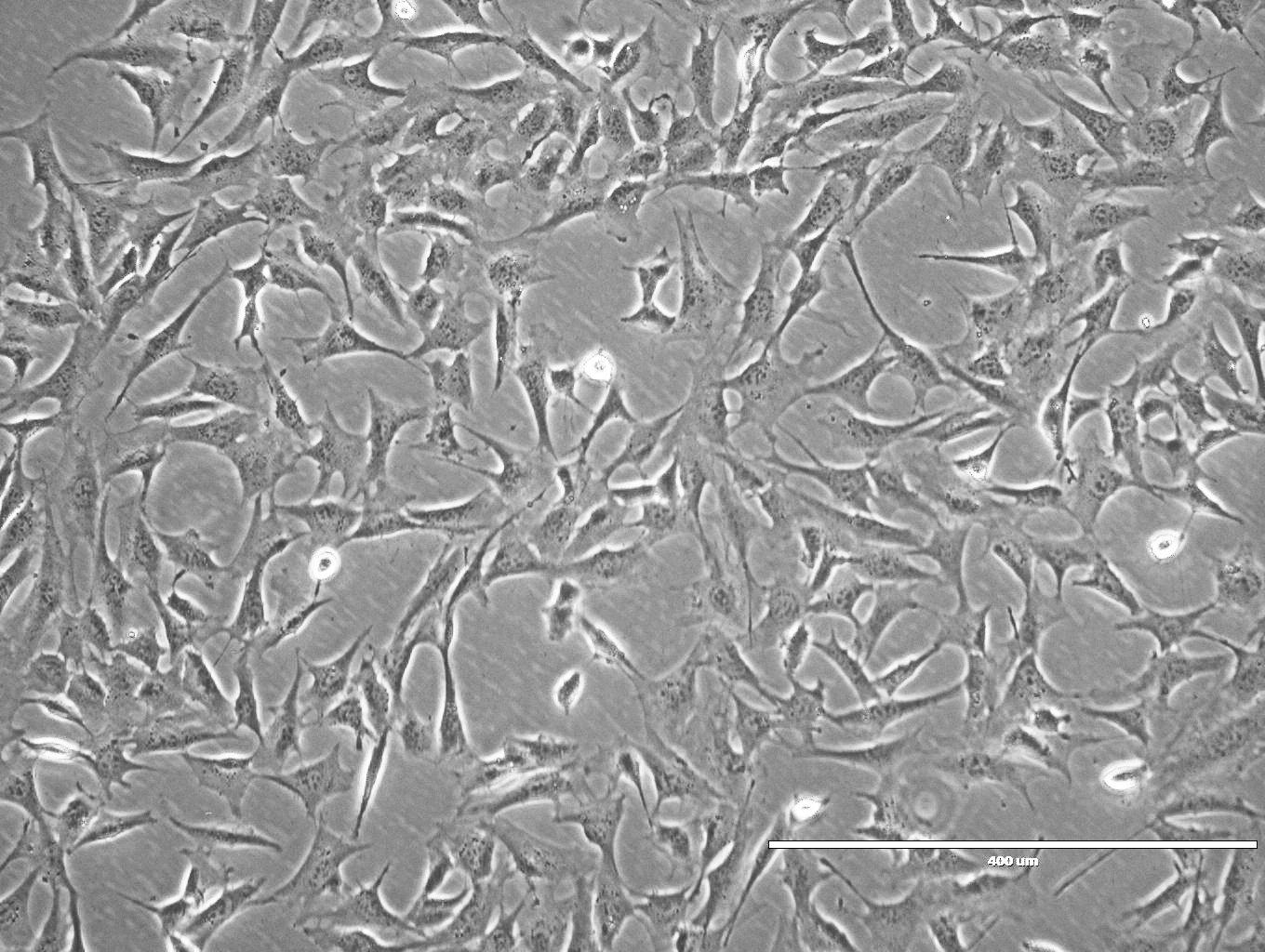

Supplement: Supplementary file 33 — Source data EV and Appendix [file 44318_2025_540_MOESM33_ESM.zip › Source data EV and Appendix/Figure EV 1/1D/Silica/MEF_0 h.jpg]

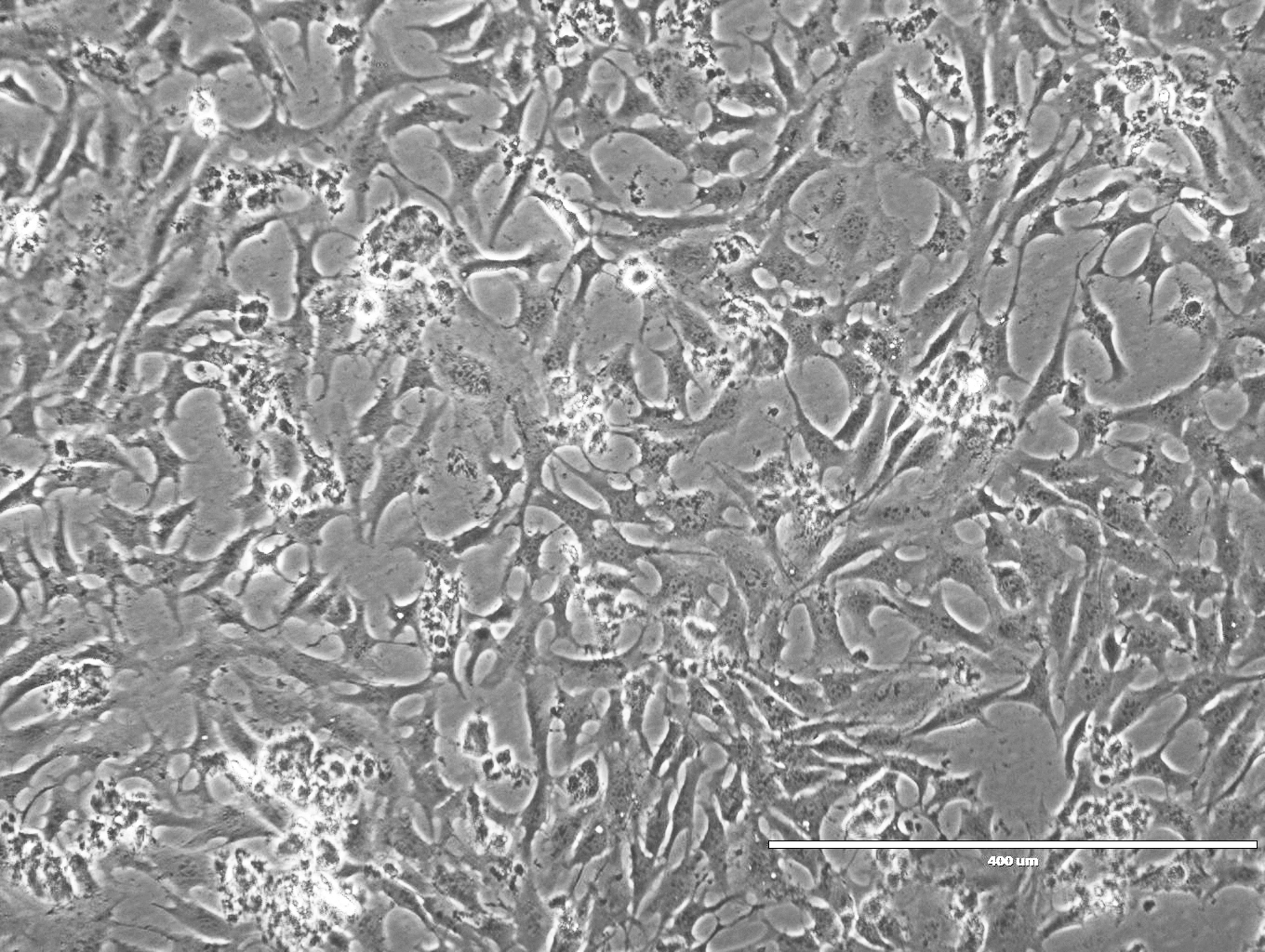

Supplement: Supplementary file 33 — Source data EV and Appendix [file 44318_2025_540_MOESM33_ESM.zip › Source data EV and Appendix/Figure EV 1/1D/Silica/MEF_12 h.jpg]

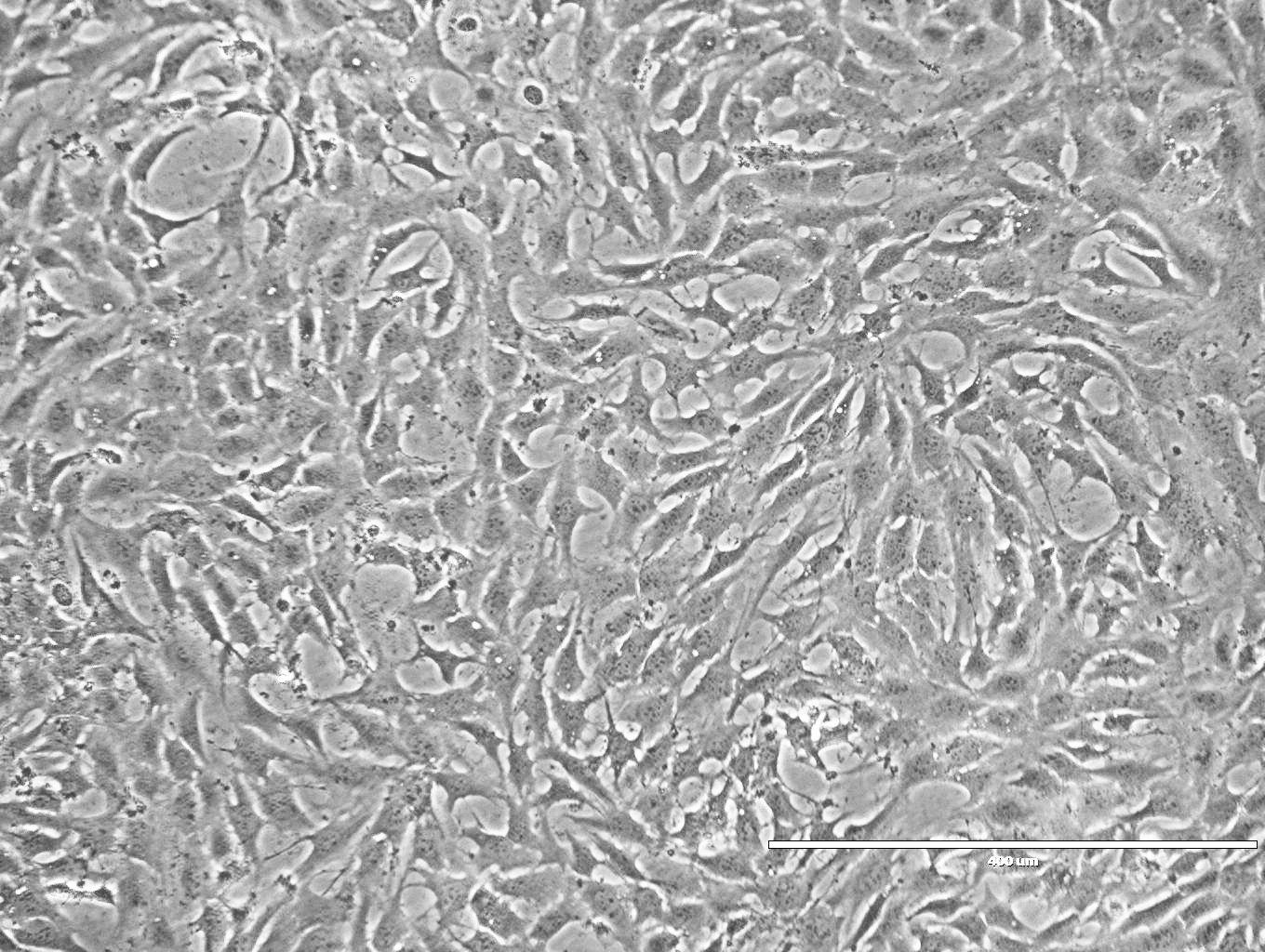

Supplement: Supplementary file 33 — Source data EV and Appendix [file 44318_2025_540_MOESM33_ESM.zip › Source data EV and Appendix/Figure EV 1/1D/Silica/MEF_24 h .jpg]

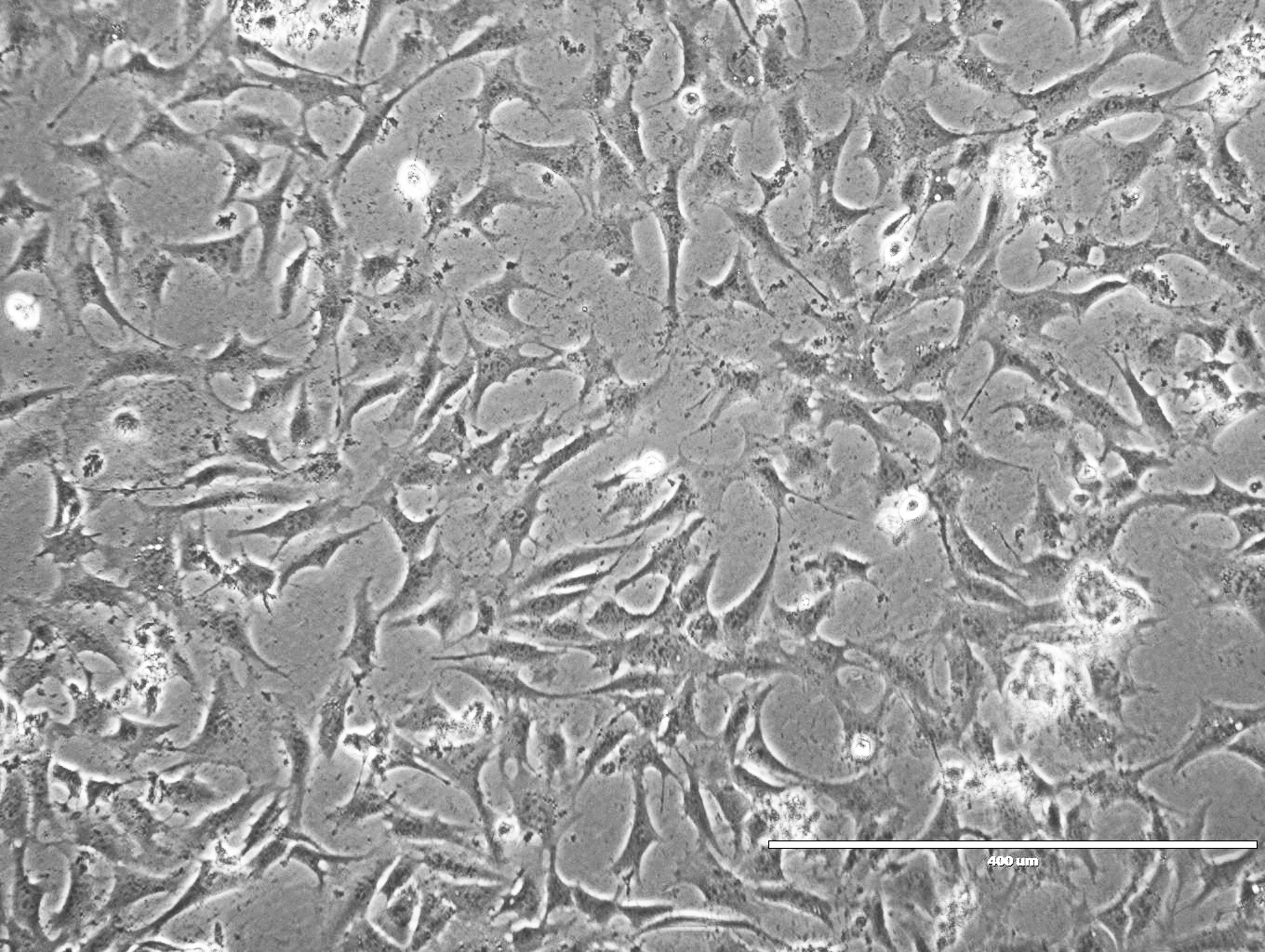

Supplement: Supplementary file 33 — Source data EV and Appendix [file 44318_2025_540_MOESM33_ESM.zip › Source data EV and Appendix/Figure EV 1/1D/Silica/MEF_3 h.jpg]

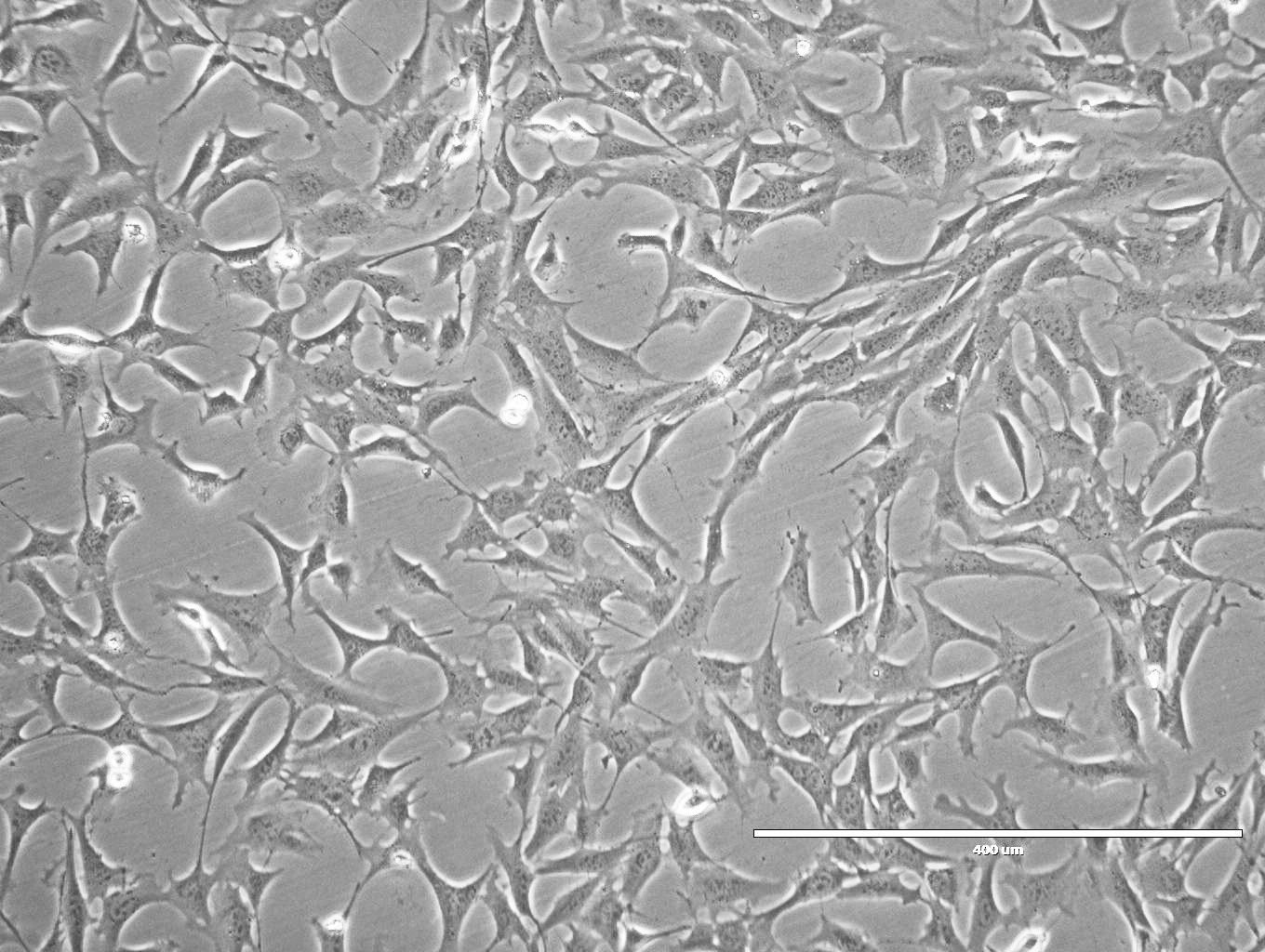

Supplement: Supplementary file 33 — Source data EV and Appendix [file 44318_2025_540_MOESM33_ESM.zip › Source data EV and Appendix/Figure EV 1/1D/Siramesine/MEF_0 h.jpg]

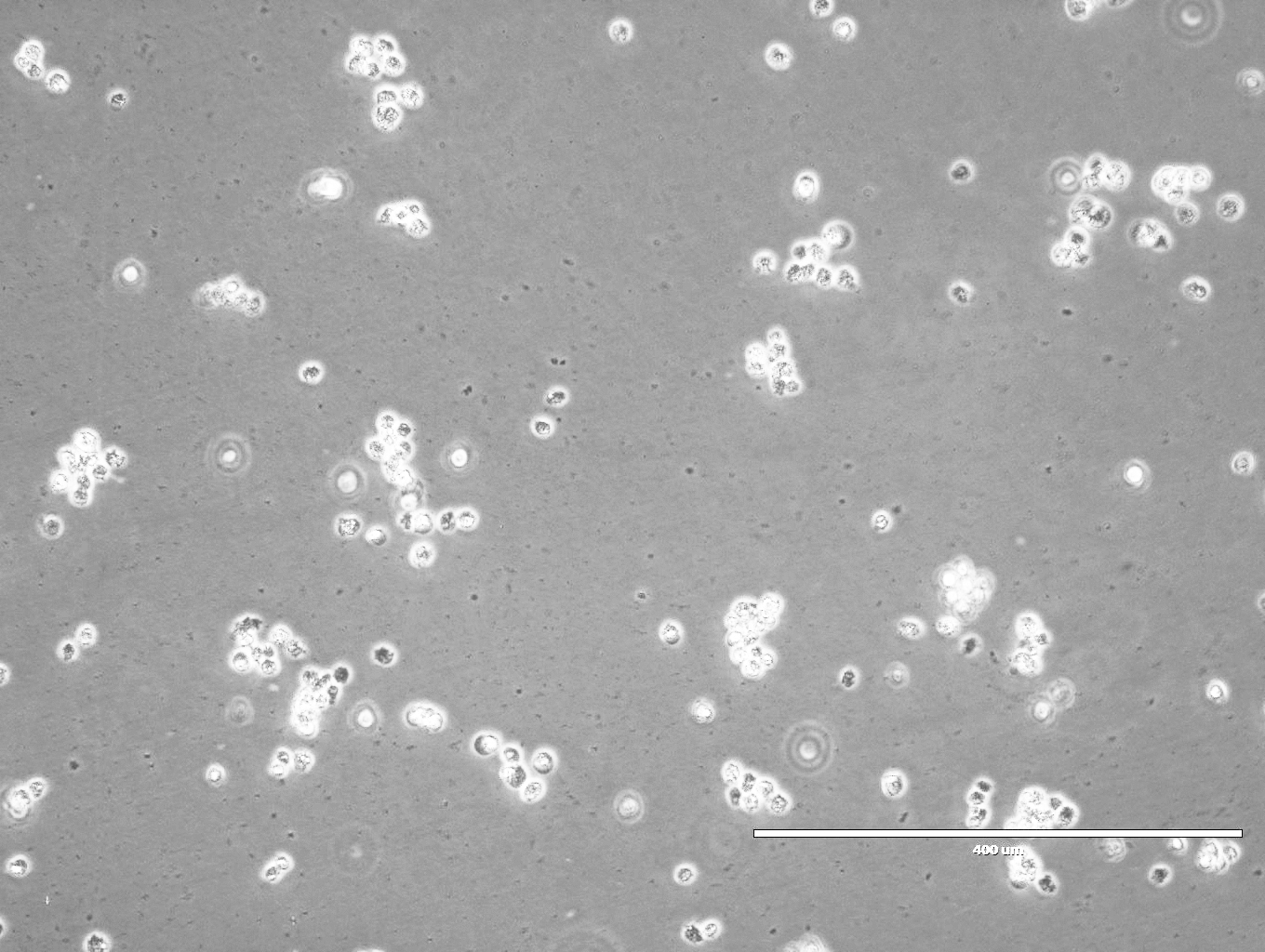

Supplement: Supplementary file 33 — Source data EV and Appendix [file 44318_2025_540_MOESM33_ESM.zip › Source data EV and Appendix/Figure EV 1/1D/Siramesine/MEF_12 h.jpg]

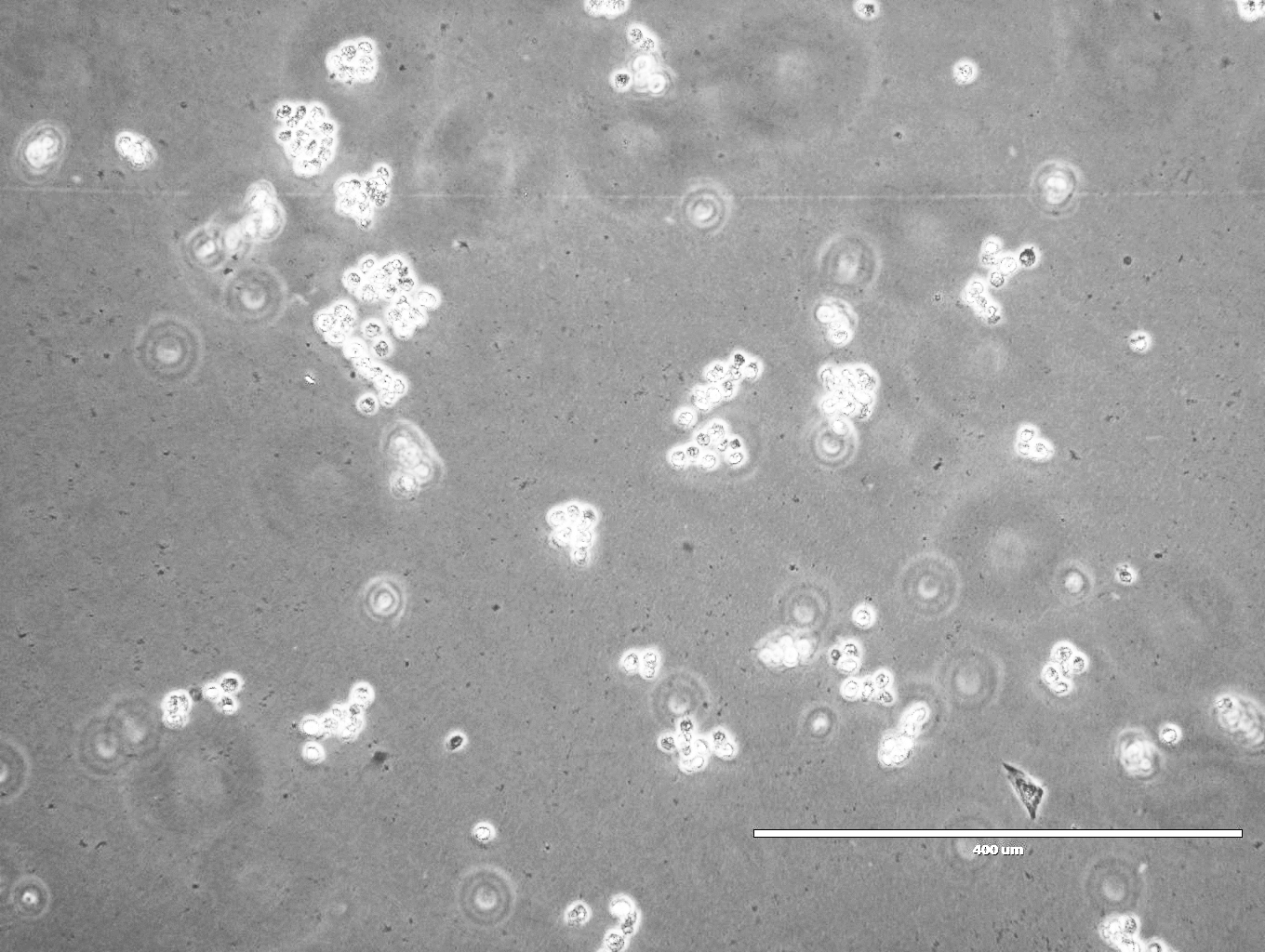

Supplement: Supplementary file 33 — Source data EV and Appendix [file 44318_2025_540_MOESM33_ESM.zip › Source data EV and Appendix/Figure EV 1/1D/Siramesine/MEF_24h.jpg]
