# Supplementary figures and images for: Programmed cell revival from imminent cell death enhances tissue repair and regeneration (part 4 of 4)
Source: EMBO J. 2025 Aug 21;44(19):5244–89. doi: 10.1038/s44318-025-00540-y (PMC12489119; doi:10.1038/s44318-025-00540-y)

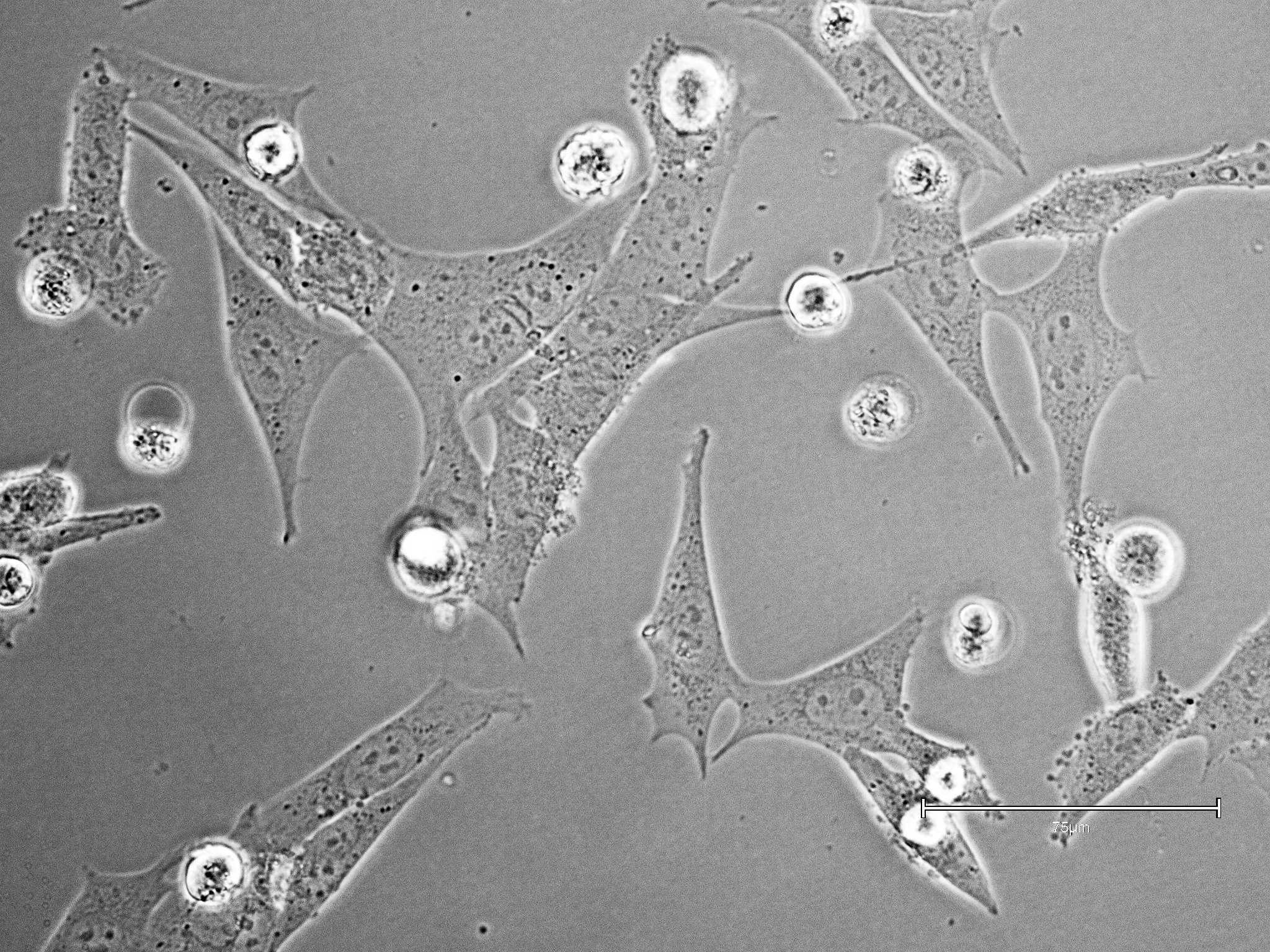

Supplement: Supplementary file 33 — Source data EV and Appendix [file 44318_2025_540_MOESM33_ESM.zip › Source data EV and Appendix/Figure EV 2/2N/80uM/24h.jpg]

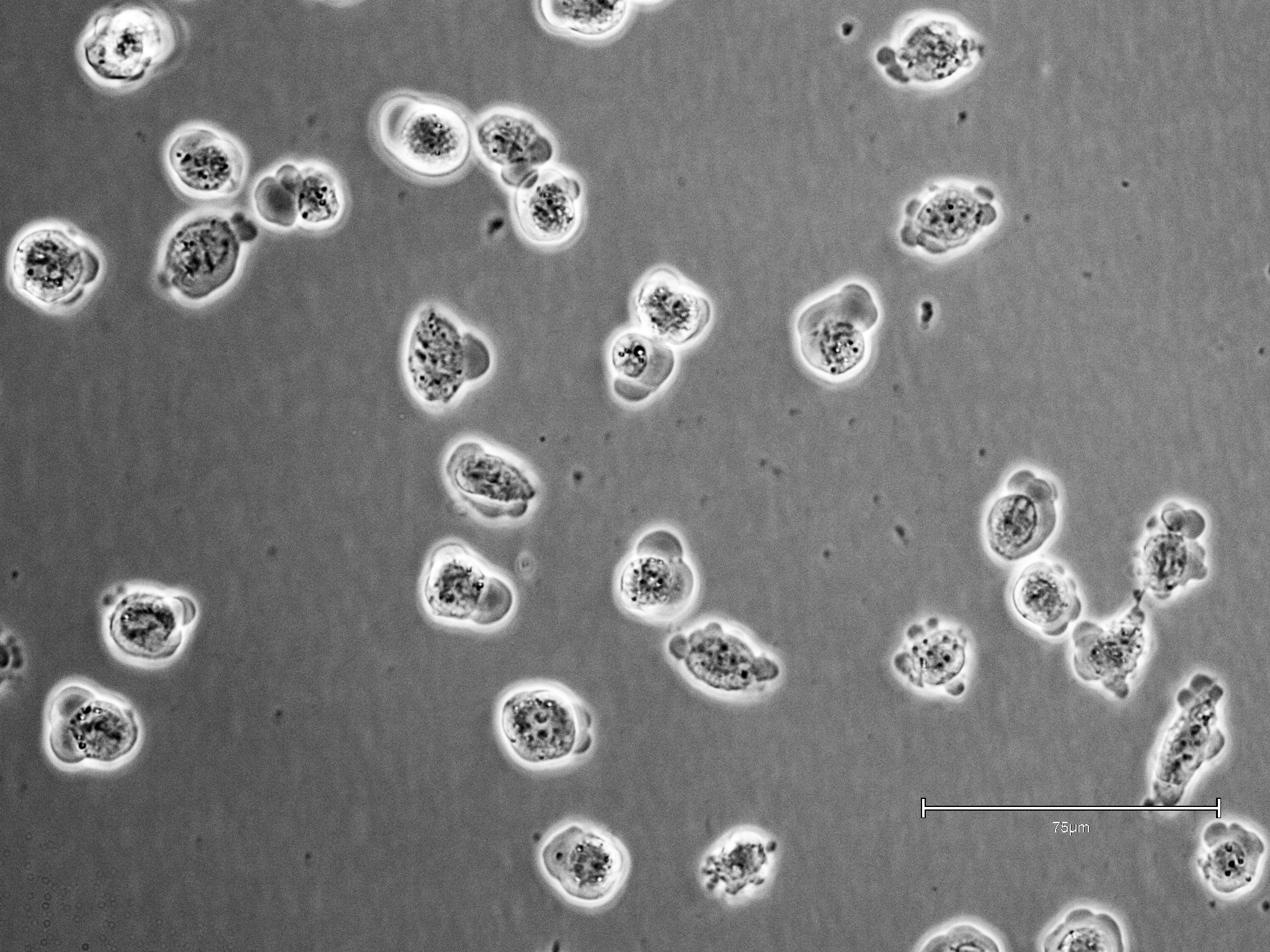

Supplement: Supplementary file 33 — Source data EV and Appendix [file 44318_2025_540_MOESM33_ESM.zip › Source data EV and Appendix/Figure EV 2/2N/80uM/30mins.tif]

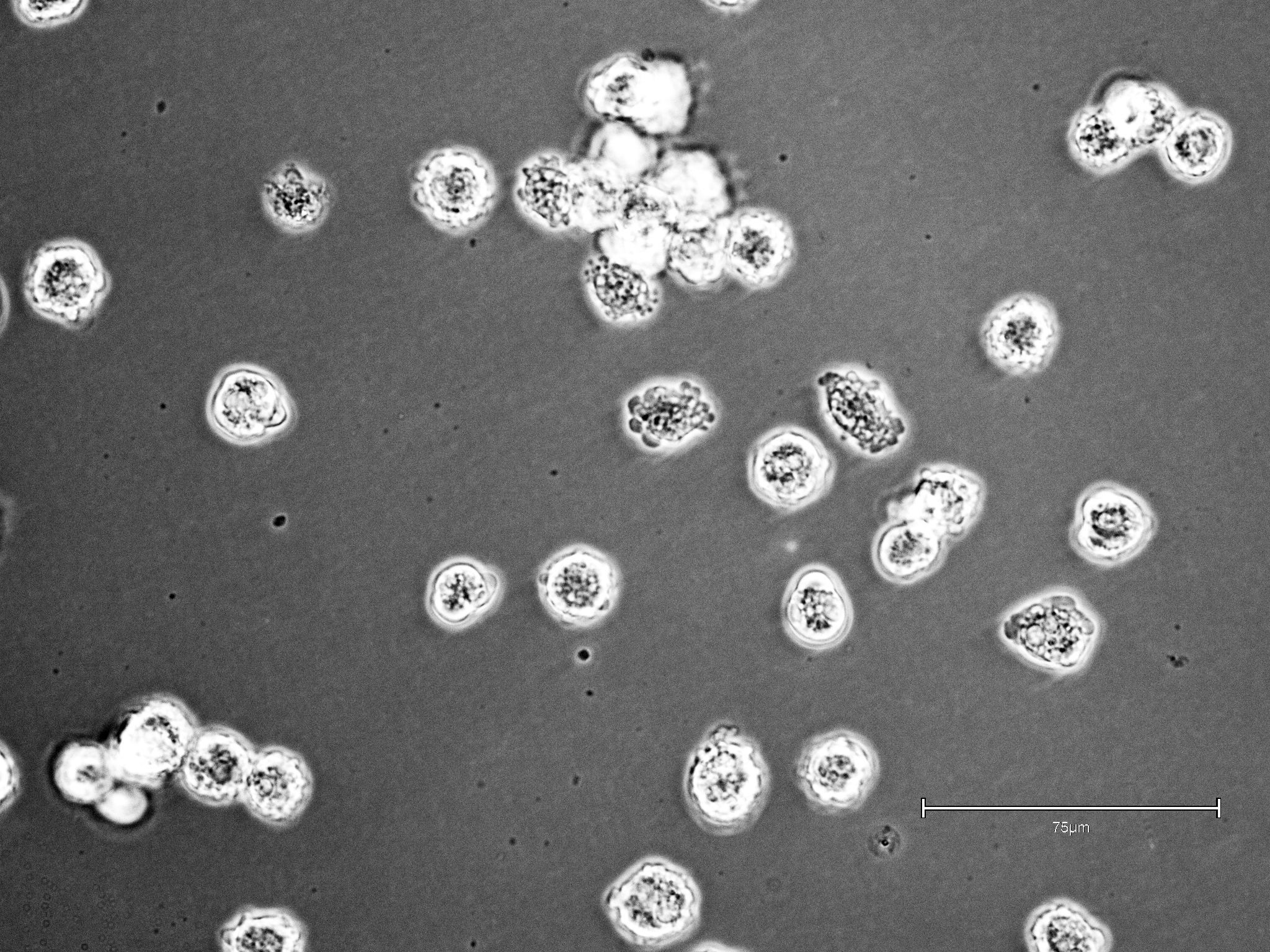

Supplement: Supplementary file 33 — Source data EV and Appendix [file 44318_2025_540_MOESM33_ESM.zip › Source data EV and Appendix/Figure EV 2/2N/80uM/3h.tif]

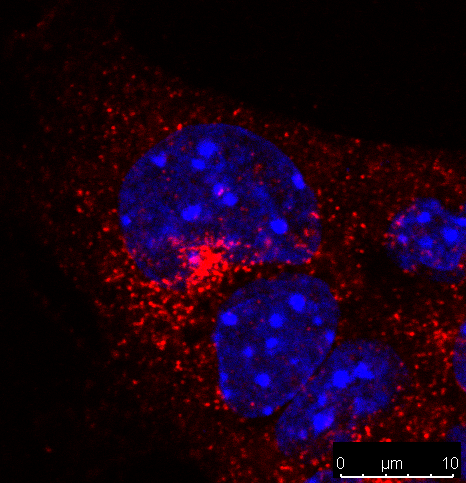

Supplement: Supplementary file 33 — Source data EV and Appendix [file 44318_2025_540_MOESM33_ESM.zip › Source data EV and Appendix/Figure EV 3/3A/16h.tif]

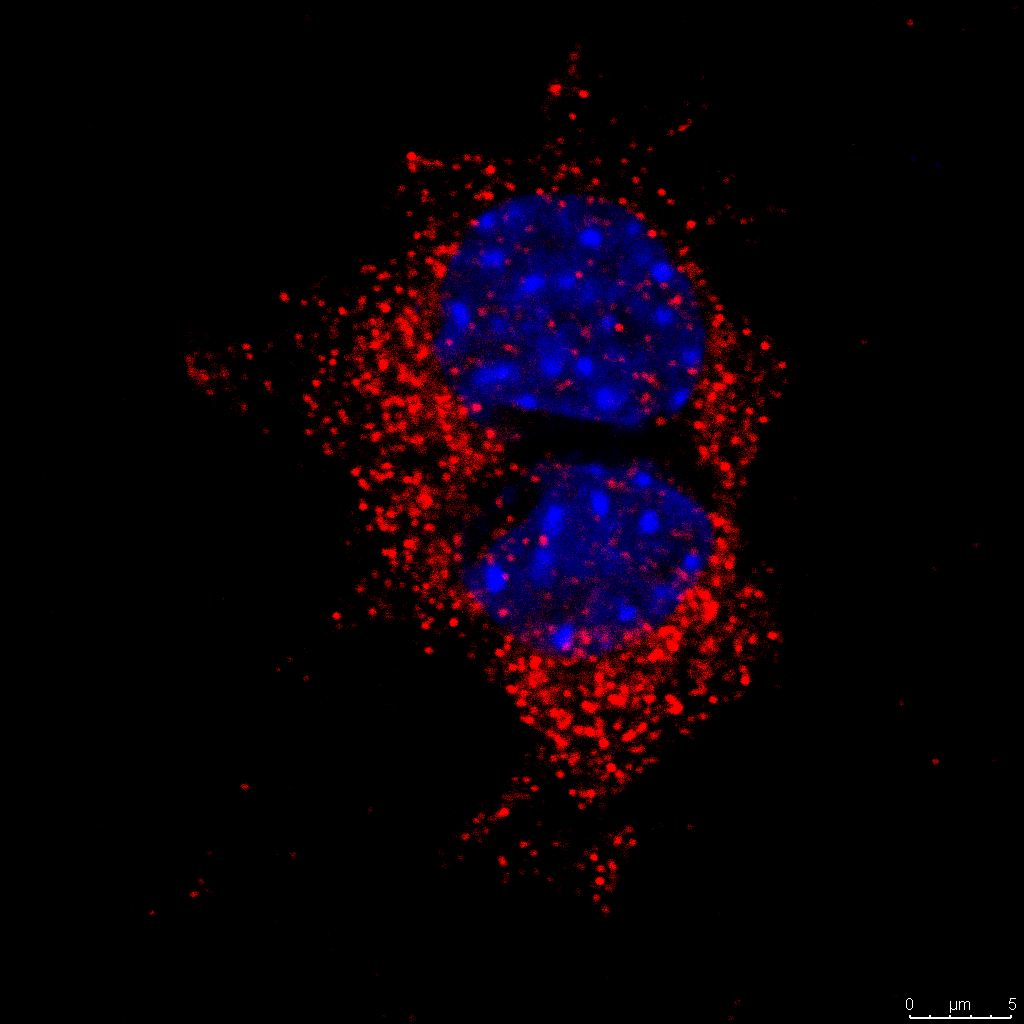

Supplement: Supplementary file 33 — Source data EV and Appendix [file 44318_2025_540_MOESM33_ESM.zip › Source data EV and Appendix/Figure EV 3/3A/2h 30 min.tif]

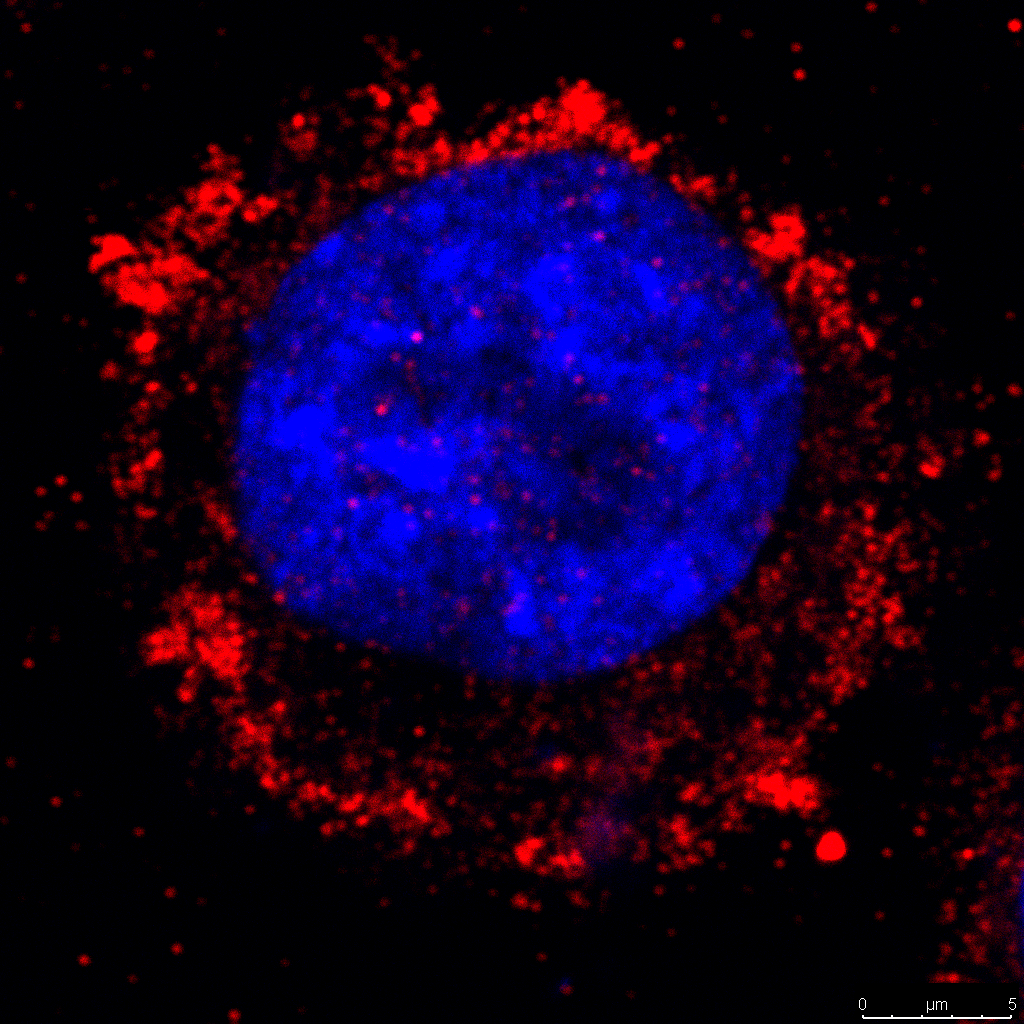

Supplement: Supplementary file 33 — Source data EV and Appendix [file 44318_2025_540_MOESM33_ESM.zip › Source data EV and Appendix/Figure EV 3/3A/30 min.tif]

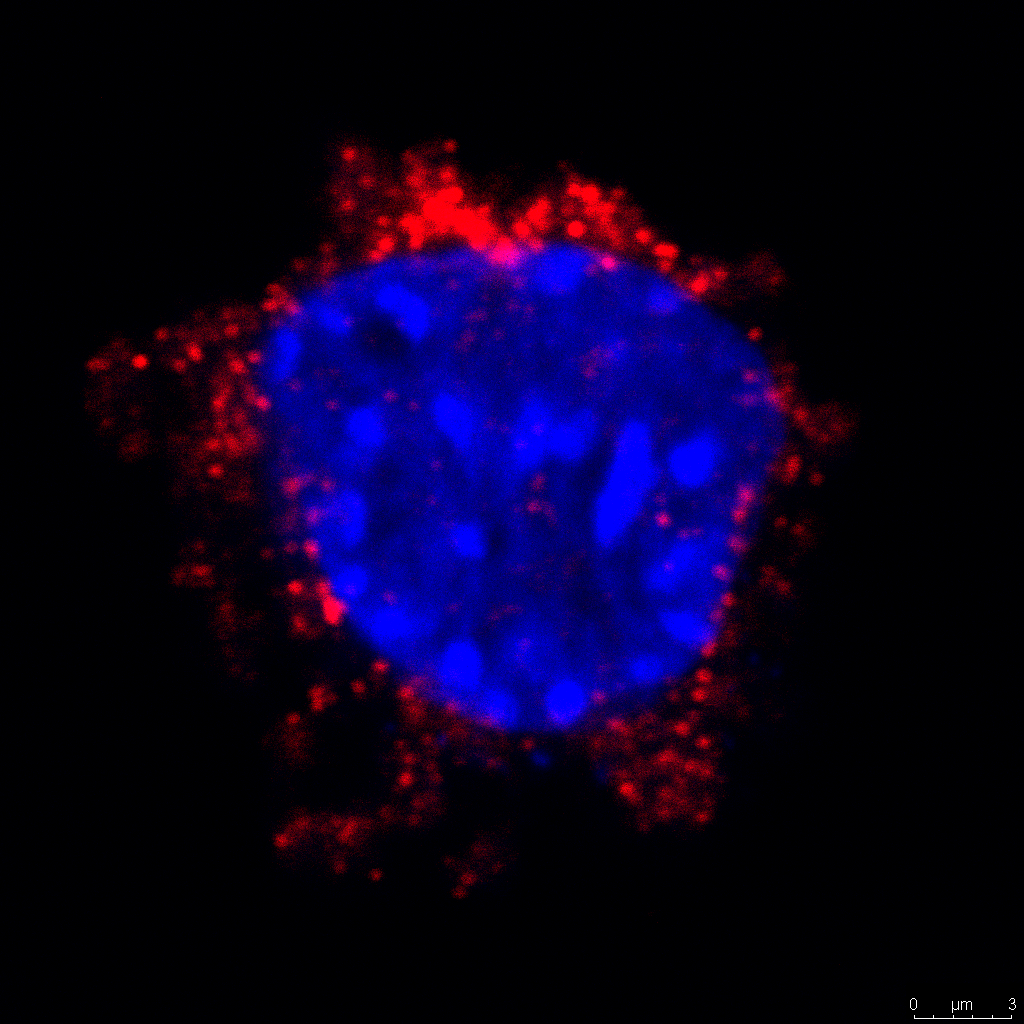

Supplement: Supplementary file 33 — Source data EV and Appendix [file 44318_2025_540_MOESM33_ESM.zip › Source data EV and Appendix/Figure EV 3/3A/5 min.tif]

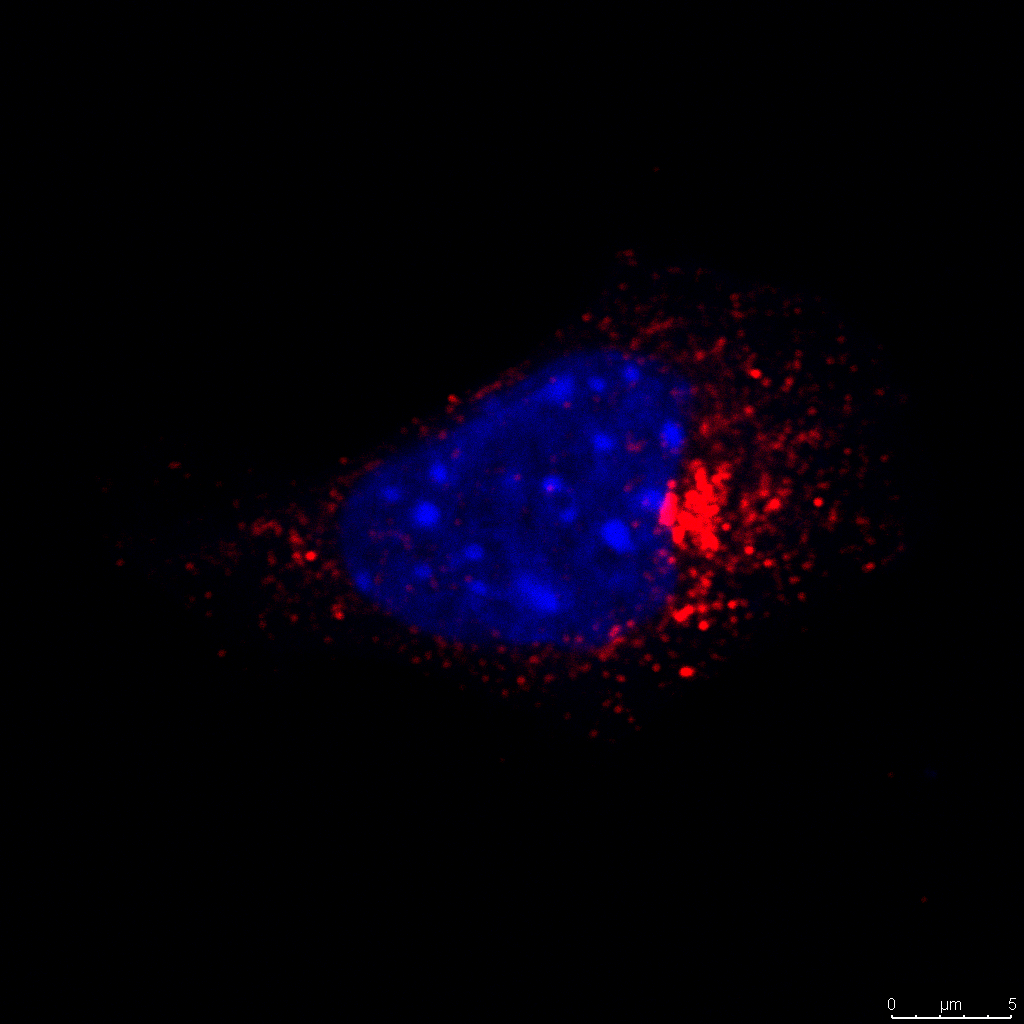

Supplement: Supplementary file 33 — Source data EV and Appendix [file 44318_2025_540_MOESM33_ESM.zip › Source data EV and Appendix/Figure EV 3/3A/Control.tif]

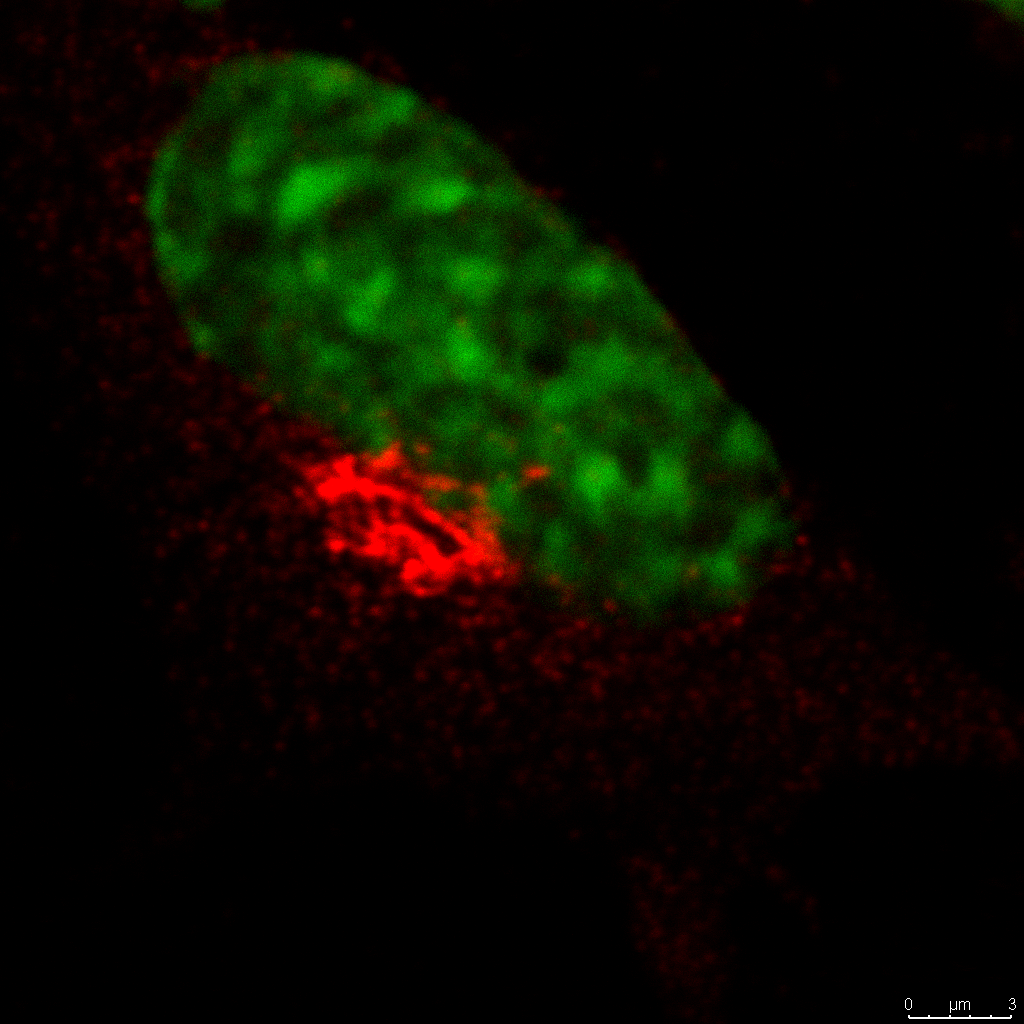

Supplement: Supplementary file 33 — Source data EV and Appendix [file 44318_2025_540_MOESM33_ESM.zip › Source data EV and Appendix/Figure EV 3/3B/16 h.tif]

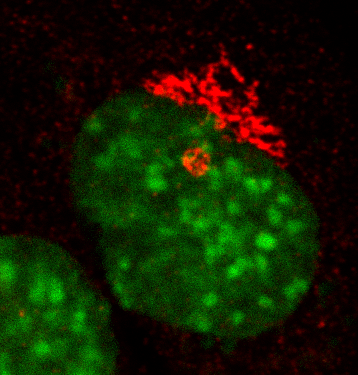

Supplement: Supplementary file 33 — Source data EV and Appendix [file 44318_2025_540_MOESM33_ESM.zip › Source data EV and Appendix/Figure EV 3/3B/16h-A.tif]

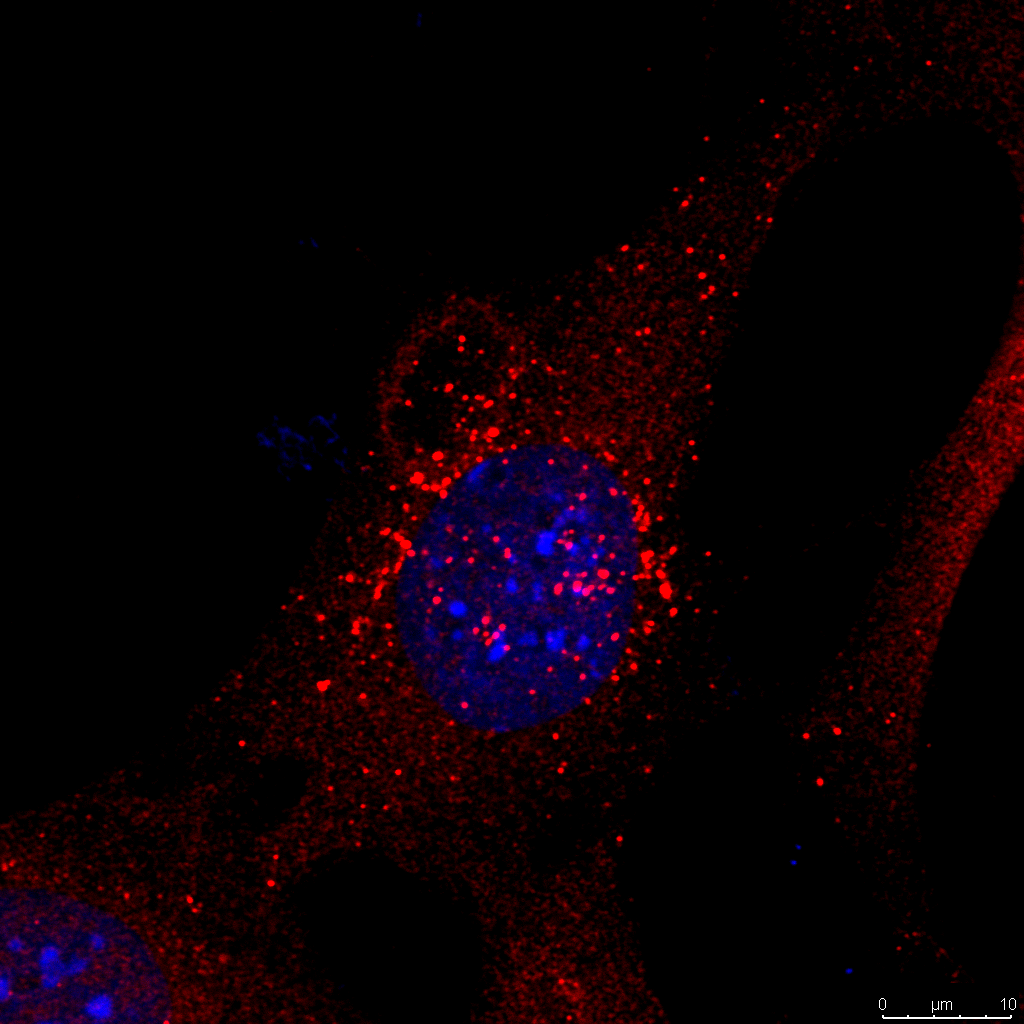

Supplement: Supplementary file 33 — Source data EV and Appendix [file 44318_2025_540_MOESM33_ESM.zip › Source data EV and Appendix/Figure EV 3/3C/16 h.tif]

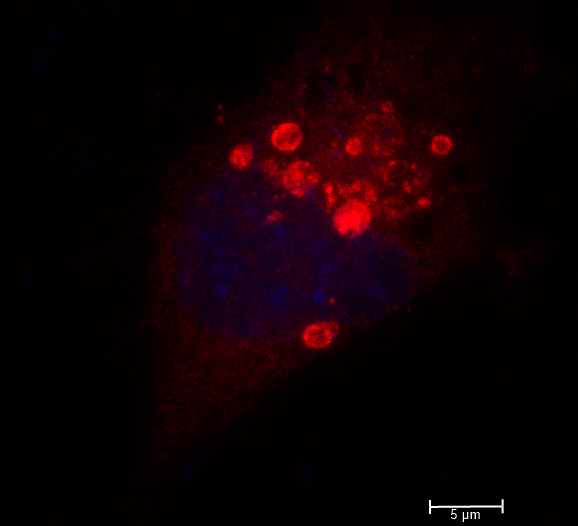

Supplement: Supplementary file 33 — Source data EV and Appendix [file 44318_2025_540_MOESM33_ESM.zip › Source data EV and Appendix/Figure EV 3/3C/2 h 30 min.tif]

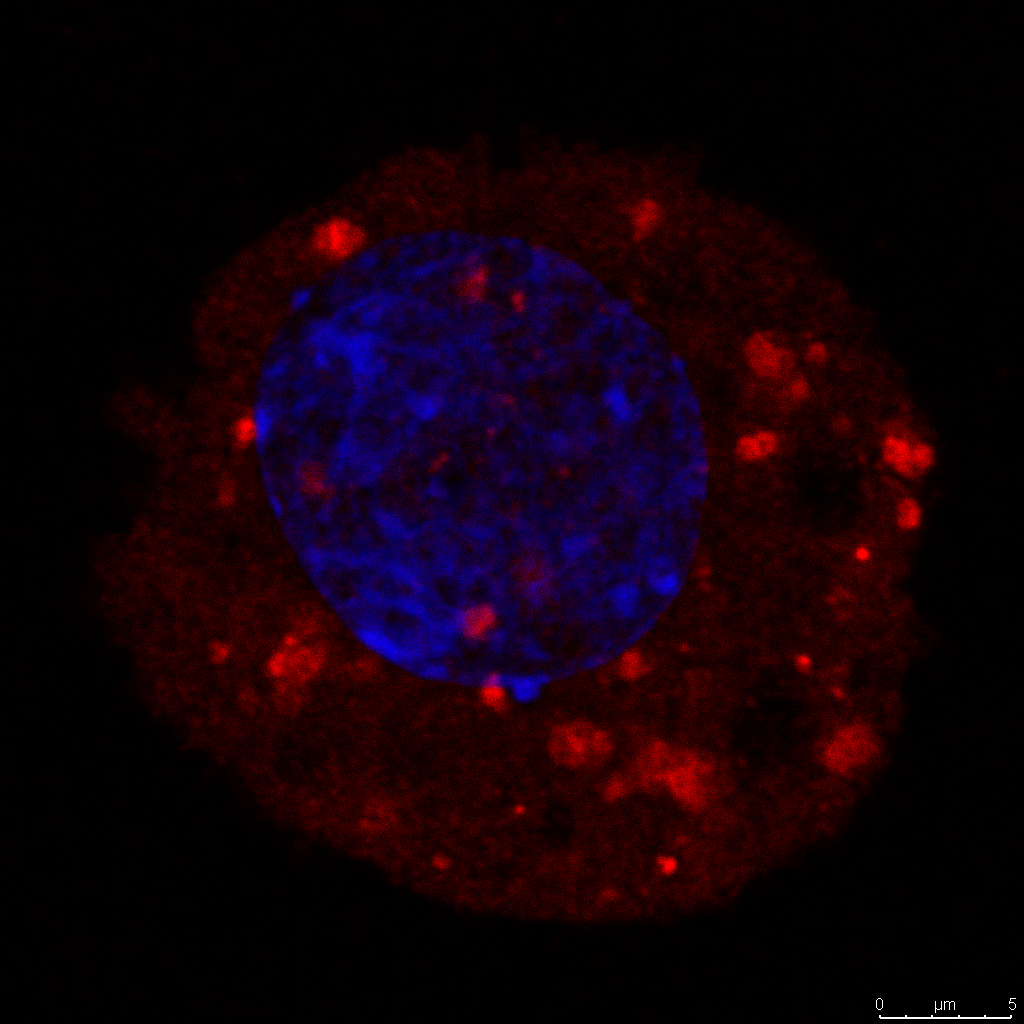

Supplement: Supplementary file 33 — Source data EV and Appendix [file 44318_2025_540_MOESM33_ESM.zip › Source data EV and Appendix/Figure EV 3/3C/30 min.tif]

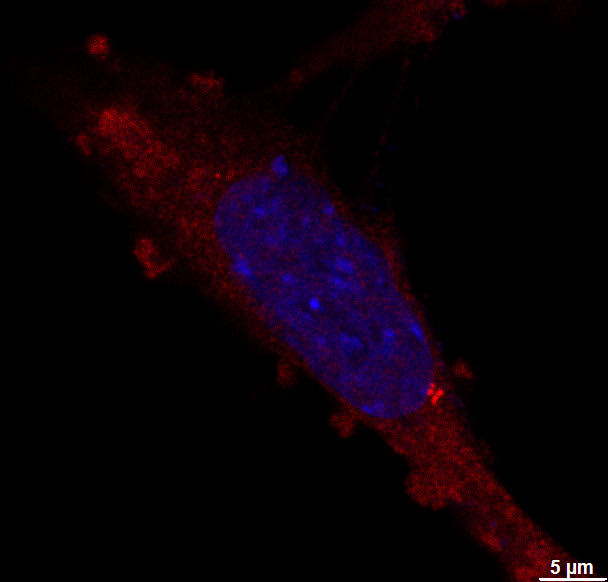

Supplement: Supplementary file 33 — Source data EV and Appendix [file 44318_2025_540_MOESM33_ESM.zip › Source data EV and Appendix/Figure EV 3/3C/5min.tif]

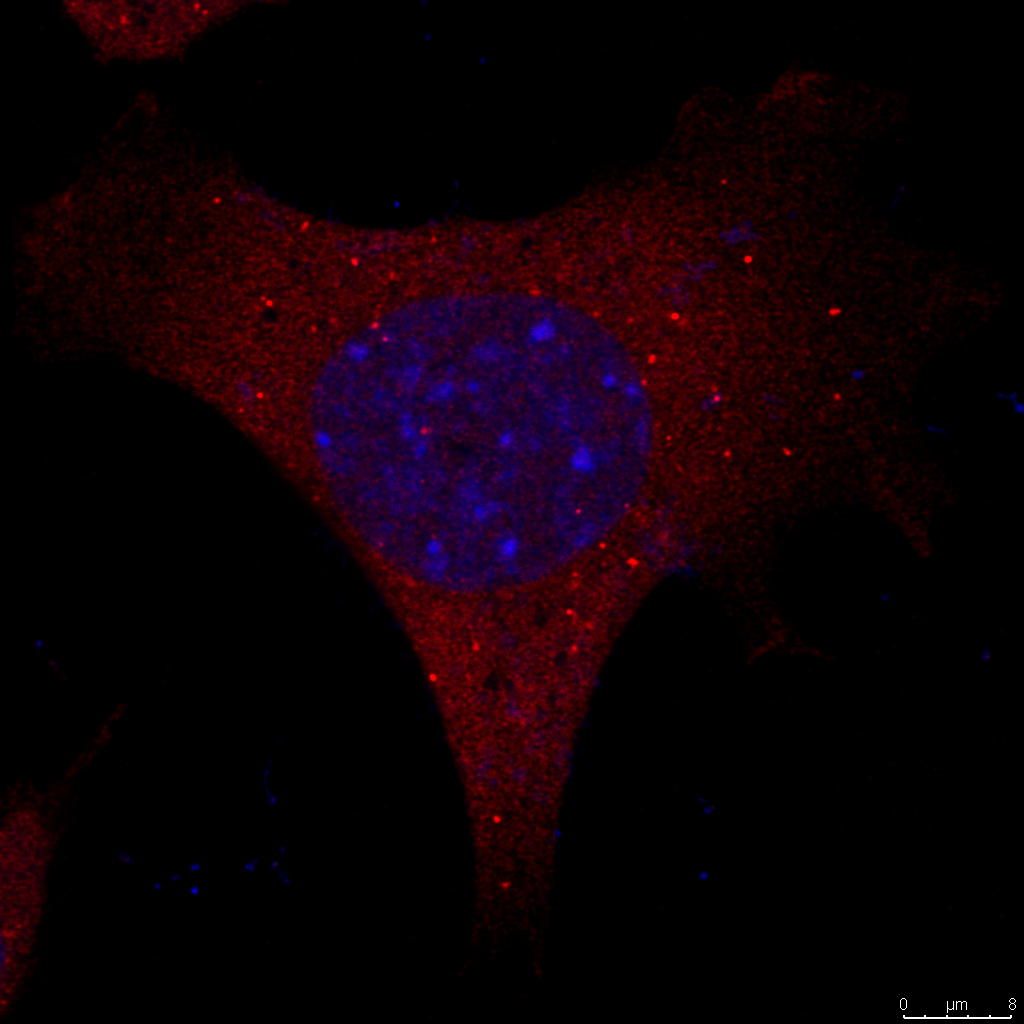

Supplement: Supplementary file 33 — Source data EV and Appendix [file 44318_2025_540_MOESM33_ESM.zip › Source data EV and Appendix/Figure EV 3/3C/Control.tif]

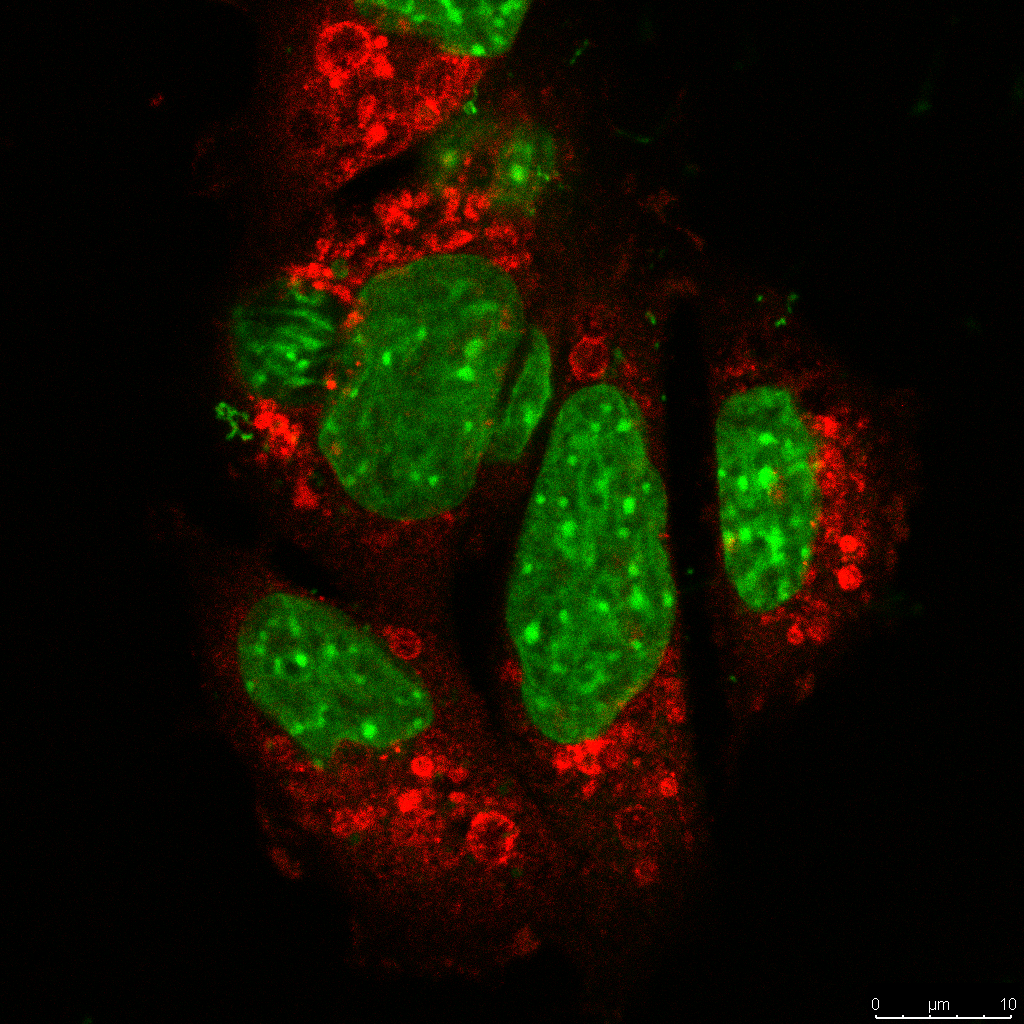

Supplement: Supplementary file 33 — Source data EV and Appendix [file 44318_2025_540_MOESM33_ESM.zip › Source data EV and Appendix/Figure EV 3/3D/Autophagosome_2 h 30 min.tif]

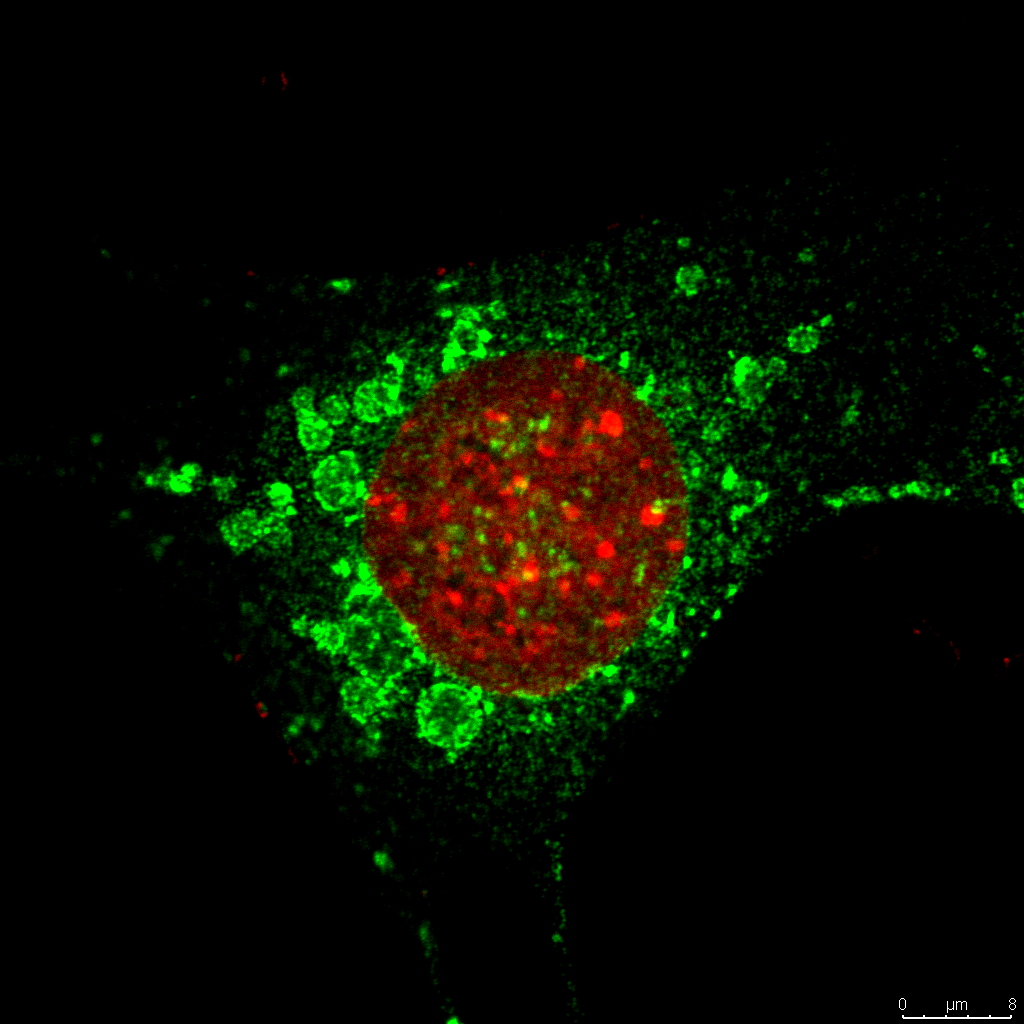

Supplement: Supplementary file 33 — Source data EV and Appendix [file 44318_2025_540_MOESM33_ESM.zip › Source data EV and Appendix/Figure EV 3/3E/Late Endosome_16h.tif]

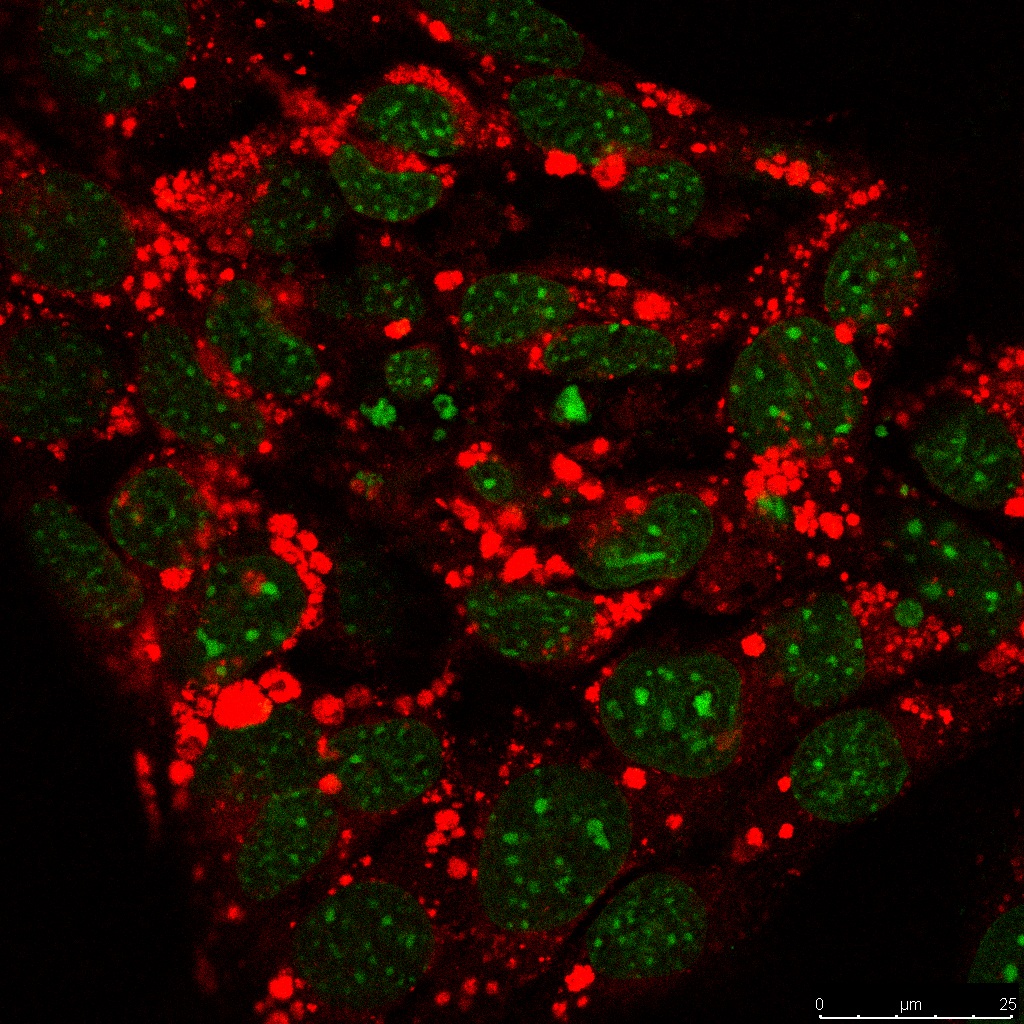

Supplement: Supplementary file 33 — Source data EV and Appendix [file 44318_2025_540_MOESM33_ESM.zip › Source data EV and Appendix/Figure EV 3/3F/Lysosome_16h.tif]

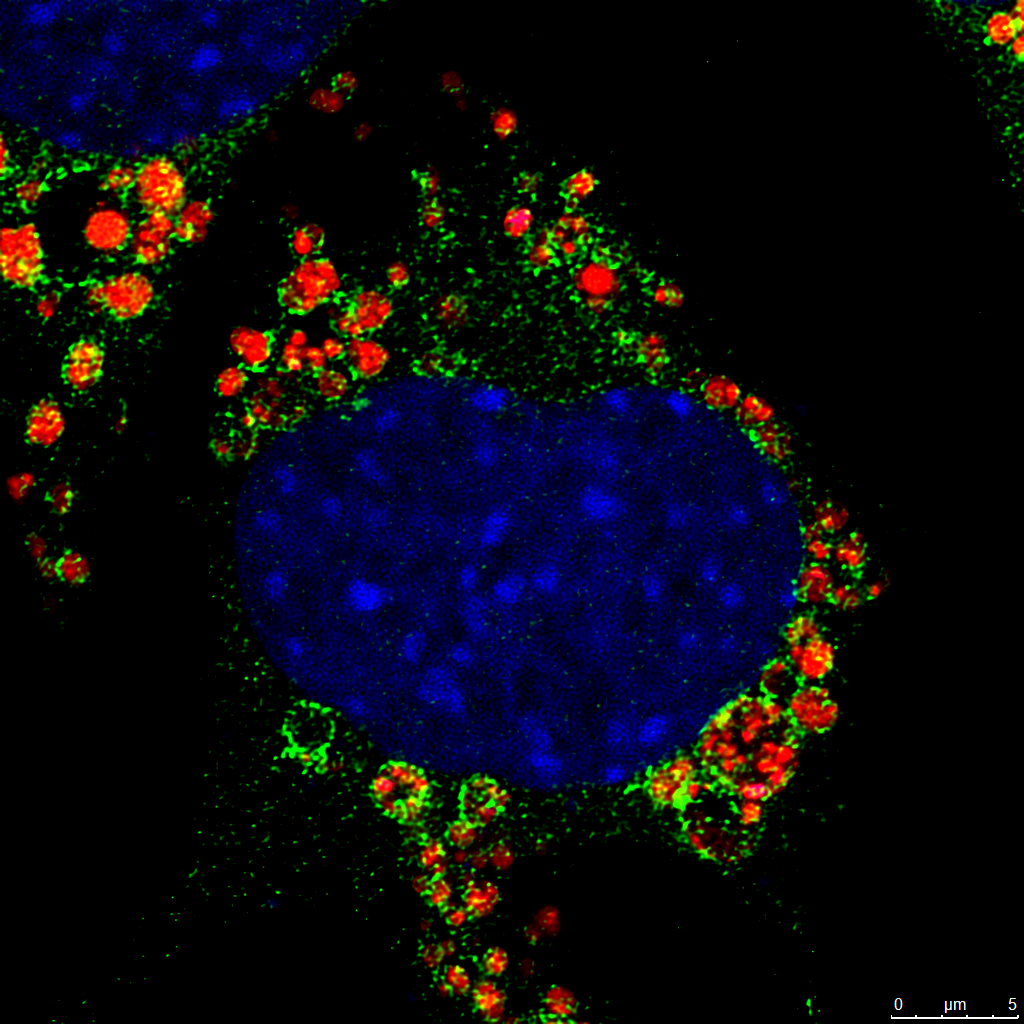

Supplement: Supplementary file 33 — Source data EV and Appendix [file 44318_2025_540_MOESM33_ESM.zip › Source data EV and Appendix/Figure EV 3/3G/Lysotracker Red+ Rab 7_6h.tif]

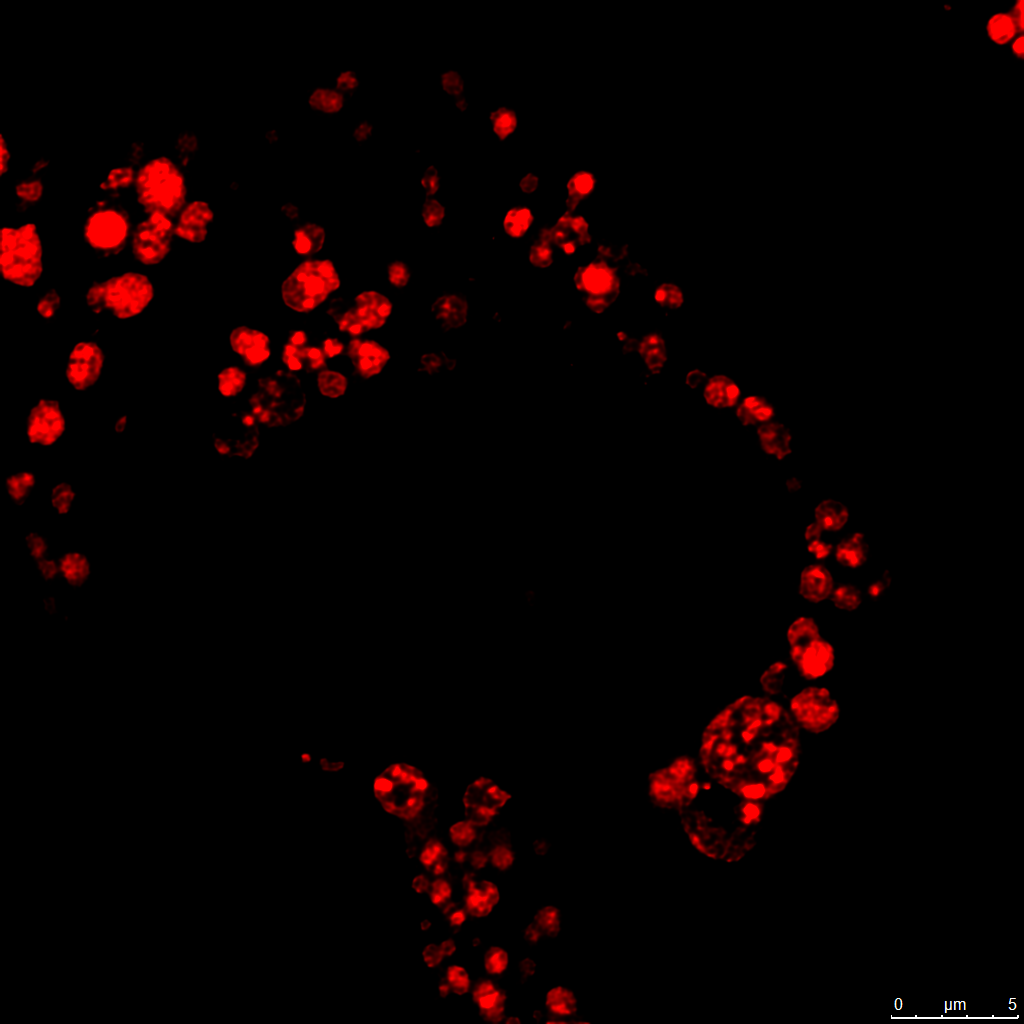

Supplement: Supplementary file 33 — Source data EV and Appendix [file 44318_2025_540_MOESM33_ESM.zip › Source data EV and Appendix/Figure EV 3/3G/Lysotracker_6h.tif]

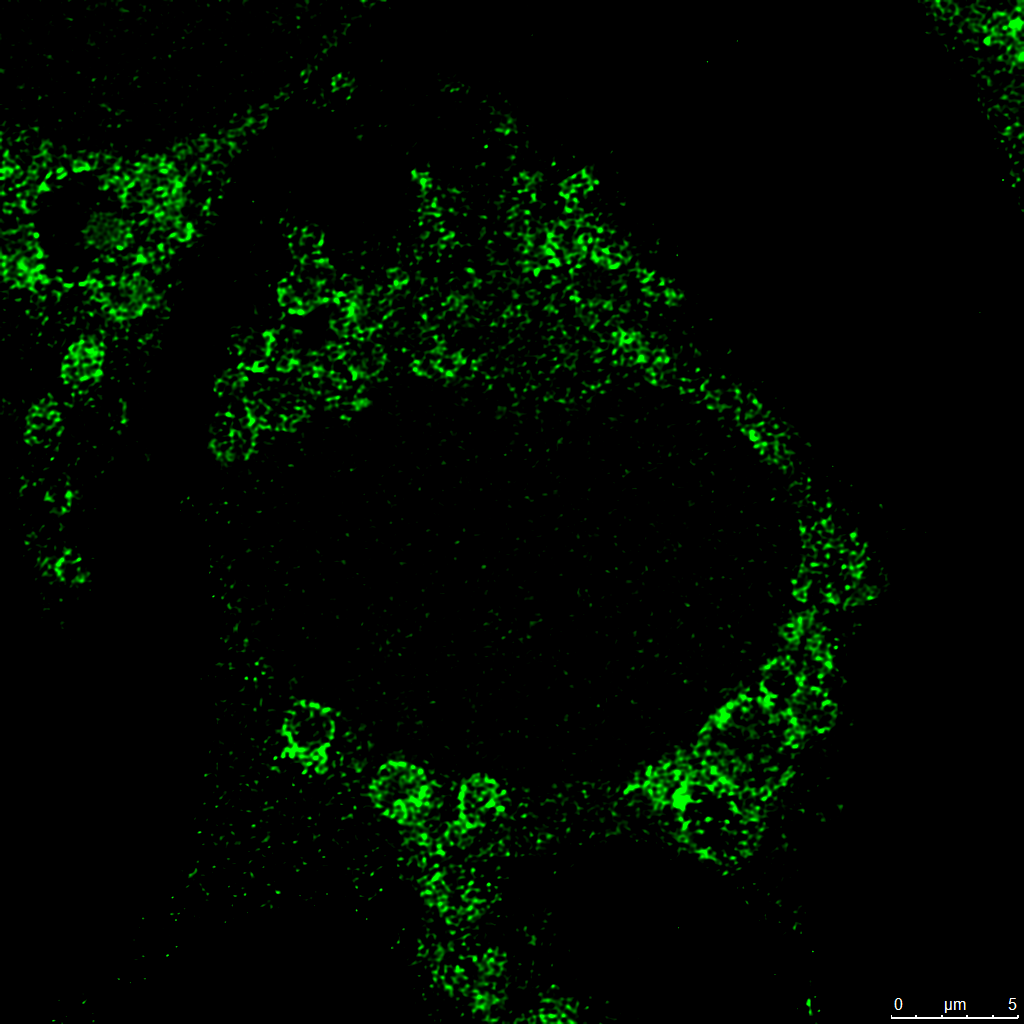

Supplement: Supplementary file 33 — Source data EV and Appendix [file 44318_2025_540_MOESM33_ESM.zip › Source data EV and Appendix/Figure EV 3/3G/Rab 7_6h.tif]

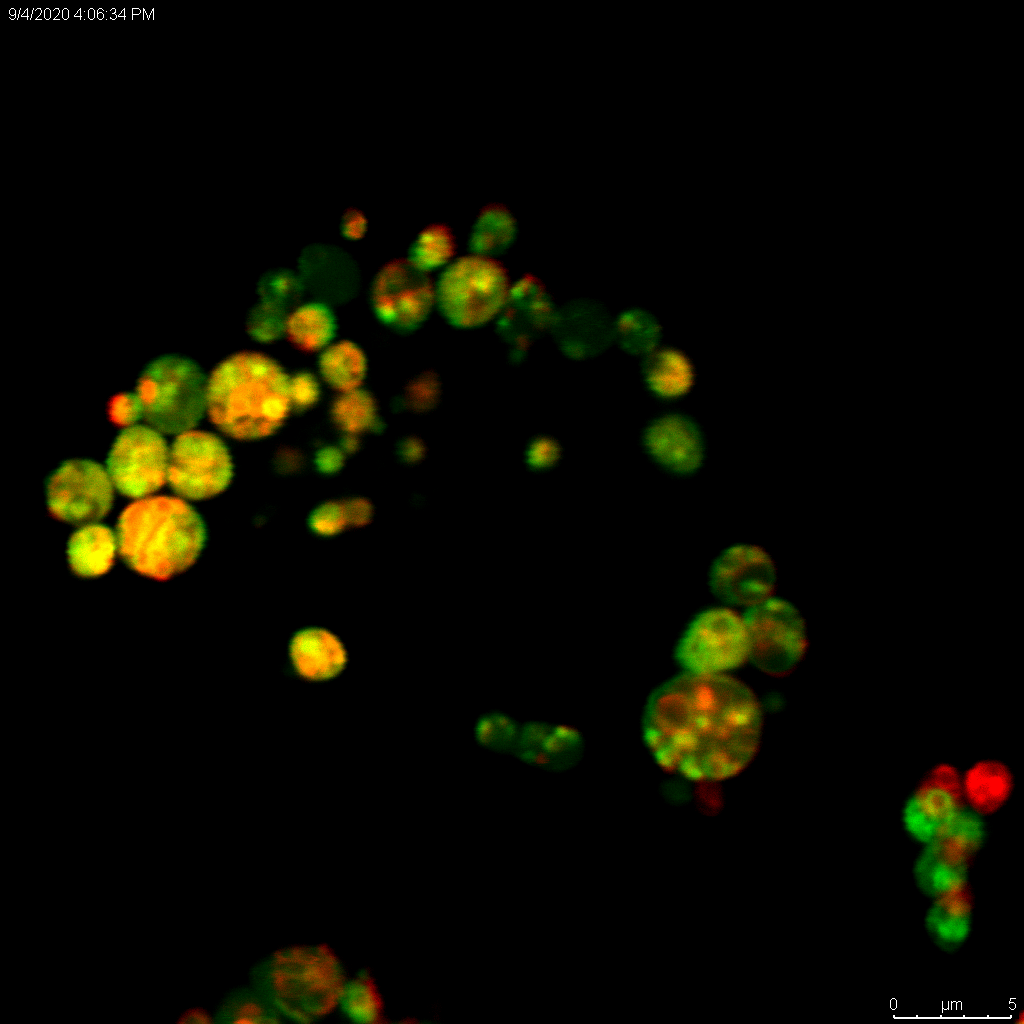

Supplement: Supplementary file 33 — Source data EV and Appendix [file 44318_2025_540_MOESM33_ESM.zip › Source data EV and Appendix/Figure EV 3/3H/Lysotracker Green+Magic Red_6h.tif]

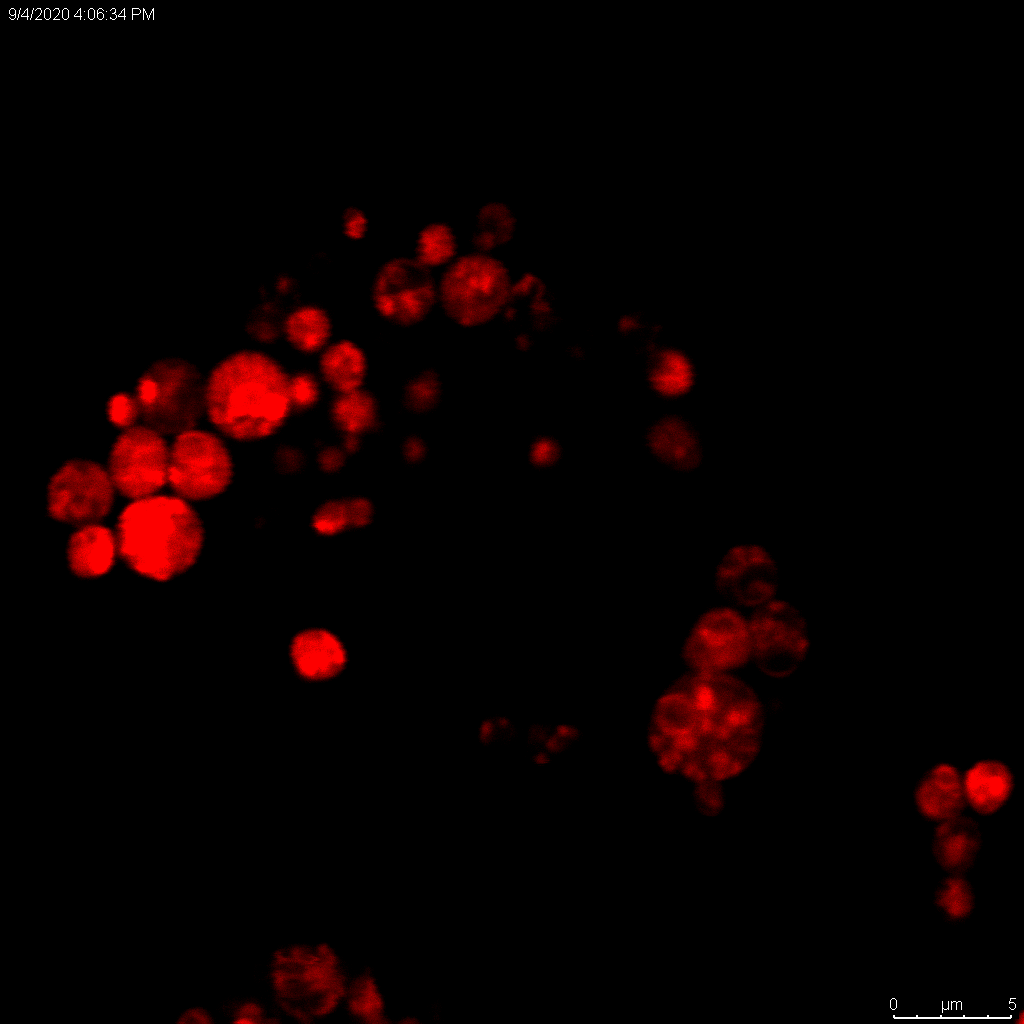

Supplement: Supplementary file 33 — Source data EV and Appendix [file 44318_2025_540_MOESM33_ESM.zip › Source data EV and Appendix/Figure EV 3/3H/Lysotracker Green_6h.tif]

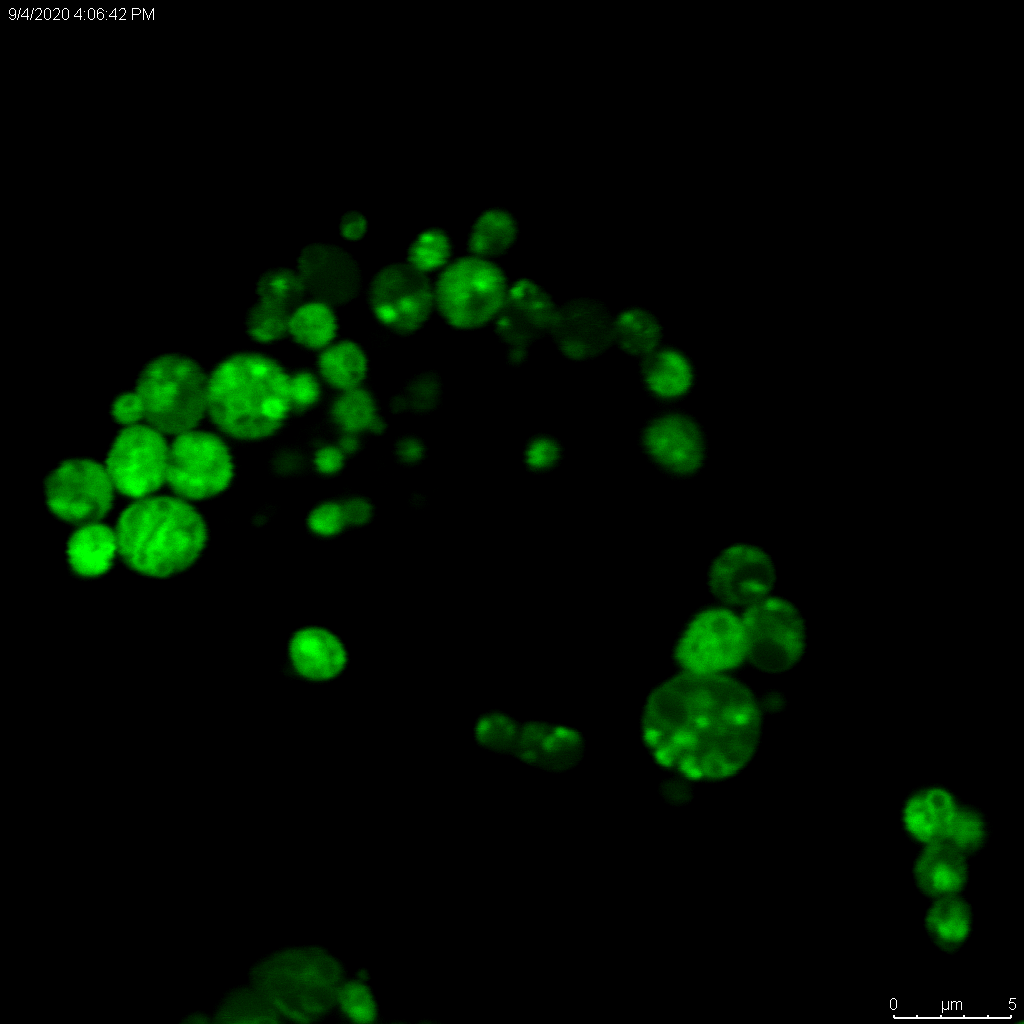

Supplement: Supplementary file 33 — Source data EV and Appendix [file 44318_2025_540_MOESM33_ESM.zip › Source data EV and Appendix/Figure EV 3/3H/Magic Red_6h.tif]

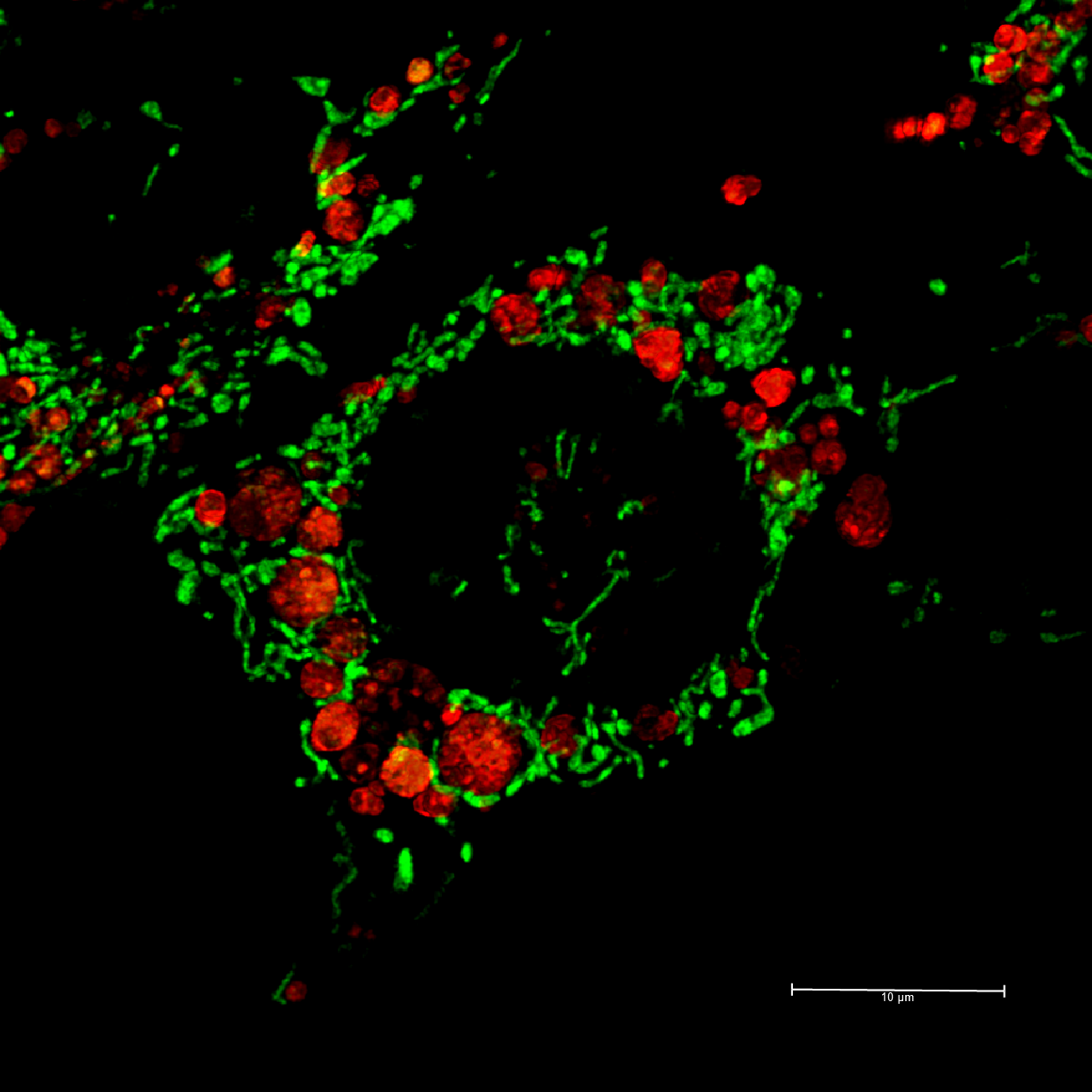

Supplement: Supplementary file 33 — Source data EV and Appendix [file 44318_2025_540_MOESM33_ESM.zip › Source data EV and Appendix/Figure EV 3/3I/Mitotracker Green+Lysotracker Red.tif]

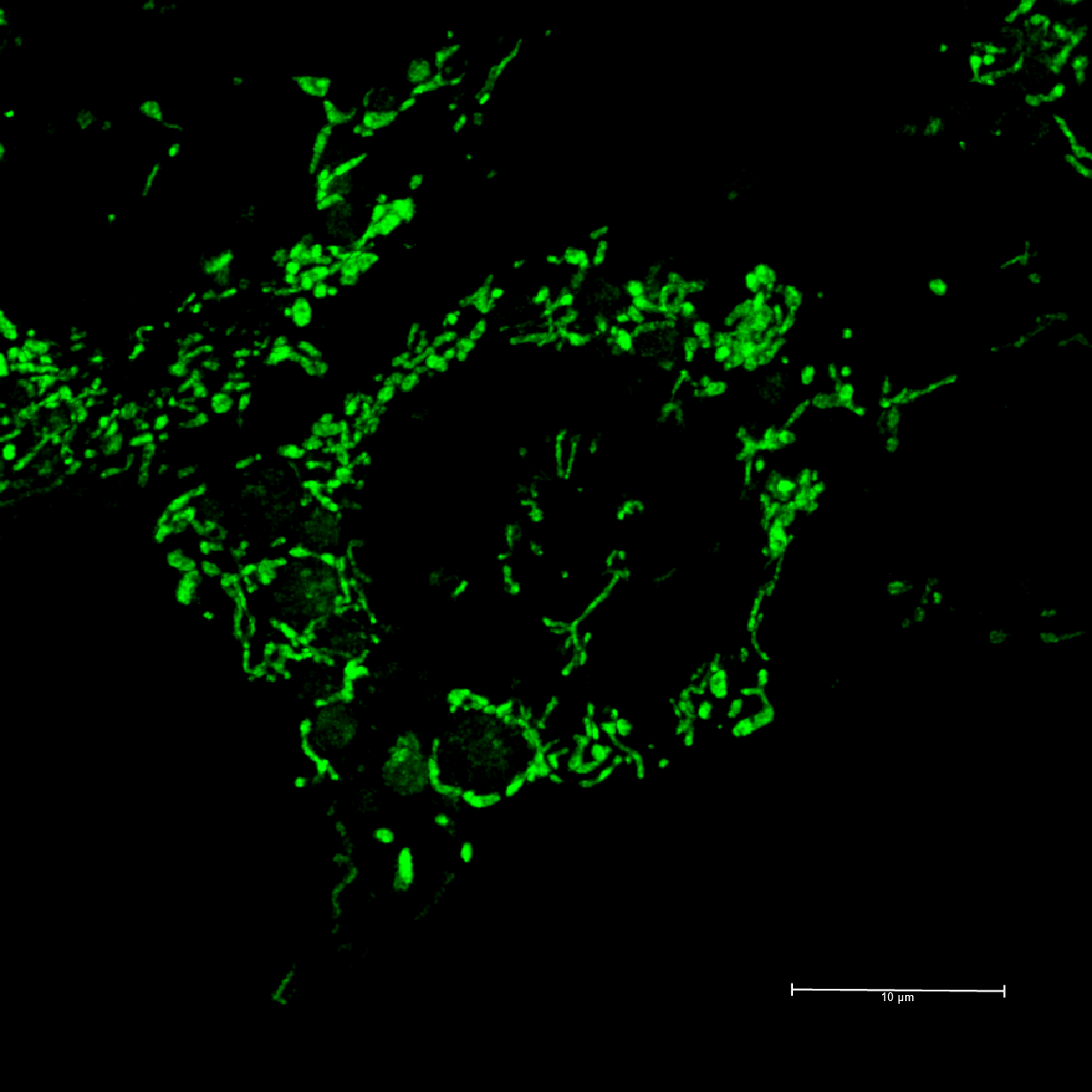

Supplement: Supplementary file 33 — Source data EV and Appendix [file 44318_2025_540_MOESM33_ESM.zip › Source data EV and Appendix/Figure EV 3/3I/Mitotracker Green.tif]

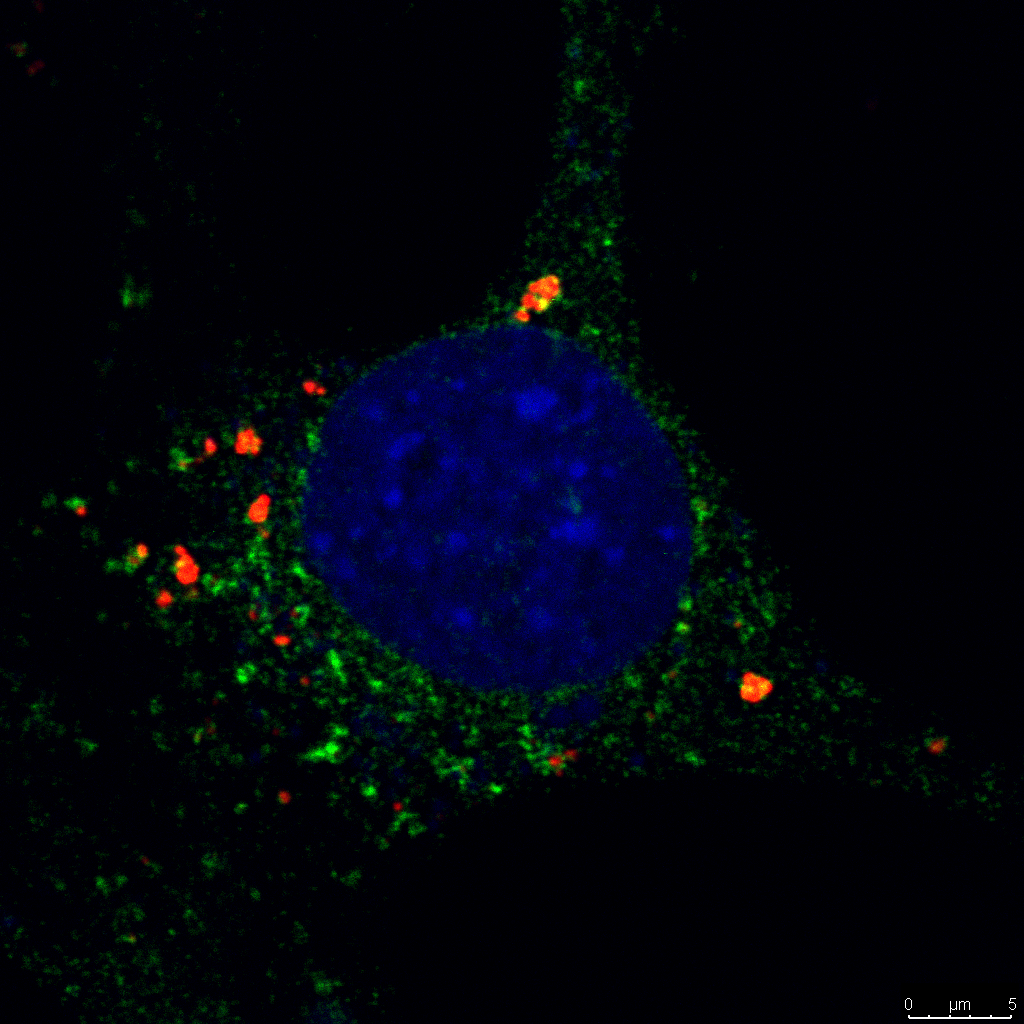

Supplement: Supplementary file 33 — Source data EV and Appendix [file 44318_2025_540_MOESM33_ESM.zip › Source data EV and Appendix/Figure EV 3/3J/Lysotracker Red+Rab 7_24h_1.tif]

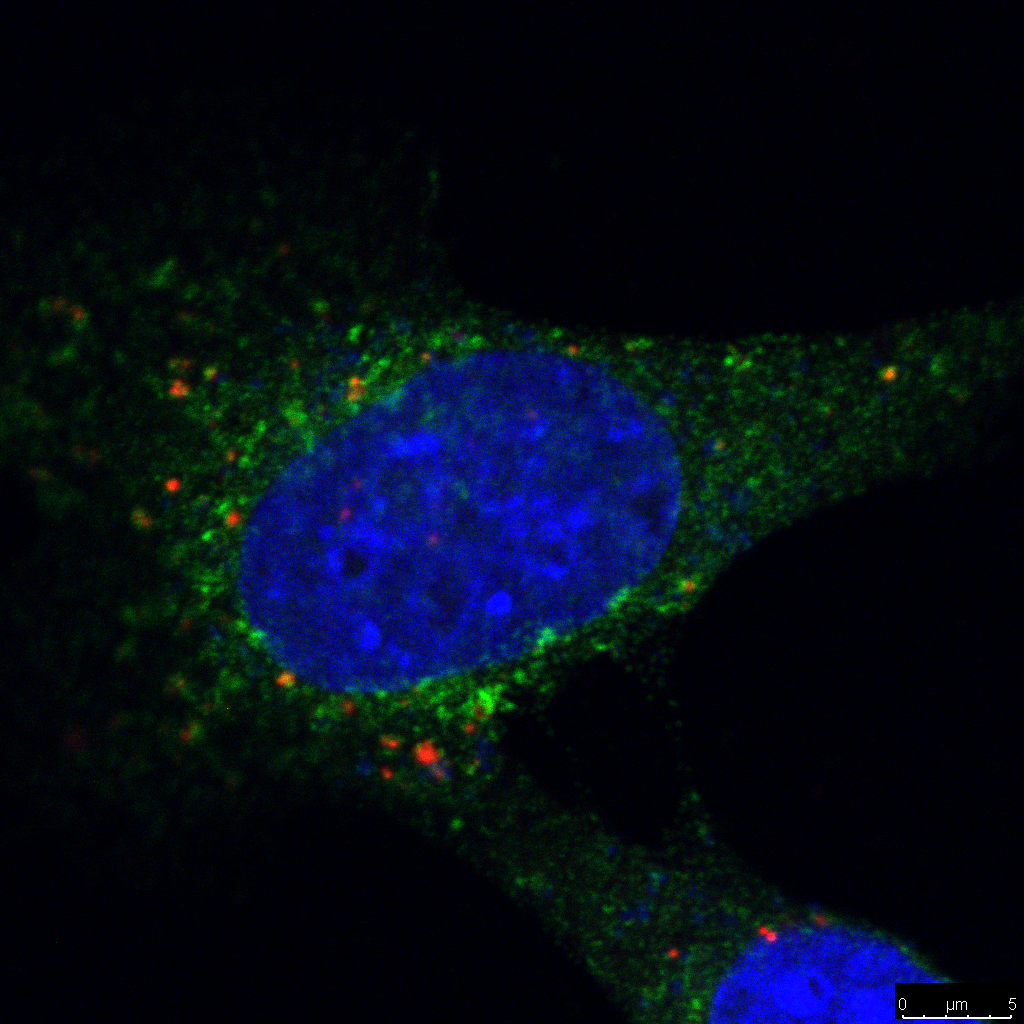

Supplement: Supplementary file 33 — Source data EV and Appendix [file 44318_2025_540_MOESM33_ESM.zip › Source data EV and Appendix/Figure EV 3/3J/Lysotracker Red+Rab 7_24h_2.tif]

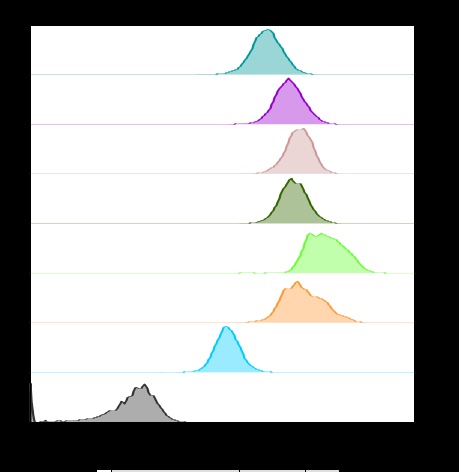

Supplement: Supplementary file 33 — Source data EV and Appendix [file 44318_2025_540_MOESM33_ESM.zip › Source data EV and Appendix/Figure EV 3/3K/Cell Rox.jpg]

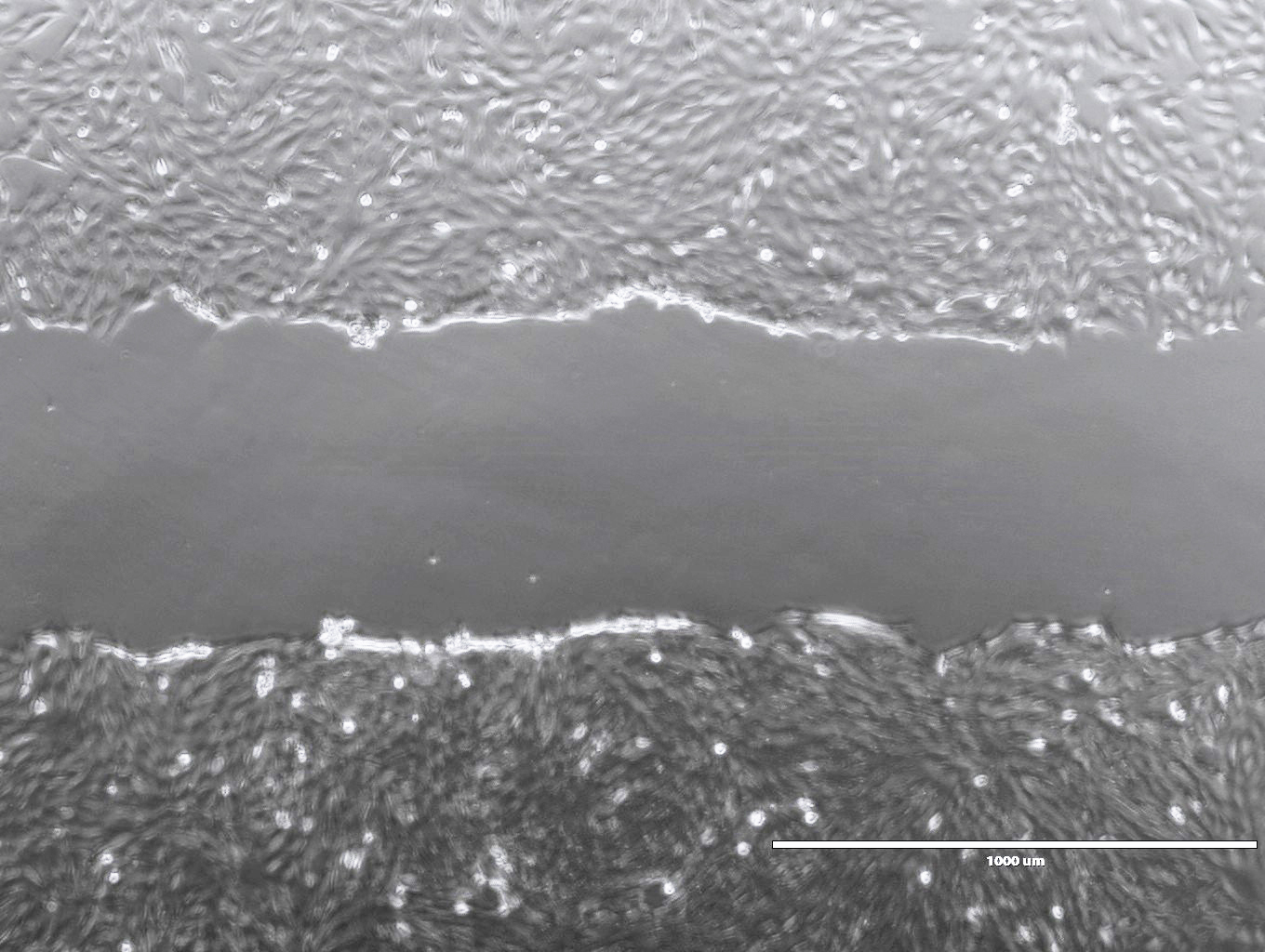

Supplement: Supplementary file 33 — Source data EV and Appendix [file 44318_2025_540_MOESM33_ESM.zip › Source data EV and Appendix/Figure EV 6/6B/Control/0 h.jpg]

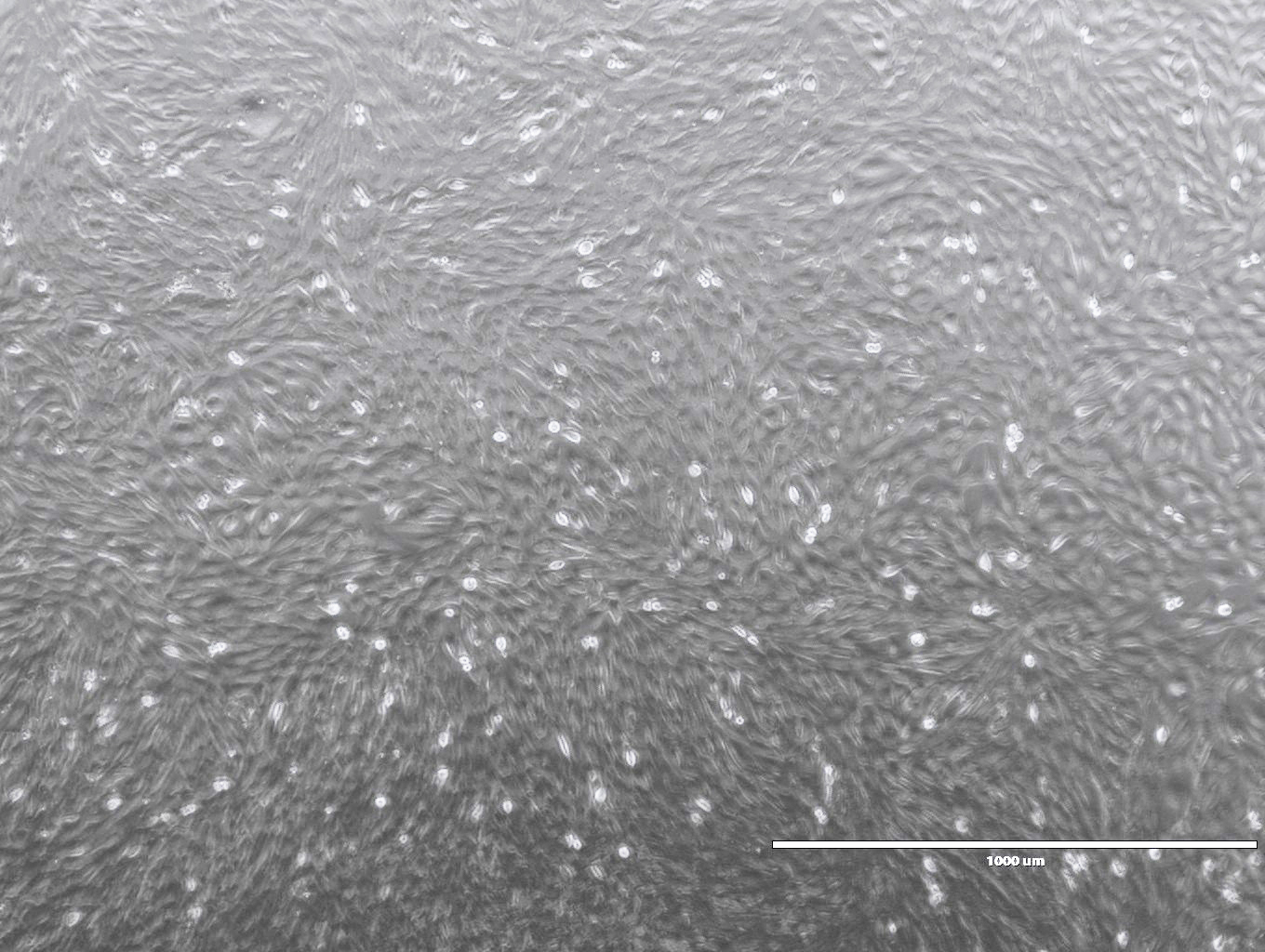

Supplement: Supplementary file 33 — Source data EV and Appendix [file 44318_2025_540_MOESM33_ESM.zip › Source data EV and Appendix/Figure EV 6/6B/Control/12 h.jpg]

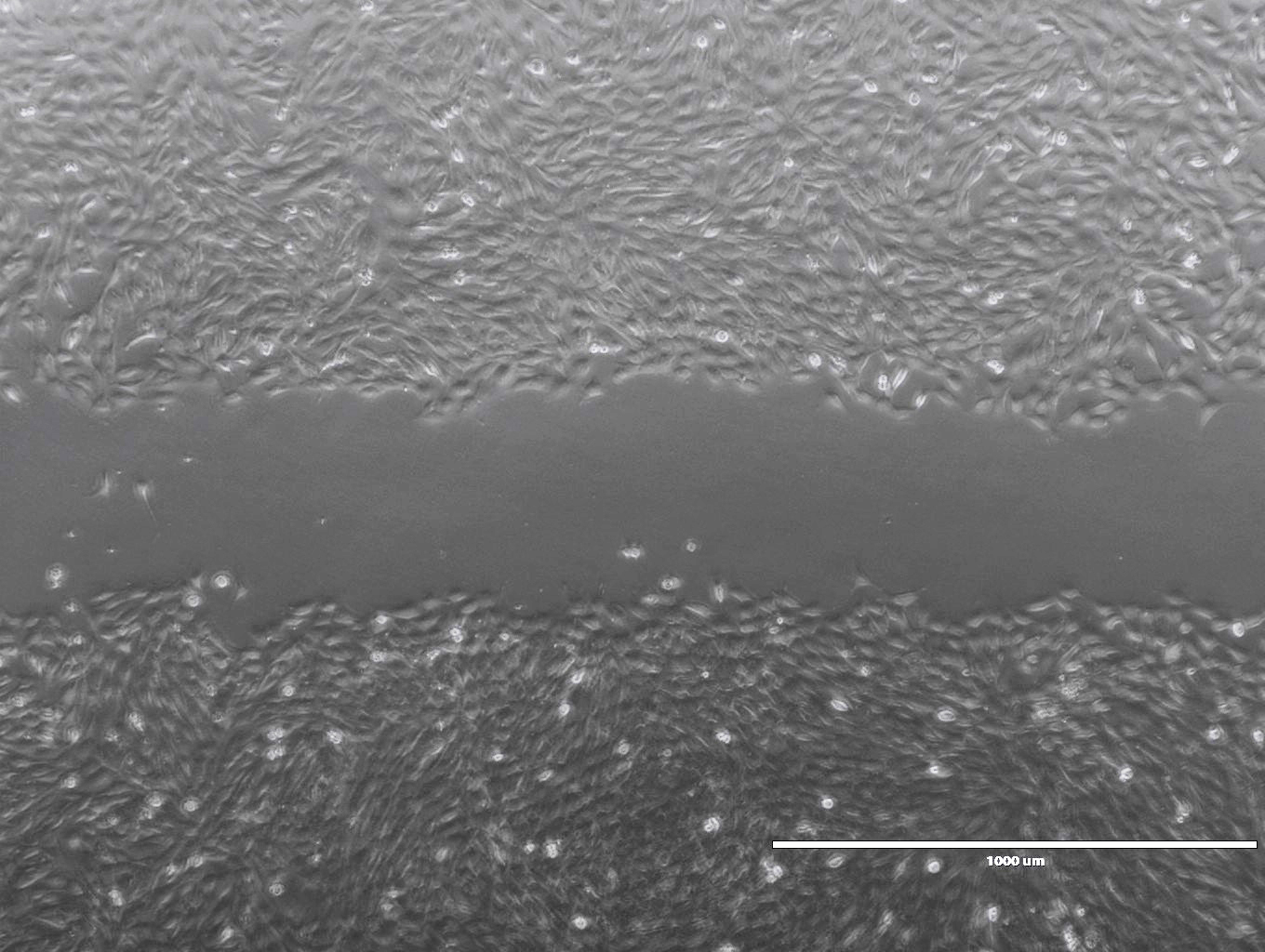

Supplement: Supplementary file 33 — Source data EV and Appendix [file 44318_2025_540_MOESM33_ESM.zip › Source data EV and Appendix/Figure EV 6/6B/Control/4 h.jpg]

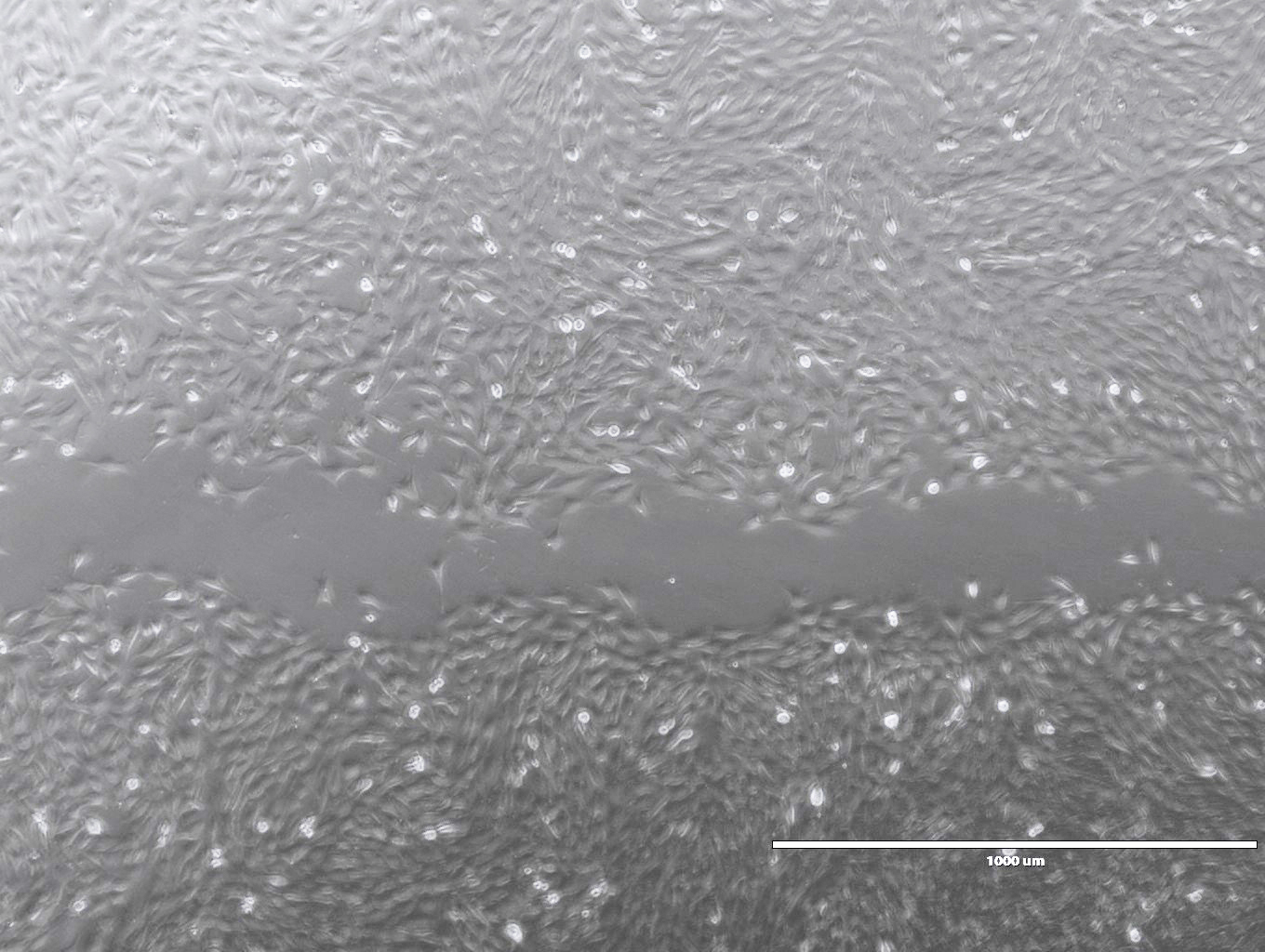

Supplement: Supplementary file 33 — Source data EV and Appendix [file 44318_2025_540_MOESM33_ESM.zip › Source data EV and Appendix/Figure EV 6/6B/Control/8 h.jpg]

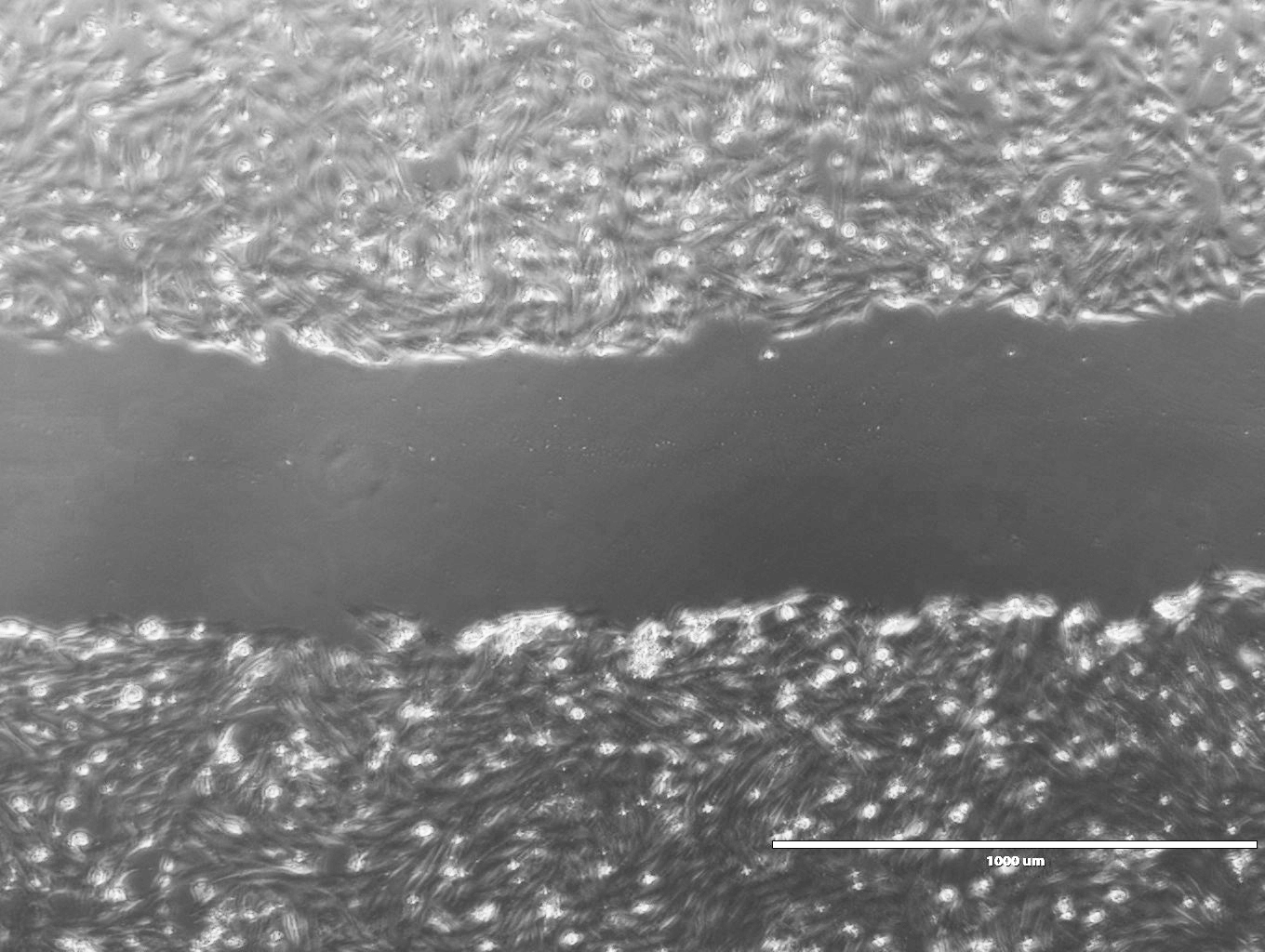

Supplement: Supplementary file 33 — Source data EV and Appendix [file 44318_2025_540_MOESM33_ESM.zip › Source data EV and Appendix/Figure EV 6/6B/LLOMe/0h.jpg]

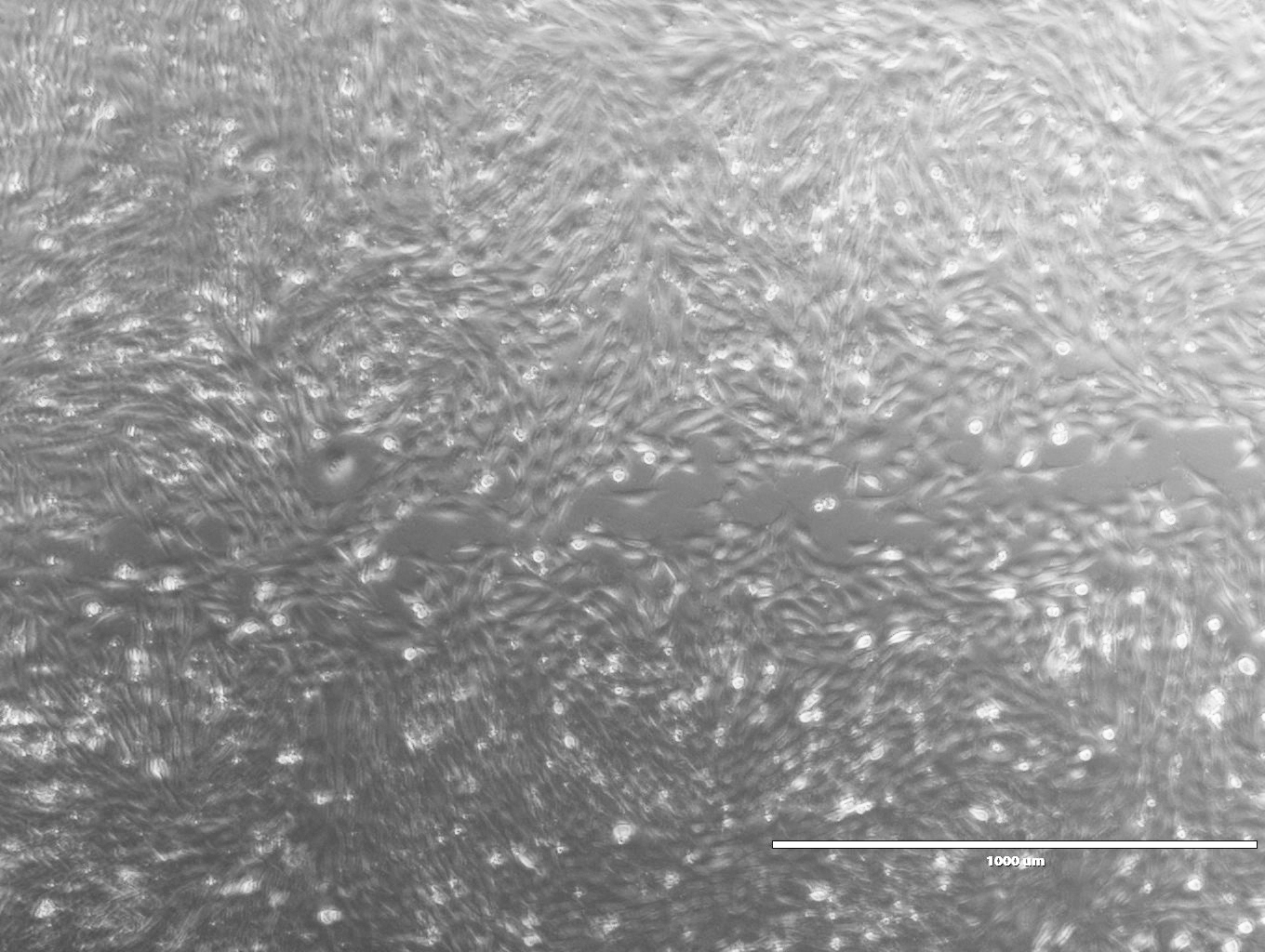

Supplement: Supplementary file 33 — Source data EV and Appendix [file 44318_2025_540_MOESM33_ESM.zip › Source data EV and Appendix/Figure EV 6/6B/LLOMe/12h.jpg]

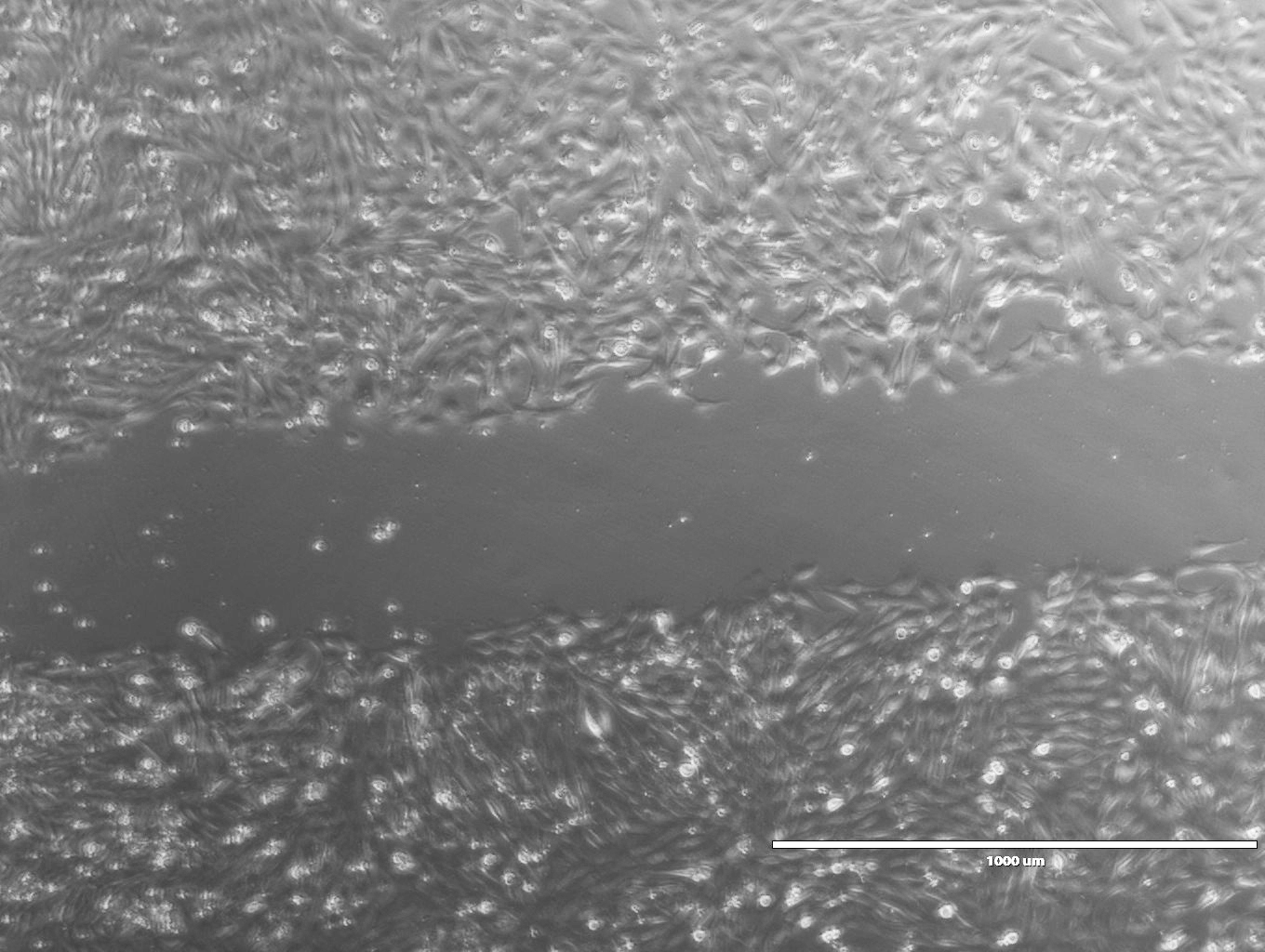

Supplement: Supplementary file 33 — Source data EV and Appendix [file 44318_2025_540_MOESM33_ESM.zip › Source data EV and Appendix/Figure EV 6/6B/LLOMe/4h.jpg]

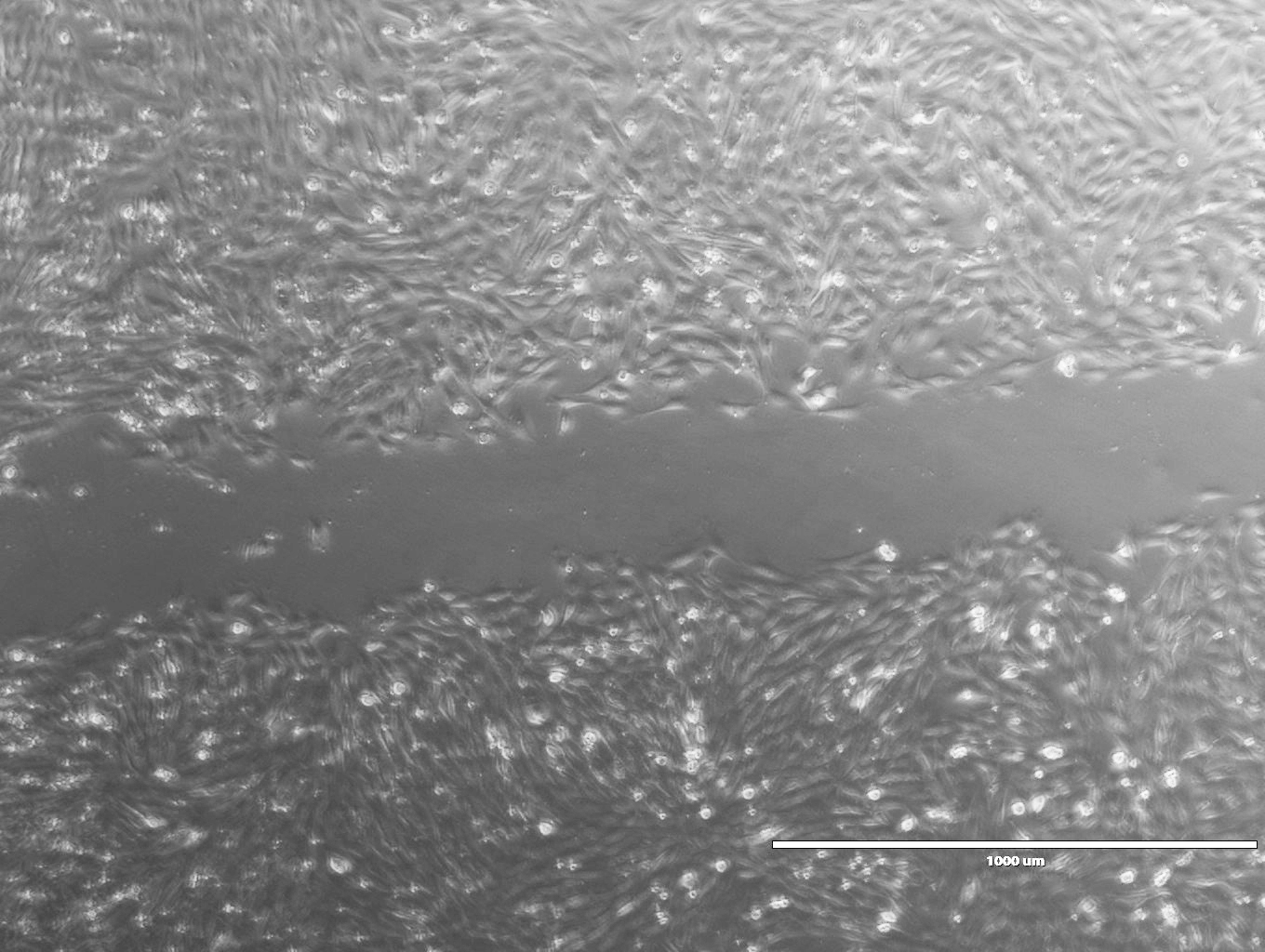

Supplement: Supplementary file 33 — Source data EV and Appendix [file 44318_2025_540_MOESM33_ESM.zip › Source data EV and Appendix/Figure EV 6/6B/LLOMe/8h.jpg]

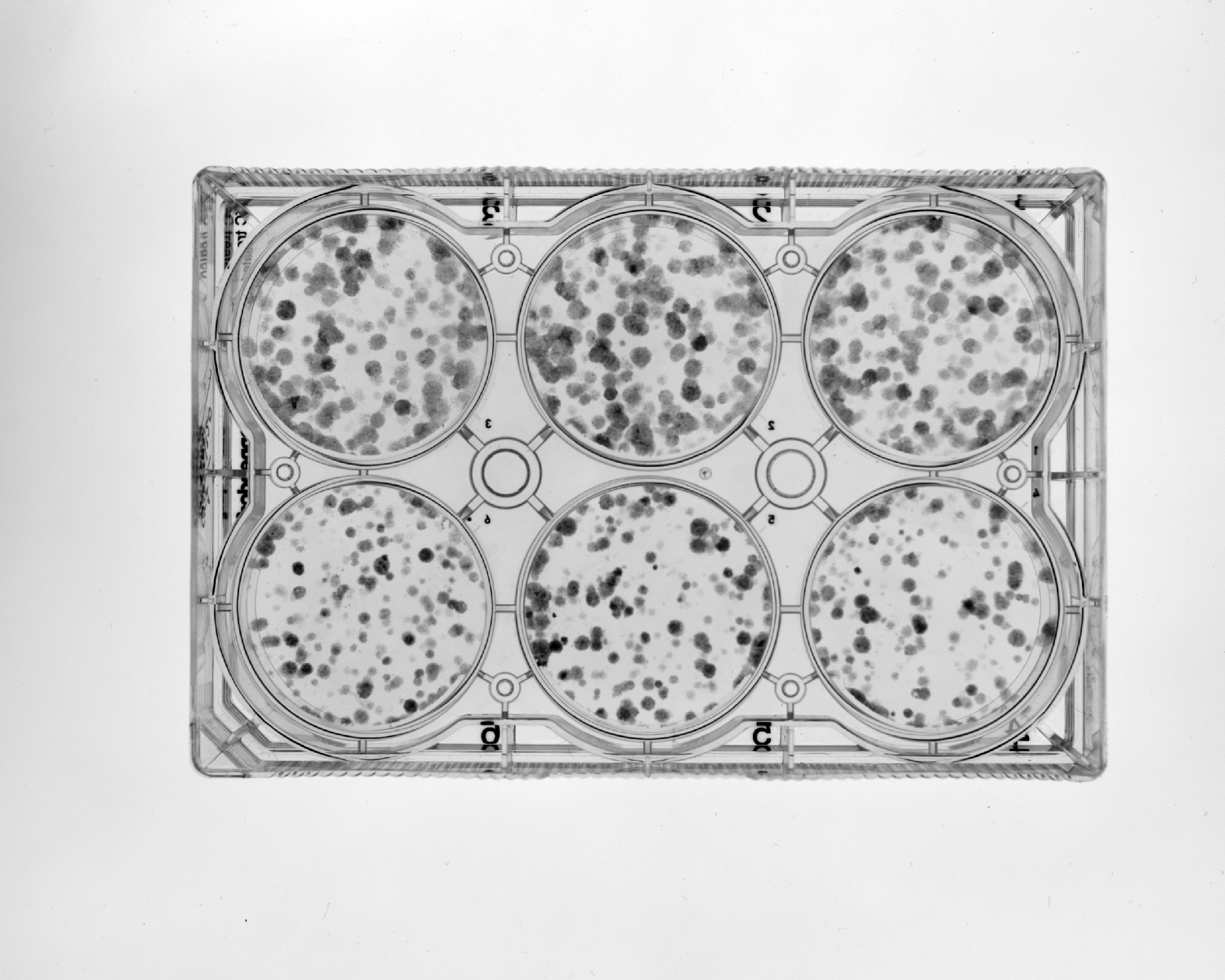

Supplement: Supplementary file 33 — Source data EV and Appendix [file 44318_2025_540_MOESM33_ESM.zip › Source data EV and Appendix/Figure EV 6/6C/Cycle 1.jpg]

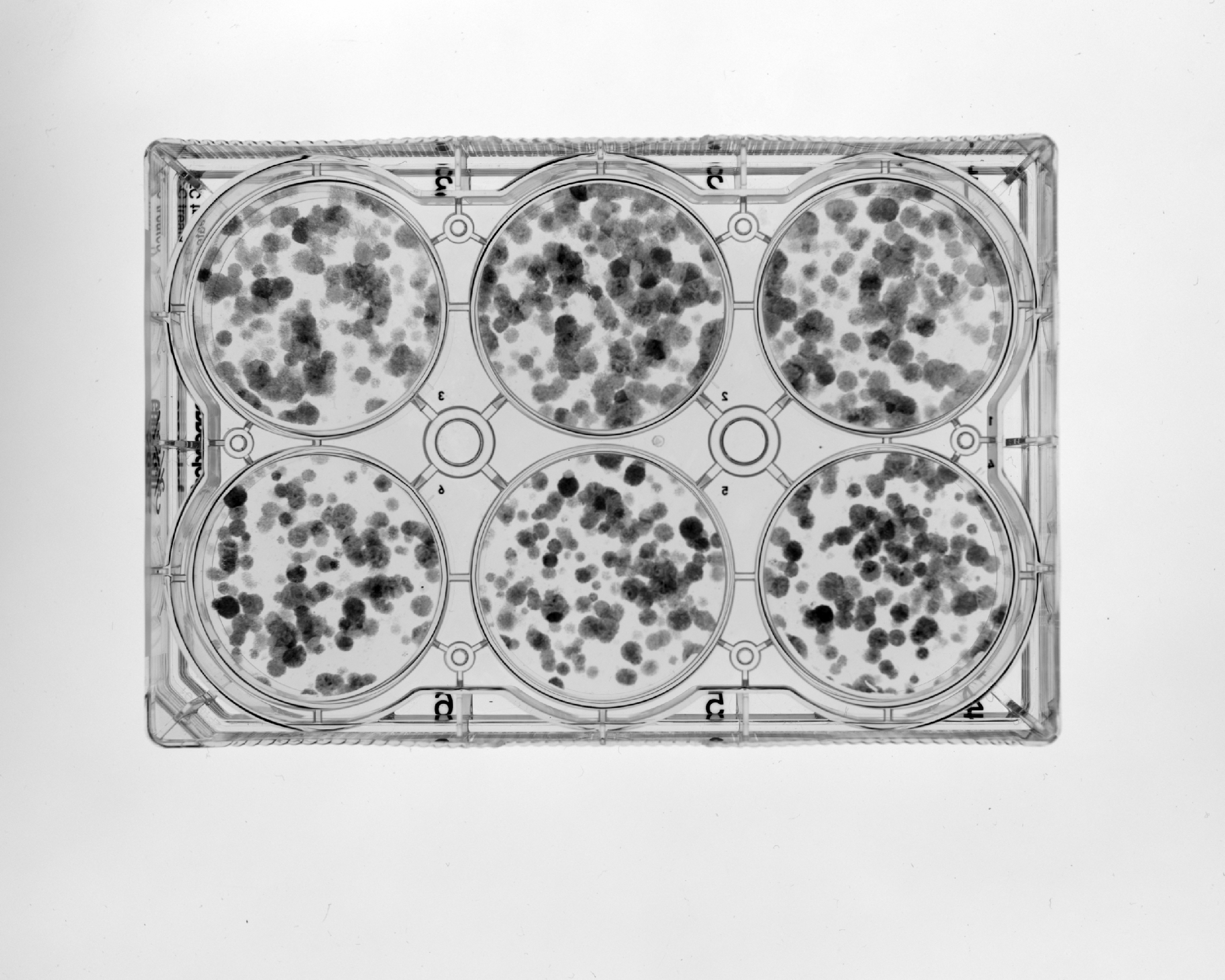

Supplement: Supplementary file 33 — Source data EV and Appendix [file 44318_2025_540_MOESM33_ESM.zip › Source data EV and Appendix/Figure EV 6/6C/Cycle 2.jpg]
