# Supplementary figures and images for: Programmed cell revival from imminent cell death enhances tissue repair and regeneration (part 3 of 4)
Source: EMBO J. 2025 Aug 21;44(19):5244–89. doi: 10.1038/s44318-025-00540-y (PMC12489119; doi:10.1038/s44318-025-00540-y)

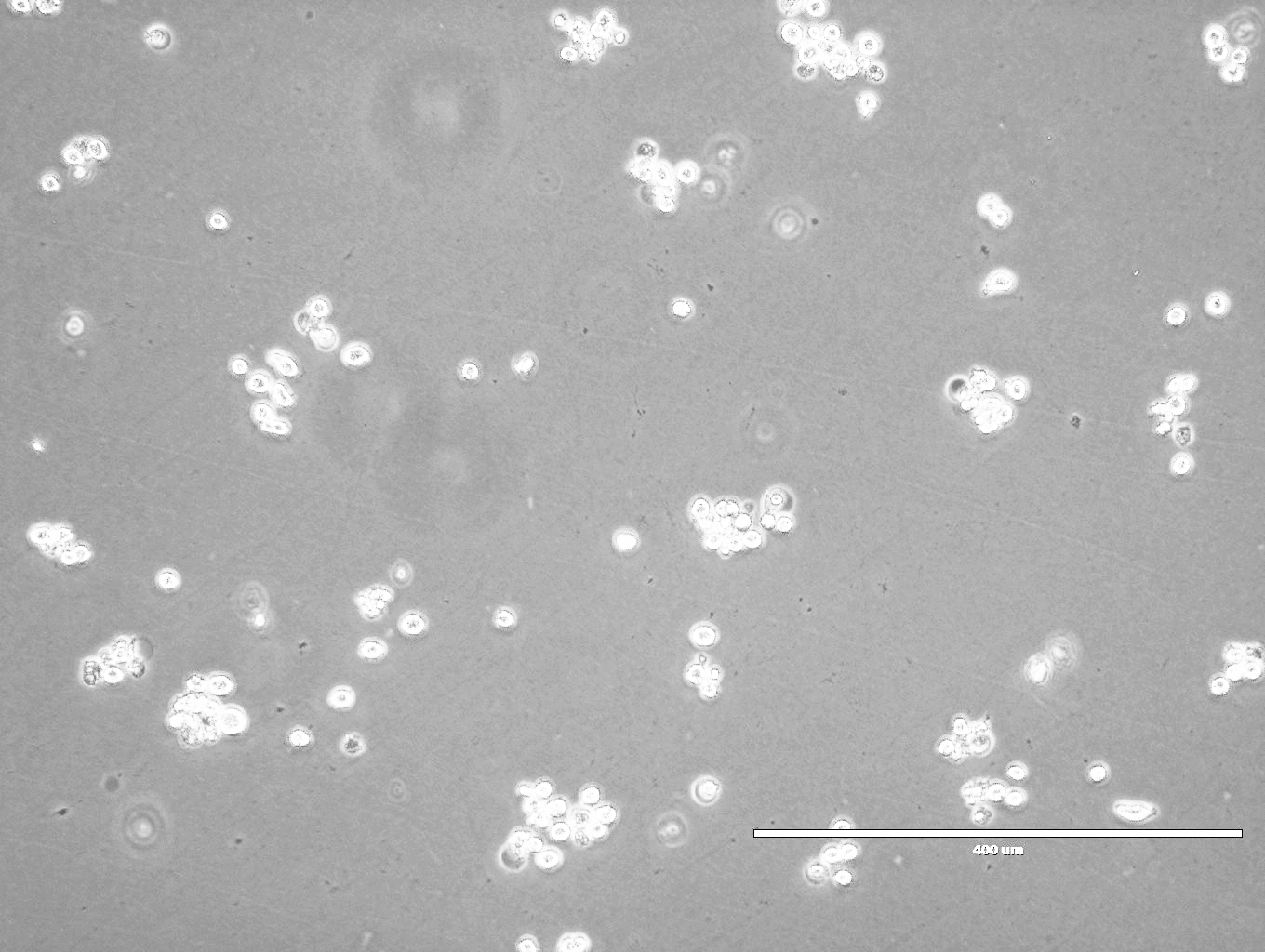

Supplement: Supplementary file 33 — Source data EV and Appendix [file 44318_2025_540_MOESM33_ESM.zip › Source data EV and Appendix/Figure EV 1/1D/Siramesine/MEF_3 h.jpg]

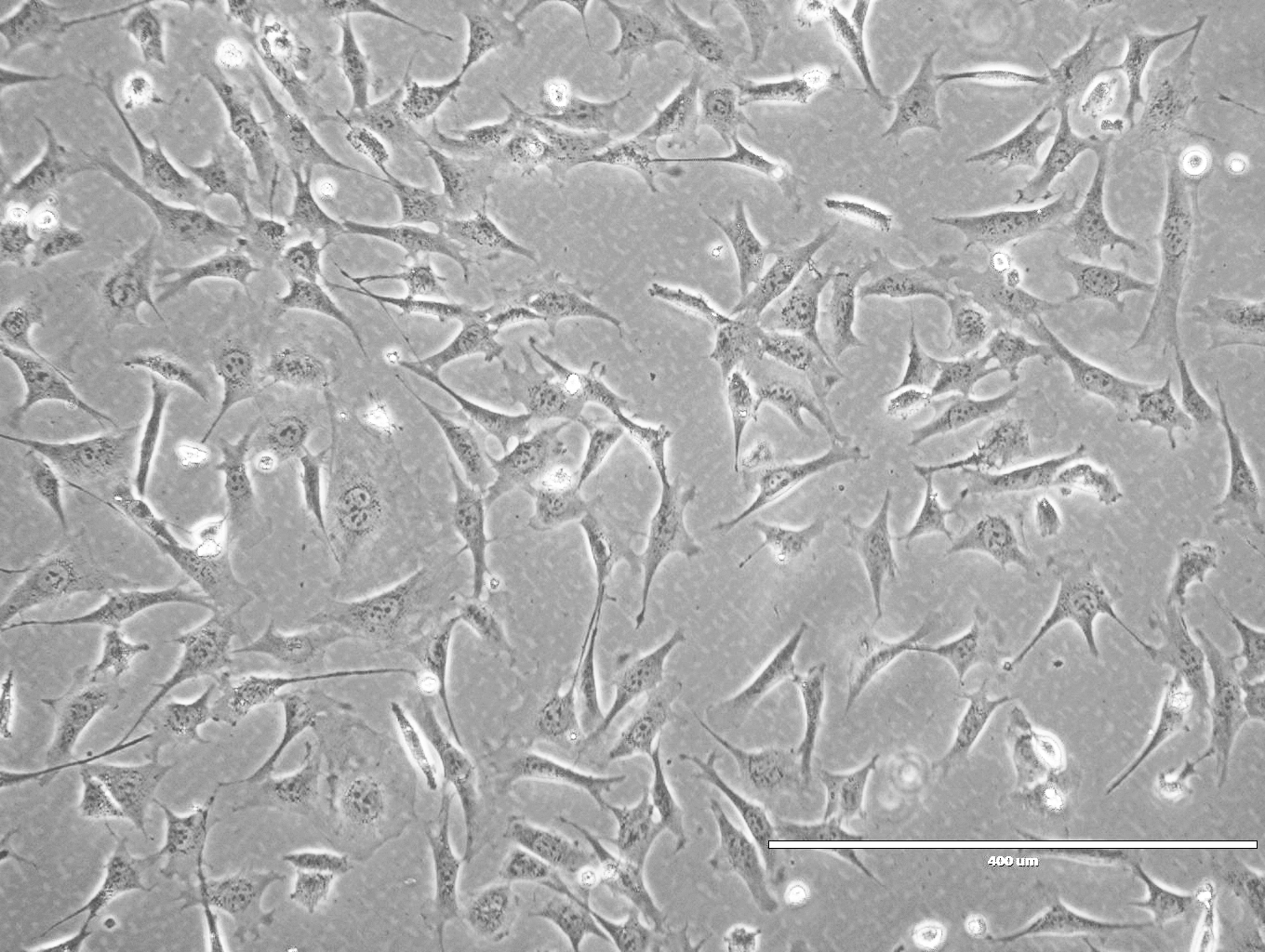

Supplement: Supplementary file 33 — Source data EV and Appendix [file 44318_2025_540_MOESM33_ESM.zip › Source data EV and Appendix/Figure EV 1/1D/Sphingosine/MEF_0 h.jpg]

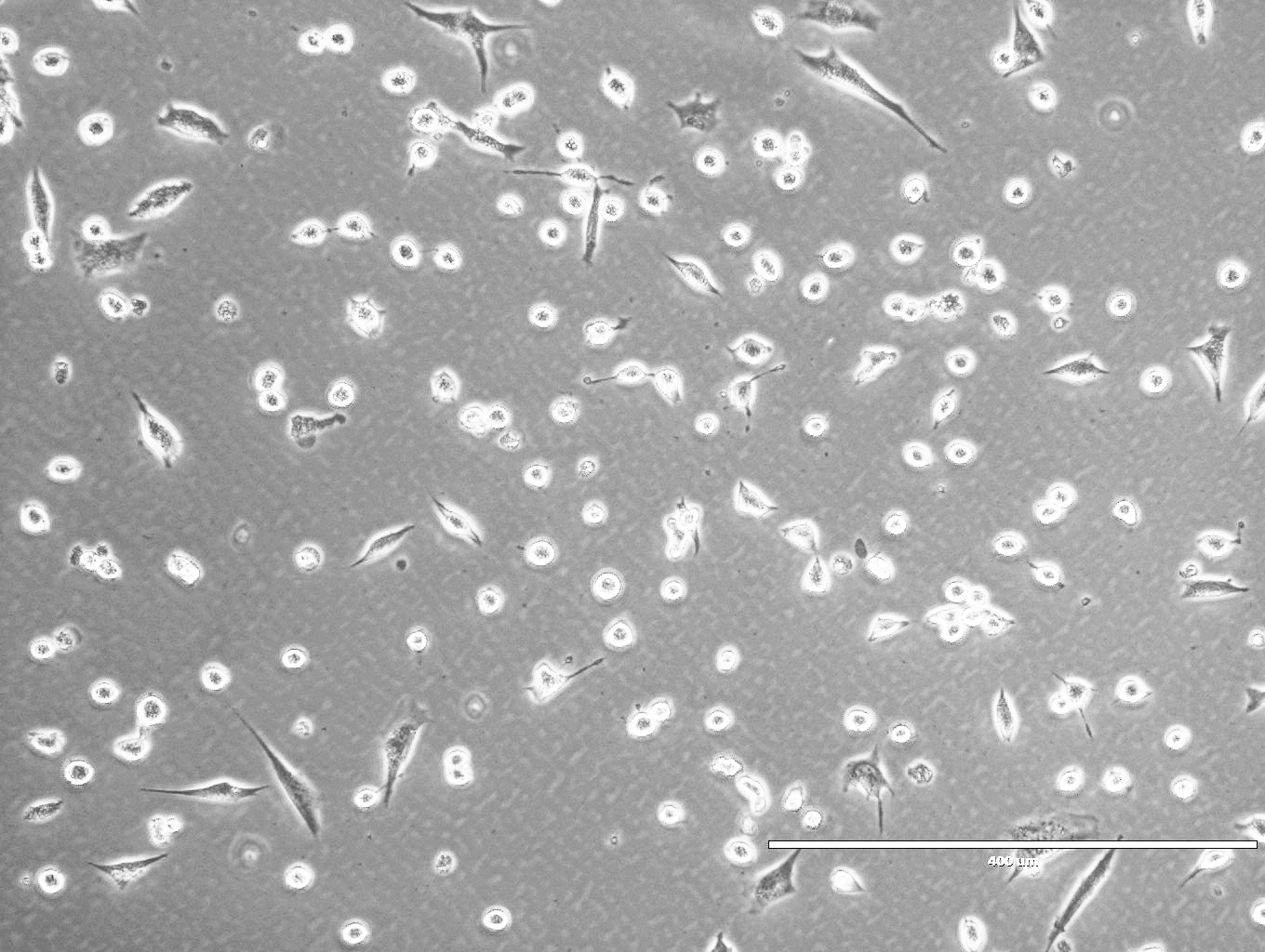

Supplement: Supplementary file 33 — Source data EV and Appendix [file 44318_2025_540_MOESM33_ESM.zip › Source data EV and Appendix/Figure EV 1/1D/Sphingosine/MEF_1 h 30 mins.jpg]

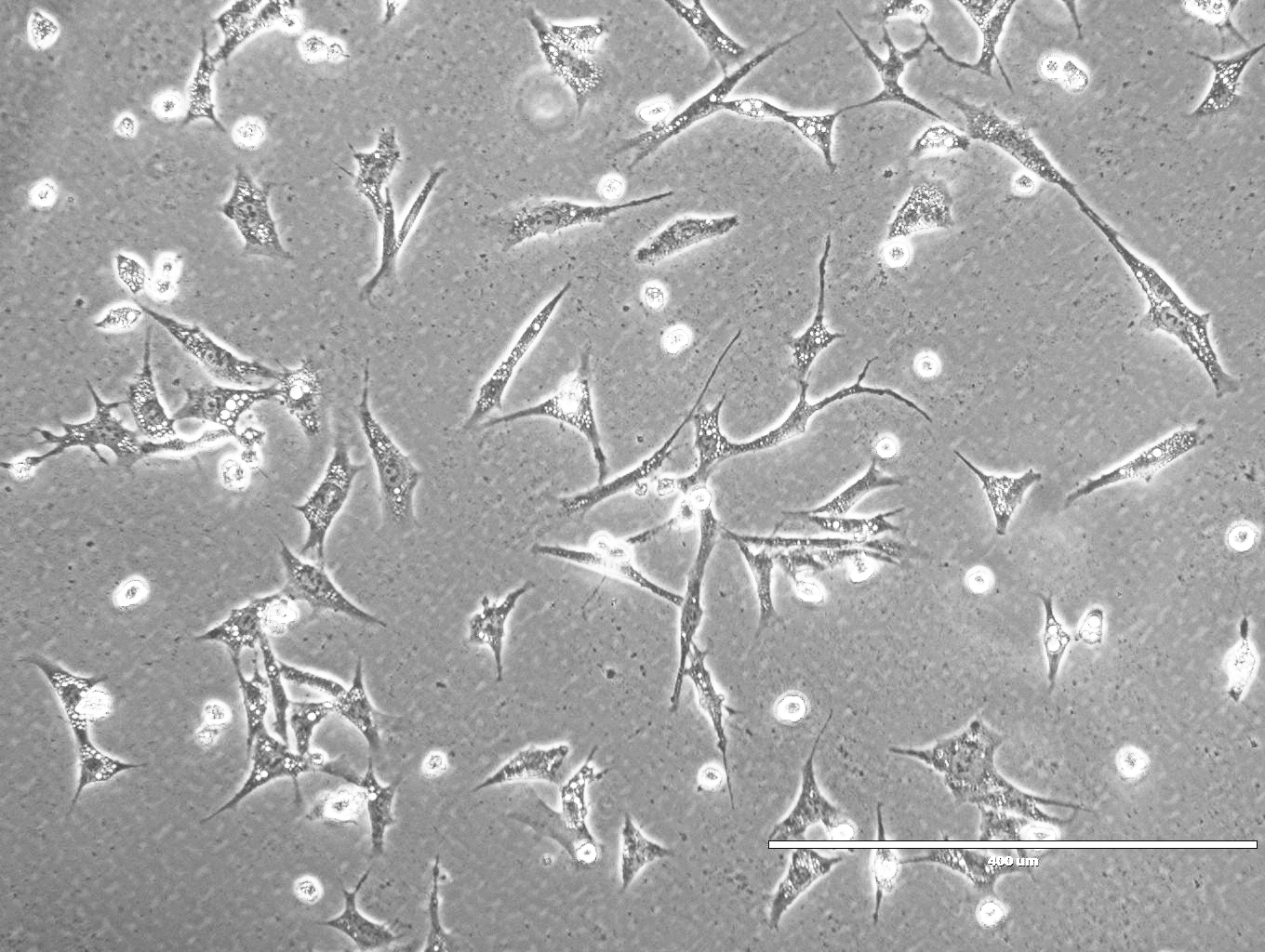

Supplement: Supplementary file 33 — Source data EV and Appendix [file 44318_2025_540_MOESM33_ESM.zip › Source data EV and Appendix/Figure EV 1/1D/Sphingosine/MEF_12 h.jpg]

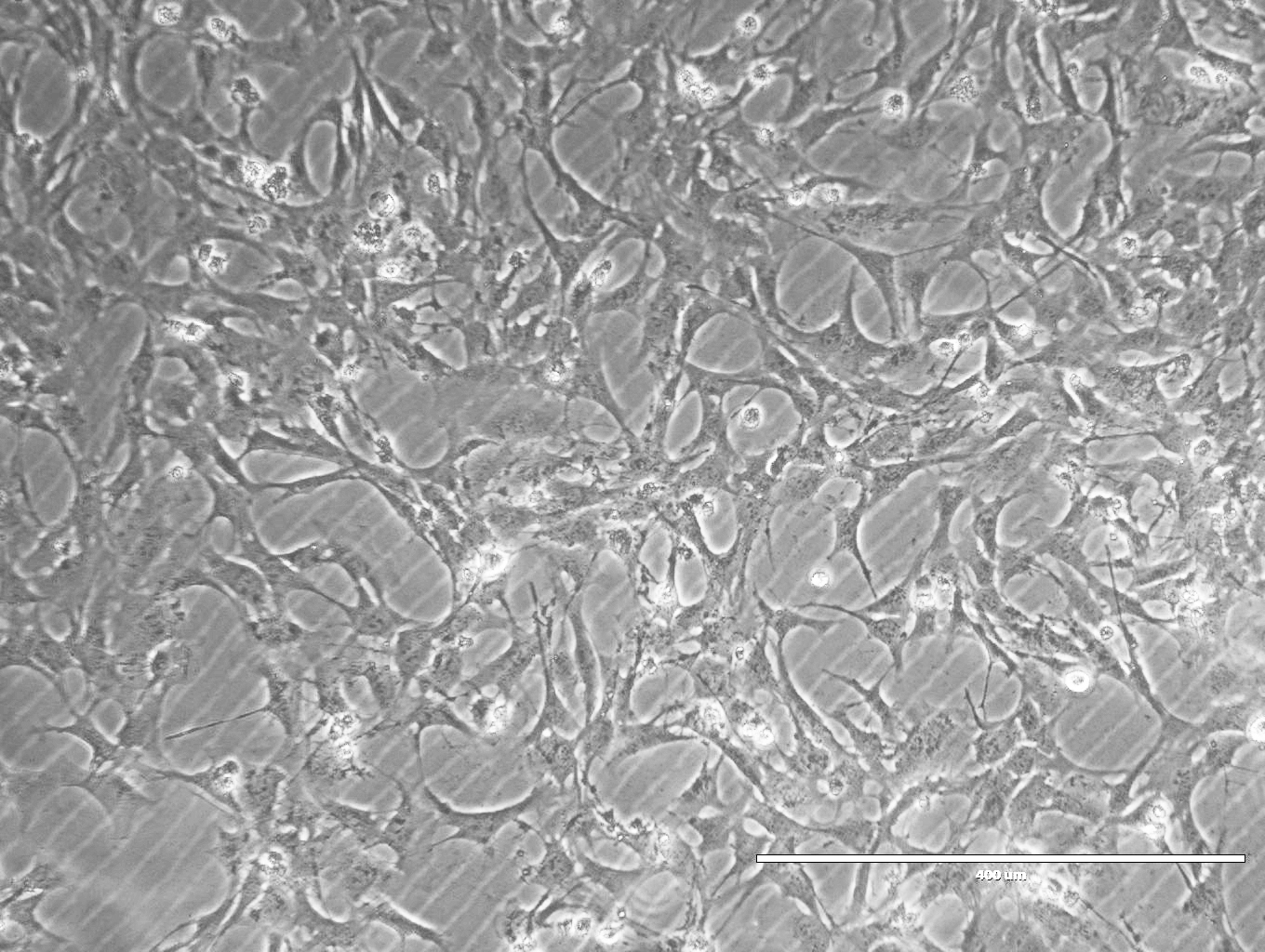

Supplement: Supplementary file 33 — Source data EV and Appendix [file 44318_2025_540_MOESM33_ESM.zip › Source data EV and Appendix/Figure EV 1/1D/Sphingosine/MEF_24 h.jpg]

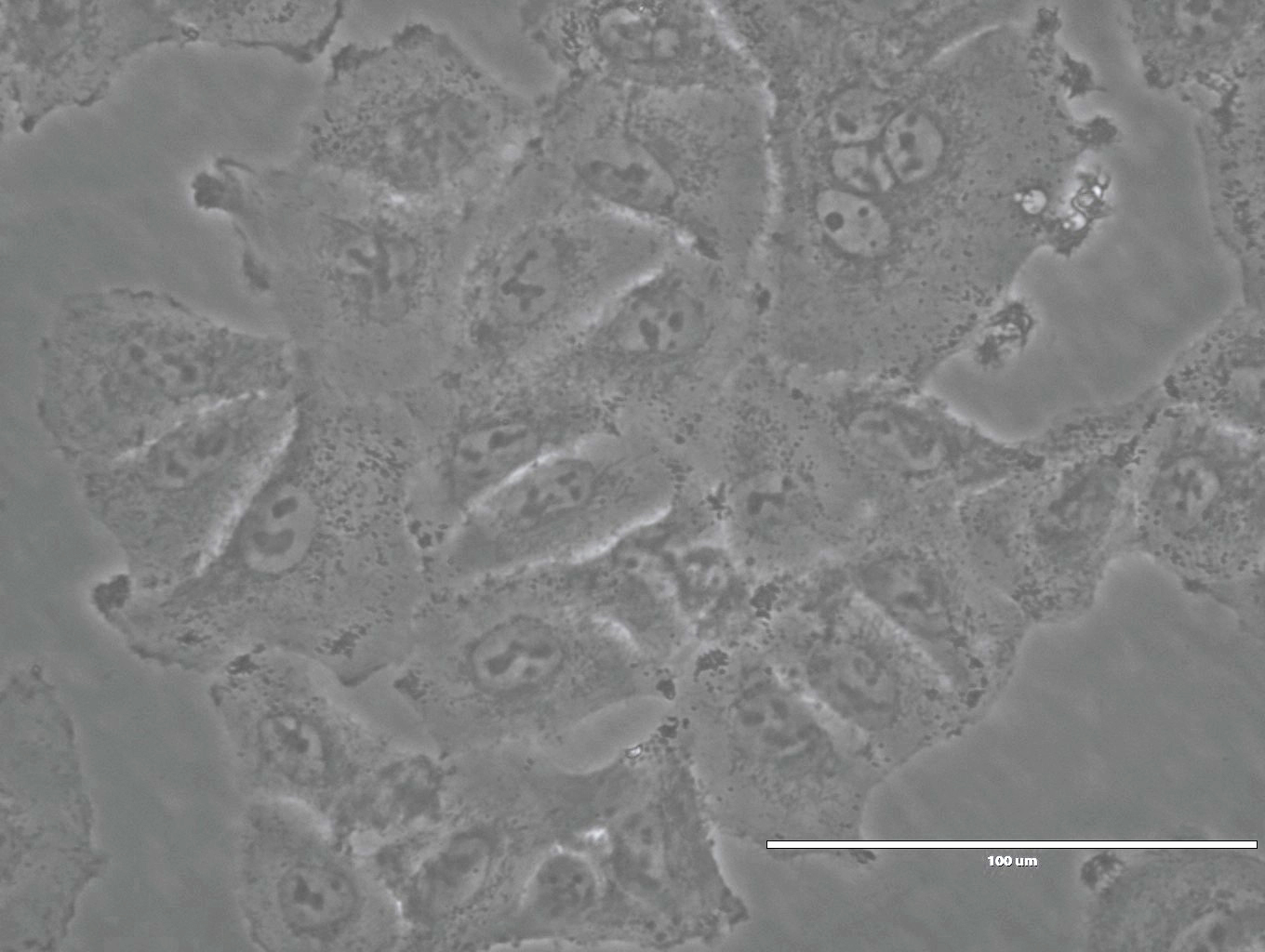

Supplement: Supplementary file 33 — Source data EV and Appendix [file 44318_2025_540_MOESM33_ESM.zip › Source data EV and Appendix/Figure EV 1/1E/A549/A549_0 h.jpg]

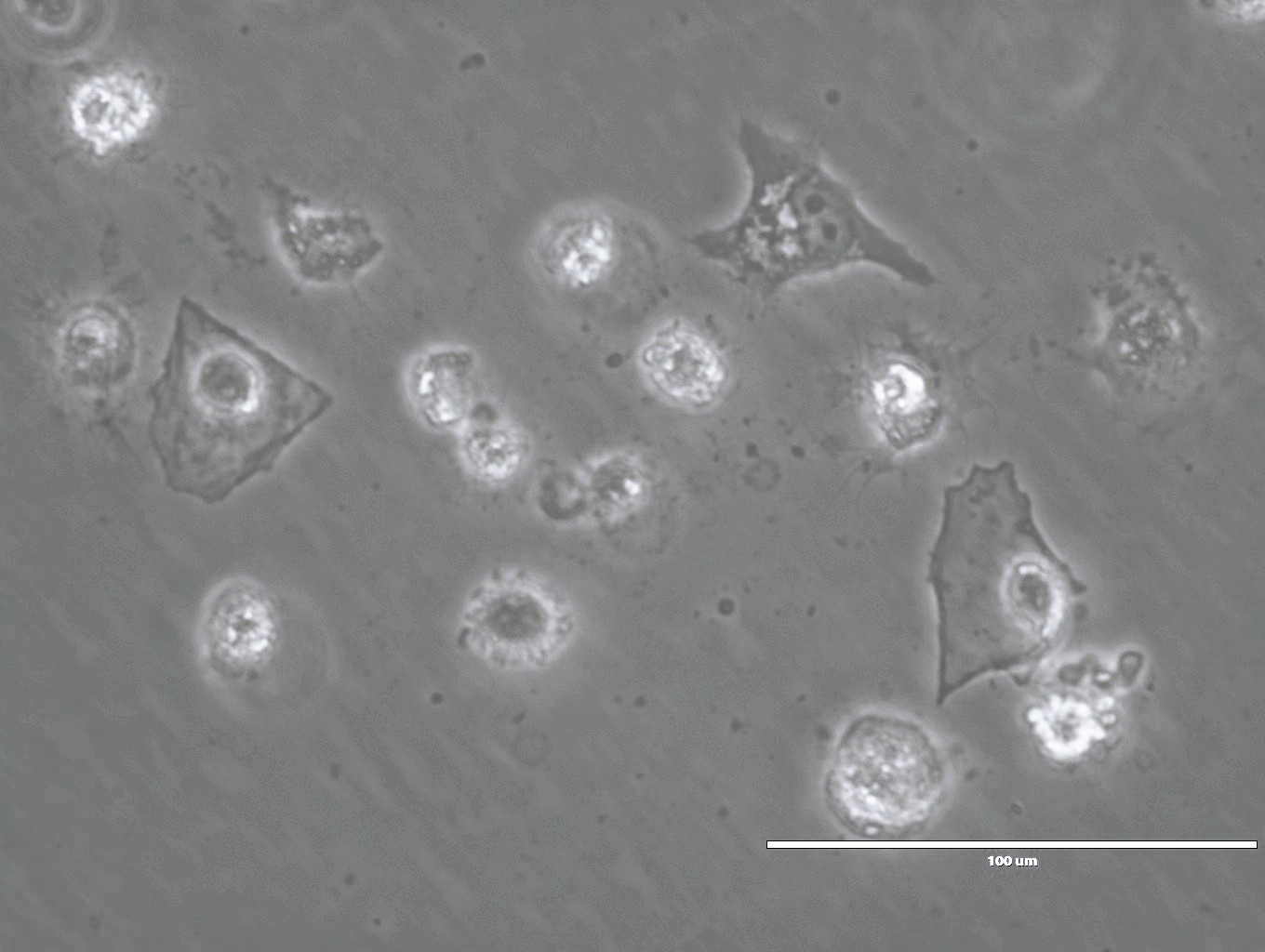

Supplement: Supplementary file 33 — Source data EV and Appendix [file 44318_2025_540_MOESM33_ESM.zip › Source data EV and Appendix/Figure EV 1/1E/A549/A549_24 h.jpg]

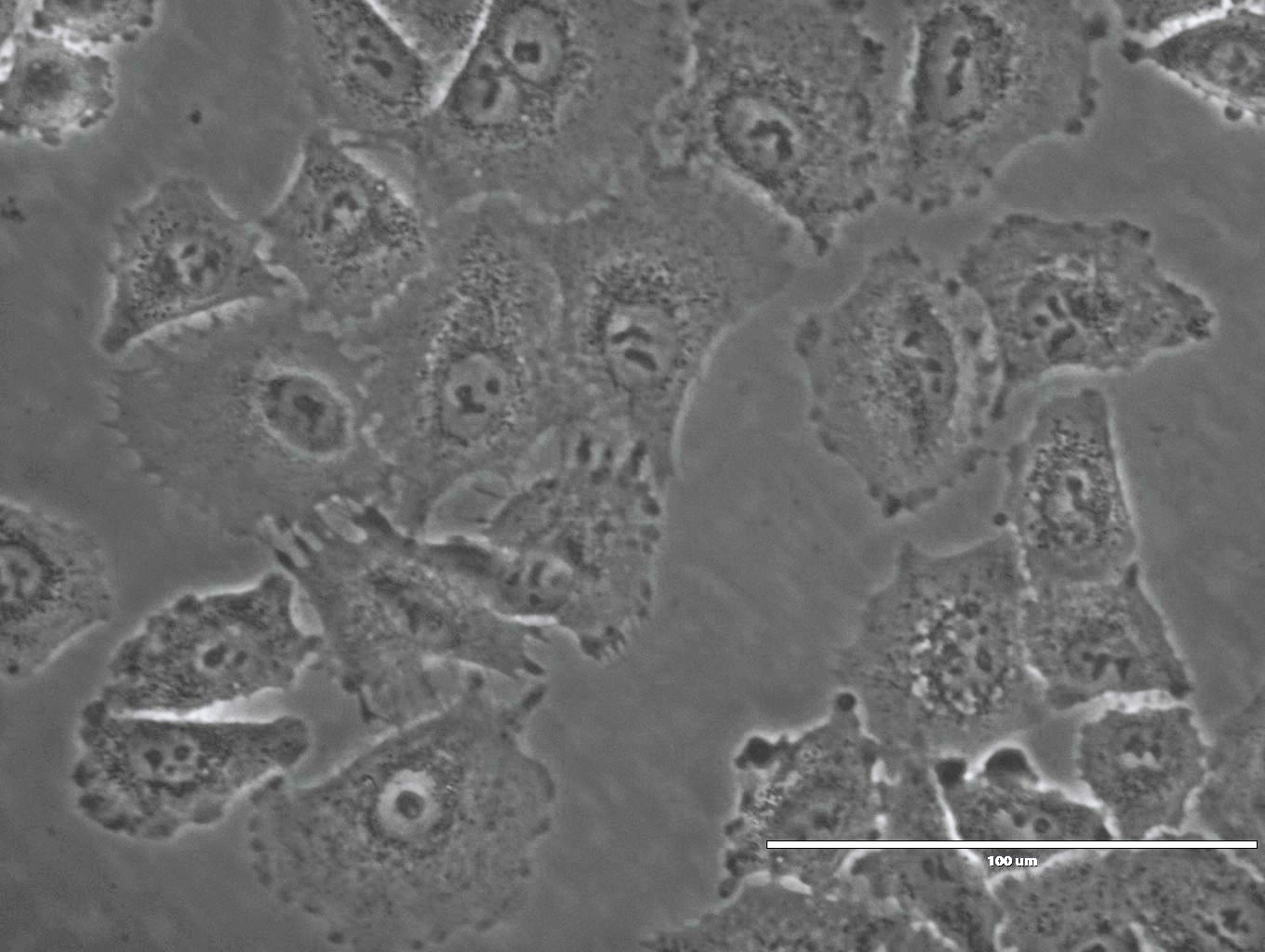

Supplement: Supplementary file 33 — Source data EV and Appendix [file 44318_2025_540_MOESM33_ESM.zip › Source data EV and Appendix/Figure EV 1/1E/A549/A549_30 min.jpg]

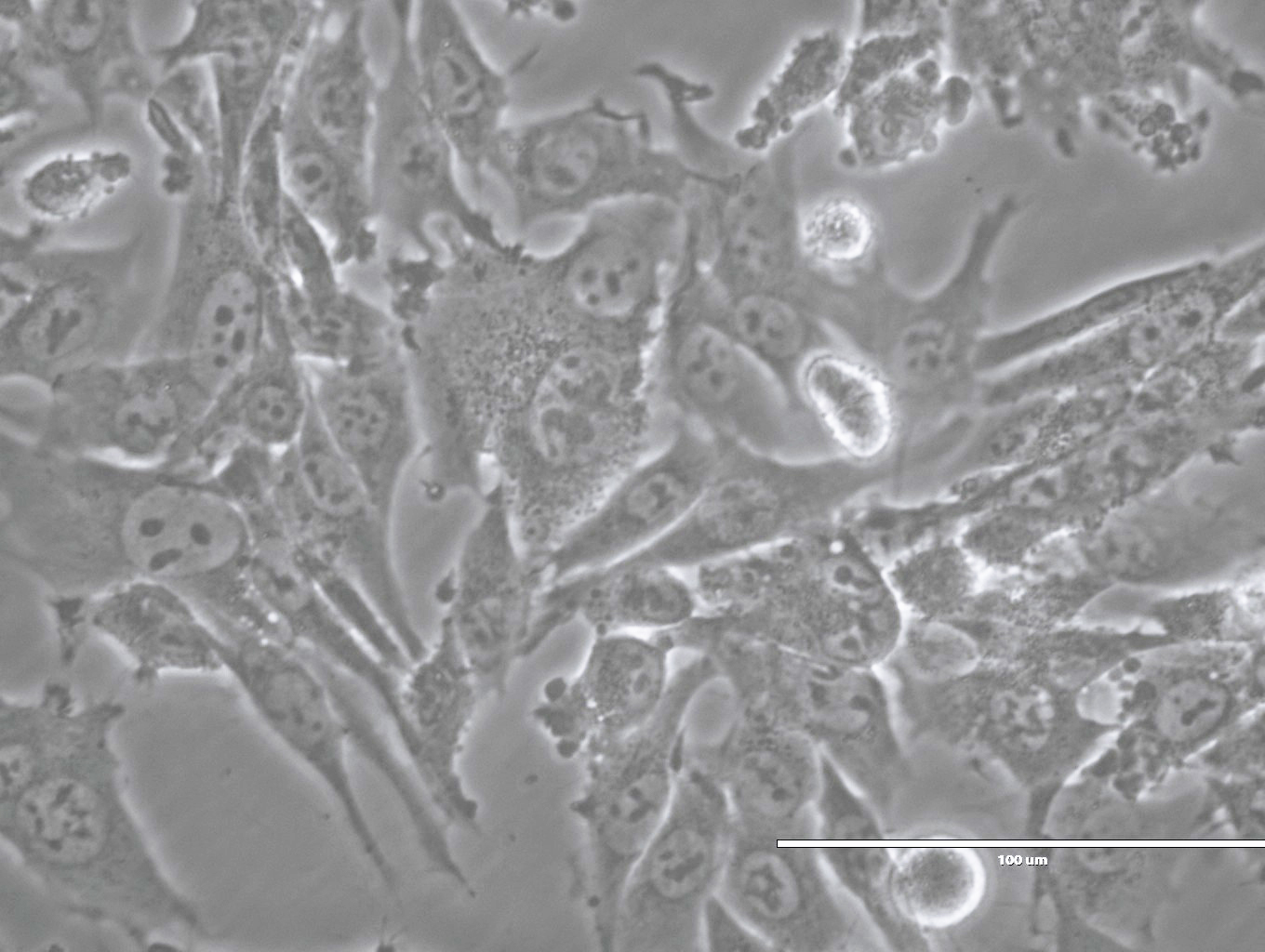

Supplement: Supplementary file 33 — Source data EV and Appendix [file 44318_2025_540_MOESM33_ESM.zip › Source data EV and Appendix/Figure EV 1/1E/BHK21/BHK21_0 h.jpg]

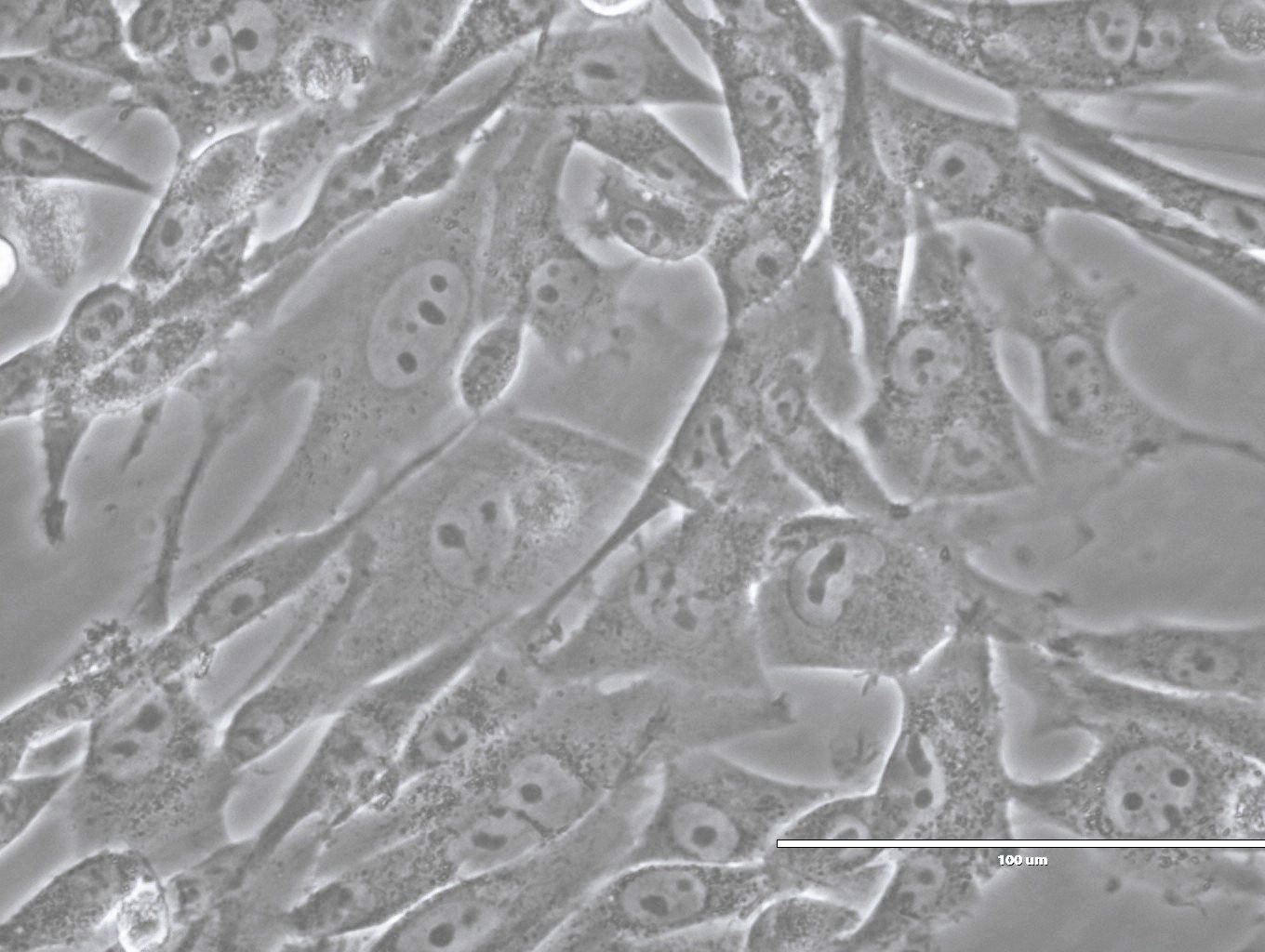

Supplement: Supplementary file 33 — Source data EV and Appendix [file 44318_2025_540_MOESM33_ESM.zip › Source data EV and Appendix/Figure EV 1/1E/BHK21/BHK21_24 h.jpg]

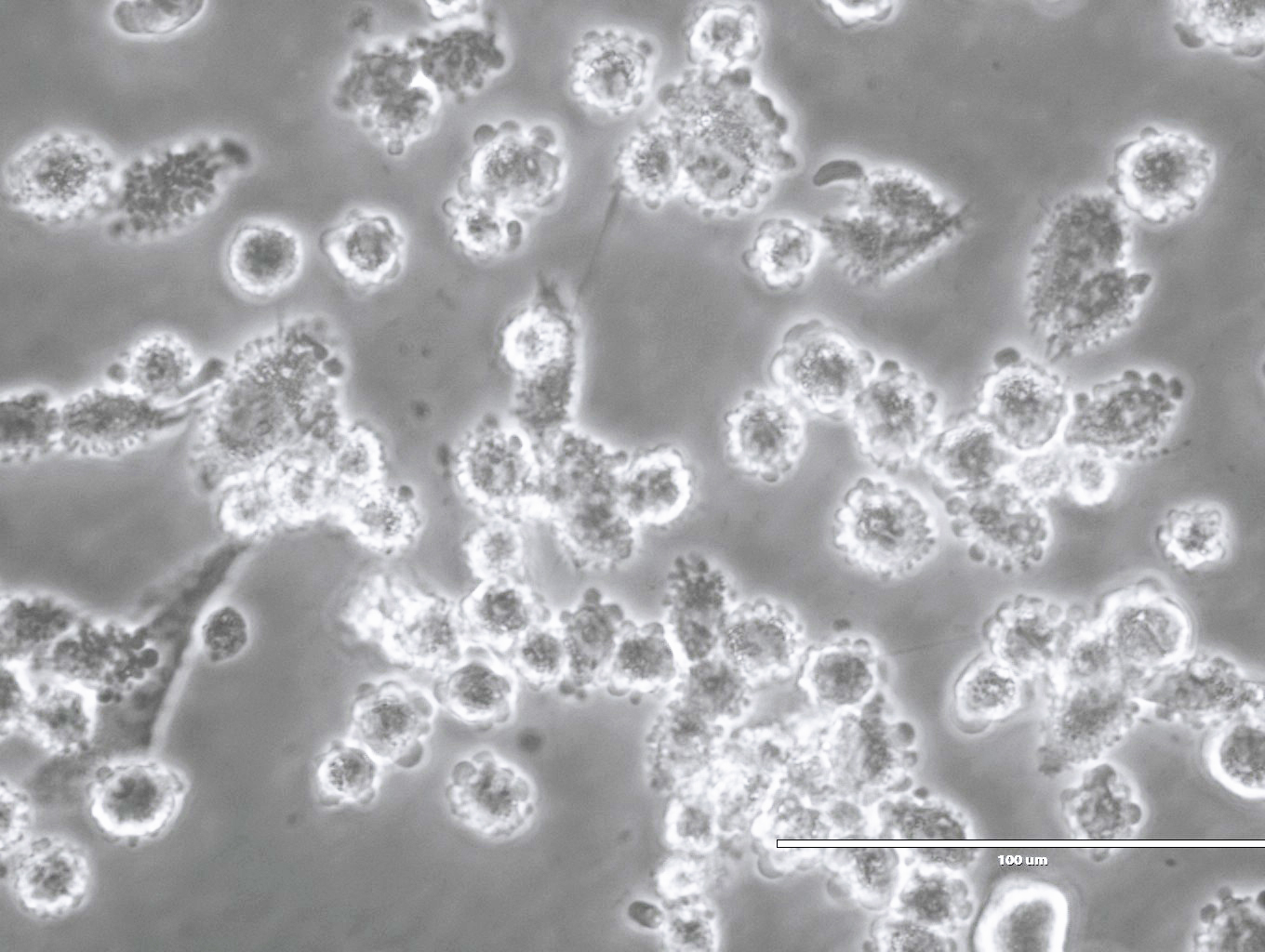

Supplement: Supplementary file 33 — Source data EV and Appendix [file 44318_2025_540_MOESM33_ESM.zip › Source data EV and Appendix/Figure EV 1/1E/BHK21/BHK21_30 min.jpg]

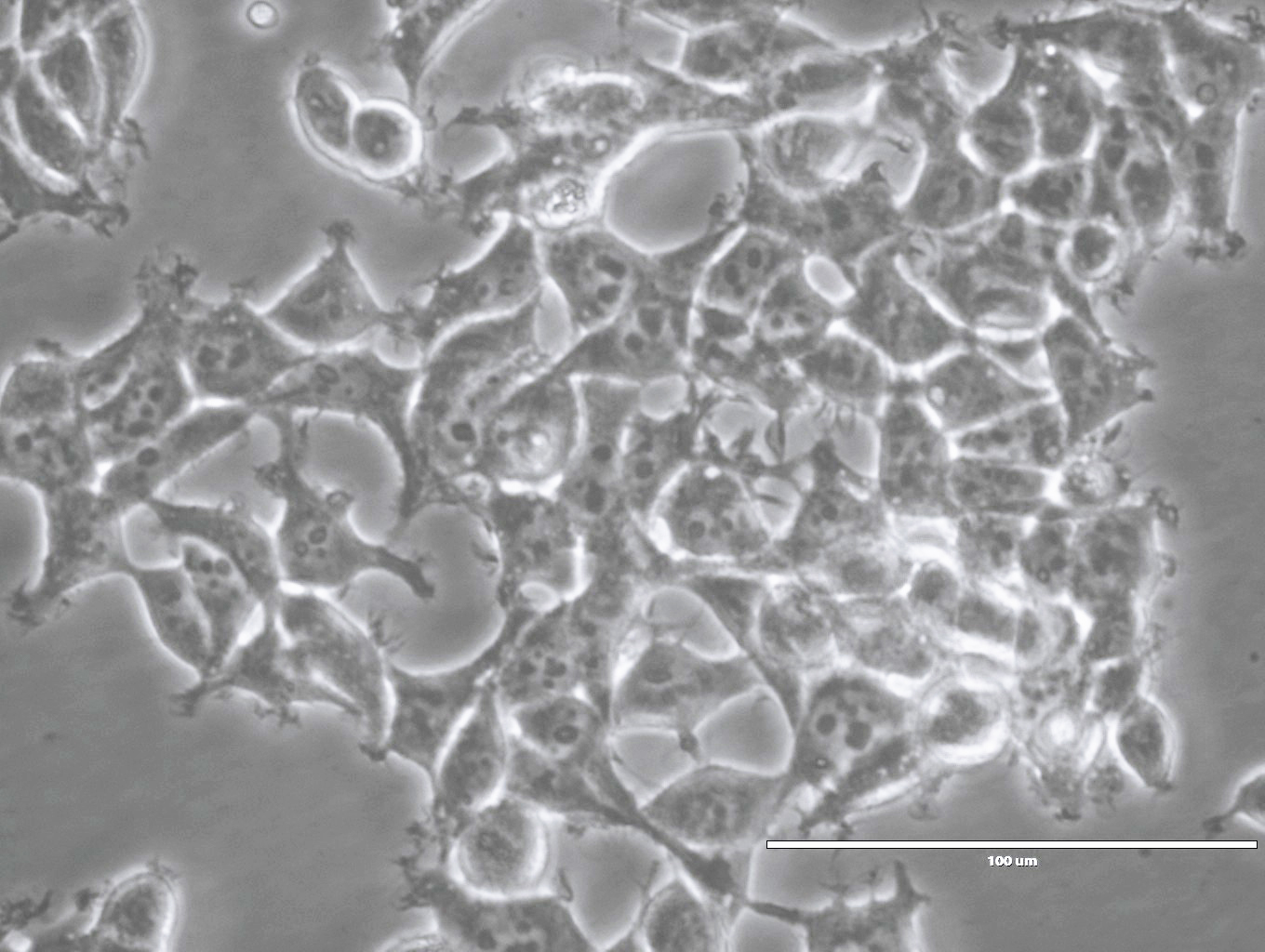

Supplement: Supplementary file 33 — Source data EV and Appendix [file 44318_2025_540_MOESM33_ESM.zip › Source data EV and Appendix/Figure EV 1/1E/HEK 293T/HEK293T_0 h.jpg]

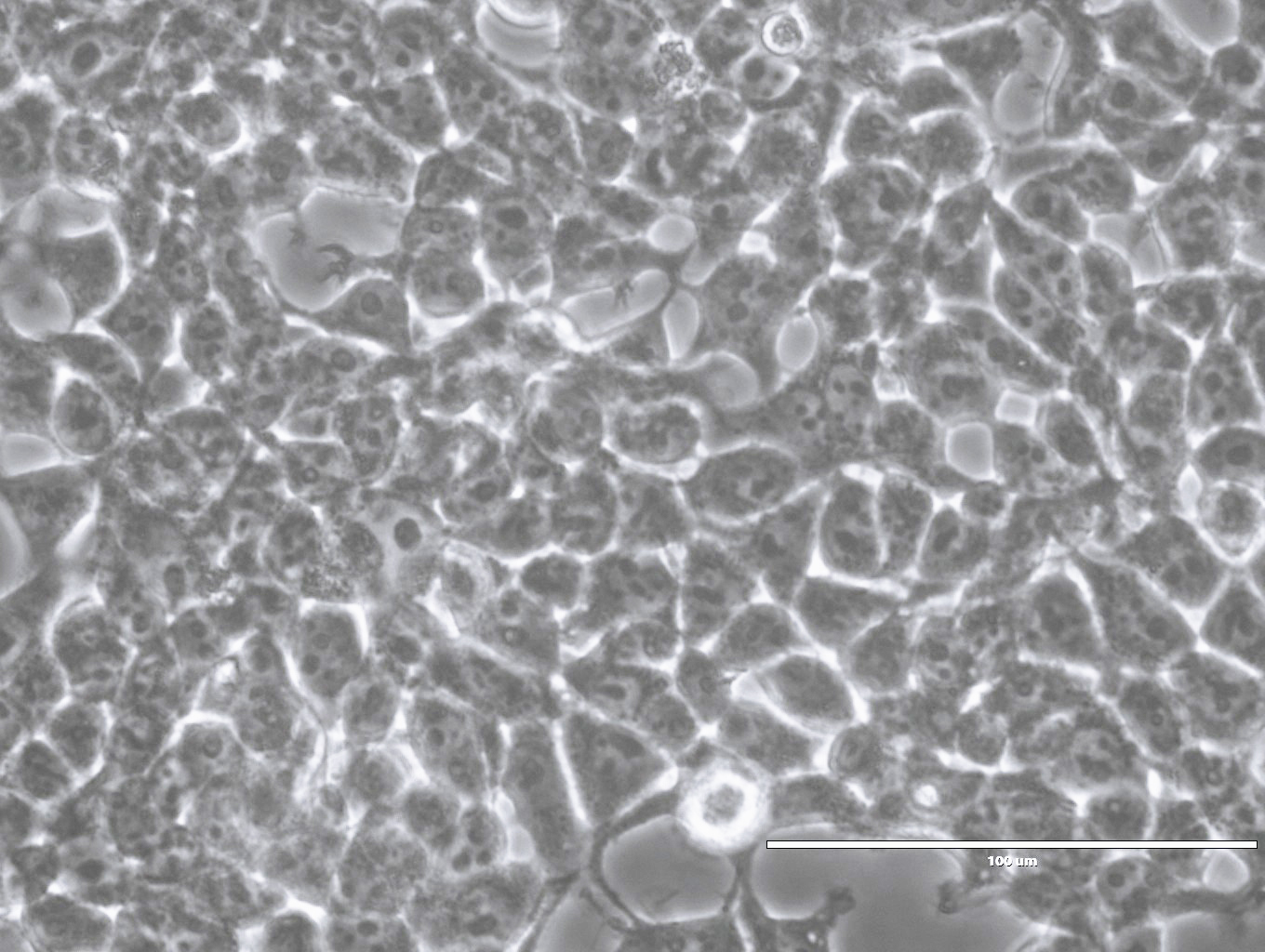

Supplement: Supplementary file 33 — Source data EV and Appendix [file 44318_2025_540_MOESM33_ESM.zip › Source data EV and Appendix/Figure EV 1/1E/HEK 293T/HEK293T_24 h.jpg]

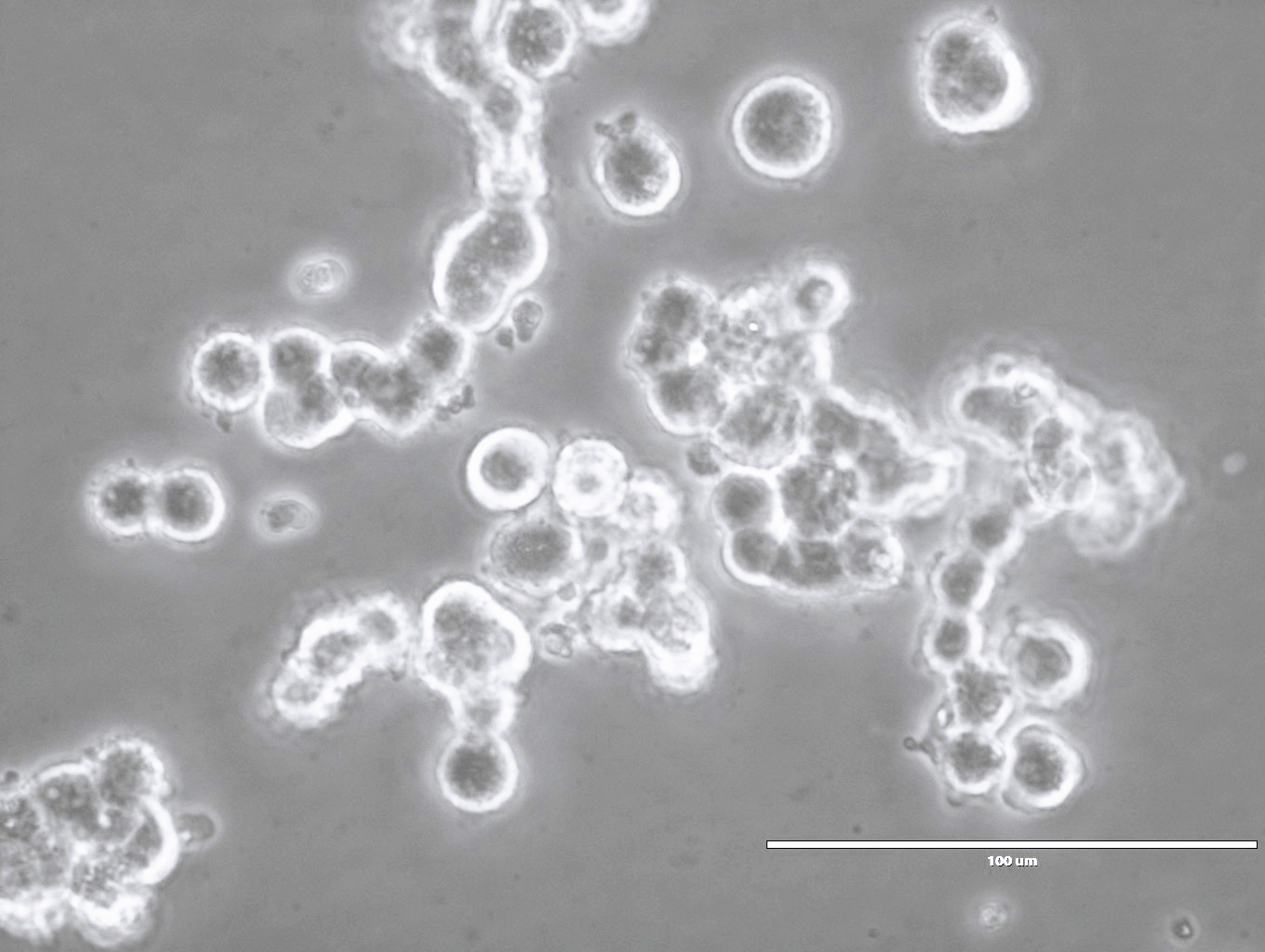

Supplement: Supplementary file 33 — Source data EV and Appendix [file 44318_2025_540_MOESM33_ESM.zip › Source data EV and Appendix/Figure EV 1/1E/HEK 293T/HEK293T_30 min.jpg]

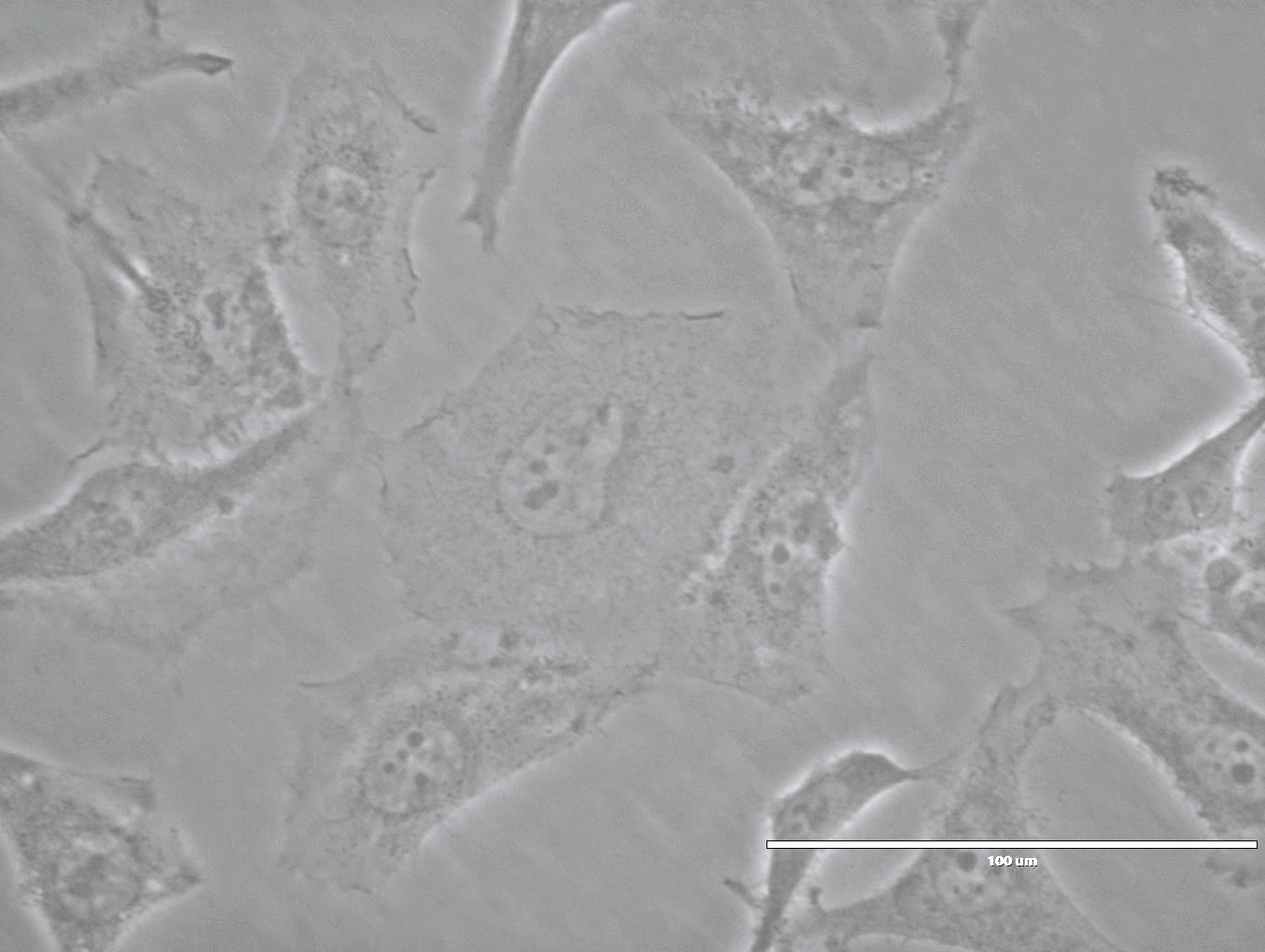

Supplement: Supplementary file 33 — Source data EV and Appendix [file 44318_2025_540_MOESM33_ESM.zip › Source data EV and Appendix/Figure EV 1/1E/HeLa/HeLa_0 h.jpg]

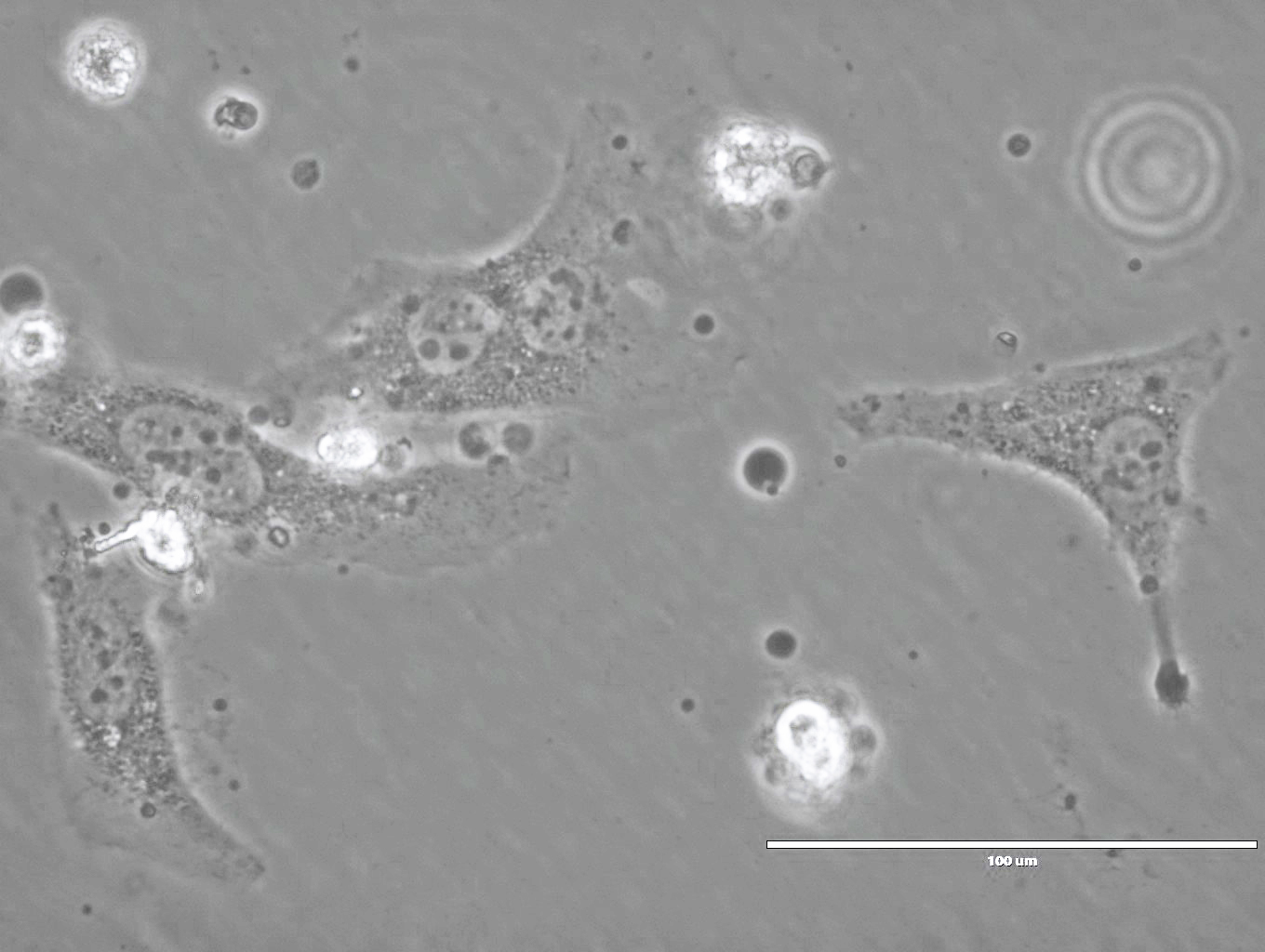

Supplement: Supplementary file 33 — Source data EV and Appendix [file 44318_2025_540_MOESM33_ESM.zip › Source data EV and Appendix/Figure EV 1/1E/HeLa/HeLa_24 h.jpg]

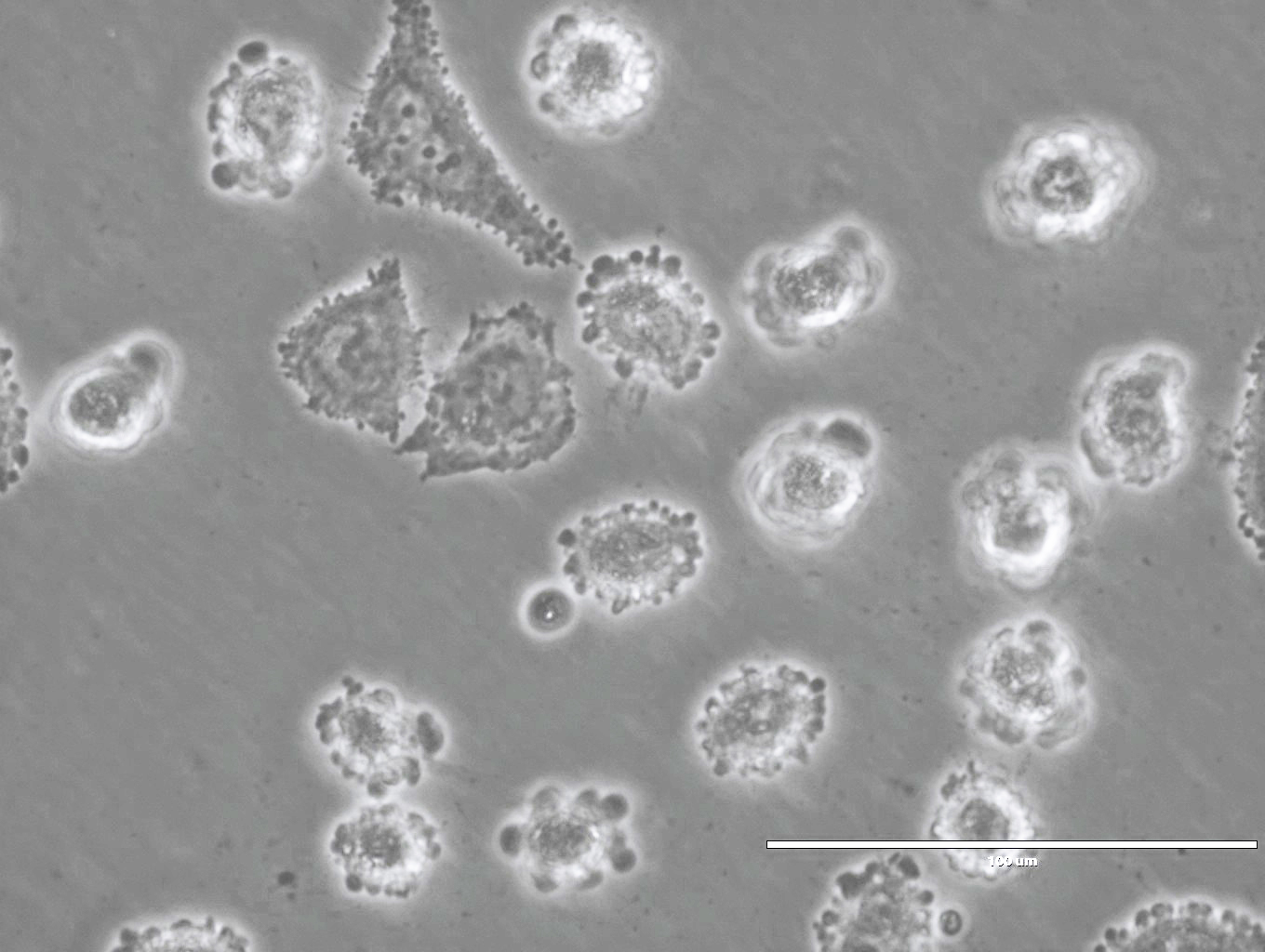

Supplement: Supplementary file 33 — Source data EV and Appendix [file 44318_2025_540_MOESM33_ESM.zip › Source data EV and Appendix/Figure EV 1/1E/HeLa/HeLa_30 min.jpg]

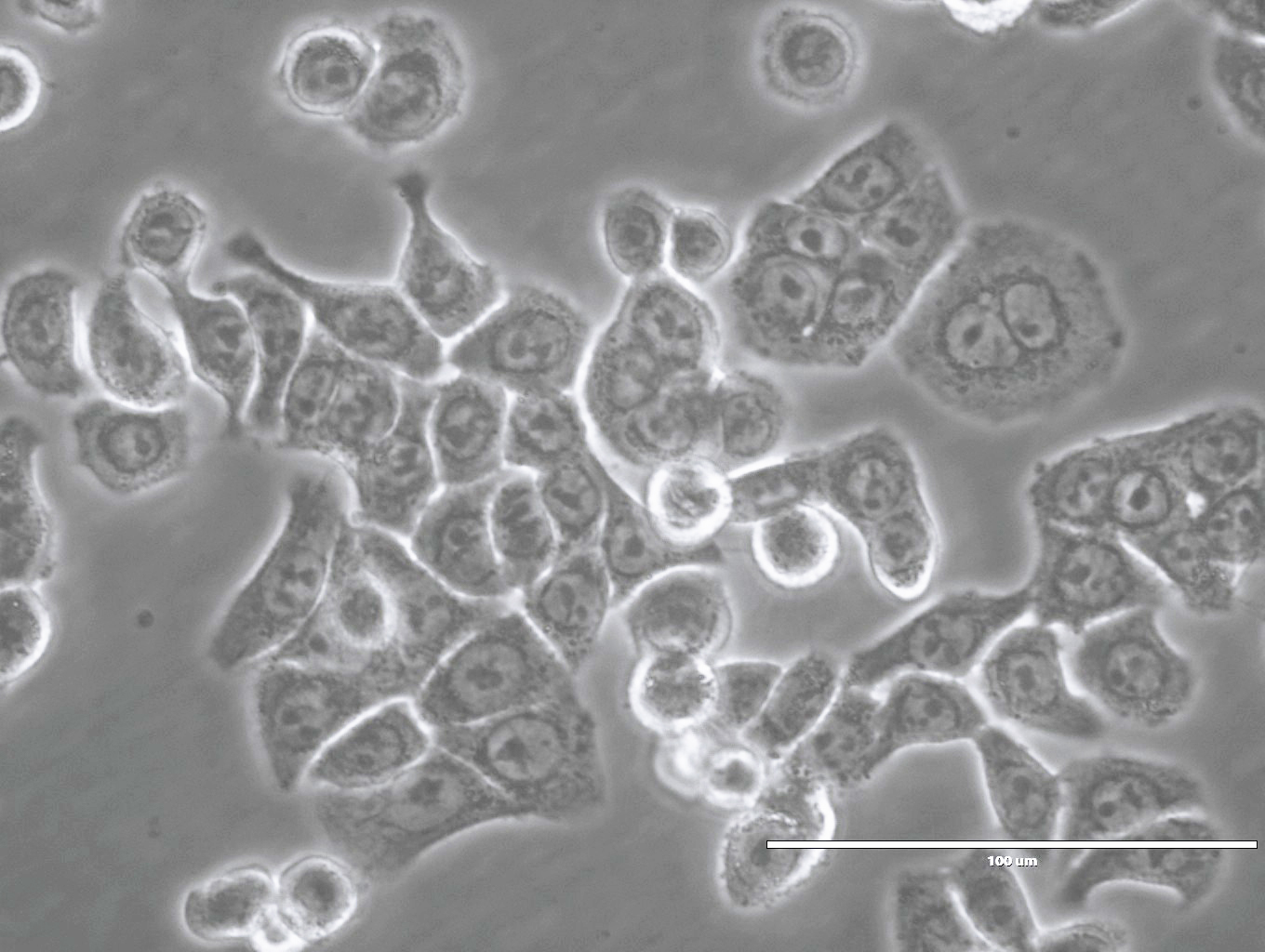

Supplement: Supplementary file 33 — Source data EV and Appendix [file 44318_2025_540_MOESM33_ESM.zip › Source data EV and Appendix/Figure EV 1/1E/HT-29/HT-29_0 h.jpg]

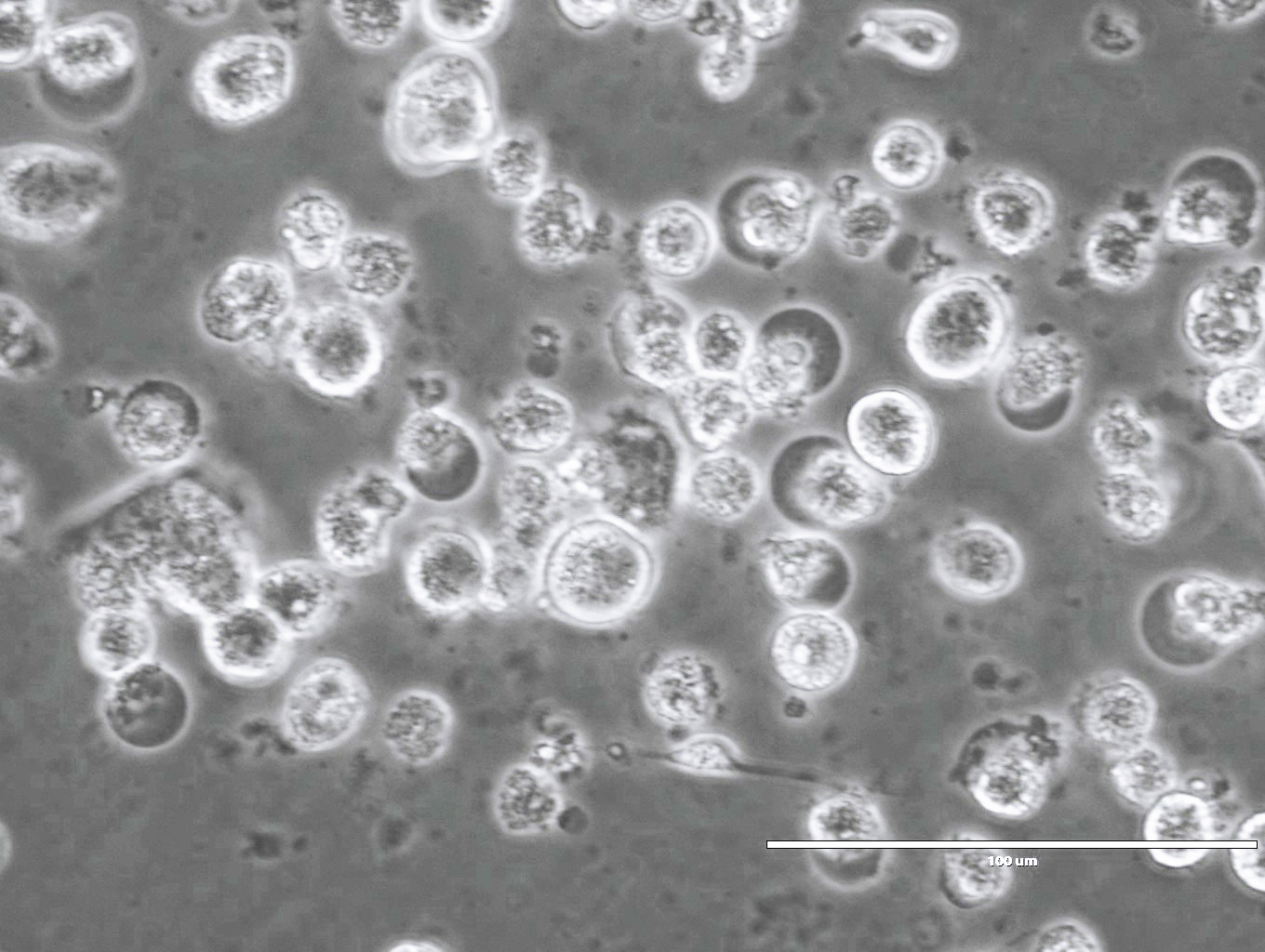

Supplement: Supplementary file 33 — Source data EV and Appendix [file 44318_2025_540_MOESM33_ESM.zip › Source data EV and Appendix/Figure EV 1/1E/HT-29/HT-29_24 h.jpg]

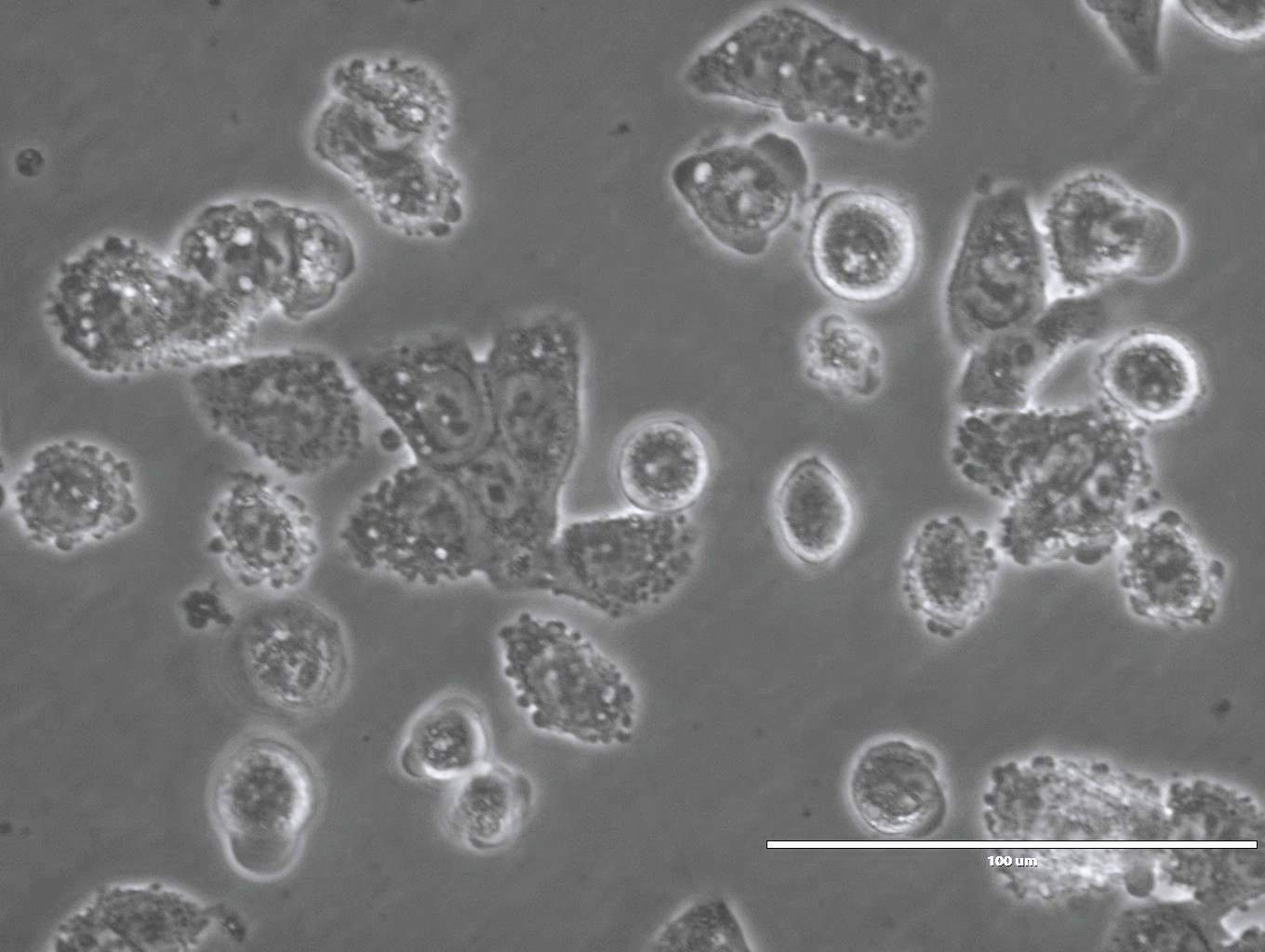

Supplement: Supplementary file 33 — Source data EV and Appendix [file 44318_2025_540_MOESM33_ESM.zip › Source data EV and Appendix/Figure EV 1/1E/HT-29/HT-29_30 min.jpg]

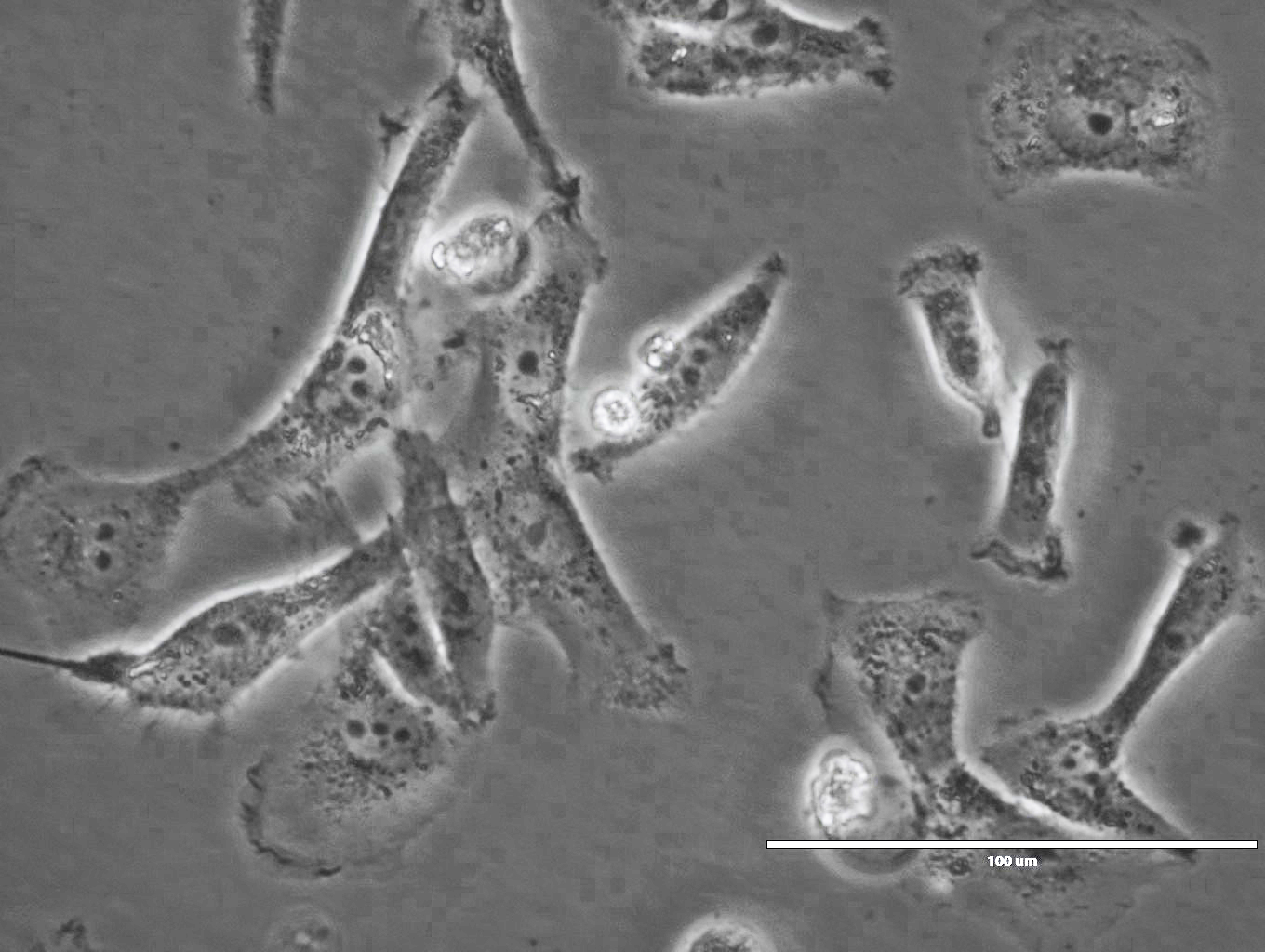

Supplement: Supplementary file 33 — Source data EV and Appendix [file 44318_2025_540_MOESM33_ESM.zip › Source data EV and Appendix/Figure EV 1/1E/MDA-MB-231/MDA-MB-231_0 h.jpg]

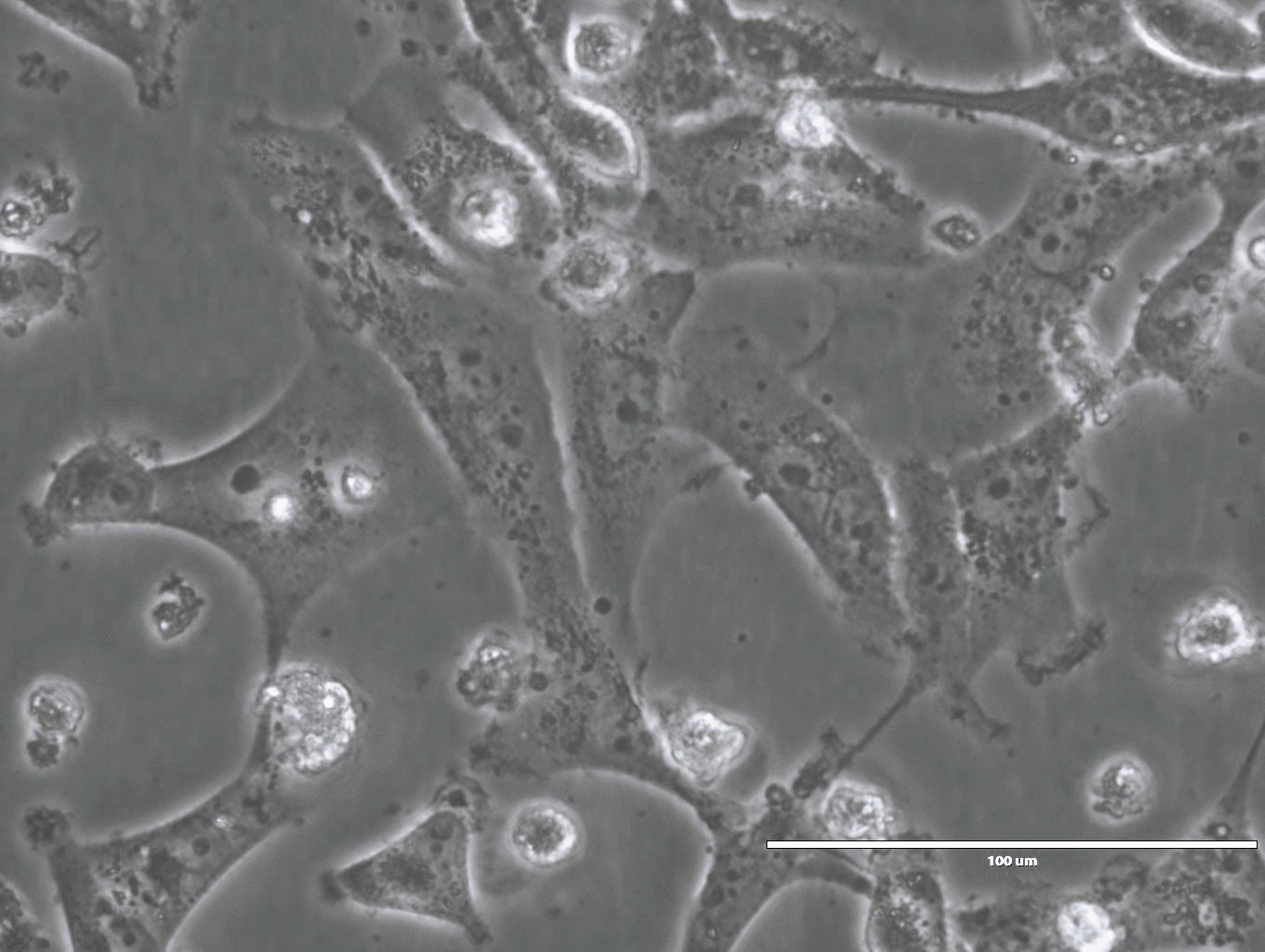

Supplement: Supplementary file 33 — Source data EV and Appendix [file 44318_2025_540_MOESM33_ESM.zip › Source data EV and Appendix/Figure EV 1/1E/MDA-MB-231/MDA-MB-231_24 h (1).jpg]

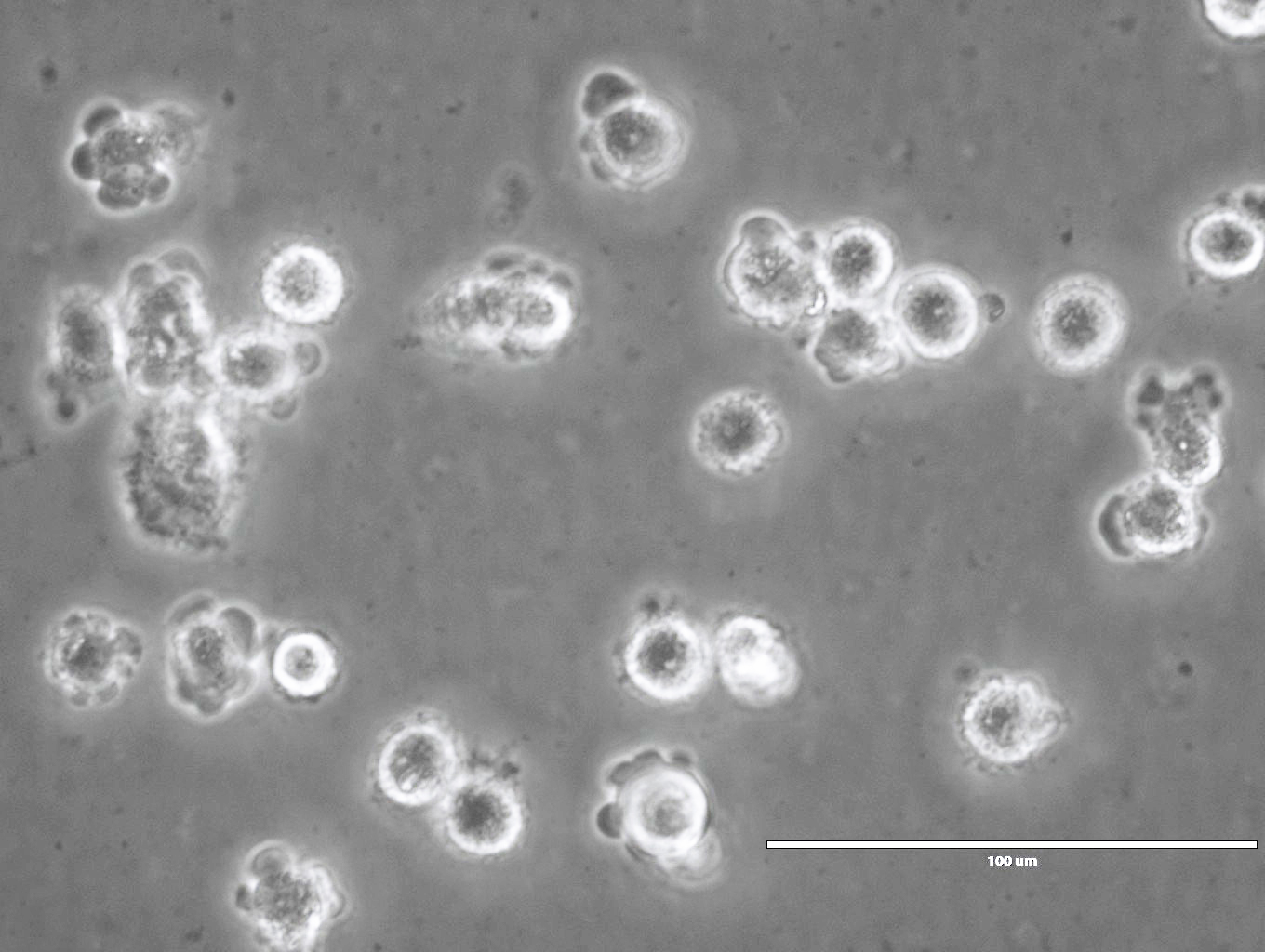

Supplement: Supplementary file 33 — Source data EV and Appendix [file 44318_2025_540_MOESM33_ESM.zip › Source data EV and Appendix/Figure EV 1/1E/MDA-MB-231/MDA-MB-231_30 min.jpg]

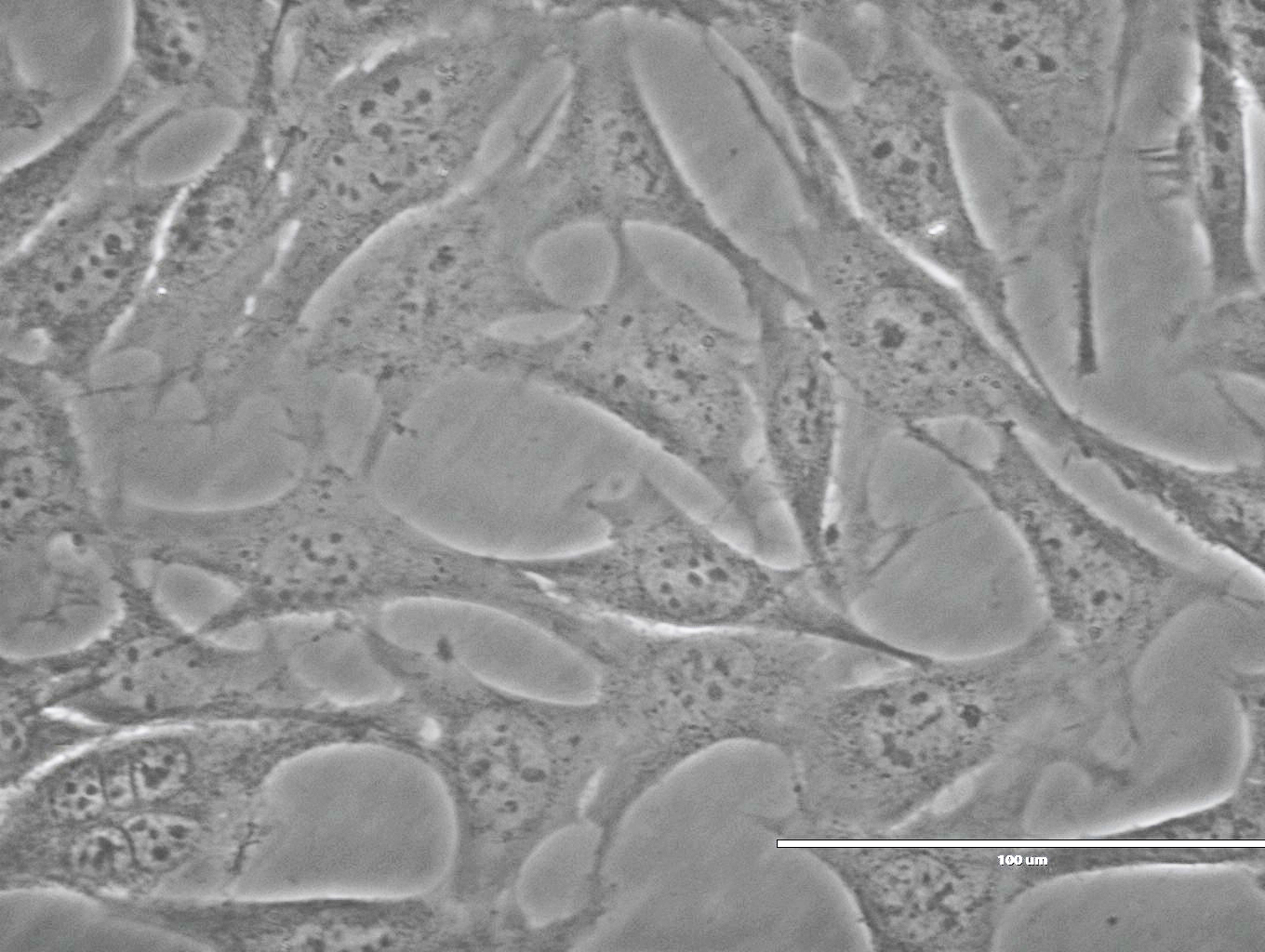

Supplement: Supplementary file 33 — Source data EV and Appendix [file 44318_2025_540_MOESM33_ESM.zip › Source data EV and Appendix/Figure EV 1/1E/MEF (1)/MEF_0 h.jpg]

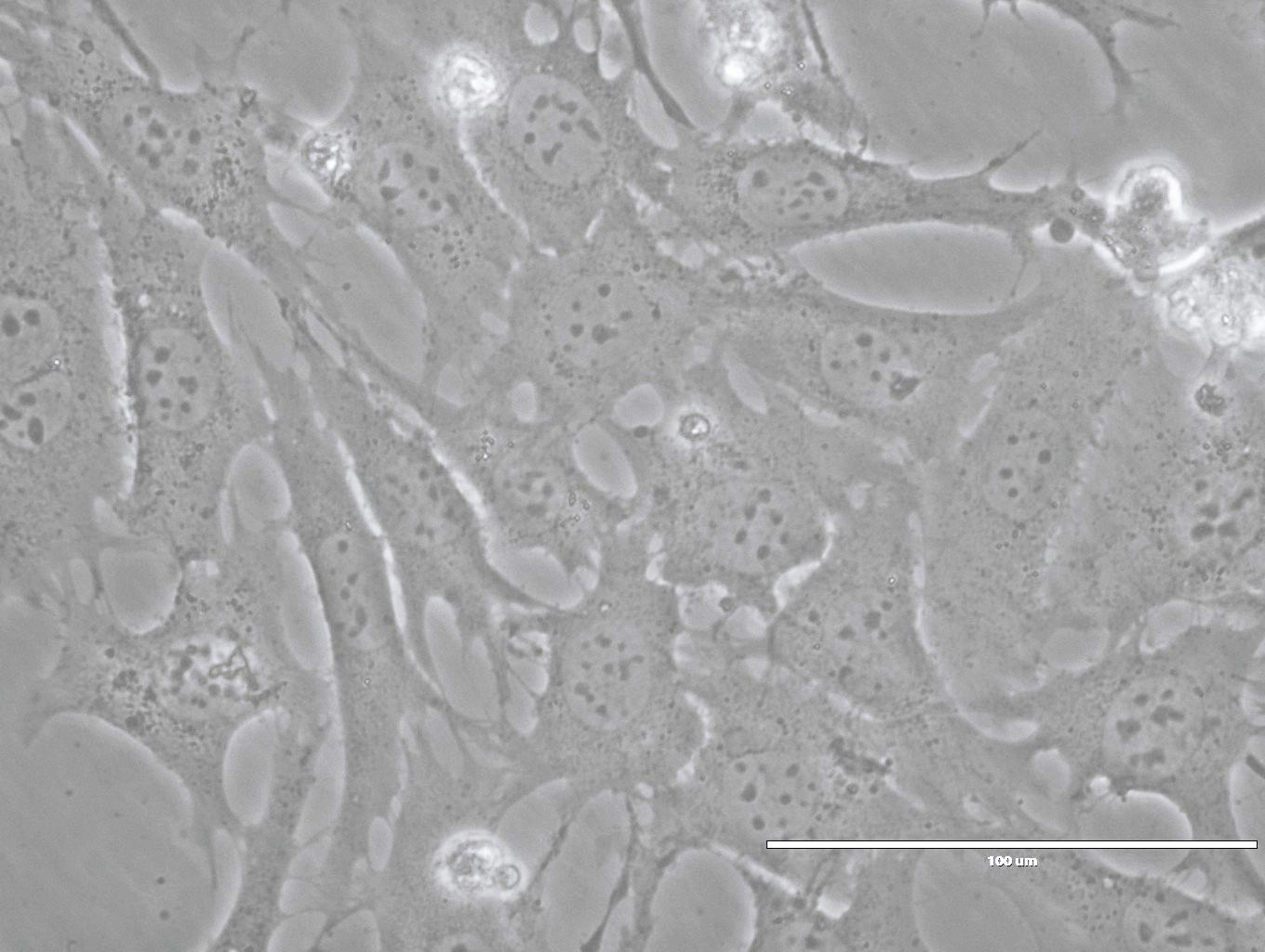

Supplement: Supplementary file 33 — Source data EV and Appendix [file 44318_2025_540_MOESM33_ESM.zip › Source data EV and Appendix/Figure EV 1/1E/MEF (1)/MEF_24 h.jpg]

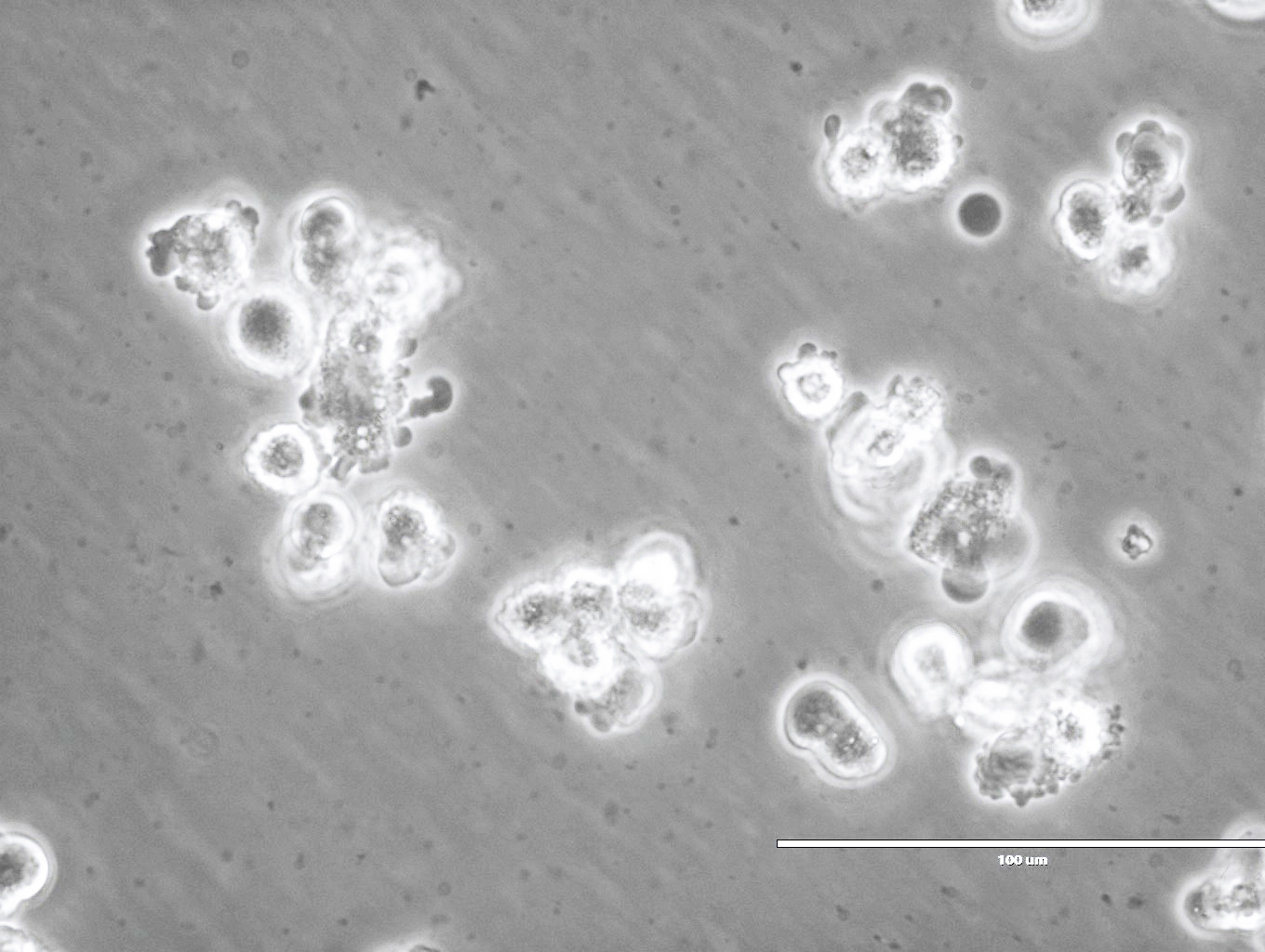

Supplement: Supplementary file 33 — Source data EV and Appendix [file 44318_2025_540_MOESM33_ESM.zip › Source data EV and Appendix/Figure EV 1/1E/MEF (1)/MEF_30 min.jpg]

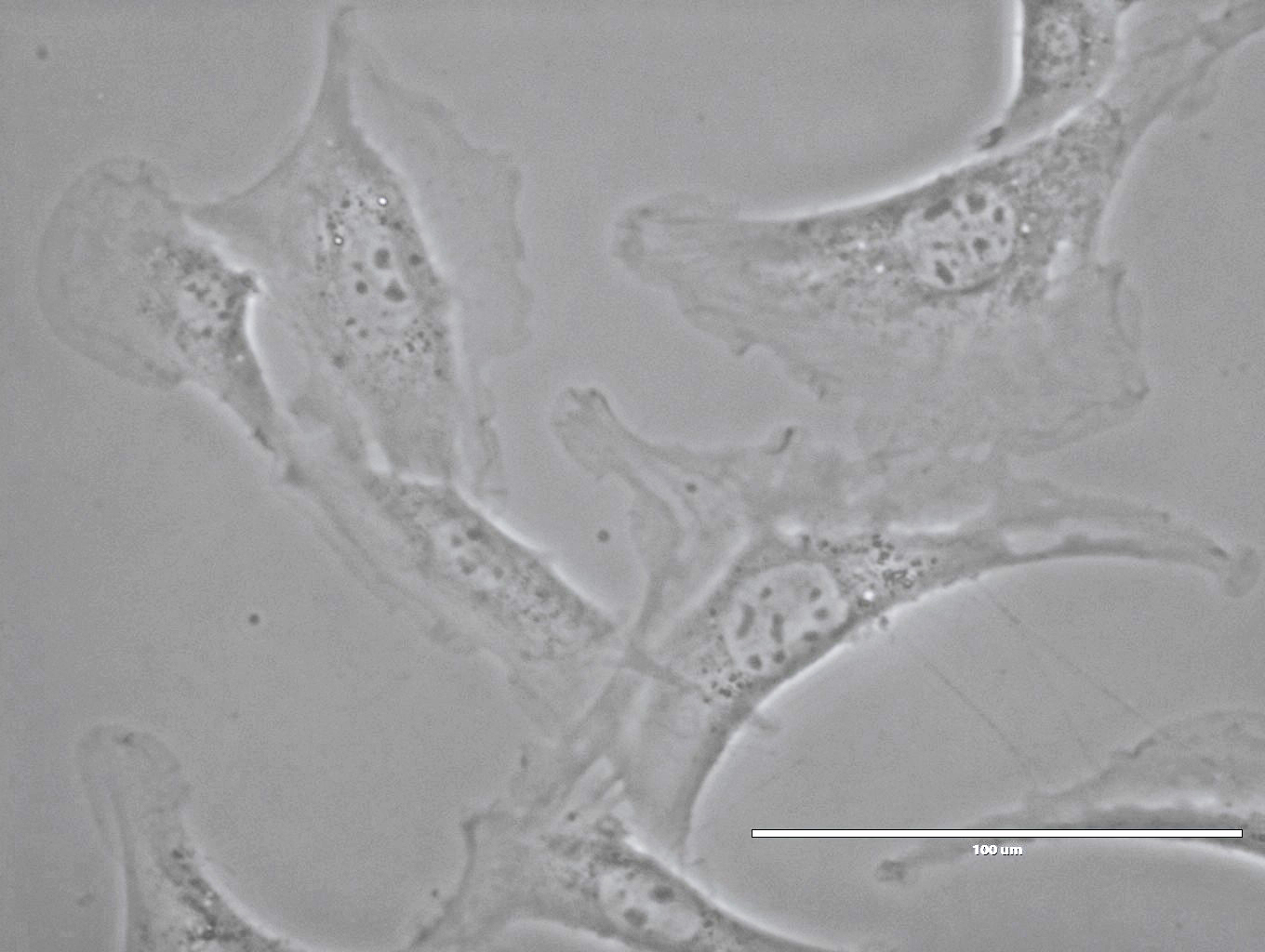

Supplement: Supplementary file 33 — Source data EV and Appendix [file 44318_2025_540_MOESM33_ESM.zip › Source data EV and Appendix/Figure EV 1/1E/NIH-3T3/NIH-3T3_0 h.jpg]

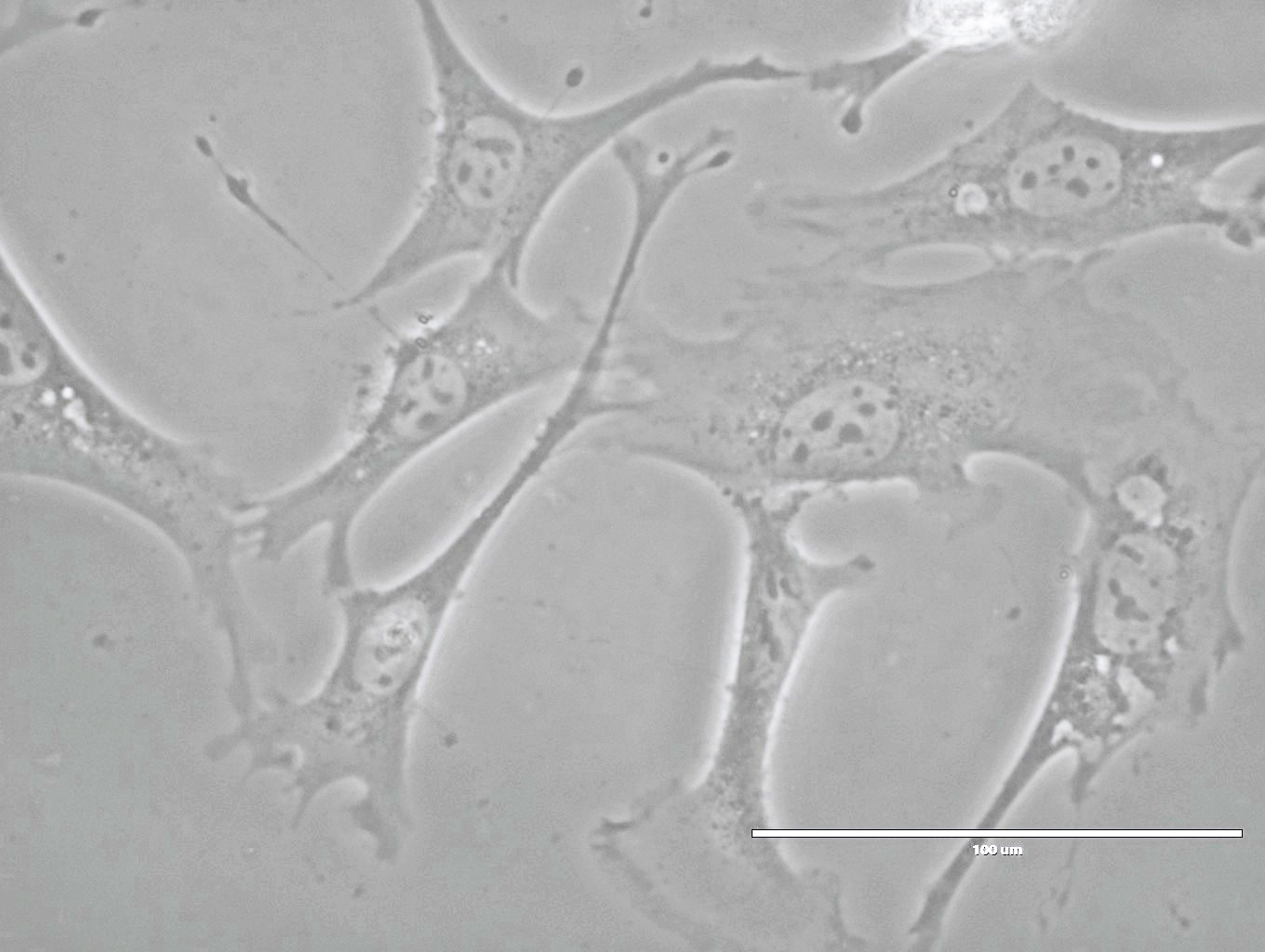

Supplement: Supplementary file 33 — Source data EV and Appendix [file 44318_2025_540_MOESM33_ESM.zip › Source data EV and Appendix/Figure EV 1/1E/NIH-3T3/NIH-3T3_24 h.jpg]

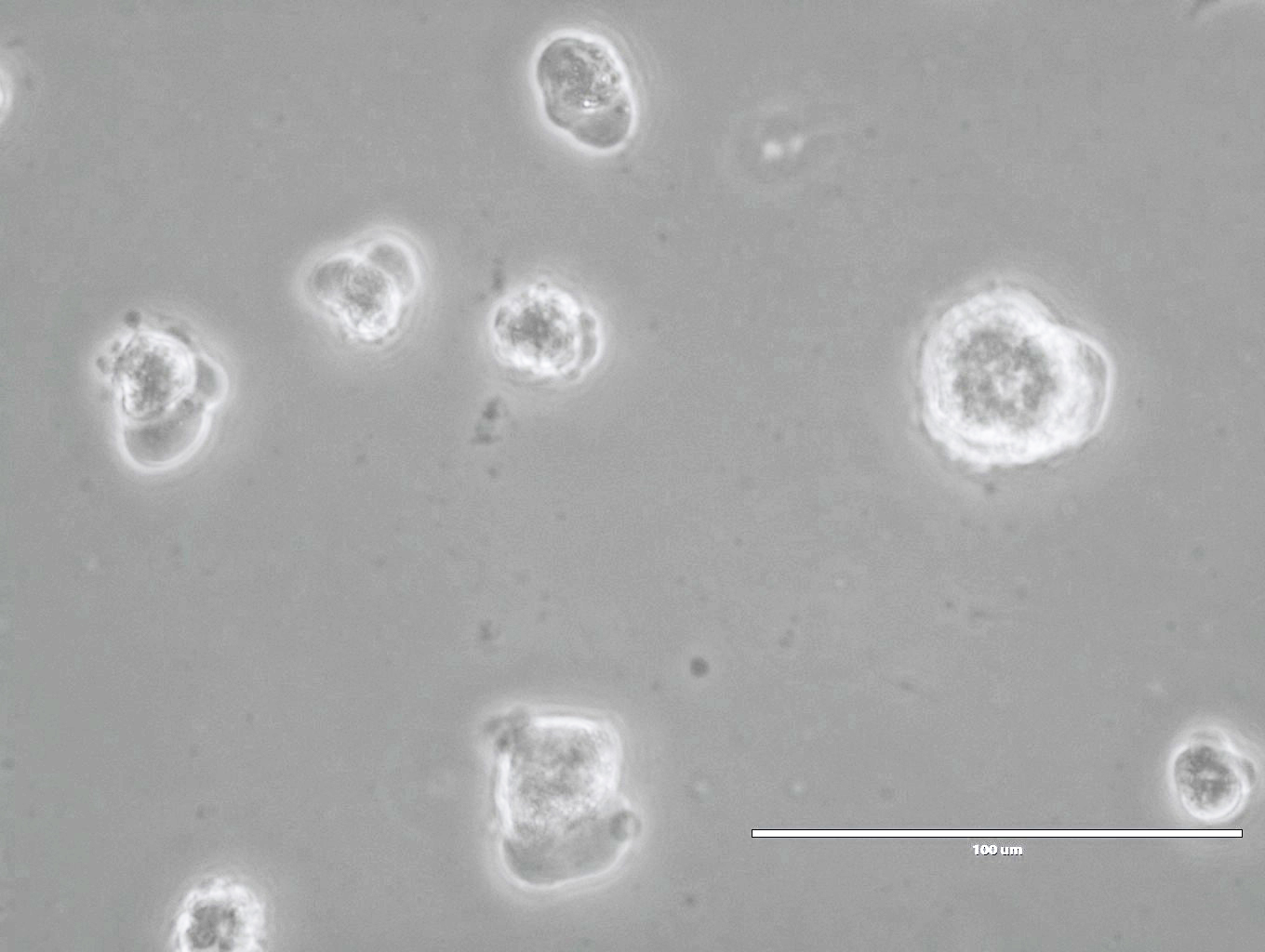

Supplement: Supplementary file 33 — Source data EV and Appendix [file 44318_2025_540_MOESM33_ESM.zip › Source data EV and Appendix/Figure EV 1/1E/NIH-3T3/NIH-3T3_30 min.jpg]

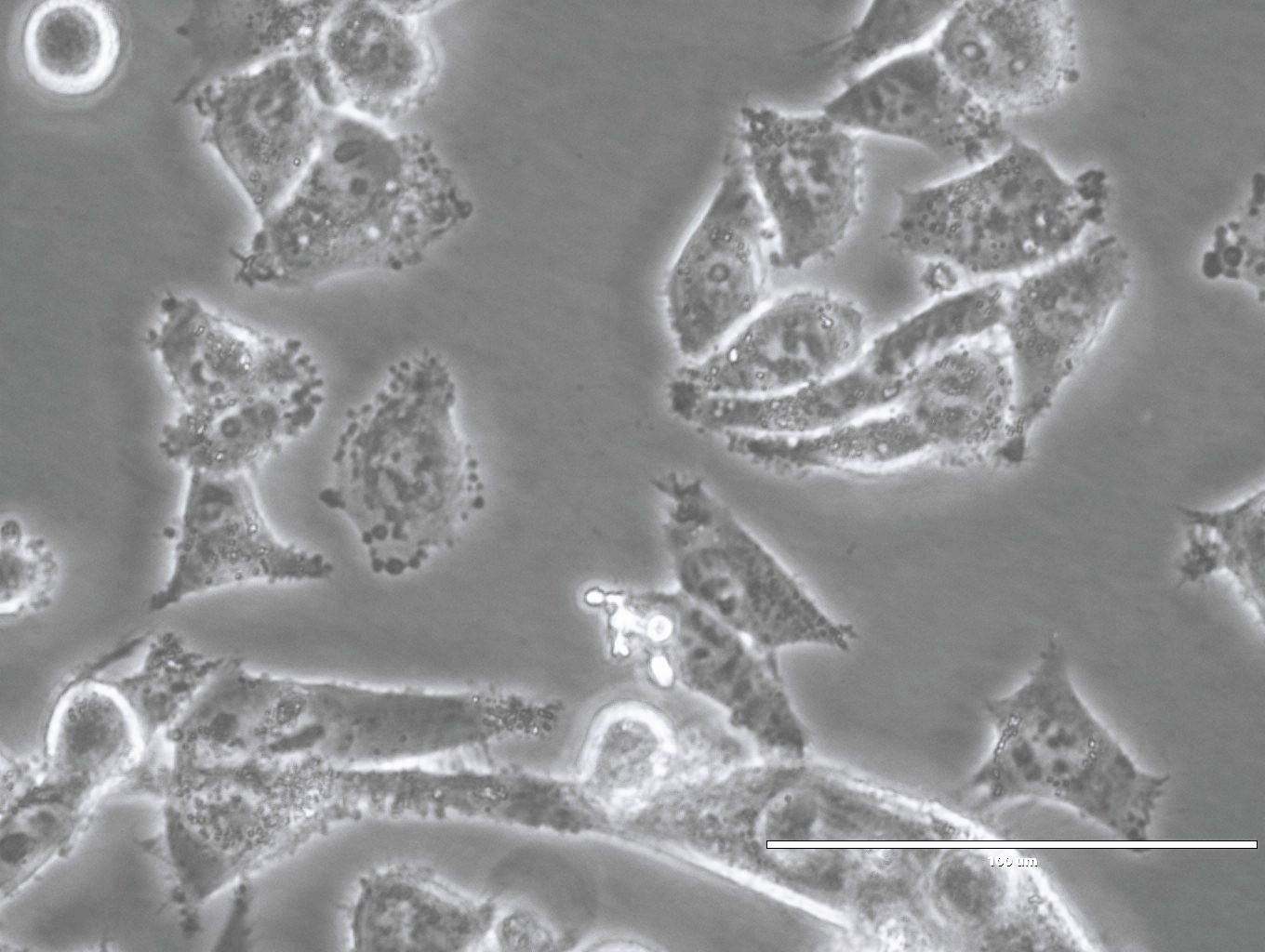

Supplement: Supplementary file 33 — Source data EV and Appendix [file 44318_2025_540_MOESM33_ESM.zip › Source data EV and Appendix/Figure EV 1/1E/UM-UC3/UM-UC3_0 h.jpg]

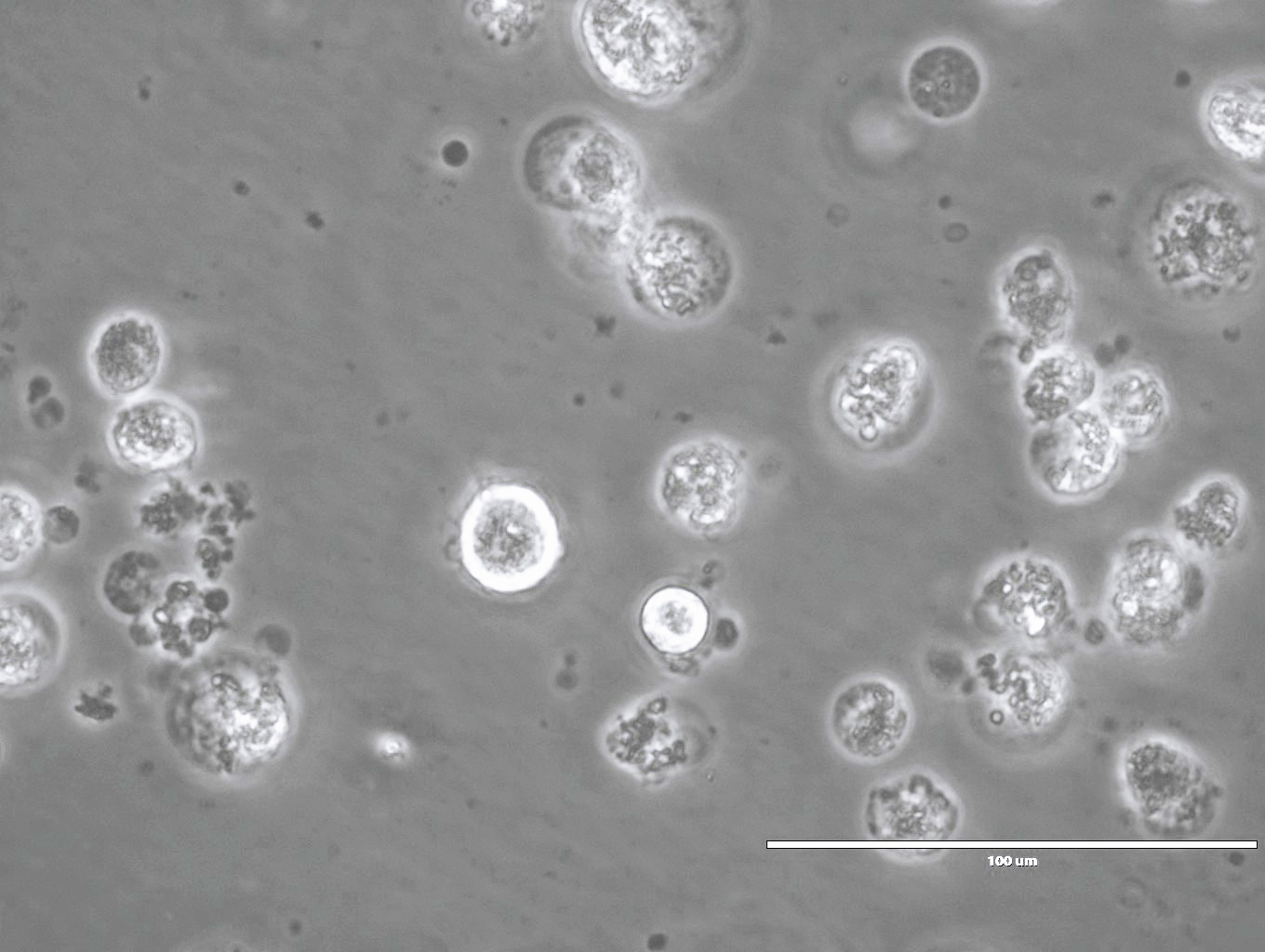

Supplement: Supplementary file 33 — Source data EV and Appendix [file 44318_2025_540_MOESM33_ESM.zip › Source data EV and Appendix/Figure EV 1/1E/UM-UC3/UM-UC3_24 h.jpg]

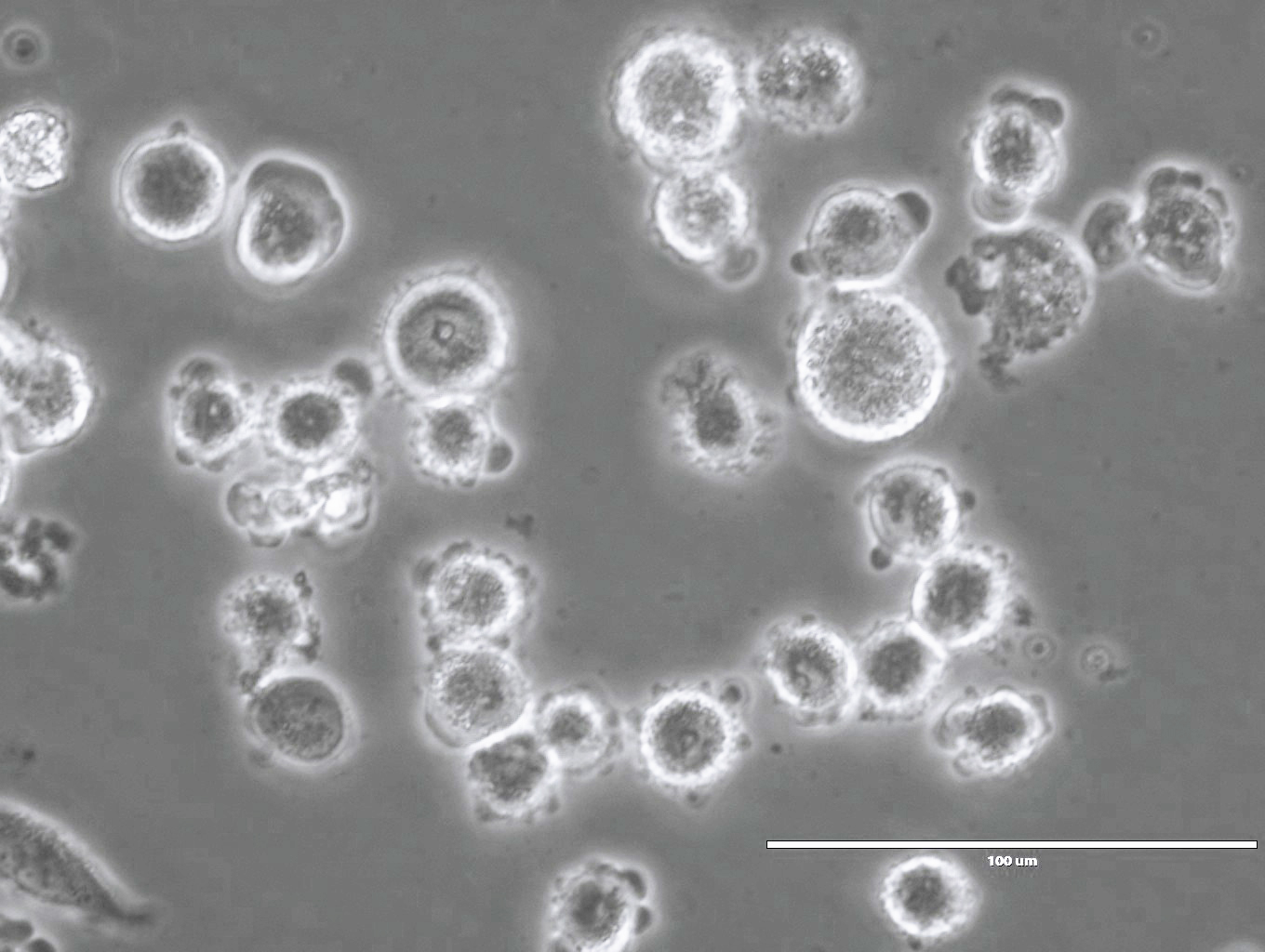

Supplement: Supplementary file 33 — Source data EV and Appendix [file 44318_2025_540_MOESM33_ESM.zip › Source data EV and Appendix/Figure EV 1/1E/UM-UC3/UM-UC3_30 min.jpg]

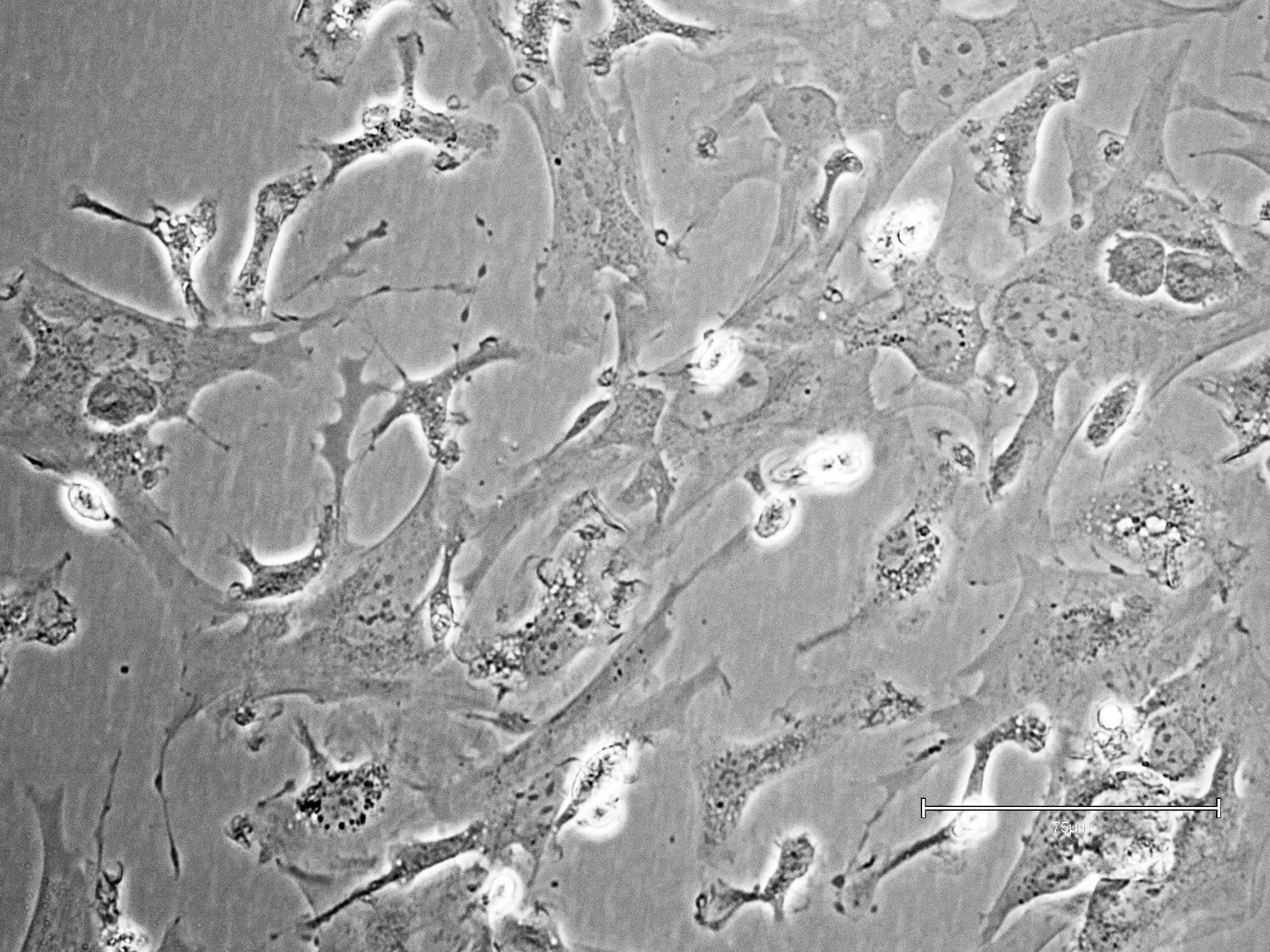

Supplement: Supplementary file 33 — Source data EV and Appendix [file 44318_2025_540_MOESM33_ESM.zip › Source data EV and Appendix/Figure EV 1/1G/0.25mM LLOMe/0H (1).jpg]

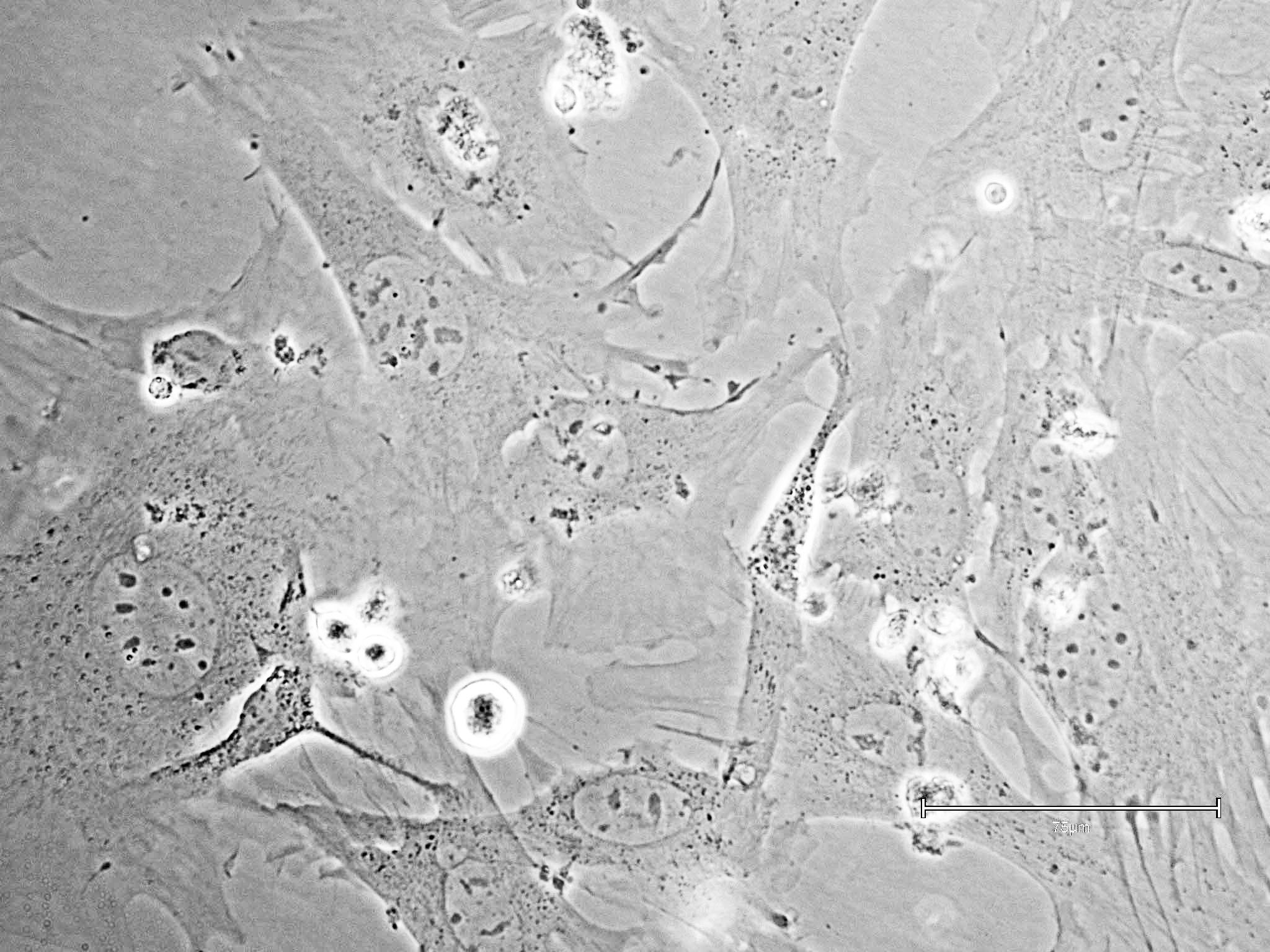

Supplement: Supplementary file 33 — Source data EV and Appendix [file 44318_2025_540_MOESM33_ESM.zip › Source data EV and Appendix/Figure EV 1/1G/0.25mM LLOMe/24H.jpg]

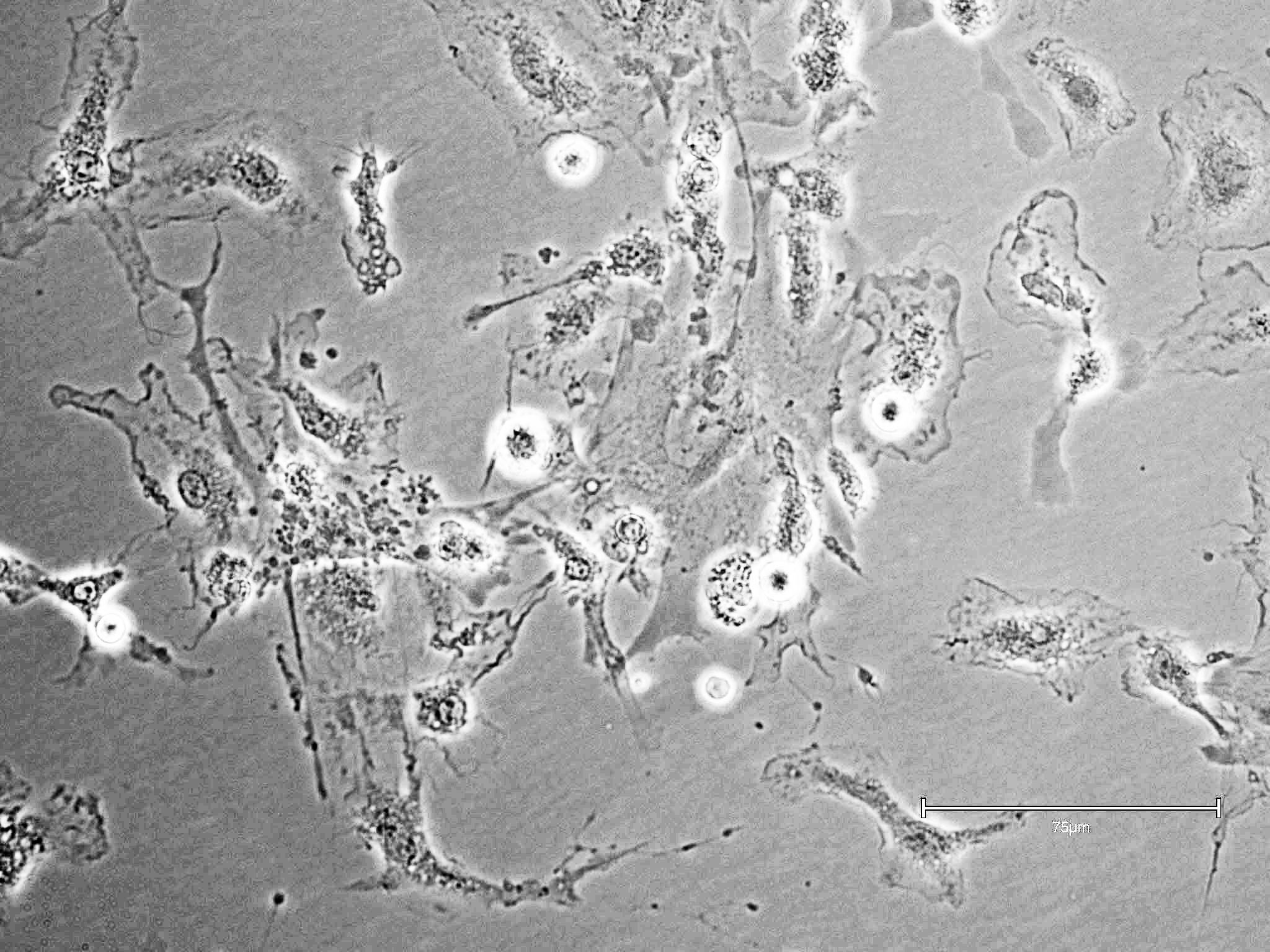

Supplement: Supplementary file 33 — Source data EV and Appendix [file 44318_2025_540_MOESM33_ESM.zip › Source data EV and Appendix/Figure EV 1/1G/0.25mM LLOMe/30MIN.jpg]

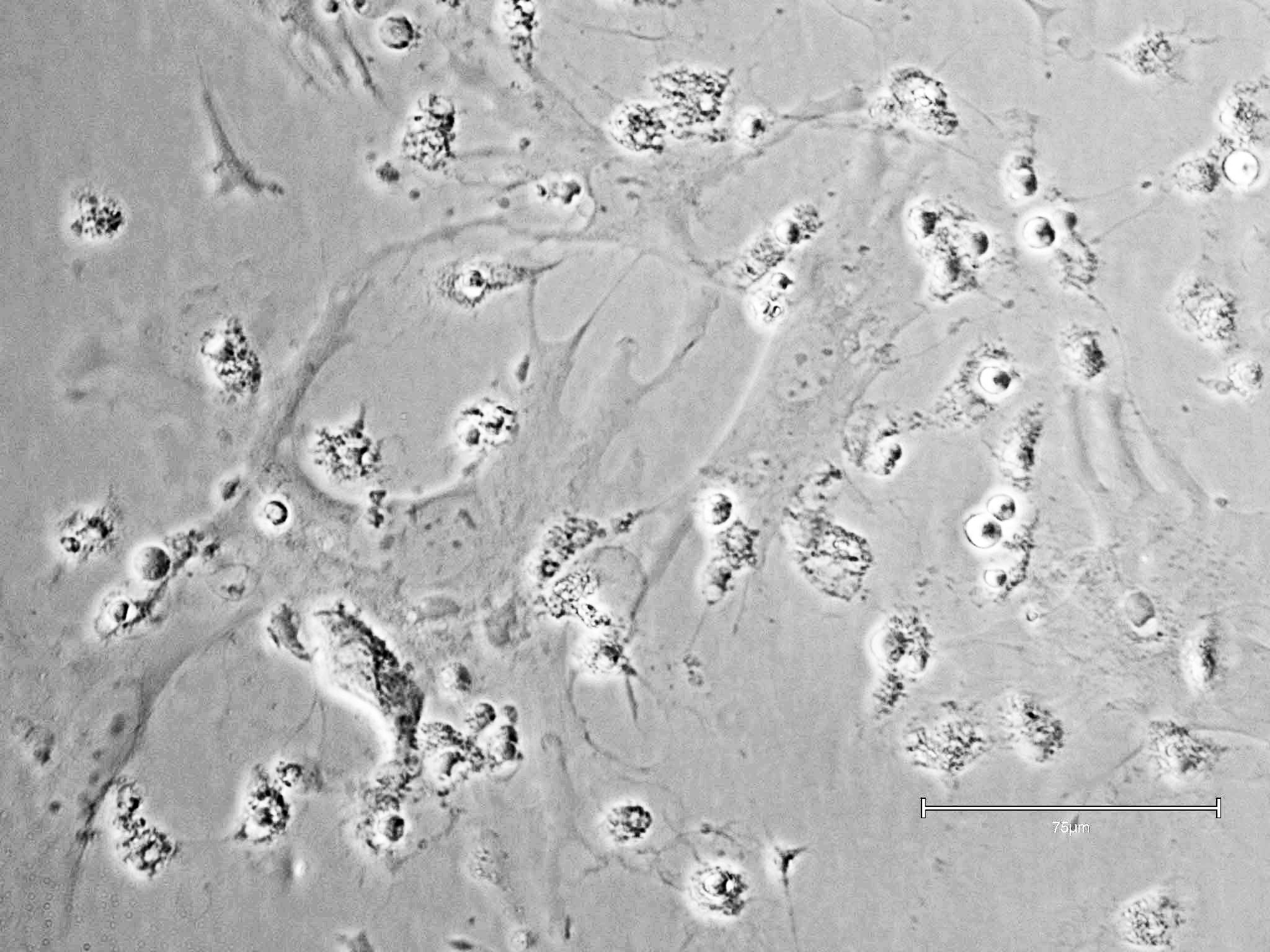

Supplement: Supplementary file 33 — Source data EV and Appendix [file 44318_2025_540_MOESM33_ESM.zip › Source data EV and Appendix/Figure EV 1/1G/0.25mM LLOMe/3H.jpg]

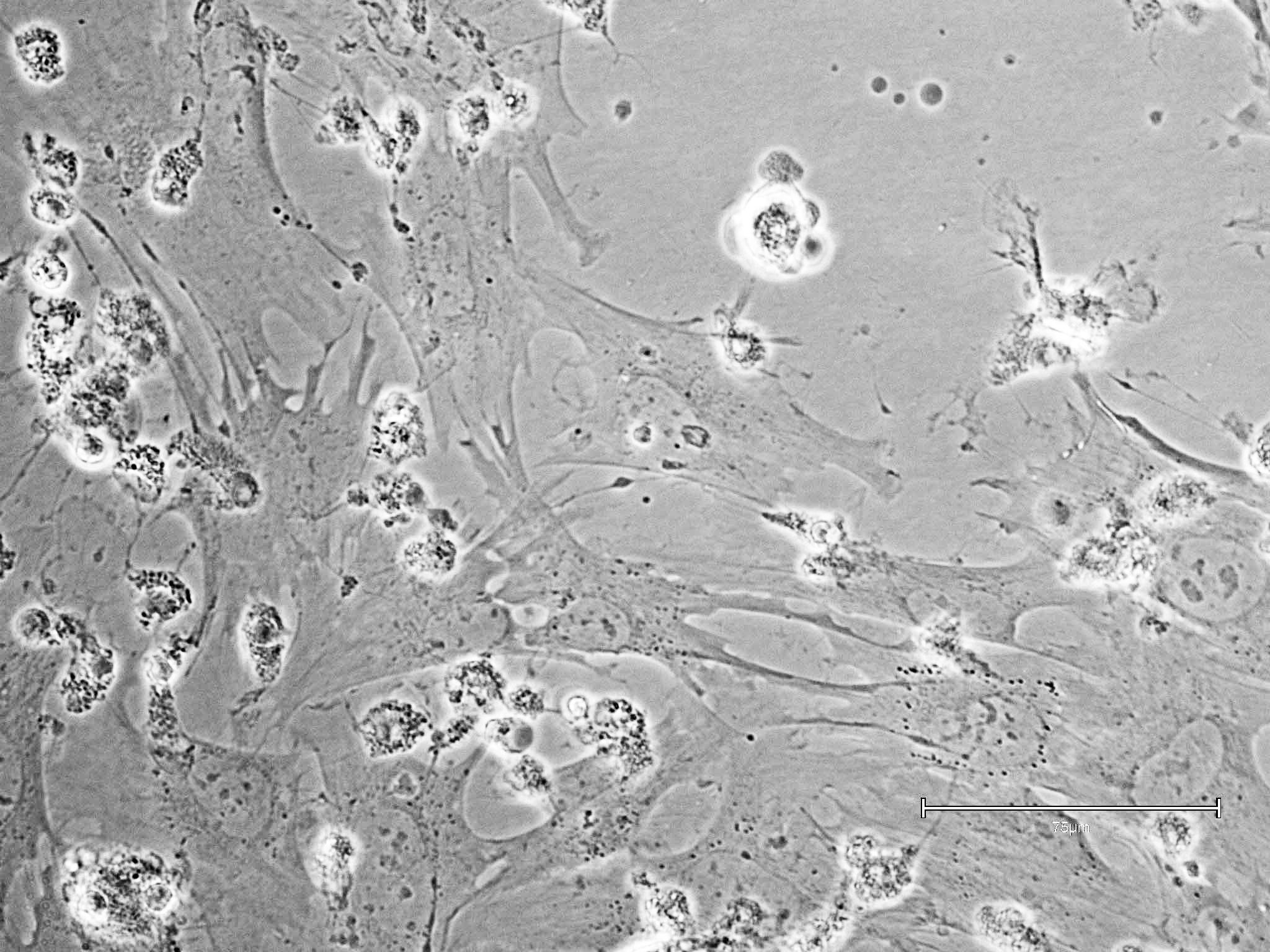

Supplement: Supplementary file 33 — Source data EV and Appendix [file 44318_2025_540_MOESM33_ESM.zip › Source data EV and Appendix/Figure EV 1/1G/0.25mM LLOMe/6H.jpg]

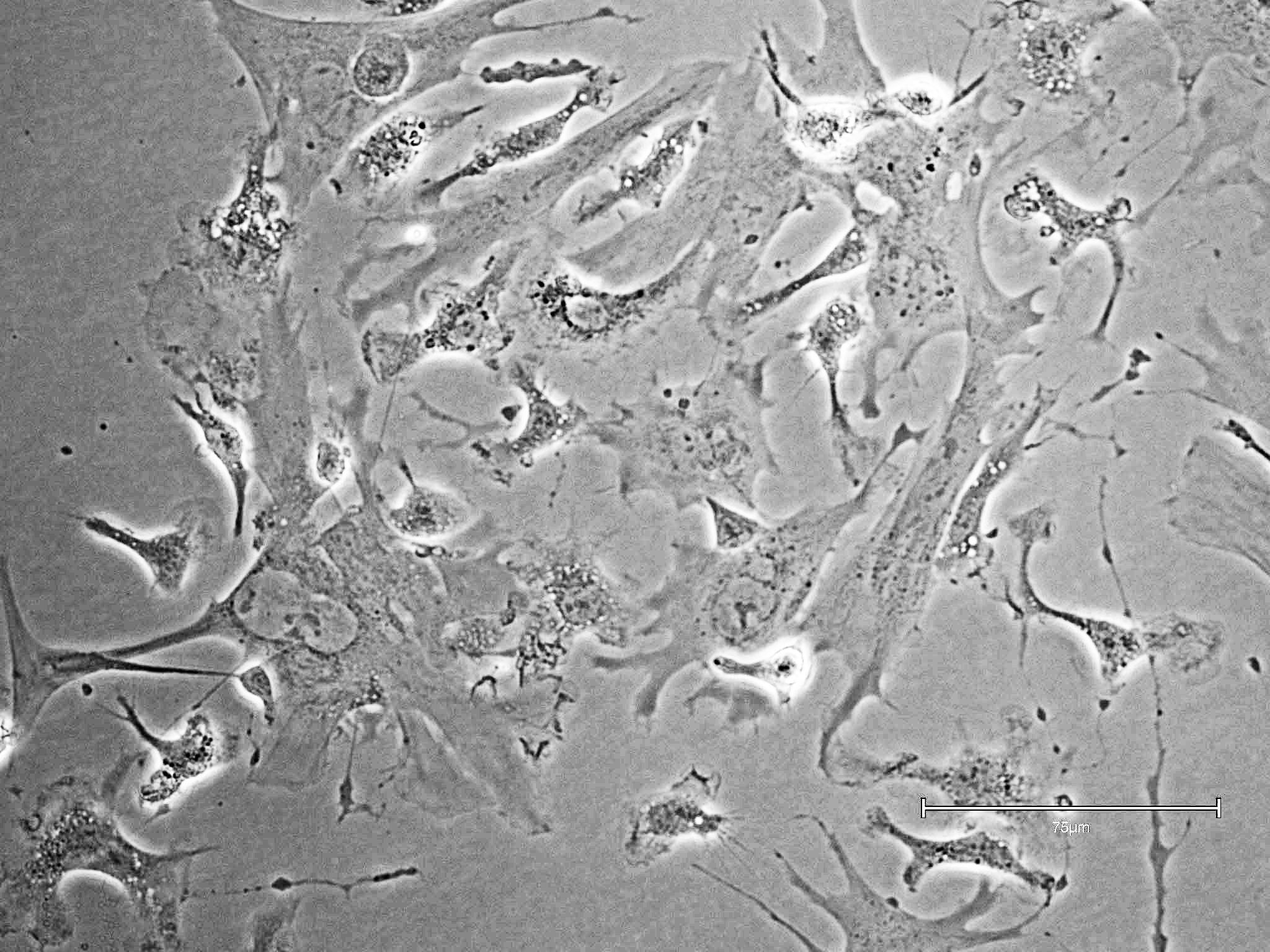

Supplement: Supplementary file 33 — Source data EV and Appendix [file 44318_2025_540_MOESM33_ESM.zip › Source data EV and Appendix/Figure EV 1/1G/0.5mM LLOMe/0H (1).jpg]

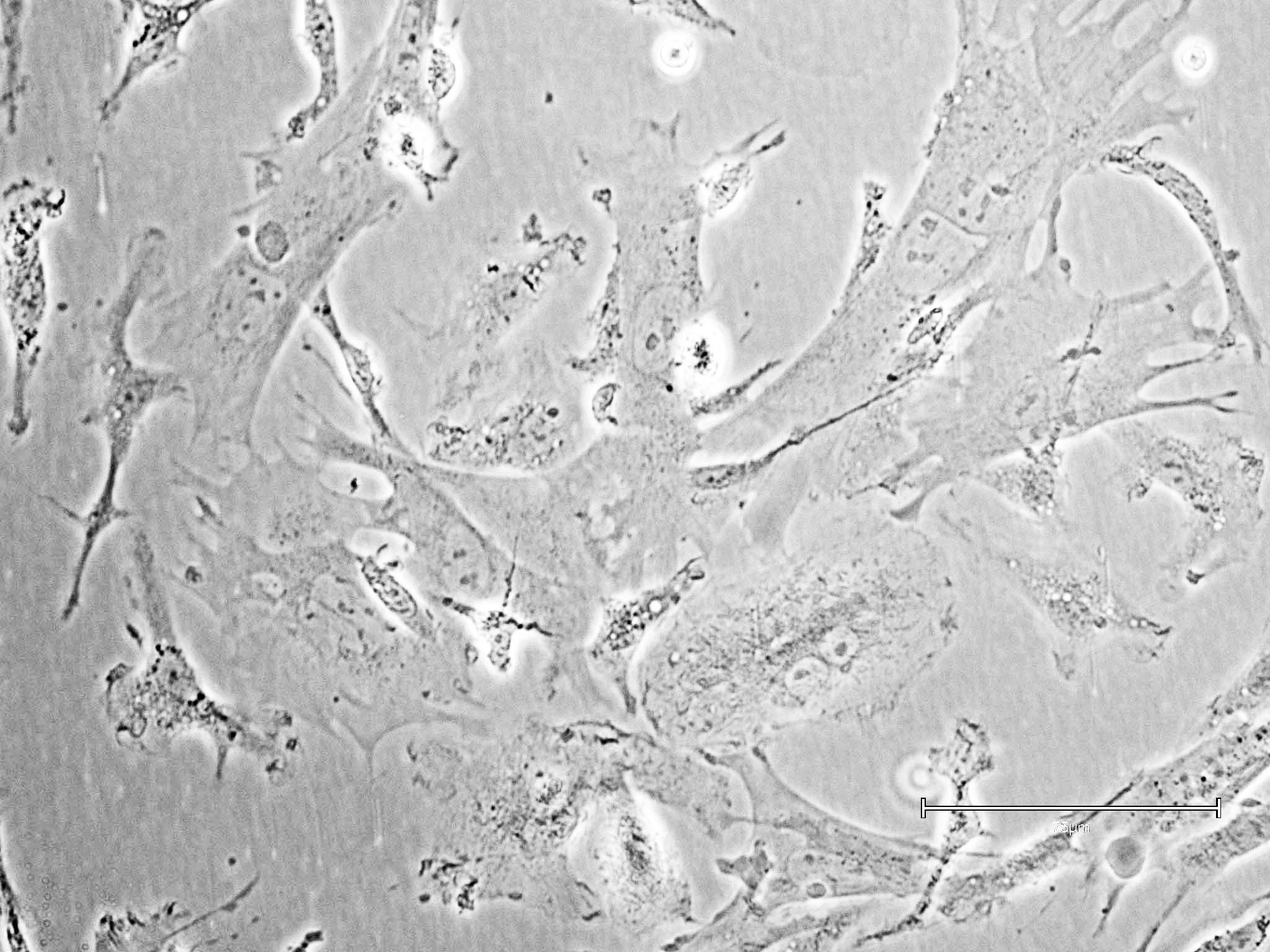

Supplement: Supplementary file 33 — Source data EV and Appendix [file 44318_2025_540_MOESM33_ESM.zip › Source data EV and Appendix/Figure EV 1/1G/0.5mM LLOMe/24H.jpg]

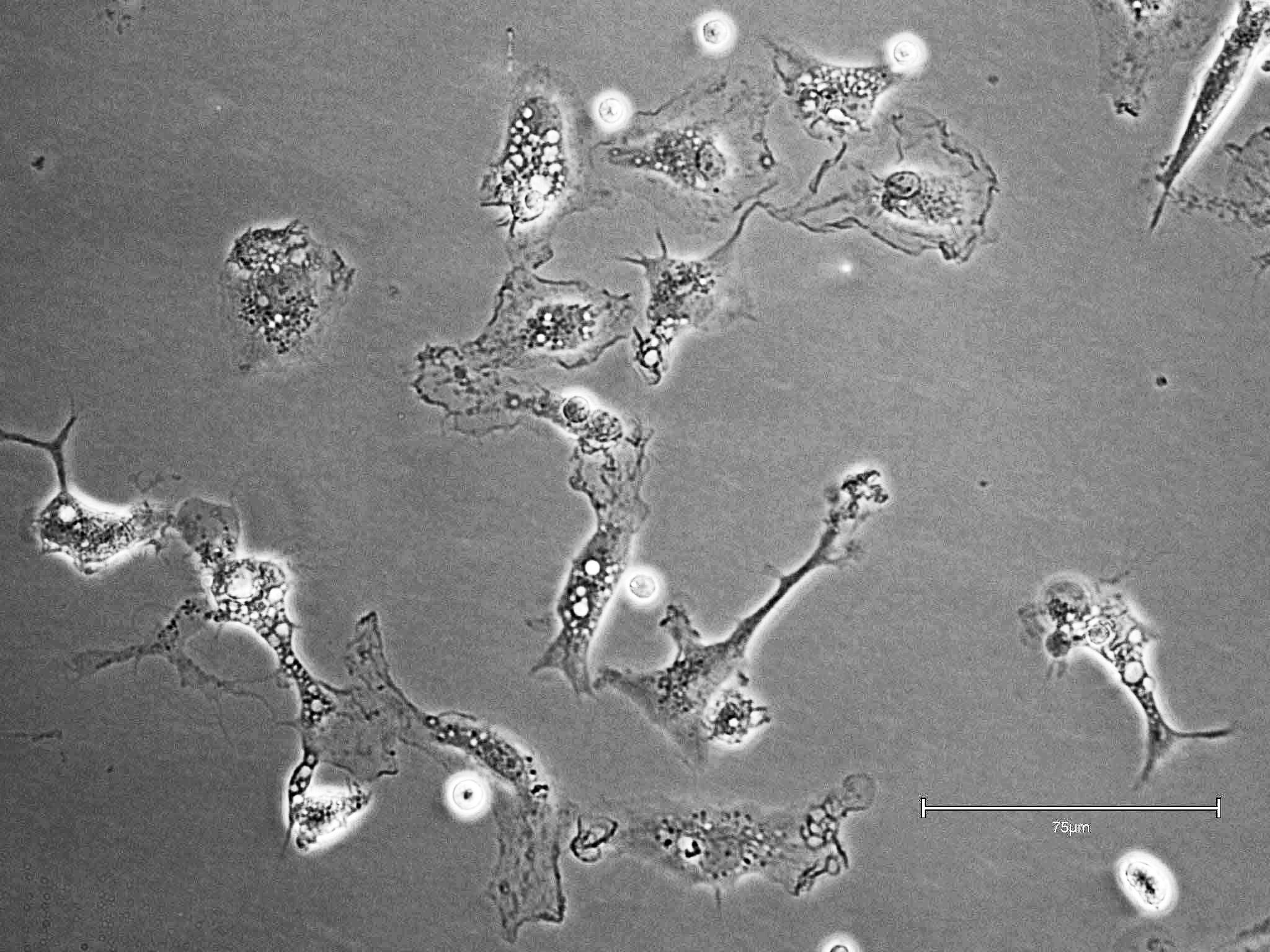

Supplement: Supplementary file 33 — Source data EV and Appendix [file 44318_2025_540_MOESM33_ESM.zip › Source data EV and Appendix/Figure EV 1/1G/0.5mM LLOMe/30 MINS.jpg]

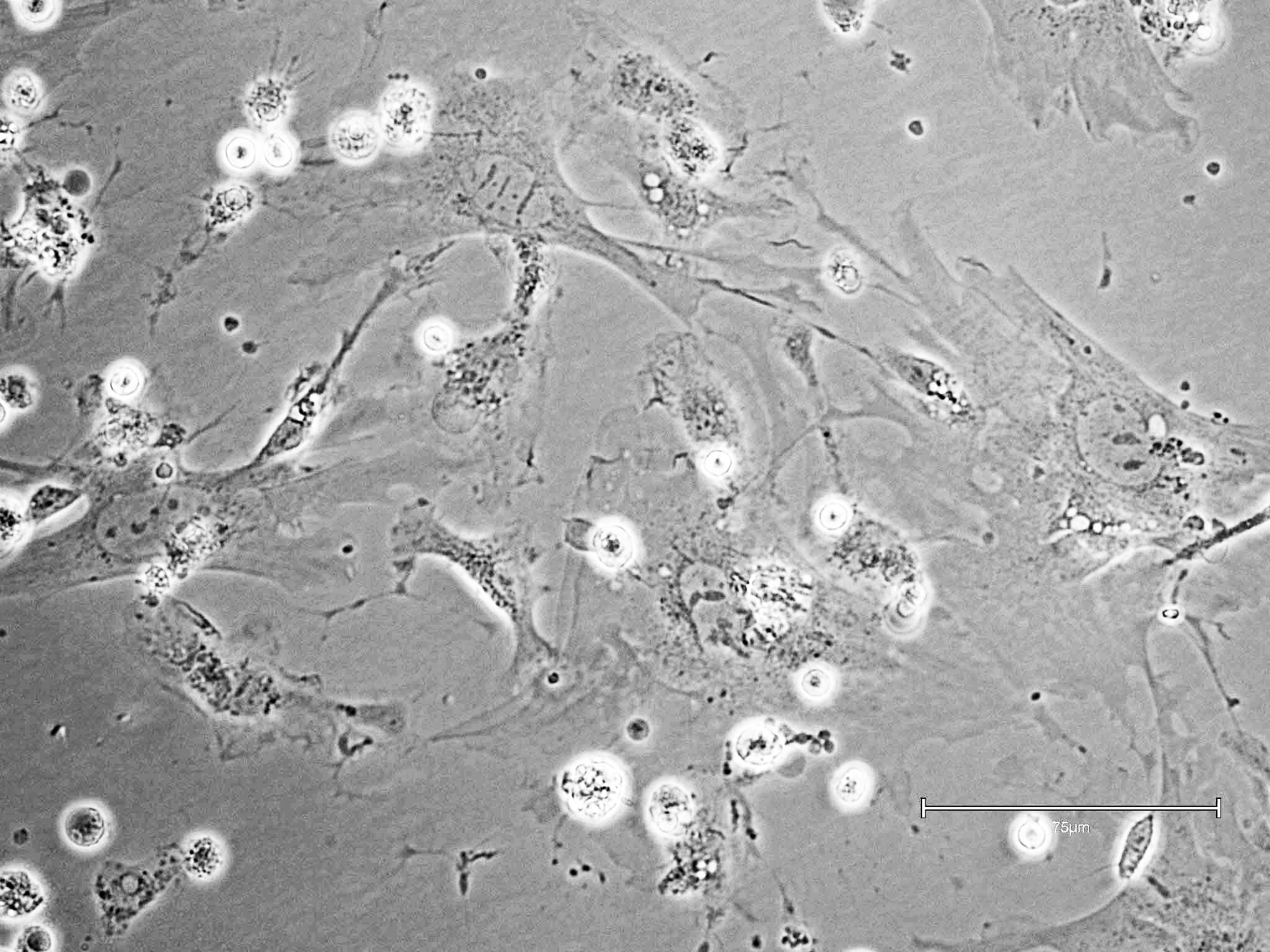

Supplement: Supplementary file 33 — Source data EV and Appendix [file 44318_2025_540_MOESM33_ESM.zip › Source data EV and Appendix/Figure EV 1/1G/0.5mM LLOMe/3H.jpg]

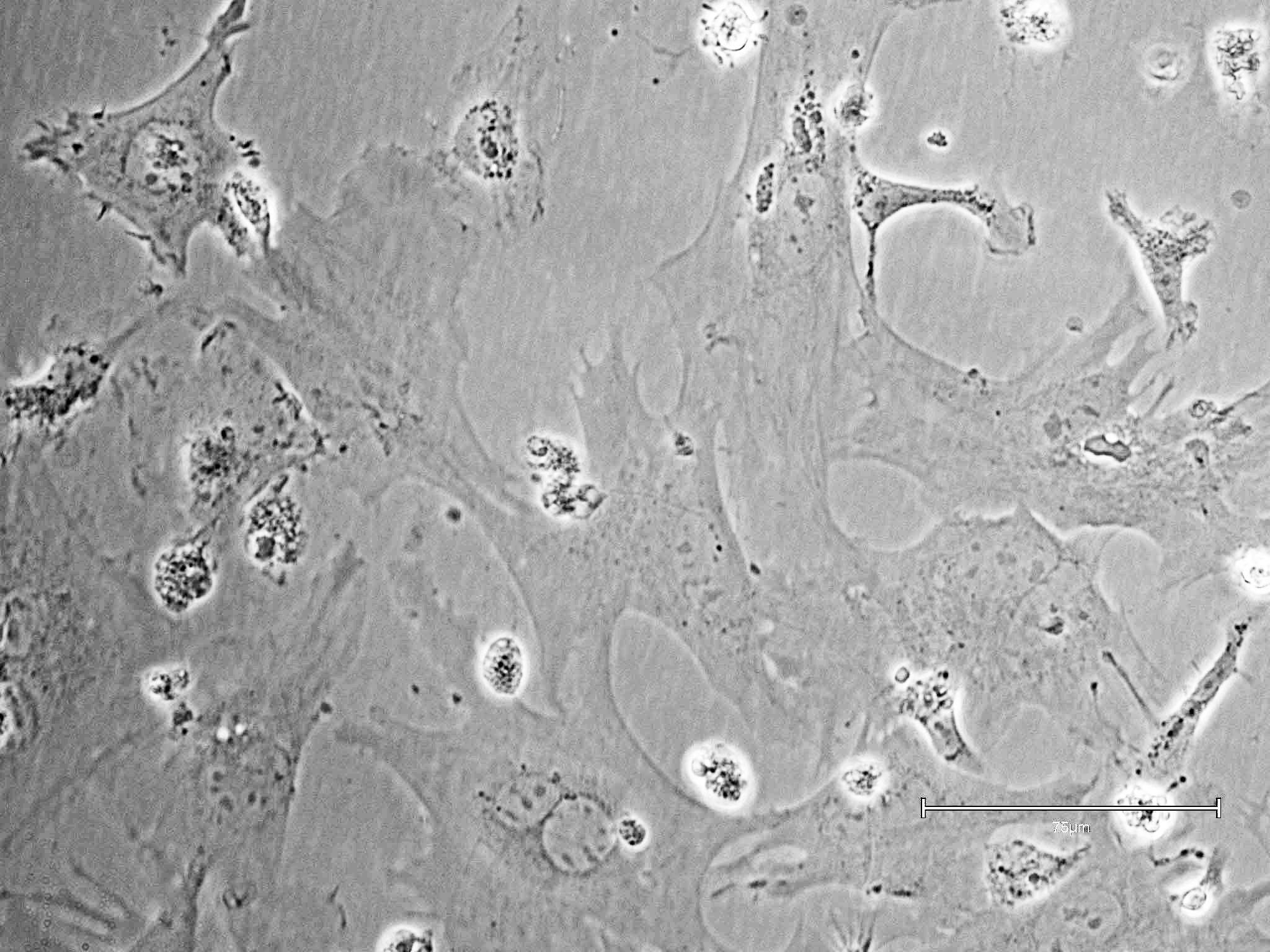

Supplement: Supplementary file 33 — Source data EV and Appendix [file 44318_2025_540_MOESM33_ESM.zip › Source data EV and Appendix/Figure EV 1/1G/0.5mM LLOMe/6H.jpg]

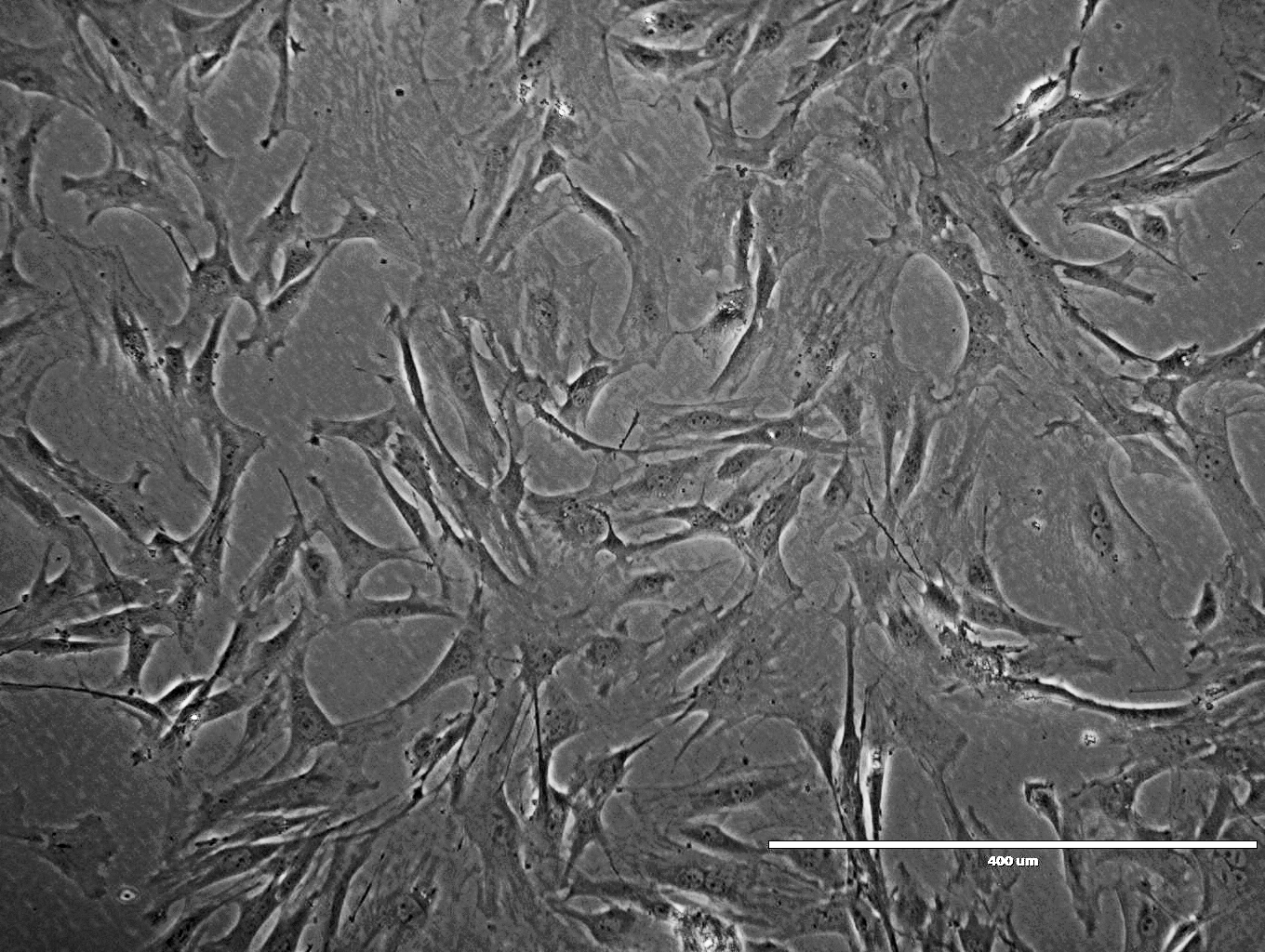

Supplement: Supplementary file 33 — Source data EV and Appendix [file 44318_2025_540_MOESM33_ESM.zip › Source data EV and Appendix/Figure EV 2/2A/0 h.jpg]

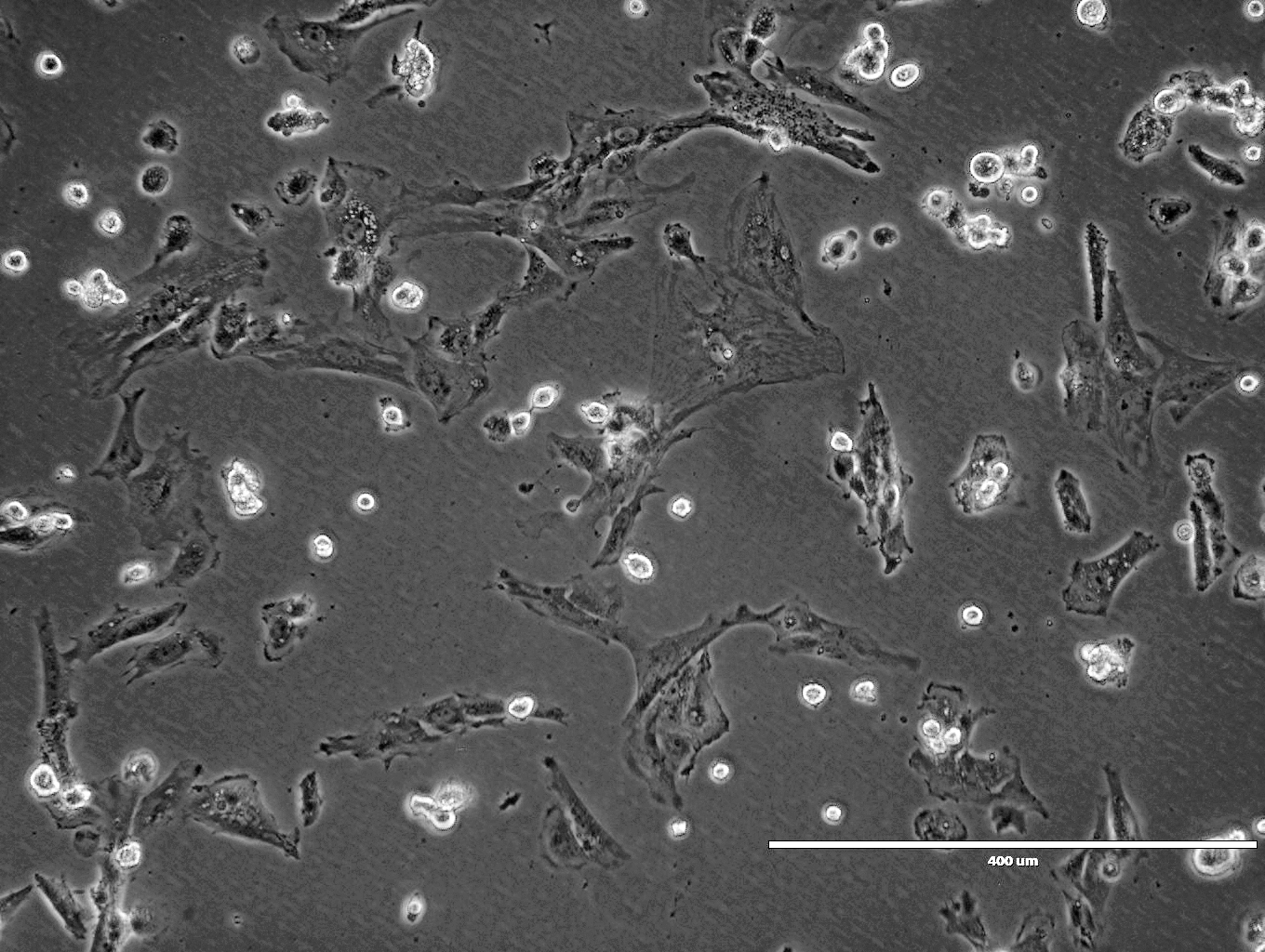

Supplement: Supplementary file 33 — Source data EV and Appendix [file 44318_2025_540_MOESM33_ESM.zip › Source data EV and Appendix/Figure EV 2/2A/1h.jpg]

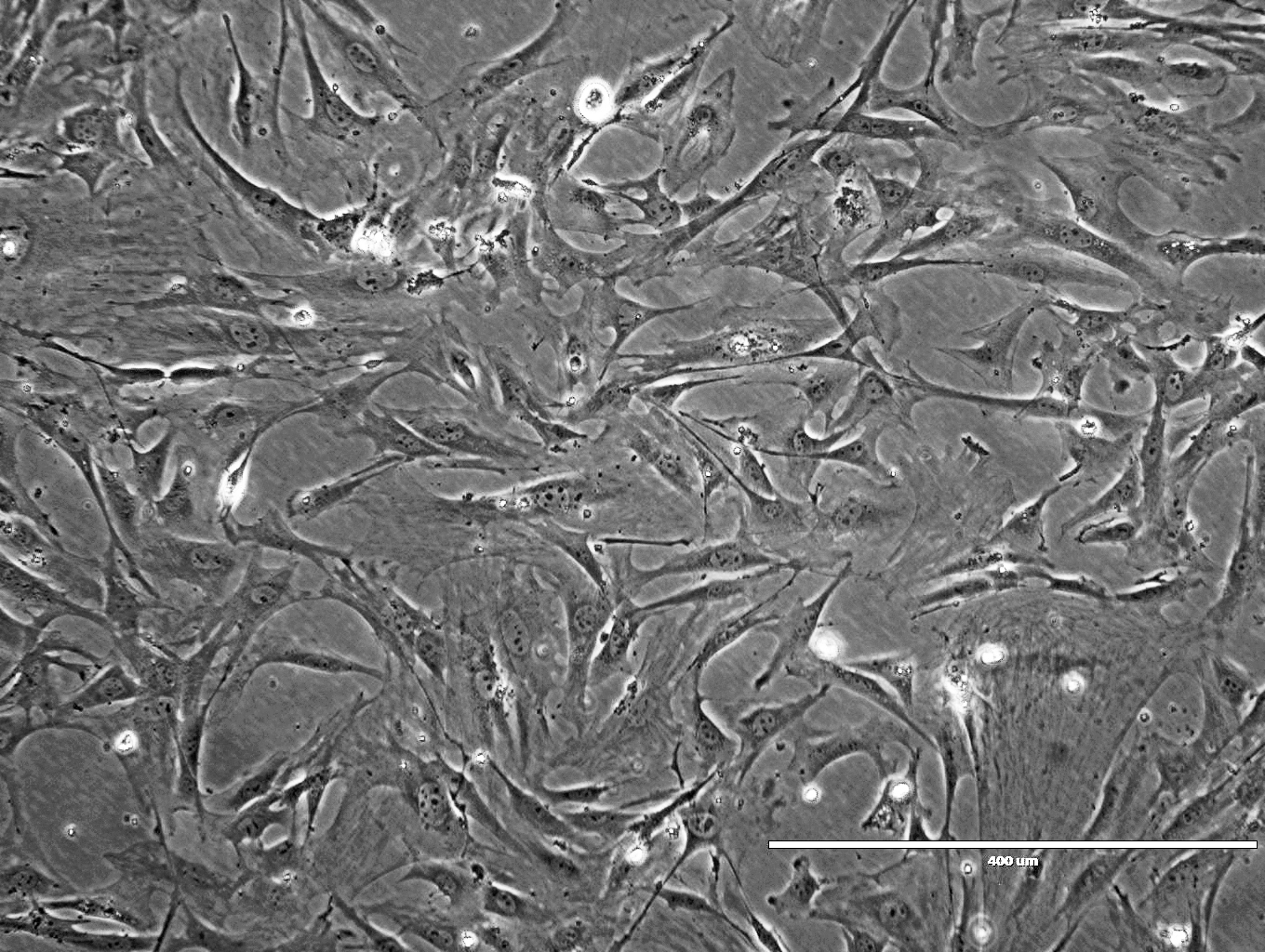

Supplement: Supplementary file 33 — Source data EV and Appendix [file 44318_2025_540_MOESM33_ESM.zip › Source data EV and Appendix/Figure EV 2/2A/24h.jpg]

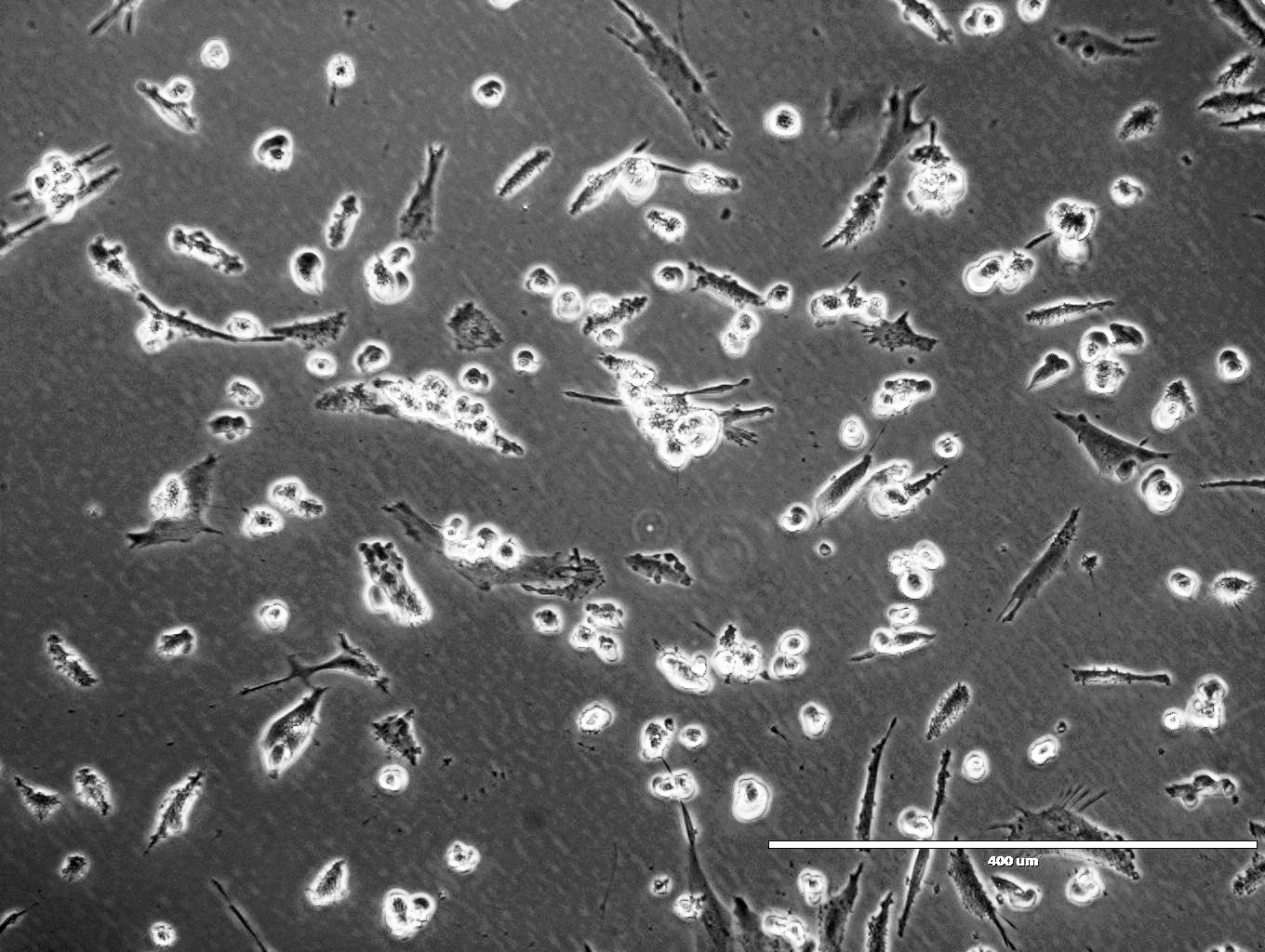

Supplement: Supplementary file 33 — Source data EV and Appendix [file 44318_2025_540_MOESM33_ESM.zip › Source data EV and Appendix/Figure EV 2/2A/30 min.jpg]

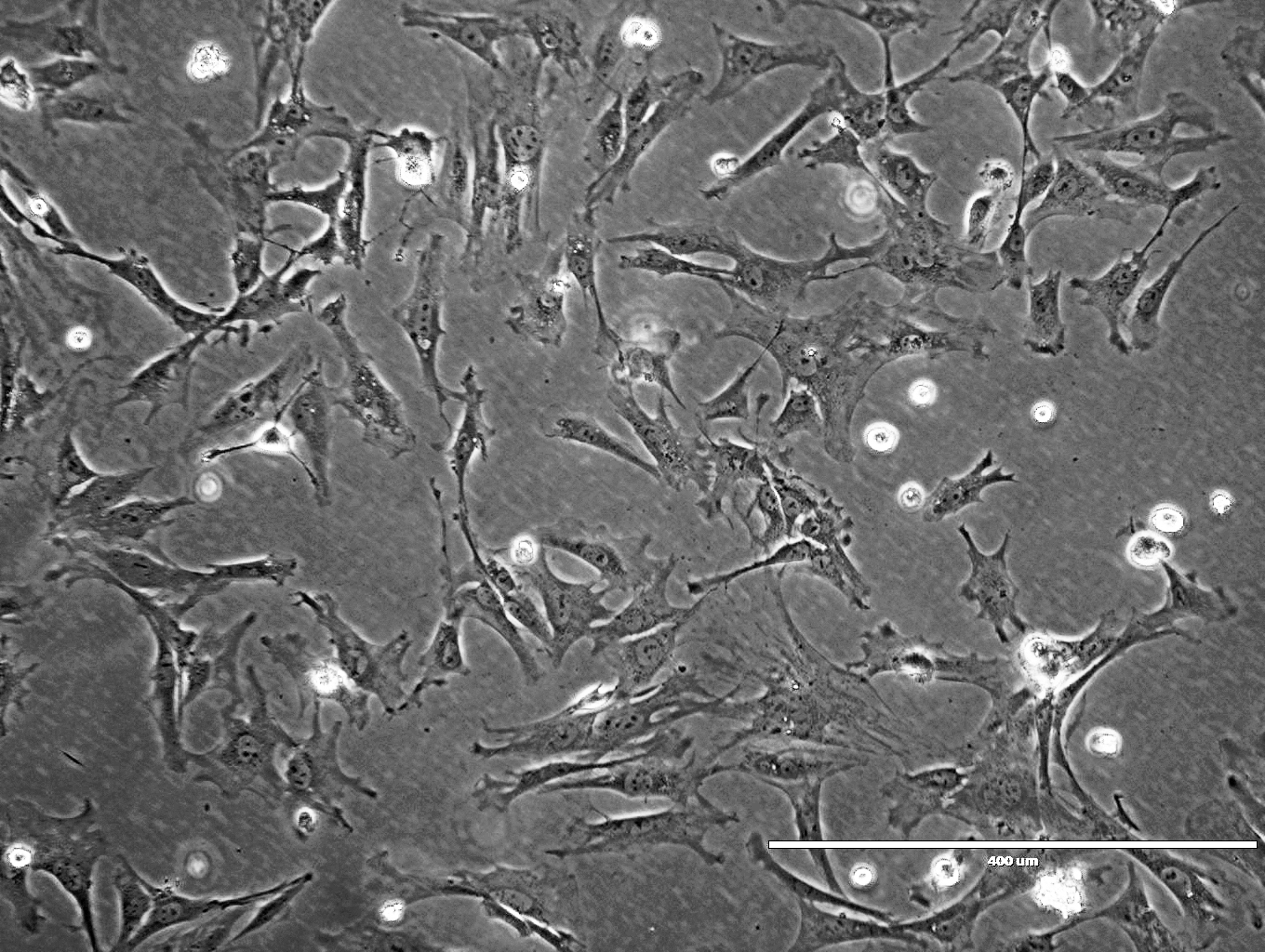

Supplement: Supplementary file 33 — Source data EV and Appendix [file 44318_2025_540_MOESM33_ESM.zip › Source data EV and Appendix/Figure EV 2/2A/6h.jpg]

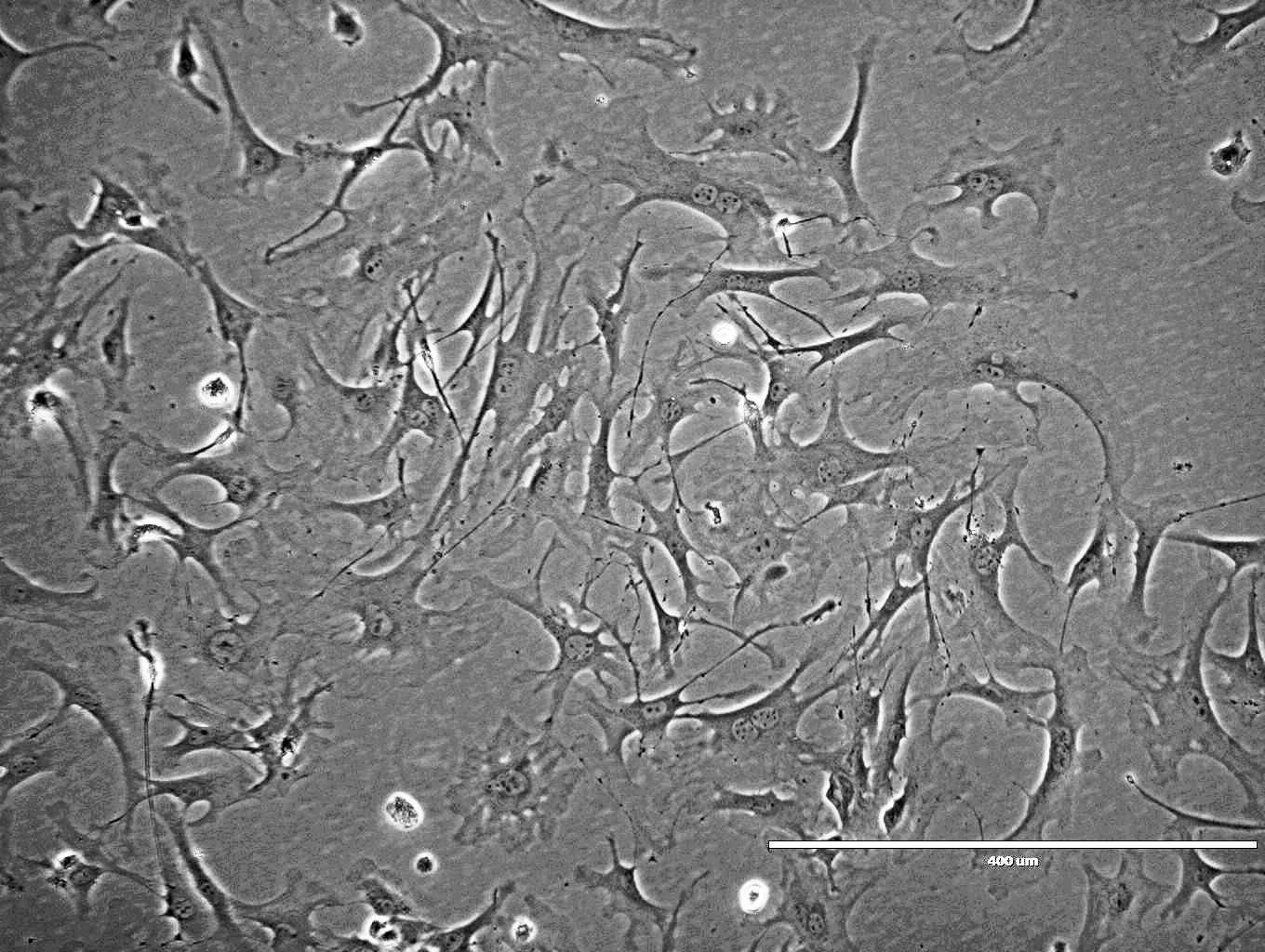

Supplement: Supplementary file 33 — Source data EV and Appendix [file 44318_2025_540_MOESM33_ESM.zip › Source data EV and Appendix/Figure EV 2/2B/0h.jpg]

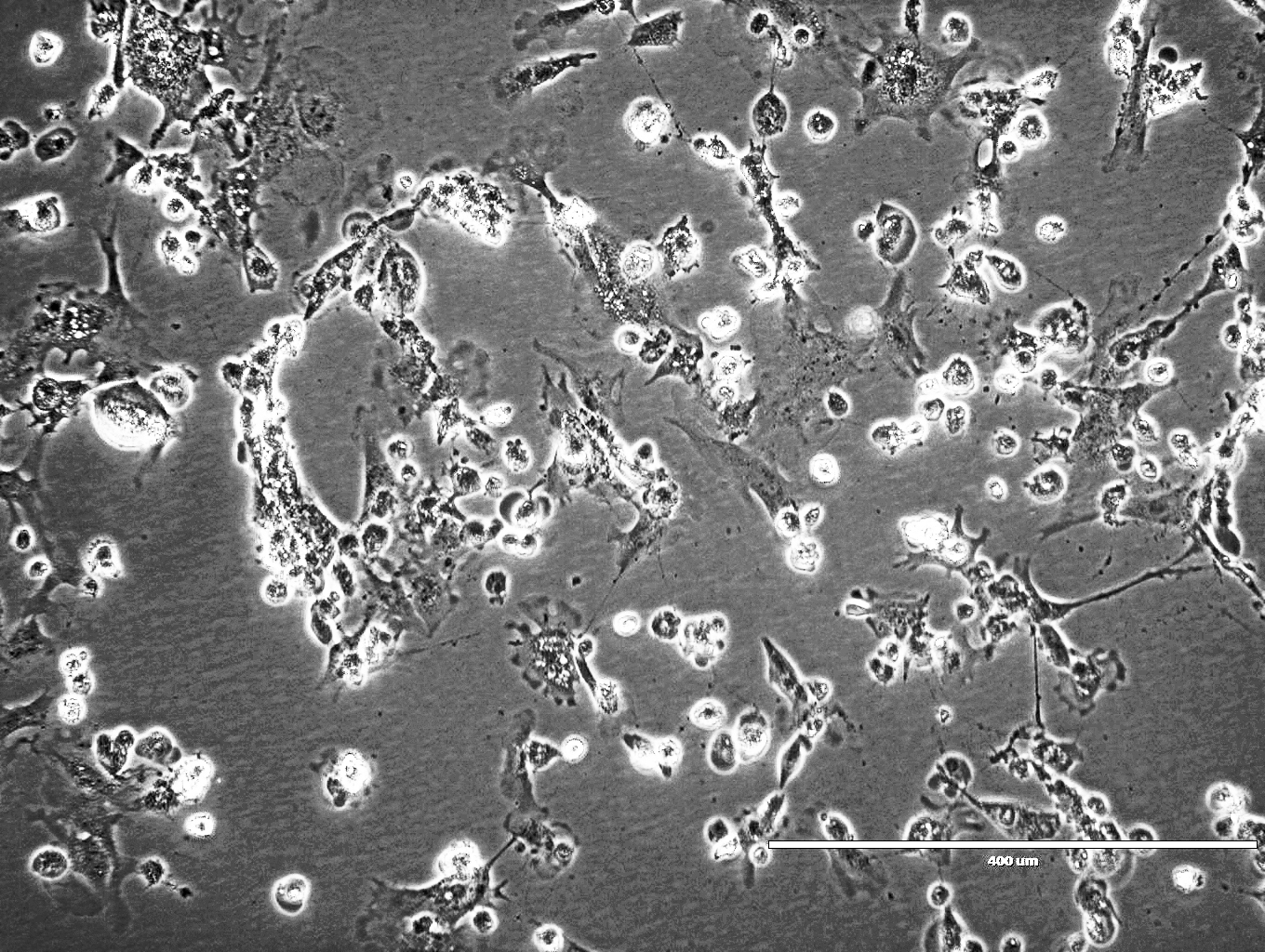

Supplement: Supplementary file 33 — Source data EV and Appendix [file 44318_2025_540_MOESM33_ESM.zip › Source data EV and Appendix/Figure EV 2/2B/1h.jpg]

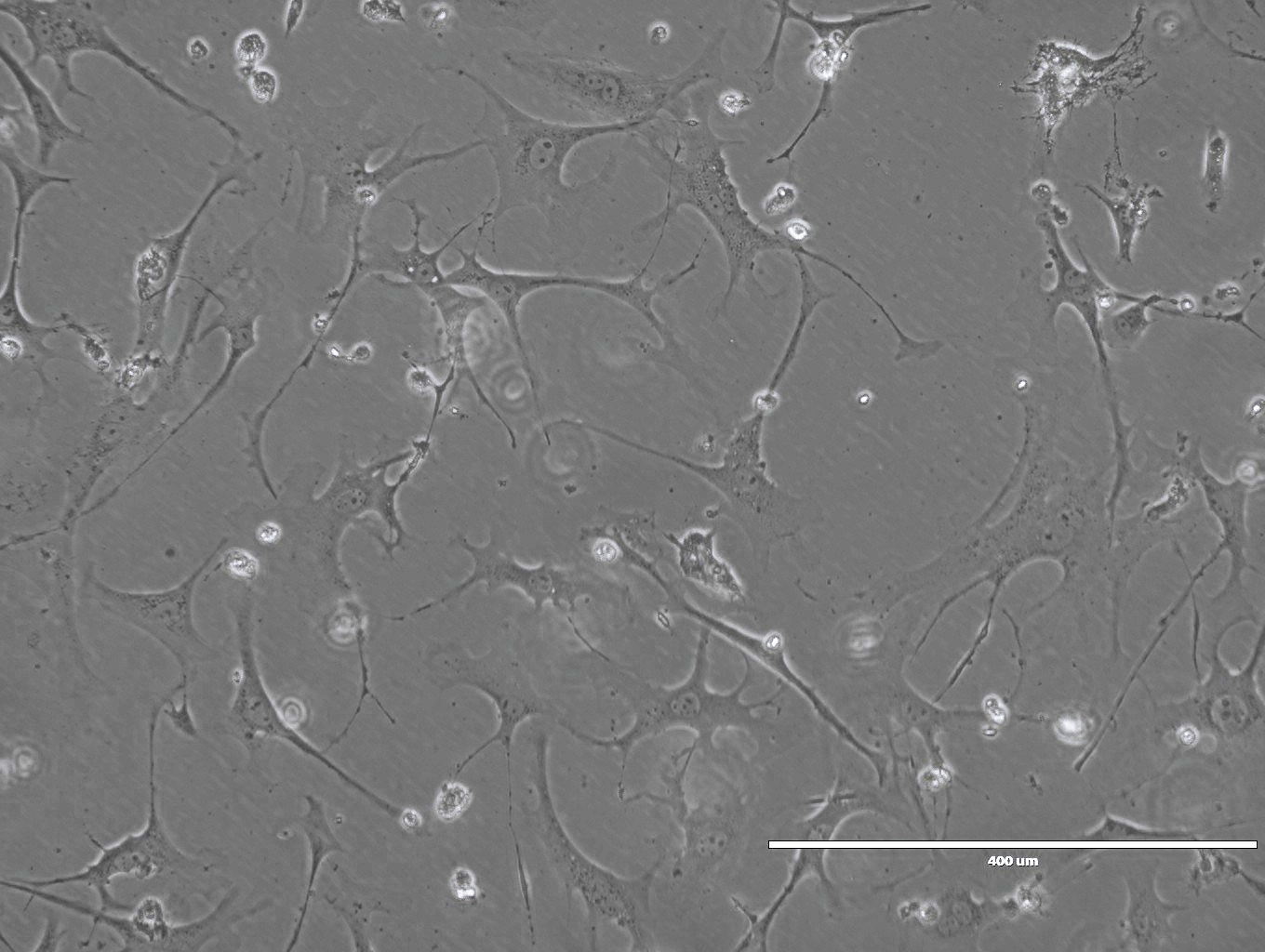

Supplement: Supplementary file 33 — Source data EV and Appendix [file 44318_2025_540_MOESM33_ESM.zip › Source data EV and Appendix/Figure EV 2/2B/24h.jpg]

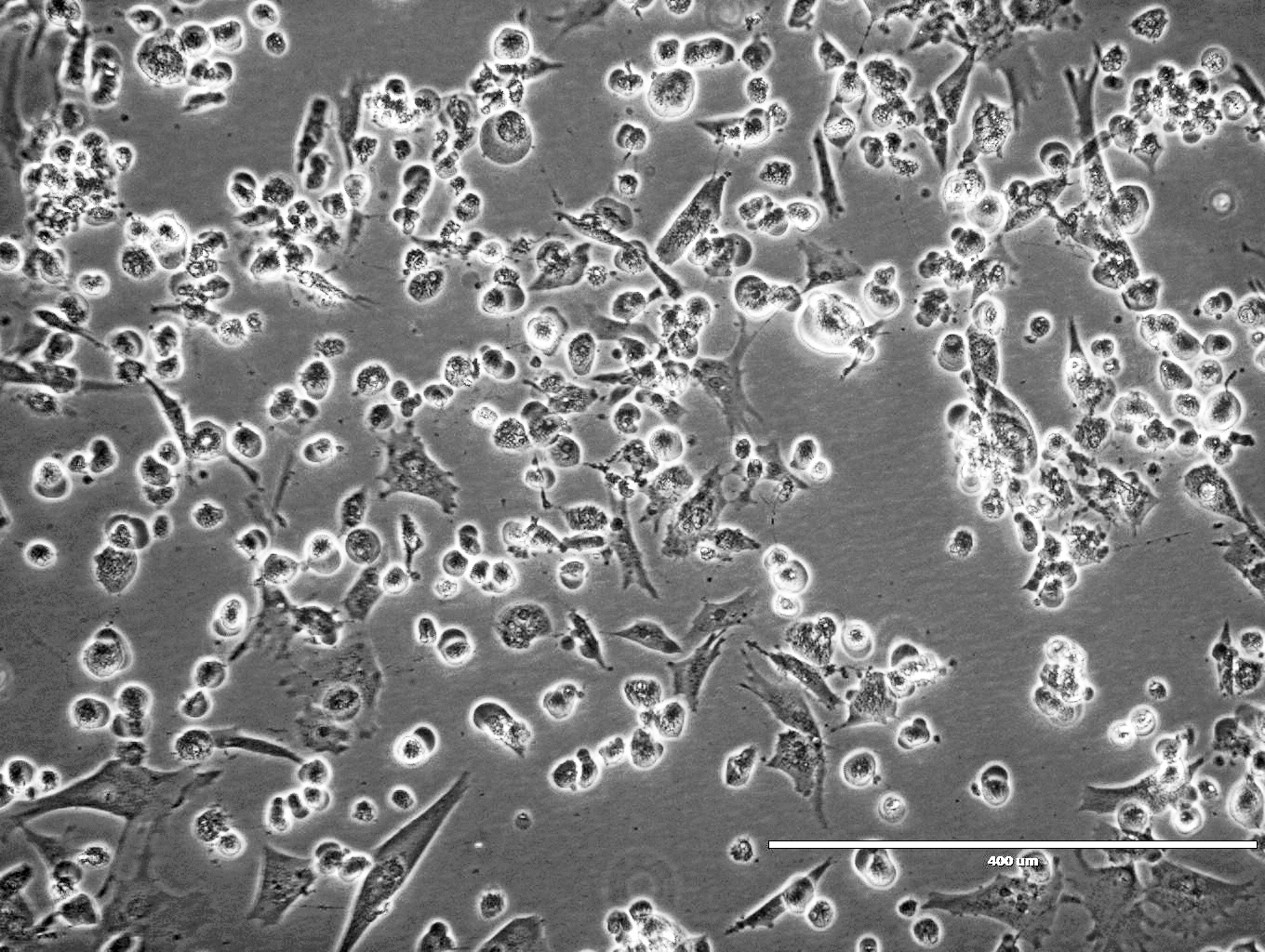

Supplement: Supplementary file 33 — Source data EV and Appendix [file 44318_2025_540_MOESM33_ESM.zip › Source data EV and Appendix/Figure EV 2/2B/30 min.jpg]

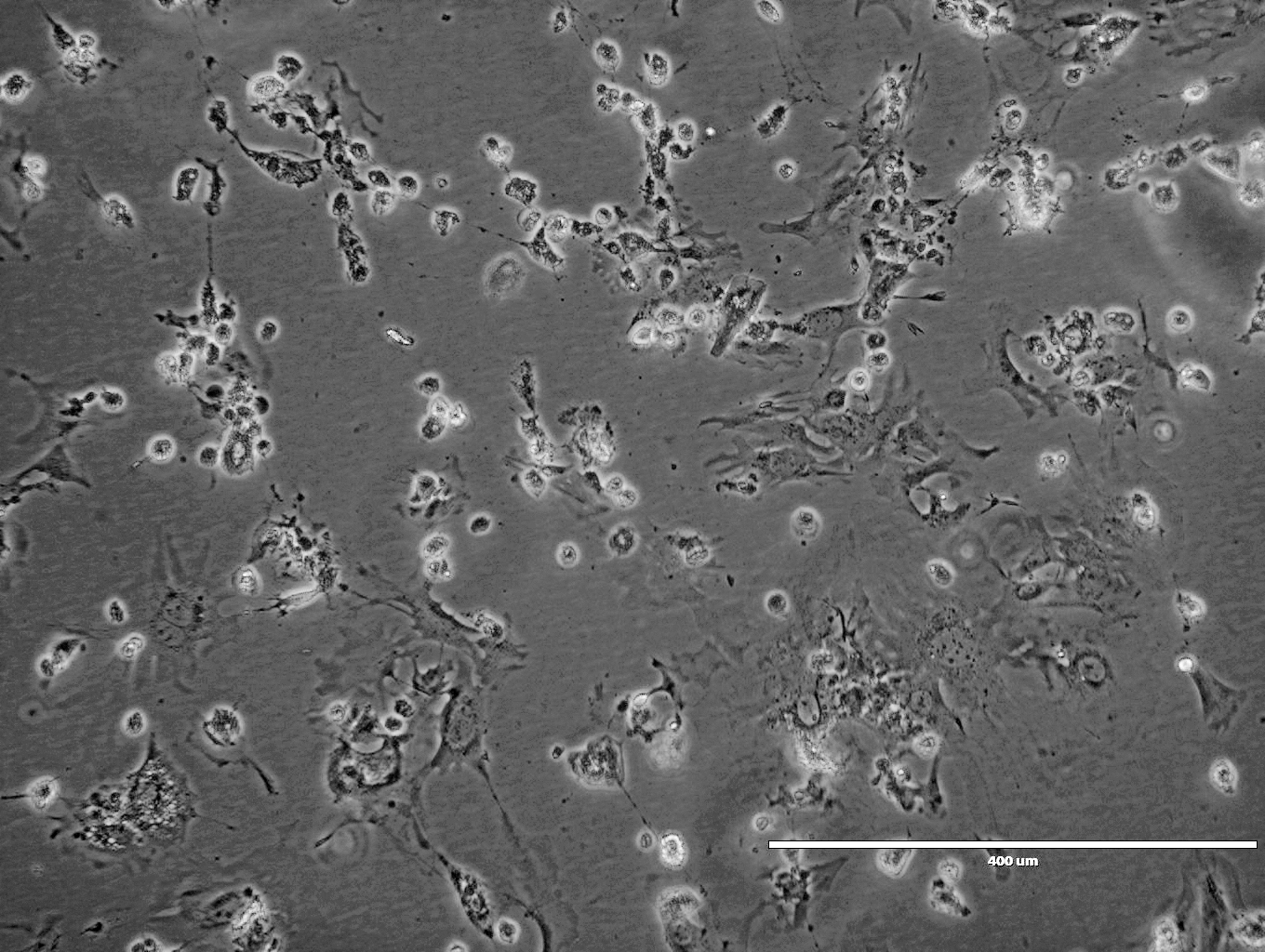

Supplement: Supplementary file 33 — Source data EV and Appendix [file 44318_2025_540_MOESM33_ESM.zip › Source data EV and Appendix/Figure EV 2/2B/6h.jpg]

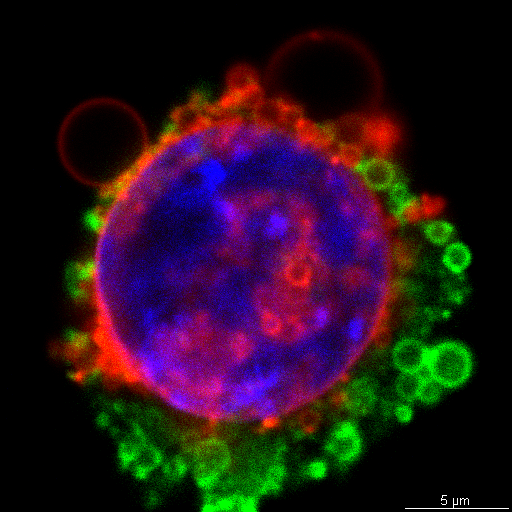

Supplement: Supplementary file 33 — Source data EV and Appendix [file 44318_2025_540_MOESM33_ESM.zip › Source data EV and Appendix/Figure EV 2/2D/12mM/Annexin FITC+DIL+DAPI_12mM.tif]

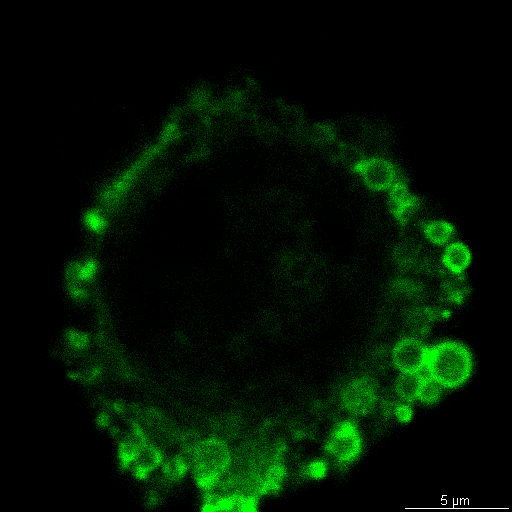

Supplement: Supplementary file 33 — Source data EV and Appendix [file 44318_2025_540_MOESM33_ESM.zip › Source data EV and Appendix/Figure EV 2/2D/12mM/Annexin FITC_12mM.tif]

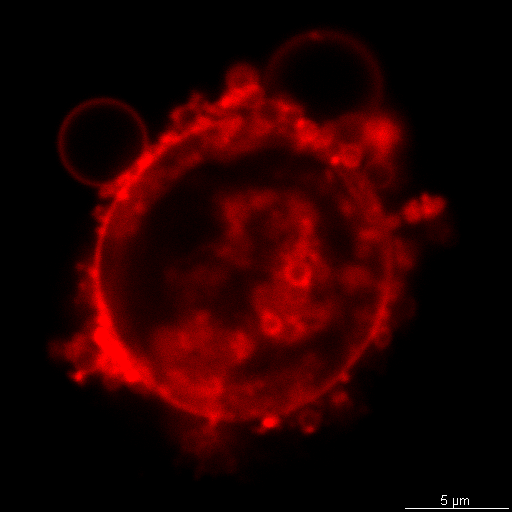

Supplement: Supplementary file 33 — Source data EV and Appendix [file 44318_2025_540_MOESM33_ESM.zip › Source data EV and Appendix/Figure EV 2/2D/12mM/DIL_12mM.tif]

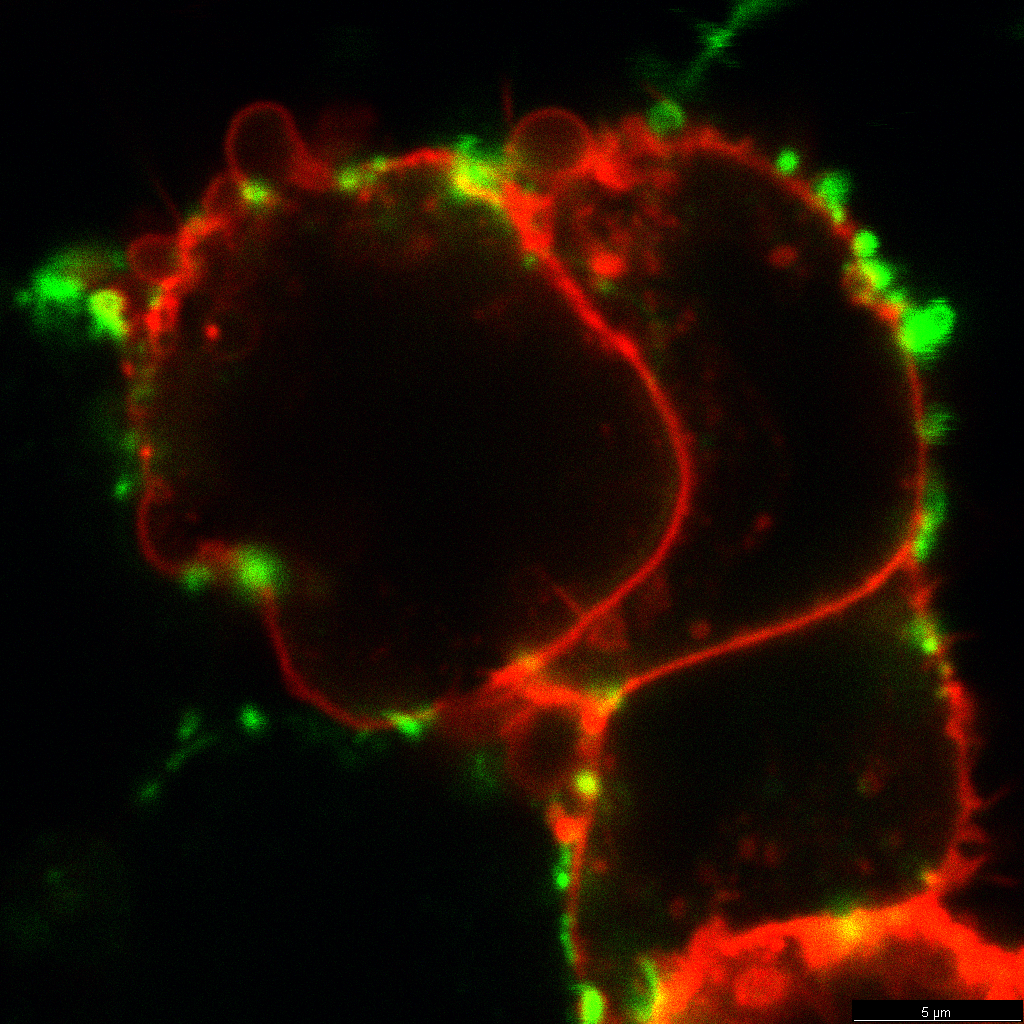

Supplement: Supplementary file 33 — Source data EV and Appendix [file 44318_2025_540_MOESM33_ESM.zip › Source data EV and Appendix/Figure EV 2/2D/4mM/Annexin FITC+DIL+DAPI_4mM.tif]

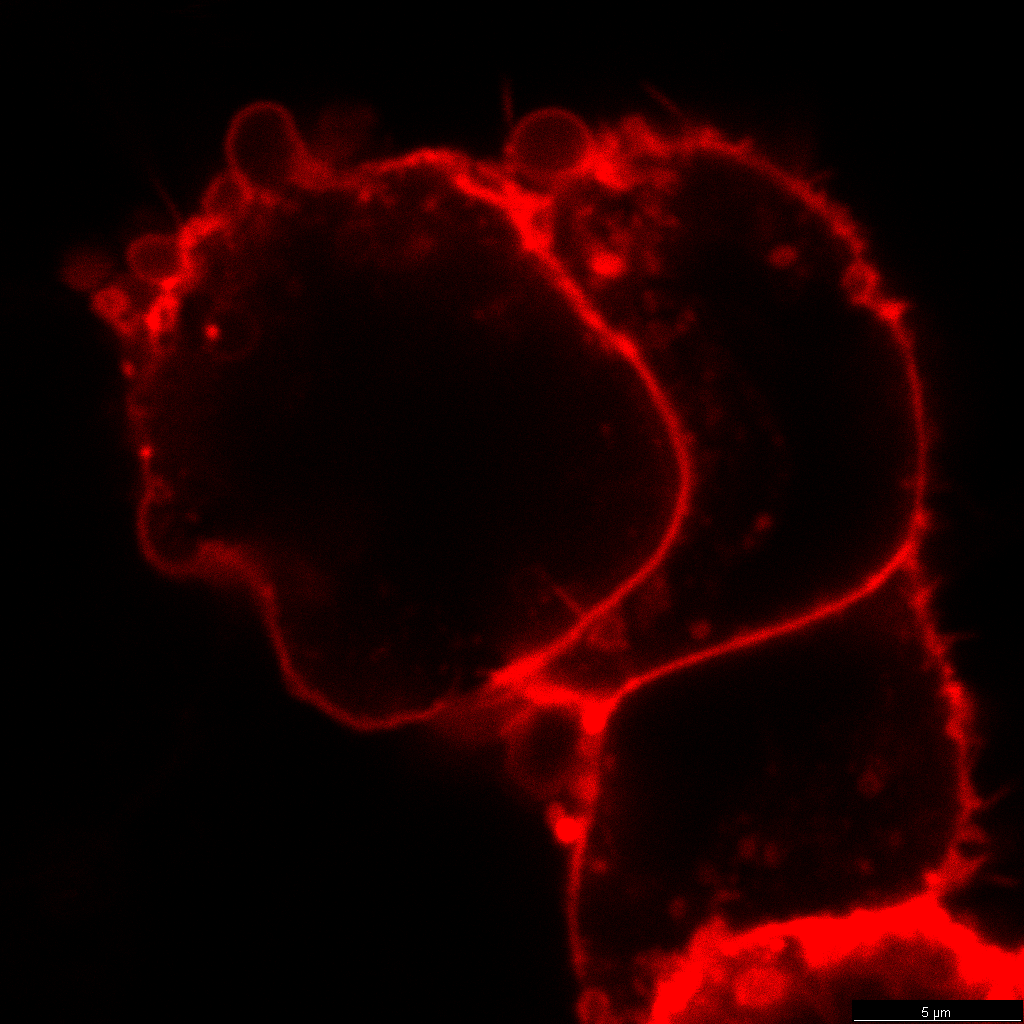

Supplement: Supplementary file 33 — Source data EV and Appendix [file 44318_2025_540_MOESM33_ESM.zip › Source data EV and Appendix/Figure EV 2/2D/4mM/DIL_4mM.tif]

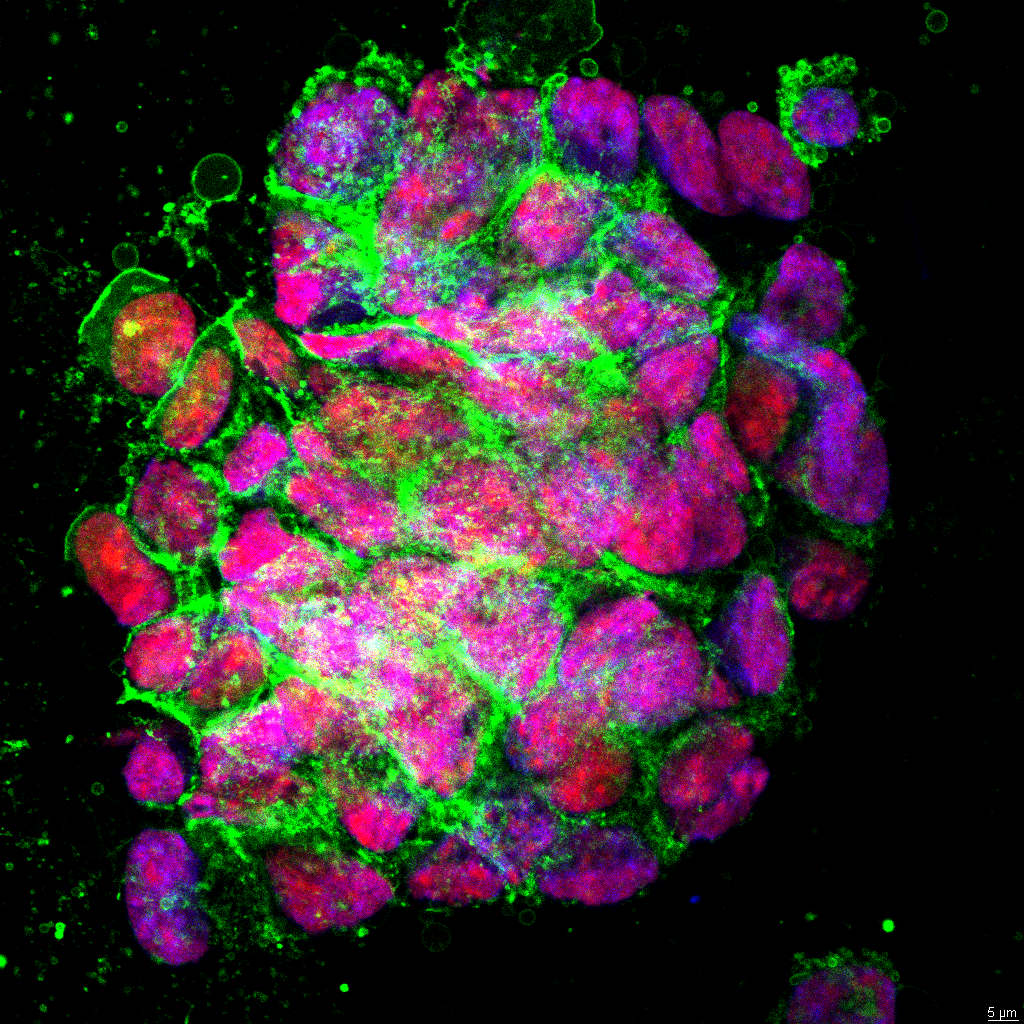

Supplement: Supplementary file 33 — Source data EV and Appendix [file 44318_2025_540_MOESM33_ESM.zip › Source data EV and Appendix/Figure EV 2/2E/12mM/Annexin FITC+EtBr+Hoechst_12mM.tif]

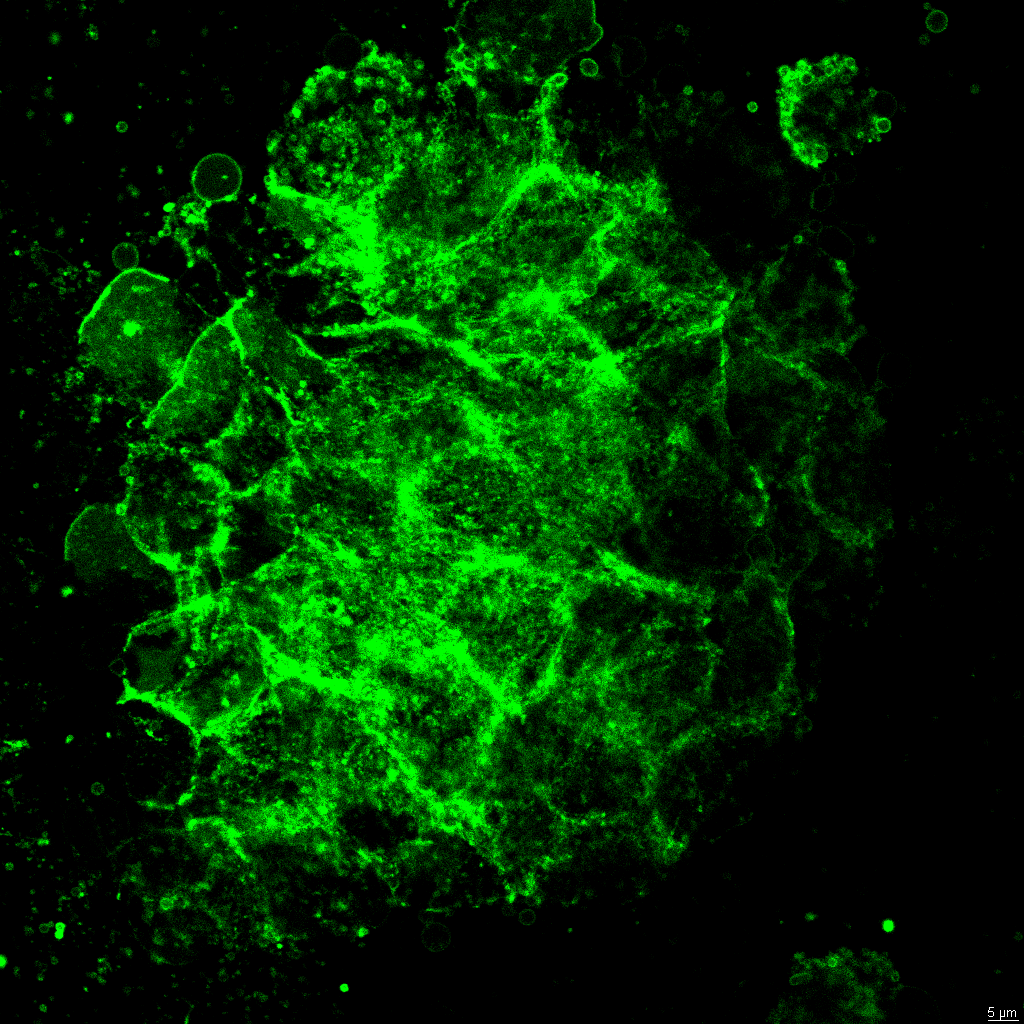

Supplement: Supplementary file 33 — Source data EV and Appendix [file 44318_2025_540_MOESM33_ESM.zip › Source data EV and Appendix/Figure EV 2/2E/12mM/Annexin FITC_12mM.tif]

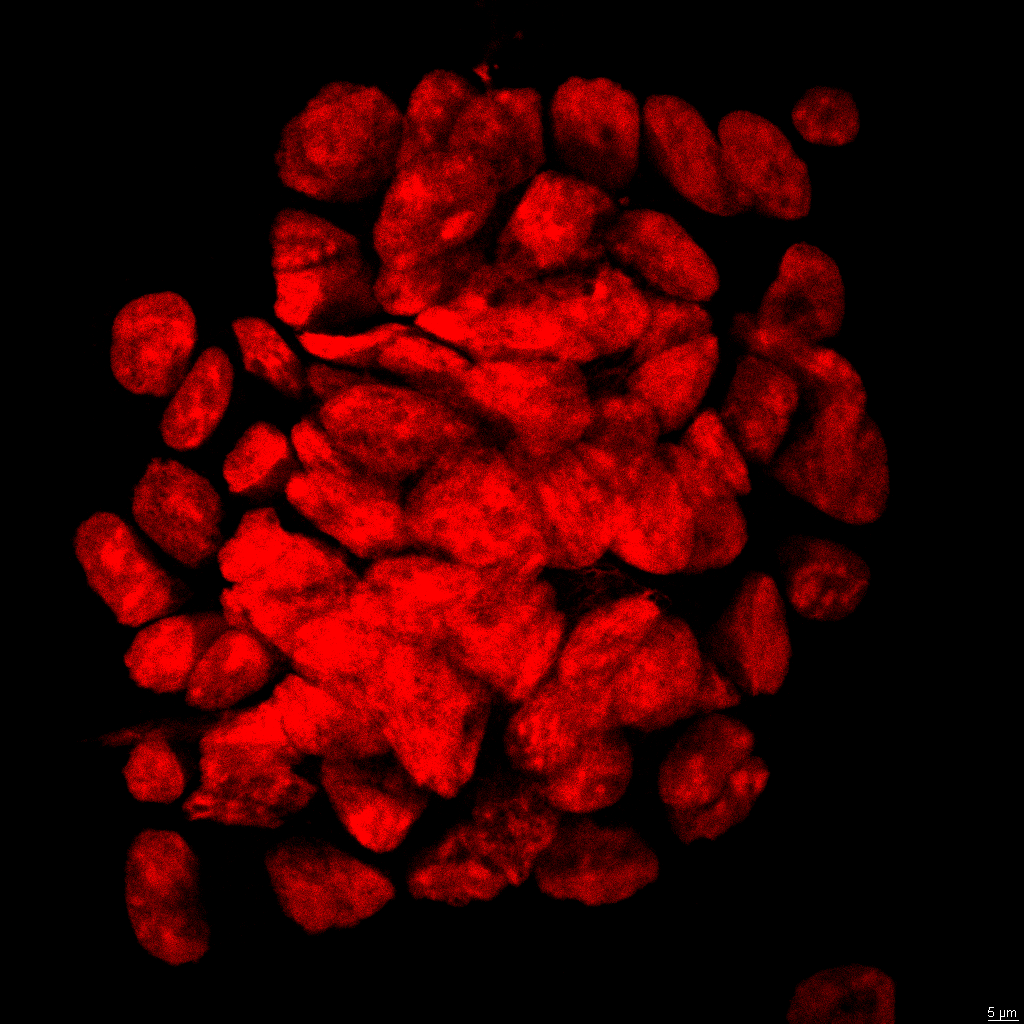

Supplement: Supplementary file 33 — Source data EV and Appendix [file 44318_2025_540_MOESM33_ESM.zip › Source data EV and Appendix/Figure EV 2/2E/12mM/EtBr_12mM.tif]

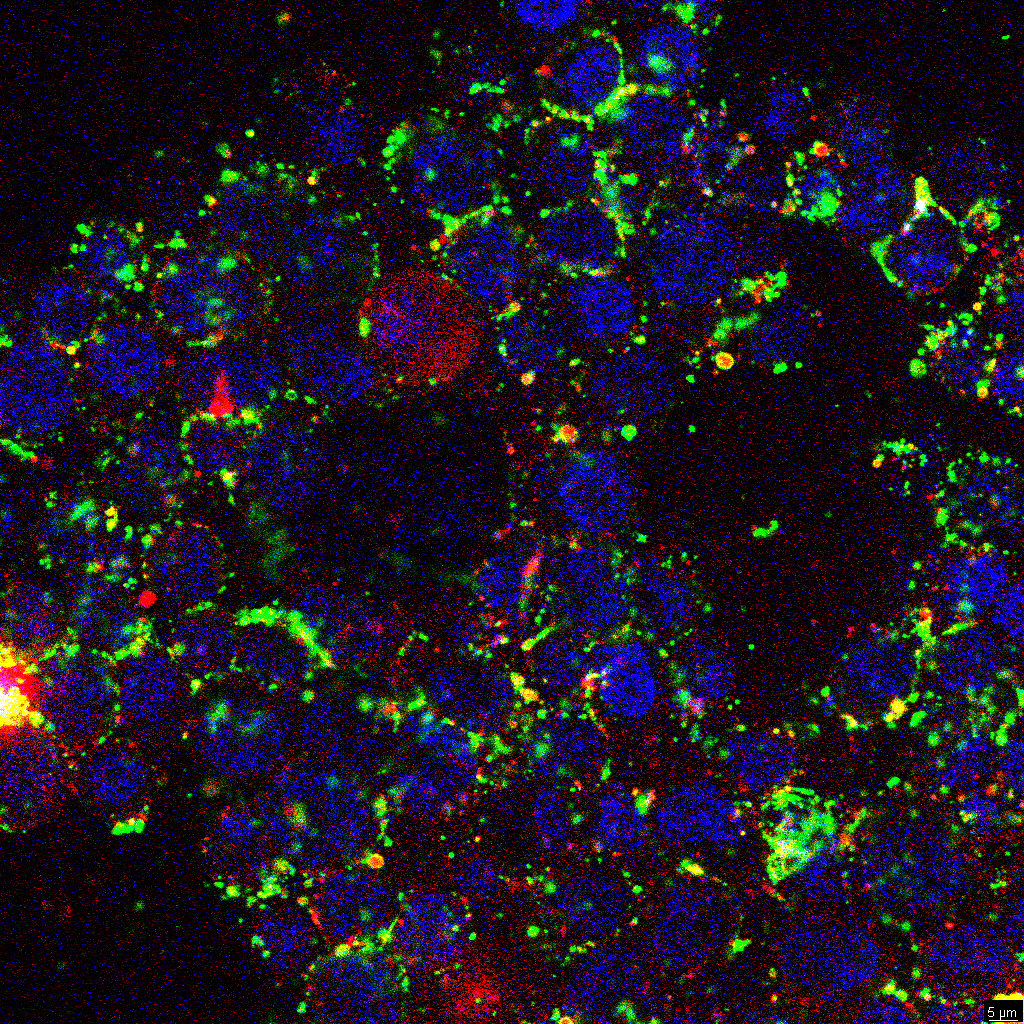

Supplement: Supplementary file 33 — Source data EV and Appendix [file 44318_2025_540_MOESM33_ESM.zip › Source data EV and Appendix/Figure EV 2/2E/4mM/Annexin FITC+EtBr+Hoechst_4mM.tif]

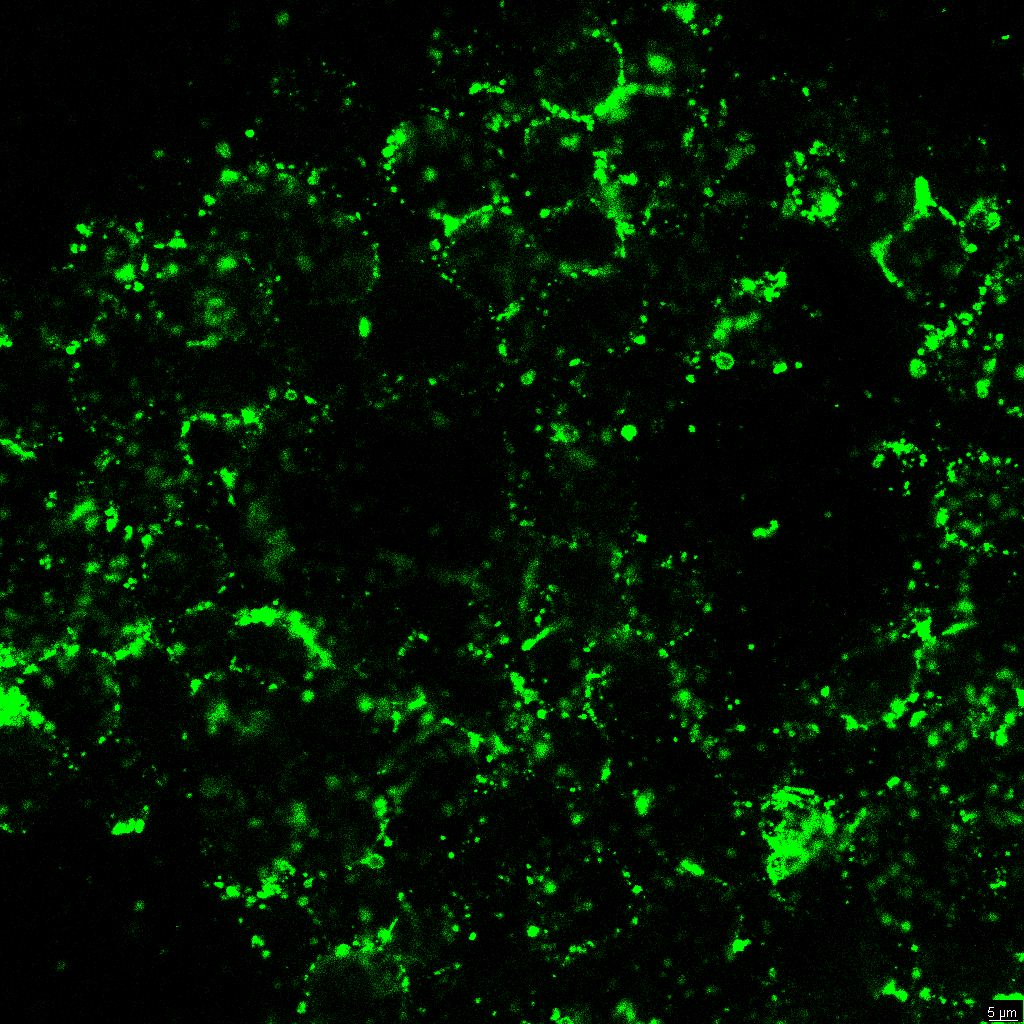

Supplement: Supplementary file 33 — Source data EV and Appendix [file 44318_2025_540_MOESM33_ESM.zip › Source data EV and Appendix/Figure EV 2/2E/4mM/Annexin FITC_4mM.tif]

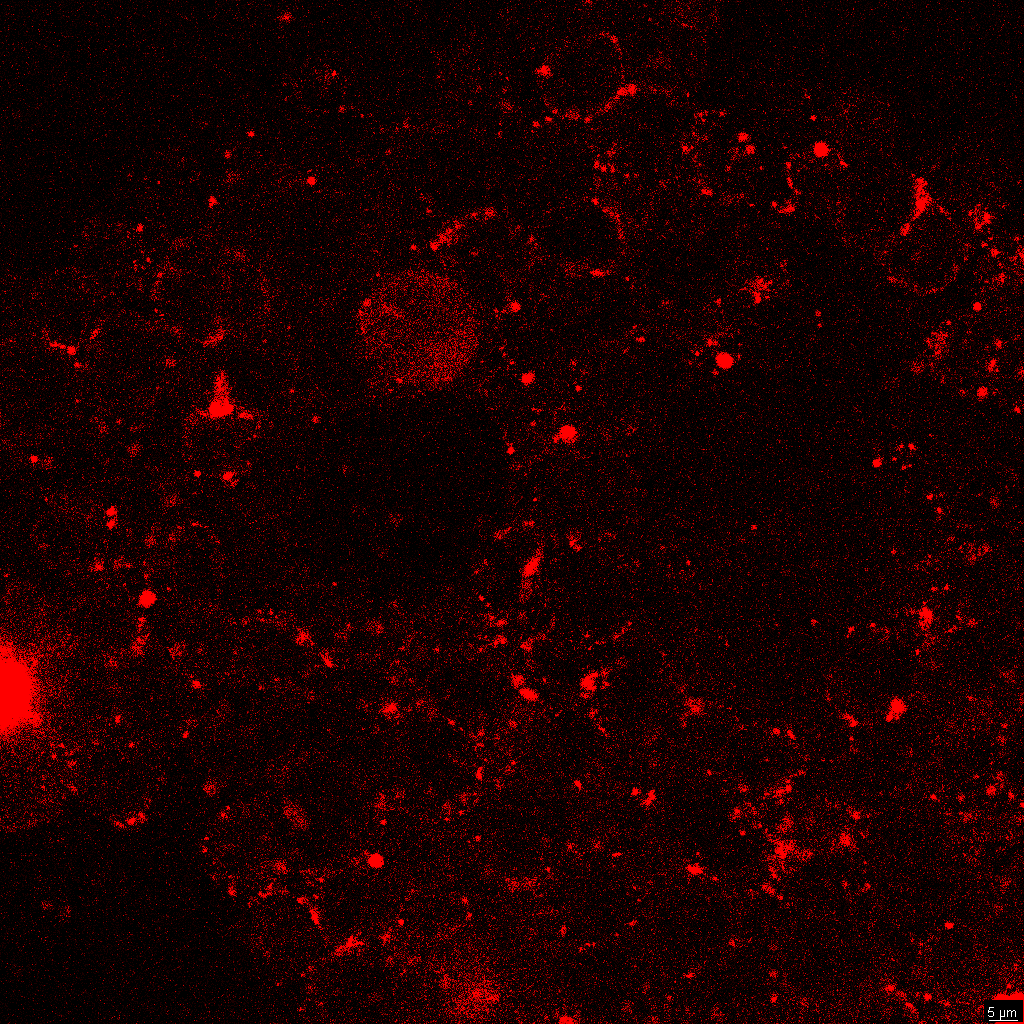

Supplement: Supplementary file 33 — Source data EV and Appendix [file 44318_2025_540_MOESM33_ESM.zip › Source data EV and Appendix/Figure EV 2/2E/4mM/EtBr_4mM.tif]

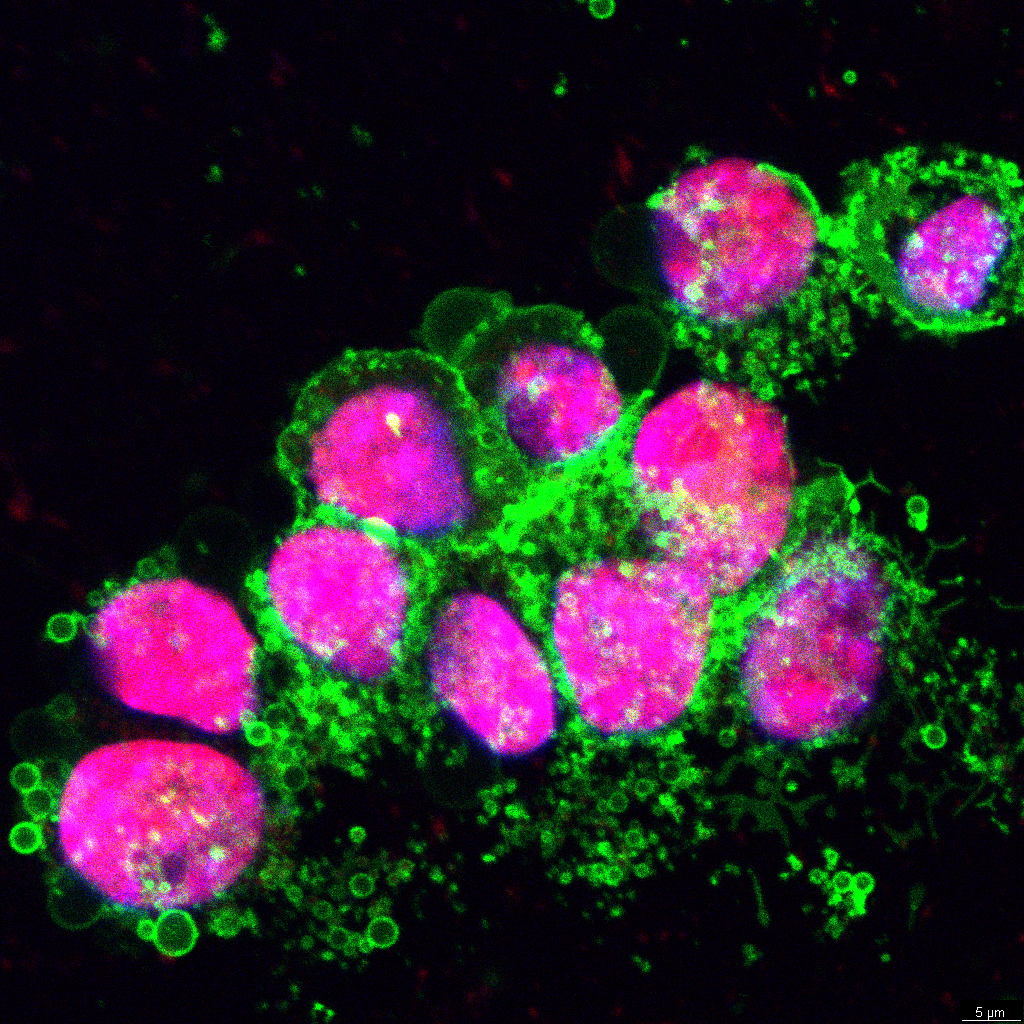

Supplement: Supplementary file 33 — Source data EV and Appendix [file 44318_2025_540_MOESM33_ESM.zip › Source data EV and Appendix/Figure EV 2/2G/12mM/Annexin FITC+PI+Hoechst_12mM.tif]

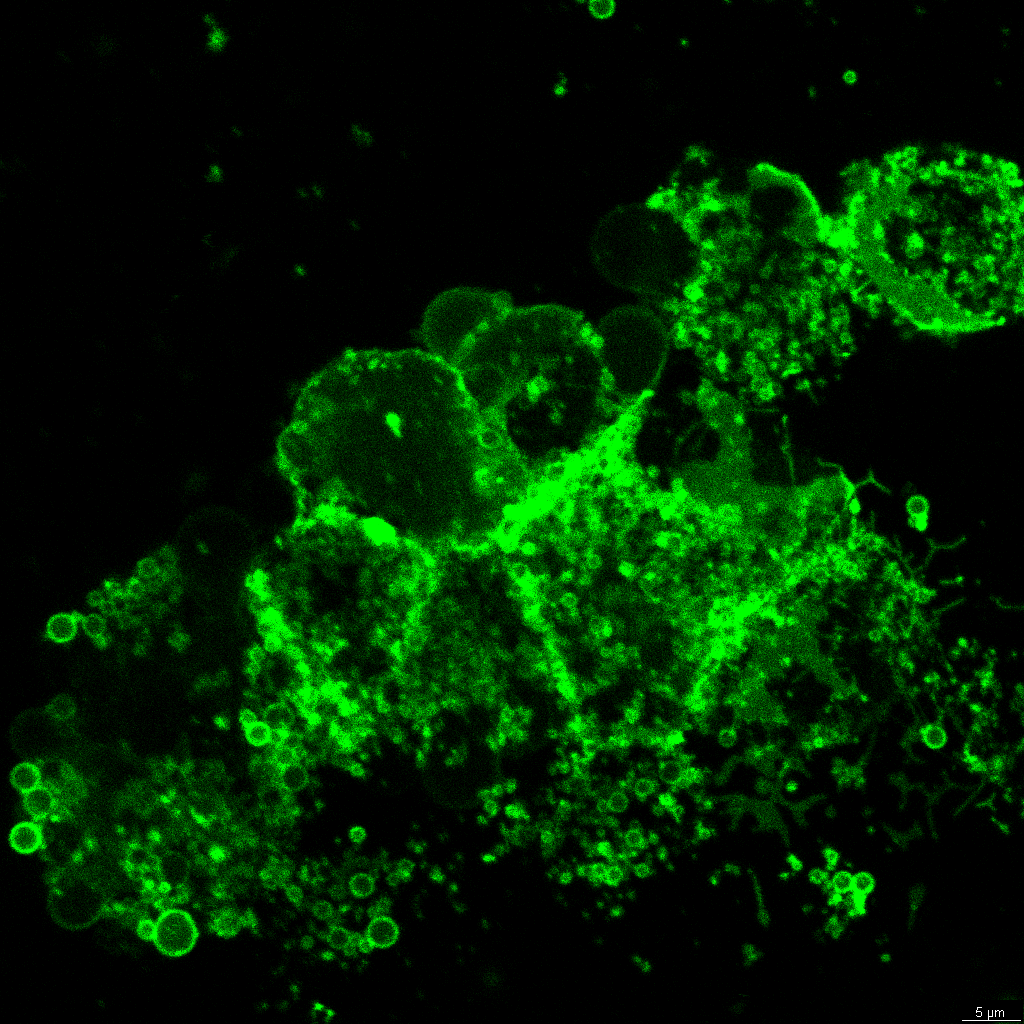

Supplement: Supplementary file 33 — Source data EV and Appendix [file 44318_2025_540_MOESM33_ESM.zip › Source data EV and Appendix/Figure EV 2/2G/12mM/Annexin FITC_12mM.tif]

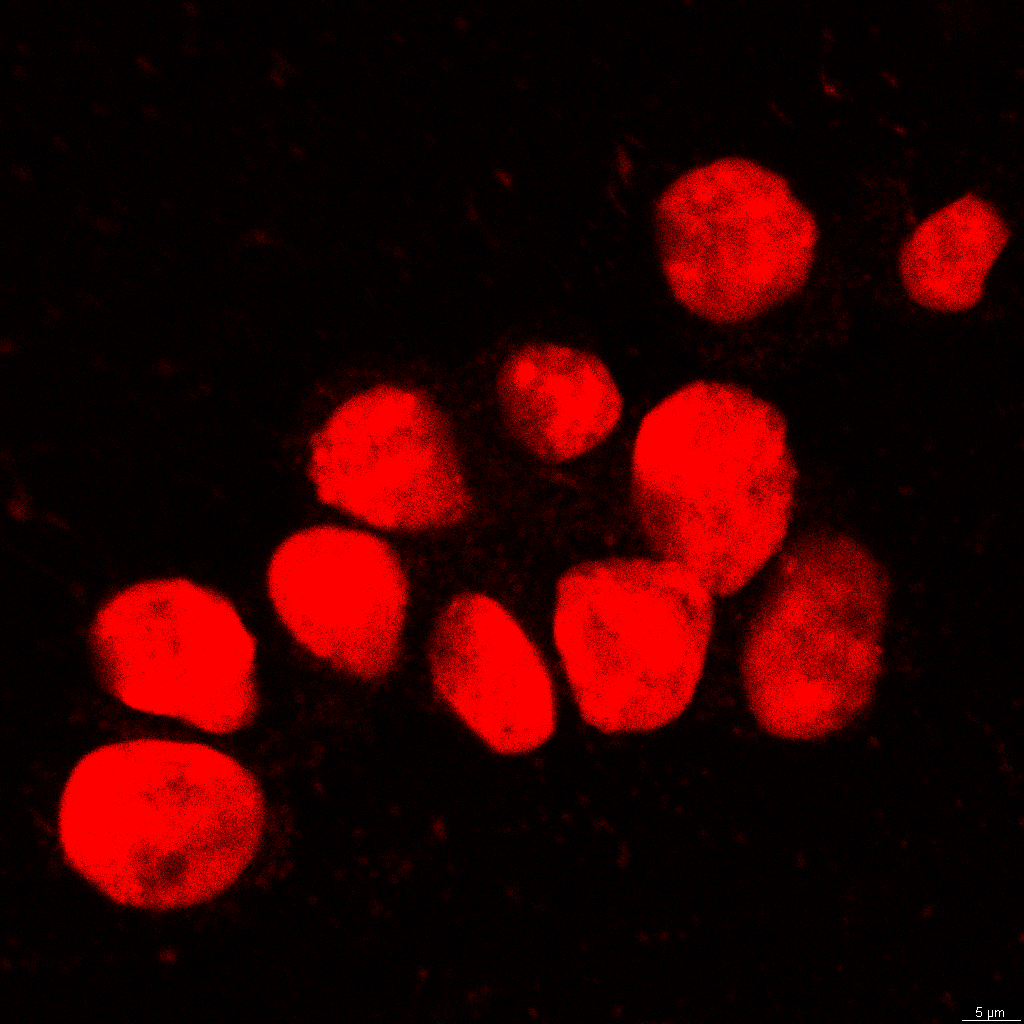

Supplement: Supplementary file 33 — Source data EV and Appendix [file 44318_2025_540_MOESM33_ESM.zip › Source data EV and Appendix/Figure EV 2/2G/12mM/PI_12mM.tif]

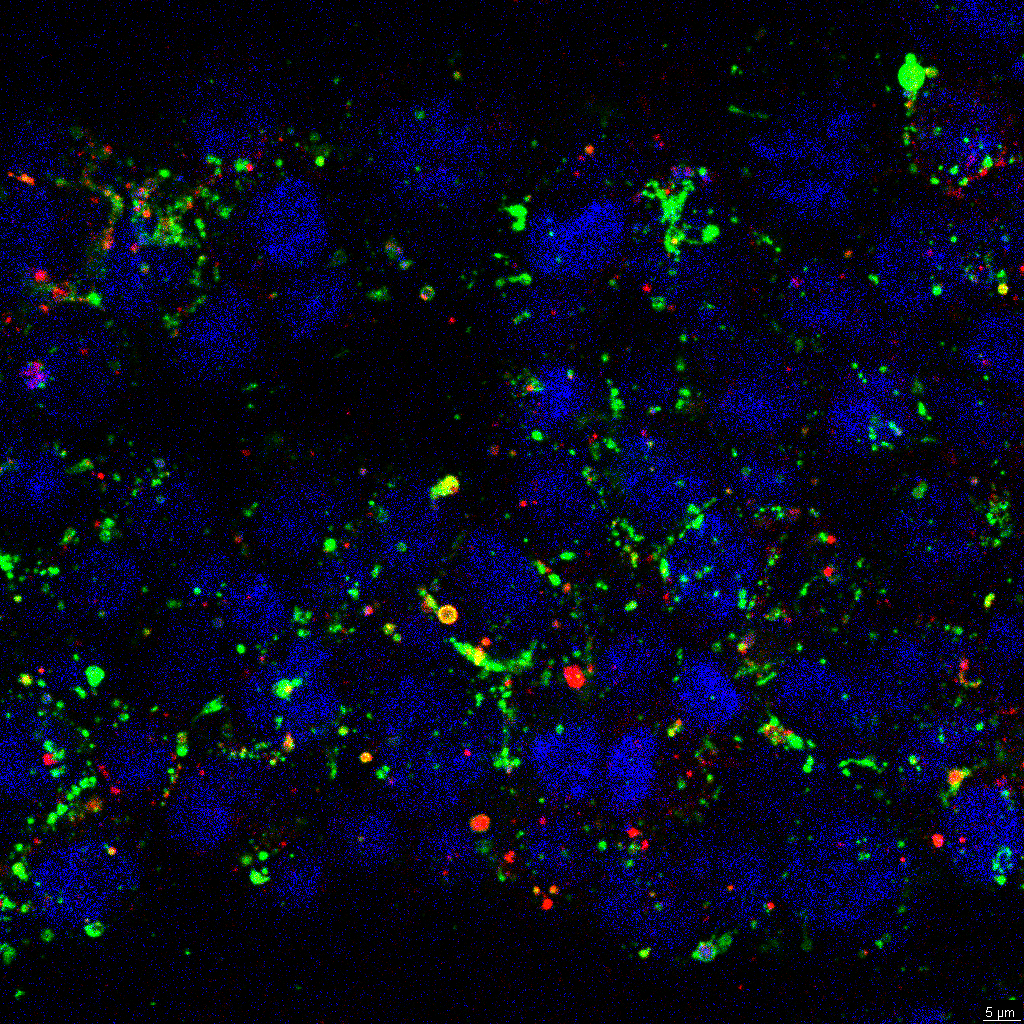

Supplement: Supplementary file 33 — Source data EV and Appendix [file 44318_2025_540_MOESM33_ESM.zip › Source data EV and Appendix/Figure EV 2/2G/4mM/Annexin FITC+PI+Hoechst_4mM.tif]

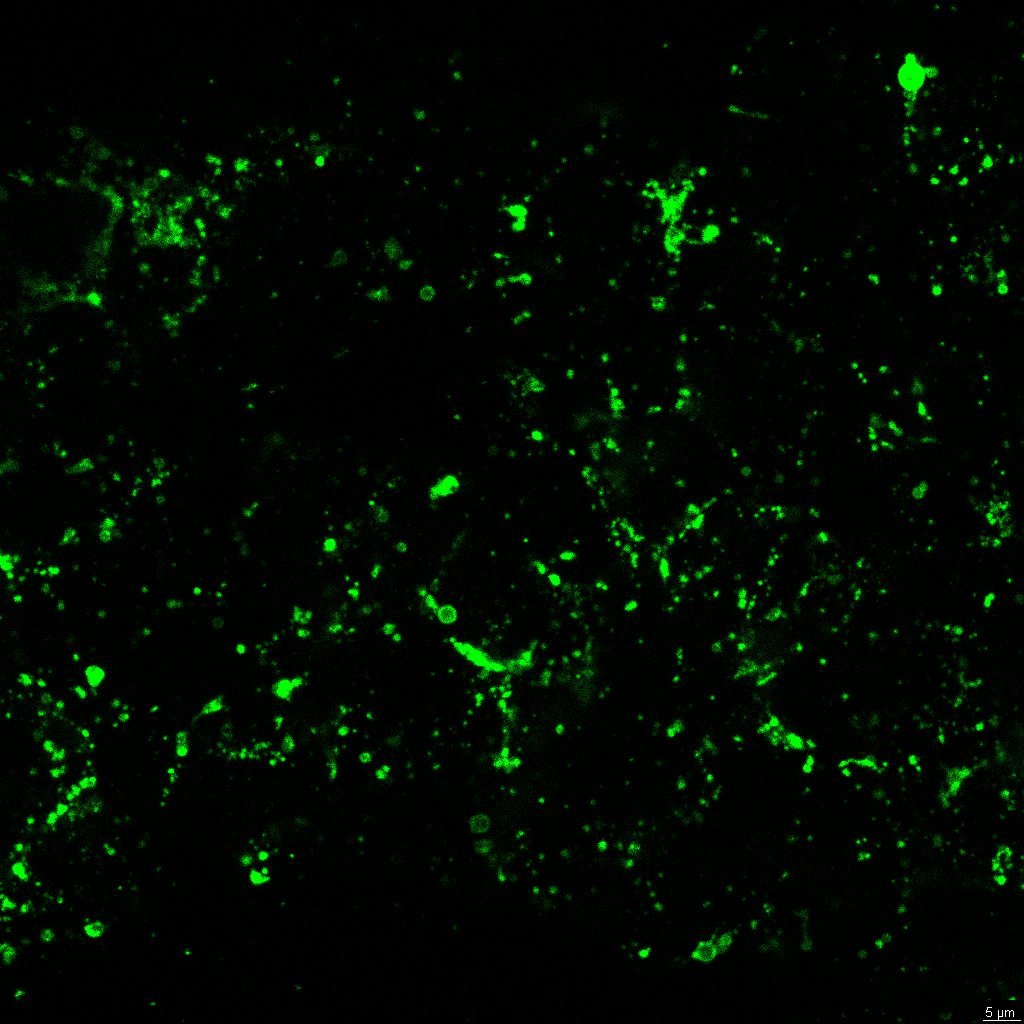

Supplement: Supplementary file 33 — Source data EV and Appendix [file 44318_2025_540_MOESM33_ESM.zip › Source data EV and Appendix/Figure EV 2/2G/4mM/Annexin FITC_4mM.tif]

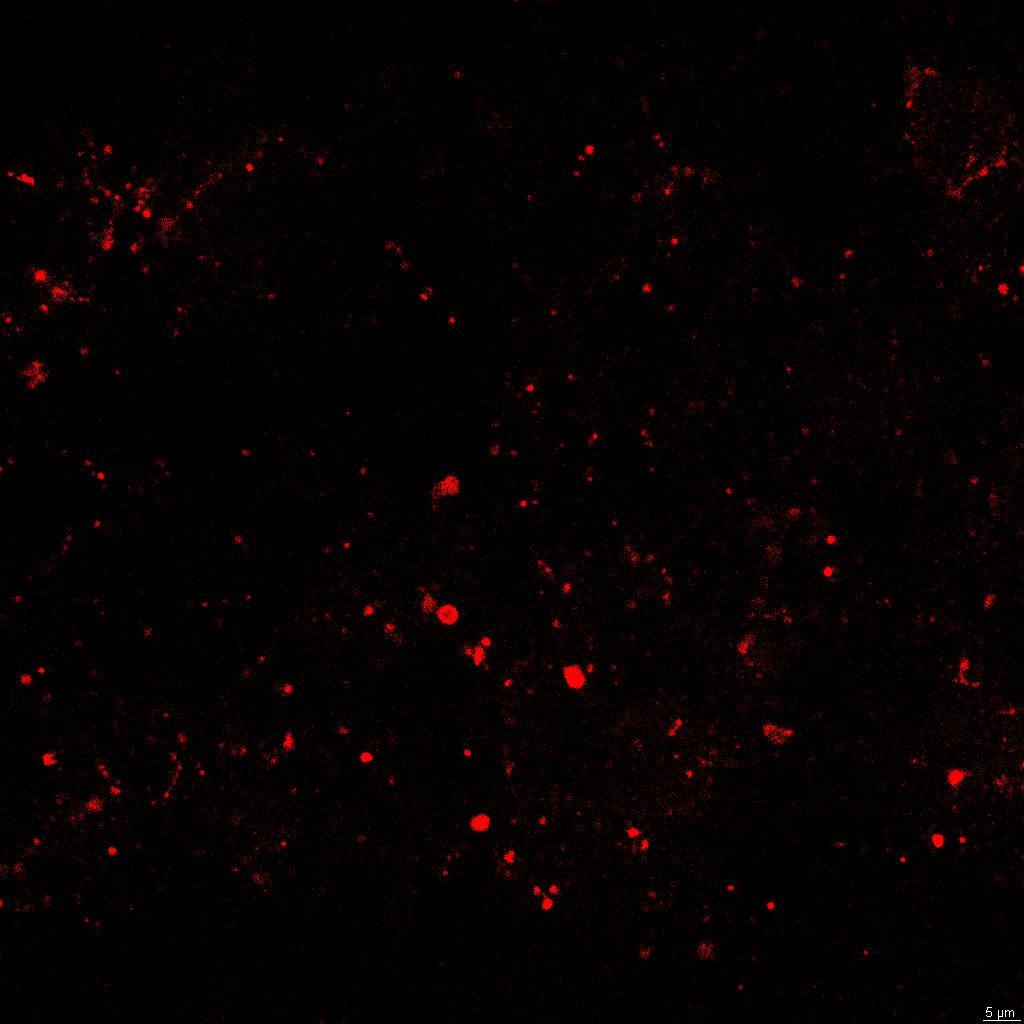

Supplement: Supplementary file 33 — Source data EV and Appendix [file 44318_2025_540_MOESM33_ESM.zip › Source data EV and Appendix/Figure EV 2/2G/4mM/PI_4mM.tif]

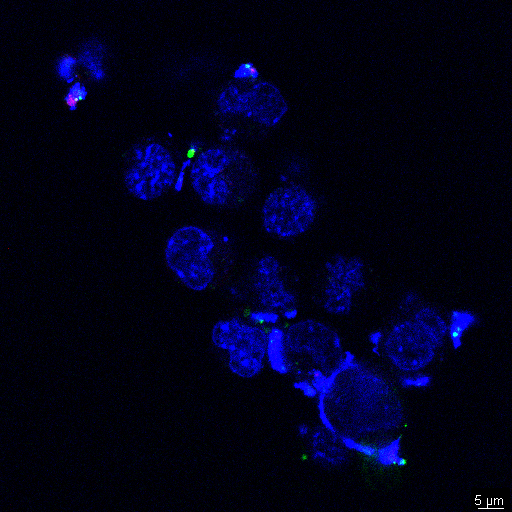

Supplement: Supplementary file 33 — Source data EV and Appendix [file 44318_2025_540_MOESM33_ESM.zip › Source data EV and Appendix/Figure EV 2/2G/Trypsinized Cells/Annexin FITC+PI+Hoechst_Trypsin.tif]

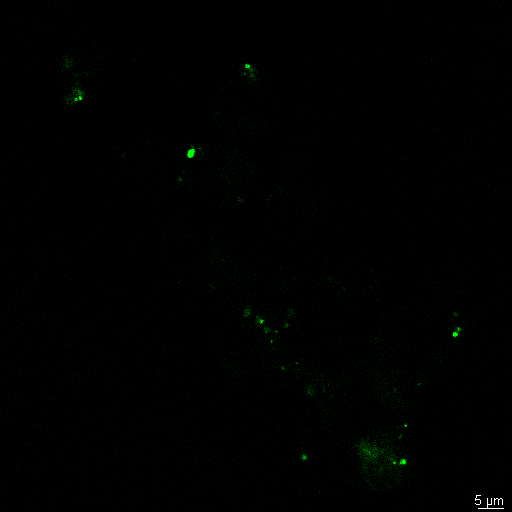

Supplement: Supplementary file 33 — Source data EV and Appendix [file 44318_2025_540_MOESM33_ESM.zip › Source data EV and Appendix/Figure EV 2/2G/Trypsinized Cells/Annexin FITC_Trypsin.tif]

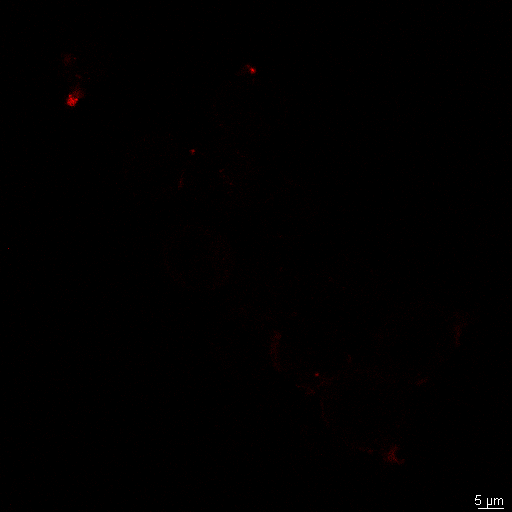

Supplement: Supplementary file 33 — Source data EV and Appendix [file 44318_2025_540_MOESM33_ESM.zip › Source data EV and Appendix/Figure EV 2/2G/Trypsinized Cells/PI_Trypsin.tif]

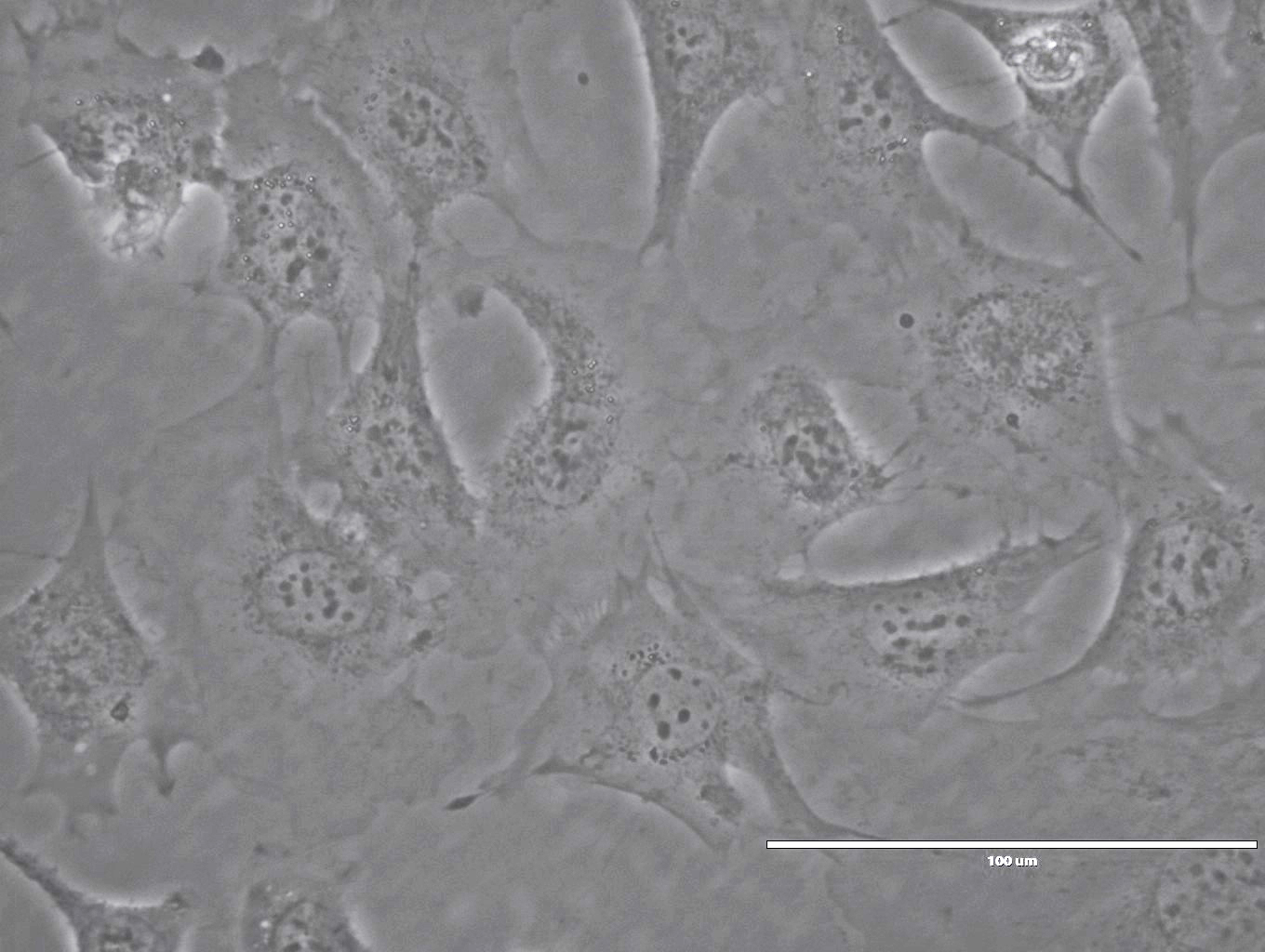

Supplement: Supplementary file 33 — Source data EV and Appendix [file 44318_2025_540_MOESM33_ESM.zip › Source data EV and Appendix/Figure EV 2/2I/12mM/0h.jpg]

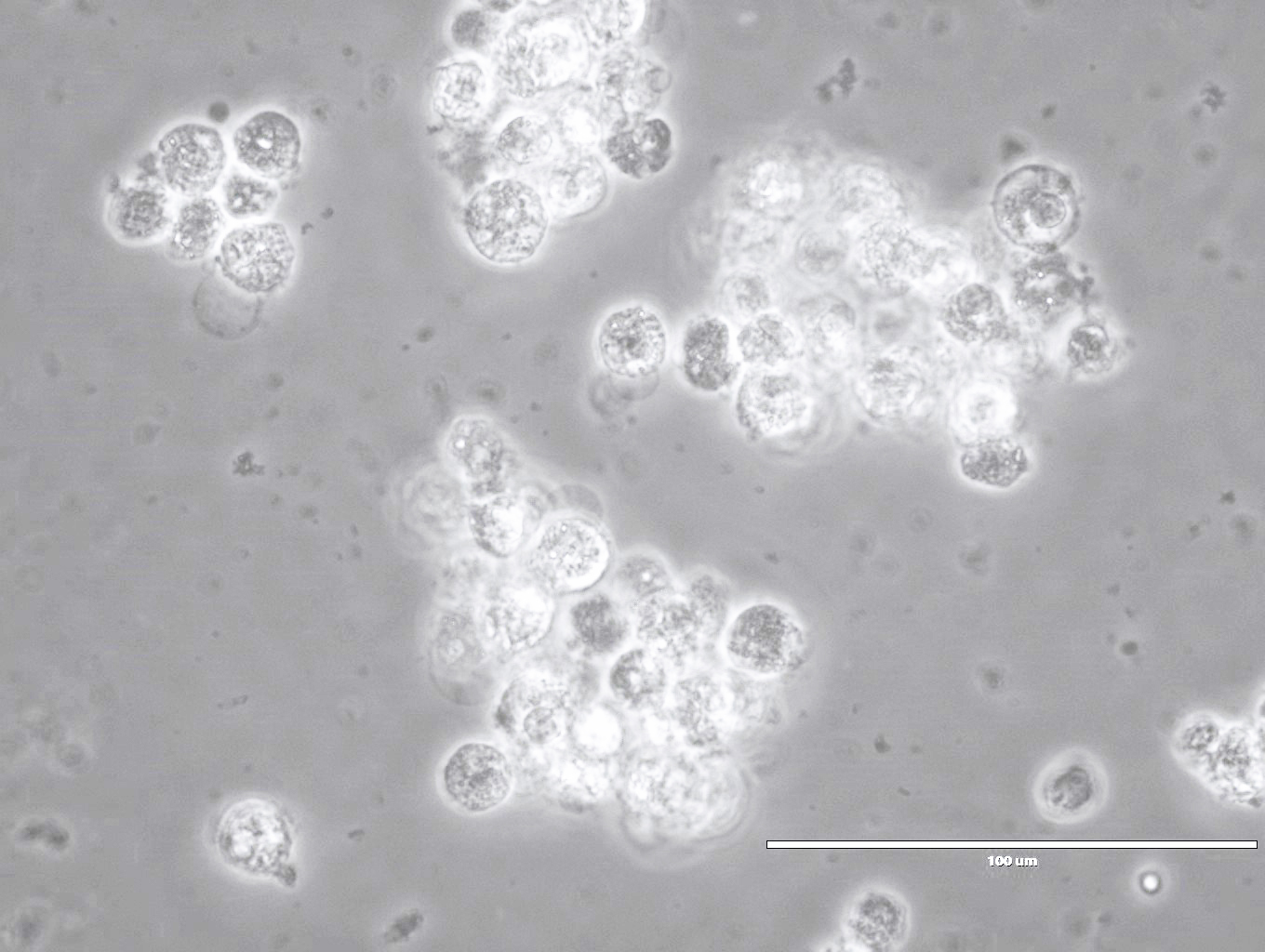

Supplement: Supplementary file 33 — Source data EV and Appendix [file 44318_2025_540_MOESM33_ESM.zip › Source data EV and Appendix/Figure EV 2/2I/12mM/24h.jpg]

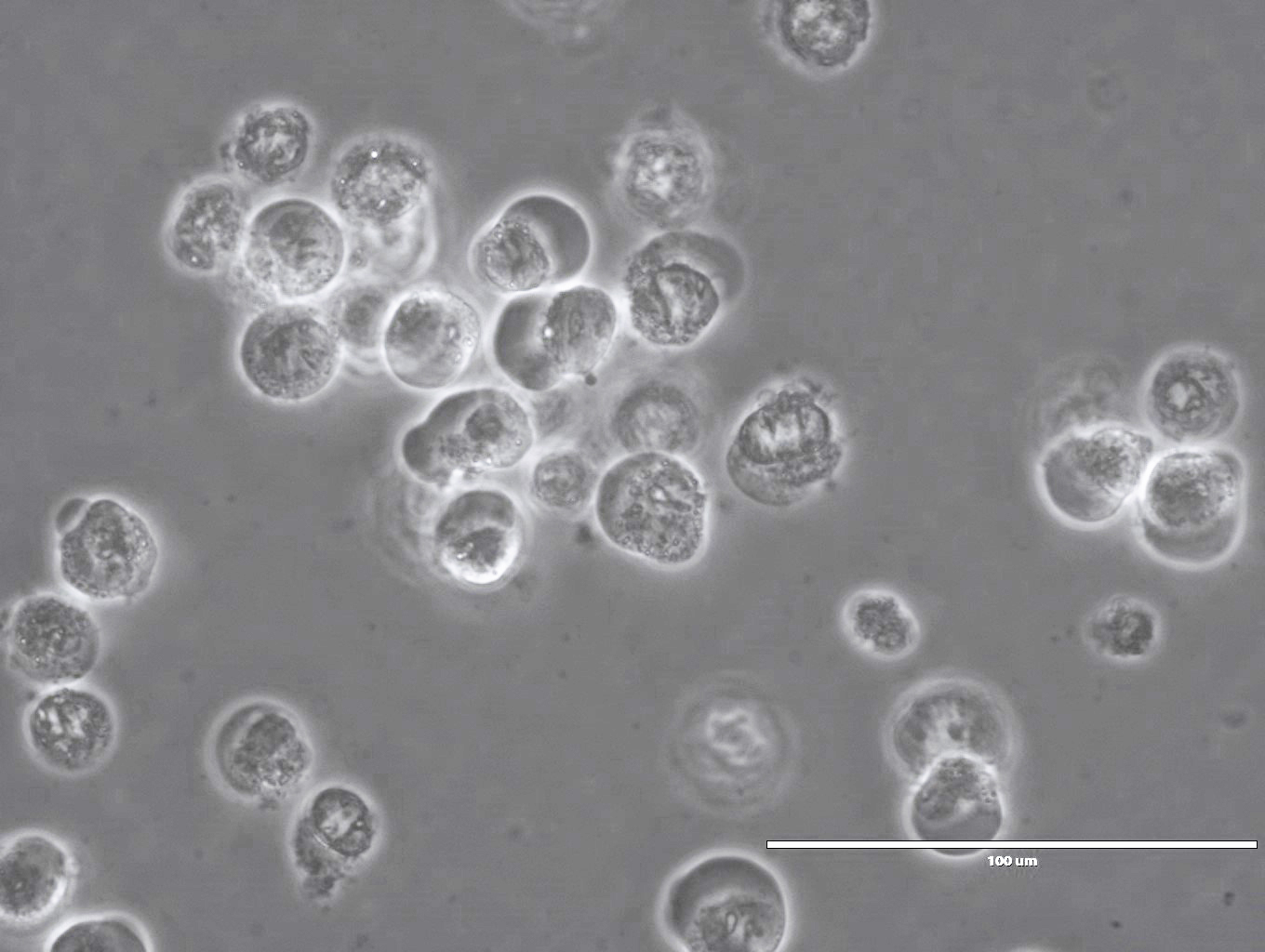

Supplement: Supplementary file 33 — Source data EV and Appendix [file 44318_2025_540_MOESM33_ESM.zip › Source data EV and Appendix/Figure EV 2/2I/12mM/30 min.jpg]

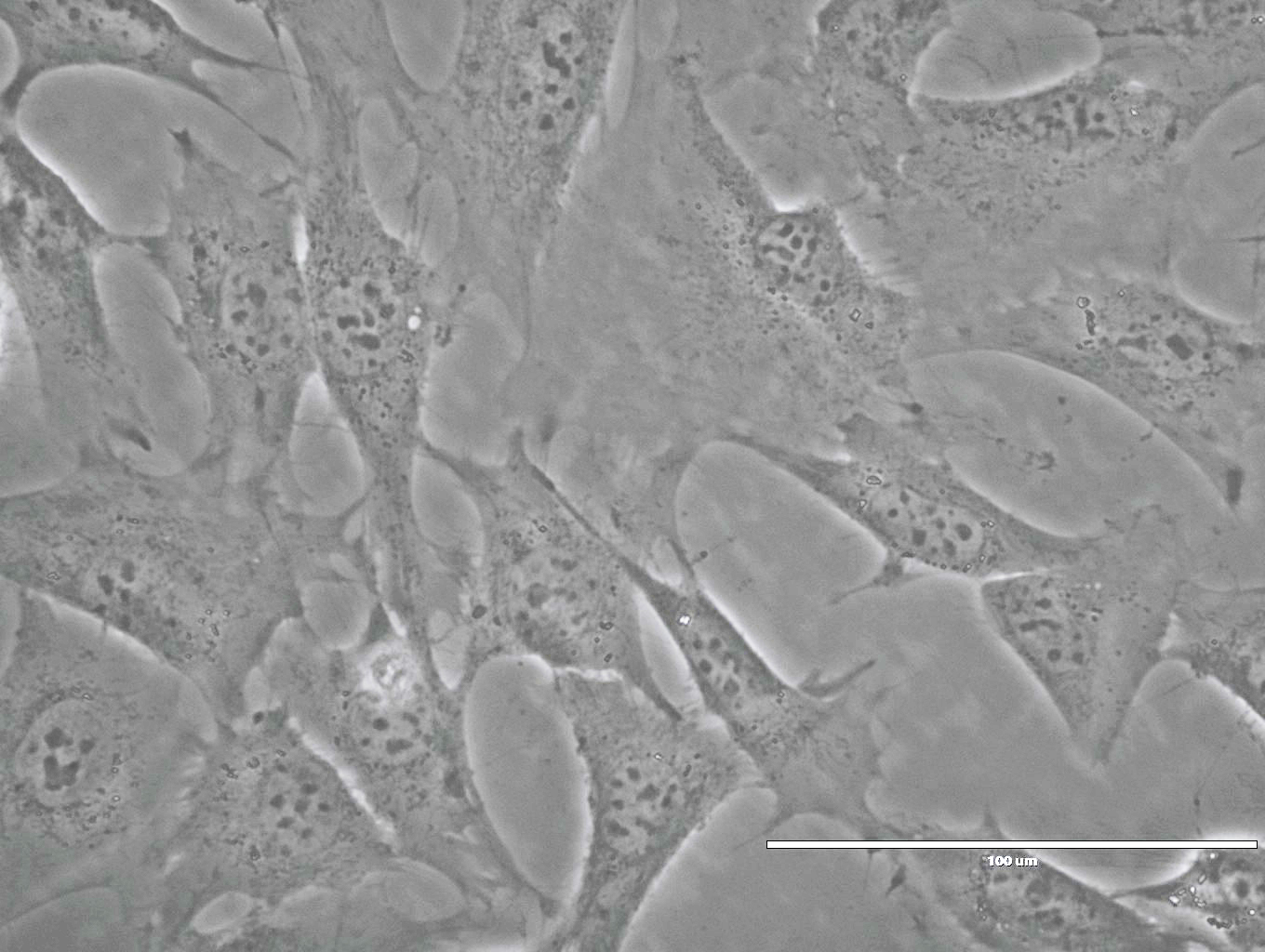

Supplement: Supplementary file 33 — Source data EV and Appendix [file 44318_2025_540_MOESM33_ESM.zip › Source data EV and Appendix/Figure EV 2/2I/8mM/8mM_0 h.jpg]

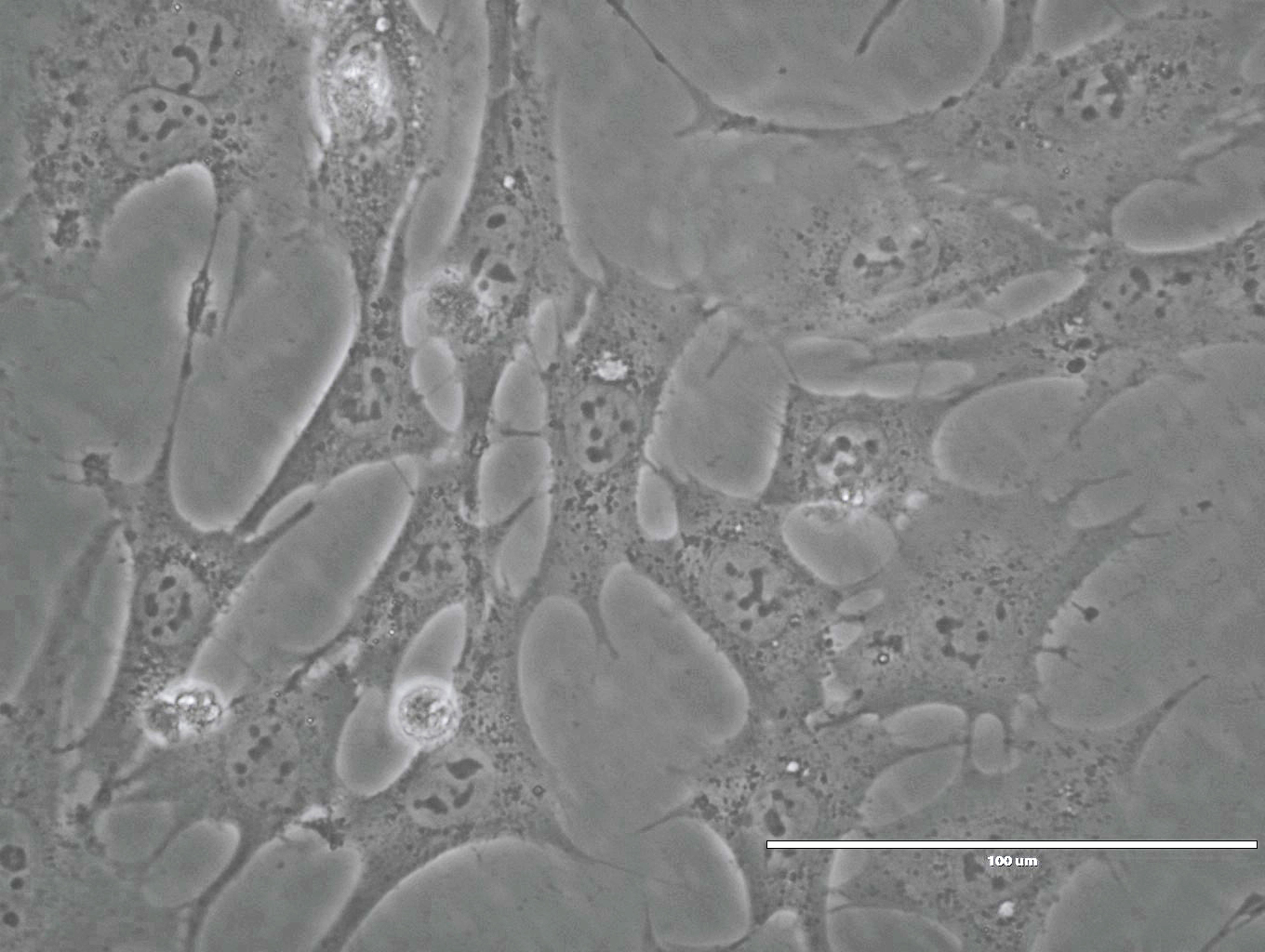

Supplement: Supplementary file 33 — Source data EV and Appendix [file 44318_2025_540_MOESM33_ESM.zip › Source data EV and Appendix/Figure EV 2/2I/8mM/8mM_24 h.jpg]

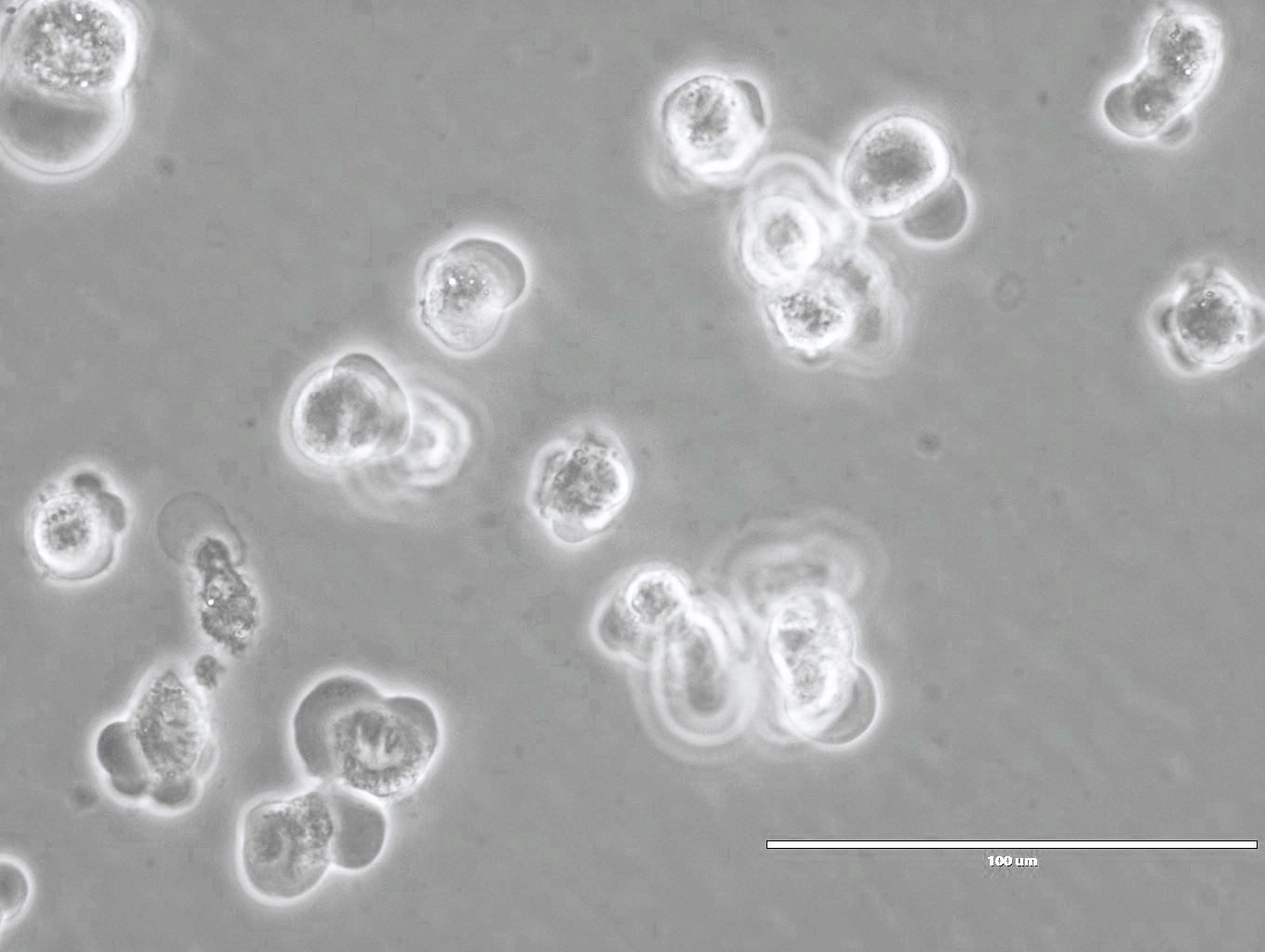

Supplement: Supplementary file 33 — Source data EV and Appendix [file 44318_2025_540_MOESM33_ESM.zip › Source data EV and Appendix/Figure EV 2/2I/8mM/8mM_30 min.jpg]

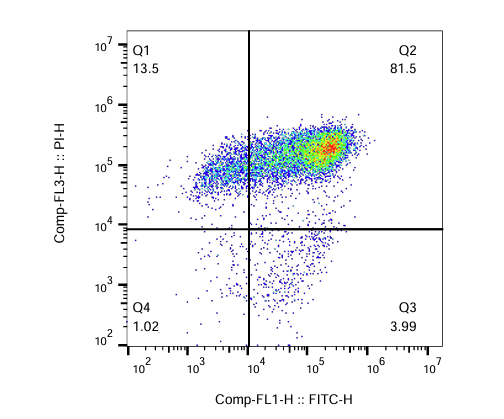

Supplement: Supplementary file 33 — Source data EV and Appendix [file 44318_2025_540_MOESM33_ESM.zip › Source data EV and Appendix/Figure EV 2/2J/LLOMe 16h.png]

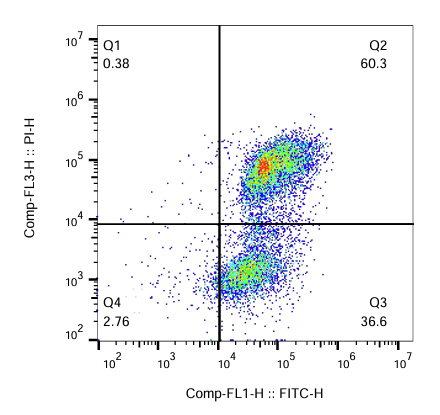

Supplement: Supplementary file 33 — Source data EV and Appendix [file 44318_2025_540_MOESM33_ESM.zip › Source data EV and Appendix/Figure EV 2/2J/LLOMe 30 min.png]

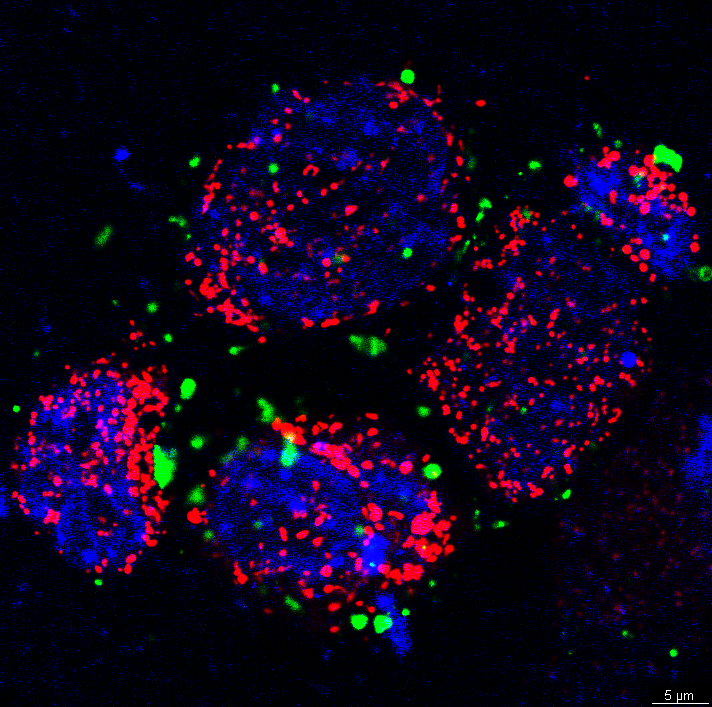

Supplement: Supplementary file 33 — Source data EV and Appendix [file 44318_2025_540_MOESM33_ESM.zip › Source data EV and Appendix/Figure EV 2/2K/Annexin FITC+Mitotracker Red+Hoechst_4mM.tif]

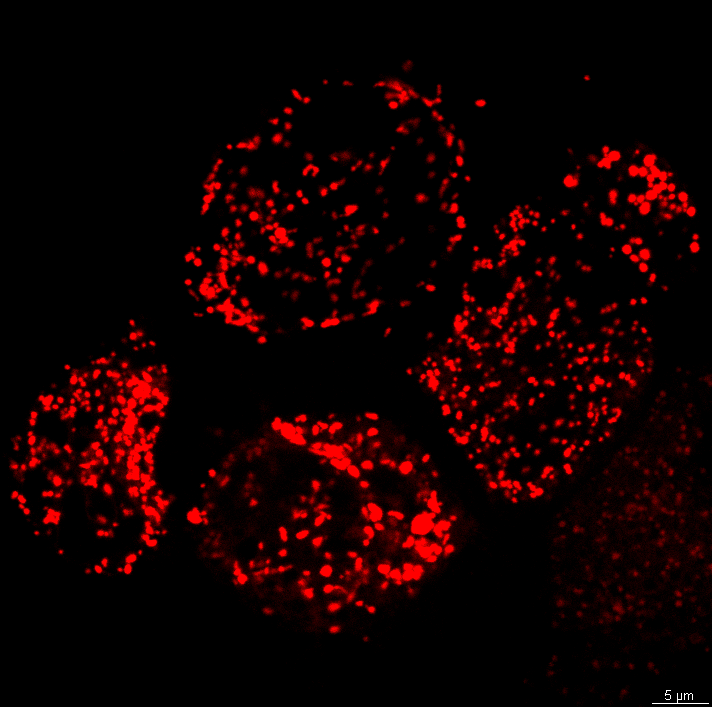

Supplement: Supplementary file 33 — Source data EV and Appendix [file 44318_2025_540_MOESM33_ESM.zip › Source data EV and Appendix/Figure EV 2/2K/Mitotracker Red_4mM.tif]

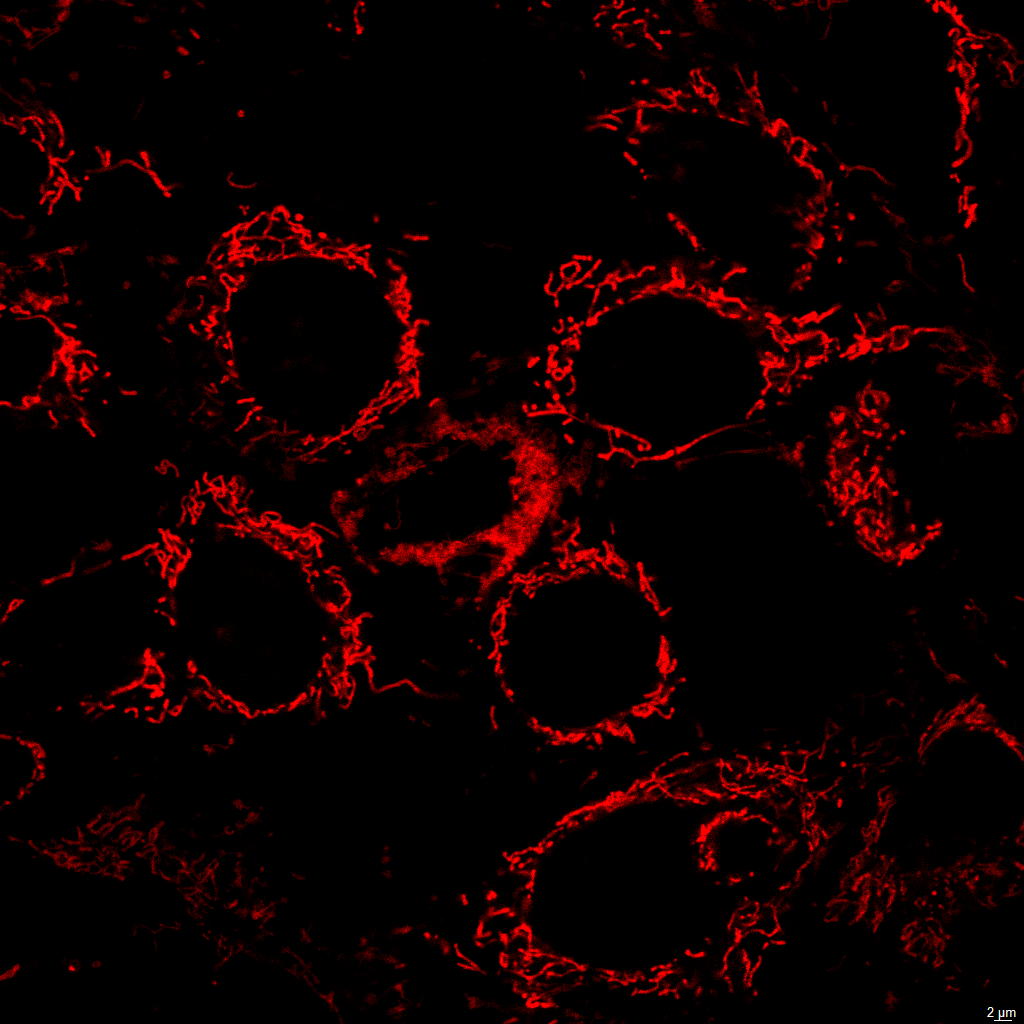

Supplement: Supplementary file 33 — Source data EV and Appendix [file 44318_2025_540_MOESM33_ESM.zip › Source data EV and Appendix/Figure EV 2/2K/Mitotracker_Control.tif]

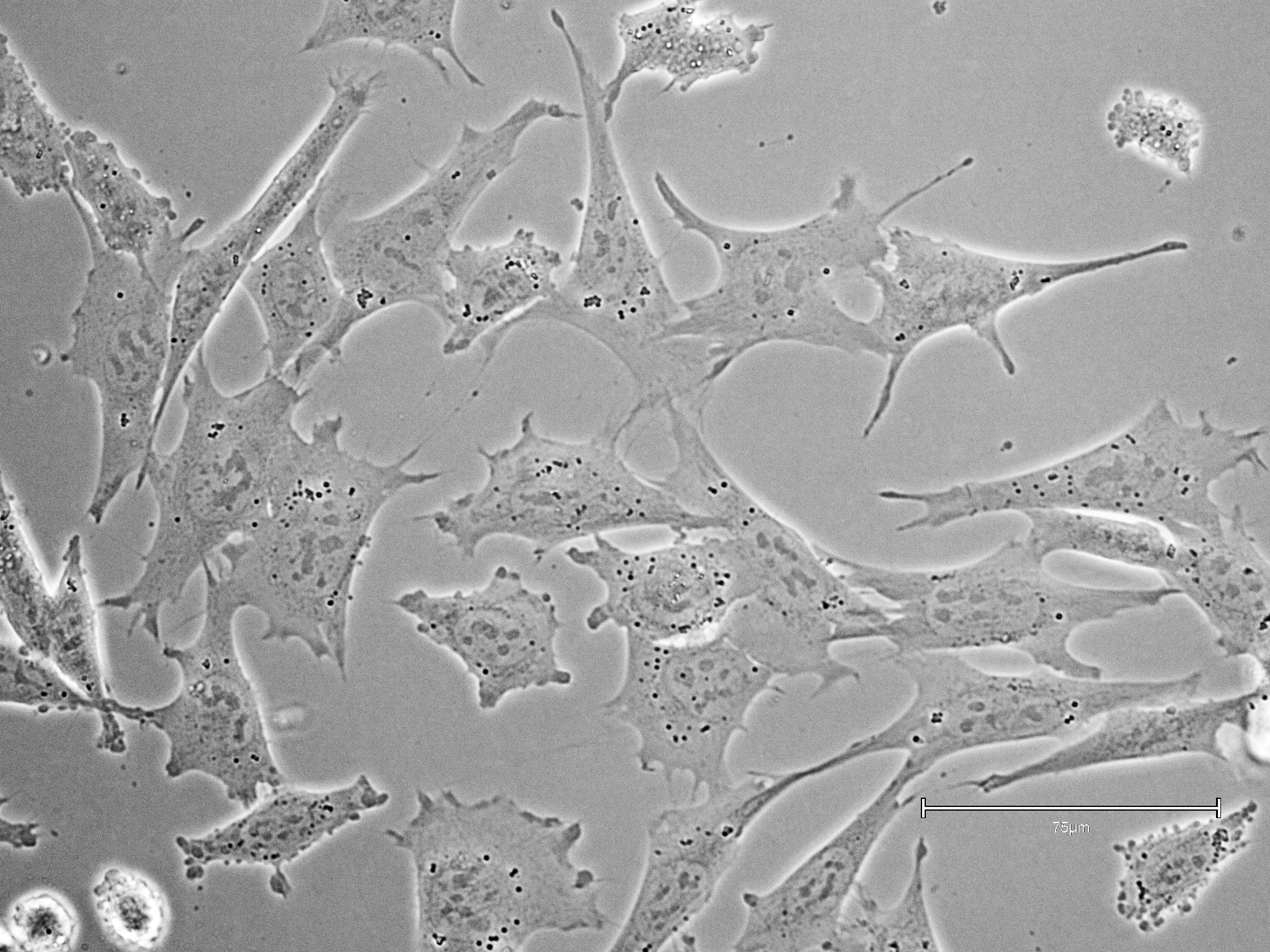

Supplement: Supplementary file 33 — Source data EV and Appendix [file 44318_2025_540_MOESM33_ESM.zip › Source data EV and Appendix/Figure EV 2/2N/0uM/0h.tif]

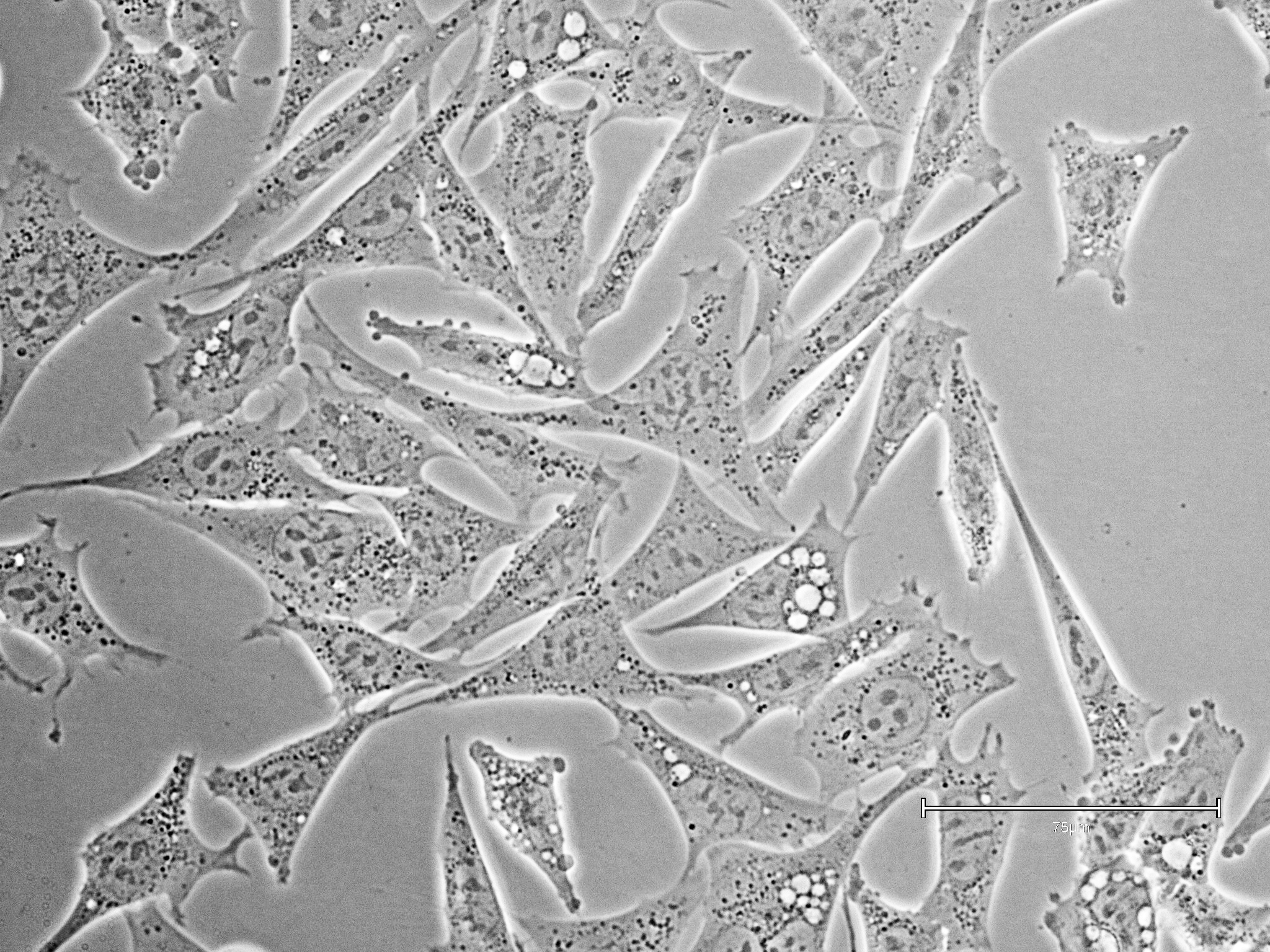

Supplement: Supplementary file 33 — Source data EV and Appendix [file 44318_2025_540_MOESM33_ESM.zip › Source data EV and Appendix/Figure EV 2/2N/0uM/12h.jpg]

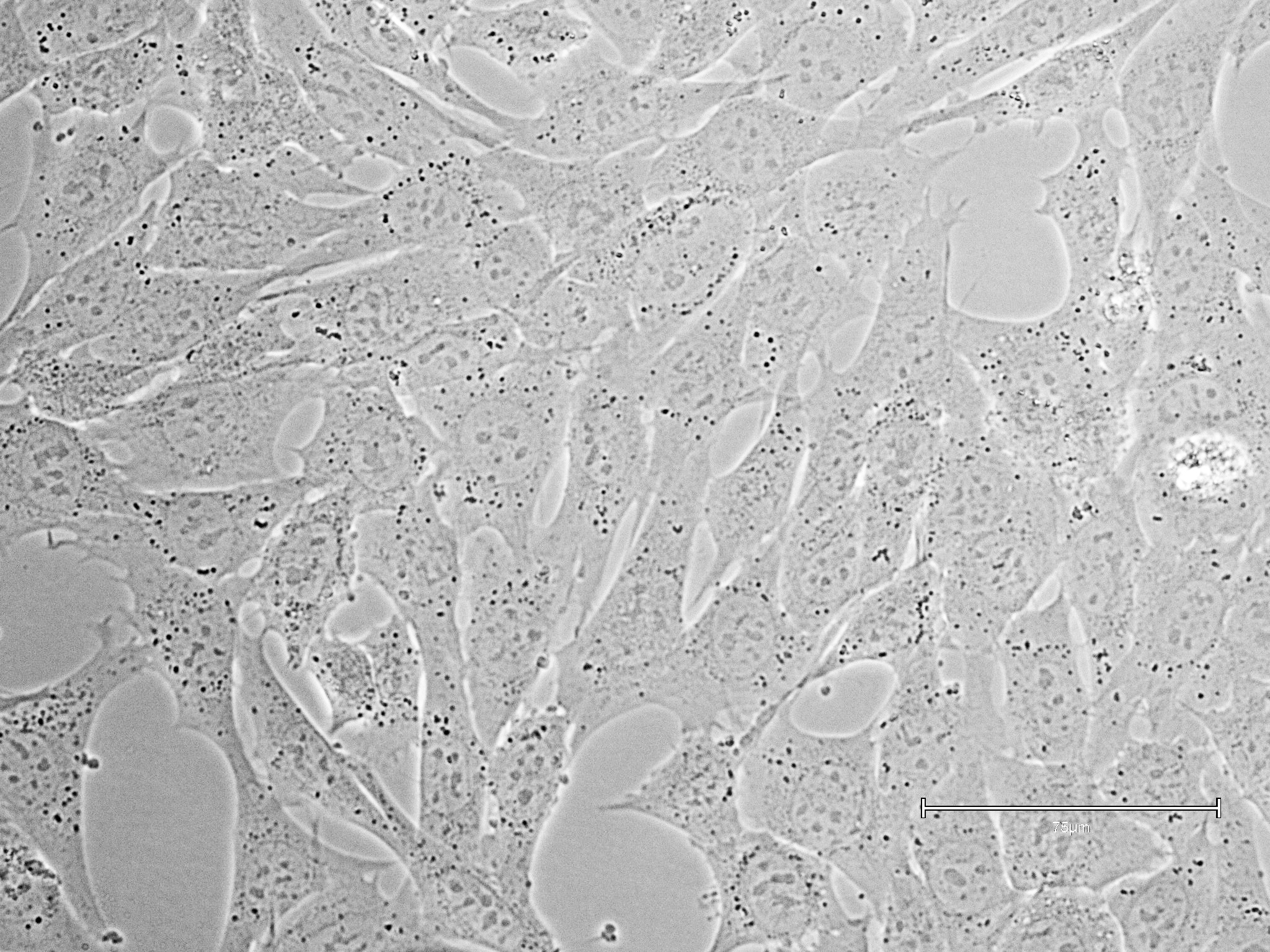

Supplement: Supplementary file 33 — Source data EV and Appendix [file 44318_2025_540_MOESM33_ESM.zip › Source data EV and Appendix/Figure EV 2/2N/0uM/24h.jpg]

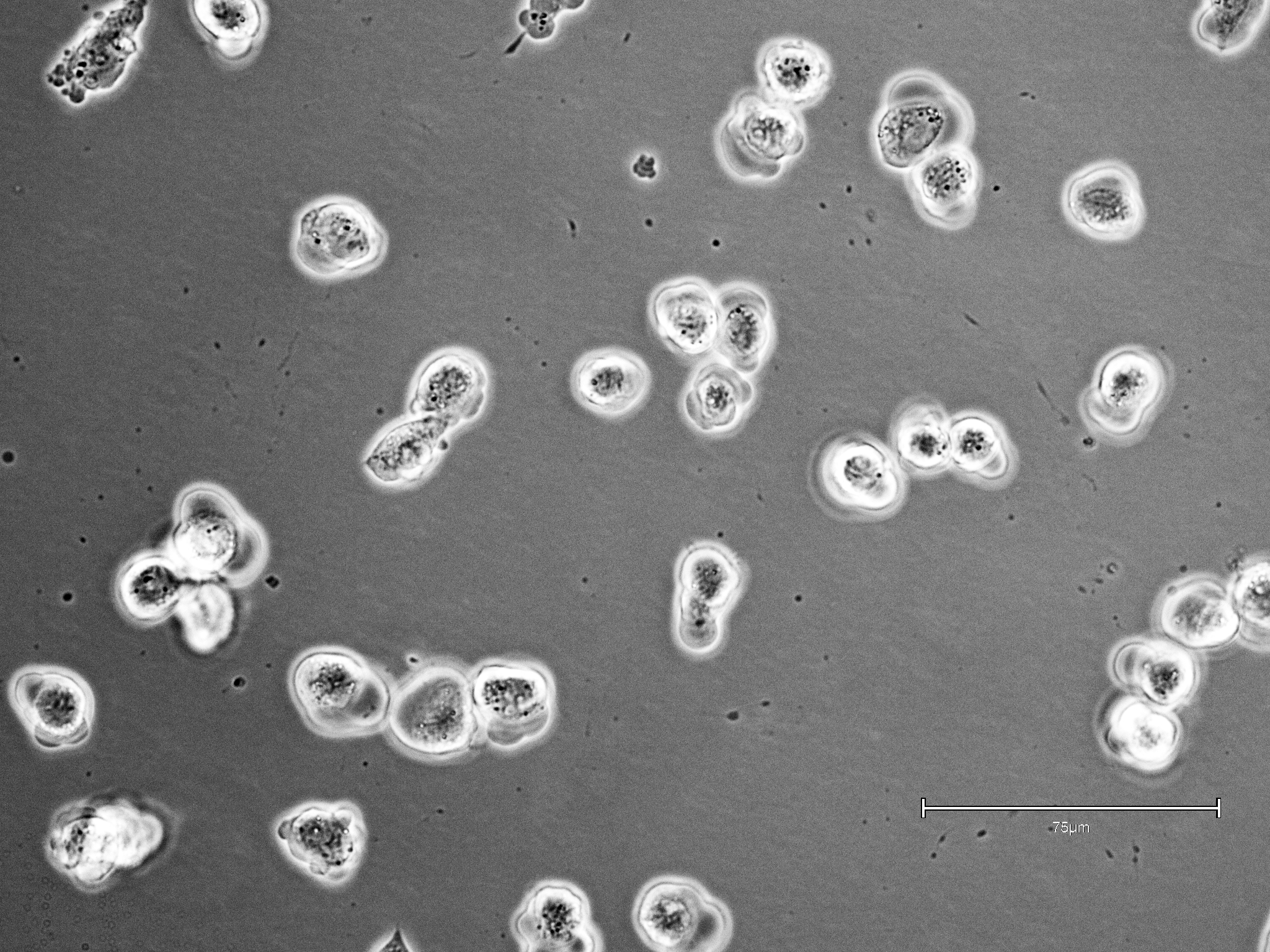

Supplement: Supplementary file 33 — Source data EV and Appendix [file 44318_2025_540_MOESM33_ESM.zip › Source data EV and Appendix/Figure EV 2/2N/0uM/30mins.tif]

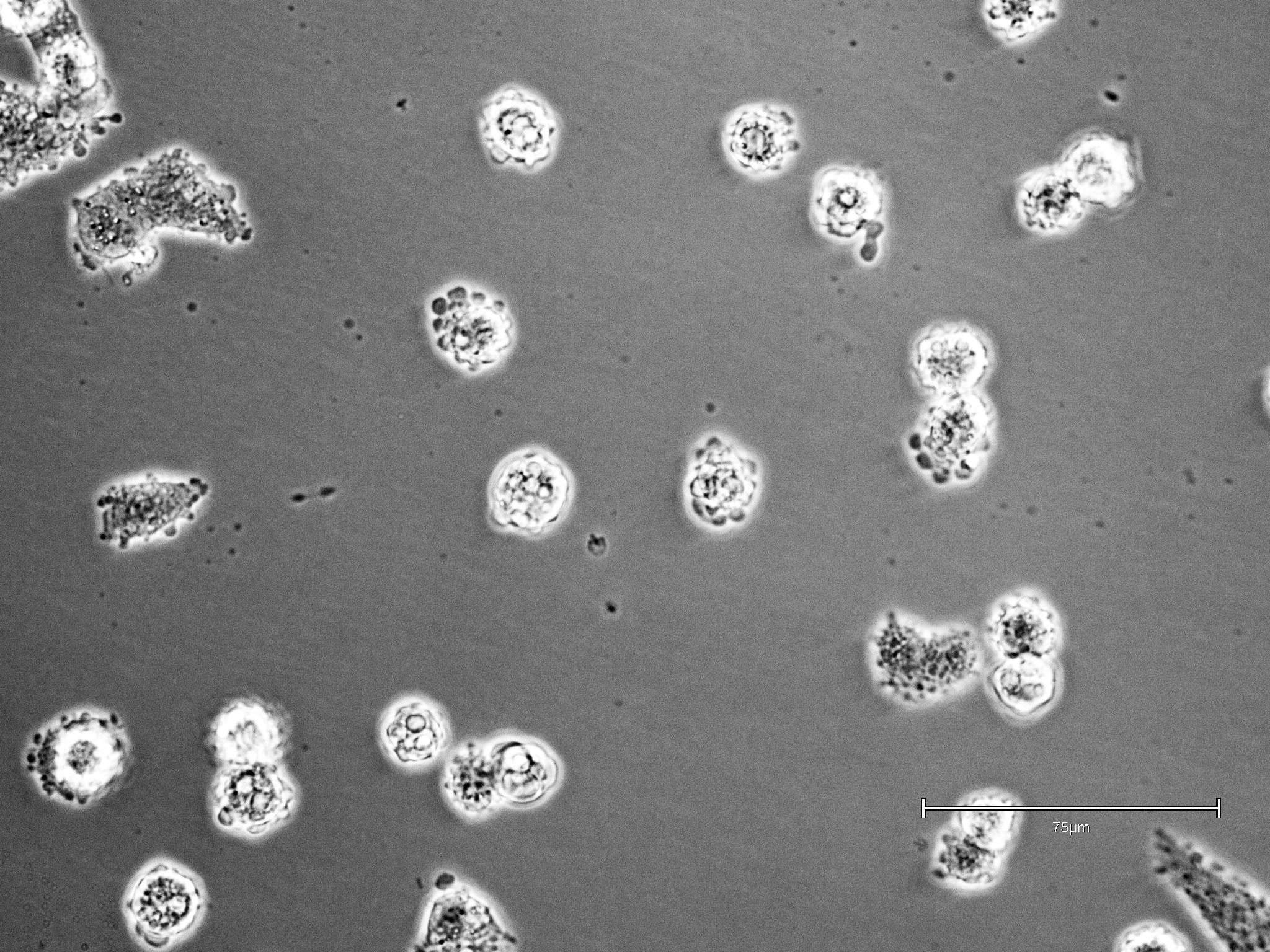

Supplement: Supplementary file 33 — Source data EV and Appendix [file 44318_2025_540_MOESM33_ESM.zip › Source data EV and Appendix/Figure EV 2/2N/0uM/3h.tif]

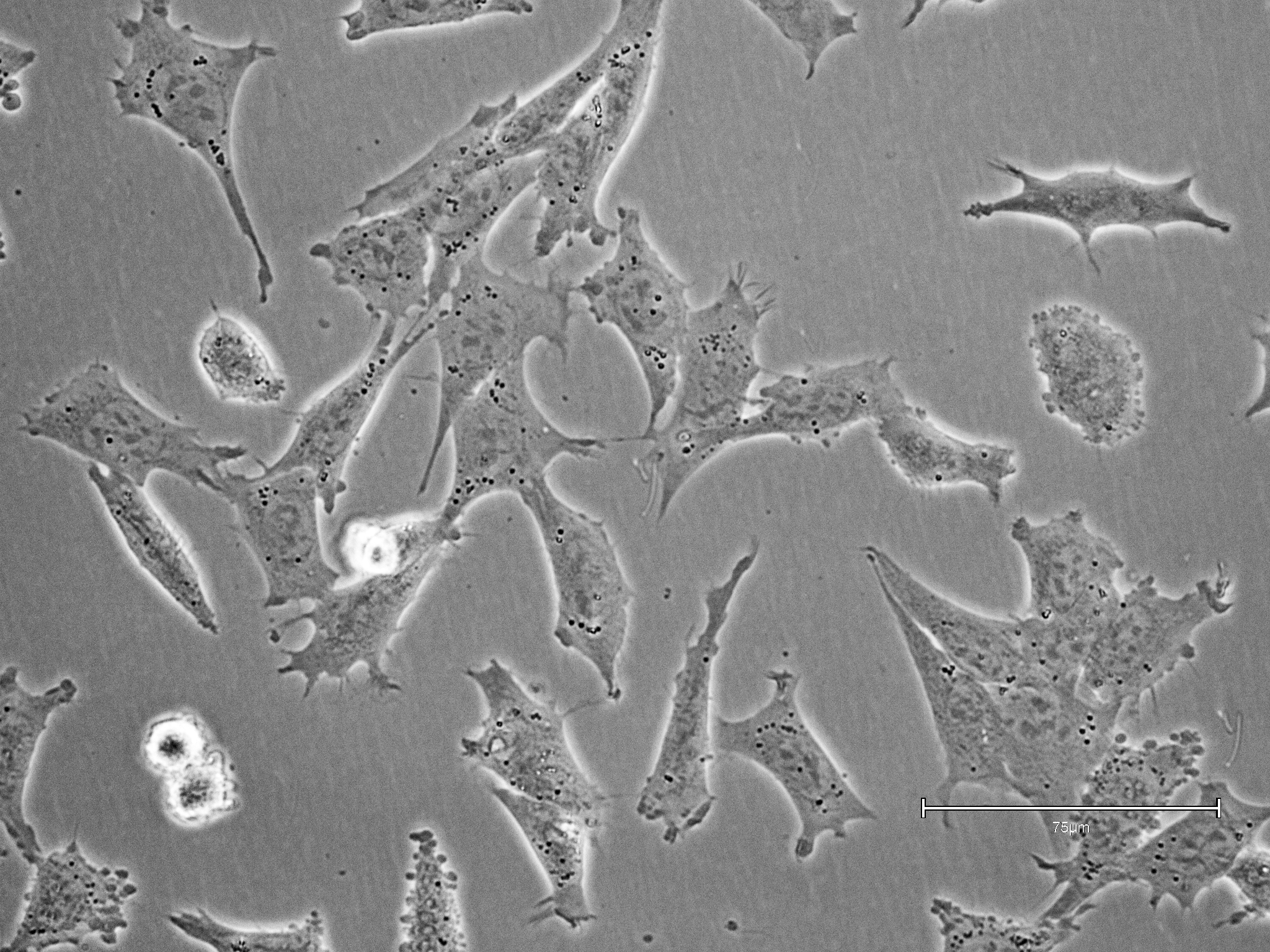

Supplement: Supplementary file 33 — Source data EV and Appendix [file 44318_2025_540_MOESM33_ESM.zip › Source data EV and Appendix/Figure EV 2/2N/20uM/0h.tif]

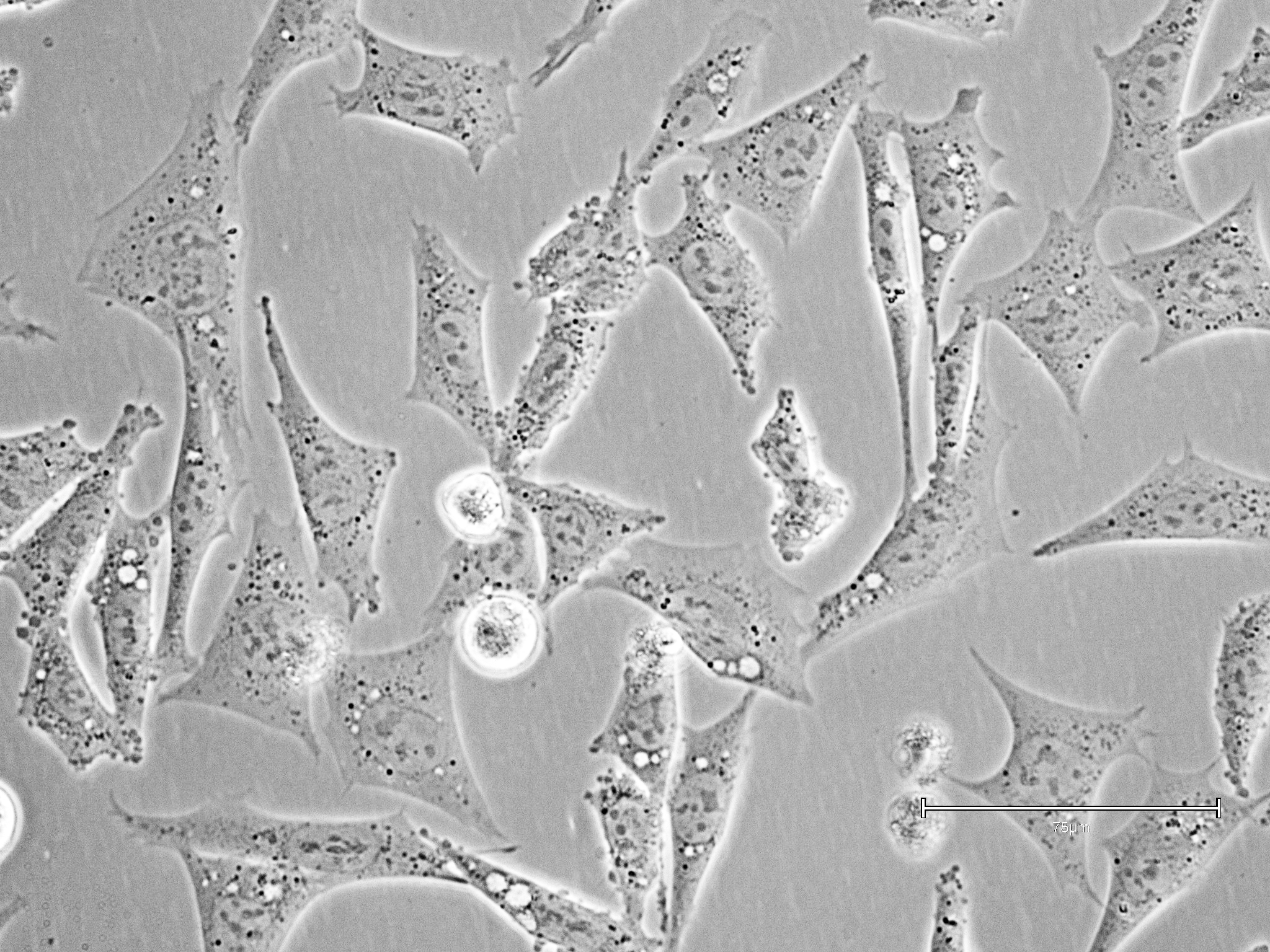

Supplement: Supplementary file 33 — Source data EV and Appendix [file 44318_2025_540_MOESM33_ESM.zip › Source data EV and Appendix/Figure EV 2/2N/20uM/12h.jpg]

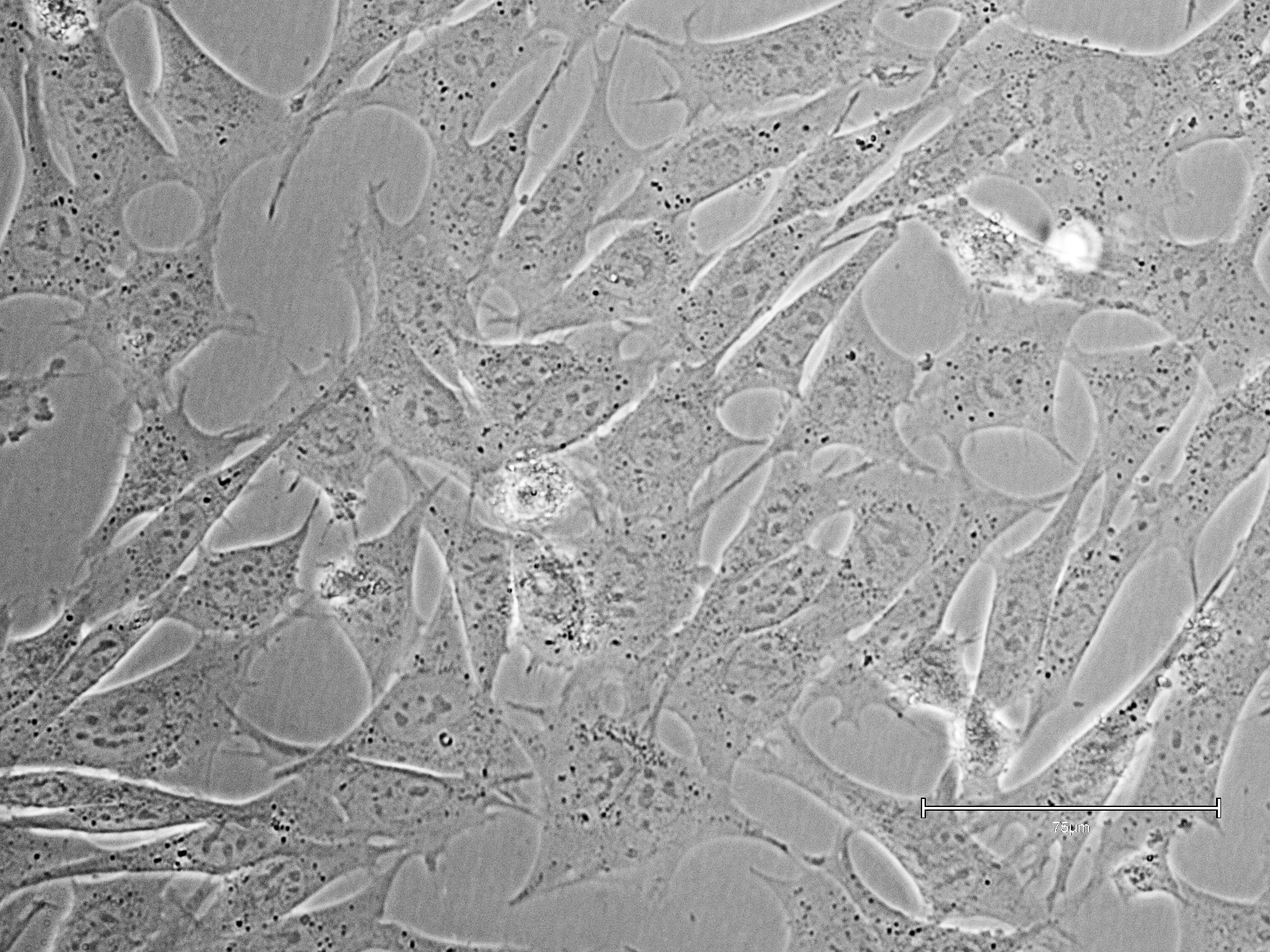

Supplement: Supplementary file 33 — Source data EV and Appendix [file 44318_2025_540_MOESM33_ESM.zip › Source data EV and Appendix/Figure EV 2/2N/20uM/24h.jpg]

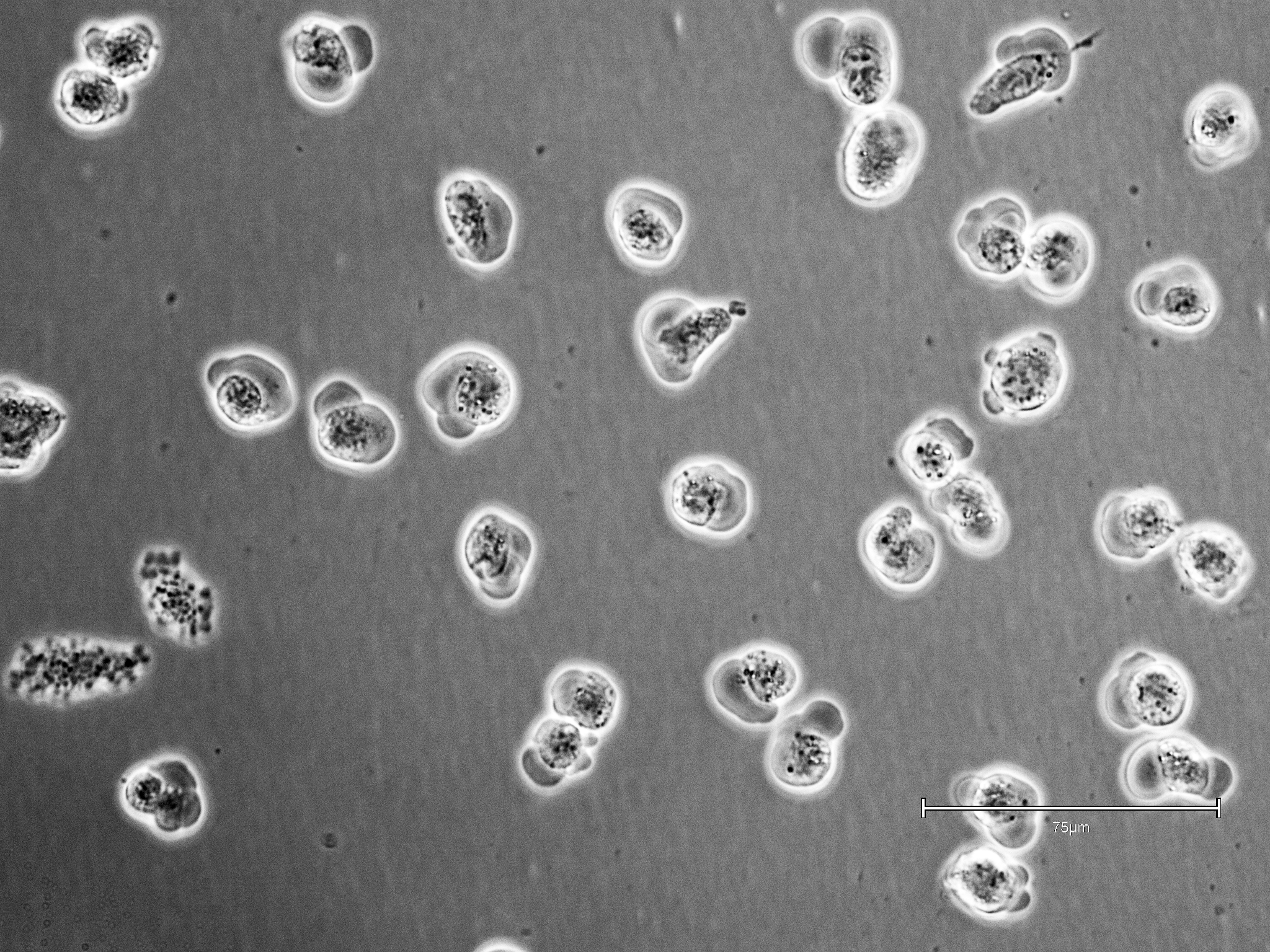

Supplement: Supplementary file 33 — Source data EV and Appendix [file 44318_2025_540_MOESM33_ESM.zip › Source data EV and Appendix/Figure EV 2/2N/20uM/30mins.tif]

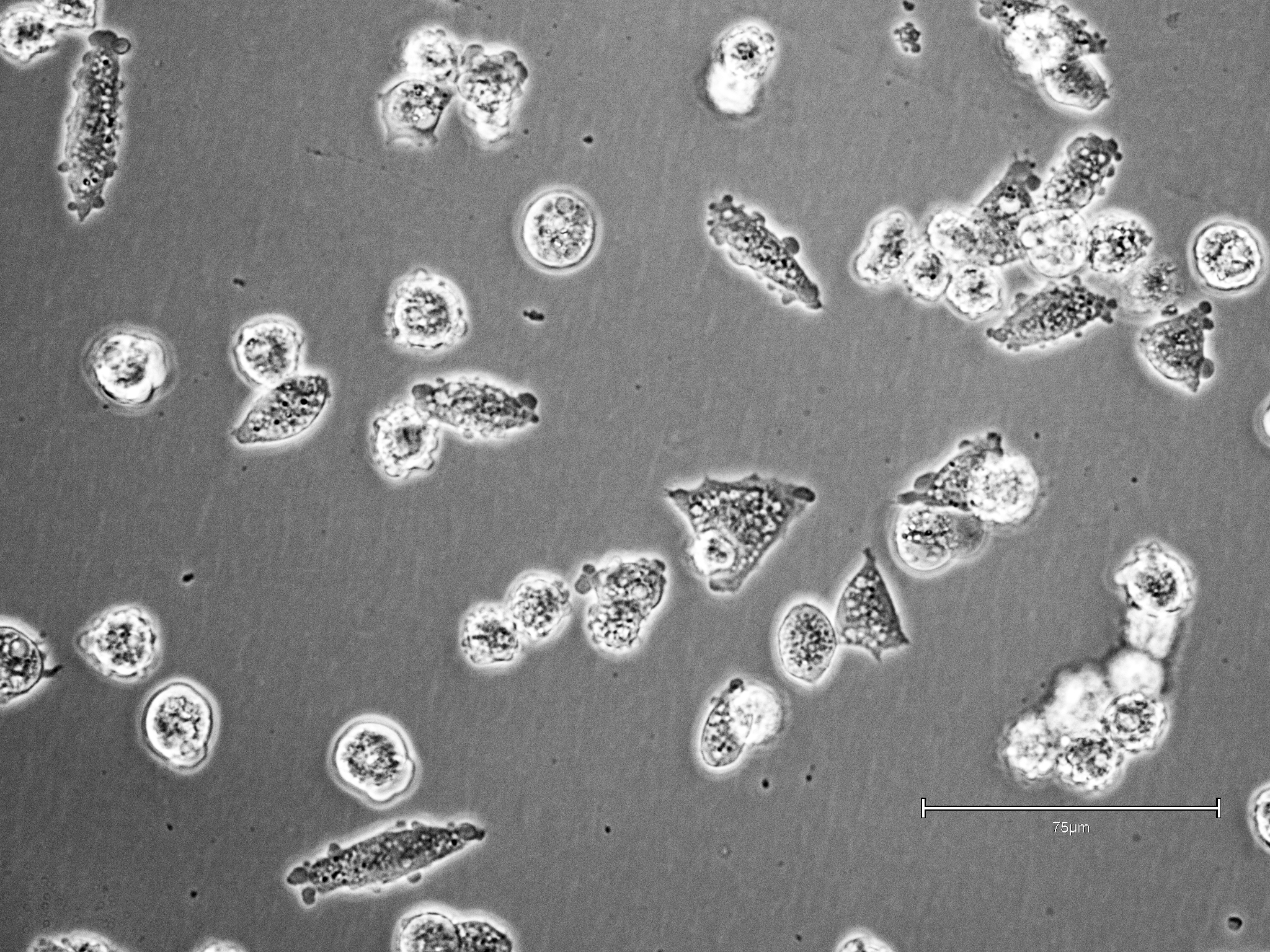

Supplement: Supplementary file 33 — Source data EV and Appendix [file 44318_2025_540_MOESM33_ESM.zip › Source data EV and Appendix/Figure EV 2/2N/20uM/3h.tif]

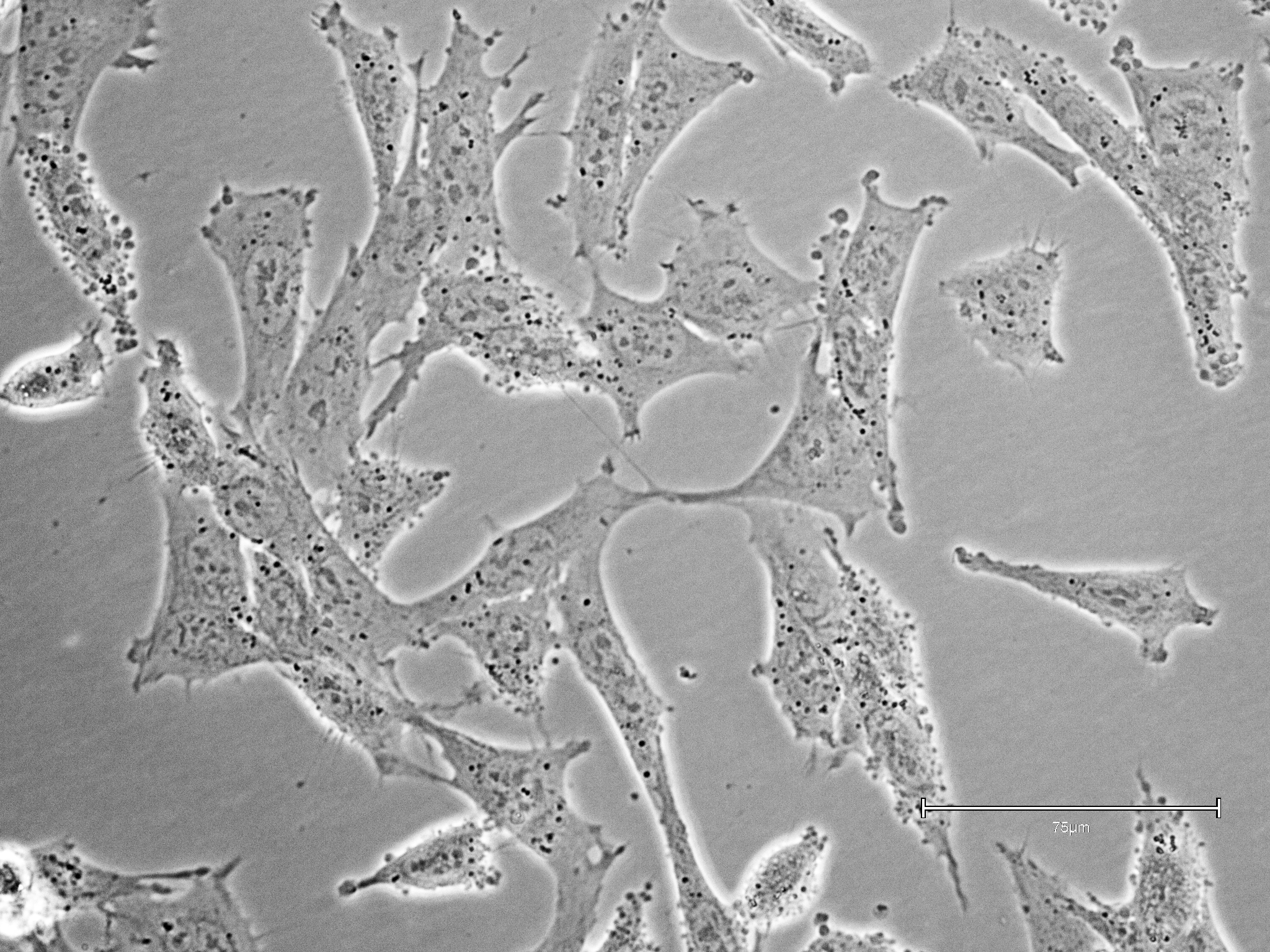

Supplement: Supplementary file 33 — Source data EV and Appendix [file 44318_2025_540_MOESM33_ESM.zip › Source data EV and Appendix/Figure EV 2/2N/40uM/0h.tif]

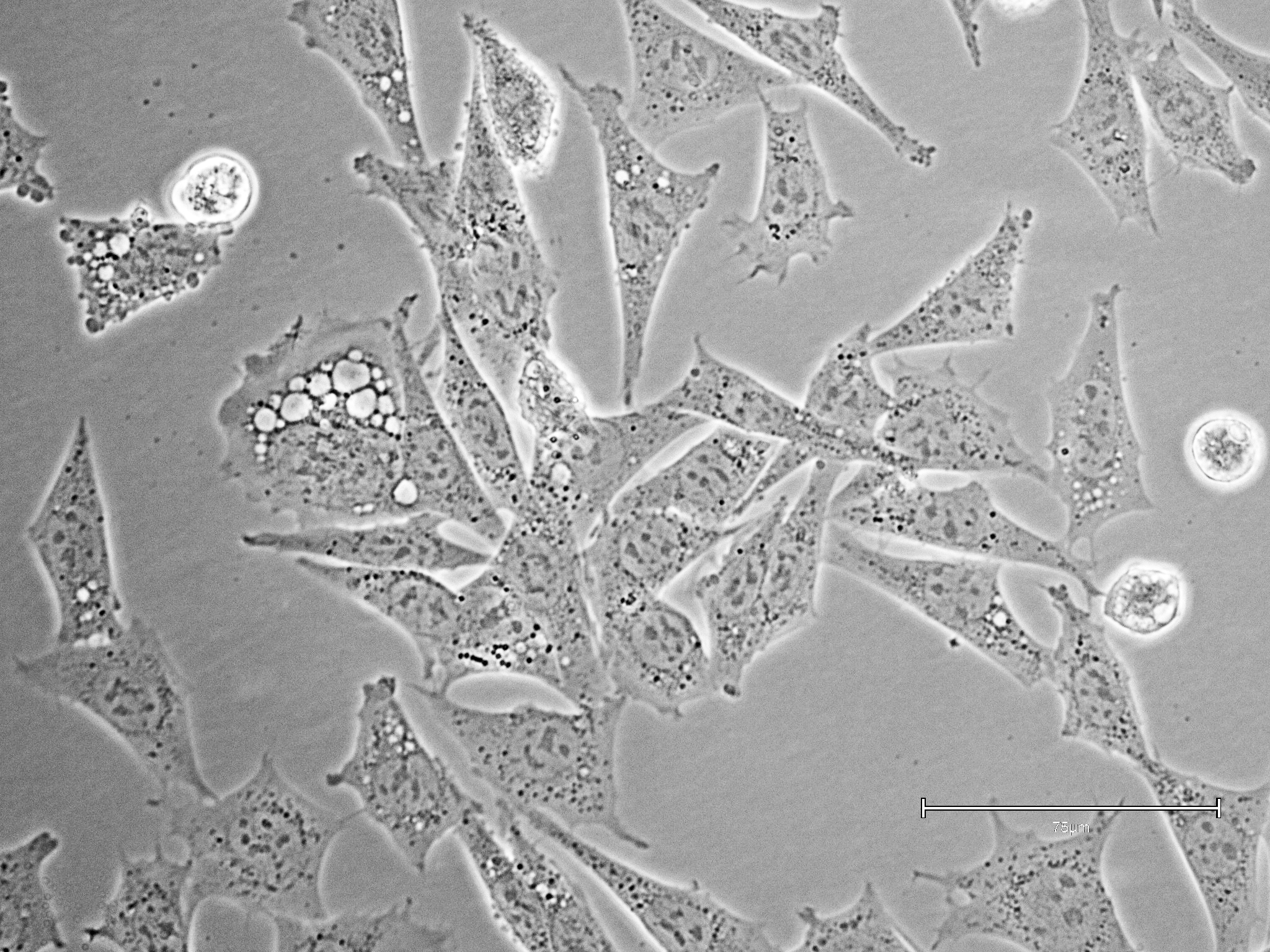

Supplement: Supplementary file 33 — Source data EV and Appendix [file 44318_2025_540_MOESM33_ESM.zip › Source data EV and Appendix/Figure EV 2/2N/40uM/12h.jpg]

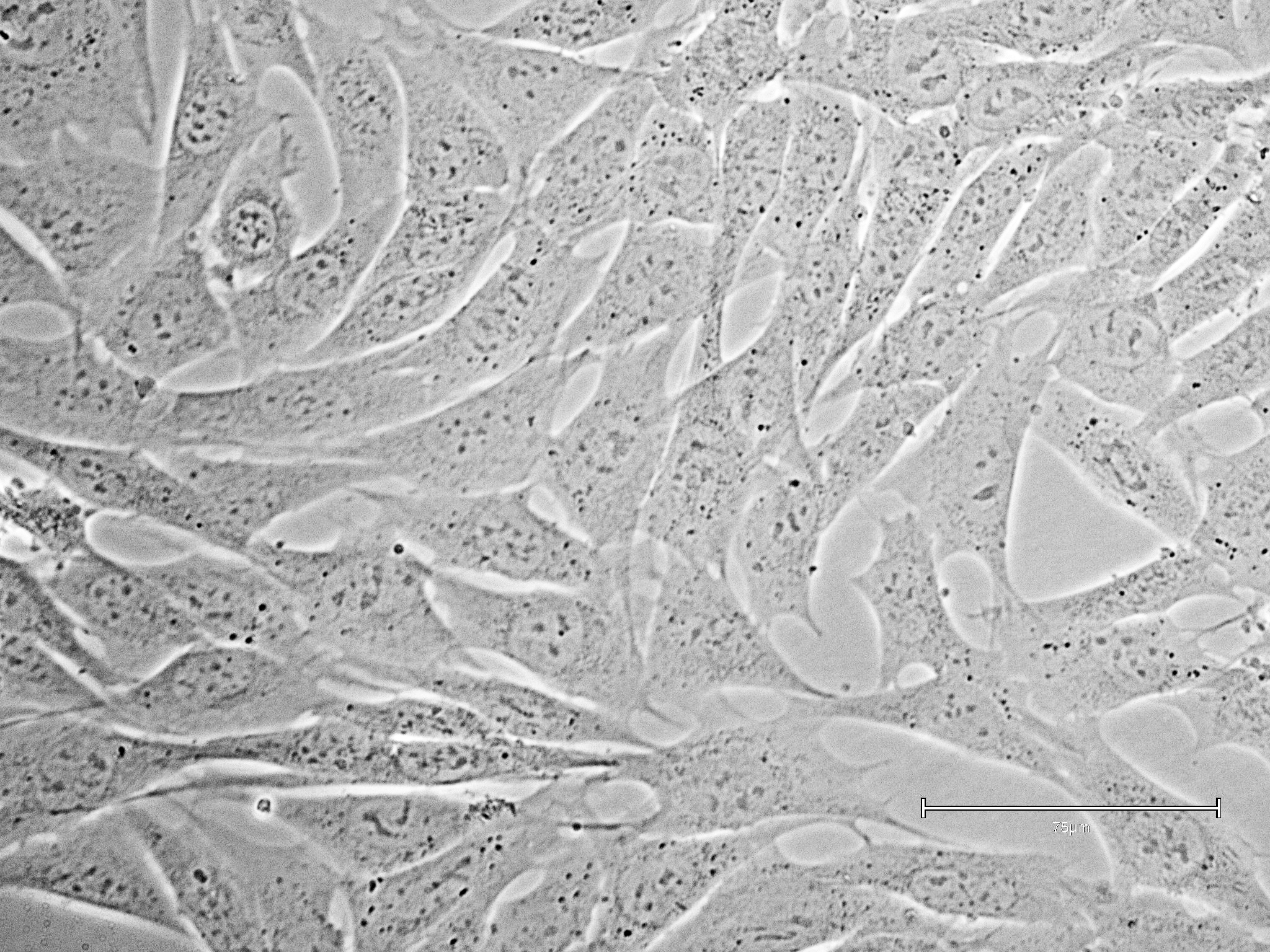

Supplement: Supplementary file 33 — Source data EV and Appendix [file 44318_2025_540_MOESM33_ESM.zip › Source data EV and Appendix/Figure EV 2/2N/40uM/24h.jpg]

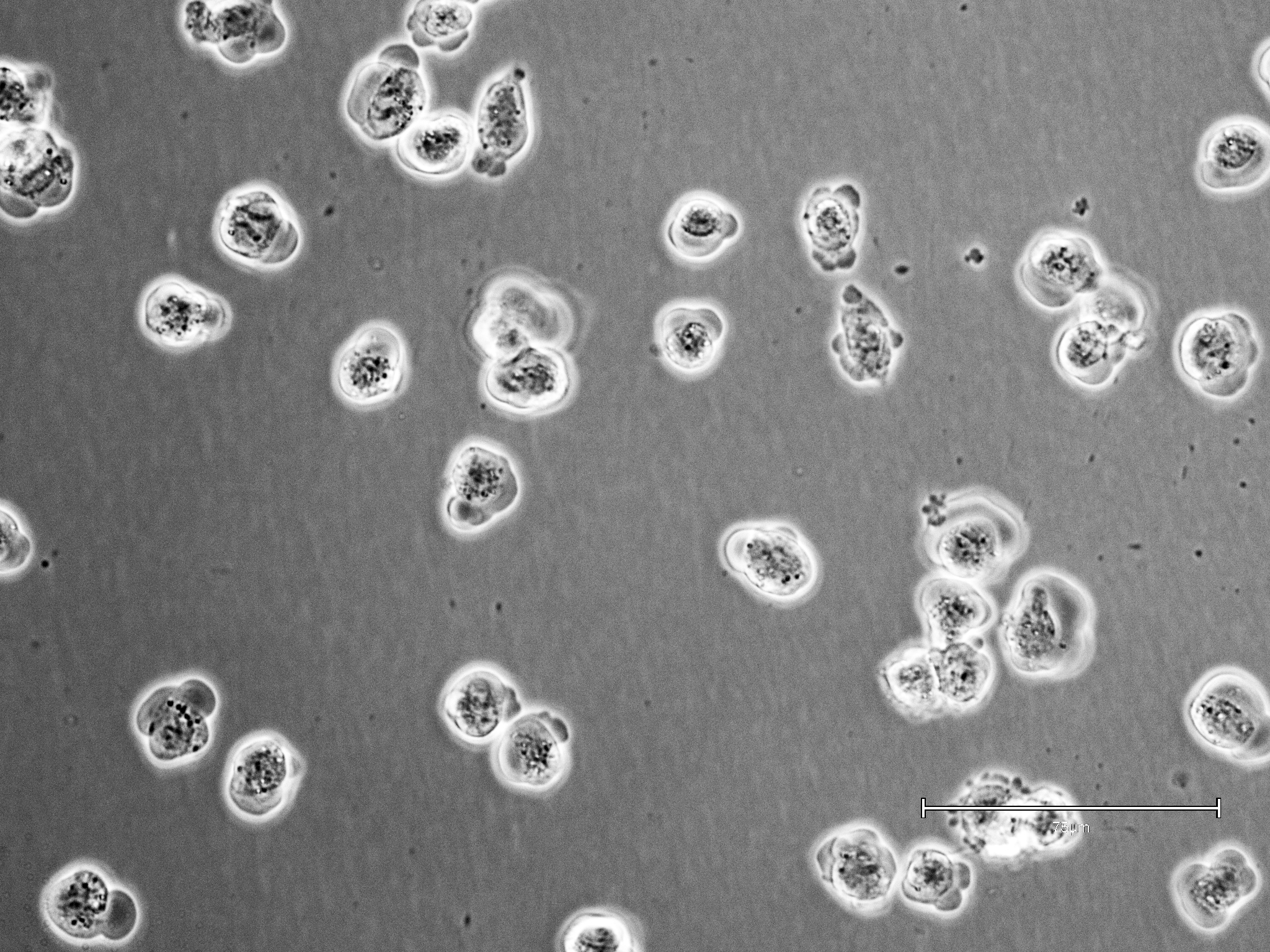

Supplement: Supplementary file 33 — Source data EV and Appendix [file 44318_2025_540_MOESM33_ESM.zip › Source data EV and Appendix/Figure EV 2/2N/40uM/30mins.tif]

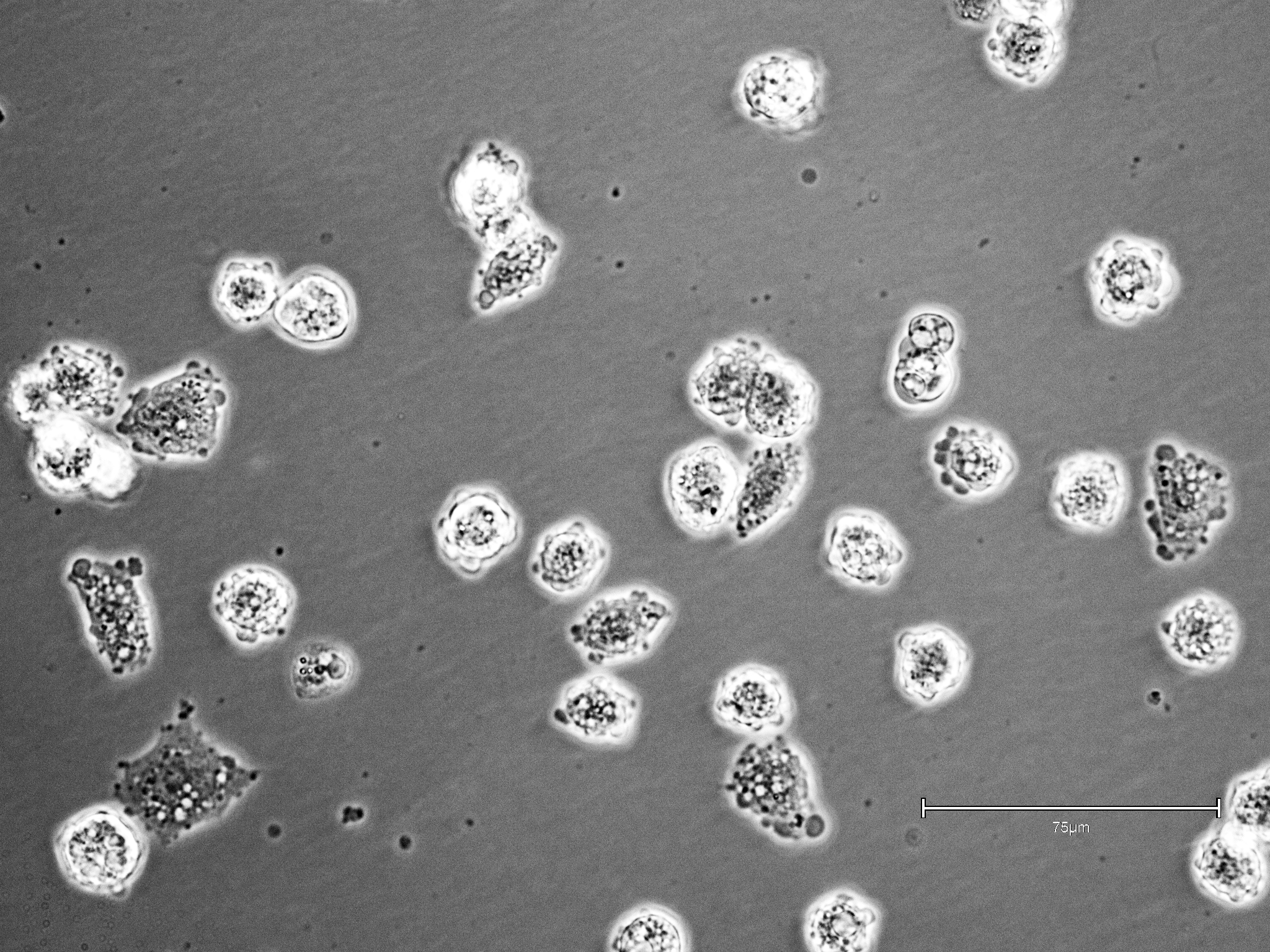

Supplement: Supplementary file 33 — Source data EV and Appendix [file 44318_2025_540_MOESM33_ESM.zip › Source data EV and Appendix/Figure EV 2/2N/40uM/3h.tif]

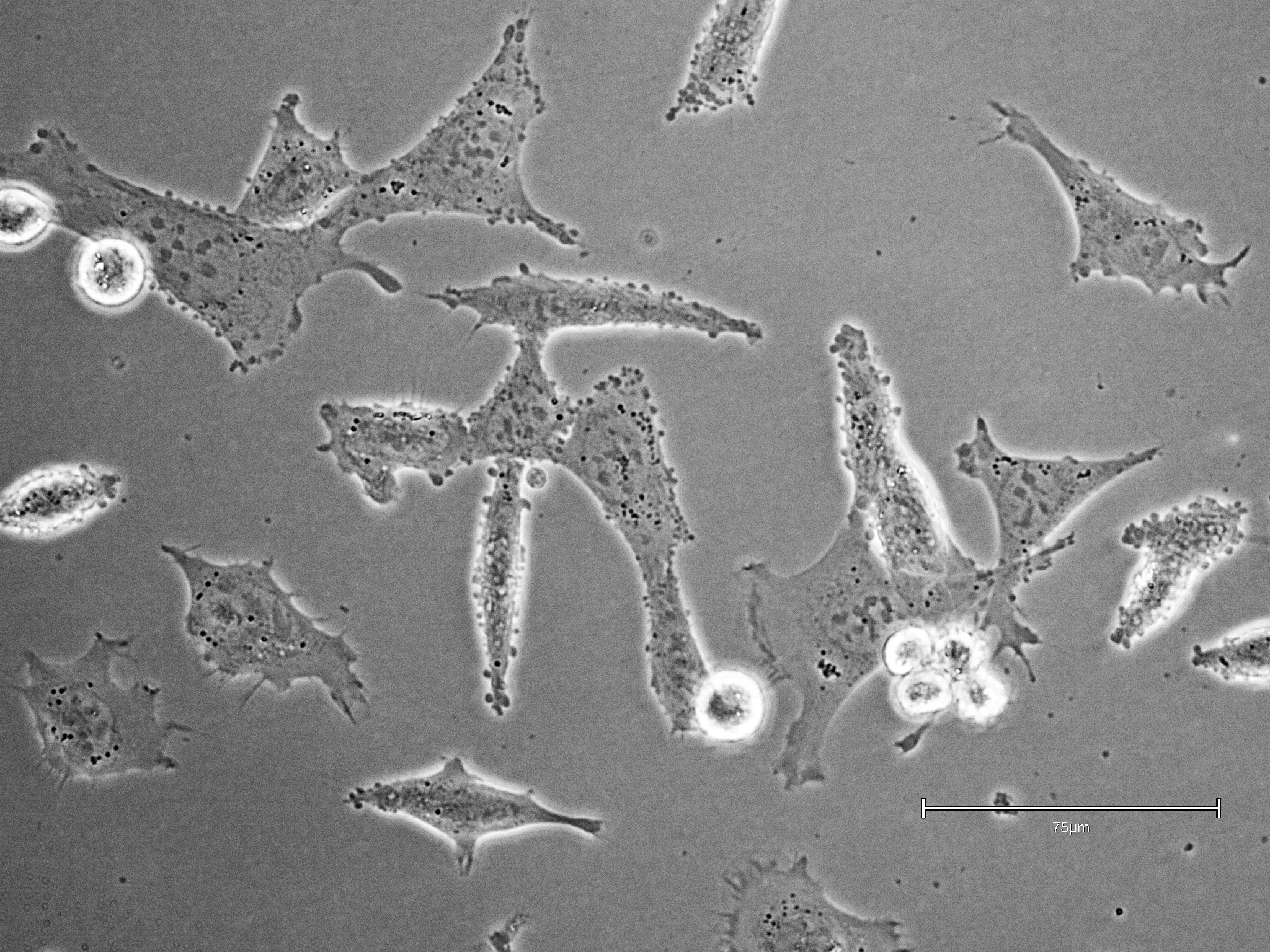

Supplement: Supplementary file 33 — Source data EV and Appendix [file 44318_2025_540_MOESM33_ESM.zip › Source data EV and Appendix/Figure EV 2/2N/80uM/0h.tif]

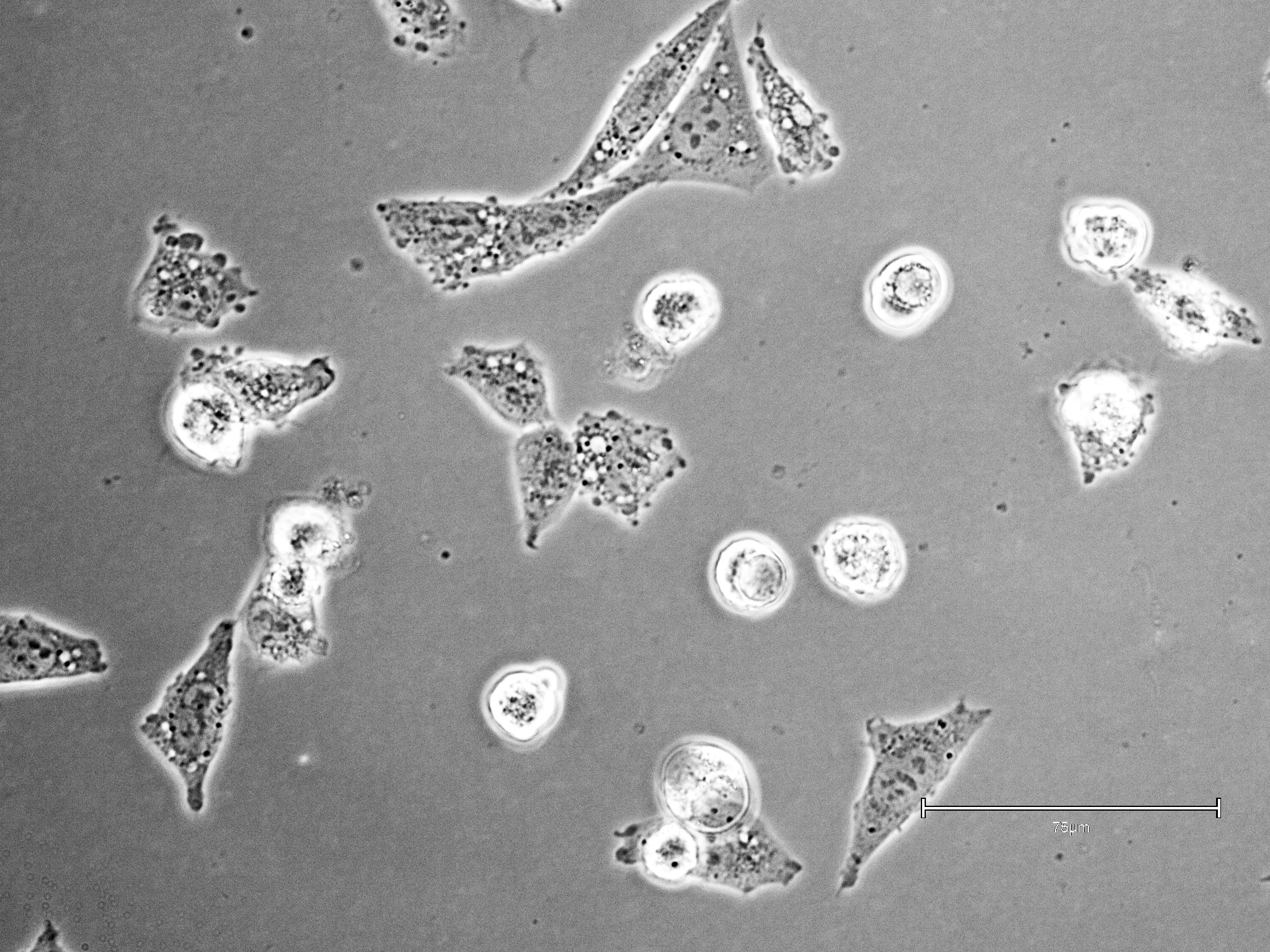

Supplement: Supplementary file 33 — Source data EV and Appendix [file 44318_2025_540_MOESM33_ESM.zip › Source data EV and Appendix/Figure EV 2/2N/80uM/12h.jpg]
