# Supplementary material for: Programmed cell revival from imminent cell death enhances tissue repair and regeneration
Source: EMBO J. 2025 Aug 21;44(19):5244–89. doi: 10.1038/s44318-025-00540-y (PMC12489119; doi:10.1038/s44318-025-00540-y)
Supplement: Supplementary file 34 — Expanded View Figures [file 44318_2025_540_MOESM34_ESM.pdf]

Expanded View Figures

Figure EV1. Cells resuscitate from cell death-like conditions.

(**A**) Snapshots of time-lapse live microscopy images of MEF cells treated with 4 mM LLOMe. Magnification 10X. Scale bar, 400 µm. (**B**) MEF cells were treated with 4 mM LLOMe. The floating cells were collected and washed with PBS before plating in a new dish. Snapshot images of live microscopy of MEF cells after replating. Refer to Movie EV3. Dotted circles represent the revival of the indicated floating cells at the indicated time points. (**C**)The graph depicts the quantification of the percentage of the well covered by the object (area of the well covered by cells) from time-lapse live microscopy. (**D**) Representative time-lapse live microscopy images of MEF cells treated with GPN (200 µM), sphingosine (15 µM), LLOMe (4 mM), Siramesine (200 µM), Alum (1 mg/ml), and Silica (1200 µg/ml). Magnification 10X. Scale bar, 400 µm. (**E**) Representative time-lapse live microscopy images of different cell lines treated with LLOMe. (**F**) The table depicts the cell lines treated with LLOMe, the concentrations of LLOMe used, and the morphological changes. Magnification 40X. Scale bar, 100 µm. (**G**) Representative time-lapse live microscopy images of BMDMs cells treated with 0.25 and 0.5 mM LLOMe. Magnification 40X. Scale bar, 75 µm. Source data are available online for this figure.

© The Author(s)

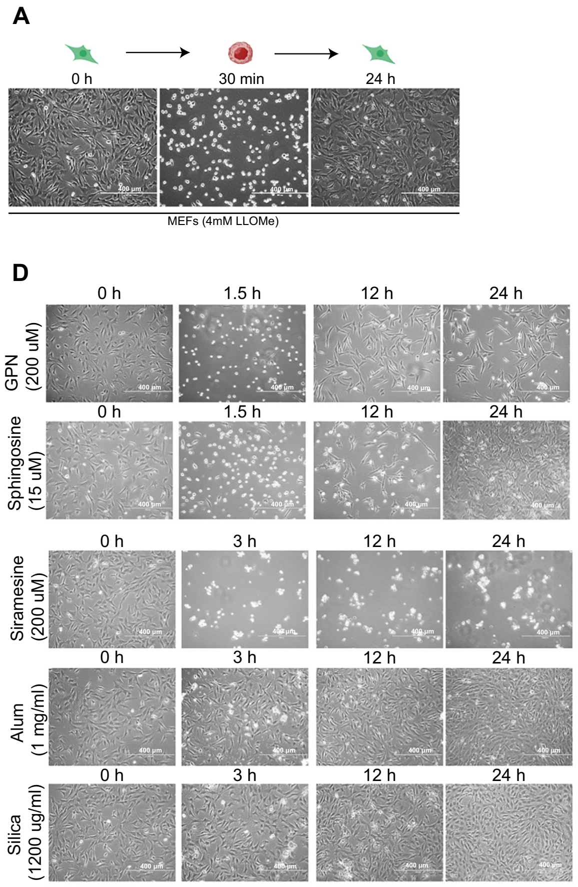

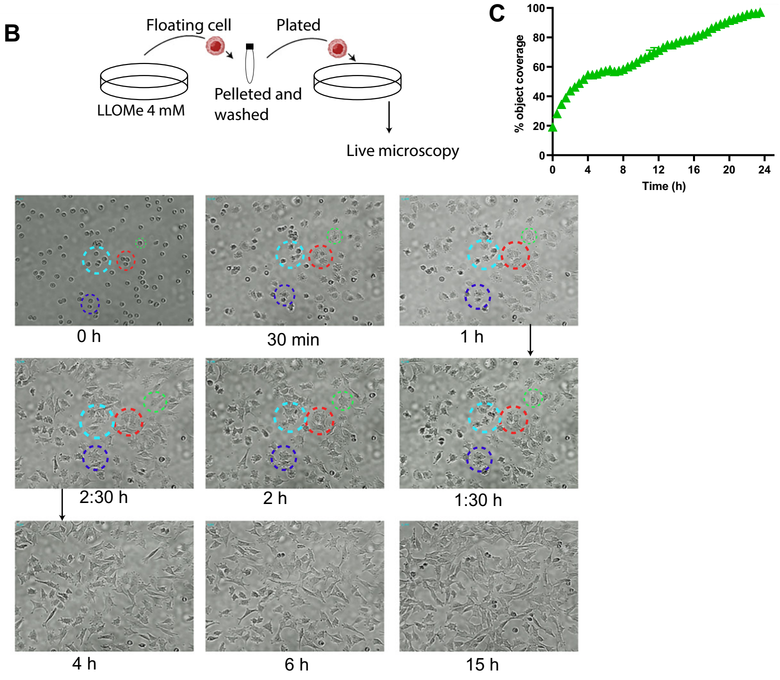

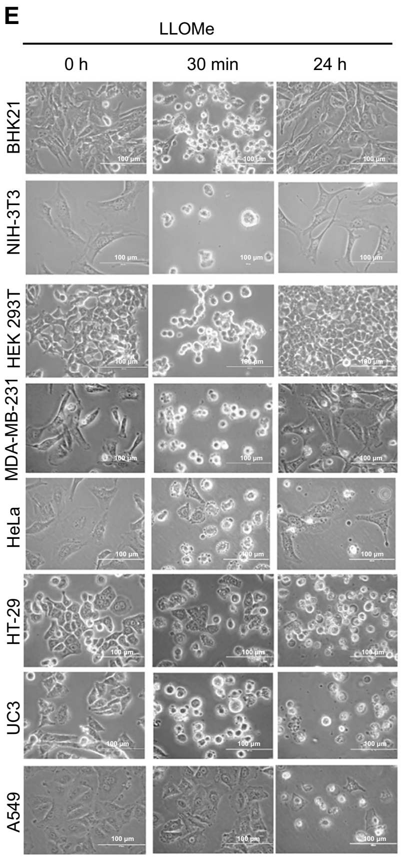

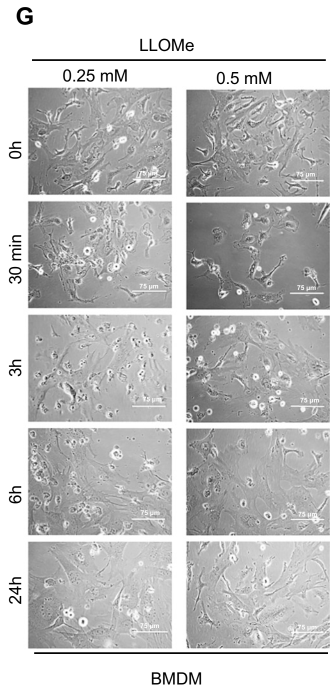

F

Cell Lines	LLOMe (Conc.)	Floating (30 Mins)	Recovery (24 h)
MEF	4mM	Yes	Yes (90%)
BHK21	8mM	Yes	Yes (90%)
NIH-3T3	4mM	Yes	Yes (90%)
MDAMB-231	4mM	Yes	Yes (90%)
HEK293T	4mM	Yes	Yes (90%)
HeLa	8mM	Rounded	Yes (50%)
HT-29	4mM	No	Cell Death
UC3	4mM	Yes	Cell Death
A549	8mM	No	Cell Death

 © The Author(s)

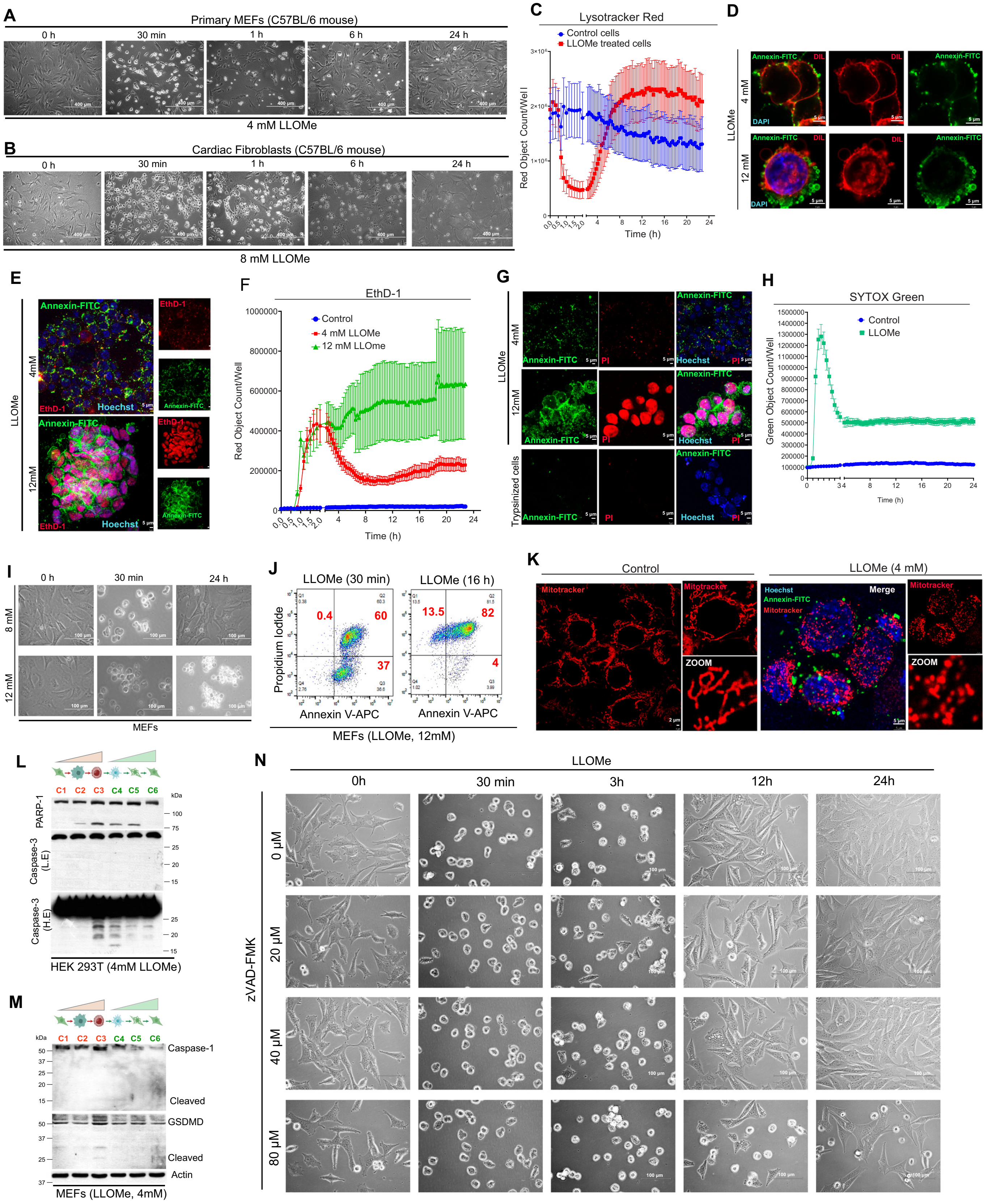
A
Primary MEFs (C57BL/6 mouse)
0 h
30 min
1 h
6 h
24 h
4 mM LLOMe
B
Cardiac Fibroblasts (C57BL/6 mouse)
8 mM LLOMe
C
Lysotracker Red
Control cells
LLOMe treated cells
Red Object Count/Well
Time (h)
D
Annexin-FITC
DIL
DAPI
LLOMe
4 mM
12 mM
E
EthD-1
Hoechst
F
EthD-1
Control
4 mM LLOMe
12 mM LLOMe
G
PI
Trypsinized cells
H
SYTOX Green
Control
LLOMe
Green Object Count/Well
I
MEFs
J
LLOMe (30 min)
LLOMe (16 h)
Propidium Iodide
Annexin V-APC
MEFs (LLOMe, 12mM)
K
Control
LLOMe (4 mM)
Mitotracker
ZOOM
Merge
L
PARP-1
Caspase-3 (L.E)
Caspase-3 (H.E)
HEK 293T (4mM LLOMe)
M
Caspase-1
Cleaved
GSDMD
Actin
MEFs (LLOMe, 4mM)
N
LLOMe
zVAD-FMK
0h
30 min
3h
12h
24h
0 μM
20 μM
40 μM
80 μM

◀ **Figure EV2. Revival from near-cell death.**

(**A**, **B**) Representative time-lapse live microscopy images of (**A**) primary MEF (C57BL/6 mouse) cells treated with 4 mM LLOMe and (**B**) primary cardiac fibroblast (C57BL/6 mouse) cells treated with 8 mM LLOMe. Magnification 10X. Scale Bar, 400 µm. (**C**) Time-lapse live microscopy of MEF cells stained with Lysotracker red (10 nM), washed, and then treated with 4 mM LLOMe, performed using IncuCyte S3. Graphs depict quantification of red objects (lysosomes-stained cells) per well, Mean ± SD, nine fields. (**D**, **E**) Representative live cell confocal microscopy images of MEF cells treated with LLOMe (4 or 12 mM) for 30 min and stained with (**D**) Annexin V-FITC (green), DIL (red) and DAPI (blue) (**E**) Annexin V-FITC (green), ethidium homodimer-1 (EthD-1) (red) and Hoechst (blue). Scale Bar, 5 µm. (**F**) IncuCyte time-lapse live microscopy of MEF cells stained with 2 µM EthD-1, washed and then treated with 4 mM LLOMe. Graphs depict quantification of red objects (lysosomes-stained cells) per well, mean ± SD, nine fields. Refer to Movie EV8. (**G**) Representative live cell confocal microscopy of MEF cells treated with LLOMe (4 or 12 mM) for 30 min and stained with Annexin V-FITC (green), and Propidium Iodide, PI (Red). Trypsinized floating cells were used as a control. Scale Bar, 5 µm. (**H**) Time-lapse live microscopy of MEF cells stained with 20 nM SYTOX Green and then treated with 4 mM LLOMe, performed using IncuCyte S3. Graphs depict quantification of green objects per well, mean ± SD, nine fields. Refer to Movie EV9. (**I**) Representative time-lapse live microscopy images of MEF cells treated with LLOMe (8 or 12 mM). Magnification 40X. Scale bar, 100 µm. (**J**) Representative flow cytometry analysis of MEF cells treated with 12 mM LLOMe for 30 min or 16 h and stained with Annexin V-APC and Propidium Iodide (PI). (**K**) Representative live cell confocal microscopy images of MEF cells, untreated or treated with 4 mM LLOMe for 30 min and stained with Annexin V-FITC (green), Mitotracker (red) and Hoechst (blue) as indicated. Scale Bar, 2 or 5 µm. Zoom panels show digital magnifications. (**L**, **M**) Western blot analysis with the lysate of (**L**) HEK293T cells with Caspase-3 and PARP-1, and (**M**) MEF cells with Caspase-1 and GSDMD antibodies at different time points. (**N**) Representative time-lapse live microscopy images of zVAD-FMK-treated MEF cells subjected to LLOMe (4 mM) treatment. Magnification 40X. Scale bar, 100 µm. Source data are available online for this figure.

 © The Author(s)

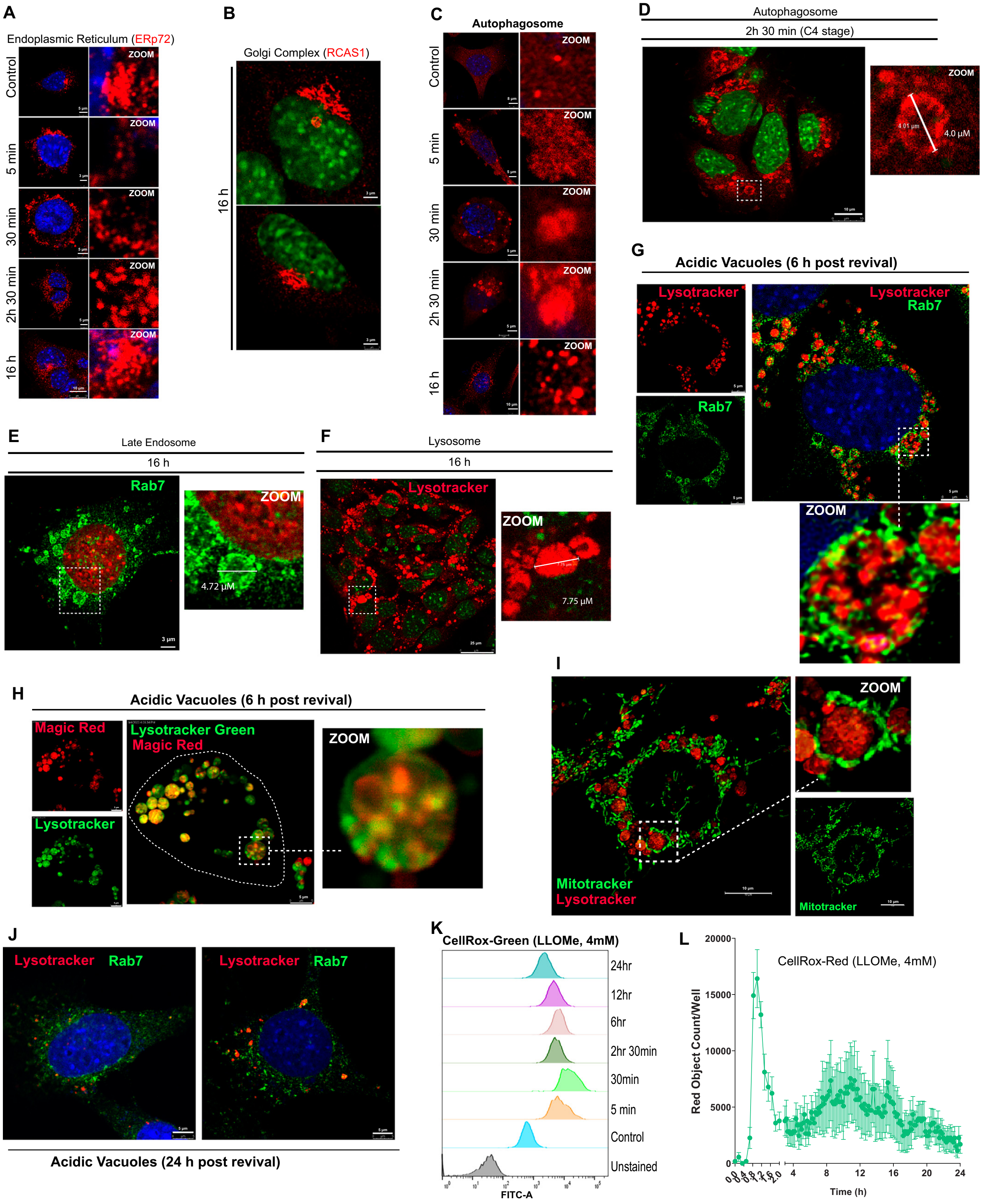

A
Endoplasmic Reticulum (ERp72)
Control
5 min
30 min
2h 30 min
16 h
ZOOM
B
Golgi Complex (RCAS1)
16 h
C
Autophagosome
D
Autophagosome
2h 30 min (C4 stage)
4.0 μM
E
Late Endosome
16 h
Rab7
4.72 μM
F
Lysosome
16 h
Lysotracker
7.75 μM
G
Acidic Vacuoles (6 h post revival)
Lysotracker
Rab7
H
Acidic Vacuoles (6 h post revival)
Magic Red
Lysotracker Green
Lysotracker
I
Mitotracker
Lysotracker
J
Lysotracker Rab7
Acidic Vacuoles (24 h post revival)
K
CellRox-Green (LLOMe, 4mM)
24hr
12hr
6hr
2hr 30min
30min
5 min
Control
Unstained
FITC-A
L
CellRox-Red (LLOMe, 4mM)
Red Object Count/Well
Time (h)

Figure EV3. Organelle dynamics during the cell death phase and revival.

(**A–C**) Representative confocal microscopy images of MEF cells treated with 4 mM LLOMe for the indicated time points and immunostained with (**A**) ERp72 (endoplasmic reticulum), (**B**) RCAS1 (Golgi complex), and (**C**) LC3B (autophagosome). The nucleus is stained with DAPI (pseudo-colored to green in (**B**) for better contrast). Zoom panels show digital magnifications. Scale Bar, 3–10 µm as indicated. (**D**) Representative confocal microscopy images of MEF cells treated with 4 mM LLOMe for 2 h 30 min (C4 stage) and immunostained with LC3B (autophagosome). The nucleus is stained with DAPI (pseudo-colored to green for better contrast). Zoom panels show digital magnifications. Scale Bar, 10 µm. (**E**, **F**) Representative confocal microscopy images of MEF cells treated with 4 mM LLOMe for 16 h and immunostained with (**E**) Rab7 or stained with (**F**) lysotracker red. The nucleus is stained with DAPI (pseudo-colored to red/green for better contrast). Zoom panels show digital magnifications. Scale Bar, 3 or 25 µm as indicated. (**G–I**) Representative confocal microscopy images of MEF cells treated with 4 mM LLOMe for 6 h and immunostained with (**G**) Rab7 (late endosomes) and stained with lysotracker red or (**H**) stained with magic red and lysotracker green or (**I**) stained with mitotracker green and lysotracker red. Zoom panels show digital magnifications. Scale Bar, 5 or 10 µm as indicated. (**J**) Representative confocal microscopy images of MEF cells treated with 4 mM LLOMe for 24 h post revival and stained with lysotracker red and immunostained with anti-Rab7 (late endosomes). The nucleus is stained with DAPI. Scale Bar, 5 µm. (**K**) Flow cytometry analysis of ROS production using CellROX Green in MEF cells treated with 4 mM LLOMe at the indicated time points. (**L**) Time-lapse live microscopy using IncuCyte of MEF cells stained with CellROX-red and then treated with 4 mM LLOMe. Graphs depict quantification of red object count per well, mean ± SD, nine fields. Source data are available online for this figure.

 © The Author(s)

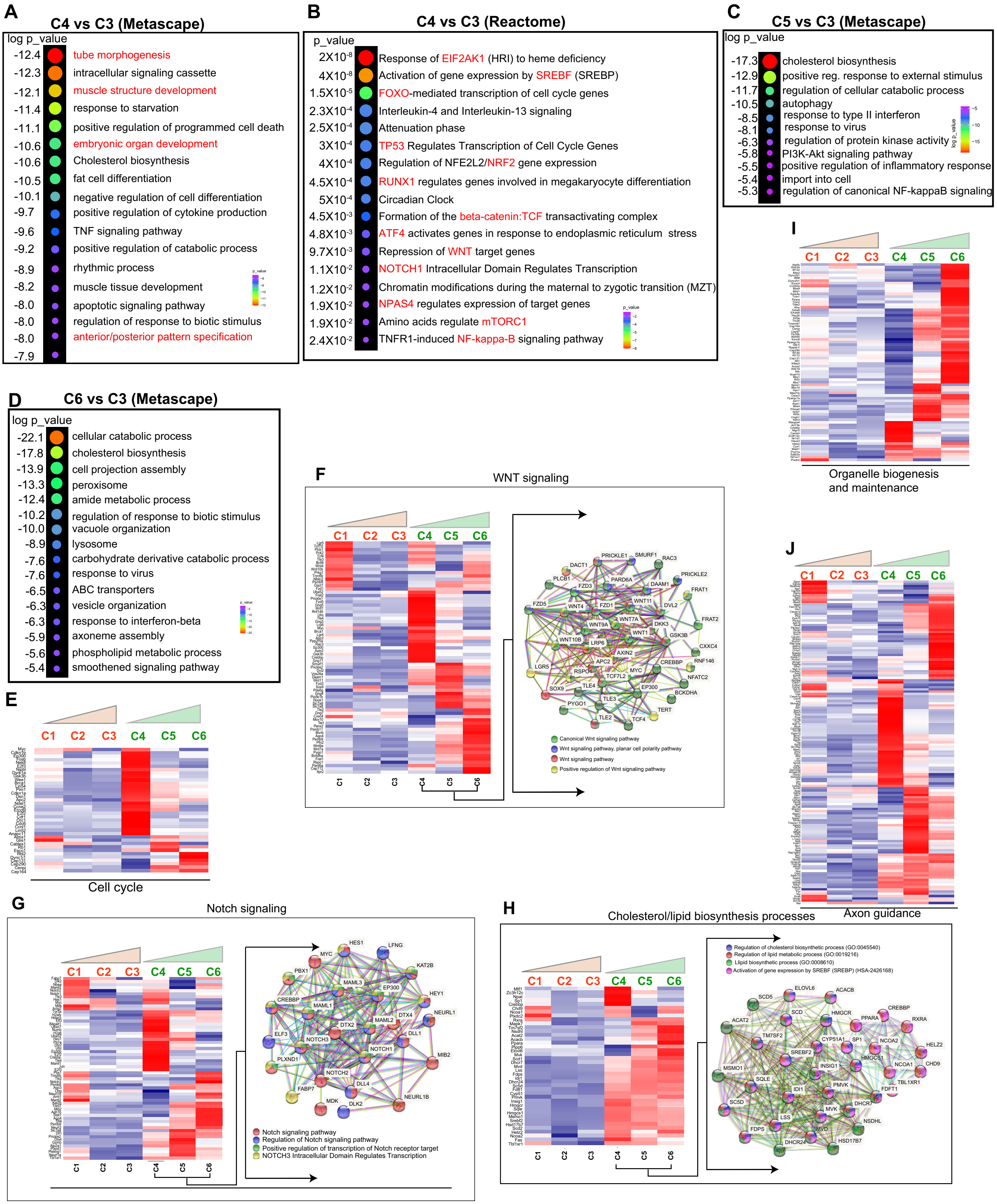
A
C4 vs C3 (Metascape)
log p_value
-12.4 tube morphogenesis
-12.3 intracellular signaling cassette
-12.1 muscle structure development
-11.4 response to starvation
-11.1 positive regulation of programmed cell death
-10.6 embryonic organ development
-10.6 Cholesterol biosynthesis
-10.5 fat cell differentiation
-10.1 negative regulation of cell differentiation
-9.7 positive regulation of cytokine production
-9.6 TNF signaling pathway
-9.2 positive regulation of catabolic process
-8.9 rhythmic process
-8.2 muscle tissue development
-8.0 apoptotic signaling pathway
-8.0 regulation of response to biotic stimulus
-8.0 anterior/posterior pattern specification
-7.9
B
C4 vs C3 (Reactome)
p_value
2X10-8 Response of EIF2AK1 (HRI) to heme deficiency
4X10-8 Activation of gene expression by SREBF (SREBP)
1.5X10-5 FOXO-mediated transcription of cell cycle genes
2.3X10-4 Interleukin-4 and Interleukin-13 signaling
2.5X10-4 Attenuation phase
3X10-4 TP53 Regulates Transcription of Cell Cycle Genes
4X10-4 Regulation of NFE2L2/NRF2 gene expression
4.5X10-4 RUNX1 regulates genes involved in megakaryocyte differentiation
5X10-4 Circadian Clock
4.5X10-3 Formation of the beta-catenin:TCF transactivating complex
4.8X10-3 ATF4 activates genes in response to endoplasmic reticulum stress
9.7X10-3 Repression of WNT target genes
1.1X10-2 NOTCH1 Intracellular Domain Regulates Transcription
1.2X10-2 Chromatin modifications during the maternal to zygotic transition (MZT)
1.9X10-2 NPAS4 regulates expression of target genes
1.9X10-2 Amino acids regulate mTORC1
2.4X10-2 TNFR1-induced NF-kappa-B signaling pathway
C
C5 vs C3 (Metascape)
log p_value
-17.3 cholesterol biosynthesis
-12.9 positive reg. response to external stimulus
-11.7 regulation of cellular catabolic process
-10.5 autophagy
-8.5 response to type II interferon
-8.1 response to virus
-6.3 regulation of protein kinase activity
-5.8 PI3K-Akt signaling pathway
-5.5 positive regulation of inflammatory response
-5.4 import into cell
-5.3 regulation of canonical NF-kappaB signaling
D
C6 vs C3 (Metascape)
log p_value
-22.1 cellular catabolic process
-17.8 cholesterol biosynthesis
-13.9 cell projection assembly
-13.3 peroxisome
-12.4 amide metabolic process
-10.2 regulation of response to biotic stimulus
-10.0 vacuole organization
-8.9 lysosome
-7.6 carbohydrate derivative catabolic process
-7.6 response to virus
-6.5 ABC transporters
-6.3 vesicle organization
-6.3 response to interferon-beta
-5.9 axoneme assembly
-5.6 phospholipid metabolic process
-5.4 smoothened signaling pathway
E
C1 C2 C3 C4 C5 C6
Cell cycle
F
WNT signaling
C1 C2 C3 C4 C5 C6
Canonical Wnt signaling pathway
Wnt signaling pathway, planar cell polarity pathway
Wnt signaling pathway
Positive regulation of Wnt signaling pathway
G
Notch signaling
C1 C2 C3 C4 C5 C6
Notch signaling pathway
Regulation of Notch signaling pathway
Positive regulation of transcription of Notch receptor target
NOTCH3 Intracellular Domain Regulates Transcription
H
Cholesterol/lipid biosynthesis processes
C1 C2 C3 C4 C5 C6
Regulation of cholesterol biosynthetic process (GO:0045540)
Regulation of lipid metabolic process (GO:0019216)
Lipid biosynthetic process (GO:0008610)
Activation of gene expression by SREBF (SREBP) (HSA-2426168)
I
C1 C2 C3 C4 C5 C6
Organelle biogenesis and maintenance
J
C1 C2 C3 C4 C5 C6
Axon guidance

Figure EV4. RNA-sequencing analysis of cell death and revival phase.

(**A–D**) Bubble plots depict top pathways from metascape (**A**, **C**, **D**) and Reactome (**B**) pathway analysis performed with genes upregulated ($p_{adj} < 0.05$, >1.5 folds, $n = 3$, Wald chi-squared test) in C4 or C5 or C6 stage as compared to C3 stage. (**E–J**) Heatmap generated for genes representing GO terms (**E**) Cell Cycle (**F**) WNT Signaling, (**G**) NOTCH signaling, (**H**) Cholesterol/lipid biosynthesis processes, (**I**) Organelle biogenesis and maintenance, (**J**) Axon guidance induced ($p < 0.05$, >1.5 folds, ≤0.6 folds, base mean >10, $n = 3$, Wald chi-squared test) across C1 to C6 stage. STRING analysis of core genes of the pathway is also depicted.

© The Author(s)

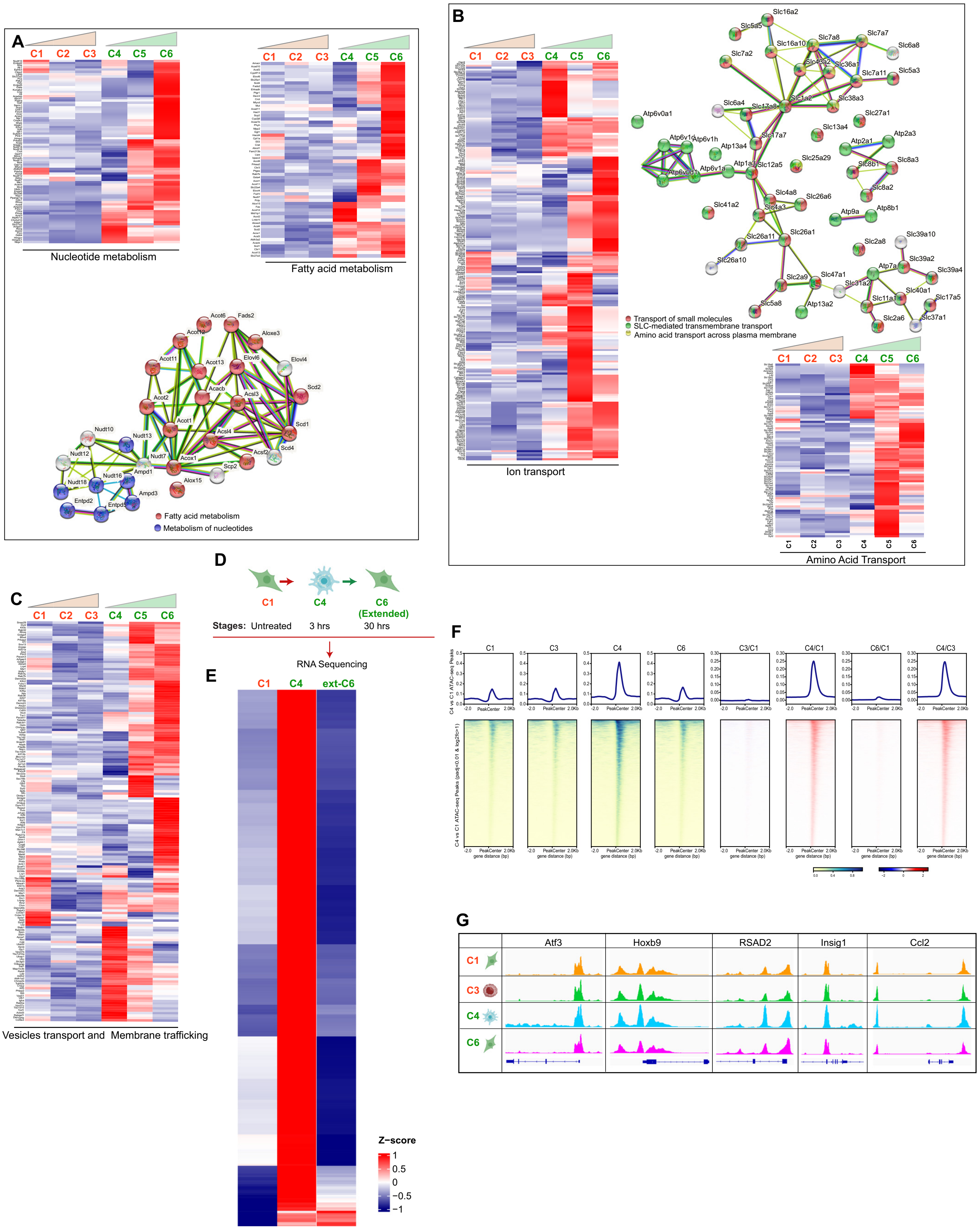
A
C1 C2 C3 C4 C5 C6
Nucleotide metabolism
Fatty acid metabolism
Fatty acid metabolism
Metabolism of nucleotides
B
Ion transport
Transport of small molecules
SLC-mediated transmembrane transport
Amino acid transport across plasma membrane
Amino Acid Transport
C
Vesicles transport and Membrane trafficking
D
C1 C4 C6 (Extended)
Stages: Untreated 3 hrs 30 hrs
RNA Sequencing
E
C1 C4 ext-C6
Z-score
F
C1 C3 C4 C6 C3/C1 C4/C1 C6/C1 C4/C3
G
Atf3 Hoxb9 RSAD2 Insig1 Ccl2
C1 C3 C4 C6

Figure EV5. Pathways of programmed cell revival.

(**A–C**) Heatmap generated for genes ($p < 0.05$, >1.5 folds, base mean >10, $n = 3$, Wald chi-squared test) representing GO terms (**A**) Nucleotide and fatty acid metabolism. (**B**) Ion transport and amino acid transport. (**C**) Vesicles transport and membrane trafficking across different stages. (**D**) RNA sequencing experiment is performed with MEF cells treated with 4 mM LLOMe at the indicated three time points (C1, C4, and extended-C6) with three biological replicates. (**E**) The genes induced in the C4 stage ($p < 0.05$, >1.5 fold, see Dataset EV7) compared to the C1 stage was plotted across all three stages (C1, C4, and extended-C6). (**F**) The heatmaps represent the ATAC-seq signal intensities at loci with increased accessibility at C4 compared to C1 (padj<0.01, log2 foldchange >2) at different stages of 4 mM LLOMe treatment to MEF cells (C1, C3, C4, C6, C3/C1, C4/C1, C4/C3, and C6/C1). (The heatmaps represent a 4 kb window centered on the peak midpoint, and sorted based on the C4 enrichment compared to C1). (**G**) Genome browser screenshots of five selected genomic regions' signals in C1, C3, C4, and C6. (The blue bar at the bottom indicates the reference sequence (mm10) of the selected genomic regions).

© The Author(s)

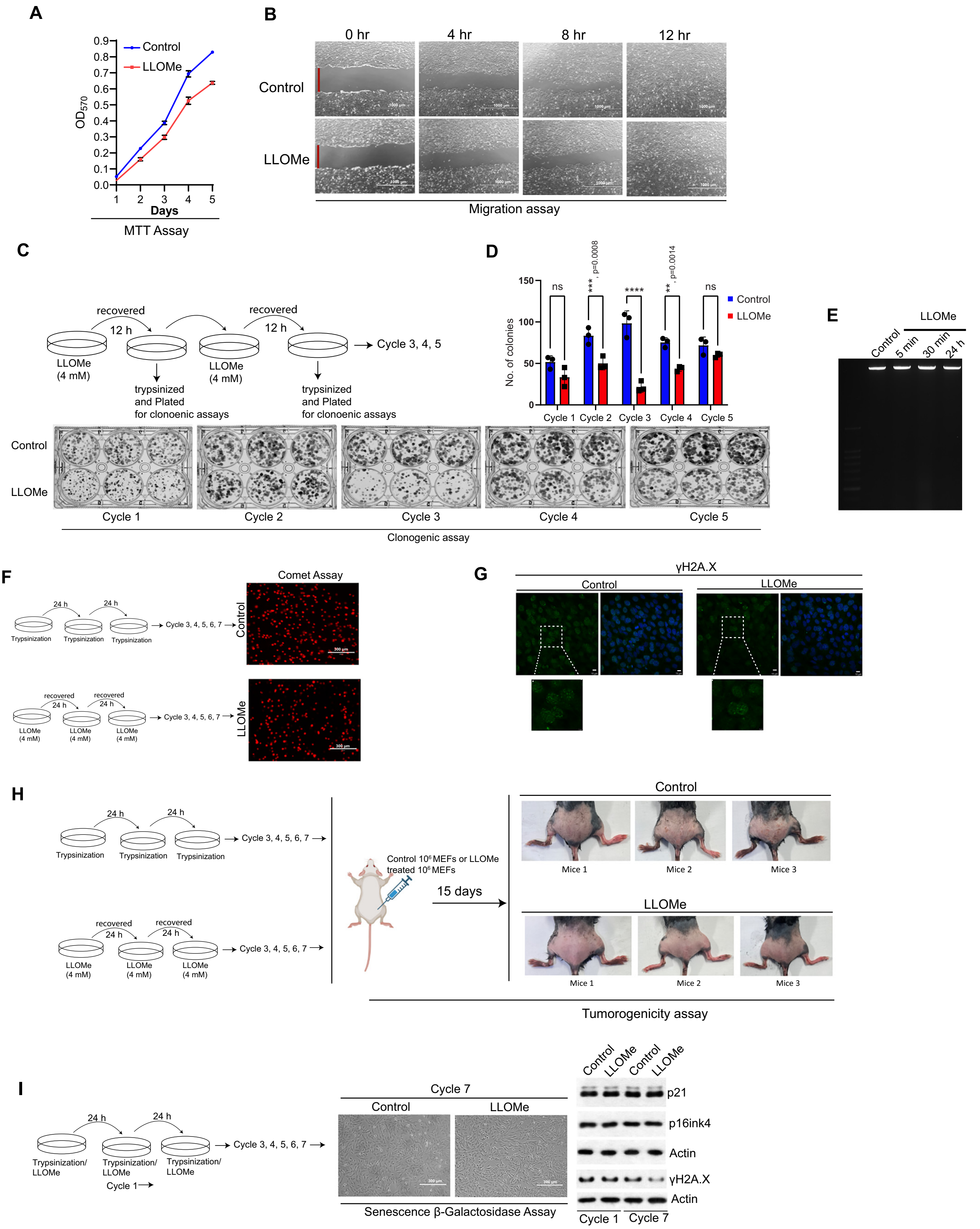
A
Control
LLOMe
OD570
Days
MTT Assay
B
0 hr
4 hr
8 hr
12 hr
Control
LLOMe
Migration assay
C
recovered
12 h
LLOMe (4 mM)
trypsinized and Plated for clonoenic assays
Cycle 3, 4, 5
Control
LLOMe
Cycle 1
Cycle 2
Cycle 3
Cycle 4
Cycle 5
Clonogenic assay
D
No. of colonies
ns
***, p=0.0008
****
**, p=0.0014
ns
Control
LLOMe
E
LLOMe
Control
5 min
30 min
24 h
F
Comet Assay
24 h
Trypsinization
Cycle 3, 4, 5, 6, 7
Control
recovered
LLOMe (4 mM)
LLOMe
G
γH2A.X
Control
LLOMe
H
Control 10⁶ MEFs or LLOMe treated 10⁶ MEFs
15 days
Mice 1
Mice 2
Mice 3
Tumorogenicity assay
I
Trypsinization/ LLOMe
Cycle 1
Cycle 7
Senescence β-Galactosidase Assay
p21
p16ink4
Actin
γH2A.X

Figure EV6. LLOMe treatment does not make cells tumorigenic.

(**A**) Analysis of cell proliferation using MTT assays with MEF cells untreated or treated with 4 mM LLOMe for the indicated time points. (three technical replicates, mean ± SD). (**B**) Representative time-lapse live microscopy images of migration assays performed with control or 4 mM LLOMe-treated MEF cells at the indicated time points. Magnification 4X. Scale bar, 1000 µm. (**C**, **D**) Schematic representation of the experimental design where the same cells were given five cycles of 4 mM LLOMe treatment and plated for (**C**) clonogenic assays in each cycle. (**D**) Graph depicts the number of colonies from clonogenic assays ($n = 3$, mean ± SD, $^{**}p = 0.0014$, $^{***}p = 0.0008$, $^{****}p < 0.0001$, ns non-significant $p > 0.05$, unpaired t-test). (**E**) Agarose gel image for genomic DNA isolated from control and 4 mM LLOMe-treated MEF cells for the indicated time points. (**F**) Comet assays were performed with MEF cells that were subjected to seven cycles of trypsinization (control) or LLOMe treatment. (**G**) Immunofluorescence experiments using γH2A.X antibody were performed with MEF cells that were subjected to seven cycles of trypsinization (control) or LLOMe treatment. (**H**) MEF cells that were subjected to seven cycles of trypsinization (control) or LLOMe treatment were injected into the flanks of mice ($n = 6$). (**I**) MEF cells that were exposed to seven cycles of trypsinization (control) or LLOMe treatment were subjected to senescence-associated β-galactosidase assays or Western blot with p21, p16ink4, and γH2A.X antibody. Source data are available online for this figure.

 © The Author(s)

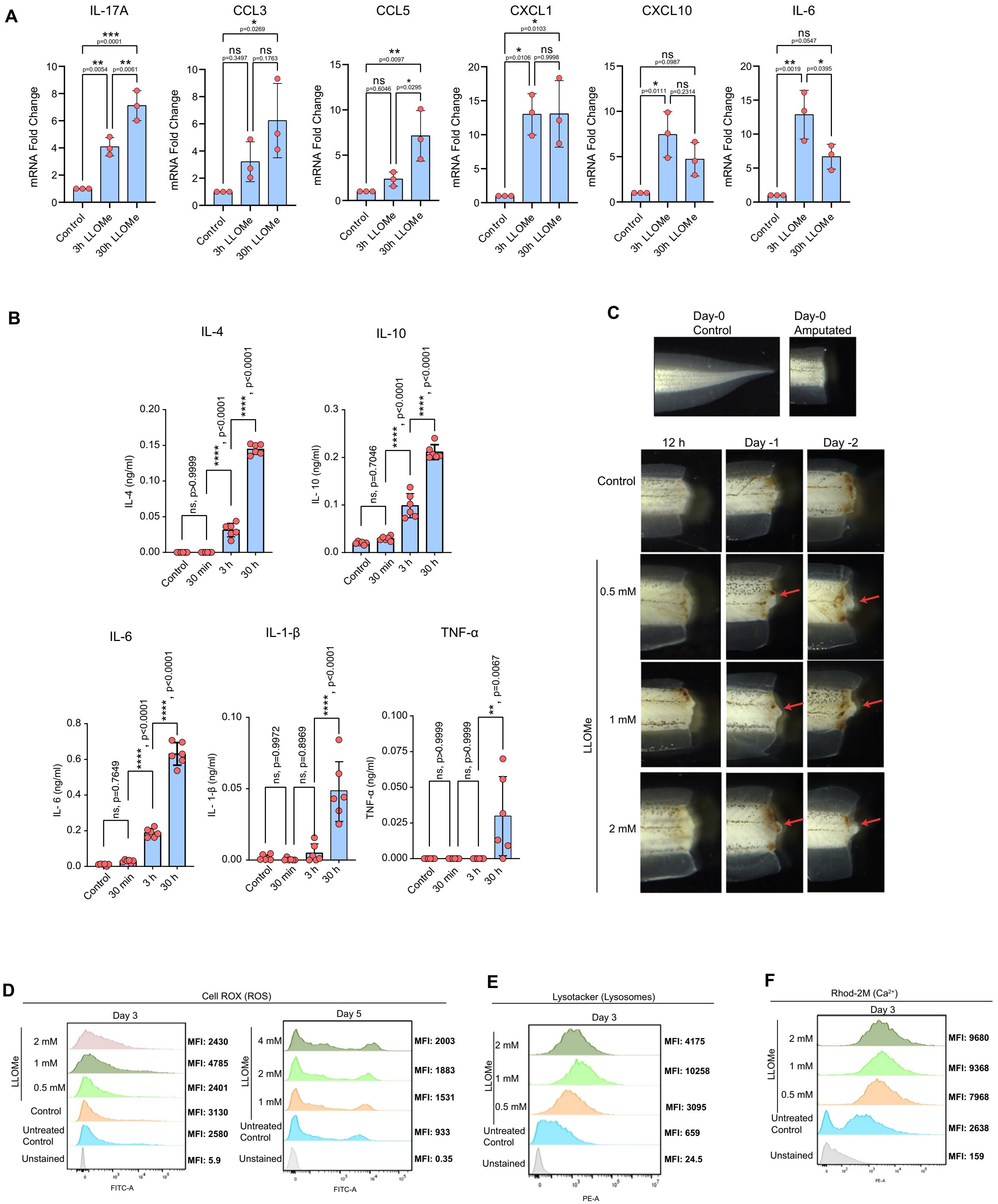
A
IL-17A
CCL3
CCL5
CXCL1
CXCL10
IL-6
mRNA Fold Change
Control
3h LLOMe
30h LLOMe
*** p=0.0001
** p=0.0054
** p=0.0061
* p=0.0269
ns p=0.3497
ns p=0.1763
** p=0.0097
ns p=0.6046
* p=0.0295
* p=0.0103
* p=0.0106
ns p=0.9998
ns p=0.0987
* p=0.0111
ns p=0.2314
ns p=0.0547
** p=0.0019
* p=0.0395
B
IL-4
IL-10
IL-4 (ng/ml)
IL- 10 (ng/ml)
ns, p>0.9999
****, p<0.0001
ns, p=0.7046
Control
30 min
3 h
30 h
IL-6
IL-1-β
TNF-α
IL- 6 (ng/ml)
IL- 1-β (ng/ml)
TNF-α (ng/ml)
ns, p=0.7649
ns, p=0.9972
ns, p=0.8969
ns, p>0.9999
**, p=0.0067
C
Day-0 Control
Day-0 Amputated
12 h
Day -1
Day -2
Control
LLOMe
0.5 mM
1 mM
2 mM
D
Cell ROX (ROS)
Day 3
Day 5
LLOMe
2 mM
1 mM
0.5 mM
4 mM
Control
Untreated Control
Unstained
MFI: 2430
MFI: 4785
MFI: 2401
MFI: 3130
MFI: 2580
MFI: 5.9
MFI: 2003
MFI: 1883
MFI: 1531
MFI: 933
MFI: 0.35
FITC-A
E
Lysotacker (Lysosomes)
Day 3
MFI: 4175
MFI: 10258
MFI: 3095
MFI: 659
MFI: 24.5
PE-A
F
Rhod-2M (Ca2+)
Day 3
MFI: 9680
MFI: 9368
MFI: 7968
MFI: 2638
MFI: 159
PE-A

Figure EV7. LLOMe induces wound healing and tissue regeneration.

(**A**) A qRT-PCR analysis of MEF cells treated with LLOMe for different time points as indicated in the figure ($n = 3$, mean ± SD, *$p < 0.05$, **$p < 0.01$, ***$p < 0.001$, ns non-significant $p > 0.05$, ordinary one-way ANOVA, Tukey's multiple comparison test). Exact *p* values are depicted in the Figure and Appendix Table S2. (**B**) ELISA with supernatant of the MEF cells treated with LLOMe for different time points as indicated in the figure ($n = 3$, Mean ± SD, **$p = 0.0067$, ****$p < 0.0001$, ns non-significant $p > 0.05$, ordinary one-way ANOVA, Tukey's multiple comparison test). Exact *p* values are depicted in the Figure and Appendix Table S2. (**C**) Top panel, representative images of a control tadpole tail and amputated tail on day 0. Bottom panel, representative images of untreated or LLOMe-treated amputated tails at the indicated time points. The red arrow indicates blastema formation. (**D–F**) Flow cytometry analysis of (**D**) ROS production using CellROX Green, (**E**) lysosomes numbers using lysotracker, and (**F**) mitochondrial calcium using Rhod-2 AM in untreated and 4 mM LLOMe-treated regenerated tissue of tadpole at the indicated time points. Source data are available online for this figure.

 © The Author(s)
