# Supplementary material for: Programmed cell revival from imminent cell death enhances tissue repair and regeneration
Source: EMBO J. 2025 Aug 21;44(19):5244–89. doi: 10.1038/s44318-025-00540-y (PMC12489119; doi:10.1038/s44318-025-00540-y)
Supplement: Supplementary file 13 — Movie EV3 [file 44318_2025_540_MOESM13_ESM.zip › Movie EV3/Movie EV3.doc]

**Movie EV3: Cells revive from apoptosis-like conditions. (Related to Figure 1)**

Live imaging of MEF cells treated with 4 mM LLOMe
