# Supplementary material for: Programmed cell revival from imminent cell death enhances tissue repair and regeneration
Source: EMBO J. 2025 Aug 21;44(19):5244–89. doi: 10.1038/s44318-025-00540-y (PMC12489119; doi:10.1038/s44318-025-00540-y)
Supplement: Supplementary file 14 — Movie EV4 [file 44318_2025_540_MOESM14_ESM.zip › Movie EV4/Movie EV4.doc]

**Movie EV4: Revival from near-cell death. (Related to Figure 1 and EV1, 2)**

MEF cells that were treated with 4 mM LLOMe. The floating cells were collected, washed, and replated in a new culture plate. Live imaging of revival from cell death phase.
