# Supplementary material for: Programmed cell revival from imminent cell death enhances tissue repair and regeneration
Source: EMBO J. 2025 Aug 21;44(19):5244–89. doi: 10.1038/s44318-025-00540-y (PMC12489119; doi:10.1038/s44318-025-00540-y)
Supplement: Supplementary file 15 — Movie EV5 [file 44318_2025_540_MOESM15_ESM.zip › Movie EV5/Movie EV5.doc]

**Movie EV5: Resuscitation from cell death phase. (Related to Figure 1 and EV1, 2)**

Live imaging of cells treated with LLOMe (4 mM) or GPN (200 µM) captured using IncuCyte S3 system.
