# Supplementary material for: Programmed cell revival from imminent cell death enhances tissue repair and regeneration
Source: EMBO J. 2025 Aug 21;44(19):5244–89. doi: 10.1038/s44318-025-00540-y (PMC12489119; doi:10.1038/s44318-025-00540-y)
Supplement: Supplementary file 16 — Movie EV6 [file 44318_2025_540_MOESM16_ESM.zip › Movie EV6/Movie EV6.doc]

**Movie EV6: Cells resuscitate from cell death-like conditions. (Related to Figure 1 and EV1, 2)**

Live imaging of cells treated with LLOMe (4 mM) or sphingosine (15 µM) captured using the IncuCyte S3 system.
