# Supplementary material for: Programmed cell revival from imminent cell death enhances tissue repair and regeneration
Source: EMBO J. 2025 Aug 21;44(19):5244–89. doi: 10.1038/s44318-025-00540-y (PMC12489119; doi:10.1038/s44318-025-00540-y)
Supplement: Supplementary file 17 — Movie EV7 [file 44318_2025_540_MOESM17_ESM.zip › Movie EV7/Movie EV7.doc]

**Movie EV7: Live cell imaging of lysotracker-stained cells treated with LLOMe. (Related to Figure 1, 2)**

Live imaging of lysotracker-stained cells treated with LLOMe (4 mM)
