# Supplementary material for: Programmed cell revival from imminent cell death enhances tissue repair and regeneration
Source: EMBO J. 2025 Aug 21;44(19):5244–89. doi: 10.1038/s44318-025-00540-y (PMC12489119; doi:10.1038/s44318-025-00540-y)
Supplement: Supplementary file 19 — Movie EV9 [file 44318_2025_540_MOESM19_ESM.zip › Movie EV9/Movie EV9.doc]

**Movie EV9: Live cell imaging of SYTOX green-stained cells treated with LLOMe. (Related to Figure 1)**

Live imaging of SYTOX green-stained cells treated with 4mM and 12 mM LLOMe.
