# Supplementary material for: Programmed cell revival from imminent cell death enhances tissue repair and regeneration
Source: EMBO J. 2025 Aug 21;44(19):5244–89. doi: 10.1038/s44318-025-00540-y (PMC12489119; doi:10.1038/s44318-025-00540-y)
Supplement: Supplementary file 20 — Movie EV10 [file 44318_2025_540_MOESM20_ESM.zip › Movie EV10/Movie EV10.doc]

**Movie EV10: Live cell imaging of LLOMe treated cells . (Related to Figure 1)**

Live imaging of cells treated with 4mM, 8 mM, and 12 mM of LLOMe.
