# Supplementary material for: Programmed cell revival from imminent cell death enhances tissue repair and regeneration
Source: EMBO J. 2025 Aug 21;44(19):5244–89. doi: 10.1038/s44318-025-00540-y (PMC12489119; doi:10.1038/s44318-025-00540-y)
Supplement: Supplementary file 22 — Movie EV12 [file 44318_2025_540_MOESM22_ESM.zip › Movie EV12/Movie EV12.doc]

**Movie EV12: Caspase activation during the cell death phase and resurrection.** Live microscopy of HEK293T cells transfected with ZipGFP-based caspase reporter (constitutively expresses mCherry) plasmids and treated with 4 mM LLOMe. Control cells are similarly transfected with the reporter plasmid.
