# Supplementary material for: Programmed cell revival from imminent cell death enhances tissue repair and regeneration
Source: EMBO J. 2025 Aug 21;44(19):5244–89. doi: 10.1038/s44318-025-00540-y (PMC12489119; doi:10.1038/s44318-025-00540-y)
Supplement: Supplementary file 24 — Movie EV14 [file 44318_2025_540_MOESM24_ESM.zip › Movie EV14/Movie EV14.doc]

**Movie EV14: Organelle dynamics during cell death and resuscitation phase. (Related to Figure 2)**

Time-lapse imaging of Lysotracker red and Mitotracker green stained MEF cells and treated with 4 mM LLOMe.
