# Supplementary material for: Programmed cell revival from imminent cell death enhances tissue repair and regeneration
Source: EMBO J. 2025 Aug 21;44(19):5244–89. doi: 10.1038/s44318-025-00540-y (PMC12489119; doi:10.1038/s44318-025-00540-y)
Supplement: Supplementary file 25 — Movie EV15 [file 44318_2025_540_MOESM25_ESM.zip › Movie EV15/Movie EV15.doc]

**Movie EV15: The pathways/genes critical for programmed cell revival. (Related to Figure 5)**

Time-lapse imaging of MEF cells treated with 4 mM LLOMe and BAPTA-AM.
